# DNA Damage Checkpoints Govern Global Gene Transcription and Exhibit Species-Specific Regulation on *HOF1* in *Candida albicans*

**DOI:** 10.3390/jof10060387

**Published:** 2024-05-29

**Authors:** Yan Zhang, Huaxin Cai, Runlu Chen, Jinrong Feng

**Affiliations:** Department of Pathogen Biology, School of Medicine, Nantong University, Nantong 226007, China; zy720189@163.com (Y.Z.); caihuaxin2023@126.com (H.C.); chenrunlu@126.com (R.C.)

**Keywords:** DNA damage response, methyl methanesulfonate, RNA-seq, Rad53, Hof1, *Candida albicans*

## Abstract

DNA damage checkpoints are essential for coordinating cell cycle arrest and gene transcription during DNA damage response. Exploring the targets of checkpoint kinases in *Saccharomyces cerevisiae* and other fungi has expanded our comprehension of the downstream pathways involved in DNA damage response. While the function of checkpoint kinases, specifically Rad53, is well documented in the fungal pathogen *Candida albicans*, their targets remain poorly understood. In this study, we explored the impact of deleting *RAD53* on the global transcription profiles and observed alterations in genes associated with ribosome biogenesis, DNA replication, and cell cycle. However, the deletion of *RAD53* only affected a limited number of known DNA damage-responsive genes, including *MRV6* and *HMX1*. Unlike *S. cerevisiae*, the downregulation of *HOF1* transcription in *C. albicans* under the influence of Methyl Methanesulfonate (MMS) did not depend on Dun1 but still relied on Rad53 and Rad9. In addition, the transcription factor Mcm1 was identified as a regulator of *HOF1* transcription, with evidence of dynamic binding to its promoter region; however, this dynamic binding was interrupted following the deletion of *RAD53*. Furthermore, Rad53 was observed to directly interact with the promoter region of *HOF1*, thus suggesting a potential role in governing its transcription. Overall, checkpoints regulate global gene transcription in *C. albicans* and show species-specific regulation on *HOF1*; these discoveries improve our understanding of the signaling pathway related to checkpoints in this pathogen.

## 1. Introduction

DNA damage, caused by intrinsic and extrinsic factors, is unavoidable in cells but could be lethal if not adequately repaired. To address this potentially fatal event, cells detect and repair damaged DNA through an intricate and precise mechanism referred to as the DNA damage response (DDR) [1,2]. Among the components of the DDR, DNA damage checkpoints are considered pivotal hubs for coordinating cell cycle arrest and gene transcription to facilitate the repair process [3]. Upon the detection of damaged DNA signals, checkpoint kinases are activated through a cascade of phosphorylation events to halt the cell cycle progression and prevent the transmission of damaged DNA to daughter cells [2]. In *Saccharomyces cerevisiae*, Mec1 and Tel1 have been identified as two primary sensor kinases that function with partial redundancy in sensing DNA damage and initiating its repair [4,5]. Once activated, these sensors transmit the signal of DNA damage to downstream effector kinases Rad53 or Chk1, with the involvement of adaptors Rad9 or Mrc1 [5,6,7]. Activated Rad53 further regulates the transcription of DNA damage response genes and dNTP pools directly or in a Dun1-dependent manner [2,8,9]. Understanding the cellular response to diverse types of DNA damage stresses has dramatically enriched our understanding of the DNA damage repair process across various model organisms. Particularly in *S. cerevisiae*, numerous transcription factors function downstream from checkpoint kinases Rad53 and Dun1 [10]. For instance, multiple nucleic acid metabolism genes involved in DNA replication and repair are regulated by the transcription factor complexes MBF (Swi6–Mbp1) and SBF (Swi6–Swi4); this regulation depends on Rad53 but not its downstream kinase Dun1 [10]. In the pathogenic fungus *Cryptococcus neoformans*, the transcription of DNA damage repair genes *RAD51*, *RAD54*, and *RDH54* is induced by gamma radiation; however, the induction is significantly inhibited by *RAD53* deletion rather than *CHK2*, thus suggesting checkpoint-specific regulation [11].

Emerging evidence suggests that most genes responding to DNA damage are not directly involved in DNA repair but instead participate in various indirect processes such as cell cycle progression, stress response, protein homeostasis, and energy metabolism [12]. In addition, checkpoint kinases play a role in regulating the global gene transcription to facilitate DNA repair. In *S. cerevisiae*, the primary checkpoint effector kinase Rad53 governs the transcription of genes related to the vacuolar protein catabolic process by modulating the activity of transcription factor Msn4 in response to methyl methanesulfonate (MMS) [10]. Moreover, Rad53 regulates the transcription of genes associated with cellular amino acids and the derivative metabolic process through transcription factor Gcn4, as well as genes involved in cell division via the Ndd1/Mcm1/Fkh2 complex; these actions are dependent on downstream kinase Dun1 [10]. Specifically, Hof1, an F-BAR protein, is essential for regulating actin cytoskeleton organization and cytokinesis, and it exhibits decreased expression after MMS exposure in *S. cerevisiae*. Notably, this reduction can be blocked by deleting the *DUN1* gene, thus highlighting its role in checkpoint-mediated transcriptional control [10]. Furthermore, the regulation of *HOF1* and other cell cycle elements depends on the Ndd1/Mcm1/Fkh2 complex, with Ndd1 serving as a coactivator for transcription [13]. Similarly, MMS stress suppresses *HOF1* expression in the pathogenic yeast *Candida albicans*, which is an effect that can be reversed by *RAD53* deletion [14]. However, the regulatory mechanism by which Rad53 governs *HOF1* and other cell cycle genes remains unclear in *C. albicans*, primarily due to the lack of a known orthologous counterpart for Ndd1.

Checkpoint kinases regulate global cellular processes, including the virulence of various pathogens. In *Cryptococcus neoformans*, Rad53 is phosphorylated by the two phosphatidylinositol 3-kinase (PI3K)-like kinases Tel1 and Mec1, and it governs the expression of DNA damage repair genes in response to gamma radiation [11]. The perturbation of *RAD53* attenuates the virulence of *C. neoformans* and increases susceptibility to specific antifungal drugs such as amphotericin B [11]. In *Candida glabrata*, the overexpression of *RAD53* reduces biofilm formation and enhances biofilm susceptibility to fluconazole under hypoxia [15], while the checkpoint kinase Chk1 is crucial for the virulence of the plant pathogen *Ustilago maydis* [16]. In *C. albicans*, DNA damage repair genes like *RTT109* and *RAD23* are implicated in virulence; however, the role of checkpoints in virulence remains uncertain [17,18].

In our previous study, we revealed the transcriptional response to MMS-induced DNA damage stress in *C. albicans* and identified a set of MMS-responsive genes [12]. Nevertheless, the regulatory mechanisms governing these potential DNA damage-responsive genes during DNA damage responses remain unclear. Profiling checkpoint-related transcription is crucial for defining the essential DNA damage response genes. To address this, we employed RNA sequencing assays to elucidate the transcriptional consequences of *RAD53* deletion in *C. albicans*. We discovered that Rad53 potentially regulates ribosome biogenesis, the cell cycle, and DNA replication circuits in *C. albicans*. Additionally, we noted the specific mechanism of the Rad53- and Rad9-mediated transcriptional regulation of *HOF1* in *C. albicans*; this pattern differed from that seen in *S. cerevisiae*, as the expression of *HOF1* in *C. albicans* was independent of Dun1. Furthermore, our findings demonstrate that checkpoint kinase Rad53 directly interacts with the promoter region of *HOF1* and regulates the dynamic binding of Mcm1 to its promoter region. Overall, our study provides valuable insights into understanding checkpoint-dependent transcriptional regulation during DNA damage response in *C. albicans*, thus highlighting a species-specific regulation on *HOF1*.

## 2. Materials and Methods

### 2.1. Strains, Media, and Reagents

*C. albicans* strains were cultured in YPD media supplemented with 50 mg/L uridine, as previously described [19]. Deletion strains were selected on synthetic complete (SC) media lacking uridine, histidine, or arginine. To inhibit the *MET3* promoter, strains carrying this promoter were cultured in liquid SC media supplemented with 5 mM methionine and cysteine. Strains and primers used in this study are listed in Tables S1 and S2, respectively. Methyl methanesulfonate (MMS) was purchased from Sigma (St. Louis, MO, USA). Digitonin was purchased from Meryer (Shanghai, China). Additional reagents and amino acids for making media were acquired from Sangon (Shanghai, China). Solid media consisted of 2% agar.

### 2.2. DNA Manipulation

To construct the *DUN1* deletion strain (Table S1, JC25), two copies of the *DUN1* gene in wild-type strain (SN148 background) were substituted with *HIS1* and *ARG4* markers following previous protocols [12]. Similarly, the *RAD9* deletion strain was produced by replacing the two *RAD9* gene copies with *HIS1* markers in the SN148 background. Furthermore, based on the *HOF1* deletion strain, the *DUN1* or *RAD9* genes were deleted using a transient CRISPR/Cas9 system to generate the *HOF1 DUN1* or *HOF1 RAD9* double-gene deletion strains (Table S1, JC27 and JC28). The successful knockout strains were confirmed by PCR analysis.

To investigate the influence of Fkh2 on *HOF1* transcription, the *FKH2* gene in the wild-type SN148 strain was knocked out using a transient CRISPR/Cas9 system as before (Table S1, JC29). To overexpress *FKH2*, the ORF of the *FKH2* gene with its terminator was amplified by PCR and inserted into the Xhol I and Kpn I sites of the CIP10–ADH1 plasmid [20], thus generating CIP10–ADH1–FKH2. Subsequently, the linearized CIP10–ADH1–FKH2 plasmid was transformed into an SN148 strain after digestion with the Stu I enzyme (Table S1, JC30). The successful overexpression of the *FKH2* gene was confirmed through qRT-PCR analysis. Similarly, the ORF of *DUN1* genes was amplified and integrated into the CIP10–ADH1 plasmid to construct a *DUN1* overexpression strain (Table S1, JC37).

To suppress the expression of *MCM1*, a DNA fragment containing the *MET3* promoter was amplified from the pFA–URA3–MET3 plasmid [21] and introduced into the wild-type SN148 strain to replace its original promoter using a transient CRISPR/Cas9 system (Table S1, JC31). The successful substitution was confirmed by PCR analysis. The suppression and overexpression levels of the *MCM1* gene were validated by qRT-PCR. The diagram for making the mutant strains is presented in Figure S1.

### 2.3. Microscopy

For visualizing the distribution of nuclei, *C. albicans* cells were stained with DAPI [14]. Generally, overnight cultures of *C. albicans* cells were diluted 1/10 in fresh YPD media and incubated at 30 °C with shaking for 3 h before being treated with 0.02% MMS for 2 h. Subsequently, the MMS-treated cells were collected and fixed in 70% ethanol for 5 min, washed twice with 1×PBS, and then incubated in a solution containing DAPI at a concentration of 1.0 μg/mL for 10 min. Following two additional washes with PBS, the cells were mounted for analysis using a Leica DM5000B microscope (Leica Microsystems, Wetzlar, Germany) equipped with a 40 × objective. The data regarding nuclei separation represent an average from two independent experiments.

To assess filamentation triggered by genotoxic stress in *C. albicans* cells, overnight cultures were diluted in fresh YPD media and incubated at 30 °C with shaking for 3 h. Subsequently, the cell culture was treated with either 0.02% MMS or 40 mM hydroxyurea (HU) and incubated for the specified duration. Imaging was performed using a Leica DM5000B microscope (Leica Microsystems) equipped with a 40 × objective lens.

### 2.4. RNA Preparation and RNA Sequencing (RNA-Seq) Assay

The *C. albicans* wild-type SN148 strain and the *RAD53* deletion strain were inoculated into 3 mL liquid YPD and incubated overnight at 30 °C on a shaker rotating at 200 rpm. The overnight cultures were diluted to an optical density at 600 nm (OD600) of 0.1 in 10 mL YPD media and grown with shaking at 30 °C until reaching an OD600 of 0.6–0.8. Subsequently, the cells were treated with 0.015% MMS for 90 min before being harvested for RNA extraction. Total RNA was extracted using a trizol reagent kit from Invitrogen (Waltham, MA, USA) according to the manufacturer’s protocol. RNA quality was assessed using an Agilent 2100 Bioanalyzer (Agilent Technologies, Santa Clara, CA, USA) and checked using RNase-free agarose gel electrophoresis. Following total RNA extraction, mRNA was enriched using Oligo (dT) beads (Illumina, San Diego, CA, USA) and fragmented into short pieces with a fragmentation buffer. The fragments were then reverse transcribed into cDNA using the NEB Next Ultra RNA Library Prep Kit (NEB #7530, Ipswich, MA, USA). The resulting double-stranded cDNA fragments were purified and subjected to end repair, followed by the addition of an A base and ligation to Illumina sequencing adapters. Subsequently, the ligated fragments were subjected to size selection by agarose gel electrophoresis and polymerase chain reaction (PCR)-amplified. RNA library sequencing was performed on the Illumina Novaseq6000 by Gene Denovo Biotechnology Co., Ltd. (Guangzhou, China). The reads from each sample were aligned and assembled using StringTie v1.3.1 in a reference-based manner. The mapped reads of each sample were assembled by using StringTie v1.3.1 in a reference-based approach. The *C. albicans* SC5314 genome data downloaded from NCBI (https://www.ncbi.nlm.nih.gov/genome/21?genome_assembly_id=294796 (accessed on 28 December 2021) were used as a reference genome. Raw data have been deposited in the NCBI SRA database (PRJNA811694 and PRJNA985884).

Transcript analysis was conducted using the DESeq2 software 1.24.0, thus focusing on transcripts with adjusted *p* values ≤ 0.05 between the two groups. Pathway enrichment (KEGG) analysis was conducted to identify significantly enriched metabolic pathways or signal transduction pathways in differentially expressed genes (DEGs) compared to the whole genome background. The calculated *p* value went through FDR correction, with a threshold set at FDR ≤ 0.05. Pathways meeting this criterion were defined as significantly enriched pathways in DEGs. Gene set enrichment analysis (GSEA) was performed using the GseaPreranked tool (v.2.2.0) and the weighted enrichment statistics on 6387 (for *C. albicans*) gene sets, with each containing 5 to 500 genes [22].

### 2.5. Real-Time PCR (qRT-PCR)

To validate the transcription of specific genes, qRT-PCRs were conducted. The samples of the wild-type SN148 strain and the *RAD53* deletion strain, treated or untreated with 0.015% MMS for 90 min, were collected as previously described. Total RNA was extracted using an RNA-easy isolation reagent (R701, Vazyme, Nanjing, China), and cDNA was synthesized using the cDNA synthesis kit (R212, Vazyme, Nanjing, China) incorporating DNase to eliminate residual DNA in the template. qRT-PCRs were carried out utilizing ChamQ SYBR qPCR Master Mix (Vazyme, Nanjing, China) according to the protocol from the manufacturer. Specific primers for the target genes, along with the GAPDH primers serving as controls, are listed in Table S2. The data for each gene represent an average derived from at least three independent experiments.

### 2.6. ChIP Assay

The overnight culture of the strains harboring the Fkh2-HA, Mcm1-HA, or Rad53-HA fusion was transferred to 40 mL liquid YPD and grown until reaching OD600 around 0.6. The cells were then exposed to 0.02% MMS for 90 min, with an untreated wild-type cell culture of comparable growth time used as a control. Following this treatment, the samples were fixed with 1% formaldehyde for 30 min before being harvested for ChIP analysis [23]. Cell disruption was achieved by agitating the cells with glass beads in a disruptor (SI-DD48, Scientific Industries, Bohemia, NY, USA) for five rounds of 6 min each, thus generating whole-cell extract (WCE). The WCE was subsequently sonicated 9 times for 10 s each using an ultrasonic cell disruptor (0.2 kW, JY92-2D, Ningbo, China), thus resulting in DNA fragments with an average length of 200 bp. Immunoprecipitation was performed using anti-HA beads from ROCHE. The bound DNA fragments were eluted and purified utilizing a MicroElute Cycle Pure Kit (Omega Bio-tek, Norcross, GA, USA). Finally, potential binding events at the promoter of *HOF1* were detected by PCR.

### 2.7. ChEC Assay

To quantify the binding of Mcm1 and Fkh2 to the *HOF1* promoter, a chromatin endogenous cleavage (ChEC) coupled with qPCR assay was used [24]. Initially, a pFA–HA–Mnase–Ura3 plasmid was synthesized following the sequence derived from pFA–MNase–CaURA3. Mcm1 or Fkh2 were then tagged with an HA–Mnase fusion using the PCR products amplified from pFA–HA–Mnase–Ura3 plasmid. The successful integration of the HA–Mnase fusion constructs was confirmed by PCR and western blot analyses. In the ChEC experiment, overnight cultures were diluted to an initial OD600 of 0.1 in 20 mL liquid YPD medium and cultured at 30 °C until reaching an OD600 of 0.6–0.8. Subsequently, cells were exposed to 0.02% MMS for 90 min, while untreated cells under the same culture conditions served as controls. Harvested cells were washed and treated with 0.1% digitonin, as previously described. Mnase digestion was performed with 5 mM CaCl_2_ for 5 min, followed by quenching with 60 μL of 250 mM EGTA solution. Total DNA was extracted using a kit from Yuanye Bio-Technology (Shanghai, China), and DNA fragments ranging from 100 bp to 400 bp were selectively isolated using a Gel DNA Extraction Mini Kit (Vazyme, China). The enriched DNA fragments were then employed for qPCR analysis, with GAPDH primers used as internal controls for quantification. The data presented here are an average derived from a minimum of three independent experiments.

### 2.8. Yeast One-Hybrid Assay

The pGADT7 and pHIS2 plasmids were employed for yeast one-hybrid assay [25]. The HOF1 promoter, in different lengths, was amplified by PCR and inserted into the EcoR I site of the pHIS2 plasmid as bait using the Universal One Step Cloning Kit (Yeasen, Shanghai, China). The pGADT7 plasmids containing N-terminus, C-terminus, or full-length Rad53 were obtained from our previous study [26]. Yeast Y187 strain was transformed with various combinations of pGADT7 and pHIS2 plasmids using the lithium acetate method and selected on solid SC medium lacking tryptophan and leucine (SC−Trp−Leu). The transformants were picked, resuspended in distilled water, and standardized to a consistent cell density. Subsequently, 3 μL cell suspension was dropped onto SC medium lacking tryptophan, leucine, and histidine (SC−Trp−Leu−His) containing 50 or 75 mM 3-amino-1,2,4-triazole (3-AT). The plates containing cotransformed yeast cells were cultured at 30 °C for 2–3 days. Yeast cells containing pGADT7 and pHIS2–HOF1p-1 plasmids served as negative controls.

## 3. Results

### 3.1. Checkpoint Kinases Play Distinct Roles in Response to Genotoxic Stresses

**Figure 1:**
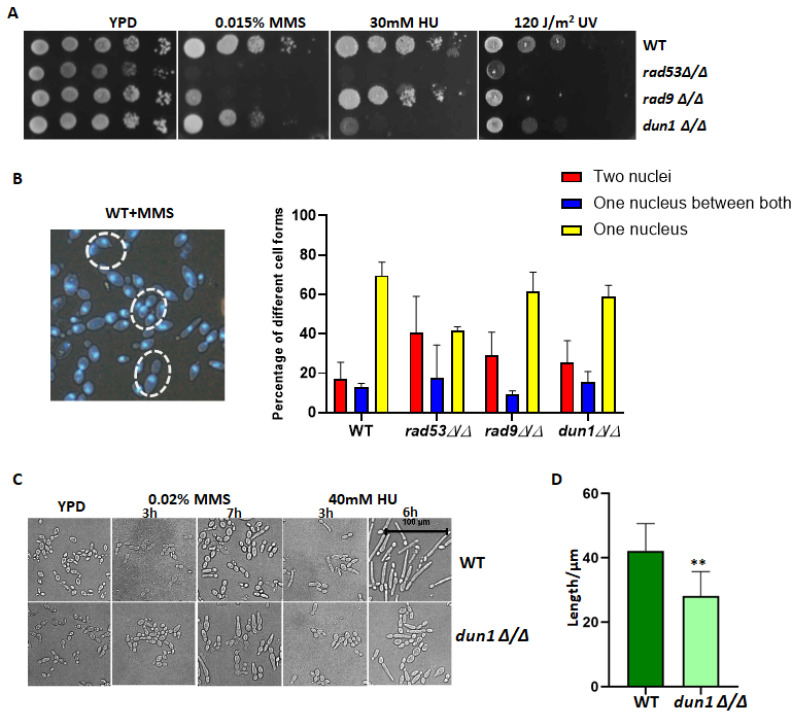
Functional characterization of checkpoint kinases responding to genotoxic stresses in *C. albicans*. (**A**) Phenotypic assay of the *RAD53* deletion, the *RAD9* deletion, and the *DUN1* deletion strains under genotoxic stresses. Two independent mutants for each strain were used for phenotypic assays, thus showing consistent results. (**B**) Nuclei separation of the wild type (SN148), the *RAD53* deletion, the *RAD9* deletion, and the *DUN1* deletion strains. The log phase cells were treated with 0.02% MMS for 120 min and then stained with DAPI. Cells with buds containing different types of nuclei were divided into three groups as indicated. The result was averaged from two independent experiments. (**C**) Filamentous growth of the *DUN1* strain induced by genotoxic stress. The wild-type and the *DUN1* deletion cells were treated with 0.02% MMS or 40 mM HU for the indicated time. The cell morphology was checked and imaged (400×). (**D**) The long bud of the *DUN1* deletion cells induced by 40 mM HU for 6 h was measured using Image J software 1.42. Over 30 cells were checked for each strain. The difference was compared using a paired *t* test with GraphPad Prism 8.0.1 software. ** represents *p* < 0.01.

Checkpoint kinases are essential for cells to coordinate the cell cycle progression and gene transcription in response to DNA damage. However, the specific functions of these checkpoint kinases in *C. albicans* remain unclear. In this study, we generated deletion strains lacking Dun1 or Rad9 and evaluated their importance in DNA damage stress response in comparison to Rad53. MMS is a commonly employed DNA-damaging agent that induces alkylating damage to DNA, thus resulting in single-strand breaks (SSBs) that can progress to double-strand breaks (DSBs) [12]. Meanwhile, hydroxyurea (HU) serves as a widely utilized genotoxic agent by inhibiting ribonucleotide reductase (RNR), thus leading to cell cycle arrest in the S-phase through the induction of replication stress [27]. Under normal conditions without MMS stress, the deletion of *RAD53* caused a significant growth defect, while the deletion of *RAD9* or *DUN1* allowed for similar growth rates as the wild-type strain on solid YPD media. However, under stress conditions, the deletion of *RAD53* led to strong sensitivity to MMS, HU, or UV (Figure 1A). In contrast, the deletion of *RAD9* exhibited pronounced sensitivity to MMS- or UV-induced DNA damage stress while displaying only moderate sensitivity to HU-induced DNA replication stress (Figure 1A). In contrast, the deletion of *DUN1* showed strong sensitivity to HU but mild sensitivity to MMS or UV (Figure 1A).

The primary role of checkpoint kinases in response to DNA damage stress is in ensuring the proper arrest of the cell cycle. Previous studies have highlighted the essential functions of Rad53 and Rad9 in arresting the cell cycle under various genotoxic stresses in *C. albicans* [28]. To investigate whether Dun1 plays a similar role in regulating the cell cycle progression, we treated the mutant cells lacking *DUN1*, *RAD9*, or *RAD53* with MMS and examined nuclear separation as an indicator of expected arrest. Upon exposure to MMS-induced damaged DNA that triggers checkpoint activation, wild-type cells displayed restrained nuclei separation; a total of 69.6% of treated WT cells showed arrested nuclei separation, and only 17.1% escaped this blockage (Figure 1B). In contrast, 40.6% of the *RAD53* deletion cells escaped the blockage, and only 41.9% exhibited checkpoint-mediated arrest; likewise, for *RAD9*-deleted cells, 29.3% escaped the blockage, while 61.5% exhibited checkpoint-mediated arrest, thus demonstrating their consistent roles in cell cycle arrest as previously reported [28]. In the case of *DUN1*-deleted cells, 25.5% of cells bypassed the block, while 59.1% arrested the cell cycle, thus indicating a moderate role of Dun1 in cell cycle regulation.

The checkpoints play a critical role in controlling the filamentous growth induced by genotoxic stresses in *C. albicans*. The deletion of *RAD53* significantly triggered MMS- or HU-induced filamentous growth, while the deletion of *RAD9* only hindered filamentous growth in response to MMS, but not HU [28]. Since Dun1 is a potential downstream kinase of Rad53, we investigated whether it shares a similar function in regulating genotoxic stress-induced filamentous growth. After a 7 h MMS treatment, both wild-type cells and *DUN1*-deleted cells exhibited elongation without significant difference (Figure 1C). Following a 6 h HU treatment, wild-type cells showed significant elongation, while *DUN1*-deleted cells also elongated but with impaired bud development; the mutant cells showed much shorter buds compared to the wild-type cells (Figure 1C,D), thus suggesting a role of Dun1 in regulating HU-induced filamentous growth in *C. albicans*.

In summary, the checkpoint kinase Rad53 plays a central role in response to genotoxic stresses, whereas Dun1 primarily functions during replication stress, and Rad9 mainly responds to DNA damage stress.

### 3.2. Profiling the Rad53-Related Transcriptome in C. albicans

Rad53 has been recognized as a key player in the cellular stress response [28,29]. However, in growth conditions devoid of stress, the absence of *RAD53* led to a reduced growth rate. A thorough transcriptional analysis was carried out to explore the effects of Rad53 depletion under nutrient-rich conditions, thus revealing notable expression variations; a total of 6104 transcripts were identified, with 2026 showing significant changes based on a log2-fold cut-off of 1.0 (Table S3). Notably, among these differentially expressed genes, 1211 exhibited upregulation, with significant alterations observed in 21 genes displaying log2-fold cut-offs greater than six (Figure S2A and Table S3). Noteworthy among them was *Orf19.4653*, thus encoding a putative GPI-linked cell wall protein exhibiting the highest upregulation (log2-fold cut-off = 14.91). In addition, genes encoding cell wall proteins such as *CDA2*, *PGA31*, and *PGA23*, along with the chromosome stability-related gene *REC8*, showed upregulation. Conversely, we observed downregulation in 815 genes, including notable repression in 30 genes displaying log2-fold cut-offs over 10 (Figure S2B and Table S3). For instance, *Orf19.446.1*, encoding a protein with a NADH-ubiquinone oxidoreductase B18 subunit domain, and *IMG2*, encoding a mitochondrial ribosomal protein of the large subunit, demonstrated significant downregulation, thus indicating compromised respiration capacity. Notably, the downregulation of *Orf19.5042* implicated its potential role in rDNA maintenance and mitotic exit, thereby providing insights into how Rad53 influences the cell cycle progression.

To elucidate the comprehensive transcriptome associated with Rad53, we performed KEGG term analysis on genes with altered transcription. Notably, ribosome biogenesis exhibited significant enrichment, thus comprising 30 upregulated genes and five downregulated genes. Additionally, the pathways associated with steroid biosynthesis, aminoacyl-tRNA biosynthesis, sesquiterpenoid and triterpenoid biosynthesis, as well as terpenoid backbone biosynthesis, were enriched. These findings underscore the crucial role of Rad53 in maintaining the normal growth of *C. albicans* cells. Consistent with its involvement in cell cycle regulation, a set of 30 upregulated genes and 10 downregulated genes were significantly enriched in the cell cycle process, including *CAK1* (a monomeric CDK-activating kinase), and several CDC genes, such as *CDC28* and *CDC53* (Figure S2C and Table S3). Collectively, our results highlight the extensive transcriptional regulation mediated by Rad53 under nutrient-rich conditions.

### 3.3. Profiling the Rad53-Related Transcriptome under DNA Damage Stress in C. albicans

**Figure 2:**
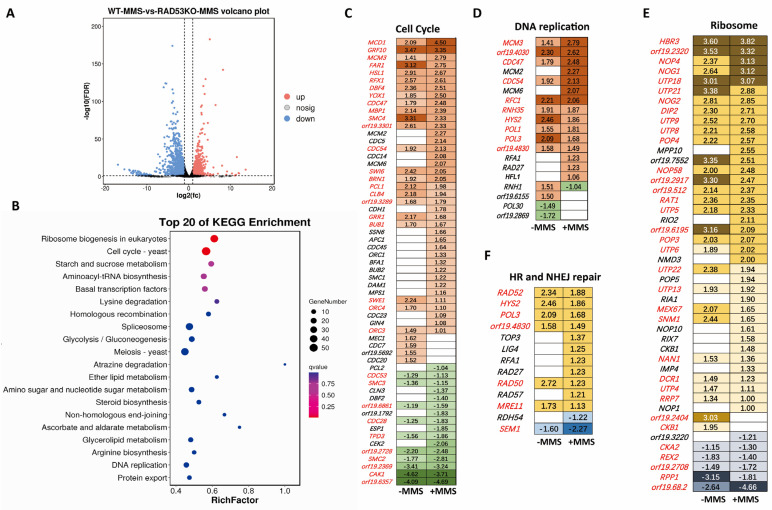
Overview of *RAD53*-related transcriptome under MMS stress in *C. albicans*. (**A**) Volcano plot showing the global transcriptional changes affected by Rad53 under the stress of MMS. (**B**) Top-20 KEGG terms of differed genes in response to MMS by deleting *RAD53*. Cell cycle-involved genes (**C**), DNA replication-involved genes (**D**), and ribosome biogenesis-involved genes (**E**) affected by Rad53 in *C. albicans*. The fold change for each gene under the normal condition (first column) or the MMS stress condition (second column) is shown after the gene name, with blanks indicating no significant change based on transcriptome data. Genes highlighted in red indicate consistent changes between normal and MMS stress conditions. (**F**) DNA damage repair genes were affected by deleting *RAD53* under MMS stress conditions. The fold change for each gene is shown after the gene name.

In the presence of MMS stress, a total of 6103 transcripts were detected, with 2404 transcripts exhibiting significant changes upon *RAD53* deletion using a log2-fold cut-off of 1.0 (Figure 2A, Table S4). Among them, 1234 genes were upregulated, while 1170 genes were downregulated. Notable upregulated genes included *BUD20*, *PGA31*, and *Orf19.4653*, which are associated with cell wall synthesis (Table S4). As well, *CSA2* encoding an extracellular heme-binding protein was also significantly upregulated. Conversely, *NUE1*, encoding a mitochondrial protein required for the expression of mitochondrial respiratory chain complex I, and *PEX17*, encoding a putative peroxin, were among the prominently downregulated genes. Despite Rad53’s critical role in DNA damage response, only a limited number of genes, like *RTT101* and *DAP1*, exhibited significant alterations upon its deletion; most differentially expressed genes were associated with antioxidation response, cell wall integrity, or other DNA damage-unrelated pathways.

The transcriptome affected by deleting *RAD53* in both normal and MMS stress conditions demonstrated high consistency, with 797 genes being commonly upregulated, 414 genes being specifically induced in nutrient-rich conditions, and 437 genes being specifically induced under MMS stress (Figure S3 and Table S5). Among these commonly upregulated genes, *Orf19.4653*, *Orf19.6487*, and *LDG3* consistently showed over 10-fold upregulation under both conditions. Similarly, there were 703 commonly suppressed genes; 112 genes were specifically suppressed in rich media, and 467 genes were specifically suppressed under the MMS stress condition (Figure S3 and Table S6). Noteworthy downregulated genes like *LEU2*, *ARC18*, and *Orf19.6308* consistently exhibited an over 10-fold decrease in expression under both conditions.

The enrichment of KEGG terms was compared between normal and MMS stress conditions after deleting *RAD53*. In the MMS stress condition, several KEGG terms, including ribosome biogenesis, cell cycle, starch, and sucrose terms, were enriched (Figure 2B). Notably, there was high consistency in the top-20 KEGG terms identified under both normal or MMS stress conditions; specifically, cell cycle, DNA replication, ribosome biosynthesis, and other metabolism terms were consistently enriched in these two conditions. This observation suggests a well-defined regulation mediated by Rad53. Collectively, our findings highlight the critical role of Rad53 in governing global gene transcription.

Cell cycle-related genes were enriched in both normal and MMS stress conditions upon the deletion of *RAD53*, which is known to function in checkpoints for cell cycle arrest following DNA damage. In normal conditions, 37 cell cycle-related genes were enriched, while 55 cell cycle-related genes were enriched in the MMS stress condition (Figure 2C). Moreover, high consistency was observed in the enriched genes across both conditions. Among the 37 genes affected by *RAD53* deletion in normal conditions, 33 were also found in the MMS stress group. Only four genes (*MEC1*, *CDC7*, *Orf19.5692*, and *CDC20*) were specifically affected by Rad53 under nonstress conditions. The top-3 commonly upregulated genes in both conditions were *MCD1*, *GRF10*, and *MCM3*; where *MCD1* encodes an Alpha-kleisin cohesin subunit involved in sister chromatid cohesion in mitosis and meiosis, thus supporting its role in cell cycle arrest. Additionally, *CDC28*, encoding a cyclin-dependent protein kinase [30,31], was downregulated upon *RAD53* deletion.

The genes associated with DNA replication were significantly enriched upon the deletion of *RAD53* under both normal and MMS stress conditions (Figure 2D). Moreover, there was a remarkable consistency in the genes affected by Rad53 between these conditions. Particularly, the upregulation of *POL1*, a putative DNA-directed DNA polymerase alpha, and *POL3*, the large subunit of DNA polymerase III, indicates their close functional association with Rad53. However, deleting *RAD53* resulted in the downregulation of *POL30* (PCNA) in both normal and MMS stress conditions (log2-fold change = −0.75, *p* < 0.05).

Recent studies have implicated ribosomes in cell cycle regulation [32], and our findings reveal that ribosome biogenesis ranks highest in KEGG terms under both conditions (Figure 2E). Specifically, we noted the upregulation of 28 genes, including *HBR3*, participating proteasomal and 40S ribosomal subunit biogenesis; *NOP4*, encoding a putative nucleolar protein; and *UTP18*, encoding a U3 snoRNA-associated protein. Additionally, five genes, such as *CKA2*, *REX2*, *Orf19.2708*, *RPP1*, and *Orf19.68.2*, displayed consistent downregulation under normal and MMS stress conditions according to RNA-seq data.

Unlike the top-20 KEGG pathways affected by *RAD53* in the absence of stress, DNA damage repair pathways were specifically enriched under MMS stress conditions. In this context, we observed a significant enrichment of several genes involved in homologous recombination (HR, Figure 2F) and nonhomologous end joining (NHEJ, Figure 2F). Notably, *RAD52*, a well-known HR-related gene, exhibited moderate upregulation, along with increased transcription levels of other HR-related genes like *RAD50* and *RAD57*. However, *RDH54* and *SEM1* displayed downregulation. Furthermore, classical NHEJ-associated genes, including *LIG4*, *RAD27*, *RAD50*, and *MRE11*, were enriched [33].

### 3.4. Pooling RAD53-Dependent DNA Damage-Responsive Genes

**Figure 3:**
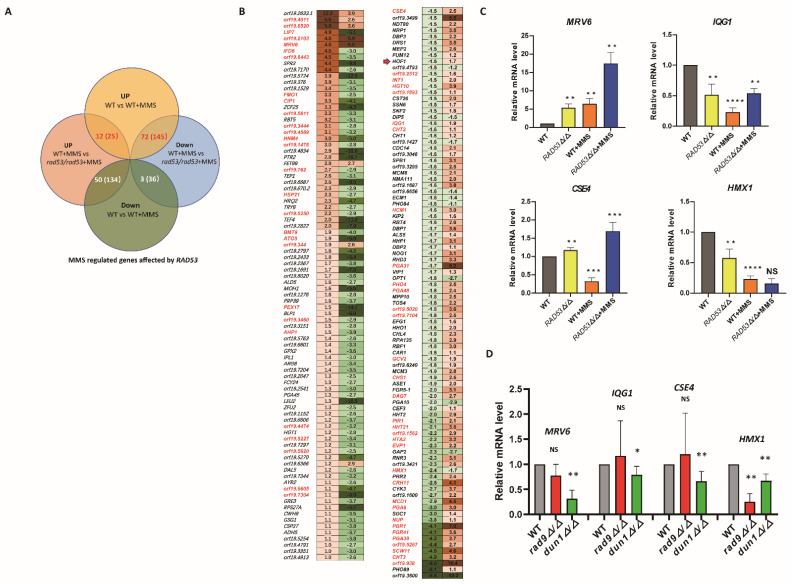
Uncovering *RAD53*-dependent DNA damage responsive genes in *C. albicans*. (**A**) Overview of MMS-responsive genes affected by deleting *RAD53*. The number without brackets represents the defined MMS-responsive genes, while the number with brackets represents the putative MMS-responsive genes. (**B**) List of MMS-induced (left panel) or -repressed genes (right panel) affected by Rad53 in *C. albicans*. The fold change for each gene affected by MMS stress in wild-type strain (left column) or by deleting *RAD53* upon exposure to MMS (right column) is listed after the gene name. Genes highlighted in red represent the defined MMS-responsive genes, and those genes in black represent the putative MMS-responsive genes. (**C**) Relative transcription of MMS-responsive genes affected by deleting *RAD53* in MMS stress conditions. The transcription of indicated genes in wild-type strain under MMS stress was compared to the level in wild-type strain without MMS treatment, and the transcription of indicated genes in the *RAD53* deletion strain was compared to the level in wild-type strain with or without MMS treatment. (**D**) Relative transcription levels of Rad53-regulated genes affected by deleting *RAD9* or *DUN1* in the MMS stress condition. The transcriptional levels of indicated genes in the *RAD9* or *DUN1* deletion strains were compared to those in wild-type strain under MMS treatment. The qRT-PCR assays for each strain were repeated at least 3 times. The difference between each group was compared using paired *t* test with GraphPad Prism 8.0.1 software. * represents *p* < 0.05, ** represents *p* < 0.01, *** represents *p* < 0.001, **** represents *p* < 0.0001. NS represents no significant difference.

The transcripts that differ by deleting *RAD53* may encompass genes that did not exhibit significant changes in response to MMS-induced DNA damage. In a previous study, we identified 306 defined (genes with consistent transcription in two independent RNA-seq data sets) and 705 putative (the remaining genes from two independent RNA-seq data sets selected by a *p* value less than 0.05) MMS-responsive genes [12]. To identify Rad53-dependent DNA damage-responsive genes, we examined the transcription of these MMS-responsive genes in the present study. Most of the defined MMS-responsive gene clusters were not found among the gene list affected by deleting *RAD53*, thus suggesting their transcription is likely independent of *RAD53*. However, among these genes, 12 defined and 25 putative upregulated genes, including *MRV6* and *GST1*, exhibited higher transcription levels in the *RAD53* deletion strain compared to the level in wild-type strain when treated with MMS (Figure 3A,B), thus indicating a suppressive role for Rad53 on these specific MMS-responsive genes. In addition, under MMS stress, 72 defined and 145 putative upregulated genes displayed reduced transcription levels in the *RAD53* deletion strain compared to the wild-type strain (Figure 3A,B). These findings indicate that Rad53 is responsible for, to some extent, the transcription of these 217 upregulated genes induced by MMS.

In the gene cluster suppressed by MMS, 50 defined and 134 putative MMS-responsive genes exhibited higher transcription levels in the *RAD53* deletion strain compared to the wild type under MMS treatment (Figure 3A,B), thus indicating a repressive role of Rad53 on these MMS-responsive genes. Moreover, among the downregulated genes, three defined and 36 putative ones showed even lower transcription levels in the *RAD53* deletion under the MMS stress condition compared to the wild-type strain (Figure 3A,B). Collectively, these findings suggest that Rad53 may influence a total of 137 defined and 340 putative MMS-responsive genes in response to DNA damage induced by MMS.

We further validated the regulatory role of Rad53 in controlling these potential targets under MMS stress. In the wild-type strain, *MRV6* was upregulated as expected after MMS treatment; however, this upregulation was amplified upon *RAD53* deletion (Figure 3C). In addition, *IQG1* and *CSE4* were downregulated in response to MMS; nevertheless, the deletion of *RAD53* increased their transcriptional level (Figure 3C). Furthermore, *HMX1* was downregulated upon exposure to MMS stress, but its transcription was exacerbated by the deletion of *RAD53*. Under normal conditions, *RAD53* deletion consistently influenced the transcription of *MRV6*, *CSE4*, and *HMX1*. In general, our findings suggest that Rad53 plays a regulatory role on potential targets in response to MMS-induced DNA damage stress.

In light of the functional correlation between Rad53 and Rad9 or Dun1, we explored the transcription of potential targets of Rad53 by deleting *RAD9* or *DUN1*. The deletion of *DUN1*, but not *RAD9,* led to decreased transcription levels of *MRV6*, *IQG1*, and *CSE4* under MMS-induced stress conditions (Figure 3D). In contrast, both *RAD9* and *DUN1* deletions caused decreased transcription levels of *HMX1* upon MMS treatment (Figure 3D). Taken together, these findings highlight the diverse regulatory roles played by Rad53, Rad9, and Dun1 on downstream genes.

### 3.5. Transcription of HOF1 Depends on Checkpoint Kinases Rad9 and Rad53

**Figure 4:**
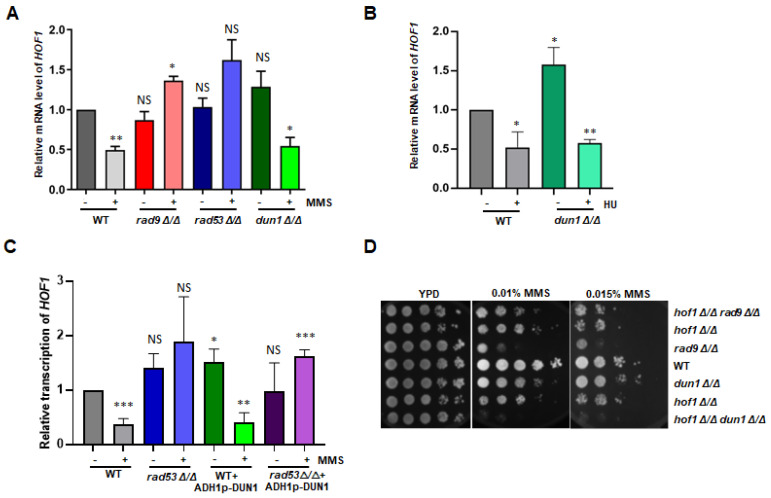
Checkpoint kinases Rad53 and Rad9 regulate the transcription of *HOF1* in *C. albicans*. (**A**) The transcription of *HOF1* after deleting *RAD9*, *RAD53*, and *DUN1* was checked by qRT-PCR. The wild-type strain (SN148), the *RAD9* deletion strain, the *RAD53* deletion strain, and the *DUN1* deletion strain were treated with 0.015% MMS for 90 min before being harvested for RNA extraction. The transcription of *HOF1* in each strain was compared to the level in wild type with no MMS stress. (**B**) The transcription of *HOF1* after deleting *DUN1* was checked by qRT-PCR. The wild-type strain (SN148) and the *DUN1* deletion strain were treated with 40 mM HU for 90 min before being harvested for RNA extraction. The transcription of *HOF1* in each strain was compared to the level in wild type without MMS stress. (**C**) The transcription of *HOF1* after overexpressing *DUN1*. The wild-type strain or the *RAD53* deletion strain with or without the *DUN1* overexpression cassette was treated with MMS, as mentioned in panel A. The qRT-PCR assay for each strain was repeated at least 3 times. The transcription of *HOF1* in each group was compared to the level in wild type without MMS stress. The difference between each group was compared using paired *t* test with GraphPad Prism 8.0.1 software. * represents *p* < 0.05, ** represents *p* < 0.01, and *** represents *p* < 0.001. NS represents no significant difference. (**D**) Phenotypic assay of the *HOF1 RAD9* double deletion and the *HOF1 DUN1* double deletion strains to MMS stress.

Within the potential targets of Rad53 (Figure 3B), we observed the downregulation of *HOF1*, a previously reported checkpoint-related gene, following MMS treatment; however, its expression increased post *RAD53* deletion. This discovery is consistent with our previous result demonstrating a decline in the protein level of Hof1 with MMS treatment, which then returned to normal after *RAD53* removal [14]. To validate this observation, qRT-PCRs were conducted. In the wild-type strain, the transcriptional suppression of *HOF1* was evident under MMS stress, as its expression declined to approximately half compared to basal levels. Conversely, in the *RAD53* deletion strain, *HOF1* transcription remained unchanged, thus resembling wild-type levels without MMS stress (Figure 4A) and indicating that the Rad53-mediated repression of *HOF1* originates from transcriptional mechanisms.

Rad53 acts as a critical effector in the signaling pathways of checkpoints, thus relying on the participation of additional kinases like adaptor Rad9 and potential downstream kinase Dun1 [29]. To determine the direct association of *HOF1* regulation with Rad53 or its broader connection to the checkpoint pathway, we examined the transcriptional levels of *HOF1* in the *RAD9* or *DUN1* mutants. Consistent with findings from *RAD53* mutant analysis, the transcriptional level of *HOF1* in the *RAD9* mutant resembled that observed in the wild-type strain; however, under the MMS stress condition, instead of decreasing, *HOF1* transcription increased beyond wild-type levels (Figure 4A). Conversely, in the *DUN1* mutant strain, *HOF1* transcription exhibited a pattern similar to that seen in the wild-type strain; following exposure to MMS, a decrease in *HOF1* transcription was observed (Figure 4A). In general, our results indicate that the proper regulation on *HOF1* transcription upon MMS-induced DNA damage relies on checkpoint kinases Rad53 and Rad9 rather than Dun1.

Given that the deletion of *DUN1* only resulted in moderate sensitivity to MMS in *C. albicans*, it may elucidate the Dun1-independent regulation on *HOF1* under the MMS stress condition. In contrast, the deletion of *DUN1* conferred strong sensitivity to HU in *C. albicans*, as evidenced by our experimental findings. Hence, we investigated whether Dun1 governs the transcription of *HOF1* under the HU stress condition. Consistent with the observations during MMS stress, HU treatment significantly suppressed the transcription of *HOF1* to 52% of the level in the wild-type strain; likewise, in the *DUN1* deletion strain, HU exposure led to a significant decrease in *HOF1* transcription compared to the stress-free state in wild-type or *DUN1*-deficient cells (Figure 4B). Consequently, our results indicate that the transcriptional regulation of *HOF1* is independent of Dun1 in *C. albicans*.

In *S. cerevisiae*, checkpoint kinases govern global transcriptional shifts, with *HOF1* identified as a target downstream of Dun1, in conjunction with the Ndd1/Fkh2/Mcm1 transcription factor complex [10]. Since the transcription of *HOF1* was not Dun1-related in *C. albicans*, we examined the transcription pattern of the orthologs for other targets of Dun1 in *S. cerevisiae*. *ADE4* is downregulated upon MMS treatment in *S. cerevisiae*, and its downregulation can be blocked by deleting *DUN1* [10]. However, in *C. albicans*, the deletion of *DUN1* did not induce notable alterations in the *ADE4* transcription under both treated and untreated conditions with MMS (Figure S4). Furthermore, *RNR3* shows increased transcription following MMS treatment in *S. cerevisiae*, and its upregulation can be repressed by deleting *DUN1* [10]. In contrast to this finding, we observed a slight decrease in *RNR3* transcription in *C. albicans* post MMS treatment, with this reduction prevented by *DUN1* deletion (Figure S4). In general, our results indicate nonconserved regulation between *C. albicans* and *S. cerevisiae* for both *HOF1* and other cell cycle-related targets of Dun1.

Dun1 is recognized as a downstream kinase of Rad53 [9]. To investigate whether elevating *DUN1* levels can restore the compromised downregulation of *HOF1* in the absence of *RAD53*, we introduced an overexpression cassette for *DUN1* into the *RAD53* deletion strain. Interestingly, despite *DUN1* overexpression in the wild-type strain, *HOF1* transcription decreased following MMS treatment (Figure 4C). Furthermore, when the *RAD53* deletion strain harbored the *DUN1* overexpression cassette, the *HOF1* transcription remained unaffected by MMS treatment (Figure 4C). Thus, our findings indicate that overexpressing *DUN1* cannot compensate for the Rad53-dependent decrease in *HOF1* transcription.

Previously, we investigated the genetic interaction between *RAD53* and *HOF1*, and we observed that *RAD53* is epistatic to *HOF1* in response to MMS-induced DNA damage. To further elucidate the relationship between Hof1 and checkpoint kinases, we constructed the double deletion strains of *HOF1* with either *RAD9* or *DUN1* and assessed their sensitivity to MMS. Spot assays revealed that single-gene deletions of *RAD9* or *HOF1* both showed similar MMS sensitivity levels, whereas the double deletion strain of *RAD9* with *HOF1* exhibited comparable MMS sensitivity to their single mutants, with no significant increase in susceptibility to MMS (Figure 4D). In contrast, the *DUN1* mutant showed mild sensitivity to the low concentration of MMS; however, its double mutant with *HOF1* demonstrated pronounced hypersensitivity towards MMS (Figure 4D), thus suggesting an independent role for *HOF1* in DNA damage response that is unrelated to Dun1. Taken together, our findings indicate that both the transcriptional regulation and functional involvement of *HOF1* in MMS-induced DNA damage response rely on checkpoint kinases Rad53 and Rad9, but probably not on Dun1.

### 3.6. Transcription Factors Mcm1 and Fkh2 Regulate the Transcription of HOF1

**Figure 5:**
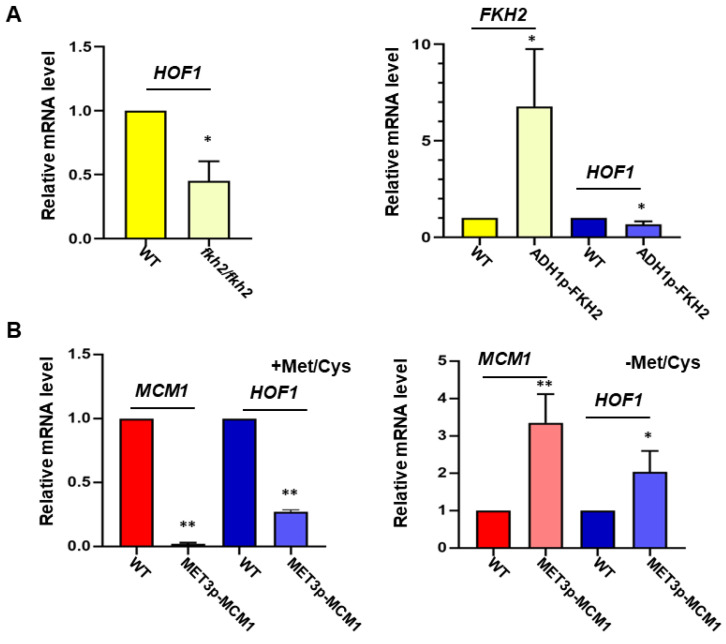
Transcription of *HOF1* affected by Mcm1 and Fkh2. (**A**) The transcription of *HOF1* was affected by deleting (left panel) or overexpressing *FKH2* (right panel). The log phase cells of indicated strains were used for qRT-PCR assays. (**B**) The transcription of *HOF1* was affected by repressing (left panel) or overexpressing *MCM1* (right panel). The promoter of *MCM1* was replaced by a *MET3* promoter in SN148 background. The overnight culture of the indicated strains was inoculated into SC media plus Met/Cys (5 mM for each) or SC-Met/Cys for 4 h before being harvested for RNA extraction. The qRT-PCR assay for each strain was repeated at least 3 times. The transcription of indicated genes was compared to the level in wild type using a paired *t* test with GraphPad Prism 8.0.1 software. * represents *p* < 0.05, and ** represents *p* < 0.01.

In *S. cerevisiae*, the regulation of *HOF1* involves an Ndd1/Fkh2/Mcm1 transcription factor complex [10]. However, *C. albicans* lacks an ortholog of Ndd1, with only Fkh2 and Mcm1 having been identified. To investigate the function of Fkh2 and Mcm1 in regulating *HOF1* in *C. albicans*, we deleted *FKH2* and also suppressed *MCM1* to assess the transcription of *HOF1*, respectively. The loss of Fkh2 notably reduced *HOF1* transcription levels to around 30% compared to wild-type strains (Figure 5A, left panel). Likewise, the suppression of *MCM1* led to a significant decrease in *HOF1* transcription (Figure 5B, left panel). Therefore, both Fkh2 and Mcm1 are essential for the proper transcriptional regulation of *HOF1*.

Furthermore, we examined the impact of *FKH2* and *MCM1* overexpression on *HOF1* transcription levels to further understand their regulatory roles. Notably, the ADH1 promoter significantly enhanced the transcription of *FKH2* (Figure 5A, right panel); nevertheless, overexpressing *FKH2* did not elevate *HOF1* transcription but instead caused a slight decrease. In contrast, employing the *MET3* promoter increased the *MCM1* transcript levels by a nearly 3-fold amount compared to the wild-type strain; moreover, elevated *MCM1* further augmented the transcription of *HOF1* (Figure 5B, right panel). These findings suggest that Mcm1 may serve as a crucial regulator governing the transcription of *HOF1*.

### 3.7. Mcm1 and Fkh2 Target the Promoter of HOF1

**Figure 6:**
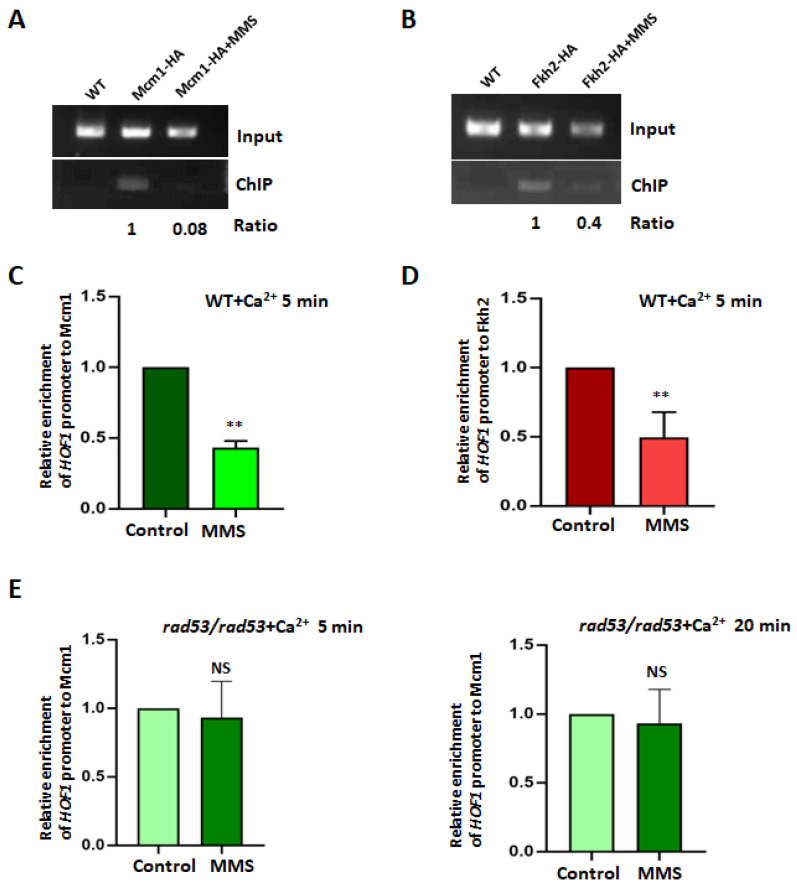
Transcription factors Mcm1 and Fkh2 bind to the promoter of *HOF1*. (**A**,**B**) Detection of the binding of Mcm1 or Fkh2 to the promoter of *HOF1* by ChIP analysis. The log phase wild-type cells (SN148) carrying Mcm1–HA or Fkh2–HA fusion, with or without 0.02% MMS treatment, were fixed with 1% formaldehyde. A wild-type strain without an HA tag was used as a control. Immunoprecipitated pellets were used as templates for PCR with the primer pairs HOF1–Chip-F and R. The intensity of the band was quantified using ImageJ software. Under no stress conditions, the ratio of the band in the ChIP group to the input group was normalized as 1. The result was averaged from two independent experiments. (**C**,**D**) The enrichment of *HOF1* to Mcm1 and Fkh2 was checked by ChEC assay coupled with qPCR. Wild-type cells carrying Mcm1–Mnase or Fkh2–Mnase fusions were used. The extracted DNA fragments around 100 bp to 400 bp from cells with or without MMS treatment were applied for qPCR assays, and the GAPDH level was used as a control. The difference was compared using a paired *t* test with GraphPad Prism 8.0.1 software. ** represents *p* < 0.01. (**E**) Rad53 regulates the dynamic enrichment of the *HOF1* promoter to Mcm1. The *RAD53* deletion cells carrying Mcm1–Mnase fusions were used. The extracted DNA fragments around 100 bp to 400 bp from cells with or without MMS treatment were applied to check the signal of the *HOF1* promoter with primers HOF1–pro-F/R, and the GAPDH level was used as a control. The difference was compared using a paired *t* test with GraphPad Prism 8.0.1 software. NS represents no significant difference.

Given that transcription factors Fkh2 and Mcm1 are essential for the transcription of *HOF1*, we conducted a ChIP analysis to explore their binding to the *HOF1* promoter under normal conditions. A band representing the promoter region (−532 bp to −261 bp, including a potential binding motif of ScMcm1) of *HOF1* was observed from the Mcm1–HA bound samples (Figure 6A), yet MMS treatment notably reduced this binding. Likewise, Fkh2 exhibited binding to the *HOF1* promoter under normal conditions, but it showed reduced binding upon MMS treatment (Figure 6B).

To quantify the altered binding of Mcm1 and Fkh2 to the promoter of *HOF1* post MMS treatment, we employed a ChEC assay coupled with qPCR. Generally, the fusion proteins Mcm1–Mnase or Fkh2–Mnase triggered specific cleavage at their respective DNA surroundings, thus resulting in the liberation of enriched DNA fragments (Figure S5) and demonstrating their binding capability to the *HOF1* promoter. The enrichment of the *HOF1* promoter with Mcm1 decreased to 43% compared to the untreated group following MMS treatment (Figure 6C), as indicated by our findings. Similarly, a notable reduction in the enrichment of the *HOF1* promoter with Fkh2 was observed under MMS treatment conditions (Figure 6D). Hence, both Mcm1 and Fkh2 exhibit dynamic binding patterns towards the *HOF1* promoter depending on the cellular status.

The deletion of *RAD53* in *C. albicans* has been shown to impede the downregulation of *HOF1* expression during MMS-induced stress [14]. Considering that Mcm1 plays a critical role in regulating the transcription of *HOF1*, we investigated whether this process involves altering the dynamic binding between the Mcm1–Mnase fusion and its target site on the *HOF1* promotor using a ChEC–qPCR assay in *RAD53* deletion cells (Figure 6D). The activation of Mcm1–Mnase in the *RAD53* deletion cells by Ca^2+^ for 5 min or 20 min did not lead to noticeable changes in the enrichment of the *HOF1* promoter under normal or MMS stress conditions. Therefore, *RAD53* deletion hinders the liberation of Mcm1 from the *HOF1* promoter.

### 3.8. Rad53 Targets the Promoter of HOF1

**Figure 7:**
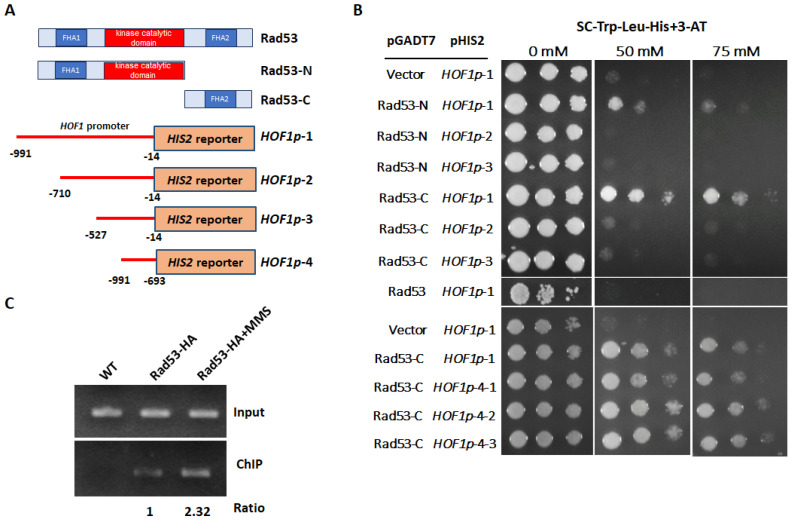
Rad53 is involved in the direct regulation of *HOF1*. (**A**) Diagram of yeast one-hybrid assay. (**B**) The FHA2 region of Rad53 binds to the promoter of *HOF1*. The different domains of Rad53 were cloned into the pGADT7 plasmid, and various regions of the *HOF1* promoter were cloned into the pHIS2 plasmid before being transformed into the yeast Y187 strain. The transformants were dissolved in distilled water and dropped onto SC–Trp–Leu–His plates containing different concentrations of 3-AT. The plates were kept at 30 °C for 2–3 days. (**C**) Checkpoint kinase Rad53 binds to the promoter of *HOF1*. The log phase wild-type cells carrying the Rad53–HA fusion, with or without 0.02% MMS treatment, were fixed with 1% formaldehyde. A wild-type strain without an HA tag was used as a control. Immunoprecipitated pellets were used as templates for PCR with the primer pairs HOF1–pro-F/R. The band intensity was quantified using ImageJ software. Under no stress conditions, the ratio of the band in the ChIP group to the input group was normalized as 1. The result was averaged from two independent experiments.

A recent study has revealed that checkpoint kinase Rad53 interacts with the promoters of approximately 20% of genes and coordinates genome-wide replication and transcription under replication stress in *S. cerevisiae* [34]. Moreover, studies on localization have shown that the majority of GFP-tagged Rad53 proteins in *S. cerevisiae* are localized within the nucleus [35]. Considering that Rad53 functions as a suppressor of *HOF1* transcription without being a transcription factor itself, we employed a yeast one-hybrid system to explore the potential interaction between Rad53 and the promoter region of *HOF1*. A series of pGADT7 plasmids containing either the FHA1 domain, the kinase catalytic domain (KD) (Rad53–N), or the FHA2 domain (Rad53–C) from our previous study were used as the prey [26]. Additionally, distinct segments of the *HOF1* promoter were integrated into the pHIS2 plasmid using it as the bait (Figure 7A). Our findings revealed no substantial interaction between the complete Rad53 protein and the *HOF1* promoter (Figure 7B). The FHA1/KD domains of Rad53 exhibited no discernible interaction with various regions of the *HOF1* promoter, except for a weak interaction detected with the complete sequence of the *HOF1* promoter (Figure 7B). However, a distinct interaction was detected between the FHA2 domain and the complete *HOF1* promoter but not its truncated versions, thus indicating a possible binding motif within the DNA segment ranging from −991 bp to −710 bp. To further validate the positive interaction between the FHA2 domain of Rad53 and the *HOF1* promoter, we integrated the DNA fragment encompassing the −991 bp to −693 bp region of the *HOF1* promoter into the pHIS2 plasmid and assessed its interaction with the FHA2 domain of Rad53; our results validated a robust interaction between the upstream region of *HOF1* promoter and the FHA2 domain of Rad53 (Figure 7C). These findings align well with previous observations showing increased signal intensity upstream rather than downstream from the transcription start site in the *HOF1* promoter of *S. cerevisiae* when probed by the Rad53 protein [34]. Therefore, our yeast one-hybrid assays provide evidence for a direct association between the checkpoint kinase Rad53 and the *HOF1* promoter.

To further investigate the interaction between the Rad53 and the *HOF1* promoter in *C. albicans*, a ChIP assay was conducted. Under normal conditions, a faint band representing *HOF1* was detected in the Rad53–HA-coated sample; upon treatment with MMS, a prominent signal of the promoter of *HOF1* was observed (Figure 7D). However, the Rad53–Mnase fusion exhibited no apparent DNA digestion activity, and the ChEC assay was not performed for Rad53. But collectively, these findings suggest that Rad53 directly contributes to the modulation of *HOF1* transcription in *C. albicans*, thus involving dynamic enrichment at its promoter region.

## 4. Discussion

In this study, we investigated the transcriptional response related to the cell cycle checkpoint kinases in *C. albicans* cells lacking Rad53 and observed a significant alteration in the gene transcription upon the deletion of *RAD53*, both in nutrient-rich and MMS stress conditions. In addition, our findings demonstrated that Rad53 and Rad9, rather than Dun1, play a role in restricting the MMS-mediated repression of *HOF1* in *C. albicans*. Moreover, we uncovered that Rad53 is crucial for regulating the dynamic binding of Mcm1 to the promoter region of *HOF1* and can directly bind to its promoter as well.

The DNA damage response is pivotal for cells to maintain genome integrity and fidelity during growth and development. Checkpoints serve as a critical hub for halting the cell cycle and repairing DNA damage, thus safeguarding daughter cells from inheriting such damage. In this study, we investigated the essentiality of checkpoint kinases Rad53, Rad9, and Dun1 in *C. albicans* under various genotoxic stress conditions. Our findings revealed diverse levels of essentiality among these checkpoint kinases, with Rad53 being identified as the most critical and Rad9 and Dun1 showing partial essentiality in addressing DNA damage or replication stress. The deletion of *RAD53* caused dramatic sensitivity to various genotoxic agents; Rad9 played a critical role in responding to DNA damage caused by MMS and UV radiation, while its significance was limited in replication stress induced by HU; Dun1, a potential downstream kinase of Rad53, appeared dispensable for MMS- or UV-induced DNA damage stress but played an indispensable role in HU-induced replication stress. Interestingly, our results indicate that the significance of these checkpoint kinases varies across different organisms, thus encompassing *C. albicans* and other eukaryotes. Although *RAD53* is essential in *S. cerevisiae*, it is nonessential in either *C. albicans* or *C. glabrata* [36,37]. Particularly, Rad53 exhibits a noncanonical role in DNA damage response within *C. glabrata*, where it remains unphosphorylated upon MMS treatment [37]. Moreover, the loss of *DUN1* leads to pronounced sensitivity to both MMS and HU stresses in *S. cerevisiae* [38]; however, its role seems less significant in responding to DNA damage but more evident in dealing with replication stress in *C. albicans*. Thus, the orthologs of checkpoint kinases in different organisms may have nonconserved roles in the DNA damage response.

Checkpoint kinases are activated to arrest the cell cycle progression and regulate transcriptional changes to facilitate DNA damage repair. Our research uncovered that the deletion of *RAD53* influenced the transcription of genes related to DNA replication and the cell cycle. *MCD1* encodes an Alpha-Kleisin cohesin complex subunit for sister chromatid cohesion in *S. cerevisiae* [39]. Additionally, Smc4 is a subunit of the nuclear condensin complex responsible for chromatin binding, chromosome condensation, and meiotic chromosome separation [40]. The increased transcription of cell cycle genes may indicate disrupted cell cycle control due to checkpoint malfunction, thus resulting in the sustained expression of these genes. Consistently, most identified DNA replication genes showed increased transcription upon *RAD53* deletion, thus indicating unrestricted DNA replication under MMS stress without checkpoint control. Furthermore, we conducted a comparison between our RNA-seq data and the documented cell cycle-modulated genes through GSEA analysis. The altered genes resulting from *RAD53* deletion demonstrated significant similarity to cell cycle-related genes influenced by Swi4/Swi6 and Ndt50, thus validating its involvement in cell cycle regulation (Table S7).

Interestingly, RNA-seq data revealed enrichment in pathways unrelated to DNA damage repair upon *RAD53* deletion, particularly those associated with ribosome biogenesis. The KEGG term for ribosome biogenesis was enriched by deleing *RAD53* under both rich-nutrient and MMS stress conditions. Ribosome biogenesis has been linked to disease and DNA repair processes [32]. Previous studies have shown that nonfunctional rRNA decay in yeast requires the involvement of DNA repair factors Rtt101 and Mms1, thus suggesting a cellular response to genotoxic stress affecting both rRNA and DNA integrity [41,42]. Ribosome biogenesis is also related to cell size; Dot6 acts as an activator in mediating the transcription of ribosome biogenesis genes, and the deletion of *DOT6* results in decreased cell size. Here, the deletion of *RAD53* resulted in disrupted cell cycle and caused a bulged cell form, thus indicating a potential link between checkpoint kinases and cell size through regulators like Dot6. Furthermore, deleting *RAD53* resulted in slow growth under both nutrient-rich conditions and MMS stress conditions, with several amino acid biosynthesis genes being downregulated consistently. These observations suggest that checkpoint kinases may downregulate biosynthesis gene expression as part of their role in slowing down cell growth. In summary, our results highlight the significant regulatory roles played by checkpoint kinases in coordinating gene transcription under different stress conditions.

Rad53 plays multiple roles in regulating the transcription of various DNA damage-responsive genes in *C. albicans*. Our previous research revealed transcriptional alterations in response to MMS, thus showing that Rad53 positively regulates the transcription of potential target genes like *RAD7* and *HTA2* [12]. However, the deletion of *RAD53* affected only a limited number of MMS-responsive genes, thus leaving a significant portion unaffected by Rad53. This discrepancy suggests the involvement of alternative mechanisms independent of Rad53 control. Notably, among the Rad53-related genes, several MMS-induced genes like *MRV6* were further upregulated by losing *RAD53*; similarly, MMS-repressed genes, including *IQG1* and *CSE4*, exhibited increased transcription by losing *RAD53*. The upregulation of these MMS-responsive genes indicates that Rad53 exerts a negative influence on their expression, thus affecting DNA damage response either directly or indirectly. Strikingly, selected MMS-responsive genes displayed significant differences in mutants lacking specific checkpoints; particularly noteworthy was the downregulation observed for *MRV6, CSE4,* and *IQG1* upon the deletion of *DUN1* compared to their upregulation upon *RAD53* deletion—thus providing further evidence of the distinct roles for Rad53 and Dun1 in the DNA damage response.

**Figure 8:**
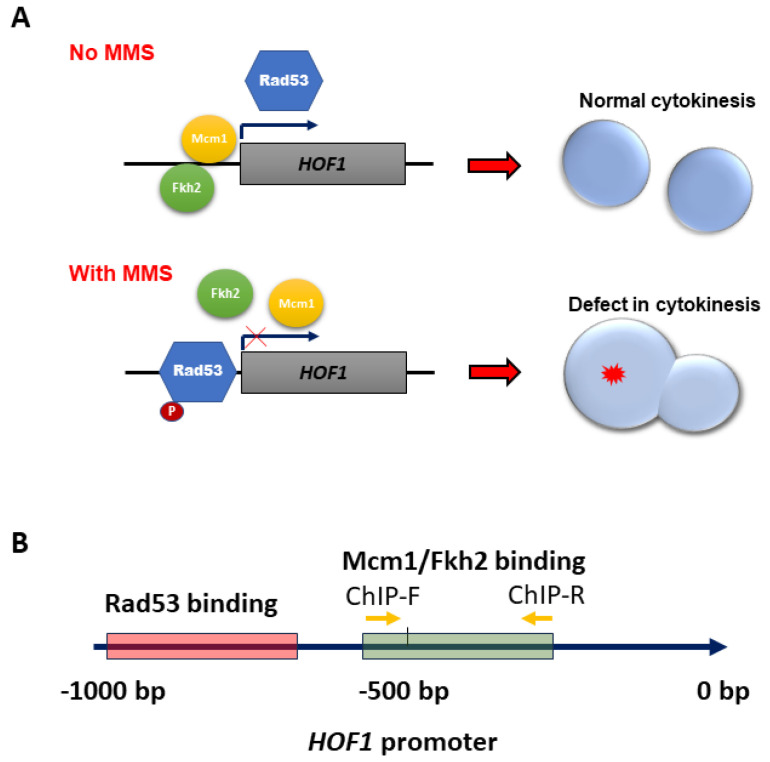
Overview of checkpoint-related regulation on *HOF1* in response to MMS in *C. albicans*. (**A**) The dynamic binding of Rad53 and Mcm1/Fkh2 to the promoter of *HOF1*. Under normal conditions, Mcm1 and Fkh2 bind to the promoter of *HOF1* and regulate the transcription of *HOF1* to ensure regular and timely cytokinesis. Upon DNA damage stress, Mcm1 or Fkh2 dissociates from the promoter of *HOF1* either by activated Rad53 or through a competition with activated Rad53, thereby subsequently diminishing the transcription of *HOF1* to impede cytokinesis and giving enough time for cells to repair damaged DNA. (**B**) The binding region for Rad53 and Mcm1/Fkh2 in the *HOF1* promoter. The red box represents the binding region of Rad53, and the green box represents the detected binding region for Mcm1 and Fkh2.

The transcriptional regulation of *HOF1* in *C. albicans* appears to differ from that in *S. cerevisiae* in terms of both the involved transcription factors and the underlying mechanism. In *S. cerevisiae*, the Fkh2/Mcm1/Ndd1 complex serves as a transcription factor complex for *HOF1*, with Mcm1 binding to its promoter throughout the cell cycle, Fkh2 acting as an inhibitor, and Ndd1 functioning as an activator [10]. However, it is possible that Ndd1 may have been lost during evolution in *C. albicans*, thus leading to the assumption of its role by other transcription factors. In particular, Mcm1 may functionally replace Ndd1, as evidenced by the effective increase in *HOF1* transcription upon *MCM1* overexpression in our study. Consistent with this, we observed the consistent upregulation of the target genes of Mcm1, *PCL1*, *MRD1*, and *SIM1*, along with the downregulation of *RAM1*, under both nonstress and MMS stress conditions, thus indicating that the loss of Rad53 influenced the transcriptional activity of Mcm1. Moreover, Mcm1/Fkh2 was found to bind to the promoter of *HOF1*; however, this binding decreased upon MMS treatment in *C. albicans*. The dynamic binding of transcription factor Mcm1 to the promoter of *HOF1* is consistent with the transcription of *HOF1*, thus suggesting that Mcm1 binding stimulates *HOF1* transcription and ensures normal cytokinesis by maintaining elevated *HOF1* levels (Figure 8A). Nevertheless, upon MMS treatment, the binding affinity between Mcm1 and the *HOF1* promoter decreased, thus leading to the downregulation of *HOF1* transcription as a response to arrest cytokinesis during DNA damage repair (Figure 8A). The deletion of *RAD53*, but not *DUN1*, abrogated this MMS-induced downregulation of *HOF1*, thus suggesting its potential dependence on a transcription factor-mediated mechanism. In the absence of Rad53 phosphorylation, Mcm1 and Fkh2 likely remain bound to the promoter region, thus leading to unaltered transcription under MMS stress conditions (Figure 8A). Therefore, species-specific mechanisms govern the transcriptional regulation of *HOF1* and potentially other cell cycle genes in *C. albicans* and *S. cerevisiae*.

Checkpoint kinase Rad53 also exhibits a potential direct regulation on *HOF1* transcription. Consistent with previous findings on Rad53’s involvement in gene promoter interactions in *S. cerevisiae* [26], we have identified a direct association between Rad53 and the promoter of *HOF1*. This association was further enhanced with MMS treatment in *C. albicans*, thus indicating potential competition between Rad53 and Mcm1/Fkh2 for binding at the *HOF1* promoter site. Our yeast one-hybrid assay revealed that Rad53 primarily interacts with the −991 bp to −693 bp region of the *HOF1* promoter, while the ChIP assay demonstrated that Mcm1 and Fkh2 bind to the −532 bp to −261 bp region of the *HOF1* promoter (Figure 8B). Moreover, consistent results were obtained in ChIP assays using primers spanning the −624 bp to −301 bp regions of the *HOF1* promoter. Although the current result did not indicate simultaneous binding of Rad53 and Mcm1/Fkh2, it can be inferred that they target adjacent regions within the *HOF1* promoter. Under MMS stress conditions, activated Rad53 binds to the *HOF1* promoter, thus impeding Mcm1 or Fkh2 from binding and consequently reducing *HOF1* transcription. Conversely, the loss of checkpoint Rad53 enables the unrestricted binding of Mcm1 or Fkh2 to the promoter of *HOF1*, thereby facilitating its necessary induction (Figure 8A,B). These findings collectively suggest that Rad53 regulates *HOF1* transcription, either independently or in conjunction with Mcm1/Fkh2.

Overall, our study presents a comprehensive overview of checkpoint-dependent transcriptional regulation during the MMS-induced DNA damage response in *C. albicans*, thus shedding light on species-specific control over cell cycle-specific genes such as *HOF1*. Furthermore, our study demonstrates how the checkpoint kinase Rad53 can specifically interact with the *HOF1* promoter, thereby potentially influencing gene transcription. These findings offer valuable insights into the checkpoint-dependent transcriptional regulation, morphogenesis, and virulence mechanisms employed by *C. albicans*.

## Data Availability

The raw data have been deposited in the NCBI SRA database (PRJNA811694 and PRJNA985884).

## Supplementary Materials

The following supporting information can be downloaded at https://www.mdpi.com/article/10.3390/jof10060387/s1, Figure S1. The diagram of constructing the mutant strains for *RAD9*, *DUN1*, *FKH2*, and *MCM1*. Figure S2. Overview of Rad53-related transcriptome in *C. albicans*. Figure S3. Comparison of transcriptome affected by deleting *RAD53* in normal condition or MMS stress condition in *C. albicans*. Figure S4. The transcription of *ADE4* and *RNR3* after deleting *DUN1*. Figure S5. The digestion of Fkh2–Mnase and Mcm1–Mnase on genomic DNA. Table S1. Strains used in this study. Table S2. Primers used in this study. Table S3. RNA-seq data of the *RAD53* deletion strain under normal condition. Table S4. RNA-seq data of the *RAD53* deletion strain under MMS stress condition. Table S5. Common upregulated genes by deleting *RAD53* under normal and MMS stress conditions. Table S6. Common downregulated genes by deleting *RAD53* under normal and MMS stress conditions. Table S7. GSEA analysis of genes affected by Rad53.
