# Supplementary material for: DNA Damage Checkpoints Govern Global Gene Transcription and Exhibit Species-Specific Regulation on HOF1 in Candida albicans
Source: J Fungi (Basel). 2024 May 29;10(6):387. doi: 10.3390/jof10060387 (PMC11204775; doi:10.3390/jof10060387)
Supplement: Supplementary file 1 [file jof-10-00387-s001.zip › supporting data.pdf]

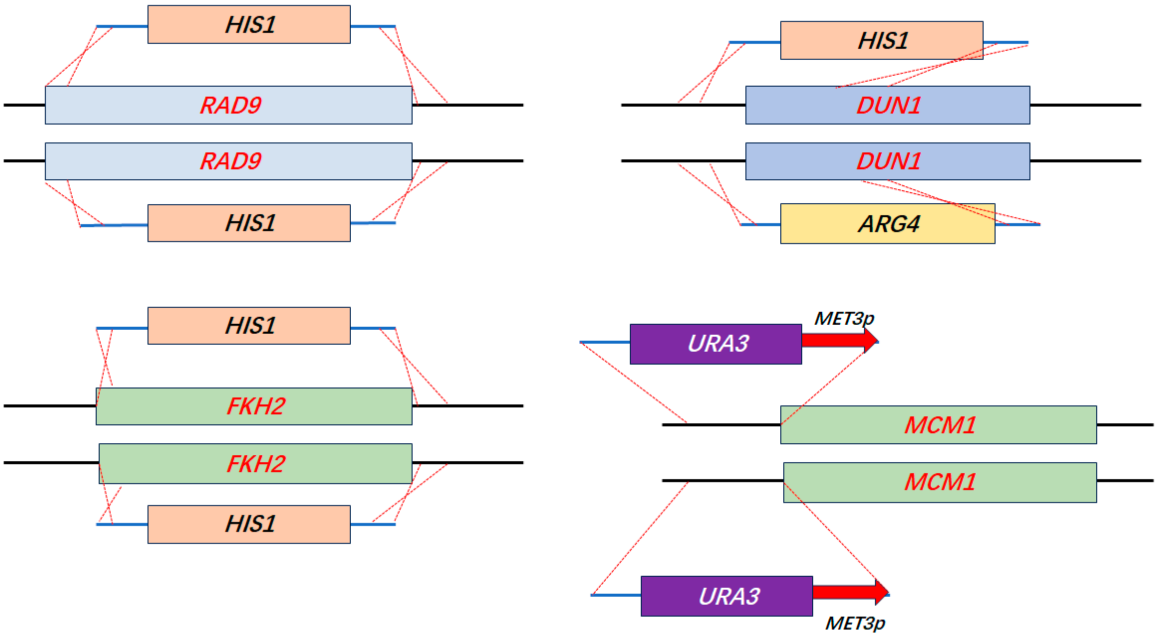

Figure S1. The diagram of constructing the mutant strains for *RAD9*, *DUN1*, *FKH2* and *MCM1*.

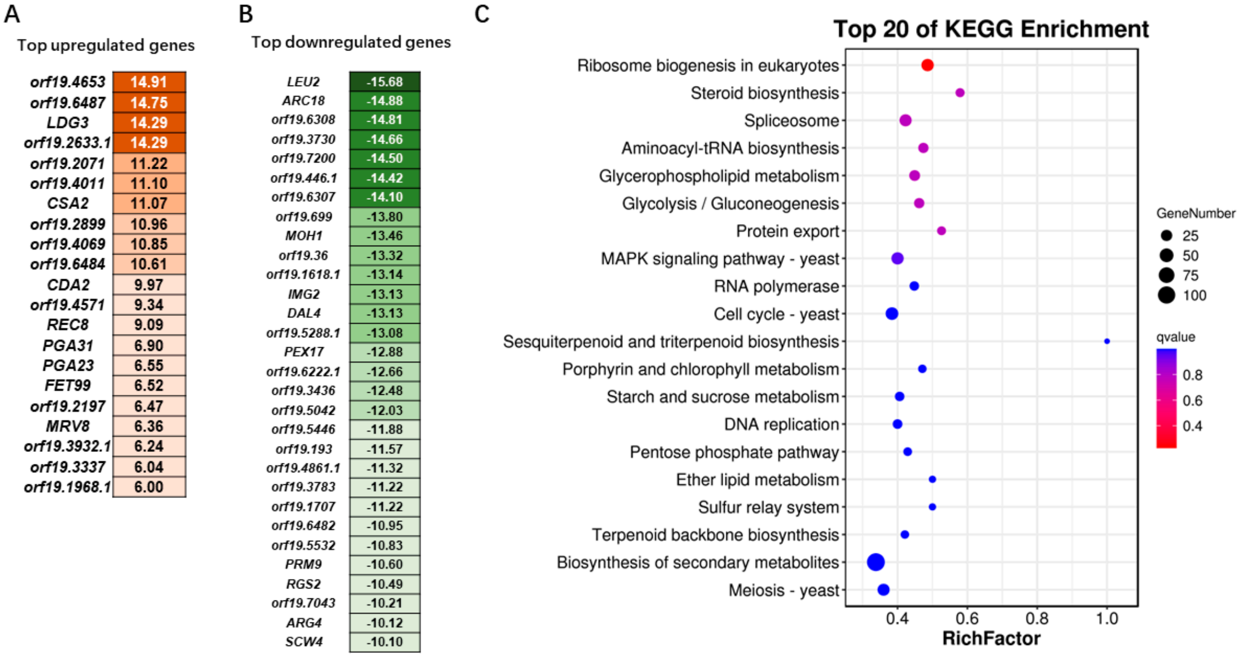

Figure S2 Overview of *RAD53*-related transcriptome in *C. albicans*. (A) Upregulated genes with log2 fold over 6. (B) Downregulated genes with log2 fold over 10. The change

fold of each gene was shown after the gene name. (C) Top 20 KEGG pathways affected by deleting *RAD53* in rich media.

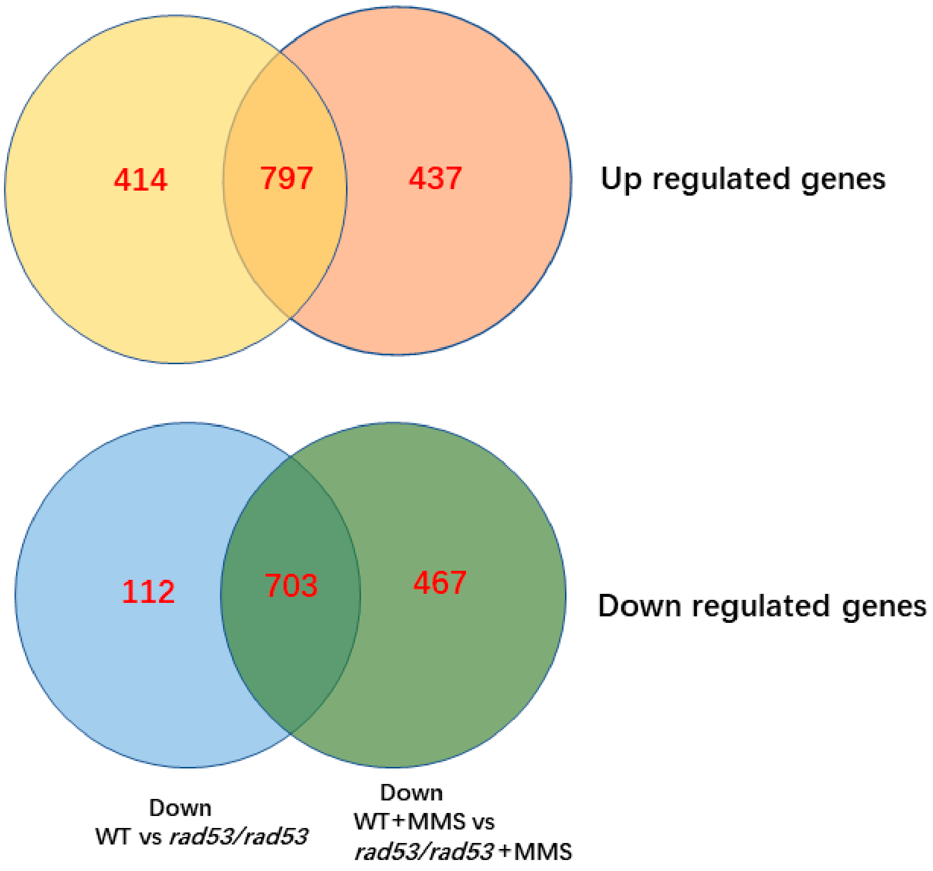

Figure S3 Comparison of transcriptome affected by deleting *RAD53* in normal condition or MMS stress condition in *C. albicans*.

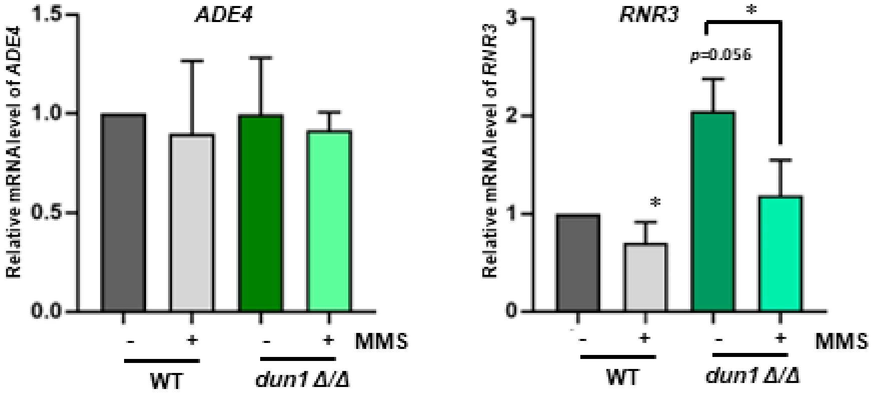

Figure S4 The transcription of *ADE4* and *RNR3* after deleting *DUN1*. The wild-type strain (SN148) and the *DUN1* deletion strain were treated with MMS. The qRT-PCR assay for each strain was repeated at least 3 times. The transcription of *RNR3* in each strain with MMS treatment was compared to the level in wild-type without MMS stress. The difference between each group was compared using paired t test with GraphPad Prism 8.0.1 software. * represents $P<0.05$ and ** represents $P<0.01$.

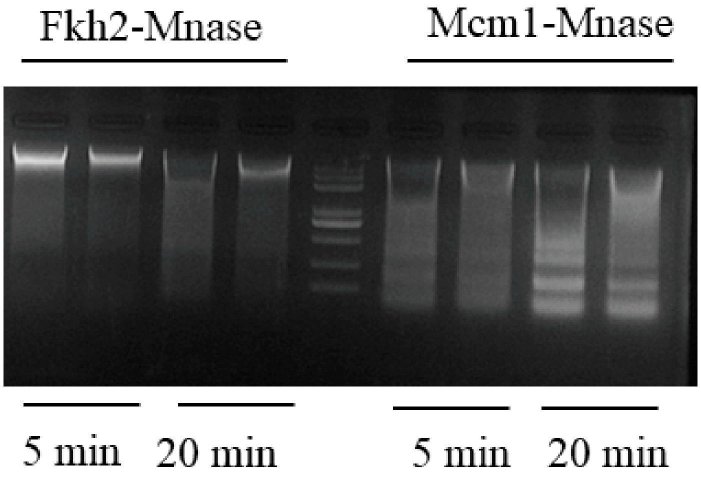

Figure S5 The digestion of Fkh2-Mnase and Mcm1-Mnase on genomic DNA. The indicated cells were treated with 5 mM $CaCl_2$ for 5min or 20min.
