# Supplementary material for: DNA Damage Checkpoints Govern Global Gene Transcription and Exhibit Species-Specific Regulation on HOF1 in Candida albicans
Source: J Fungi (Basel). 2024 May 29;10(6):387. doi: 10.3390/jof10060387 (PMC11204775; doi:10.3390/jof10060387)
Supplement: Supplementary file 1 [file jof-10-00387-s001.zip › Table S1.pdf]

Strains	Genotype	Source
SN148	*arg4/arg4 leu2/leu2 his1/his1 ura3::imm434/ura3::imm434 iro1::imm434/iro1::imm43*	Lab stock
JC23	*rad53::HIS1/rad53::HIS1*	Feng et al，2021
JC24	*rad9::HIS1/rad9::HIS1*	This study
JC25	*dun1::HIS1/dun1::ARG4*	This study
JC27	*hof1::HIS1/hof1::HIS1 rad9::ARG4/rad9::ARG4*	This study
JC28	*hof1::HIS1/hof1::HIS1 dun1::ARG4/dun1::ARG4*	This study
JC29	*fkh2::HIS1/fkh2::HIS1*	This study
JC30	*SN148::CIP10-ADH1-FKH2*	This study
JC31	*SN148::pMET3-MCM1/pMET3-MCM1*	This study
JC32	*SN148::FKH2/FKH2::HA-URA3*	This study
JC33	*SN148::MCM1/MCM1::HA-URA3*	This study
JC34	*SN148::RAD53/RAD53::HA-URA3*	This study
JC35	*SN148::FKH2/FKH2::HA-Mnase-URA3*	This study
JC36	*SN148::MCM1/MCM1::HA-Mnase-URA3*	This study
JC37	*SN148::CIP10+ADH1-DUN1*	This study
JC38	*rad53::HIS1/rad53::HIS1+CIP10-ADH1-DUN1*	This study
