# Supplementary material for: DNA Damage Checkpoints Govern Global Gene Transcription and Exhibit Species-Specific Regulation on HOF1 in Candida albicans
Source: J Fungi (Basel). 2024 May 29;10(6):387. doi: 10.3390/jof10060387 (PMC11204775; doi:10.3390/jof10060387)
Supplement: Supplementary file 1 [file jof-10-00387-s001.zip › Table S2.pdf]

Name	Oligos	Description
P7	ATCTCATTAGATTTGGAACTTGTGGGTT	CAS9 gene
P8	TTCGAGCGTCCCAAAACCTTCT	CAS9 gene
P1	AAGAAAGAAAGAAAACCAGGAGTGAA	sgRNA
P4	ACAAATATTTAAACTCGGGACCTGG	sgRNA
P5	GCGGCCGCAAGTGATTAGACT	sgRNA
P6	GCAGCTCAGTGATTAAGAGTAAAGATGG	sgRNA
DUN1-Re-F	gaggttattgggtttagtataattctatctgatgatgagtaaccgaattgggaaatactccaatacctcg ccagaac	repair DNA for *DUN1*
DUN1-Re-R	TGTTTCATCAATTCTTAATTTTGATTTGTTAATTATTCGTTGAAATAAT TCTCCGCTATTCTCACTATAGGGAGACCG	repair DNA for *DUN1*
DUN1-Te-F	ggacaaaaatagtgcccaac	genotype confirmation for *DUN1*
DUN1-Te-R	GTGGCAGCAGTGGAGGTAA	genotype confirmation for DUN1
DUN1-sg-F	actattgatacaacaacaatGTTTTAGAGCTAGAAATAGCAAG	sgRNA for *DUN1*
DUN1-sg-R	ATTGTTGTTGTATCAATAGTCAAATTAAAAATAGTTTACGCA	sgRNA for *DUN1*
RAD9-sg-F	caagaaatgattaatcaagaGTTTTAGAGCTAGAAATAGCAAG	sgRNA for *RAD9*
RAD9-sg-R	TCTTGATTAATCATTTCTTGCAAATTAAAAATAGTTTACGCA	sgRNA for *RAD9*
RAD9-Re-F	gggggaggggagaatttttatttcattacacggtttaaaattatgataatataggtgatccaatacctcg ccagaac	repair DNA for *RAD9*
RAD9-Re-R	TCTTTATAACATAACATGGATTTGACACACCAACCCTCAACCTAACA CTTGGATATGACTCACTATAGGGAGACCG	repair DNA for *RAD9*
RAD9-Te-F	gaatggaggaaagcaaaaac	genotype confirmation for *RAD9*
RAD9-Te-R	GAGGAGGAGGAAGAGGTC	genotype confirmation for RAD9
ADE4-qPCR-F	tccgtgaagggtttgtta	RT-PCR
ADE4-qPCR-R	CTAATGCCTTGGTGTCAG	RT-PCR
RNR3-qPCR-F	gactggactttgttttcacc	RT-PCR
RNR3-qPCR-R	GCGGTTTCTTTTGGTGACG	RT-PCR
HOF1-RT-F	ctacagacattccaacagc	RT-PCR
HOF1-RT-R	GGAGCAGTAGTCGCAATGA	RT-PCR
FKH2-sg-F	aagtcattggcagtttcgatGTTTTAGAGCTAGAAATAGCAAG	sgRNA for *FKH2*
FKH2-sg-R	ATCGAAACTGCCAATGACTTCAAATTAAAAATAGTTTACGCA	sgRNA for *FKH2*

FKH2-Re-F	ttgctaggatttacgattatttacaaatgtcagcacaatttatcacaccgaaaaagtccaatacctcgc cagaac	repair DNA for *FKH2*
FKH2-Re-R	AAGTTTAATCCAATGTCTCCATTTGTTGTTGTAGAAGCAGTTGGGGC AGTTGAAGTACCTCACTATAGGGAGACCG	repair DNA for *FKH2*
FKH2-Te-F	gtatgtgtgtgtgagaggc	genotype confirmation for *FKH2*
FKH2-Te-R	GCAGTTATCTTTTTGGGAC	genotype confirmation for *FKH2*
FKH2-F-Resem	aacaacaacatcgataccgtcgaccgctaggatttacgattatttaca	Clone FKH2
FKH2-R-Resem	AGGGAACAAAAGCTGGGTACGCAGTTATCTTTTTGGGAC	Clone FKH2
MCM1-Promoter-sgF	aacaattgttcatagataatGTTTTAGAGCTAGAAATAGCAAG	sgRNA for *MCM1*
MCM1-Promoter-sgR	ATTATCTATGAACAATTGTTCAAATTAAAAATAGTTTACGCA	sgRNA for *MCM1*
MCM1-Promoter-reF	cttttccaatcttttattcaaactcaaaaaacaacaacaaccaaaaaaattttattttcggatccggat ggtataaacg	repair DNA for *MCM1*
MCM1-Promoter-reR	TGAATGGGAATTCCCCTCATTACCTTGACTAAATTCATTTGTTTCTT CTTTAATAGCCATCATGTTTTCTGGGGAGGGTA	repair DNA for *MCM1*
MCM1-Promoter-TeF	ctccttgttcactgtctacc	genotype confirmation for *MCM1*
MCM1-TR	TAACAACACCCCTAACGAC	genotype confirmation for *MCM1*
MCM1-RT-F	cactaaatctgaagggaag	RT-PCR
MCM1-RT-R	TCTCCTGTATTTCCATCAC	RT-PCR
FKH2-RT-F	cagttgttgccccgaaaatg	RT-PCR
FKH2-RT-R	GGGAATGAGTTAGATGAGC	RT-PCR
HOF1-Chip-F	gaaaaggtccatactcaaagc	ChIP
HOF1-Chip-R	ACTGGTCCTACTTGTTTCA	ChIP
FKH2-Tag-F	aatccatctatttctaacaacacaccaaagatggctaaaggcacaggtatgtattttaaagctgcag gtcgacggatc	Tag primers
CaRad53-Tag-F	caaacctcagaagaaatgaatattattccacttatttcgggtttaagtagtataagttcagctgcaggtc gacggatc	Tag primers
CaRad53-Tag-R	GTAAATGTAATTGCATGGAGGTAAATAACTAAATTAATGAACCTTTG TTTCTATAGGAAACTCACTATAGGGAGACCG	Tag primers
MCM1-Tag-F	caatacctgtattttggtaatattcaaaataacaacatacctaatcaacagcaatatcaagctgcagg tcgacggatc	Tag primers

MCM1-Tag-R	TATTCACCTAAATCCCCTGACCTCTGGCCAAACACTTTCTTTGTAGA TGGGAGGGGAGCGCTCACTATAGGGAGACCG	Tag primers
Ca-GAPDH-NF	cattgaagttgtcgccgtc	RT-PCR
Ca-GAPDH-NR	GAGCACCTTCGAGTTTGG	RT-PCR
HOF1-Pro-F1	aatacgactcactatagggcgaattcgcaacagccttttatccat	Yeast One hybrid
HOF1-Pro-F1	aatacgactcactatagggcgaattccgaagcgttgaagaagat	Yeast One hybrid
HOF1-Pro-F3	aatacgactcactatagggcgaattcggtccatactcaaagcaa	Yeast One hybrid
NewHOF1-Pro-R1	ggatcgattcgcgaacgcgtgagctcATCTTCTTCAACGCTTCG	Yeast One hybrid
New HOF1-Pro-R	ggatcgattcgcgaacgcgtgagctcACATCAAGGAAGAACGAGG	Yeast One hybrid
