# Supplementary material for: DNA Damage Checkpoints Govern Global Gene Transcription and Exhibit Species-Specific Regulation on HOF1 in Candida albicans
Source: J Fungi (Basel). 2024 May 29;10(6):387. doi: 10.3390/jof10060387 (PMC11204775; doi:10.3390/jof10060387)
Supplement: Supplementary file 1 [file jof-10-00387-s001.zip › Table S3.pdf]

Gene	Gene function	log2(fc)	PValue
orf19.4653	Protein similar to GPI-linked cell-wall proteins; induced in low iron; Spider biofilm induced; regulated in Spider biofilms by Bcr1, Tec1, Ndt80, Brg1	14.91462	3.15E-12
orf19.6487	**Ortholog of *C. parapsilosis CDC317* : CPAR2_808350, *C. dubliniensis CD36* : Cd36_72060, *Candida metapsilosis* : CMET_5893 and *Candida orthopsilosis Co 90-125* : CORT_0C00820**	14.75473	2.22E-13
LDG3	Putative LDG family protein; F-12/CO2 early biofilm induced	14.29491	3.03E-11
orf19.2633.1	**Ortholog of *C. dubliniensis CD36* : Cd36_00150, *Lodderomyces elongisporus NRLL YB-4239* : LELG_01269 and *Candida tropicalis NEW ASSEMBLY* : CTRG1_CGOB_00075**	14.28987	1.76E-07
orf19.2071	Protein of unknown function; induced by alpha pheromone in SpiderM medium	11.21977	0.014647
orf19.4011	Similar to S. pombe mug180, a predicted esterase/lipase; highly induced during chlamydospore formation in both C. albicans and C. dubliniensis; flow model biofilm induced	11.09672	0.000011
CSA2	Extracellular heme-binding protein involved in heme-iron acquisition; regulated by Tsa1, Tsa1B in minimal media at 37 deg; induced by ketoconazole, nitric oxide, Hap43; required for normal RPMI biofilm formation	11.07347	0.010961
orf19.2899	**Ortholog of *C. dubliniensis CD36* : Cd36_45850, *C. parapsilosis CDC317* : CPAR2_500750, *Pichia stipitis Pignal* : PICST_43336, *Candida tropicalis MYA-3404* : CTRG_04038 and *Candida albicans WO-1* : CAWG_03191**	10.95855	0.000807
orf19.4069	Protein of unknown function; repressed by alpha pheromone in SpiderM medium (5)	10.85331	0.010342
orf19.6484	**Ortholog of *C. parapsilosis CDC317* : CPAR2_808370, *C. dubliniensis CD36* : Cd36_72070, *Candida metapsilosis* : CMET_5895 and *Candida orthopsilosis Co 90-125* : CORT_0C00800**	10.61335	7.37E-53
CDA2	Putative chitin deacetylase; transcription is positively regulated by Tbf1p	9.965784	0.003742
HIS1	ATP phosphoribosyl transferase; enzyme of histidine biosynthesis; acid upregulated/alkaline repressed by Rim101; regulated by Gcn2, Gcn4; strain CA9 is a his1 mutant; flow model biofilm induced; Spider biofilm repressed	9.762606	4.15E-10

orf19.4571	CoA-transferase family protein; rat catheter biofilm repressed	9.344296	0.005436
REC8	Protein involved in control of chromosome stability and homologous recombination during meiosis-like concerted chromosome loss (CCL) process that leads to depolyploidization after mating	9.090112	0.009326
PGA31	Cell wall protein; putative GPI anchor; expression regulated upon white-opaque switch; induced by Congo Red and cell wall regeneration; Bcr1-repressed in RPMI a/a biofilms	6.898111	1.52E-90
PGA23	Putative GPI-anchored protein of unknown function; Rim101-repressed; Cyr1-regulated; colony morphology-related gene regulation by Ssn6	6.551304	2.92E-58
FET99	Multicopper oxidase family protein; similar to S. cerevisiae Fet3; does not complement S. cerevisiae fet3 mutant growth under low-iron; iron-repressed; regulated by Tup1, Rim101; flow model biofilm induced; Spider biofilm repressed	6.515077	5.12E-20
orf19.2197	Has domain(s) with predicted N,N-dimethylaniline monooxygenase activity, NADP binding, flavin adenine dinucleotide binding activity	6.467606	0.004219
MRV8	Membrane protein involved in mycelial growth, biofilm formation and epithelial damage; Spider biofilm induced	6.358779	4.92E-53
orf19.3932.1	Has domain(s) with predicted nucleic acid binding, nucleotide binding activity	6.244048	4.27E-05
orf19.3337	Protein of unknown function; merged with orf19.3338; rat catheter, flow and Spider model biofilm induced; promoter bound by Bcr1, Efg1, Ndt80, and Rob1; orf19.3338 Bcr1-repressed in RPMI a/a biofilms	6.044394	4.92E-05
orf19.1968.1	Predicted non-catalytic subunit of N-terminal acetyltransferase; Spider biofilm induced	6.001877	0.011316
orf19.7452	Ortholog(s) have chromatin binding activity and role in DNA replication initiation, DNA unwinding involved in DNA replication, double-strand break repair via break-induced replication	5.794416	7.27E-06
AOX2	Alternative oxidase; cyanide-resistant respiration; induced by antimycin A, oxidants; growth; Hap43, chlamydospore formation repressed; rat catheter, Spider biofilm induced; regulated in Spider biofilms by Bcr1, Tec1, Ndt80, Brg1	5.725504	0.005119
RBR1	Glycosylphosphatidylinositol (GPI)-anchored cell wall protein; required for filamentous growth at acidic pH; expression repressed by Rim101 and activated by Nrg1; Hap43-induced	5.699459	6.56E-16

CFL5	Ferric reductase; induced in low iron; ciclopirox olamine, flucytosine induced; amphotericin B, Sfu1 repressed; Tbf1, Hap43 induced	5.653858	1.66E-09
orf19.3499	Secreted potein; Hap43-repressed; fluconazole-induced; regulated by Tsa1, Tsa1B under H2O2 stress conditions; induced by Mnl1p under weak acid stress; Spider biofilm induced	5.587161	5.91E-36
NIP7	Putative nucleolar protein with role in ribosomal assembly; hyphal-induced; Hap43-induced; Spider biofilm induced	5.151405	0.001343
orf19.344	Protein of unknown function; upregulated by fluphenazine treatment or in an azole-resistant strain that overexpresses CDR1 and CDR2; transcript possibly regulated by Tac1	5.097261	3.35E-12
orf19.5353	**Ortholog of *C. dubliniensis CD36* : Cd36_24630, *C. parapsilosis CDC317* : CPAR2_407790, *Candida tropicalis MYA-3404* : CTRG_02045, *Candida albicans WO-1* : CAWG_06134 and *Candida metapsilosis* : CMET_2127**	4.887873	6.66E-05
ARG11	Putative ornithine transporter of the mitochondrial inner membrane; induced during the mating process	4.868959	3.64E-07
orf19.1430	**Ortholog of *C. dubliniensis CD36* : Cd36_43870, *C. parapsilosis CDC317* : CPAR2_402120, *Candida tropicalis MYA-3404* : CTRG_05742 and *Candida albicans WO-1* : CAWG_03407**	4.861087	0.000174
BMT7	Beta-mannosyltransferase, member of a 9-gene family that includes characterized genes BMT1, BMT2, BMT3, and BMT4 with roles in beta-1,2-mannosylation of cell wall phosphopeptidomannan; downregulated in azole-resistant strain; Hap43p-induced	4.827413	1.26E-17
CRZ2	C2H2 transcription factor, involved in regulation of early adaptation to murine GI tract; Rim101-repressed at pH 8; required for yeast cell adherence to silicone substrate; Spider biofilm induced	4.675895	4.89E-16
orf19.5704	Ortholog(s) have rRNA binding activity, role in RNA splicing, mitochondrial RNA processing, mitochondrial genome maintenance, positive regulation of rRNA processing, rRNA metabolic process and mitochondrion localization	4.606201	5.88E-13
orf19.1438	Protein with homology to NADH dehydrogenase; regulated by Sef1p-, Sfu1p-, and Hap43p	4.477772	2.94E-10
orf19.7077	Putative ferric reductase; induced by Mac1 under copper starvation; Plc1-regulated; Rim101-repressed	4.377193	2.03E-17

orf19.6366	Has domain(s) with predicted RNA binding activity	4.374737	2.09E-21
orf19.5592	**Ortholog of *C. dubliniensis CD36* : Cd36_63980, *Candida tenuis NRRL Y-1498* : CANTEDRAFT_115187, *Candida tropicalis NEW ASSEMBLY* : CTRG1_02719 and *Candida tropicalis MYA-3404* : CTRG_02719**	4.360256	0.005854
**Ortholog of *C. dubliniensis CD36* : Cd36_30690, *C. parapsilosis CDC317* : CPAR2_204140, *Debaryomyces hansenii CBS767* : DEHA2F16940g and *Candida tropicalis MYA-3404* : CTRG_00570**		4.354981	1.17E-19
orf19.5141	**Ortholog of *Candida albicans WO-1* : CAWG_05647**	4.206451	0.008272
orf19.2445	Putative dicarboxylic amino acid permease; fungal-specific (no human or murine homolog); induced by alpha pheromone in SpiderM medium (6)	4.205343	5.85E-06
CAG1	Heterotrimeric G protein alpha subunit; positive role in mating pheromone response; opaque-enriched transcript; transcript repressed by MTLa1-MTLalpha2; regulated by hemoglobin-responsive Hbr1 via MTL genes; rat catheter biofilm repressed	4.193308	0.000325
orf19.7455	**Ortholog of *C. dubliniensis CD36* : Cd36_86630, *Candida tropicalis NEW ASSEMBLY* : CTRG1_05698 and *Candida tropicalis MYA-3404* : CTRG_05698**	4.181588	2.71E-09
orf19.7463	Ortholog(s) have role in ascospore wall assembly and ascospore wall, nuclear envelope localization	4.177538	7.04E-05
orf19.2916	**Ortholog of *C. dubliniensis CD36* : Cd36_45740, *C. parapsilosis CDC317* : CPAR2_401900, *Candida tenuis NRRL Y-1498* : CANTEDRAFT_116046 and *Pichia stipitis Pignal* : PICST_28890**	4.173457	0.017205
orf19.5896	Protein of unknown function; induced by alpha pheromone in SpiderM medium (4)	4.090698	1.14E-05
orf19.3897	Protein of unknown function; decreased transcription is observed upon fluphenazine treatment or in an azole-resistant strain that overexpresses CDR1 and CDR2; transcription is repressed in response to alpha pheromone in SpiderM medium	4.038919	0.001321
AGT1	Agmatinase, involved in metabolism of agmatine; downregulated upon adherence to polystyrene; regulated by Gcn2p and Gcn4p	4.023952	5.45E-06
MDJ1	Putative member of the HSP40 (DnaJ) family of chaperones; rat catheter and Spider biofilm induced	4.019689	2.51E-28
EXG2	GPI-anchored cell wall protein, similar to S. cerevisiae exo-1,3-beta-glucosidase Exg2p; predicted Kex2p	4.005087	1.13E-08

	substrate; induced during cell wall regeneration; possibly an essential gene, disruptants not obtained by UAU1 method; Hap43p-repressed		
ZCF22	**Predicted Zn(II)2Cys6 transcription factor**	3.992338	1.02E-06
orf19.3404	Protein of unknown function; transcription repressed by fluphenazine treatment	3.951644	8.72E-06
orf19.6723	Protein of unknown function; Spider biofilm induced	3.874019	1.44E-07
PGA39	Putative GPI-anchored protein	3.810255	0.00392
orf19.3954.1	Ortholog of ***C. parapsilosis CDC317*** **: CPAR2_101170,** ***C. auris B8441*** **: B9J08_004296,** ***Candida tenuis NRRL Y-1498*** **: CANTEDRAFT_113855 and** ***Debaryomyces hansenii CBS767*** **: DEHA2G20240g**	3.809157	0.000351
PGA57	Putative GPI-anchored protein; Hap43p-induced gene	3.805376	8.18E-10
BMT3	Beta-mannosyltransferase; adds 2nd beta-mannose to the acid-stable fraction of cell wall phosphopeptidomannan, elongation of beta-mannose chains on the phosphopeptidomannan acid-labile fraction; Hap43-induced; Spider biofilm induced	3.747878	5.31E-11
orf19.4046	Putative transcription factor containing a Zn(2)-Cys(6) binuclear cluster	3.741849	3.35E-19
orf19.4394	Protein of unknown function; induced by alpha pheromone in SpiderM medium	3.739291	0.01408
orf19.2457	Protein of unknown function; induced during chlamydospore formation in both C. albicans and C. dubliniensis; Spider biofilm induced	3.736966	0.008565
orf19.1434	Ortholog(s) have DNA polymerase binding, protein kinase activator activity, signaling adaptor activity	3.648565	2.99E-15
CCP2	Has domain(s) with predicted heme binding, peroxidase activity and role in response to oxidative stress	3.625748	5.26E-07
HBR3	Essential protein; regulated by hemoglobin; S. cerevisiae ortholog is essential; Hap43p-induced gene	3.60185	3.5E-15
RBF1	Transcription factor; glutamine-rich activation domain; binds RPG-box DNA sequences; predominantly nuclear; mutation causes accelerated induction of filamentous growth; antigenic during human oral infection; Sko1p-repressed	3.598979	2.77E-14
orf19.6398	S. pombe ortholog SPBC460.04c is a predicted sulfonate/alpha-ketoglutare dioxygenase; induced by nitric oxide; Spider biofilm induced	3.572326	2.56E-05

MNN1	Putative alpha-1,3-mannosyltransferase; of the mannosyltransferase complex; negatively regulated by Rim101; transcript elevated in chk1 and nik1 mutants, but not in sln1 mutant; Spider and flow model biofilm induced	3.560868	3.37E-19
orf19.2449	Putative adhesin-like protein; macrophage-induced gene	3.558967	0.016738
orf19.3894	Putative inositol oxygenase; mutation confers hypersensitivity to toxic ergosterol analog; Spider biofilm induced	3.557127	5.44E-12
orf19.711	**Protein of unknown function; induced by nitric oxide; predicted ORF from Assembly 19; removed from Assembly 20; restored based on transcription data**	3.553212	1.2E-15
BDF1	Essential chromatin-binding bromodomain protein; repressed upon adherence to polystyrene; reduced mRNA abundance detected in null mutant; macrophage/pseudohyphal-repressed	3.552948	6.23E-20
orf19.4668	Protein with a glycoside hydrolase domain; mutants are viable	3.539724	3.29E-11
orf19.1955	Ortholog(s) have palmitoyltransferase activity, role in protein palmitoylation, protein targeting to membrane and endoplasmic reticulum palmitoyltransferase complex, extrinsic component of endoplasmic reticulum membrane localization	3.531746	4.64E-05
orf19.2320	Putative serine/threonine-protein kinase; possibly an essential gene, disruptants not obtained by UAU1 method	3.529032	2.79E-10
RSM22	Predicted mitochondrial small ribosomal subunit; rat catheter and Spider biofilm induced	3.523368	2.03E-11
SAP4	Secreted aspartyl proteinase; sap4,5,6 mutant defective in protein utilization for nitrogen; virulence role complicated by URA3 effects; expressed during mucosal and systemic infections; N-glycosylated; rat catheter, Spider biofilm induced	3.498359	6.77E-05
SUA5	Ortholog(s) have N(6)-L-threonylcarbamoyladenine synthase activity, single-stranded telomeric DNA binding activity	3.498083	1.28E-17
MAK5	Putative nucleolar DEAD-box RNA helicase; oxidative stress-repressed via Cap1; repressed by prostaglandins	3.49595	1.01E-09
TBF1	Essential transcription factor; induces ribosomal protein genes and the rDNA locus; acts with Cbf1 at subset of promoters; recruits Fhl1 and Ifh1 to promoters; role is analogous to that of S. cerevisiae Rap1; Spider biofilm induced	3.493516	1E-11

orf19.7361	Ortholog(s) have tRNA-intron endonuclease activity, role in tRNA-type intron splice site recognition and cleavage and mitochondrial outer membrane, tRNA-intron endonuclease complex localization	3.483204	2.35E-08
GRF10	Putative homeodomain transcription factor, involved in control of filamentous growth; null mutant is an adenine auxotroph; Spider biofilm induced; promoter bound by Bcr1, Tec1, Efg1, Ndt80 and Brg1	3.465275	1.41E-10
orf19.3378	Protein of unknown function; regulated by Tsa1, Tsa1B in minimal media at 37 degrees C	3.456259	4.87E-07
ECE1	**Candidalysin, cytolytic peptide toxin essential for mucosal infection; hypha-specific protein; regulated by Rfg1, Nrg1, Tup1, Cph1, Efg1, Hog1, farnesol, phagocytosis; fluconazole-induced; rat catheter and Spider biofilm induced**	3.455357	2.68E-18
orf19.4160	Ortholog(s) have N(6)-L-threonylcarbamoyladenine synthase activity and role in mitochondrial tRNA threonylcarbamoyladenosine modification, tRNA threonylcarbamoyladenosine modification	3.453376	1.76E-12
orf19.270	**Ortholog of *C. dubliniensis CD36* : Cd36_82780, *C. parapsilosis CDC317* : CPAR2_102150, *Pichia stipitis Pignal* : psti_CGOB_00155 and *Candida tropicalis MYA-3404* : CTRG_02557**	3.45016	2.38E-10
STE3	Protein similar to S. cerevisiae Ste3p, the receptor for a-factor mating pheromone; alpha mating-type-specific transcription	3.448226	0.000541
orf19.3970	**Putative ribosome biogenesis factor; possibly essential, disruptants not obtained by UAU1 method; rat catheter and Spider biofilm induced**	3.438702	2.25E-09
orf19.2247	Ortholog of C. dubliniensis CD36 : Cd36_21220, C. parapsilosis CDC317 : CPAR2_406700, Candida tenuis NRRL Y-1498 : CANTEDRAFT_127772 and Candida tropicalis MYA-3404 : CTRG_01766 (5)	3.431253	0.012638
orf19.6950	Putative vacuolar membrane transporter for cationic amino acids; Spider biofilm induced	3.420468	0.003259
CRD2	Metallothionein; for adaptation to growth in high copper; basal transcription is cadmium-repressed; Ssn6 regulated; complements copper sensitivity of an S. cerevisiae cup1 mutant; regulated by Sef1, Sfu1, and Hap43; Spider biofilm induced	3.417632	0.000827
orf19.7624	Ortholog(s) have role in maturation of SSU-rRNA from tricistronic rRNA transcript (SSU-rRNA, 5.8S rRNA, LSU-rRNA), rRNA processing and nucleolus, small-subunit processome localization	3.409541	1.47E-11

PGA37	Putative GPI-anchored protein; Hap43-repressed; Spider biofilm induced	3.397576	8.45E-05
UTP21	Putative U3 snoRNP protein; Hap43-induce; physically interacts with TAP-tagged Nop1; Spider biofilm induced	3.378998	6.57E-11
orf19.1920	**Ortholog of *C. dubliniensis CD36* : Cd36_15040**	3.370493	0.000105
orf19.2049	Plasma membrane-associated protein; heterozygous null mutant displays sensitivity to virgineone; Spider biofilm induced	3.36334	4.76E-14
orf19.5290	Protein of unknown function; repressed by Sfu1; Hap43-induced gene	3.353556	2.45E-06
orf19.7552	Putative U3-containing small subunit processome complex protein; Hap43-induced gene; repressed in core stress response; Spider biofilm induced	3.352368	1.42E-12
MRPL8	Mitochondrial 60S ribosomal protein subunit; Hap43p-repressed gene	3.332274	3.37E-20
orf19.7085	Protein of unknown function; induced in core stress response; induced by cadmium stress via Hog1; oxidative stress-induced via Cap1; induced by Mnl1 under weak acid stress; macrophage-repressed; rat catheter and Spider biofilm induced	3.317811	1.41E-22
GLE1	Putative nucleoporin; moderately induced at 42 degrees C	3.316609	1.55E-10
ULP3	SUMO deconjugation enzyme that removes the small ubiquitin-like modifier (SUMO) from proteins	3.316535	3.33E-07
RPC53	Ortholog(s) have RNA polymerase III activity, role in tRNA transcription by RNA polymerase III and RNA polymerase III complex localization	3.315702	9.04E-09
HGT10	Glycerol permease involved in glycerol uptake; member of the major facilitator superfamily; induced by osmotic stress, at low glucose in rich media, during cell wall regeneration; 12 membrane spans; Hap43p-induced gene	3.310813	1.53E-20
orf19.416	**Ortholog of *C. dubliniensis CD36* : Cd36_05200, *C. parapsilosis CDC317* : CPAR2_107660, *C. auris B8441* : B9J08_003698 and *Candida tenuis NRRL Y-1498* : cten_CGOB_00159**	3.309406	8.3E-06
SMC4	Ortholog(s) have ATPase, DNA/DNA annealing activity, chromatin binding, double-stranded DNA binding, single-stranded DNA binding activity	3.306025	7.12E-12
PGA58	Putative GPI-anchored protein; transcription is positively regulated by Tbf1p	3.303275	4.98E-12

orf19.2917	Putative GTPase; heterozygous null mutant exhibits resistance to parnafungin in the C. albicans fitness test; Hap43p-induced gene	3.295706	6.89E-16
orf19.2639	Ortholog(s) have structural constituent of ribosome activity and mitochondrial large ribosomal subunit localization	3.284997	1.22E-18
orf19.1956	Ortholog(s) have role in reciprocal meiotic recombination and mitochondrial matrix, mitochondrion localization	3.282336	1.44E-19
MUB1	Predicted protein required for ubiquitination; role in meiosis, regulation of cell budding in S. cereviae; Spider biofilm induced	3.28132	1.24E-08
orf19.494	**Putative RNA-binding protein; role in assembly of box H/ACA snoRNPs and thus pre-rRNA processing; Spider biofilm induced**	3.280239	1.82E-09
BRE1	Putative E3 ubiquitin ligase with RING-type zinc finger domain; involved in ubiquitination of histone H2B during hyphal development; transposon mutation affects filamentous growth	3.279873	1.08E-14
CUP2	Putative copper-binding transcription factor; required for normal resistance to copper; activates transcription of metallothionein genes; Hap43-repressed; Spider biofilm induced	3.278582	3.38E-08
TAZ1	Putative lyso-phosphatidylcholine acyltransferase, required for normal phospholipid content of mitochondrial membranes; rat catheter biofilm induced	3.27635	2.15E-11
INO1	Inositol-1-phosphate synthase; antigenic in human; repressed by farnesol in biofilm or by caspofungin; upstream inositol/choline regulatory element; glycosylation predicted; rat catheter, flow model induced; Spider biofilm repressed	3.274327	1.51E-05
SSK1	Response regulator of two-component system; role in oxidative stress response, cell wall biosynthesis, virulence, hyphal growth on solid media; expressed in hyphae and yeast; peroxisomal targeting sequence (PTS1); Spider biofilm induced	3.273972	3.06E-17
PAM16	Putative maltase; regulated by Gcn4; repressed by amino acid starvation (3-AT); rat catheter biofilm induced	3.273606	1.3E-17
DAL8	Putative allantoate permease; fungal-specific (no human or murine homolog)	3.268972	4.85E-06
CGT1	mRNA 5' guanylyltransferase; small subunit of mRNA capping enzyme; binds an RNA Pol II C-terminal domain peptide; Cet1p and Cgt1p form a 2:1 complex; functional homolog of S. cerevisiae Ceg1p	3.267885	1.89E-09

orf19.4721	Ortholog(s) have RNA binding activity, role in mRNA processing, mitochondrial translation and mitochondrion localization	3.264307	4.02E-09
DOT4	Protein similar to ubiquitin C-terminal hydrolase; localizes to cell surface of hyphal cells, but not yeast-form cells; repressed upon high-level peroxide; Hap43p-induced; rat catheter biofilm induced	3.263317	2.24E-09
orf19.4245	Protein with a predicted pleckstrin domain; Hap43-repressed gene	3.258072	9.75E-10
SOD3	Cytosolic manganese-containing superoxide dismutase; protects against oxidative stress; repressed by ciclopirox olamine, induced during stationary phase when SOD1 expression is low; Hap43-repressed; Spider and flow model biofilm induced	3.253382	9.14E-17
SUA72	Predicted transcription factor; induced during planktonic growth, whereas related SUA71 is downregulated	3.244061	0.000101
orf19.6227	**Ortholog of *C. dubliniensis CD36* : Cd36_06390, *C. parapsilosis CDC317* : CPAR2_209040, *C. auris B8441* : B9J08_003388 and *Candida tenuis NRRL Y-1498* : CANTEDRAFT_114052**	3.235955	7.46E-09
orf19.5991	**Ortholog(s) have role in assembly of large subunit precursor of preribosome, maturation of 5.8S rRNA from tricistronic rRNA transcript**	3.21021	4.32E-13
SDA1	Predicted nuclear protein involved in actin cytoskeleton organization, passage through Start, 60S ribosome biogenesis; rat catheter biofilm induced; Hap43-induced	3.199097	4.36E-11
orf19.1117	Protein similar to Candida boidinii formate dehydrogenase; virulence-group-correlated expression; Hap43-repressed; Spider biofilm repressed	3.176779	0.013117
NOP14	Putative nucleolar protein; Hap43-induced; mutation confers resistance to 5-fluorocytosine (5-FC), 5-fluorouracil (5-FU), and tubercidin (7-deazaadenosine); heterozygous mutant is resistant to parnafungin; Spider biofilm induced	3.163518	6.59E-11
HCA4	Putative role in regulation of cell wall biogenesis; Hap43p-induced gene; possibly an essential gene, disruptants not obtained by UAU1 method; flow model and rat catheter biofilm induced	3.159258	1.34E-08
orf19.6195	Ortholog(s) have 3'-5'-exoribonuclease activity	3.158251	3.94E-11
RER2	Putative cis-prenyltransferase involved in dolichol synthesis; participates in endoplasmic reticulum (ER) protein sorting; flow model biofilm induced	3.14623	6.97E-16

orf19.5879	Has domain(s) with predicted oxidoreductase activity and role in metabolic process	3.139471	3.51E-06
KSP1	Putative serine/threonine protein kinase; mRNA binds She3 and is localized to hyphal tips; mutation confers hypersensitivity to amphotericin B	3.118086	7.09E-07
FAR1	**Protein involved in regulation of pheromone-mediated mating; repressed by A1p and Alpha2p in white-phase cells; null mutant shows no pheromone response in opaque cells; overexpression causes enhanced pheromone response and cell cycle arrest**	3.116274	6.97E-09
CHS5	Putative chitin biosynthesis protein; fungal-specific; repressed upon yeast-to-hypha switch; rat catheter biofilm repressed	3.11255	1.32E-16
orf19.6244	**Ortholog of *C. dubliniensis CD36* : Cd36_06250, *C. parapsilosis CDC317* : CPAR2_206820, *C. auris B8441* : B9J08_003766 and *Candida tenuis NRRL Y-1498* : CANTEDRAFT_125137**	3.10516	1.77E-07
CAS5	Transcription factor involved in regulation of cell wall homeostasis, adherence, stress response; mutants have reduced CFU in mice, hyphal defect in C. elegans infection; Spider biofilm induced	3.103886	5.3E-08
orf19.3831	Ortholog(s) have enzyme activator activity, telomerase inhibitor activity, role in box C/D RNA 3'-end processing, negative regulation of telomere maintenance via telomerase and nucleolus, nucleoplasm localization	3.103234	1.09E-07
DDR48	Immunogenic stress-associated protein; filamentation regulated; induced by benomyl/caspofungin/ketoconazole or in azole-resistant strain; Hog1, farnesol, alkaline repressed; stationary phase enriched; Spider, flow model biofilm induced	3.097999	3.99E-14
STE4	Beta subunit of heterotrimeric G protein of mating signal transduction pathway; required for mating; transcript is specific to cells homozygous at MTL; induced by alpha pheromone; ortholog of S. cerevisiae Ste4	3.092246	0.002634
orf19.5277	Ortholog(s) have role in DNA recombination, nuclear-transcribed mRNA catabolic process, 3'-5' exonucleolytic nonsense-mediated decay, protein ubiquitination and cytoplasm, nucleolus, nucleus, polysome localization (5)	3.089081	1.58E-06
orf19.5952	Protein of unknown function; induced by nitric oxide independent of Yhb1; Sef1, Sfu1, and Hap43-induced; rat catheter and Spider biofilm induced	3.087717	3.34E-11
orf19.675	Cell wall protein; induced in core stress response and core caspofungin response; iron-regulated; amphotericin B, ketoconazole, and hypoxia induced; regulated by Cyr1,	3.085816	1.42E-12

	Ssn6; induced in oralpharyngeal candidasis; Spider biofilm repressed		
orf19.2520	Ortholog(s) have structural constituent of ribosome activity and mitochondrial small ribosomal subunit localization	3.078338	6.84E-11
orf19.6940	Protein of unknown function	3.076747	0.008911
orf19.4243	Ortholog(s) have endonuclease activity, ubiquitin binding activity and role in mRNA cleavage, nuclear-transcribed mRNA catabolic process, no-go decay	3.07371	0.015028
orf19.4455	Protein of unknown function; Spider biofilm induced	3.067263	1.54E-06
orf19.6853	Protein of unknown function; Spider biofilm repressed	3.048676	1.81E-12
ATG15	Putative lipase; fungal-specific (no human or murine homolog); Hap43p-repressed gene	3.047771	1.66E-15
orf19.7193	Specificity factor required for ubiquitination; role in protein targeting to vacuole; involved in ubiquitin-dependent protein catabolism via the multivesicular body sorting pathway; Spider biofilm induced	3.045965	1.35E-07
ECM331	GPI-anchored protein; mainly at plasma membrane, also at cell wall; Hap43, caspofungin-induced; Plc1-regulated; Hog1, Rim101-repressed; colony morphology-related regulated by Ssn6; induced by ketoconazole and hypoxia	3.045314	1.39E-18
orf19.5802	Ortholog(s) have transferase activity, role in maturation of SSU-rRNA and cytoplasm localization	3.038951	1.03E-05
MSM1	Mitochondrial methionyl-tRNA synthetase (MetRS); functionally complements methionine auxotrophy of an E. coli MetRS mutant; transcript regulated by Nrg1; flow model biofilm induced	3.037653	2.51E-08
orf19.6859	**Ortholog of *C. dubliniensis CD36* : Cd36_44860, *Candida tropicalis NEW ASSEMBLY* : CTRG1_04054, *Candida tropicalis MYA-3404* : CTRG_04054 and *Candida albicans WO-1* : CAWG_03295**	3.035624	0.013513
orf19.2404	Ortholog(s) have RNA binding, ribonuclease MRP activity, ribonuclease P activity, tRNA binding activity	3.02584	7E-08
RPO41	Putative mitochondrial RNA polymerase; repressed in core stress response; Spider biofilm induced	3.023835	6.04E-11
PIM1	ATP-dependent Lon protease; role in degradation of misfolded proteins in mitochondria, biogenesis and maintenance of mitochondria; rat catheter biofilm induced	3.023315	3.64E-11

orf19.3648	Ortholog(s) have crossover junction endodeoxyribonuclease activity, endodeoxyribonuclease activity	3.014337	7.21E-05
UTP18	**Putative U3 snoRNA-associated protein; Hap43-induced; repressed in core stress response; physically interacts with TAP-tagged Nop1**	3.012549	2.21E-13
orf19.938	**Ortholog of *C. dubliniensis CD36* : Cd36_50520, *C. parapsilosis CDC317* : CPAR2_304000, *C. auris B8441* : B9J08_000441 and *Candida tenuis NRRL Y-1498* : cten_CGOB_00233**	3.011713	0.013865
MSS116	Putative DEAD-box protein; required for efficient splicing of mitochondrial Group I and II introns; Hap43-induced; rat catheter biofilm induced	3.002769	3.88E-10
ASR1	Heat shock protein; transcript regulated by cAMP, osmotic stress, ciclopirox olamine, ketoconazole; repressed by Cyr1, Ras1; colony morphology-related regulated by Ssn6; stationary phase enriched; Hap43-induced; Spider biofilm induced	2.995781	6.6E-15
EAF7	Subunit of the NuA4 histone acetyltransferase complex	2.983286	5.48E-12
orf19.2394	Ortholog(s) have aldehyde dehydrogenase (NAD+) activity, role in response to furfural and intracellular anatomical structure localization	2.98236	0.009961
SPO1	Protein similar to phospholipase B; fungal-specific (no human or murine homolog)	2.975197	2.19E-05
orf19.6175	Putative 35S rRNA processing protein; Hap43-induced; repressed by prostaglandins; Spider biofilm induced	2.971731	6.13E-12
orf19.6083	Has domain(s) with predicted nucleic acid binding, nucleotide binding activity	2.969077	2.58E-08
orf19.2319	Putative nucleolar protein with a predicted role in pre-rRNA processing; Hap43-induced gene; repressed in core stress response	2.963654	3.92E-08
orf19.1426	Ortholog of S. cerevisiae Skg6; localizes to the cell cortex, cell bud neck, cell bud tip, incipient cellular bud site, and is membrane-bound; Spider biofilm induced	2.962999	1.25E-09
orf19.6871	Protein of unknown function; Hap43-induced; flow model biofilm repressed	2.962341	2.52E-07
orf19.2125	Protein of unknown function; GlcNAc-induced protein; Spider biofilm induced; rat catheter biofilm repressed	2.957063	2.41E-15
MAS2	Putative processing peptidase, catalytic (alpha) subunit; protein level decreases in stationary phase cultures	2.95307	8.57E-11
orf19.4330	**Ortholog of *C. dubliniensis CD36* : Cd36_52790, *Candida tropicalis NEW ASSEMBLY* : CTRG1_05968, *Candida tropicalis MYA-3404* :**	2.940838	0.011911

	**CTRG_05968 and *Candida albicans WO-1* : CAWG_04684**		
NAM2	Mitochondrial leucyl-tRNA synthetase	2.938599	0.000026
orf19.478	Protein required for fusion of cvt-vesicles and autophagosomes with the vacuole; plays a role in autophagy, protein targeting to vacuole and vesicle docking; flow model biofilm induced	2.932075	0.000184
orf19.6866	Putative U1-70K component of the U1 snRNP, involved in splicing; ortholog of S. cerevisiae SNP1; downregulated upon adherence to polystyrene	2.927975	1.53E-06
orf19.4349	Protein of unknown function; transcript repressed by elevated CO2; Spider biofilm induced	2.92759	1.3E-07
HSL1	Probable protein kinase involved in determination of morphology during the cell cycle of both yeast-form and hyphal cells via regulation of Swe1p and Cdc28p; required for full virulence and kidney colonization in mouse systemic infection	2.913519	4.53E-11
orf19.4895	**Ortholog of *C. dubliniensis CD36* : Cd36_09650, *C. parapsilosis CDC317* : CPAR2_805060, *C. auris B8441* : B9J08_004034 and *Candida tenuis NRRL Y-1498* : CANTEDRAFT_95780**	2.912889	2.82E-07
orf19.5430	Small-subunit processome component; repressed by prostaglandins	2.906203	2.6E-09
orf19.4476	Protein with a NADP-dependent oxidoreductase domain; transcript induced by ketoconazole; rat catheter and Spider biofilm induced	2.904549	0.000082
PES1	**Pescadillo homolog required for dispersal of biofilm cells into planktonic yeast cells; essential in yeast cells, not in hyphal cells; mutation confers hypersensitivity to 5-fluorocytosine, 5-fluorouracil, tubercidin**	2.897553	2.18E-11
orf19.6234	**Putative U2 snRNP component; mutation confers hypersensitivity to 5-fluorocytosine (5-FC), 5-fluorouracil (5-FU), and tubercidin (7-deazaadenosine); Hap43-induced, Spider biofilm induced**	2.892449	5.32E-07
NTO1	Putative histone acetyltransferase complex subunit; induced upon low-level peroxide stress; Spider biofilm induced	2.886816	4.46E-07
orf19.4068	Protein of unknown function; flow model biofilm induced	2.886343	1.79E-05
orf19.1522	Ortholog of ***C. dubliniensis CD36* : Cd36_16850, *C. parapsilosis CDC317* : CPAR2_211990, *C. auris***	2.883807	0.000266

	B8441 **: B9J08_001195 and *Candida tenuis NRRL Y-1498* : cten_CGOB_00169**		
SGD1	Predicted small ribosomal subunit biogenesis protein; repressed in core stress response; transcript increases in populations of cells exposed to fluconazole over multiple generations; Spider biofilm induced	2.874135	2.34E-06
orf19.660	Protein of unknown function; mRNA binds She3	2.873043	0.000634
orf19.7376	**Ortholog of *C. dubliniensis CD36* : Cd36_85880, *C. parapsilosis CDC317* : CPAR2_806870, *C. auris B8441* : B9J08_002007 and *Candida tenuis NRRL Y-1498* : CANTEDRAFT_96983**	2.871914	0.000094
PIN4	Protein with similarity to S. cerevisiae Pin4p; transposon mutation affects filamentous growth	2.866121	1.29E-06
HDA1	Histone deacetylase; inducer of filamentation; conserved deacetylation motif; regulates white-to-opaque switch frequency but not opaque-to-white switch; greater expression in white cells than opaque cells; inhibited by trichostatin-A	2.855491	5.53E-08
MNN15	Putative alpha-1,3-mannosyltransferase; predicted role in protein O-linked glycosylation; Spider biofilm induced	2.855087	7.14E-08
orf19.954	Putative DnaJ-like chaperone; Hap43-repressed gene	2.852137	3.34E-10
MST1	Ortholog(s) have threonine-tRNA ligase activity, role in mitochondrial threonyl-tRNA aminoacylation and mitochondrion localization	2.845252	2.58E-08
CSP1	Putative cell wall associated protein; gene only found in C. albicans and C. dubliniensis; highly upregulated during chlamydospore development in both species; localized to chlamydospore cell wall	2.843274	0.01001
NRP1	Ortholog(s) have cytoplasmic stress granule localization	2.842494	1.16E-05
RGA2	Putative GTPase-activating protein (GAP) for Rho-type GTPase Cdc42; involved in cell signaling pathways controlling cell polarity; induced by low-level peroxide stress; flow model biofilm induced	2.841812	1.92E-07
SMM1	Putative dihydrouridine synthase; Hap43-induced gene; rat catheter biofilm induced; Spider biofilm induced	2.839574	3.62E-06
PRA1	Cell surface protein that sequesters zinc from host tissue; enriched at hyphal tips; released extracellularly; binds to host complement regulators; mediates leukocyte adhesion and migration; immunogenic in mouse; produced at ambient pH	2.836131	0.001843

orf19.1687	Ortholog of S. cerevisiae Prp43, an RNA helicase in the DEAH-box family that functions in both RNA polymerase I and polymerase II transcript metabolism; Hap43-induced gene	2.828124	1.69E-08
orf19.1639	Has domain(s) with predicted oxidoreductase activity	2.827442	0.000048
ECM18	Ortholog of ***S. cerevisiae*** **: ECM18,** ***C. glabrata CBS138*** **: CAGL0B01969g,** ***C. parapsilosis CDC317*** **: CPAR2_103190,** ***C. auris B8441*** **: B9J08_000758 and** ***Debaryomyces hansenii CBS767*** **: DEHA2G08448g**	2.827212	2.48E-05
orf19.310	Putative mitochondrial protein with a predicted role in cell wall biogenesis; possibly an essential gene, disruptants not obtained by UAU1 method	2.827212	2.48E-05
PRP22	Putative RNA-dependent ATPase; induced upon adherence to polystyrene; induced by Mnl1p under weak acid stress	2.825835	3.34E-12
orf19.4929	Ortholog(s) have mRNA binding, translation regulator activity and role in mitochondrial cytochrome c oxidase assembly, positive regulation of mitochondrial translational initiation (5)	2.824175	2.49E-06
orf19.4596	Protein of unknown function; Spider biofilm induced	2.819955	0.000036
orf19.6919	**Ortholog of** ***C. dubliniensis CD36*** **: Cd36_71210,** ***C. parapsilosis CDC317*** **: CPAR2_702710,** ***C. auris B8441*** **: B9J08_002141 and** ***Candida tenuis NRRL Y-1498*** **: CANTEDRAFT_116256**	2.818928	1.38E-05
NOG2	Putative nucleolar GTPase; repressed by prostaglandins; Hap43-induced, rat catheter and Spider biofilm induced	2.808286	3.98E-14
PRN1	Protein with similarity to pirins; induced by benomyl and in response to alpha pheromone in SpiderM medium; transcript induced by Mnl1 in weak acid stress; rat catheter and Spider biofilm induced	2.806226	4.66E-06
SRP40	Putative chaperone of small nucleolar ribonucleoprotein particles; macrophage/pseudohyphal-induced; rat catheter biofilm induced	2.800665	1.09E-11
orf19.4305	Ortholog of ***C. dubliniensis CD36*** **: Cd36_52590,** ***C. parapsilosis CDC317*** **: CPAR2_100565,** ***Debaryomyces hansenii CBS767*** **: DEHA2G06908g and** ***Candida guilliermondii ATCC 6260*** **: PGUG_02858**	2.78915	0.002689
orf19.6899	Putative oxidoreductase; mutation confers hypersensitivity to toxic ergosterol analog; rat catheter and Spider biofilm induced	2.787694	2.2E-08
orf19.5681	**Ortholog of** ***C. dubliniensis CD36*** **: Cd36_50190,** ***C. parapsilosis CDC317*** **: CPAR2_302680,** ***C. auris***	2.78389	3.29E-12

	***B8441* : B9J08_004528 and *Candida tenuis NRRL Y-1498* : CANTEDRAFT_109949**		
orf19.5954	Ortholog(s) have ubiquitin ligase activator activity	2.783075	0.017943
orf19.4814	Ortholog of ***C. dubliniensis CD36* : Cd36_08960, *C. parapsilosis CDC317* : CPAR2_301680, *Candida tenuis NRRL Y-1498* : cten_CGOB_00021 and *Debaryomyces hansenii CBS767* : DEHA2F11088g**	2.781723	0.014303
NOT4	Putative E3 ubiquitin-protein ligase; required for maintenance, but not induction, of hyphal development; homozygous null mutant is avirulent in mouse systemic infection despite persistence in host; repressed in rat oral candidiasis	2.776229	2.02E-09
RRP6	Putative nuclear exosome exonuclease component; Hap43p-induced gene; mutation confers hypersensitivity to 5-fluorocytosine (5-FC), 5-fluorouracil (5-FU), and tubercidin (7-deazaadenosine)	2.773703	6.48E-09
orf19.7173	Ortholog of ***C. dubliniensis CD36* : Cd36_73730, *C. parapsilosis CDC317* : CPAR2_702310, *Candida tropicalis MYA-3404* : CTRG_05152 and *Candida albicans WO-1* : CAWG_05735**	2.77259	0.020272
IML1	Putative protein with a role in autophagy; rat catheter biofilm induced	2.77103	3.03E-06
orf19.4101	**Ortholog of *C. parapsilosis CDC317* : CPAR2_104840, *C. auris B8441* : B9J08_001223, *Candida tenuis NRRL Y-1498* : CANTEDRAFT_116070 and *Debaryomyces hansenii CBS767* : DEHA2F04994g**	2.765673	0.001422
PRP5	Putative pre-mRNA processing RNA-helicase; induced upon adherence to polystyrene; rat catheter and Spider biofilm induced	2.764172	5.55E-06
LIP5	Cold-activated secreted lipase, differentially expressed lipase gene family member with possible roles in nutrition and acidic microenvironment; LIP5 and LIP8 expressed at all stages of mucosal and systemic infection; affects filamentation	2.760601	6.72E-06
orf19.6166	**Ortholog of *C. dubliniensis CD36* : Cd36_80810, *C. parapsilosis CDC317* : CPAR2_101800, *C. auris B8441* : B9J08_002362 and *Candida tenuis NRRL Y-1498* : CANTEDRAFT_103482**	2.758538	5.77E-05
PHO80	Ortholog(s) have cyclin-dependent protein serine/threonine kinase regulator activity	2.756661	0.000255

orf19.7042	Protein of unknown function; induced by benomyl or in an azole-resistant strain overexpressing MDR1; induced by nitric oxide; Spider biofilm induced	2.754888	5.77E-06
orf19.4563	Protein of unknown function; repressed by prostaglandins; Hap43-induced, Spider biofilm induced	2.752633	2.95E-09
orf19.6252	Ortholog(s) have RNA polymerase II complex binding activity	2.75115	3.76E-10
orf19.2202	Protein of unknown function; induced by alpha pheromone in SpiderM medium	2.74104	0.001027
orf19.4252	Ortholog(s) have protein serine/threonine kinase activity	2.734172	3.45E-09
PAC1	Ortholog(s) have microtubule binding, microtubule plus-end binding activity	2.733948	3.47E-05
FZO1	Mitochondrial biogenesis protein; rat catheter and Spider biofilm induced	2.732312	5.47E-08
ZCF35	Zn(II)2Cys6 transcription factor; Hap43-induced; Spider biofilm induced	2.726779	5.25E-05
RSC8	Component of the RSC chromatin remodeling complex; possibly an essential gene, disruptants not obtained by UAU1 method	2.72202	1.29E-12
MEF1	Ortholog(s) have role in mitochondrial translation and mitochondrion localization	2.719869	4.44E-09
RSM24	Ortholog(s) have structural constituent of ribosome activity and mitochondrial small ribosomal subunit localization	2.719576	1.29E-12
AES4	Protein of unknown function; Hap43-repressed gene	2.719458	0.000164
CEF1	Putative ribosome-associated protein; ortholog of S. cerevisiae Tma16; Hap43-induced gene; Spider biofilm induced	2.719363	0.000005
orf19.7422	Ortholog(s) have role in endonucleolytic cleavage in 5'-ETS of tricistronic rRNA transcript (SSU-rRNA, 5.8S rRNA and LSU-rRNA)	2.718695	6.53E-11
RAD50	**Putative DNA double-strand break repair factor; involved in response to oxidative stress and drug resistance; flow model biofilm repressed**	2.716388	3.29E-06
GLG2	Putative self-glucosylating initiator of glycogen synthesis; expression regulated upon white-opaque switch; hypha-induced; Spider biofilm induced	2.710847	0.007058
orf19.4600	Protein of unknown function; possible mitochondrial protein; Spider biofilm induced	2.708829	3.38E-05
orf19.1697	Ortholog(s) have role in cytoplasmic translation, poly(A)+ mRNA export from nucleus and cytoplasm localization	2.701269	4.1E-09

EXO1	Putative exodeoxyribonuclease; cell-cycle regulated periodic mRNA expression	2.699805	3.58E-08
orf19.4779	Putative transporter; slightly similar to the Sit1p siderophore transporter; Gcn4p-regulated; fungal-specific; induced by Mnl1p under weak acid stress	2.699724	2.34E-09
CFL4	C-terminus similar to ferric reductases; induced in low iron; Sfu1-repressed; ciclopirox olamine induced; colony morphology-related gene regulation by Ssn6; Hap43-repressed; Sef1-regulated	2.696293	0.00589
orf19.7450	Ortholog(s) have myosin II tail binding, protein-macromolecule adaptor activity, role in protein localization to cell division site, septin ring assembly and cellular bud neck, cellular bud neck septin ring localization	2.684798	3.68E-07
KRE1	Cell wall glycoprotein; beta glucan synthesis; increases glucan content in S. cerevisiae kre1, complements killer toxin sensitivity; caspofungin induced; Spider/rat catheter/flow model biofilm induced; Bcr1-repressed in RPMI a/a biofilms	2.682944	1.64E-05
SPA2	Protein involved in cell polarity, Spitzenkorper formation; required for mouse virulence; localizes to hyphal tip; cell-cycle regulated localization in yeast-form cells; functional domains conserved with S. cerevisiae ; Hap43p-induced gene	2.680866	7.27E-09
orf19.4792	Protein with a regulator of G-protein signaling domain; Plc1-regulated; Spider biofilm induced; rat catheter biofilm repressed	2.671109	0.002387
orf19.7067	Ortholog(s) have RNA polymerase II C-terminal domain phosphoserine binding, RNA polymerase II complex binding, transcription coregulator activity, triplex DNA binding activity	2.664869	2.67E-09
PET127	Protein with a predicted role in 5'-end processing of mitochondrial RNAs; ortholog of S. cerevisiae Pet127; Hap43-induced; rat catheter and Spider biofilm induced	2.657785	3.08E-07
SSQ1	Protein involved in intracellular sequestering of iron ion and mitochondrial iron-sulfur cluster assembly; repression leads to defects in respiratory growth, activation of autophagy, attenuated virulence	2.65693	1.31E-06
orf19.2386	Ortholog(s) have role in endonucleolytic cleavage in 5'-ETS of tricistronic rRNA transcript	2.656038	8.02E-09
AFT2	Putative Aft domain transcription factor; role in regulation of iron metabolism, oxidative stress, adhesion, hyphal growth, colony morphology, virulence;	2.653349	0.000105

	complements S. cerevisiae aft1 mutation; Spider biofilm induced		
CRZ1	Calcineurin-regulated C2H2 transcription factor; role in maintenance of membrane integrity, azole tolerance; not required for mouse virulence; repressed by low iron; regulates Ca++ influx during alkaline pH response; Spider biofilm induced	2.6510 04	4.99E-08
orf19.347 3	Ortholog(s) have enzyme activator activity, histone acetyltransferase activity, structural molecule activity	2.6481 09	1.77E-05
MAK21	Putative 66S pre-ribosomal particle subunit; mutation confers hypersensitivity to tubercidin (7-deazaadenosine)	2.6471 41	5.6E-09
DUR35	Putative urea transporter	2.6399 52	0.0124 23
FIP1	Puttaive pre-mRNA polyadenylation factor; heterozygous null mutant exhibits hypersensitivity to parnafungin and cordycepin in the C. albicans fitness test	2.6379 38	1.87E-12
IMP2	Predicted subunit of the mitochondrial inner membrane peptidase complex involved in protein targeting to mitochondria	2.6375 73	5.1E-11
NOG1	Putative GTPase; mutation confers hypersensitivity to 5-fluorocytosine (5-FC), 5-fluorouracil (5-FU), and tubercidin (7-deazaadenosine); repressed by prostaglandins; Hap43-induced	2.6356 9	1.89E-11
OYE32	NAD(P)H oxidoreductase family protein; induced by nitric oxide, amphotericin B, oxidative stress via Cap1; upregulation associated with MDR1 overexpression or benomyl treatment; macrophage-downregulated protein; Spider biofilm induced	2.6355 08	2.52E-12
JIP5	Ortholog of S. cerevisiae Jip5; predicted role in biogenesis of the large ribosomal subunit; repressed in core stress response; Hap43-induced gene	2.6327 16	9.66E-07
orf19.760 1	Putative mitochondrial 2' O-ribose methyltransferase; predicted role in methylation of U(2791) in 21S rRNA; Spider biofilm induced	2.6313 91	0.0016 16
orf19.356 3	**Ortholog of *C. dubliniensis CD36* : Cd36_19810, *C. parapsilosis CDC317* : CPAR2_206450, *C. auris B8441* : B9J08_005014 and *Candida tenuis NRRL Y-1498* : CANTEDRAFT_113905**	2.6254 9	0.0004 76
MNN46	Possible mannosyltransferase; Spider biofilm induced	2.6242 64	0.0035 71
BRF1	Component of the general transcription factor for RNA polymerase III (TFIIIB); possibly an essential gene, disruptants not obtained by UAU1 method	2.6221 61	2.62E-05

orf19.1684	Ortholog of C. dubliniensis CD36 : Cd36_81500, C. parapsilosis CDC317 : CPAR2_503620, C. auris B8441 : B9J08_001004 and Candida tenuis NRRL Y-1498 : CANTEDRAFT_93693 (5)	2.620882	0.00024
HAP42	Predicted transcription factor; possibly an essential gene, disruptants not obtained by UAU1 method	2.620834	3.29E-05
MPH1	Protein similar to S. cerevisiae Mph1p, which is a DNA helicase involved in DNA repair; induced under hydroxyurea treatment	2.616259	0.000268
CNS1	Putative co-chaperone; Hap43p-induced gene; mutation confers hypersensitivity to radicicol	2.612964	3.15E-09
SRP101	Signal recognition particle (SRP) receptor alpha subunit; involved in SRP-dependent protein targeting; rat catheter biofilm repressed	2.612803	6.45E-10
orf19.4133	Protein wth a predicted role in transcription from RNA polymerase II promoters; Spider biofilm induced	2.612692	0.000131
DRS1	Putative nucleolar DEAD-box protein; Hap43-induced; mutation confers hypersensitivity to 5-fluorouracil (5-FU), tubercidin (7-deazaadenosine); Tbf1-induced; repressed in core stress response	2.612647	1.84E-08
orf19.6712	**Ortholog of *S. cerevisiae* : SGM1, *C. glabrata CBS138* : CAGL0M00462g, *C. dubliniensis CD36* : Cd36_87610, *C. parapsilosis CDC317* : CPAR2_808090 and *C. auris B8441* : B9J08_004134**	2.611954	5.53E-07
orf19.3301	Putative ubiquitin ligase complex component; induced by heavy metal (cadmium) stress; Hog1-induced; transcript induced by Mnl1p under weak acid stress; flow model biofilm induced; Spider biofilm induced	2.608578	0.000166
orf19.7223	Ortholog(s) have protein-lysine N-methyltransferase activity, role in peptidyl-lysine monomethylation and cytosol, nucleus localization (5)	2.605218	1.42E-05
orf19.2612	C2H2 zinc finger transcription factor; expression reduced in ssr1 null mutant; flow model biofilm induced	2.602703	4.41E-06
SOF1	Putative protein with a predicted role in 40S ribosomal subunit biogenesis; rat catheter biofilm induced	2.602059	4.65E-10
orf19.4706	**Protein of unknown function; induced in cyr1 or ras1 mutant; induced by fluconazole, by alpha pheromone in SpiderM medium and during oralpharyngeal candidasis; Spider biofilm induced**	2.601969	2.09E-12
HST3	**Histone H3K56 deacetylase; reduced copy number increases opaque cell formation; repressed by MMS, hydroxyurea and high-levels of hydrogen peroxide; Hap43p-induced; ectopic expression blocks genotoxin-induced switching; nicotinamide target**	2.597826	6.63E-06

SWC4	Subunit of the NuA4 histone acetyltransferase complex	2.597402	1.5E-07
MET4	Putative transcription coactivator; predicted role in sulfur amino acid metabolism; required for yeast cell adherence to silicone substrate; Spider biofilm induced	2.595184	1.9E-06
UBP12	Ubiquitin-specific protease; cleaves ubiquitin from ubiquitinated proteins; Spider biofilm induced	2.594907	8.2E-07
CHL4	Protein described as having role in chromosome segregation; RNA abundance regulated by tyrosol and cell density	2.593896	0.006301
CTF1	Putative zinc-finger transcription factor, similar to A. nidulans FarA and FarB; activates genes required for fatty acid degradation; induced by oleate; null mutant displays carbon source utilization defects and slightly reduced virulence	2.591626	8.45E-05
orf19.1267	Ortholog of ***S. cerevisiae*** **: CAJ1,** ***C. glabrata CBS138*** **: CAGL0J02750g,** ***C. dubliniensis CD36*** **: Cd36_45380,** ***C. parapsilosis CDC317*** **: CPAR2_500650 and** ***C. auris B8441*** **: B9J08_005169**	2.588962	3.92E-08
orf19.4278	Ortholog(s) have chromatin binding, methylated histone binding activity and role in negative regulation of transcription by RNA polymerase II	2.581218	1.25E-05
orf19.267	Protein required for normal filamentous growth; mRNA binds She3	2.580858	3.69E-07
orf19.1106	Protein with Mob2p-dependent hyphal regulation; fluconazole-induced	2.580763	0.000941
orf19.501	Ortholog(s) have rRNA (cytosine-C5-)-methyltransferase activity	2.577508	2.51E-11
orf19.427	Ortholog(s) have telomeric DNA binding activity (5)	2.576029	6.22E-06
RCT1	Fluconazole-induced protein; Ras1, Cyr1 repressed and Efg1 induced; regulated by Nrg1, Tup1, Tbf1, Ssn6; induced in oralpharyngeal candidasis; rat catheter biofilm repressed	2.573171	1.33E-09
orf19.6896	Predicted ORF overlapping the Major Repeat Sequence on chromosome 7; member of a family encoded by FGR6-related genes in the RB2 repeat sequence	2.572729	2.19E-06
RFX1	Transcription factor; involved in DNA damage responses, morphogenesis, and virulence; Spider biofilm induced	2.569422	0.001947
orf19.2812	Protein of unknown function; Spider biofilm induced	2.567685	6.56E-05
orf19.6586	Protein of unknown function; transcript induced by benomyl or in azole-resistant strain overexpressing MDR1; Ssn6 colony morphology-related regulation;	2.566544	5.9E-06

	induced by NO; Hap43-repressed; rat catheter and flow model biofilm induced		
orf19.3999	Ortholog of ***S. cerevisiae*** **: YMR160W,** ***C. glabrata CBS138*** **: CAGL0M09493g,** ***C. dubliniensis CD36*** **: Cd36_54670,** ***C. parapsilosis CDC317*** **: CPAR2_502560 and** ***C. auris B8441*** **: B9J08_004333**	2.566427	0.000845
MSS11	Transcription factor; activator that binds to Flo8 via a LisH motif to cooperatively activate transcription of hypha-specific genes; required for hyphal growth	2.564132	0.000205
orf19.5275	Ortholog(s) have ATPase activator activity, protein-macromolecule adaptor activity and role in late endosome to vacuole transport, late endosome to vacuole transport via multivesicular body sorting pathway (5)	2.558013	2.71E-05
PLC1	Ortholog(s) have chaperone binding, unfolded protein binding activity and role in chaperone-mediated protein complex assembly, protein folding, protein import into mitochondrial intermembrane space, protein refolding	2.557241	1.3E-08
orf19.2459	Protein of unknown function; mRNA binds to She3; Hap43 repressed gene; Spider biofilm induced	2.556142	5.88E-05
orf19.1959	**Ortholog of** ***S. cerevisiae*** **: OTU2,** ***C. dubliniensis CD36*** **: Cd36_51210,** ***C. parapsilosis CDC317*** **: CPAR2_303850,** ***C. auris B8441*** **: B9J08_000423 and** ***Candida tenuis NRRL Y-1498*** **: CANTEDRAFT_114159**	2.553552	3.75E-08
orf19.3470	Putative flavodoxin; similar to S. cerevisiae Tyw1, an iron-sulfur protein required for synthesis of wybutosine modified tRNA; predicted Kex2p substrate; Spider biofilm induced	2.552921	3.96E-07
SOD5	Cu-containing superoxide dismutase; protects against oxidative stress; induced by neutrophils, hyphal growth, caspofungin, osmotic/oxidative stress; oralpharyngeal candidiasis induced; rat catheter and Spider biofilm induced	2.550031	8.79E-07
orf19.2604	S. pombe ortholog SPAC2C4.06c is a predicted tRNA (cytosine-5-)-methyltransferase; Spider biofilm induced	2.543641	0.00015
orf19.3726	Ortholog of ***C. dubliniensis CD36*** **: Cd36_65640 and** ***Candida albicans WO-1*** **: CAWG_0158**	2.542616	0.00581
orf19.520	Ortholog of ***C. dubliniensis CD36*** **: Cd36_29660,** ***C. parapsilosis CDC317*** **: CPAR2_205200,** ***C. auris B8441*** **: B9J08_005479 and** ***Candida tenuis NRRL Y-1498*** **: CANTEDRAFT_96118**	2.530643	0.000193
orf19.4628	Putative cleavage and polyadenylation factor; heterozygous null mutant exhibits hypersensitivity to	2.529152	7.47E-06

	parnafungin and cordycepin in the C. albicans fitness test; possibly an essential gene, disruptants not obtained by UAU1 method		
PRP24	**Ortholog(s) have U6 snRNA binding, snRNA binding activity, role in spliceosomal complex assembly, spliceosomal tri-snRNP complex assembly and U6 snRNP localization**	2.527819	0.000116
AIP5	Protein that stimulates actin assembly; interacts with polarisome components Bni1p and Bud6p; Hap43-repressed; induced by prostaglandins	2.522415	7.2E-09
orf19.536	Ortholog(s) have chromatin binding, molecular adaptor activity, ubiquitin binding activity and role in chromatin organization, histone acetylation, transcription by RNA polymerase II	2.522142	3.69E-07
UTP9	Small-subunit processome protein; Ssr1-induced; repressed by prostaglandins; physically interacts with TAP-tagged Nop1	2.516723	3.56E-10
ENT2	Epsin, involved in endocytosis; mutants are defective in hyphal growth and virulence	2.516537	9.51E-06
orf19.5431	Protein of unknown function; Hap43-repressed; Spider biofilm induced	2.51373	4.64E-06
RPN4	C2H2 transcription factor; putative regulator of proteasome genes; DNA recognition sequence (GAAGGCAAAA) enriched in regions upstream of proteasome genes; induced in core stress response; Hap43-induced; Spider biofilm induced	2.499891	3.62E-09
CBK1	Ser/Thr kinase of cell wall integrity pathway; mutants show abnormal morphology and aggregation; Mob2p associated; required for wild-type hyphal growth and transcriptional regulation of cell-wall-associated genes	2.497947	2.01E-06
Putative nucleolar complex protein; Hap43-induced; transposon mutation affects filamentous growth; mutation confers hypersensitivity to 5-fluorouracil (5-FU), tubercidin (7-deazaadenosine); repressed in core stress response		2.493404	2.73E-08
DBP6	Ortholog(s) have role in maturation of 5.8S rRNA from tricistronic rRNA transcript (SSU-rRNA, 5.8S rRNA, LSU-rRNA), maturation of LSU-rRNA from tricistronic rRNA transcript (SSU-rRNA, 5.8S rRNA, LSU-rRNA)	2.490944	2.93E-06
TOS4	Putative fork-head transcription factor; rat catheter and Spider biofilm repressed	2.489162	2.87E-07
IFU2	Ortholog(s) have ribosomal large subunit binding, tRNA binding activity and role in peptide biosynthetic process, rescue of stalled ribosome, ribosome-associated ubiquitin-dependent protein catabolic process	2.487373	5.48E-06

NAM9	Putative mitochondrial ribosomal component of the small subunit; possibly an essential gene, disruptants not obtained by UAU1 method; Spider biofilm repressed	2.486017	3.93E-11
orf19.1383	Protein of unknown function; induced by alpha pheromone in SpiderM medium (5)	2.48531	0.006272
orf19.5126	Putative adhesin-like protein	2.480984	1.74E-09
ERV1	Predicted component of the mitochondrial intermembrane space (IMS), involved in protein import into mitochondrial intermembrane space	2.475363	7.99E-07
orf19.537	**Ortholog of *C. dubliniensis CD36* : Cd36_29810, *C. parapsilosis CDC317* : CPAR2_206030, *C. auris B8441* : B9J08_002565 and *Candida tenuis NRRL Y-1498* : CANTEDRAFT_102086**	2.474456	0.002695
orf19.2213	Ortholog(s) have role in negative regulation of telomere maintenance, positive regulation of helicase activity and chromosome, telomeric region, site of double-strand break localization (5)	2.472334	0.003795
orf19.871	Ortholog(s) have GTPase activator activity, role in positive regulation of GTPase activity, positive regulation of TORC1 signaling and Lst4-Lst7 complex, cytoplasm, vacuolar membrane localization	2.468996	4.62E-05
GLN3	GATA transcription factor, involved in regulation of nitrogen starvation-induced filamentous growth; regulates transcription of Mep2 ammonium permease; regulated by Gcn2 and Gcn4; mRNA binds She3; Spider biofilm induced	2.468325	8.82E-05
CCT3	**Putative cytosolic chaperonin Cct ring complex subunit; mutation confers hypersensitivity to cytochalasin D**	2.468207	9.55E-12
HYS2	Putative DNA polymerase III (delta) subunit with a predicted role in DNA replication and DNA repair; cell-cycle regulated periodic mRNA expression	2.464244	5.07E-06
orf19.3684	Putative oxidoreductase; Spider biofilm induced	2.463694	0.000716
CTR1	Copper transporter; transcribed in low copper; induced Mac1, Tye7, macrophage interaction, alkaline pH via Rim101; 17-beta-estradiol repressed; complements S. cerevisiae ctr1 ctr3 copper transport mutant; flow model/Spider biofilm induced	2.463192	6.47E-11
HHT2	Putative histone H3; farnesol regulated; Hap43-induced; rat catheter and Spider biofilm repressed	2.462968	1.98E-13
PGA27	Putative GPI-anchored protein	2.461001	0.007013

GUT1	Putative glycerol kinase; downregulated upon adherence to polystyrene; greater mRNA abundance observed in a cyr1 homozygous null mutant than in wild type	2.458481	2.42E-05
orf19.2387	Putative tRNA-Pro synthetase; genes encoding ribosomal subunits, translation factors, and tRNA synthetases are downregulated upon phagocytosis by murine macrophage	2.457404	4.87E-09
orf19.6888	Zn(II)2Cys6 domain transcription factor; regulated by Mig1 and Tup1; rat catheter and Spider biofilm induced	2.455155	0.001395
orf19.7159	Putative protein of unknown function; Hap43p-repressed gene; ortholog of ***S. cerevisiae*** **YMR185W**	2.453424	0.004033
RSM7	Ortholog(s) have structural constituent of ribosome activity and mitochondrial small ribosomal subunit localization	2.45261	6.68E-12
PEL1	Predicted enzyme of mitochondrial phospholipid biosynthesis; rat catheter and flow model biofilm induced	2.450033	1.49E-05
orf19.2072	Ortholog(s) have (R)-carnitine transmembrane transporter activity, choline transmembrane transporter activity, ethanolamine transmembrane transporter activity (5)	2.448358	0.000332
orf19.2547	Has domain(s) with predicted RNA binding, ribonuclease activity	2.447689	0.000709
orf19.1666	Ortholog of Dig2, a MAP kinase-responsive inhibitor of Ste12; regulates mating-specific genes and invasive growth in S. cerevisiae; induced by alpha pheromone in SpiderM medium	2.447359	4.88E-06
ECM32	**Ortholog(s) have DNA helicase activity, role in regulation of translational termination and cytoplasmic stress granule, polysome localization**	2.44691	1.99E-05
SNM1	Protein similar to RNase MRP RNA binding protein; ciclopirox olamine induced; regulated by Sef1, Sfu1, and Hap43; Spider biofilm induced	2.444196	0.000025
SLK19	Alkaline-induced protein of plasma membrane; affects cell aggregation, cell wall; similar to S. cerevisiae Slk19p (a kinetochore protein with roles in mitosis, meiosis); required for wild-type virulence in mouse; macrophage-downregulated	2.444114	8.82E-08
orf19.6742	Ortholog(s) have RNA polymerase II CTD heptapeptide repeat phosphatase activity, protein serine/threonine phosphatase activity	2.443669	5.97E-05
CDC11	Septin; cell and hyphal morphology, agar-invasive growth, full virulence and kidney tissue invasion in mouse, but not kidney colonization, immunogenicity;	2.44078	1.42E-10

	hyphal and cell-cycle-regulated phosphorylation; rat catheter biofilm repressed		
orf19.3826	Predicted transmembrane protein with a role in cell wall polymer composition; Plc1-regulated; Spider biofilm induced	2.438667	8.78E-07
orf19.3163	Ortholog of ***C. dubliniensis CD36*** **: Cd36_51910,** ***C. parapsilosis CDC317*** **: CPAR2_101650,** ***C. auris B8441*** **: B9J08_001942 and** ***Candida tenuis NRRL Y-1498*** **: CANTEDRAFT_94797**	2.438271	2.58E-07
orf19.3306	Ortholog(s) have four-way junction DNA binding activity and role in homologous chromosome pairing at meiosis, meiotic recombination checkpoint signaling, synaptonemal complex assembly (5)	2.437547	0.005636
MNN45	Mannosyltransferase; transcript upregulated in Ssk1 response regulator mutant or in nik1 null mutant, but not in chk1 or sln1 null mutants; pheromone induced; Spider biofilm induced	2.436252	9.38E-06
orf19.2610	Putative tRNA binding protein; intron-containing gene; Spider biofilm induced	2.433726	8.03E-08
RVB2	Putative trancription modulator; RuvB-like protein family member; heterozygous null mutant displays sensitivity to virgineone	2.43199	1.18E-10
RTA2	Flippase involved in sphingolipid long chain base release; mediates calcineurin-dependent ER stress response and resistance to azoles; Plc1p, Ca2+, calcineurin-regulated	2.424483	1.88E-13
KAR3	Kinesin-like microtubule motor protein; required for nuclear fusion during mating; C-terminal motor domain; mutants are viable; null has high-frequency white-opaque switching phenotype	2.423324	0.001659
LIG1	tRNA ligase; functional homolog of S. cerevisiae Trl1	2.418719	8.48E-06
orf19.841	Ortholog(s) have SNARE binding activity, role in Golgi vesicle docking, Golgi vesicle transport and Golgi membrane localization	2.418265	1.98E-05
MRP2	Protein similar to S. cerevisiae Mrp2p, which is a component of the small subunit of the mitochondrial ribosome; transposon mutation affects filamentous growth	2.416408	4.19E-10
PRP3	Predicted splicing factor, component of the U4/U6-U5 snRNP complex; Hap43-induced gene; rat catheter biofilm induced	2.41586	0.001721
SWI6	Putative component of the MBF and SBF transcription complexes involved in G1/S cell-cycle progression; periodic mRNA expression, peak at cell-cycle G1/S phase	2.415327	0.000225

orf19.3461	Protein of unknown function; oxidative stress-induced via Cap1; induced by alpha pheromone in SpiderM medium	2.414245	3.09E-06
NGG1	Ortholog(s) have histone acetyltransferase activity and role in chromatin organization, histone acetylation, regulation of transcription by RNA polymerase II	2.413351	2.55E-05
SKY1	Predicted protein serine/threonine kinase; Spider biofilm induced	2.41086	4.59E-06
KIS1	Snf1p complex scaffold protein; similar to S. cerevisiae Gal83p and Sip2p with regions of similarity to Sip1p (ASC and KIS domain); interacts with Snf4p; mutants are hypersensitive to caspofungin and hydrogen peroxide; Hap43p-repressed gene	2.406204	3.96E-07
CYC3	Mitochondrial cytochrome c heme lyase; antigenic cell-wall protein; filament induced; induced on polystyrene adherence, interaction with macrophage; N-glycosylation, 2 heme-binding motifs; rat catheter, Spider biofilm induced	2.401036	1.88E-09
NSA1	Putative 66S pre-ribosomal particles conmponent; Hap43-induced; repressed by prostaglandins	2.400916	5.6E-07
orf19.3751	Putative serine/threonine protein kinase; possibly an essential gene, disruptants not obtained by UAU1 method	2.40075	3.62E-06
orf19.1766	Secreted protein; fluconazole-induced	2.399931	0.012476
orf19.3135	Ortholog(s) have protein-containing complex binding, protein-macromolecule adaptor activity	2.398484	1.29E-05
STP4	C2H2 transcription factor; induced in core caspofungin response; colony morphology-related gene regulation by Ssn6; induced by 17-beta-estradiol, ethynyl estradiol; rat catheter and Spider biofilm induced	2.393376	2.79E-08
orf19.4092	Trimeric GatFAB AmidoTransferase(AdT) complex subunit; involved in the formation of Q-tRNAQ; repressed by fluphenazine treatment or in an azole-resistant strain that overexpresses CDR1; Spider biofilm induced	2.392506	2.07E-09
TFG1	Protein similar to S. cerevisiae Tfg1p, which is part of transcription factor TFIIF; transposon mutation affects filamentous growth; possibly an essential gene, disruptants not obtained by UAU1 method	2.388558	7.97E-09
UTP22	Putative U3 snoRNP protein; Ssr1-induced; repressed by prostaglandins; heterozygous null mutant is resistant to parnafungin	2.382333	3.01E-07

RAX1	Protein with a predicted role in bud site selection; hypha-induced expression; Spider biofilm induced	2.381163	1.5E-07
orf19.4441	Ortholog(s) have DNA replication origin binding, double-stranded DNA binding, single-stranded DNA binding activity	2.38098	4.47E-07
orf19.7291	Ortholog(s) have tRNA (adenine-N1-)-methyltransferase activity, role in tRNA methylation and nucleus, tRNA (m1A) methyltransferase complex localization	2.380378	0.000283
orf19.5070	**Similar to cell-wall mannoproteins; induced in low iron; induced in cyr1 homozygous null; regulated by osmotic and oxidative stress via Hog1; Spider biofilm induced**	2.380344	6.53E-09
RET2	Delta subunit of the coatomer complex (COPI); coats Golgi-derived transport vesicles; involved in retrograde transport between Golgi and ER; interacts with Crk1 in the two-hybrid system; Spider biofilm repressed	2.380177	1.36E-08
SLM2	Ortholog(s) have role in TOR signaling, actin cytoskeleton organization, actin filament bundle assembly, eisosome assembly, endosomal transport and establishment or maintenance of actin cytoskeleton polarity	2.378714	2.75E-06
RRP15	Putative nucleolar protein; constituent of pre-60S ribosomal particles; Hap43-induced; repressed by prostaglandins	2.377154	2.25E-09
orf19.5425	Ortholog(s) have 3'-tRNA processing endoribonuclease activity	2.372876	1.62E-05
RPL29	Ribosomal protein L29; induced upon germ tube formation; colony morphology-related gene regulation by Ssn6; intron in 5'-UTR; Spider biofilm repressed	2.372646	1.07E-07
CWC25	Putative splicing factor required for the first step of pre-mRNA splicing; Spider biofilm induced	2.371773	8.99E-05
YTA7	Ortholog(s) have ATPase, chromatin binding, histone binding activity	2.371697	3.39E-05
ISW2	Ortholog of S. cerevisiae Isw2; an ATPase involved in chromatin remodeling; required for chlamydospore formation; Hap43-induced gene; repressed by high-level peroxide stress	2.371283	0.000474
orf19.4110	Ortholog of C. dubliniensis CD36 : Cd36_20490, C. parapsilosis CDC317 : CPAR2_104180, C. auris B8441 : B9J08_001215 and Candida tenuis NRRL Y-1498 : CANTEDRAFT_116074 (5)	2.370224	0.00172
PKH2	Putative serine/threonine protein kinase; predicted role in sphingolipid-mediated signaling pathway that	2.368782	3.81E-05

	controls endocytosis; mRNA binds She3 and is localized to hyphal tips		
orf19.6119	**Protein of unknown function; Spider biofilm repressed**	2.368604	7.47E-05
SPT8	Ortholog(s) have TBP-class protein binding, transcription coregulator activity	2.367447	0.000665
NOP4	Putative nucleolar protein; Hap43-induced; mutation confers hypersensitivity to 5-fluorocytosine (5-FC), 5-fluorouracil (5-FU), and tubercidin (7-deazaadenosine); represses in core stress response	2.36664	9.44E-06
RML2	Ortholog(s) have structural constituent of ribosome activity, role in mitochondrial translation and mitochondrial large ribosomal subunit localization	2.365458	3.26E-10
DBF4	**Regulatory subunit of Cdc7p-Dbf4p protein kinase complex that acts as negative regulator of hyphal development; cell-cycle regulated periodic mRNA expression; S. cerevisiae ortholog is not cell-cycle regulated**	2.36483	2.53E-09
SMC6	**Putative structural maintenance of chromosomes (SMC) protein; Hap43-induced; cell-cycle regulated periodic mRNA expression; S. cerevisiae ortholog not cell-cycle regulated; Spider biofilm induced**	2.364752	0.000117
orf19.53	Ortholog(s) have role in mRNA cis splicing, via spliceosome and U2-type prespliceosome, U2AF complex, commitment complex localization	2.364021	0.000347
REG1	Putative protein phosphatase regulatory subunit; Hap43-repressed gene; macrophage/pseudohyphal-induced; possibly regulated upon hyphal formation; flow model biofilm induced	2.359933	2.68E-06
CFT2	Putative mRNA cleavage and polyadenlylation factor; heterozygous null mutant exhibits hypersensitivity to parnafungin and cordycepin in the C. albicans fitness test	2.359509	3.18E-07
DHR2	DEAH-box ATP-dependent RNA helicase, required for 18S rRNA synthesis; rat catheter biofilm induced	2.357379	0.00012
SAP10	Secreted aspartyl protease; roles in adhesion, virulence (RHE model), cell surface integrity; distinct specificity from Sap9; at cell membrane and wall; GPI-anchored; induced in low iron; Tbf1-activated; Spider biofilm induced	2.35709	2.29E-06
RAT1	5'-->3' exoribonuclease; similar to S. cerevisiae nuclear exoribonuclease Rat1p; suppresses S. cerevisiae kem1 mutant slow growth, mating defect, and haploid invasive growth defect	2.357067	1.12E-05

SPT7	Putative SAGA transcriptional regulatory complex subunit; mutation confers hypersensitivity to toxic ergosterol analog, and to amphotericin B	2.356338	0.000228
VHR1	Transcriptional activator of genes involved in biotin metabolism; required for survival and proliferation in macrophages; expression upregulated during growth in the mouse cecum; Spider biofilm induced	2.354201	0.002413
orf19.952	Has domain(s) with predicted DNA binding activity	2.353016	0.000591
orf19.2391	Putative protein of unknown function; Hap43p-repressed gene; ***S. cerevisiae*** **ortholog YKR023W localizes to mitochondria**	2.352771	0.002718
orf19.7011	Ortholog(s) have role in maturation of SSU-rRNA from tricistronic rRNA transcript (SSU-rRNA, 5.8S rRNA, LSU-rRNA) and nucleus, preribosome, small subunit precursor localization	2.351975	1.12E-06
FET31	Putative multicopper oxidase; ketoconazole/caspofungin/amphotericin B repressed; Sef1/Sfu1/Hap43 regulated; reports differ if functional homolog of ScFet3; rat catheter and Spider biofilm induced	2.350712	1.46E-05
orf19.6694	Ortholog of ***C. dubliniensis CD36*** **: Cd36_73320,** ***C. parapsilosis CDC317*** **: CPAR2_703860,** ***C. auris B8441*** **: B9J08_002111 and** ***Candida tenuis NRRL Y-1498*** **: CANTEDRAFT_114480**	2.347394	0.006437
orf19.6506	Ortholog(s) have role in histone deacetylation, negative regulation of antisense RNA transcription and positive regulation of transcription by RNA polymerase II	2.346378	0.000241
orf19.3449	Ortholog(s) have ubiquitin protein ligase activity and role in histone catabolic process, histone ubiquitination, mRNA destabilization, negative regulation of gene expression	2.346161	6.14E-05
orf19.6855	**Ortholog of** ***C. dubliniensis CD36*** **: Cd36_04370,** ***C. parapsilosis CDC317*** **: CPAR2_105410,** ***C. auris B8441*** **: B9J08_000021 and** ***Candida tenuis NRRL Y-1498*** **: CANTEDRAFT_114891**	2.345901	3.49E-07
RCK2	Predicted MAP kinase-activated protein kinase, similar to S. cerevisiae serine/threonine protein kinase Rck2p; induced by osmotic stress via Hog1p; macrophage/pseudohyphal-repressed; mutants are sensitive to rapamycin	2.343576	1.93E-07
orf19.4835	Ortholog(s) have role in endonucleolytic cleavage in 5'-ETS of tricistronic rRNA transcript	2.342366	7.49E-08

orf19.3431	Ortholog(s) have 3'-5' exonuclease activity, DNA-directed DNA polymerase activity, role in mitochondrial DNA catabolic process, mitochondrial DNA replication, mitochondrial genome maintenance and mitochondrion localization	2.342056	0.002151
RAD52	Required for homologous DNA recombination, repair of UV- or MMS-damaged DNA, telomere length, UV-induced LOH; constitutive expression, MMS-induced; weakly complements S. cerevisiae rad52 mutant; slow growth, increased white-to-opaque switch	2.340971	0.000467
KAR4	**Transcription factor; required for gene regulation in response to pheromones; ortholog of S. cerevisiae Kar4; role in karyogamy; opaque-specific, a-specific; induced by alpha factor**	2.338772	4.59E-06
orf19.4204	Ortholog(s) have structural constituent of ribosome activity and mitochondrial small ribosomal subunit localization	2.33716	4.33E-07
BOI2	Putative SH3-domain-containing protein; mutation confers hypersensitivity to toxic ergosterol analog; Spider biofilm induced	2.335924	6.48E-07
AHC1	Ortholog(s) have histone acetyltransferase activity, role in histone acetylation, replication-born double-strand break repair via sister chromatid exchange and Ada2/Gcn5/Ada3 transcription activator complex localization	2.334382	2.73E-06
STI1	Protein that interacts with Cdc37 and Crk1 in two-hybrid; may be involved in Cdc37 chaperone activity; soluble protein in hyphae; protein in exponential and stationary phase yeast cultures; YNB biofilm induced; Spider biofilm induced	2.330194	9.73E-09
MSW1	Protein similar to S. cerevisiae Msw1p, which is mitochondrial tryptophanyl-tRNA synthetase; Hap43p-repressed gene; likely to be essential for growth, based on an insertional mutagenesis strategy	2.329989	4.69E-07
RAD2	Ortholog of S. cerevisiae Rad2, a nucleotide excision repair nuclease; null mutant is extremely sensitive to UV irradiation; transcript induced by interaction with macrophages	2.329006	0.000806
LTV1	**Putative GSE complex component; repressed by prostaglandins**	2.325284	6.68E-07
orf19.3275	**Ortholog of *C. dubliniensis CD36* : Cd36_25870, *C. parapsilosis CDC317* : CPAR2_804000, *C. auris B8441* : B9J08_001023 and *Candida tenuis NRRL Y-1498* : CANTEDRAFT_115661**	2.325048	0.001539

GUF1	Ortholog(s) have GTPase activity, mitochondrial ribosome binding activity, role in positive regulation of translation and mitochondrial matrix localization	2.323813	0.000109
AFG3	Similar to S. cerevisiae Afg3p, a subunit of the mitochondrial inner membrane m-AAA protease; likely to be essential for growth, based on an insertional mutagenesis strategy	2.322436	1.06E-06
PUS4	Putative pseudouridine synthase; transcript regulated by Nrg1, Mig1, and Tup1	2.321791	1.03E-07
KES1	Putative oxysterol binding protein family; probable peripheral membrane protein of the Golgi complex; flow model and Spider biofilm repressed	2.321142	6.81E-08
PGA11	Putative GPI-anchored protein	2.318448	0.017732
BUD14	Putative SH3-domain-containing protein; predicted role in bud-site selection; Spider biofilm induced	2.310577	0.000532
orf19.1873	Protein of unknown function; Hap43-repressed gene; repressed by nitric oxide	2.308927	2.41E-09
PGA13	GPI-anchored cell wall protein involved in cell wall synthesis; required for normal cell surface properties; induced in oralpharyngeal candidasis; Spider biofilm induced; Bcr1-repressed in RPMI a/a biofilms	2.308253	2.41E-08
NUP49	Nuclear pore protein	2.308248	1.15E-05
DEP1	Ortholog(s) have role in histone deacetylation, negative regulation of chromatin silencing at telomere and negative regulation of ribosomal DNA heterochromatin assembly	2.307429	0.000135
CZF1	Transcription factor; regulates white-opaque switch; hyphal growth regulator; expression in S. cerevisiae causes dominant-negative inhibition of pheromone response; required for yeast cell adherence to silicone; Spider biofilm induced	2.300999	0.004496
orf19.4030	Ortholog(s) have DNA primase activity, single-stranded DNA binding activity and role in DNA replication, DNA replication, synthesis of RNA primer	2.300761	5.17E-05
orf19.4097	Ortholog of C. dubliniensis CD36 : Cd36_23480, C. parapsilosis CDC317 : CPAR2_407050, C. auris B8441 : B9J08_003033 and Candida tenuis NRRL Y-1498 : CANTEDRAFT_116822 (5)	2.297811	0.005585
PCP1	Ortholog(s) have serine-type endopeptidase activity, role in regulation of mitochondrion organization, signal peptide processing and mitochondrial inner membrane localization	2.297272	3.27E-10

DIP2	Putative small ribonucleoprotein complex; Tn mutation affects filamentous growth; physically interacts with TAP-tagged Nop1; heterozygous null mutant exhibits resistance to parnafungin; Hap43-induced gene; Spider biofilm induced	2.296088	8.97E-06
orf19.1576	Ortholog(s) have nuclear localization sequence binding, ubiquitin binding activity and role in response to ethanol	2.294868	0.000247
orf19.5051	Has domain(s) with predicted protein heterodimerization activity	2.294002	0.004191
orf19.4246	Protein with similarity to S. cerevisiae Ykr070w; Tn mutation affects filamentation; Hog1-repressed; colony morphology-related gene regulation by Ssn6p; induced during cell wall regeneration; possibly essential	2.293841	2.17E-10
FGR15	Putative transcription factor with zinc finger DNA-binding motif; lacks an ortholog in S. cerevisiae; transposon mutation affects filamentous growth; caspofungin induced; mutation causes marginal increase in caspofungin sensitivity	2.290818	0.009416
PET112	**Ortholog(s) have glutaminyl-tRNA synthase (glutamine-hydrolyzing) activity, role in glutaminyl-tRNAGln biosynthesis via transamidation, mitochondrial translation and glutamyl-tRNA(Gln) amidotransferase complex, mitochondrion localization**	2.290603	0.000163
orf19.6681	Protein of unknown function; Spider biofilm induced	2.28933	0.000232
BRG1	Transcription factor; recruits Hda1 to hypha-specific promoters; Tn mutation affects filamentation; Hap43-repressed; Spider and flow model biofilm induced; required for Spider biofilm formation; Bcr1-repressed in RPMI a/a biofilms	2.289044	4.9E-07
SLU7	Protein similar to S. cerevisiae RNA splicing factor, Slu7; induced by alpha pheromone in SpiderM medium	2.288902	0.006423
orf19.254	Protein of unknown function; Hog1p-repressed; Spider biofilm induced	2.287892	0.000269
CWC2	Ortholog(s) have U6 snRNA binding activity and role in mRNA cis splicing, via spliceosome, mRNA splicing, via spliceosome	2.287549	0.00038
orf19.3170	**Ortholog(s) have role in RNA polymerase I assembly, RNA polymerase II core complex assembly, RNA polymerase III assembly, formation of translation preinitiation complex and cytoplasm localization**	2.286402	0.000118
RSC4	Component of the RSC chromatin remodeling complex	2.285671	3.72E-06

ESA1	Subunit of the NuA4 histone acetyltransferase complex; member of MYST family; Spider biofilm induced	2.283764	4.22E-05
FGR47	Protein lacking an ortholog in S. cerevisiae; transposon mutation affects filamentous growth	2.282333	0.002805
SFL2	Transcription factor involved in regulation of morphogenesis; regulates transcription in response to carbon dioxide levels; required for filamentous growth, for virulence in RHE model but not in mice; Spider biofilm induced	2.28167	0.000468
LHS1	Protein similar to S. cerevisiae Hsp70p; predicted Kex2p substrate; possibly essential, disruptants not obtained by UAU1 method; flow model biofilm repressed	2.280838	2.28E-05
TSC11	Protein similar to S. cerevisiae Tsc11p which is involved in sphingolipid biosynthesis; transposon mutation affects filamentous growth	2.280569	0.008302
MSS1	Ortholog(s) have role in mitochondrial tRNA wobble uridine modification and mitochondrial inner membrane localization	2.277866	0.00042
IFM1	Putative mitochondrial translation initiation factor; transcript regulated by Nrg1, Mig1, and Tup1	2.273268	0.000245
CRK1	Protein kinase of the Cdc2 subfamily involved in hyphal development, virulence; promotes hyphal development independently of Cph1 and Efg1; functionally complements pheromone hypersensitivity of S. cerevisiae sgv1 mutant; Hap43p-repressed	2.27162	0.001299
orf19.6268	Putative cohesin complex subunit; expression downregulated in an ssr1 null mutant (5)	2.270726	0.003133
MRPL40	Putative mitochondrial ribosomal protein; Spider biofilm repressed	2.265684	6.95E-09
orf19.2527	Putative protein of unknown function; Hap43-induced; required for normal biofilm growth; F-12/CO2 early biofilm induced	2.262442	1.44E-06
orf19.6049	**Ortholog(s) have protein kinase activator activity and role in chromosome segregation, protein phosphorylation, regulation of cytokinesis**	2.261339	0.00011
orf19.5168	Ortholog(s) have unfolded protein binding activity, role in maturation of SSU-rRNA, ribosomal small subunit biogenesis and cytoplasm localization	2.261132	0.00013
NPR2	Putative urea transporter; induced during infection of murine kidney, compared to growth in vitro; has murine homolog	2.260999	0.002522
orf19.3064.1	**Ortholog of *C. parapsilosis CDC317* : CPAR2_108915, *C. auris B8441* : B9J08_004667, *Candida tenuis NRRL Y-1498* :**	2.260841	0.005605

	**CANTEDRAFT_102387 and *Debaryomyces hansenii CBS767* : DEHA2B06776g**		
orf19.3932	Predicted RNA binding protein; stationary phase enriched; induced in core caspofungin response; induced by nitric oxide independent of Yhb1; repressed in ssr1 null; ketoconazole, hypoxia induced; Spider biofilm induced	2.259971	9.76E-08
MTO1	Putative mitochondrial protein; rat catheter biofilm induced	2.259705	0.008024
HSX11	UDP-glucose:ceramide glucosyltransferase (glucosylceramide synthase [GCS], EC 2.4.1.80); involved in glucosylceramide biosynthesis, which is important for virulence	2.255353	0.002695
orf19.2982	Ortholog(s) have SNARE binding, molecular adaptor activity, protein kinase activator activity	2.254241	0.004256
NAG4	Putative transporter; fungal-specific; similar to Nag3p and to S. cerevisiae Ypr156Cp and Ygr138Cp; required for wild-type mouse virulence and wild-type cycloheximide resistance; gene cluster encodes enzymes of GlcNAc catabolism	2.25341	0.009687
orf19.2846	Protein of unknown function; Hap43-repressed; induced in core caspofungin response; regulated by yeast-hypha switch; Spider biofilm repressed	2.251055	1.93E-05
orf19.4623	Protein of unknown function; Spider biofilm repressed	2.249882	0.002776
orf19.1830	Protein of unknown function; Hap43-induced; rat catheter and Spider biofilm induced	2.249486	0.000633
VPS27	Putative ESCRT-0 complex protein with a role in multivesicular body (MVB) trafficking	2.248352	7.84E-06
orf19.5987	Ortholog(s) have tRNA (guanine-N2-)-methyltransferase activity, role in tRNA methylation and cytoplasm, tRNA (m2G10) methyltransferase complex localization	2.246152	1.09E-05
orf19.4017	**Ortholog of *C. dubliniensis CD36* : Cd36_54840, *C. parapsilosis CDC317* : CPAR2_100270, *C. auris B8441* : B9J08_003974 and *Candida tenuis NRRL Y-1498* : cten_CGOB_00242**	2.243478	0.014154
IFH1	**Transcription factor; forms a heterodimer with Fhl11 that is tethered to promoters by Tbf1; positively regulates rRNA and ribosomal protein gene transcription; Spider biofilm induced**	2.242925	2.08E-05
DBP7	**Putative ATP-dependent DEAD-box RNA helicase; Hap43-induced; rat catheter biofilm induced**	2.240029	0.000017
SWE1	Putative protein kinase with a role in control of growth and morphogenesis, required for full virulence; mutant	2.239913	4.72E-06

	cells are small, rounded, and sometimes binucleate; not required for filamentous growth; mutant is hypersensitive to caspofungin		
BEM3	Putative GTPase-activating protein (GAP) for Rho-type GTPase Cdc42p; involved in cell signaling pathways that control cell polarity; similar to ***S. cerevisiae*** **Bem3p**	2.2372<br>8	0.0001<br>18
orf19.701<br>2	Ortholog(s) have structural constituent of ribosome activity and mitochondrial small ribosomal subunit localization	2.2340<br>01	7.38E-<br>09
orf19.640<br>8	Putative DnaJ-like heat shock/chaperone; Hap43-repressed; Spider and F-12/CO2 biofilm induced	2.2252<br>4	4.92E-<br>05
GDS1	Putative mitochondrial protein; Hap43-repressed gene; Spider biofilm induced	2.2245<br>87	9.34E-<br>05
TRM9	Putative tRNA methyltransferase; repressed during the mating process	2.2214<br>55	9.35E-<br>09
POP4	Ortholog of S. cerevisiae Pop4; a subunit of both RNase MRP and nuclear RNase P; filament induced; regulated by Nrg1, Tup1; likely essential, based on UAU1 strategy; rat catheter and Spider biofilm induced	2.2214<br>55	0.0006<br>44
orf19.684<br>0	Protein of unknown function; transcript detected in high-resolution tiling arrays; transcription induced by alpha pheromone in SpiderM medium; Spider and early-stage flow model biofilm induced (5, 6, 7, 8)	2.2178<br>46	1.98E-<br>07
ZCF29	Zn(II)2Cys6 transcription factor; mutants sensitive to caffeine, menadione, beauvericin;resistant to fenpropimorph; Hap43-repressed; mutants have decreased CFU in mouse kidneys and defects in filamentous growth; Spider biofilm induced	2.2175<br>33	3.86E-<br>05
orf19.568<br>8	Predicted ORF from Assembly 19; removed from Assembly 20; subsequently reinstated in Assembly 21 based on comparative genome analysis	2.2165<br>6	1.67E-<br>06
SEN1	Putative helicase; repressed by prostaglandins	2.2158<br>41	0.0015<br>43
orf19.502<br>0	Protein of unknown function; Hap43-induced; Spider biofilm induced	2.2154<br>81	8.28E-<br>05
SEF1	Zn2-Cys6 transcription factor; regulates iron uptake; negatively regulated by Sfu1p, positively regulated by Tbf1; promotes virulence in mice; mutants display decreased colonization of mouse kidneys; Spider biofilm induced	2.2127<br>2	0.0001<br>36
PGA56	Regulator of sorbose utilization; putative GPI-anchor; hypha and cell wall regeneration induced; colony morphology-related gene regulation by Ssn6; induced by Tbf1, Hap43; Spider biofilm induced	2.2116<br>54	3.52E-<br>08

PEX4	Putative peroxisomal ubiquitin conjugating enzyme; regulated by Sef1, Sfu1, and Hap43; rat catheter biofilm induced; Spider biofilm induced	2.211583	7.97E-05
SGO1	**Component of the spindle checkpoint; cell-cycle regulated periodic mRNA expression**	2.208744	0.005701
KIP4	S. cerevisiae Smy1 ortholog; Tn mutation affects filamentous growth; filament induced; has Mob2-dependent hyphal regulation; regulated by Nrg1, Tup1; oralpharyngeal candidasis induced; Spider biofilm induced; flow model biofilm repressed	2.208624	0.006969
NUP85	Ortholog of S. cerevisiae Nup85; a structural constituent of the nuclear pore; required for alkaline-induced hyphal morphogenesis and for SD or Spider media biofilm formation	2.207801	1.04E-06
orf19.6156	**Ortholog of *S. cerevisiae* : AIM11, *C. glabrata CBS138* : CAGL0I04928g, *C. dubliniensis CD36* : Cd36_80770, *C. parapsilosis CDC317* : CPAR2_102260 and *C. auris B8441* : B9J08_002841**	2.207235	1.81E-06
UTP8	**Essential nucleolar protein; involved in tRNA export from the nucleus and ribosomal small subunit biogenesis; physically interacts with TAP-tagged Nop1; Spider biofilm induced**	2.207178	1.51E-08
RFC1	Putative DNA replication factor C subunit; ortholog of S. cerviaie Rfc1; likely essential, based on an insertional mutagenesis strategy; rat catheter biofilm repressed	2.206981	1.03E-05
PGA52	GPI-anchored cell surface protein of unknown function; Hap43p-repressed gene; fluconazole-induced; possibly an essential gene, disruptants not obtained by UAU1 method	2.202354	1.16E-06
NAR1	Putative cytosolic iron-sulfur (FeS) protein assembly machinery protein; induced by nitric oxide; oxidative stress-induced via Cap1p	2.201162	1.83E-06
EST1	Telomerase subunit; allosteric activator of catalytic activity, but not required for catalytic activity; has TPR domain	2.198094	1.07E-07
orf19.1730	HMG-box protein; Spider biofilm repressed	2.194629	3.66E-05
orf19.6843	Ortholog(s) have role in CENP-A containing nucleosome assembly, chromatin maintenance and nucleoplasm localization	2.193294	4.17E-06
REX4	Putative RNA exonuclease; induced in a ssr1 null mutant	2.192567	0.000155
NCS2	Putative cytosolic thiouridylase subunit; Spider biofilm induced	2.191458	0.001561

orf19.4332	Ortholog of ***C. dubliniensis CD36*** **: Cd36_52800,** ***C. parapsilosis CDC317*** **: CPAR2_303110,** ***C. auris B8441*** **: B9J08_002492 and** ***Candida tenuis NRRL Y-1498*** **: CANTEDRAFT_92321**	2.188616	0.00347
orf19.7581	Protein with a predicted role in assembly of U2 snRNP into the spliceosome; Spider biofilm induced	2.187287	4.22E-05
orf19.445	Protein of unknown function; repressed by prostaglandins	2.186872	1.18E-06
orf19.813	**Protein of unknown function; mutants are viable; Hap43-induced gene; oxidative stress-induced via Cap1; rat catheter and Spider biofilm induced**	2.186749	2.98E-05
ISM1	Protein similar to isoleucyl-tRNA synthetase; isoleucyl-tRNA synthetase is the target of drugs including the cyclic beta-amino acid icofungipen/PLD-118/BAY-10-8888 and mupirocin	2.186385	0.000181
orf19.1642	Ortholog of S. cerevisiae Loc1, a nuclear protein involved in asymmetric localization of ASH1 mRNA in S. cerevisiae; Hap43-induced gene; Spider biofilm induced	2.186108	3.82E-08
orf19.6769	Has domain(s) with predicted intracellular anatomical structure localization	2.182377	0.001335
CAC2	**Component of the chromatin assembly factor I (CAF-1); involved in regulation of white-opaque switching frequency; macrophage-induced**	2.18067	0.000666
orf19.5698	**Putative mitochondrial ribosomal protein of the large subunit; transcript is upregulated in clinical isolates from HIV+ patients with oral candidiasis; Spider biofilm repressed**	2.18056	2.89E-08
CLB4	**B-type mitotic cyclin; nonessential; negative regulator of pseudohyphal growth; dispensible for mitotic exit, cytokinesis; Fkh2-represed; flow model biofilm repressed; farnesol-upregulated in biofilm; reduced total RNA in clb4 mutant**	2.180428	8.84E-05
UTP5	Putative U3 snoRNA-associated protein; Hap43p-induced gene; mutation confers resistance to 5-fluorocytosine (5-FC), 5-fluorouracil (5-FU), and tubercidin (7-deazaadenosine); physically interacts with TAP-tagged Nop1p	2.179764	2.84E-06
PST2	Flavodoxin-like protein involved in oxidative stress protection and virulence; putative NADH:quinone oxidoreductase; similar to 1,4-benzoquinone reductase; induced by benomyl, oxidative stress via Cap1; fungal-specific	2.178709	5.14E-06

MAK16	Putative constituent of 66S pre-ribosomal particles; Hap43-induced; repressed by prostaglandins; Spider biofilm induced	2.177118	2.43E-07
orf19.6675	**Protein of unknown function; from Assembly 19; removed from Assembly 20; restored based on comparative genome analysis; F-12/CO2 early biofilm induced**	2.174002	7.87E-05
orf19.4622	Ortholog(s) have role in cellular response to DNA damage stimulus, protein ubiquitination, telomere maintenance, transcription-coupled nucleotide-excision repair, ubiquitin-dependent protein catabolic process and nucleus localization	2.173345	6E-07
FGR6-1	Protein lacking an ortholog in S. cerevisiae; member of a family encoded by FGR6-related genes in the RB2 repeat sequence; transposon mutation affects filamentous growth	2.171466	0.009881
orf19.7460	Putative 2'-O-methyltransferase with a predicted role in tRNA modification; transcription is activated in the presence of elevated CO2	2.17073	0.00186
RFX2	Transcriptional repressor; regulator of filamentation, response to DNA damage, adhesion, virulence in murine mucosal, systemic infections; RFX domain; regulated by Nrg1, UV-induced; partially complements S. cerevisiae rfx1 mutant defects	2.169925	0.01916
HHF22	Putative histone H4; regulated by Efg1; flucytosine, fluconazole-induced; amphotericin B, caspofungin repressed; colony morphology-related gene regulation by Ssn6; Hap43-induced; rat catheter and Spider biofilm repressed	2.16962	2.7E-07
PSD2	Ortholog(s) have phosphatidylserine decarboxylase activity, role in phosphatidylcholine biosynthetic process, phosphatidylethanolamine biosynthetic process and endosome localization	2.168819	0.000058
FBP1	Fructose-1,6-bisphosphatase; key gluconeogenesis enzyme; regulated by Efg1, Ssn6; induced by phagocytosis; effects switch from glycolysis to gluconeogenesis in macrophage; rat flow model biofilm induced; overlaps orf19.6179	2.168475	1.23E-05
GRR1	F-box protein component of the SCF ubiquitin-ligase complex required for cell cycle progression; involved in negative control of pseudohyphal growth; regulates stability of Ume6p in response to CO2	2.168133	2.17E-05
RPN5	Putative COP9 signalosome component; macrophage/pseudohyphal-repressed	2.165378	6.3E-07

FAL1	eIF4A subfamily of DEAD-box ATP-dependent RNA helicases; predicted nucleolar protein required for maturation of 18S rRNA; Spider biofilm induced	2.162948	0.000777
orf19.1580	Protein of unknown function; Spider biofilm induced	2.160973	0.002606
orf19.7458	**Ortholog of *C. dubliniensis CD36* : Cd36_86660, *Candida tenuis NRRL Y-1498* : cten_CGOB_00028, *Candida tropicalis NEW ASSEMBLY*: CTRG1_05695 and *Candida tropicalis MYA-3404* : CTRG_05695**	2.15675	0.000706
BUL1	Protein similar but not orthologous to S. cerevisiae Bul1; a protein involved in selection of substrates for ubiquitination; mutants are viable; macrophage/pseudohyphal-induced; rat catheter biofilm induced	2.1567	0.000302
PRP18	**snRNP U5 splicing factor component; involved in positioning the 3' splice site during the 2nd catalytic step of splicing; Spider biofilm induced**	2.156508	5.55E-07
LEU3	Zn(II)2Cys6 transcription factor; predicted regulator branched-c ofhain amino acid biosynthesis genes; alkaline induced; induced by Mnl1 under weak acid stress; required for yeast cell adherence to silicone substrate; Spider biofilm induced	2.156153	4.8E-07
SPT2	Ortholog(s) have DNA binding, DNA secondary structure binding activity	2.151474	6.73E-05
SCD6	Ortholog(s) have eukaryotic initiation factor 4G binding, mRNA binding activity, role in P-body assembly, negative regulation of translational initiation, stress granule assembly and P-body, cytoplasmic stress granule localization	2.151182	2.35E-09
TCO89	**Putative homolog of *S. cerevisiae* Tco89p, which is a component of the TOR C1 complex that is involved in the regulation of growth and cell wall integrity; heterozygous null mutant displays sensitivity to rapamycin**	2.150201	0.001145
TAF145	Protein similar to S. cerevisiae Taf145p, a component of RNA polymerase II transcription factor TFIID; flucytosine repressed; likely to be essential for growth, based on an insertional mutagenesis strategy	2.149812	8.65E-05
LRG1	GTPase activator (GAP) that negatively controls small GTPases Cdc42p and Ras1p, involved in signaling pathway that controls morphogenesis in response to environmental signals	2.148863	1.65E-05

orf19.828	Putative ribosomal protein, large subunit, mitochondrial precursor; repressed by prostaglandins; Spider biofilm repressed	2.146882	2.1E-09
PRP2	Ortholog(s) have ATP-dependent activity, acting on RNA activity, role in generation of catalytic spliceosome for first transesterification step, snoRNA splicing and U2-type catalytic step 1 spliceosome localization	2.146504	0.017077
orf19.5510	Ortholog(s) have role in chromatin silencing at telomere, negative regulation of transcription from RNA polymerase II promoter by pheromones and CHRAC localization	2.142704	0.001231
orf19.6736	Protein required for mitochondrial ribosome small subunit biogenesis; role in maturation of SSU-rRNA; Spider biofilm induced	2.13875	2.89E-06
orf19.6939	Predicted ORF from Assembly 19; removed from Assembly 20; restored based on comparative genome analysis	2.13857	1.97E-05
orf19.512	**Ortholog of S. cerevisiae Kre33; essential; S. cerevisiae ortholog is essential and is required for biogenesis of the small ribosomal subunit**	2.138323	1.06E-06
AGE1	Putative GTPase activator; induced in low iron; rat catheter biofilm repressed	2.137889	0.000153
MBP1	Putative component of the MBF transcription complex involved in G1/S cell-cycle progression; non-periodic mRNA expression; predicted, conserved MBF binding sites upstream of G1/S-regulated genes	2.137428	0.001549
orf19.6903	Predicted RNA polymerase III subunit C37; Spider biofilm induced	2.135435	5.05E-06
CTA8	Essential transcription factor, mediates heat shock transcriptional induction; in the absence of heat stress, Cta8p levels are modulated by growth temperature to regulate basal expression of genes involved in protein folding	2.135345	0.004662
ash2	Ortholog(s) have histone methyltransferase activity (H3-K4 specific) activity	2.135231	5.01E-07
PAM18	Predicted component of the presequence translocase-associated import motor (PAM complex) involved in protein import into mitochondrial matrix; rat catheter biofilm induced	2.133951	1.37E-07
HTA3	Putative histone H2A; amphotericin B repressed; flucytosine induced; RNA abundance regulated by tyrosol and cell density; Spider biofilm repressed	2.133211	7.13E-07
orf19.3213	Protein of unknown function; Spider biofilm induced	2.130032	0.000278

ARX1	Putative ribosomal large subunit biogenesis protein; repressed in core stress response; repressed by prostaglandins	2.125085	2.14E-06
PCL1	Cyclin homolog; transcript induced by filamentous growth; induced by alpha pheromone in SpiderM medium	2.124848	9.28E-07
MRS7	Member of the LETM1-like protein family, mitochondrial membrane protein	2.12184	2.98E-05
GOA1	Protein required for respiratory growth, resistance to oxidants, chlamydospore formation, hyphal growth under some conditions, and virulence; relocalizes from the cytoplasm to the mitochondrion during oxidative or osmotic stress	2.118873	0.009219
BLM3	Putative proteasome activator; binds core proteasome and stimulates proteasome-mediated protein degradation by inducing gate opening; ortholog of S. cerevisiae Blm10; transcript regulated by Nrg1 and Mig1	2.117245	0.000106
RBT4	Pry family protein; required for virulence in mouse systemic/rabbit corneal infections; not filamentation; mRNA binds She3, is localized to hyphal tips; Hap43-induced; in both yeast and hyphal culture supernatants; Spider biofilm induced	2.114177	1.98E-06
YTA6	Protein similar to S. cerevisiae Yta6p ATPase but ortholog of S. cervisiae Sap1; transposon mutation affects filamentous growth; induced by Mnl1 under weak acid stress	2.113733	0.000648
HHF1	Putative histone H4; repressed in fkh2 mutant; regulated by Efg1; fluconazole induced; amphotericin B repressed; farnesol regulated; colony morphology-related gene regulation by Ssn6; Hap43-induced; rat catheter and Spider biofilm repressed	2.113402	3.55E-07
RAD18	Putative transcription factor with zinc finger DNA-binding motif; Hap43p-repressed gene	2.112434	0.00118
orf19.2680	Ortholog(s) have phosphatidylcholine flippase activity, phosphatidylethanolamine flippase activity, phosphatidylserine flippase activity	2.112338	0.00013
ELP3	Predicted histone acetyltransferase; role in regulation of transcription, tRNA wobble uridine modification; Spider biofilm induced	2.112139	6.93E-06
orf19.7489.3	Ortholog(s) have 2 iron, 2 sulfur cluster binding, iron-sulfur cluster binding activity	2.111082	0.01741
orf19.932	Putative aminophospholipid translocase (flippase); merged with orf19.2226 in Assembly 21; possibly an	2.109656	0.000103

	essential gene, disruptants not obtained by UAU1 method		
orf19.4414	**Ortholog of *C. dubliniensis CD36* : Cd36_45590, *C. parapsilosis CDC317* : CPAR2_501450, *C. auris B8441* : B9J08_003351 and *Candida tenuis NRRL Y-1498* : CANTEDRAFT_105024**	2.109599	0.021281
orf19.3813	Ortholog of ***C. dubliniensis CD36* : Cd36_44340, *C. parapsilosis CDC317* : CPAR2_302240, *Candida tenuis NRRL Y-1498* : CANTEDRAFT_105331 and *Debaryomyces hansenii CBS767* : DEHA2E03454g**	2.108524	0.000763
MDR1	Plasma membrane MDR/MFS multidrug efflux pump; methotrexate is preferred substrate; overexpression in drug-resistant clinical isolates confers fluconazole resistance; repressed in young biofilms; rat catheter biofilm induced	2.105401	1.77E-05
REP1	**Putative transcription factor involved in transcription of N-acetylglucosamine-inducible genes; involved in negative regulation of MDR1 transcription; mutants show increased resistance to azole drugs**	2.104337	0.008528
ZCF30	Predicted Zn(II)2Cys6 transcription factor; mutants are viable; repressed by fluphenazine; Spider biofilm induced	2.102931	0.013301
orf19.989	Mitochondrial ribosomal protein of the small subunit; Spider biofilm repressed	2.101352	4.39E-08
orf19.2040	Ortholog(s) have oxidoreductase activity, acting on NAD(P)H activity	2.101277	9.42E-05
orf19.5439	**Ortholog of *C. dubliniensis CD36* : Cd36_80270, *C. parapsilosis CDC317* : CPAR2_504010, *Pichia stipitis Pignal* : PICST_29581, *Candida tropicalis MYA-3404* : CTRG_06175 and *Candida albicans WO-1* : CAWG_02365**	2.100813	1.28E-05
orf19.6679	Has domain(s) with predicted metal ion binding activity	2.098047	0.000291
MSK1	Putative mitochondrial lysine-tRNA synthetase; flucytosine repressed	2.097737	0.001473
MEP2	Ammonium permease and regulator of nitrogen starvation-induced filamentation; 11 predicted transmembrane regions; in low nitrogen cytoplasmic C-terminus activates Ras/cAMP and MAPK signal transduction pathways to induce filamentation	2.097296	1.86E-06
orf19.4029	Ortholog(s) have unfolded protein binding activity, role in ribosomal large subunit assembly, ribosomal large subunit biogenesis and cytosol localization	2.092376	2.72E-06
SPB1	Putative AdoMet-dependent methyltransferase; Hap43-induced; repressed by prostaglandins; possibly essential	2.091878	0.000533

	gene, disruptants not obtained by UAU1 method; Spider biofilm induced		
POL3	Large subunit of DNA polymerase III; partially complements defects of an S. cerevisiae cdc2 mutant; differing reports about periodic (G1/S) or non-periodic mRNA expression through cell cycle; Hap43p-repressed	2.091618	9.97E-05
Protein encoded in retrotransposon Zorro3 with a potential zinc finger; lacks an ortholog in S. cerevisiae; transposon mutation affects filamentous growth		2.091292	5.93E-05
orf19.1259	Ortholog(s) have ubiquitin protein ligase activity, role in histone catabolic process, histone ubiquitination, regulation of transcription from RNA polymerase II promoter in response to oxidative stress and Lid2 complex localization	2.090708	0.000168
HOT1	**Putative transcription factor; required for inhibition of filamentous growth by farnesoic acid and for expression of PHO81; filament induced**	2.08967	0.000327
MCD1	Alpha-kleisin cohesin complex subunit; for sister chromatid cohesion in mitosis and meiosis; repressed by alpha pheromone in SpiderM medium; periodic cell-cycle expression; Hap43-repressed; rat catheter and Spider biofilm repressed	2.089503	0.000116
orf19.962	**Protein with a fungal RNA polymerase I subunit RPA14 domain; proposed to play a role in the recruitment of pol I to the promoter; Hap43-induced gene**	2.08869	1.58E-06
orf19.389	Protein of unknown function; Hap43-induced gene	2.088554	0.002105
HCM1	Protein with forkhead domain; similar to S. cerevisiae Hcm1p; Hap43p-induced gene	2.086308	0.000171
AOX1	Alternative oxidase; low abundance; constitutively expressed; one of two isoforms (Aox1p and Aox2p); involved in a cyanide-resistant respiratory pathway present in plants, protists, and some fungi, absent in S. cerevisiae; Hap43p-repressed	2.085228	0.005735
TES1	Putative acyl-CoA thioesterase	2.077561	0.005603
BEM2	Putative Rho1p GTPase activating protein (GAP); serum-induced transcript; Spider biofilm induced; flow model biofilm repressed	2.076399	4.16E-05
AAP1	Putative amino acid permease; fungal-specific; possibly essential, disruptants not obtained by UAU1 method; Spider biofilm induced	2.07549	0.011168

TOP2	DNA topoisomerase II; catalyzes ATP-dependent DNA relaxation and decatenation in vitro; Y842 predicted to be catalytic; functional homolog of S. cerevisiae Top2p; sensitive to amsacrine or doxorubicin; farnesol-upregulated in biofilm	2.073849	0.000102
DBP9	Putative DEAD-box helicase; Hap43-induced; Spider biofilm induced	2.071037	0.000918
orf19.5206	Putative chaperone protein; role in the assembly of box H/ACA snoRNPs and thus for pre-rRNA processing; Spider biofilm induced (5)	2.070922	0.005323
orf19.4518	Protein kinase of unknown function; mutants are viable	2.069709	0.000365
orf19.3432	Predicted membrane transporter, member of the drug:proton antiporter (12 spanner) (DHA1) family, major facilitator superfamily (MFS); induced by nitric oxide	2.069523	0.021518
CTA24	Putative transcription factor; positive regulator of gene expression; Efg1-repressed; member of a family of telomere-proximal genes; transcript upregulated in RHE model of oral candidiasis	2.068768	4.54E-08
MEX67	Nuclear export protein; has NTF2-like domain; interacts with Mtr2p via the NTF2-like domain	2.068656	1.45E-06
FGR29	Protein lacking an ortholog in S. cerevisiae; transposon mutation affects filamentous growth; rat catheter biofilm repressed	2.066762	0.006424
SVL3	**Protein of unknown funtion; S. cerevisiae ortholog Svl3 plays a role in endocytosis and is localized to the bud neck; Spider biofilm induced**	2.065507	7.44E-05
ENP2	Putative nucleolar protein; essential; heterozygous mutation confers resistance to 5-fluorocytosine (5-FC), 5-fluorouracil (5-FU), and tubercidin (7-deazaadenosine); Hap43-induced; Spider biofilm induced	2.062865	0.001977
orf19.6698	Ortholog(s) have asparagine-tRNA ligase activity, role in asparaginyl-tRNA aminoacylation, mitochondrial asparaginyl-tRNA aminoacylation and mitochondrion localization	2.062806	0.000267
PDX1	Pyruvate dehydrogenase complex protein X; essential component of the mitochondrial pyruvate dehydrogenase complex; role in the respiratory pathway; protein present in exponential and stationary growth phase yeast; Spider biofilm repressed	2.060829	8.51E-07

orf19.3088	bZIP transcription factor; possibly transcriptionally regulated upon hyphal formation; Hap43; F-12/CO2 early biofilm induced; Spider biofilm induced	2.059022	2.23E-05
TAC1	Zn(2)-Cys(6) transcriptional activator of drug-responsive genes (CDR1 and CDR2); binds DRE element; gene in zinc cluster region near MTL locus; resequencing indicates that TAC1 spans orf19.3188 and orf19.3189; Spider biofilm induced	2.057898	0.002091
SOL1	Cell cycle regulator; partial functional homolog of S. cerevisiae Sic1p; protein degradation regulated by SCF(CDC4) pathway; CDK phosphorylation predicted	2.05569	8.15E-05
orf19.6113	Protein of unknown function; transcript detected on high-resolution tiling arrays	2.054794	0.000194
FUN30	Ortholog(s) have ATP-dependent activity, acting on DNA, DNA binding, chromatin binding activity	2.054216	4.91E-05
TAF12	Essential component of transcription factor TFIID complex, involved in transcription regulation	2.050894	8.51E-05
MRD1	Ortholog(s) have rRNA primary transcript binding activity	2.048565	1.01E-05
BUD31	Bud31 ortholog; not subject to mating-type regulation, in contrast to S. cerevisiae Bud31 which has a role in specifying the bud site; Spider biofilm induced	2.047075	2.29E-05
orf19.7495	Protein with NADPH oxidoreductase containing flavin mononucleotide (FMN) domain; induced by nitric oxide	2.040521	0.012427
orf19.2973	Ortholog(s) have RNA binding, flap-structured DNA binding activity and role in nuclear-transcribed mRNA catabolic process, nuclear-transcribed mRNA poly(A) tail shortening, positive regulation of endodeoxyribonuclease activity	2.04042	3.26E-06
YRB2	**Ortholog(s) have role in protein export from nucleus, regulation of chromatin silencing at telomere, ribosomal small subunit export from nucleus and cytosol, nucleus localization**	2.037241	0.000191
PTH1	Putative gluconate transport protein; antigenic during human oral infection; possibly an essential gene, disruptants not obtained by UAU1 method	2.035716	1.92E-05
MIA40	Predicted component of the mitochondrial intermembrane space import machinery; Hap43p-repressed gene	2.030542	7.8E-08
TPT1	tRNA 2'-phosphotransferase; enzyme of tRNA splicing; functionally complements viability of S. cerevisiae tpt1 null mutant	2.0301	0.000116

POP3	Putative RNase MRP and nuclear RNase P component; decreased repressed by prostaglandins; Spider biofilm induced	2.028514	6.52E-06
DBP8	Protein similar to S. cerevisiae Dbp8p, an ATP-dependent helicase involved in rRNA processing; oxidative stress-repressed via Cap1p; flucytosine repressed; likely to be essential for growth, based on insertional mutagenesis	2.028461	3.07E-05
orf19.7504	Ortholog of S. cerevisiae Rts3; a component of the protein phosphatase type 2A complex; Plc1-regulated; induced in core caspofungin response; Spider biofilm induced	2.027863	1.43E-06
orf19.6705	Putative guanyl nucleotide exchange factor with Sec7 domain; required for normal filamentous growth; regulated by yeast-hyphal switch; filament induced; regulated by Nrg1, Tup1, Mob2, Hap43; mRNA binds She3; Spider biofilm induced	2.027711	4.92E-05
ELG1	Putative subunit of an alternative replication factor C complex; role in DNA replication, genome integrity, homologous recombination-mediated repair and telomere homeostasis	2.026753	0.015207
orf19.1180	Putative 2-aminoadipate transaminase; rat catheter and Spider biofilm repressed	2.023025	6.71E-07
orf19.6008.4	Ortholog(s) have structural constituent of ribosome activity and mitochondrial large ribosomal subunit, mitochondrion localization	2.02211	0.000426
SET5	Ortholog(s) have S-adenosylmethionine-dependent methyltransferase activity and role in gene silencing, histone lysine methylation, telomere maintenance	2.021323	0.000123
WAR1	**Zn(II)2Cys6 transcription factor; plays a role in resistance to weak organic acids; required for yeast cell adherence to silicone substrate; Spider biofilm induced**	2.020218	0.015289
TOM6	Predicted component of the TOM (translocase of outer membrane) complex, involved in protein import into mitochondria	2.01887	0.000589
KCH1	Ortholog of Kch1 a potassium transporter; mediates K+ influx and activates high-affinity Ca2+ influx system during mating pheromone response in S. cerevisiae; induced by alpha pheromone in SpiderM medium	2.018824	0.001304
orf19.998	Putative adapter protein; links synaptojanins Inp52 and Inp53 to the cortical actin cytoskeleton in S. cerevisiae; mutants are viable (5)	2.018687	0.003075
NCL1	Ortholog(s) have tRNA (cytosine-5-)-methyltransferase activity, tRNA binding activity	2.015969	0.000107

NIP100	p150 subunit of dynactin; required for normal spindle formation and position	2.01447	0.021359
orf19.4055	Protein similar to S. cerevisiae Ybr075wp; transposon mutation affects filamentous growth; clade-associated gene expression	2.01376	0.000529
TRM22	Has domain(s) with predicted RNA methyltransferase activity and role in RNA processing	2.013274	0.000663
orf19.4148	**Ortholog of *C. dubliniensis CD36* : Cd36_50820, *C. parapsilosis CDC317* : CPAR2_501670, *C. auris B8441* : B9J08_002922 and *Candida tenuis NRRL Y-1498* : CANTEDRAFT_115294**	2.005993	0.001311
orf19.6660	Protein of unknown function; mRNA binds to She3; Hap43-repressed; rat catheter and flow model biofilm induced	2.004827	0.000755
HAP41	**Putative Hap4-like transcription factor; Hap43-repressed; not required for response to low iron; induced by Mnl1 under weak acid stress; Spider biofilm induced**	2.004113	5.52E-07
ATO6	Putative fungal-specific transmembrane protein	2.003097	0.003457
ISA1	**Putative mitochondrial iron-sulfur protein; alkaline repressed; induced in high iron; regulated by Sef1, Sfu1, Hap43; Spider biofilm induced**	2.002492	1.41E-05
orf19.1250	Ortholog(s) have role in maturation of SSU-rRNA from tricistronic rRNA transcript (SSU-rRNA, 5.8S rRNA, LSU-rRNA) and nucleolus localization	2.001455	0.000109
orf19.398	**Ortholog of *C. dubliniensis CD36* : Cd36_08040, *C. parapsilosis CDC317* : CPAR2_207180, *C. auris B8441* : B9J08_000292 and *Candida tenuis NRRL Y-1498* : CANTEDRAFT_114140**	2.000508	0.017296
orf19.985	**Ortholog of *S. cerevisiae* : YEL043W, *C. glabrata CBS138* : CAGL0L01221g, *C. dubliniensis CD36* : Cd36_09830, *C. parapsilosis CDC317* : CPAR2_804210 and *C. auris B8441* : B9J08_004773**	1.999211	0.00072
NRD1	Putative RNA-binding protein; transcript is upregulated in an RHE model of oral candidiasis	1.998758	8.97E-06
orf19.7506	Ortholog(s) have ATPase, DNA binding, nucleosome binding activity, role in chromatin remodeling and Isw1b complex localization	1.997357	0.005511
NOP58	Ortholog of S. cerevisiae Nop58; involved in pre-rRNA process; Tn mutation affects filamentous growth; macrophage/pseudohyphal-induced; physically interacts with TAP-tagged Nop1; Spider biofilm repressed	1.996201	6.13E-08

PRR1	Putative serine/threonine protein kinase; Hog1p-induced	1.995447	8.99E-05
ATP18	F1F0 ATP synthase complex subunit; fungal-specific; gene has intron	1.994757	0.00239
orf19.4239	Planktonic growth-induced gene	1.994702	0.001214
orf19.4818	Protein similar to S. cerevisiae Ytm1p, which is involved in biogenesis of the large ribosomal subunit; transposon mutation affects filamentous growth; protein level decreases in stationary phase cultures; Hap43p-induced gene	1.992866	2.25E-05
STE13	Putative pheromone-processing dipeptidyl aminopeptidase; possible Kex2 substrate; transposon mutation affects filamentous growth; induced by low nitrogen, germ tube formation; flow model biofilm repressed	1.992409	0.006133
orf19.972	Ortholog(s) have 5'-flap endonuclease activity, role in DNA-dependent DNA replication and Slx1-Slx4 complex localization	1.986759	0.011963
orf19.2594	**Ortholog(s) have RNA polymerase I activity and role in nucleolar large rRNA transcription by RNA polymerase I, transcription by RNA polymerase I, transcription initiation from RNA polymerase I promoter**	1.982189	8.09E-05
ATP14	Putative mitochondrial F1F0 ATP synthase subunit; macrophage/pseudohyphal-induced	1.981142	3.53E-06
GPR1	Plasma membrane G-protein-coupled receptor of the cAMP-PKA pathway; detects lactate and triggers signaling pathway that regulates beta-glucan masking and immune evasion; binds Gpa2; regulates HWP1 and ECE1; required for WT hyphal growth	1.980125	0.001614
TAF7	**Putative TFIID subunit involved in RNA polymerase II transcription initiation; possibly an essential gene, disruptants not obtained by UAU1 method**	1.980081	0.01228
MED1	RNA polymerase II mediator complex subunit; RNA polymerase II transcription cofactor	1.979143	0.000066
orf19.4728	Ortholog(s) have role in histone deacetylation and Set3 complex localization	1.978776	0.002323
SIZ1	Possible SUMO/Smt3 ligase; Rim101-repressed	1.978135	0.000853
orf19.4575	**Ortholog of *S. cerevisiae* : YPL109C, *C. glabrata CBS138* : CAGL0J07018g, *C. dubliniensis CD36* : Cd36_42050, *C. parapsilosis CDC317*: CPAR2_403320 and *C. auris B8441* : B9J08_001404**	1.977794	0.00384

DIA4	Ortholog(s) have serine-tRNA ligase activity, role in mitochondrial seryl-tRNA aminoacylation, spore germination and mitochondrion localization	1.976746	8.56E-05
orf19.3156	Protein of unknown function; induced by Mnl1 under weak acid stress	1.974984	0.000621
BFR1	Protein involved in the maintenance of normal ploidy; S. cerevisiae ortholog cofers Brefeldin A resistance; stationary phase enriched protein; Spider biofilm repressed	1.974664	1.55E-06
orf19.429	Putative non-canonical poly(A) polymerase; repressed by nitric oxide; Spider biofilm induced	1.967025	0.012462
OYE22	Putative NADPH dehydrogenase; rat catheter biofilm induced	1.966788	0.000589
orf19.7497	Ortholog(s) have ubiquitin-protein transferase activity, role in ubiquitin-dependent protein catabolic process and Cul3-RING ubiquitin ligase complex localization	1.96658	0.000576
orf19.5555	Ortholog of ***C. dubliniensis CD36*** **: Cd36_63300,** ***Candida tropicalis NEW ASSEMBLY*** **: CTRG1_05769,** ***Candida tropicalis MYA-3404*** **: CTRG_05769 and** ***Candida albicans WO-1*** **: CAWG_05077**	1.966435	0.000919
orf19.686	Protein of unknown function; regulated by Nrg1	1.965701	0.00078
MTM1	Ortholog(s) have pyridoxal phosphate binding activity, role in cellular iron ion homeostasis, pyridoxal phosphate transport and mitochondrion localization	1.964096	1.76E-05
GPT1	GABA/polyamine transporter; 9 to 11 membrane spanning segments; complements GABA uptake defect of an S. cerevisiae uga4 put4 gap1 triple mutant; complements growth of an S. cerevisiae spe1 mutant under polyamine limitation	1.962008	0.00123
MSH6	Protein similar to S. cerevisiae Msh6p, which is involved in mismatch repair; repressed under Cdc5p depletion; Hap43p-induced gene	1.961575	9.54E-06
NTG1	Protein with strong similarity to both S. cerevisiae Ntg1 and Ntg2 DNA repair glycosylases; transcript induced by iron; Hap43-repressed gene; Spider biofilm induced	1.959213	0.000654
MKT1	Ortholog(s) have mRNA 3'-UTR binding activity and role in biological process involved in interspecies interaction between organisms, cellular response to DNA damage stimulus	1.957422	0.000495
orf19.216	Protein with a metallo-dependent phosphatase domain; ketoconazole-induced; upregulation correlates with clinical development of fluconazole resistance (1, 6)	1.956794	0.001286

WSC2	Putative cell wall integrity and stress response protein; mRNA binds She3; Spider biofilm induced	1.955487	0.000412
orf19.5412	**Ortholog of *S. cerevisiae* : ECM9, *C. glabrata CBS138* : CAGL0L02255g, *C. dubliniensis CD36* : Cd36_80470, *C. parapsilosis CDC317* : CPAR2_503390 and *C. auris B8441* : B9J08_003812**	1.955315	0.007691
GZF3	GATA-type transcription factor; oxidative stress-induced via Cap1; mutant has abnormal colony morphology and altered sensitivity to fluconazole, LiCl, and copper; Spider biofilm induced	1.954574	0.000828
orf19.863	Ortholog(s) have structural constituent of ribosome activity and mitochondrial large ribosomal subunit localization	1.954428	0.000044
orf19.6503	Ortholog(s) have structural constituent of ribosome activity, role in mitochondrial translation and mitochondrial large ribosomal subunit localization	1.952333	5.93E-05
IFV1	Predicted ORF overlapping the Major Repeat Sequence on chromosome R; member of a family encoded by FGR6-related genes in the RB2 repeat sequence	1.950853	0.000488
CHS3	Major chitin synthase of yeast and hyphae; synthesizes short-chitin fibrils; Chs4-activated; transcript induced at yeast-hyphal transition; Chs1 and Chs2, but not Chs3, are inhibited by the protoberberine HWY-289; Spider biofilm induced	1.946674	6.43E-06
CKB1	**Regulatory subunit of protein kinase CK2 (casein kinase II), beta subunit; null mutants are hypersensitive to caspofungin and hydrogen peroxide medium**	1.946196	5.35E-05
ESC4	**Protein similar to S. cerevisiae Esc4; a protein that represses transposition; transposon mutation affects filamentation; rat catheter biofilm repressed**	1.94571	0.005279
ALS1	Cell-surface adhesin; adhesion, virulence, immunoprotective roles; band at hyphal base; Rfg1, Ssk1, Spider biofilm induced; flow model biofilm repressed; CAI-4 strain background effects; promoter bound Bcr1, Tec1, Efg1, Ndt80, and Brg1	1.944365	0.01296
orf19.292	**Ortholog of *C. dubliniensis CD36* : Cd36_82960, *C. parapsilosis CDC317* : CPAR2_102390, *C. auris B8441* : B9J08_001199, *Candida tenuis NRRL Y-1498* : CANTEDRAFT_133198 and *Candida albicans WO-1* : CAWG_02618**	1.943987	7.55E-05
orf19.284	**Ortholog of *C. dubliniensis CD36* : Cd36_82890, *Debaryomyces hansenii CBS767* : DEHA2G01034g, *Pichia stipitis Pignal* : PICST_32242**	1.943987	7.55E-05

	**and *Candida tropicalis NEW ASSEMBLY* : CTRG1_02580**		
orf19.1447	Has domain(s) with predicted DNA-binding transcription factor activity, sequence-specific DNA binding activity and role in regulation of transcription, DNA-templated	1.943209	0.003757
orf19.2850	**Protein of unknown function; induced by nitric oxide independent of Yhb1p**	1.94301	0.007608
NOT3	Transcriptional regulator; not required for buccal epithelial cell adherence or virulence in mouse systemic infection; null mutant colonies exhibit slightly decreased filamentation ratio; required for yeast adherence to silicone substrate	1.942414	2.42E-05
orf19.7245	Ortholog(s) have tRNA-5-taurinomethyluridine 2-sulfurtransferase activity, role in mitochondrial tRNA wobble position uridine thiolation and mitochondrion localization	1.941216	2.27E-05
DUS1	Predicted tRNA dihydrouridine synthase; Spider biofilm induced	1.940351	0.001153
orf19.6160	Ortholog(s) have role in eisosome assembly and eisosome, membrane raft localization	1.937732	1.24E-06
ASE1	Putative microtubule-associated protein; member of conserved Mcm1p regulon; periodic mRNA expression, peak at cell-cycle G2/M phase	1.936591	0.014939
RPF1	Putative nucleolar protein with a predicted role in the assembly and export of the large ribosomal subunit; essential for growth; rat catheter and Spider biofilm induced	1.936347	3.61E-05
orf19.6730	**Ortholog of *S. cerevisiae* : YCR016W, *C. glabrata CBS138* : CAGL0L07832g, *C. dubliniensis CD36* : Cd36_87480, *C. parapsilosis CDC317* : CPAR2_808240 and *C. auris B8441* : B9J08_001753**	1.934479	0.000104
orf19.5722	Ortholog(s) have DNA binding activity, role in rDNA heterochromatin assembly, termination of RNA polymerase I transcription and nucleolus, rDNA heterochromatin localization	1.933711	0.009066
TFA1	Protein with polyglutamate motifs and abundant Ser/Thr residues; described as a subunit of TFIIE, which is a basal transcription initiation factor of RNA Polymerase II; possibly an essential gene, disruptants not obtained by UAU1 method	1.930994	0.00023
orf19.6090	Putative nucleolar protein with a predicted role in pre-rRNA processing and ribosome biogenesis; repressed by nitric oxide; required for flow model biofilm formation; Spider biofilm repressed	1.930673	6.64E-06

orf19.6271	Ortholog(s) have role in mRNA splicing, via spliceosome and transcriptionally active chromatin localization (5)	1.929353	0.000393
orf19.7091	Protein of unknown function; induced by nitric oxide; Spider biofilm repressed	1.928822	1.29E-05
OAC1	Putative mitochondrial inner membrane transporter; rat catheter biofilm induced	1.927893	0.001538
ARP5	Ortholog(s) have 3'-5' DNA helicase activity	1.926332	1.92E-05
UTP13	**Putative U3 snoRNA-associated protein; Hap43-induced; repressed in core stress response; physically interacts with TAP-tagged Nop1**	1.925415	0.000142
orf19.1864	**Ortholog(s) have role in proteasome-mediated ubiquitin-dependent protein catabolic process, rescue of stalled ribosome, ribosome-associated ubiquitin-dependent protein catabolic process and RQC complex localization**	1.924967	0.000222
SET1	Lysine histone methyltransferase; methylates histone H3 K4; regulates of white-opaque switch, epithelial cell adhesion, agar-embedded filamentation, virulence in mice; unique N-terminus immunogenic in human; rat catheter biofilm repressed	1.924143	0.007098
CDC54	Putative pre-replication complex helicase subunit; transcript regulated by Nrg1 and Mig1; periodic mRNA expression, peak at cell-cycle M/G1 phase; Hap43-induced	1.924091	0.000821
ZNC1	Zn(2)-Cys(6) transcription factor; regulated by Gcn2 and Gcn4; gene located in zinc cluster region of Chromosome 5, near the MTL locus; required for yeast cell adherence to silicone substrate; Spider biofilm induced	1.923493	0.001704
orf19.4250	Ortholog of ***C. dubliniensis CD36*** **: Cd36_52200,** ***C. parapsilosis CDC317*** **: CPAR2_502480,** ***C. auris B8441*** **: B9J08_004350 and** ***Candida tenuis NRRL Y-1498*** **: CANTEDRAFT_115972**	1.923379	0.000146
VPS41	Protein similar to S. cerevisiae Vps41p, which is involved in vacuole organization and biogenesis; transposon mutation affects filamentous growth; regulated by Gcn4p; induced in response to amino acid starvation (3-aminotriazole treatment)	1.92213	0.018005
BRN1	Putative condensin complex subunit; cell-cycle regulated periodic mRNA expression	1.921802	0.018454
RPN2	Putative 26S proteasome subunit; transcript regulated by Mig1; caspofungin repressed; regulated by Gcn2 and Gcn4; gene used for strain identification by multilocus sequence typing	1.921757	7.29E-05

NSA2	Putative protein constituent of 66S pre-ribosomal particles; Hap43-induced; repressed by prostaglandins	1.91895	3.24E-06
orf19.7397.1	Predicted ORF identical to NSA2	1.91895	3.24E-06
orf19.7457	Protein with Mob2p-dependent hyphal regulation	1.91894	0.002928
orf19.7441	Ortholog(s) have role in lipid homeostasis, nuclear envelope organization and nuclear envelope localization	1.918206	0.009601
orf19.1479	Ortholog of the mitochondria localized S. cerevisiae Pib2 protein of unknown function; has a FYVE zinc finger domain; Spider biofilm induced	1.918068	0.006452
LIP4	Secreted lipase, member of a differentially expressed lipase gene family with possible roles in nutrition and/or in creating an acidic microenvironment; expressed more strongly during mucosal infections than during systemic infections	1.917881	0.019792
RPA34	Putative RNA polymerase I subunit; rat catheter biofilm induced	1.916684	0.000245
VAM3	Predicted syntaxin-like vacuolar t-SNARE, involved in vacuolar inheritance	1.9155	0.00033
HHT21	Putative histone H3; amphotericin B repressed; regulated by Efg1, farnesol; Hap43-induced; rat catheter and Spider biofilm repressed	1.915385	2.02E-07
COX11	Cytochrome oxidase assembly protein; transcript regulated by Nrg1; protein repressed during the mating process; Hap43-repressed gene; rat catheter biofilm induced	1.9143	9.73E-06
FMP40	Putative protein of unknown function, transcription is positively regulated by Tbf1p	1.912301	0.000738
PDE1	Low affinity cyclic nucleotide phosphodiesterase; mediates cAMP signaling in response to glucose or intracellular acidification; macrophage-repressed; Spider biofilm induced	1.911999	0.00382
orf19.3577.1	Ortholog(s) have mitochondrial intermembrane space localization	1.911501	0.008761
TRY2	Transcription factor; regulator of yeast form adherence; required for yeast cell adherence to silicone substrate; Spider biofilm induced	1.910538	0.001148
MTG1	Putative mitochondrial GTPase; likely essential for respiratory competence and in large ribosomal subunit assembly; mitochondrial translation; Spider biofilm induced	1.909128	0.001649
orf19.3453	Has domain(s) with predicted role in signal transduction and intracellular anatomical structure localization	1.907971	0.000448

RNH35	Putative ribonuclease H2 catalytic subunit; flucytosine induced; Spider biofilm repressed	1.906957	5.06E-05
orf19.7103	Predicted exonuclease; Spider biofilm induced	1.906322	0.005276
MDL2	Putative mitochondrial, half-size MDR-subfamily ABC transporter	1.904614	9.06E-05
orf19.4301	**Ortholog(s) have role in chromatin silencing at telomere, rDNA heterochromatin assembly, regulation of transcription by RNA polymerase II, silent mating-type cassette heterochromatin assembly**	1.902229	0.001445
orf19.6658	Stationary phase enriched protein; predicted ORF from Assembly 19; removed from Assembly 20; subsequently reinstated in Assembly 21 based on comparative genome analysis	1.897978	0.000285
RPL37	Ribosomal protein L37; Hap43-induced; Spider biofilm repressed	1.895078	2.16E-07
orf19.4820	Protein of unknown function; Spider biofilm induced	1.894591	0.000231
orf19.6418	Ortholog(s) have unfolded protein binding activity and role in protein import into nucleus, ribosomal large subunit biogenesis	1.893599	0.000173
Ortholog(s) have role in positive regulation of cellular response to hypoxia, regulation of protein glycosylation, sterol regulatory element binding protein cleavage		1.89205	0.000102
orf19.1285	Plasma membrane-localized protein of unknown function; Hap43p-repressed gene	1.887774	0.007372
RSM18	Ortholog(s) have structural constituent of ribosome activity and mitochondrial small ribosomal subunit localization	1.887637	3.57E-06
UTP6	Putative U3 snoRNA-associated protein; Hap43-induced; transposon mutation affects filamentous growth; repressed by prostaglandins	1.886848	4.54E-06
orf19.4988	S. cerevisiae ortholog Sae2 is an endonuclease that processes hairpin DNA structures with the MRX complex, involved in meiotic and mitotic double-strand break repair; upregulated in a cyr1 null mutant	1.885588	0.010267
CAB3	Putative phosphopantothenoylcysteine decarboxylase, binds to protein phosphatase Ppz1p and regulates its activity	1.885001	0.000181
MIF2	Centromere-associated protein; similar to CENP-C proteins; Cse4p and Mif2p colocalize at C. albicans centromeres	1.884007	0.001679

RLR1	Putative THO complex subunit; possibly an essential gene, disruptants not obtained by UAU1 method; protein newly produced during adaptation to the serum	1.883976	0.001075
SMC5	Protein similar to S. cerevisiae Smc5p, which is involved in DNA repair; transposon mutation affects filamentous growth	1.881653	0.003495
FYV5	Protein with a predicted role maturation of 18S rRNA; rat catheter biofilm induced	1.879563	0.003718
orf19.188	Ortholog of Candida albicans WO-1 : CAWG_04234 (4)	1.878876	0.01362
BUD23	Putative methyltransferase; Hap43-induced; repressed by prostaglandins	1.878532	5.03E-05
orf19.3737	Protein with a Vps9 vacuolar protein sorting protein domain; Hap43-repressed; repressed by ciclopirox olamine; Spider biofilm induced	1.876955	0.009469
VPS72	Ortholog(s) have histone binding activity, role in chromatin remodeling, histone exchange and Swr1 complex localization	1.874956	0.001382
CAF17	Ortholog(s) have role in protein maturation by [2Fe-2S] cluster transfer, protein maturation by [4Fe-4S] cluster transfer and mitochondrial matrix localization	1.871305	0.000189
PBS2	MAPK kinase (MAPKK); role in osmotic and oxidative stress responses, oxidative stress adaptation; required for stress regulation of Hog1p localization and activity; functional homolog of S. cerevisiae Pbs2p	1.87033	0.000375
orf19.22	Protein with homology to peroxisomal membrane proteins; Sef1p-, Sfu1p-, and Hap43p-regulated gene (5, 6)	1.868094	0.000483
orf19.5433	Ortholog(s) have protein folding chaperone activity, role in mitochondrial respiratory chain complex III assembly and mitochondrial matrix, mitochondrion localization (5)	1.865861	0.009457
PSO2	Putative DNA cross-link repair protein; expressed in opaque or white MTLa/MTLa or MTLalpha/MTLalpha, but not MTLa/MTLalpha cells; telomere-proximal gene; mutation does not affect white-to-opaque phenotypic switching	1.865576	0.012336
orf19.6431	Protein of unknown function; Spider biofilm induced	1.861074	0.019439
YOX1	**Putative homeodomain-containing transcription factor; transcriptional repressor; periodic mRNA expression, peak at cell-cycle G1/S phase**	1.85472	0.001073
MEF2	Putative mitochondrial translation elongation factor; caspofungin induced	1.853041	0.006166

orf19.3100	Protein with t-SNARE domains and a microtubule associated domain; Hap43-induced gene; repressed by alpha pheromone in SpiderM medium	1.852519	0.005979
SLX4	Putative endonuclease involved in DNA repair	1.852443	0.002482
orf19.433	Ortholog(s) have role in cellular protein-containing complex assembly, intra-Golgi vesicle-mediated transport and TRAPPII protein complex, trans-Golgi network localization	1.851395	0.00489
orf19.3793	Protein of unknown function; mRNA binds She3; regulated by Nrg1; upregulated in a cyr1 or ras1 mutant	1.84726	6.79E-05
orf19.5201.1	Has domain(s) with predicted ATPase inhibitor activity, role in negative regulation of ATPase activity, negative regulation of nucleotide metabolic process and mitochondrion localization	1.845967	0.002252
orf19.7604	Ortholog(s) have role in early endosome to Golgi transport, establishment or maintenance of cell polarity, hyphal growth, intra-Golgi vesicle-mediated transport, regulation of GTPase activity	1.84212	0.008875
orf19.1195	Ortholog(s) have metalloendopeptidase activity, role in cellular iron ion homeostasis, protein processing involved in protein targeting to mitochondrion, protein stabilization and mitochondrial matrix localization	1.841375	0.000548
STU2	Ortholog(s) have microtubule binding, microtubule plus end polymerase, microtubule plus-end binding activity	1.84105	0.011564
orf19.3114	Predicted pseudouridine synthase; Spider biofilm induced	1.840954	0.007563
orf19.5772	Ortholog(s) have role in chromatin remodeling, histone exchange and Swr1 complex, cytosol, nucleus localization	1.838914	0.002055
orf19.3539	Predicted nucleolar S-adenosylmethionine-dependent rRNA methyltransferase; Spider biofilm induced (5)	1.836277	0.011757
RPS25A	Ribosomal protein; macrophage/pseudohyphal-induced after 16 h; repressed upon phagocytosis by murine macrophage; transcript positively regulated by Tbf1; 5'-UTR intron; Hap43-induced; Spider biofilm repressed	1.834792	1.42E-06
orf19.3821	Ortholog of ***C. dubliniensis CD36*** **: Cd36_44290,** ***C. parapsilosis CDC317*** **: CPAR2_500740,** ***C. auris B8441*** **: B9J08_001760 and** ***Candida tenuis NRRL Y-1498*** **: CANTEDRAFT_137302**	1.834687	0.002312
orf19.3030	Ortholog(s) have phosphatidylinositol-3,5-bisphosphate binding, phosphatidylinositol-3-phosphate binding, phosphatidylinositol-4-phosphate binding activity	1.832064	0.005649

orf19.263.1	Protein of unknown function; gene has intron; Spider biofilm induced (1, 2)	1.830355	0.000504
GDE1	Glycerophosphocholine phosphodiesterase; mutation confers hypersensitivity to 5-fluorouracil (5-FU); F-12/CO2 early biofilm induced	1.827902	0.00348
CRL1	Predicted GTPase of RHO family; CAAX motif geranylgeranylated; expression in S. cerevisiae causes dominant-negative inhibition of pheromone response	1.827477	1.56E-05
orf19.5569	Ortholog(s) have role in establishment of mitotic sister chromatid cohesion, maintenance of rDNA, mitotic sister chromatid segregation and chromosome, telomeric region, nuclear envelope localization	1.825647	0.002208
ASK1	**Essential subunit of the Dam1 (DASH) complex, which acts in chromosome segregation by coupling kinetochores to spindle microtubules**	1.825014	0.020986
orf19.4225.1	Ortholog(s) have oxoglutarate dehydrogenase (succinyl-transferring) activity, structural constituent of ribosome activity and role in 2-oxoglutarate metabolic process, tricarboxylic acid cycle	1.823264	0.000605
DES1	Putative delta-4 sphingolipid desaturase; planktonic growth-induced gene	1.8226	0.000791
PEA2	Putative coiled-coil polarisome; predicted role in polarized morphogenesis, cell fusion, and low affinity Ca2+ influx; rat catheter biofilm induced	1.821415	0.009104
PUS1	**Putative pseudouridine synthase; predicted role in snRNA pseudouridine synthesis, tRNA pseudouridine synthesis; Spider biofilm induced**	1.82056	0.001125
orf19.173	C2H2 transcription factor; induced by Mnl1 under weak acid stress	1.818406	0.007548
CAP1	AP-1 bZIP transcription factor; apoptotic, oxidative stress response/resistance, multidrug resistance; nuclear in oxidative stress; complements S. cerevisiae yap1 mutant; oralpharyngeal candidasis-, human neutrophil, Spider biofilm induced	1.818103	5.28E-05
orf19.4369	Ortholog of S. cerevisiae Spp41; protein involved in negative regulation of expression of spliceosome components PRP4 and PRP3 in S. cerevisiae; mutants are viable	1.816378	0.014232
FUR4	Putative uracil permease	1.815118	0.009809
CSL4	Ortholog(s) have role in exonucleolytic trimming to generate mature 3'-end of 5.8S rRNA from tricistronic rRNA transcript (SSU-rRNA, 5.8S rRNA, LSU-rRNA) and mRNA processing	1.815026	0.000142

orf19.2260	Putative transcription factor with zinc finger DNA-binding motif	1.813866	0.008303
orf19.5495	Putative RNA-binding protein; induced by alpha pheromone in SpiderM medium	1.813781	0.001153
orf19.397	Ortholog(s) have structural constituent of ribosome activity and mitochondrial large ribosomal subunit localization	1.813557	2.73E-06
RSC58	Component of the RSC chromatin remodeling complex	1.812031	0.00054
orf19.223	Putative serine/threonine protein kinase; Hap43-repressed; induced by prostaglandins; possibly an essential gene, disruptants not obtained by UAU1 method; flow model biofilm induced; Spider biofilm induced (5, 6, 7, 8, 9)	1.811638	0.000507
TRP2	Putative anthranilate synthase with a predicted role in tryptophan biosynthesis; regulated by Gcn2p and Gcn4p	1.811334	0.000442
MUM2	Protein similar to S. cerevisiae Mum2, a protein essential for meiotic DNA replication and sporulation; induced by alpha pheromone in SpiderM medium; transcript regulated by Tup1	1.810335	0.000244
DST1	Ortholog(s) have RNA polymerase II complex binding, RNA polymerase II complex recruiting activity	1.809316	0.001455
SNT1	**Ortholog of S. cerevisiae Snt1; an NAD-independent histone deacetylase; heterozygous transposon mutation affects filamentous growth; null mutants are viable**	1.809242	0.0006
FTR2	High-affinity iron permease; probably interacts with ferrous oxidase; regulated by iron level, ciclopirox olamine, amphotericin B, caspofungin; complements S. cerevisiae ftr1 iron transport defect; Hap43-repressed; Spider biofilm induced	1.809165	1.46E-05
SRP54	Putative signal recognition particle (SRP) subunit; induced in ssr1 null; Spider biofilm repressed	1.807708	0.000144
orf19.94	Protein of unknown function; Spider biofilm induced	1.807417	0.000493
orf19.1163	Has domain(s) with predicted GTP binding activity	1.806269	0.000173
MAS1	Putative mitochondrial processing protease; Hap43-repressed; transcript regulated by Nrg1; oxidative stress-induced via Cap1	1.799535	0.000322
SHE3	mRNA-binding protein that localizes specific mRNAs to daughter yeast cells and to hyphal tips; required for normal filamentation and host epithelial cell damage; ortholog of S. cerevisiae She3 but target mRNAs differs	1.798133	0.00239

orf19.1825	Protein of unknown function; mutants are viable; filament induced; regulated by Nrg1, Rfg1, Tup1	1.797875	0.021833
orf19.4502	Ortholog(s) have ATPase, DNA binding, TBP-class protein binding activity (5)	1.796214	0.001047
orf19.2697	Ortholog(s) have ubiquitin protein ligase activity, ubiquitin-protein transferase activity	1.795269	0.003497
orf19.4844	Ortholog(s) have 3'-5' RNA helicase activity, RNA binding, polynucleotide adenylyltransferase activity, protein-macromolecule adaptor activity	1.793663	0.000357
orf19.4150	Putative glutaredoxin; induced by nitric oxide; Spider biofilm induced	1.791797	0.000348
TRM2	Putative tRNA methyltransferase; repressed by prostaglandins; Spider biofilm induced	1.791387	0.000325
ZCF11	Zn(II)2Cys6 transcription factor; required for wild-type filamentous growth; mRNA binds She3	1.791047	0.013578
CDC47	Phosphorylated protein described as having role in control of cell division; RNA abundance regulated by tyrosol and cell density; merged with orf19.201 in Assembly 20; unmerged from orf19.201 in a revision of Assembly 21	1.790913	0.000114
VPS16	Protein similar to S. cerevisiae Vps16p, which is involved in protein-vacuolar targeting; likely to be essential for growth, based on insertional mutagenesis; downregulated in biofilm or in azole-resistant strain that overexpresses MDR1	1.790347	0.00323
MAC1	Copper fist transcription factor; regulator of CTR1 copper transporter; induces CTR1 in low-copper conditions; required for copper regulation of SOD1 and SOD3; Spider biofilm induced	1.789565	0.004942
ISU1	Protein with similarity to NifU; possible role in iron-sulfur cluster biogenesis; induced in low iron; rat catheter biofilm induced; rat catheter and Spider biofilm induced	1.788929	4.98E-05
CYM1	Putative metalloprotease of the mitochondrial intermembrane space; rat catheter biofilm induced	1.787978	0.001488
PEX1	Peroxisomal ATPase involved in import of peroxisomal matrix proteins; involved in oxidative stress tolerance	1.786981	0.01092
ECM22	Zn(II)2Cys6 transcription factor; rat catheter and Spider biofilm induced	1.784863	9.43E-05
ENA21	Predicted P-type ATPase sodium pump; Gcn4p-regulated; flucytosine, amphotericin B, or ketoconazole-induced; osmotic stress-induced; overlaps orf19.5170.1, which is annotated as a blocked reading frame; Spider biofilm induced	1.78435	3.31E-05

orf19.3661	Putative deubiquitinating enzyme; induced by Mnl1 under weak acid stress (5)	1.783682	0.002286
SEF2	Zn(II)2Cys6 transcription factor, required for normal resistance to copper; repressed by Sfu1 in high-iron conditions; regulated by Sef1, Sfu1, and Hap43; rat catheter and Spider biofilm induced	1.783434	0.019737
DOT1	Putative modulator of white-opaque switching	1.781179	0.001091
orf19.5876	Protein of unknown function; Cyr1-repressed; induced by alpha pheromone in SpiderM medium; rat catheter and Spider biofilm induced	1.778493	0.000462
WH11	White-phase yeast transcript; expression in opaques increases virulence/switching; mutant switches as WT; Hap43, hypoxia, ketoconazol induced; required for RPMI biofilm; Bcr1-induced in RPMI a/a biofilm; rat catheter, Spider biofilm induced	1.778144	0.012877
TIF3	Putative translation initiation factor; genes encoding ribosomal subunits, translation factors, and tRNA synthetases are downregulated upon phagocytosis by murine macrophage	1.774535	3.98E-05
YTM1	Putative mRNA splicing factor; ortholog is essential in S. cerevisiae; Spider biofilm induced	1.772001	0.000257
IRS4	Protein with roles in cell wall integrity, systemic (but not oral) murine infection, adherence, hyphal growth, and agar-embedded filamentous growth; antigenic in human infection; similar to S. cerevisiae Irs4p	1.769062	0.002814
TIF4631	Putative translation initiation factor eIF4G; overexpression causes hyperfilamentation; hyphal- and macrophage-induced; genes encoding some translation factors are downregulated upon phagocytosis by murine macrophage	1.768282	0.000372
FKH2	Forkhead transcription factor; morphogenesis regulator; required for wild-type hyphal transcription, cell separation, and for virulence in cell culture; mutant lacks true hyphae, is constitutively pseudohyphal; upregulated in RHE model	1.766495	0.005161
ECM16	Ortholog of S. cerevisiae Ecm16, an essential DEAH-box ATP-dependent RNA helicase specific to the U3 snoRNP required for 18S rRNA synthesis; Hap43-induced; Spider biofilm induced	1.766377	0.009572
RHD3	GPI-anchored yeast-associated cell wall protein; induced in high iron; clade-associated gene expression; not essential for cell wall integrity; fluconazole-repressed; flow model and Spider biofilm repressed	1.766087	4.84E-05

RPT2	Putative ATPase of the 19S regulatory particle of the 26S proteasome; oxidative stress-induced via Cap1; Spider biofilm repressed	1.766004	7.49E-06
RPD31	Putative histone deacetylase; involved in regulation of white-opaque switch; Spider biofilm repressed	1.761823	0.000923
SLN1	Histidine kinase involved in a two-component signaling pathway that regulates cell wall biosynthesis; mutants are sensitive to growth on H2O2 medium; rat catheter and Spider biofilm induced	1.760886	0.008331
orf19.6943	Predicted ORF from Assembly 19; removed from Assembly 20; subsequently reinstated in Assembly 21 based on comparative genome analysis (3)	1.760308	0.002019
CSU57	Protein involved in utilization of L-sorbose as carbon source	1.759861	0.006086
SMI1B	Putative cell wall assembly regulatory protein; Rim101-repressed; possibly an essential gene, disruptants not obtained by UAU1 method	1.754473	0.005975
SEC7	Putative guanine nucleotide exchange factor (GEF); mutation confers hypersensitivity to Brefeldin A; caspofungin repressed	1.75058	0.00084
orf19.2414	Ortholog of S. cerevisiae Mpm1; a mitochondrial intermembrane space protein of unknown function; Hap43-repressed; Spider biofilm induced	1.748532	0.000121
CPP1	VH1 family MAPK phosphatase; regulates Cst20-Hst7-Cek1-Cph1 filamentation pathway; negatively regulates mating, represses yeast-hyphal switch; required for virulence in mice; yeast-enriched; induced by alpha pheromone in SpiderM medium;	1.748265	0.021475
HAP5	Component of CCAAT-binding transcription factor; roles in filamentous growth, low-iron induction of FRP1; regulates genes involved in respiratory growth; functional homolog of S. cerevisiae Hap5p; Cap1p-dependent expression in low iron	1.744485	0.005058
orf19.6435	Highly conserved subunit of mitochondrial pyruvate carrier; Hap43-repressed; Spider biofilm repressed	1.741328	0.000797
orf19.5655	Has domain(s) with predicted 2 iron, 2 sulfur cluster binding, iron ion binding, oxidoreductase activity, oxidoreductase activity and acting on paired donors,	1.740648	0.01027
FUM12	Putative fumarate hydratase; enzyme of citric acid cycle; fluconazole, Efg1 repressed; induced in high iron; protein present in exponential and stationary growth phase	1.74009	1.49E-05
UBP13	Ortholog of S. cerevisiae Ubp13; putative ubiquitin carboxyl-terminal hydrolase; flow model biofilm induced; rat catheter biofilm repressed	1.737914	0.000196

SXM1	Ortholog(s) have role in mRNA export from nucleus, protein import into nucleus and cytoplasm, nucleus localization	1.736229	0.000202
RPA135	Putative RNA polymerase I subunit A135; repressed by prostaglandins	1.735325	0.00016
orf19.3901	Putative benzene desulfurase; induced by nitric oxide independent of Yhb1p	1.73508	0.001993
CDC37	Chaperone for Crk1p; interacts with Crk1p kinase domain and with Sti1p; putative phosphorylation site at Ser14; functional homolog of S. cerevisiae Cdc37p; likely to be essential for growth; regulated by Gcn2p and Gcn4p	1.734081	0.005283
RPO26	Putative RNA polymerase subunit; heterozygous null mutant exhibits resistance to parnafungin in the C. albicans fitness test	1.73365	1.75E-05
MRE11	Putative DNA double-strand break repair factor; involved in response to oxidative stress and drug resistance	1.729712	0.009544
UBP7	Ortholog(s) have thiol-dependent deubiquitinase activity and role in protein deubiquitination	1.729489	0.002087
FHL1	**Fork-head transcription factor; forms a heterodimer with Ifh1 that is tethered to promoters by Tbf1; positively regulates rRNA and ribosomal protein gene transcription**	1.728415	0.00187
TIP120	Protein similar to human CAND1 (Cullin-Associated Nedd8-Dissociated) protein involved in regulation of SCF complexes; binds unnedddylated cullin Cdc53; mutants are viable	1.724654	0.001807
orf19.688	Mitochondrial ribosomal protein of the small subunit; S. cerevisiae ortholog is essential for viability; Spider biofilm repressed	1.722544	2.81E-05
UGA11	Putative gamma-aminobutyrate (GABA) transaminase; macrophage-induced; overlaps orf19.854.1, which is a region annotated as a blocked reading frame; Spider biofilm induced	1.721769	0.004748
MRR1	Putative Zn(II)2Cys6 transcription factor; regulator of MDR1 transcription; gain-of-function mutations cause upregulation of MDR1 (a plasma membrane multidrug efflux pump) and multidrug resistance; Hap43-induced	1.721682	0.002156
NPL6	Component of the RSC chromatin remodeling complex; Hap43-induced; Spider biofilm repressed	1.721393	0.002138
CSM3	Putative subunit of a replication fork-pausing checkpoint complex	1.720264	0.001512
orf19.2476	Ortholog(s) have histone H3-tri/di/monomethyl-lysine-4 demethylase activity (5)	1.719688	0.016854

orf19.6931	Putative cleavage factor I subunit; heterozygous null mutant exhibits hypersensitivity to parnafungin and cordycepin in the C. albicans fitness test (5)	1.717582	0.000463
RTG1	RNA polymerase II transcription factor involved in regulation of galactose catabolism genes; as result of transcriptional rewiring, performs role analogous to Gal4p in S. cerevisiae; involved in regulation of sphingolipid homeostasis	1.714822	0.000241
orf19.2333	Ortholog(s) have role in retrograde transport, endosome to Golgi and cytoplasm, late endosome localization	1.714227	0.000065
orf19.4878	Protein of unknown function; Hap43-repressed; rat catheter biofilm repressed	1.714189	0.009218
TRM1	Putative N2,N2-dimethylguanine tRNA methyltransferase; induced upon adherence to polystyrene	1.713639	0.003232
PIF1	DNA helicase and 3'-5' exonuclease; decreased transcription is observed upon fluphenazine treatment	1.713387	0.005694
DJP1	Similar to bacterial DnaJ; reported to have a role in peroxisome biogenesis; induced in low iron and upon adherence to polystyrene	1.712021	0.004529
TFG2	**Ortholog(s) have RNA polymerase II complex binding activity**	1.710939	0.000187
orf19.549	Ortholog(s) have structural constituent of ribosome activity and mitochondrial large ribosomal subunit localization	1.709821	2.01E-05
KIP3	Putative kinesin, involved in mitotic spindle organization	1.709524	0.003593
FGR44	Protein lacking an ortholog in S. cerevisiae; transposon mutation affects filamentous growth	1.708671	0.004022
SME1	Ortholog(s) have splicing factor binding activity, role in mRNA splicing, via spliceosome and U1 snRNP, U2 snRNP, U2-type prespliceosome, U4/U6 x U5 tri-snRNP complex, U5 snRNP, post-mRNA release spliceosomal complex localization	1.708627	0.013066
orf19.3919	RNI-like superfamily domain-containing protein; early-stage flow model biofilm induced; Spider biofilm induced	1.705314	0.00229
orf19.3921	**Ortholog of *S. cerevisiae* : FSH3, *C. glabrata CBS138* : CAGL0L11044g, *C. dubliniensis CD36* : Cd36_54010, *C. parapsilosis CDC317* : CPAR2_100920 and *C. auris B8441* : B9J08_004360**	1.703928	6.88E-05
ORC4	Phosphorylated protein similar to S. cerevisiae Orc4, subunit of the origin recognition complex (ORC); induced by alpha pheromone in SpiderM medium	1.703451	0.002075

PRN4	Protein with similarity to pirins; induced by benomyl treatment; flow model biofilm repressed	1.702215	0.001712
SRR1	Two-component system response regulator; involved in stress response; Plc1-regulated; upregulated in cyr1 null mutant; flow model biofilm induced; Spider biofilm induced	1.701745	0.000333
COQ10	Putative coenzyme Q (ubiquinone) binding protein; transcript is upregulated in clinical isolates from HIV+ patients with oral candidiasis	1.701232	0.001857
BUB1	Putative cell cycle checkpoint kinase; mutation confers increased sensitivity to nocodazole	1.700042	0.002056
orf19.7406	**Ortholog of *C. dubliniensis CD36* : Cd36_86220, *C. parapsilosis CDC317* : CPAR2_404490, *C. auris B8441* : B9J08_000040 and *Candida tenuis NRRL Y-1498* : CANTEDRAFT_94473**	1.698772	0.001865
orf19.3295	Has domain(s) with predicted role in cell division, chromosome segregation and MIS12/MIND type complex localization	1.697688	0.018678
YKU80	Yku70p-Yku80p Ku complex subunit involved in nonhomologous end joining during double-strand break repair repair; Hap43-repressed gene; flow model biofilm induced	1.695682	0.008454
WOR2	Zn(II)2Cys6 transcription factor; regulator of white-opaque switching; required for maintenance of opaque state; Hap43-induce	1.695617	0.001468
orf19.3581	Ortholog(s) have histone binding activity, role in DNA replication-dependent nucleosome assembly and CAF-1 complex, cytoplasm, nucleus localization (5)	1.693559	0.005757
orf19.6436	Ortholog of ***C. dubliniensis CD36* : Cd36_34210, *C. parapsilosis CDC317* : CPAR2_205590, *C. auris B8441* : B9J08_004911 and *Candida tenuis NRRL Y-1498* : cten_CGOB_00133**	1.689909	0.003584
HYR1	GPI-anchored hyphal cell wall protein; macrophage-induced; repressed by neutrophils; resistance to killing by neutrophils, azoles; regulated by Rfg1, Efg1, Nrg1, Tup1, Cyr1, Bcr1, Hap43; Spider and flow model biofilm induced	1.689427	0.008685
orf19.949	**Ortholog of *C. dubliniensis CD36* : Cd36_50420, *C. parapsilosis CDC317* : CPAR2_304100, *C. auris B8441* : B9J08_001314 and *Candida tenuis NRRL Y-1498* : CANTEDRAFT_98299**	1.687578	0.002119
SIM1	**Adhesin-like protein; involved in cell wall maintenance, redundant with Sun41; possibly**	1.685762	1.46E-05

	**secreted; macrophage-repressed; repressed by Rim101, Cyr1, Ras1; Spider biofilm induced**		
RRP9	Ribosomal protein; mutation confers resistance to 5-fluorocytosine (5-FC), 5-fluorouracil (5-FU), and tubercidin (7-deazaadenosine); physically interacts with TAP-tagged Nop1; Hap43-induced; Spider biofilm induced	1.683878	0.003624
orf19.1876	Ortholog(s) have mRNA binding activity and role in mRNA splice site selection, mRNA splicing, via spliceosome, positive regulation of mRNA splicing, via spliceosome	1.683624	0.000733
orf19.1948	**Ortholog of *C. dubliniensis CD36* : Cd36_51110, *C. parapsilosis CDC317* : CPAR2_302560, *C. auris B8441* : B9J08_005315 and *Candida tenuis NRRL Y-1498* : CANTEDRAFT_135271**	1.682107	0.007579
PMC1	Vacuolar calcium P-type ATPase; transcript regulated by calcineurin and fluconazole; mutant shows increased resistance to fluconazole, lithium; increased sensitivity to calcium; Spider biofilm induced	1.681746	0.00026
orf19.3660	**Ortholog of *C. dubliniensis CD36* : Cd36_60600, *C. parapsilosis CDC317* : CPAR2_602890, *C. auris B8441* : B9J08_001866 and *Candida tenuis NRRL Y-1498* : CANTEDRAFT_135512**	1.68173	0.010628
orf19.3289	Phosphorylated protein of unknown function	1.679811	0.002187
ELF1	Putative mRNA export protein; Walker A and B (ATP/GTP binding) motifs; required for wild-type morphology, growth; expressed in hyphal, pseudohyphal, and yeast form; Hap43-induced; Spider and flow model biofilm induced	1.678569	0.001134
PRT1	Putative translation initiation factor eIF3; mutation confers hypersensitivity to roridin A, verrucarin A; genes encoding ribosomal subunits, translation factors, tRNA synthetases are downregulated upon phagocytosis by murine macrophages	1.678513	0.000358
SEC26	Secretory vesicles coatomer complex protein	1.677932	0.000695
PTP3	Putative protein tyrosine phosphatase; hypha induced; alkaline induced; regulated by Efg1, Ras1, cAMP pathways; mutants are viable; Spider biofilm induced; rat catheter biofilm repressed; flow model biofilm repressed	1.67772	0.002822
URA7	CTP synthase 1; flucytosine induced; protein present in exponential and stationary growth phase yeast cultures	1.677681	0.000831

ZCF2	Zn(II)2Cys6 transcription factor, required for adaptation to reactive sulfur species; regulates sulfite tolerance through expression of SSU1 and CDG1; Hap43-repressed; Spider biofilm induced	1.676262	0.001962
orf19.6639	**Ortholog of S. cerevisiae Mdm36; mitochondrial distribution and morphology protein; Hap43-repressed gene**	1.674288	0.002987
CAS4	RAM cell wall integrity signaling network protein; cell separation, azole sensitivity; needed for hyphal growth; insertion mutation near 3' end of gene increases caspofungin sensitivity; pheromone/hyphal induced; flow biofilm repressed	1.673667	0.00985
orf19.7153	Putative exportin, member of the Exportin-T family; flow model biofilm repressed	1.669575	0.000588
DBP3	Putative ATP-dependent DEAD-box RNA helicase; Hap43-induced; repressed by prostaglandins; Spider biofilm induced	1.669363	0.002041
NAT1	Ortholog(s) have acetyltransferase activator activity, peptide alpha-N-acetyltransferase activity, ribosome binding activity	1.669081	0.000226
orf19.4193	Ortholog(s) have Arp2/3 complex binding activity, role in actin filament debranching, negative regulation of Arp2/3 complex-mediated actin nucleation and actin cortical patch, cytoplasm localization	1.66582	0.001362
orf19.3836	Ortholog(s) have protein-lysine N-methyltransferase activity and role in peptidyl-lysine trimethylation	1.665665	0.000813
CRH11	GPI-anchored cell wall transglycosylase, putative ortholog of S. cerevisiae Crh1p; predicted glycosyl hydrolase domain; similar to Csf4p and to antigenic A. fumigatus Aspf9; predicted Kex2p substrate; caspofungin-induced	1.665184	4.39E-05
orf19.3547	Ortholog(s) have mRNA 3'-UTR binding, mRNA 5'-UTR binding, translation repressor activity, mRNA regulatory element binding activity and role in negative regulation of translation, protein stabilization, ribosomal large subunit biogenesis	1.663243	0.000578
orf19.6754	Protein of unknown function; Spider biofilm induced	1.659891	0.000909
orf19.4634	Protein required for thiolation of uridine at wobble position of Gln, Lys, and Glu tRNAs; has a role in urmylation; S. cerevisiae ortholog has a role in invasive and pseudohyphal growth	1.659469	0.011643

AGE2	Ortholog(s) have GTPase activator activity and role in endoplasmic reticulum to Golgi vesicle-mediated transport, intra-Golgi vesicle-mediated transport	1.657876	0.011096
STP2	Amino-acid-regulated transcription factor; activates transcription of amino acid permease genes; activated by amino-acid-induced proteolytic processing (Ssy1, Csh3 dependent); required for alkalinization of medium; Spider biofilm induced	1.65748	0.009312
orf19.2019	Ortholog(s) have structural constituent of ribosome activity, role in mitochondrial translation and mitochondrial large ribosomal subunit localization	1.656748	4.87E-05
TIM50	Predicted component of the Translocase of the Inner Mitochondrial membrane (TIM23 complex); involved in mitochondrial protein import; Spider biofilm repressed	1.656738	0.001439
orf19.5442	**Ortholog of *C. dubliniensis CD36* : Cd36_80240, *C. parapsilosis CDC317* : CPAR2_504040, *C. auris B8441* : B9J08_005021 and *Candida tenuis NRRL Y-1498* : CANTEDRAFT_130872**	1.655543	0.003208
RHA1	Zinc cluster DNA-binding transcription factor; positive regulator of filamentous growth; Spider biofilm induced	1.655063	0.002428
HOF1	Protein involved in cytokinesis and DNA damage response; interacts genetically with Rad53p-dependent checkpoint; mutant is viable	1.651498	0.003726
PWP1	Putative rRNA processing protein; Hap43-induced; repressed in core stress response	1.650973	0.005368
orf19.708	Ortholog(s) have gamma-tubulin binding activity	1.650221	0.019113
orf19.5646	Ortholog of ***S. cerevisiae* : YPL034W, *C. dubliniensis CD36* : Cd36_40280, *C. parapsilosis CDC317* : CPAR2_402170, *C. auris B8441* : B9J08_003513 and *Candida tenuis NRRL Y-1498* : CANTEDRAFT_137348**	1.646915	0.003143
BRR2	Predicted RNA-dependent ATPase RNA helicase; Hap43-induced gene	1.646828	0.002018
SNF2	Protein involved in transcriptional regulation; ortholog of S. cerevisiae Snf2p, which is the catalytic subunit of the SWI/SNF chromatin remodeling complex; interacts with Swi1p; SWI/SNF complex is essential for hyphal growth and virulence	1.646197	0.001148
CHS8	Chitin synthase required for synthesis of long-chitin fibrils; nonessential; 8 or 9 membrane spanning regions; mRNA present in yeast and hyphae; induced during cell wall regeneration; flow model biofilm repressed	1.641531	0.001511

PSR1	Predicted plasma membrane associated protein phosphatase; required for normal filamentous growth; mRNA binds She3 and is localized to hyphal tips	1.6383 45	0.0073 65
PDC2	Homeodomain-like transcription factor; regulator of pyruvate decarboxylase; contains a putative C-terminal activation domain, Glu- and Pro-rich; complements glucose utilization defect of S. cerevisiae pdc2 mutant	1.6374 98	0.0045 03
orf19.425	Ortholog(s) have mitochondrial large ribosomal subunit localization	1.6371 82	0.0143 72
RLM1	Transcription factor required for wild-type resistance to cell wall perturbation caused by caspofungin treatment; regulates caspofungin-induced transcription of SKO1	1.6369 86	0.0015 69
SFU1	GATA-type transcription factor; regulator of iron-responsive genes; represses iron utilization genes if iron is present; Hap43-repressed; promotes gastrointestinal commensalism in mice; Spider biofilm induced	1.6368 01	0.0037 5
orf19.585 6	Membrane-localized protein of unknown function	1.6363 55	0.0004 53
CSF1	Ortholog(s) have role in fermentation, protein maturation	1.6340 23	0.0129 09
orf19.124 0	Ortholog of ***S. cerevisiae*** **: YPR117W,** ***C. glabrata CBS138*** **: CAGL0D04510g,** ***C. dubliniensis CD36*** **: Cd36_45200,** ***C. parapsilosis CDC317*** **: CPAR2_500480 and** ***C. auris B8441*** **: B9J08_005034**	1.6333 26	0.0091 54
POX1-3	Predicted acyl-CoA oxidase; farnesol regulated; stationary phase enriched protein; Spider biofilm induced	1.6325 78	0.0143 42
CTA1	Protein similar to S. cerevisiae Mos10p, which affects S. cerevisiae filamentous growth; activates transcription in 1-hybrid assay in S. cerevisiae; protein levels increase under weak acid stress; nonessential	1.6312 68	0.0102 44
orf19.381 7	Has domain(s) with predicted nucleic acid binding, zinc ion binding activity	1.6305 85	0.0048 35
HFI1	Ortholog(s) have transcription coactivator activity, transcription coregulator activity and role in chromatin organization, histone acetylation, transcription by RNA polymerase II	1.6290 83	0.0033 58
orf19.415 9	Ortholog(s) have magnesium ion transmembrane transporter activity, role in magnesium ion export from mitochondrion, magnesium ion transport and mitochondrial inner membrane localization	1.6276 27	0.0006 81
INN1	Protein with similarity to S. cerevisiae Inn1p, which is an essential protein of the contractile actomyosin ring required for ingression of the plasma membrane into the	1.6252 43	0.0065 79

	bud neck during cytokinesis; contains a C2 membrane targeting domain		
STH1	Putative ATP-dependent helicase, component of the RSC chromatin remodeling complex; essential gene; induced by nitric oxide	1.625025	0.017665
MEC1	Cell cycle checkpoint protein with a role in genome integrity; RNA abundance regulated by tyrosol and cell density	1.624295	0.010585
GLO3	**Putative ARF GTPase activator; role in COPI coating of Golgi vesicle, ER to Golgi vesicle-mediated transport, retrograde Golgi to ER vesicle-mediated transport; Spider biofilm repressed**	1.623585	0.000901
SLD2	Ortholog(s) have DNA replication origin binding, single-stranded DNA binding activity	1.623488	0.009107
STB2	**Ortholog of *S. cerevisiae* : STB6, *C. glabrata CBS138* : CAGL0L05016g, *C. dubliniensis CD36* : Cd36_83110, *C. parapsilosis CDC317* : CPAR2_103210 and *C. auris B8441* : B9J08_001101**	1.623437	0.013204
GCD2	Putative translation initiation factor; genes encoding ribosomal subunits, translation factors, and tRNA synthetases are downregulated upon phagocytosis by murine macrophage	1.621912	0.001384
orf19.3292	Ortholog(s) have peptide-methionine (R)-S-oxide reductase activity, role in cellular response to oxidative stress and mitochondrion localization	1.619702	0.002403
orf19.3785	DnaJ chaperone domain protein; role in pre-mRNA splicing; Spider biofilm induced (5)	1.61957	0.00957
TIM54	Predicted component of the mitochondrial TIM22 complex; involved in protein import into mitochondrial inner membrane; Spider biofilm repressed	1.618019	0.002512
orf19.3644	**Protein of unknown function; Cyr1-repressed; rat catheter and Spider biofilm induced**	1.616583	0.009407
BUD6	**Protein required for Spitzenkorper formation in hyphal cells (wild-type localization of Mlc1p to the Spitzenkorper); localizes to polarisome**	1.614957	0.005994
orf19.2418	**Ortholog of *C. dubliniensis CD36* : Cd36_28190, *C. parapsilosis CDC317* : CPAR2_802530, *Candida tenuis NRRL Y-1498* : CANTEDRAFT_109615 and *Debaryomyces hansenii CBS767* : DEHA2A12650g**	1.610934	0.010606
HRP1	**Putative cleavage factor I subunit; required for the cleavage and polyadenylation of pre-mRNA 3' ends; Spider biofilm repressed**	1.609268	4.65E-05
SAS10	Putative U3-containing small subunit processome complex subunit; Hap43p-induced gene; mutation	1.607986	0.010047

	confers resistance to 5-fluorocytosine (5-FC); repressed upon high-level peroxide stress		
SAC3	Putative nuclear pore-associated protein, required for small ribosomal subunit biogenesis; possibly an essential gene, disruptants not obtained by UAU1 method	1.606809	0.009372
orf19.1169	Putative DnaJ-like molecular chaperone; Spider biofilm induced	1.603558	0.00575
MTG2	Putative Obg family GTPase member; peripheral protein of the mitochondrial inner membrane; associates with the large ribosomal subunit; required for mitochondrial translation; rat catheter biofilm repressed	1.602853	0.019956
orf19.6326	**Ortholog of *C. dubliniensis CD36* : Cd36_60130, *C. parapsilosis CDC317* : CPAR2_603380, *C. auris B8441* : B9J08_002731 and *Candida tenuis NRRL Y-1498* : CANTEDRAFT_94402**	1.601164	0.001776
BSD2	Ortholog(s) have role in metal ion transport, protein targeting to vacuole, ubiquitin-dependent protein catabolic process and endoplasmic reticulum, fungal-type vacuole, fungal-type vacuole membrane localization	1.599625	0.005
orf19.3806	Ortholog(s) have role in negative regulation of gluconeogenesis, proteasome-mediated ubiquitin-dependent protein catabolic process, traversing start control point of mitotic cell cycle and GID complex localization	1.598768	0.020723
SET2	Ortholog(s) have histone methyltransferase activity (H3-K36 specific) activity	1.5971	0.014659
GPD2	Surface protein similar to glycerol 3-P dehydrogenase; binds host Factor H, FHL-1, plasminogen; regulated by Ssn6, Nrg1, Efg1; induced by cell wall regeneration, macrophage/pseudohyphal growth, core stress response; Spider biofilm induced	1.595414	5.64E-05
HAS1	Functional homolog of S. cerevisiae Has1p, which is a nucleolar protein of the DEAD-box ATP-dependent RNA helicase family that is involved in biogenesis of the ribosome, particularly the small (40S) subunit; caspofungin-downregulated	1.595332	0.002071
LEU1	3-isopropylmalate dehydratase; antigenic in humans; repressed in hyphae; alkaline repressed; upregulated by human whole blood or PMNs; regulated by Sef1, Sfu1, and Hap43; rat catheter biofilm induced, Spider biofilm repressed	1.595098	0.002299
PKC1	Protein kinase C; functional homolog of S. cerevisiae Pkc1p; mutant has abnormal yeast-form cell morphology and increased cell lysis; activated by phosphatidylserine;	1.59489	0.007637

	target of antifungal, cercosporamide; R400P mutant is activated		
CLA4	Ste20p family Ser/Thr kinase required for wild-type filamentous growth, organ colonization and virulence in mouse systemic infection; role in chlamydospore formation; functional homolog of S. cerevisiae Cla4p; mutant caspofungin sensitive	1.592938	0.004616
CCC1	Manganese transporter; required for normal filamentous growth; mRNA binds She3, localized to hyphal tips; repressed by NO, alkaline pH; colony morphology-related regulation by Ssn6; regulated by Sef1, Sfu1, Hap43; Spider biofilm induced	1.592622	0.001226
Predicted inositol polyphosphate kinase; Spider biofilm induced		1.591688	0.02155
orf19.1545	Ortholog(s) have structural constituent of ribosome activity and mitochondrial small ribosomal subunit localization (5)	1.590823	0.001367
orf19.439	Ortholog(s) have DNA binding, DNA strand exchange activity, single-stranded DNA binding, structural constituent of ribosome activity (4)	1.589918	0.000318
DIS3	Ortholog(s) have 3'-5'-exoribonuclease activity, endoribonuclease activity, exoribonuclease activity, ribonuclease activity, tRNA binding activity	1.589545	0.002645
VPS9	Ortholog(s) have guanyl-nucleotide exchange factor activity, ubiquitin binding activity	1.589341	0.016202
MYO2	Class V myosin; nonessential; sole class V myosin in C. albicans; required for WT actin cytoskeletal polarity, nuclear organization, migration, hyphal growth; conserved myosin ATPase/tail domains; Hap43-induced; flow model biofilm repressed	1.589171	0.0009
CDC7	Catalytic subunit of Dbf4p-regulated serine/threonine protein kinase; negative regulator of hyphal development; cell-cycle regulated periodic mRNA expression; S. cerevisiae ortholog is not cell-cycle regulated	1.587937	0.013322
orf19.4086	Has domain(s) with predicted protein tyrosine phosphatase activity (5)	1.58757	0.006386
NUF2	Kinetochore component; amount of Nuf2p and Mtw1p protein detected at each centromere is consistent with a single kinetochore microtubule attachment site	1.585996	0.010089
orf19.4830	**Ortholog of *S. cerevisiae* : YKL162C, *C. dubliniensis CD36* : Cd36_09010, *C. parapsilosis CDC317* : CPAR2_214170, *C. auris B8441* : B9J08_005393**	1.584425	0.003504

	**and *Candida tenuis NRRL Y-1498* : CANTEDRAFT_95066**		
orf19.3956	Ortholog(s) have glutaminyl-tRNA synthase (glutamine-hydrolyzing) activity and role in endoplasmic reticulum organization, glutaminyl-tRNAGln biosynthesis via transamidation	1.583687	0.002139
orf19.2057	Ortholog(s) have ATP binding, ATPase, metallopeptidase activity and role in cellular protein-containing complex assembly, proteolysis, signal peptide processing (5)	1.58309	0.001352
GYP5	Putative Rab GTPase activator; role in ER to Golgi vesicle-mediated transport; Spider biofilm induced	1.581835	0.000977
HIS3	Imidazoleglycerol-phosphate dehydratase, enzyme of histidine biosynthesis; functionally complements S. cerevisiae his3-1 mutation; hyphal-induced expression; regulated by Gcn2p and Gcn4p; fungal-specific (no human or murine homolog)	1.579093	0.001859
DNM1	Putative dynamin-related GTPase involved in mitochondrial fission; transcript upregulated inbRHE model of oral candidiasis; transcript regulated by Nrg1, Mig1, and Tup1	1.578021	0.000441
orf19.2662	Predicted ORF of unknown function; overlaps TLO34/orf19.2661	1.577777	0.002091
orf19.862	Protein of unknown function; Spider biofilm induced	1.57748	0.006507
orf19.4184	Protein with a predicted role in clathrin cage assembly; Hap43-repressed; Spider biofilm repressed	1.573729	0.017197
COG5	Ortholog(s) have role in cytoplasm to vacuole transport by the Cvt pathway, intra-Golgi vesicle-mediated transport and Golgi transport complex localization	1.572965	0.018797
orf19.295	**Ortholog of *C. dubliniensis CD36* : Cd36_82970, *C. auris B8441* : B9J08_001198, *Lodderomyces elongisporus NRLL YB-4239* : LELG_02117 and *Debaryomyces hansenii CBS767* : DEHA2G01012g**	1.572914	0.003892
orf19.281	**Ortholog of *C. dubliniensis CD36* : Cd36_82880, *C. parapsilosis CDC317* : CPAR2_102370, *Candida tenuis NRRL Y-1498* : CANTEDRAFT_118463 and *Pichia stipitis Pignal* : PICST_32241**	1.572914	0.003892
CRN1	Coronin; cortical actin cytoskeletal component; predicted role in regulation of actin patch assembly; rat catheter and Spider biofilm repressed	1.572867	0.004286
TIM44	Protein involved in transport across membranes; Spider biofilm repressed mitochondrion localization (5)	1.572352	0.000114
orf19.5782	Ortholog(s) have phospholipase activity, role in cardiolipin metabolic process, phosphatidylethanolamine	1.571883	0.002263

	metabolic process, phospholipid metabolic process and mitochondrial matrix localization		
orf19.4362	Ortholog(s) have role in protein targeting to mitochondrion and mitochondrial outer membrane, peroxisomal membrane localization	1.571724	0.007189
MSN5	Predicted karyopherin involved in nuclear import and export of proteins; flow model biofilm induced; Spider biofilm induced	1.568604	0.018476
IRE1	Protein kinase involved in regulation of unfolded protein response; role in cell wall regulation; mutant is hypersensitive to caspofungin; Spider biofilm induced	1.563192	0.011114
orf19.2415	Ortholog(s) have role in mitochondrial translation and mitochondrion localization	1.560296	0.002404
PGA1	Putative GPI-anchored protein; induced during cell wall regeneration; required for normal adhesion to host cells and for adherence during Sabouraud biofilm formation; Spider biofilm induced	1.559687	0.002919
RIM15	Ortholog(s) have protein kinase activity, protein serine/threonine kinase activity	1.55956	0.00368
orf19.4906	Putative adhesin-like protein; positively regulated by Tbf1; Spider biofilm induced	1.558139	0.005091
SAP155	Regulatory subunit of PP2A-like protein phosphatase Sit4p, involved in cell wall maintenance, regulation of hyphal growth, and virulence	1.557441	0.014243
orf19.6225.1	Ortholog(s) have role in mitochondrial cytochrome c oxidase assembly and extrinsic component of matrix side of mitochondrial inner membrane, mitochondrial matrix localization (3)	1.556369	0.008738
orf19.1335	Ortholog of S. cerevisiae Mtr4, an ATP-dependent 3'-5' RNA helicase of the DEAD-box family; Hap43-induced gene; Spider biofilm induced	1.555587	0.001711
orf19.4715	Ortholog(s) have tubulin binding activity and role in microtubule cytoskeleton organization, mitochondrial fission, mitochondrion inheritance, mitochondrion localization, nuclear migration along microtubule	1.555061	0.016248
orf19.2314	Protein with a role in nucleolar integrity and processing of pre-rRNA; mutation confers hypersensitivity to 5-fluorocytosine (5-FC), 5-fluorouracil (5-FU), and tubercidin (7-deazaadenosine); Hap43-induced; Spider biofilm induced	1.554577	0.009721
orf19.2048	Proten of unknown function; transcript positively regulated by Sfu1; Hap43 repressed; Spider biofilm induced	1.553487	0.0037

orf19.5692	Ortholog(s) have ubiquitin protein ligase activity, role in anaphase-promoting complex-dependent catabolic process, protein ubiquitination and anaphase-promoting complex, nuclear periphery localization	1.553055	0.00092
orf19.7478	Ortholog(s) have di-trans,poly-cis-decaprenylcistransferase activity, trans-hexaprenyltranstransferase activity and role in farnesyl diphosphate biosynthetic process, mevalonate pathway, ubiquinone biosynthetic process	1.552032	0.008064
orf19.341	Putative spermidine export pump; fungal-specific	1.549737	0.019187
TBP1	Transcription initiation factor; binds TATA box sequence, binding does not require TFIIA; caspofungin repressed; functional homolog of S. cerevisiae and human TATA-binding proteins; Spider biofilm induced	1.549689	0.000213
POL1	Putative DNA directed DNA polymerase alpha; RNA abundance regulated by cell cycle, tyrosol and cell density; rat catheter biofilm induced	1.548707	0.002803
MET1	Putative uroporphyrin-3 C-methyltransferase, methionine biosynthesis enzyme; upregulated by human whole blood or polymorphonuclear (PMN) cells; Hog1p-induced; fungal-specific (no human or murine homolog); Hap43p-repressed	1.548418	0.00154
NOC4	Putative nucleolar protein; Hap43-induced; mutation confers resistance to 5-fluorocytosine (5-FC), 5-fluorouracil (5-FU), and tubercidin (7-deazaadenosine); Spider biofilm induced	1.548224	0.002348
orf19.7108	D-ribulose-5-phosphate 3-epimerase; stationary phase enriched protein	1.543629	0.001848
HTA1	Histone H2A; repressed in fkh2 mutant; amphotericin B repressed; farnesol regulated; RNA abundance regulated by tyrosol and cell density; Hap43-induced gene; Spider biofilm repressed	1.542037	0.001599
SKO1	bZIP transcription factor involved in cell wall damage response; represses the yeast-to-hypha transition; mutants are caspofungin sensitive; induced by osmotic stress via Hog1; activated by Rlm1p; induced by Mnl1 under weak acid stress	1.541515	0.001392
USO6	Putative vesicular transport protein; transcript induced by filamentous growth; rat catheter biofilm repressed	1.540367	0.01175
orf19.2393	Putative transcription factor with zinc finger DNA-binding motif	1.540122	0.002236
orf19.5356	Protein with a predicted role in cell wall integrity; repressed in core stress response	1.538829	0.003267

orf19.3977	Protein with a role in translation; flow model biofilm repressed	1.53849	0.000871
SCH9	Protein kinase; involved in growth control, ribosomal protein synthesis, cell size, resistance to rapamycin,, chlamydospore formation, filamentous growth, and virulence; prevents hyphal growth in hypoxia at high CO2	1.537162	0.004022
orf19.1961	Planktonic growth-induced gene	1.536457	0.004073
PUT2	**Putative delta-1-pyrroline-5-carboxylate dehydrogenase; alkaline upregulated; protein present in exponential and stationary growth phase yeast cultures; flow model biofilm induced; Spider biofilm induced**	1.534831	0.001978
NAN1	Putative U3 snoRNP protein; Hap43p-induced gene; physically interacts with TAP-tagged Nop1p	1.534265	0.002013
orf19.760	Ortholog(s) have role in ribosome biogenesis (5)	1.532951	0.019094
orf19.5684.1	Ortholog of ***Candida tropicalis NEW ASSEMBLY*** **: CTRG1_CGOB_00063**	1.532075	0.001514
YUH2	Putative ubiquitin C-terminal hydrolase; sumoylation target	1.531893	0.001654
orf19.215	Component of a complex containing the Tor2p kinase; possible a role in regulation of cell growth; Spider biofilm induced (5)	1.530287	0.010678
FGR27	Zn(II)2Cys6 transcription factor; transposon mutation affects filamentous growth; required for yeast cell adherence to silicone substrate; Spider biofilm induced	1.528861	0.009391
orf19.7539.1	Putative adhesin-like protein; transcription detected in high-resolution tiling array experiments	1.526064	0.004442
KEX2	Subtilisin-like protease (proprotein convertase); processes aspartyl proteinase Sap2; required for hyphal growth and wild-type virulence in mice; required for maturation of candidalysin Ece1p	1.525385	0.007348
orf19.6558	Ortholog(s) have GTPase activator activity and cytosol localization	1.522871	0.001457
MDM31	Ortholog(s) have role in cellular ion homeostasis, mitochondrion inheritance, mitochondrion organization, regulation of cardiolipin metabolic process and mitochondrial inner membrane, mitochondrion localization	1.522515	0.014452
orf19.6778	Aminophospholipid translocase (flippase); maintains membrane lipid asymmetry in post-Golgi secretory vesicles; contributes to clathrin-coated vesicle formation and endocytosis; flow model biofilm repressed	1.520073	0.007444

TRM10	Ortholog(s) have tRNA (guanine(9)-N(1))-methyltransferase activity, tRNA (guanine) methyltransferase activity and role in tRNA N1-guanine methylation, tRNA methylation	1.519985	0.01341
orf19.438	Has domain(s) with predicted membrane localization (5)	1.519939	0.002496
orf19.1181	Has domain(s) with predicted catalytic activity and membrane localization	1.5196	0.003383
CDC20	Activator of anaphase-promoting complex/cyclosome; induced under Cdc5 depletion; member of conserved Mcm1 regulon; mRNA expression peaks at cell-cycle G2/M phase; mRNA binds She3 and is localized to buds of yeast cells and to hyphal tips	1.518495	0.005885
orf19.4307	Ortholog(s) have role in ESCRT III complex assembly, late endosome to vacuole transport, late endosome to vacuole transport via multivesicular body sorting pathway, protein targeting to vacuole and cytoplasm, late endosome localization	1.517364	0.005277
FLO9	Putative adhesin-like cell wall mannoprotein; repressed during the mating process; mutation confers hypersensitivity to toxic ergosterol analog; decreased transcription is observed upon fluphenazine treatment	1.517286	0.018936
PCK1	Phosphoenolpyruvate carboxykinase; glucose, C-source, yeast-hypha, Hap43 regulated; fluconazole, phagocytosis, H2O2, oral candidasis, Spider/rat catheter/flow model biofilm induced; repressed in biofilm by Bcr1, Tec1, Ndt80, Rob1, Brg1	1.517188	0.006751
DEF1	RNA polymerase II regulator; role in filamentation, epithelial cell escape, dissemination in RHE model; induced by fluconazole, high cell density; Efg1/hyphal regulated; role in adhesion, hyphal growth on solid media; Spider biofilm induced	1.515288	0.000916
orf19.4293	Ortholog(s) have role in protein maturation by iron-sulfur cluster transfer, tRNA wobble uridine modification and CIA complex, cytosol, nucleus localization	1.514712	0.005391
orf19.4021	**Ortholog of *C. dubliniensis CD36* : Cd36_54870, *C. parapsilosis CDC317* : CPAR2_100250, *Debaryomyces hansenii CBS767* : DEHA2D13992g and *Pichia stipitis Pignal* : PICST_40400**	1.514122	0.000452
orf19.3946	Ortholog(s) have role in mitochondrial cytochrome c oxidase assembly, protein insertion into mitochondrial inner membrane from matrix, protein insertion into mitochondrial membrane	1.513953	0.00317

SHA3	Putative ser/thr kinase involved in glucose transport; Tn mutation affects filamentous growth; fluconazole-induced; ketoconazole-repressed; induced in by alpha pheromone in SpiderM; possibly essential; flow model biofilm induced (1, 2, 7, 8, 9)	1.513494	0.002185
APM1	Ortholog of S. cerevisiae/S. pombe Apm1; a clathrin-associated protein complex (AP-1) subunit; phosphorylated protein; Tn mutation affects filamentous growth; Spider biofilm repressed	1.511451	0.008292
RVB1	Putative chromatin remodelling complex protein; heterozygous null mutant displays sensitivity to virgineone; Spider biofilm repressed; sumoylation regulated by Hsp90p	1.51114	0.007267
GDA1	Golgi membrane GDPase, required for wild-type O-mannosylation, not N-glycosylation; required for wild-type hyphal induction, cell wall, and cell surface charge; not required for HeLa cell adherence; functional homolog of S. cerevisiae Gda1p	1.510943	0.001543
orf19.7368	Ortholog(s) have mRNA binding, poly(U) RNA binding activity and role in nuclear-transcribed mRNA catabolic process, nonsense-mediated decay, regulation of mRNA stability, stress granule assembly (5)	1.509912	0.000503
RNH1	Putative ribonuclease H1; possibly an essential gene, disruptants not obtained by UAU1 method; flow model biofilm induced; Spider biofilm induced	1.509895	0.00445
orf19.782	Ortholog(s) have hydrolase activity, acting on ester bonds, triglyceride lipase activity, role in lipid homeostasis and lipid droplet localization (5)	1.507447	0.009316
KRE6	Essential beta-1,6-glucan synthase subunit; change in mRNA length, not abundance, at yeast-hypha transition; alkaline induced by Rim101, on cell wall regeneration; Spider biofilm induced; Bcr1-repressed in RPMI a/a biofilms	1.507379	0.000754
FUN31	Putative PAS kinase involved in cell wall damage response; similar to S. cerevisiae Psk1p, a putative serine/threonine protein kinase; induced by Mnl1 under weak acid stress; rat catheter and Spider biofilm induced	1.507092	0.007645
MNN21	Ortholog(s) have alpha-1,2-mannosyltransferase activity, role in protein glycosylation and Golgi apparatus localization	1.505308	0.013137
ARD	D-arabitol dehydrogenase, NAD-dependent (ArDH); enzyme of D-arabitol and D-arabinose catabolism; D-arabitol is a marker for active infection in humans; rat catheter and Spider biofilm induced	1.502306	0.003475

TTR1	Putative glutaredoxin; described as a glutathione reductase; induced by human neutrophils and benomyl treatment; alkaline repressed; regulated by Gcn2 and Gcn4; required for virulence in mouse model; Spider biofilm induced	1.499663	0.003355
RPN3	Putative non-ATPase regulatory subunit of the 26S proteasome lid; amphotericin B repressed; oxidative stress-induced via Cap1p	1.498792	0.001745
orf19.6155	Ortholog(s) have DNA ligase (ATP) activity, DNA ligase activity	1.498059	0.009089
BMT5	Putative beta-mannosyltransferase involved in beta-1,2-mannosylation of phospholipomannan; 9-member family includes Bmt1, Bmt2, Bmt3, and Bmtp with roles in mannosylation of cell wall phosphopeptidomannan; Spider biofilm induced (1, 5, 6)	1.497	0.011679
orf19.2259	Ortholog(s) have structural constituent of ribosome activity and mitochondrial large ribosomal subunit localization (5)	1.496526	0.000443
ABC1	Putative ubiquinol-cytochrome-c reductase; induced upon adherence to polystyrene; flow model biofilm induced; Spider biofilm induced	1.495284	0.006402
orf19.1460	Putative glutamine-dependent NAD synthetase, involved in NAD salvage pathway	1.494121	0.016143
ORC3	Protein similar to S. cerevisiae Orc3p, which is a component of the origin recognition complex involved in DNA replication; mutation confers hypersensitivity to toxic ergosterol analog; induced under hydroxyurea treatment	1.491754	0.021545
ZCF3	Zn(II)2Cys6 domain transcription factor; required for filamentous growth, resistance to rapamycin and flucytosine; possibly an essential gene, disruptants not obtained by UAU1 method; Hap43-repressed; Spider and flow model biofilm induced	1.491486	0.000455
SEC63	Endoplasmic reticulum (ER) protein-translocation complex subunit	1.490818	0.001651
DCR1	**Putative Dicer RNAse involved in RNA interference, similar to S. cerevisiae Rnt1p but orthologous to S. castellii Dcr1p, which is not conserved in S. cerevisiae**	1.486772	0.003721
orf19.7111	Putative mitochondrial outer membrane protein membrane fission effector; possibly an essential gene, disruptants not obtained by UAU1 method	1.486486	0.002128

GCD7	Putative translation initiator; downregulated in the presence of human whole blood or polymorphonuclear (PMN) cells	1.4837 85	0.0032 29
orf19.623 3	Has domain(s) with predicted oxidoreductase activity and role in metabolic process	1.4828 58	0.0041 14
MCT1	Putative malonyl-CoA acyl carrier protein acyltransferase	1.4810 57	0.0065 03
orf19.585	Ortholog(s) have structural constituent of ribosome activity and mitochondrial large ribosomal subunit localization	1.4795 09	0.0018 91
orf19.493 1	Putative tRNA-Cys synthetase; induced by alpha pheromone in SpiderM medium; ribosomal subunits, translation factors, tRNA synthetases are downregulated upon phagocytosis by murine macrophage	1.4794 69	0.0065 06
orf19.501 9	**Ortholog of *C. dubliniensis CD36* : Cd36_12800, *C. parapsilosis CDC317* : CPAR2_203460, *C. auris B8441* : B9J08_004645 and *Candida tenuis NRRL Y-1498* : CANTEDRAFT_94106**	1.4792 96	0.0033 42
DUS4	Ortholog(s) have tRNA dihydrouridine synthase activity and role in tRNA modification	1.4787 61	0.0032 53
NAB2	Ortholog(s) have 5S rRNA binding, 7S RNA binding, poly(A) binding, ribonuclease P RNA binding, tRNA binding activity	1.4787 38	0.0020 9
orf19.647 4	Protein with chitin synthesis regulation, resistance to Congo red domain; membrane-localized protein; Spider biofilm induced	1.4785 57	0.0052 52
RPA190	Putative RNA polymerase I subunit A190; Hap43p-induced gene; flucytosine induced	1.4780 47	0.0086 08
orf19.617 0	Ortholog(s) have microtubule binding, microtubule plus-end binding activity	1.4760 66	0.0194 78
orf19.687	**Ortholog of *C. dubliniensis CD36* : Cd36_62090, *C. parapsilosis CDC317* : CPAR2_602150, *C. auris B8441* : B9J08_005503 and *Candida tenuis NRRL Y-1498* : CANTEDRAFT_112751**	1.4747 57	0.0204 74
FLO8	Transcription factor; required for hyphal formation and CO2 induced white-opaque switching; regulates hyphal gene expression; required for virulence in mouse systemic infection; binds Efg1p; binds Mss11p via LisH motif; has LUFS domain	1.4747 54	0.0074 75
UTP4	Putative U3 snoRNA-associated protein; Hap43-induced; physically interacts with TAP-tagged Nop1; Spider biofilm induced	1.4715 57	0.0081 7
RPS5	Ribosomal protein S5; macrophage/pseudohyphal-induced after 16 h; downregulated upon phagocytosis by	1.4714 1	0.0002 28

	murine macrophage; Hap43-induced; Spider biofilm repressed		
HTA2	Putative histone H2A; farnesol regulated; rat catheter biofilm repressed; Spider biofilm repressed; Hap43-induced	1.471047	0.001107
SNF12	Component of the RSC chromatin remodeling complex	1.468737	0.020675
PRP46	Ortholog(s) have role in mRNA splicing, via spliceosome and Prp19 complex, spliceosomal complex localization	1.465002	0.001335
SFP1	C2H2 transcription factor involved in regulation of biofilm formation; regulates carbon source-dependent stress response; regulates response to oxidative stress; acts as repressor of START; rat catheter biofilm induced	1.463263	0.012559
orf19.5370	Ortholog(s) have fungal-type vacuole membrane localization (5)	1.462829	0.011615
orf19.6929	Ortholog(s) have acid phosphatase activity, protein tyrosine phosphatase activity, metal-dependent activity (5)	1.46143	0.000717
orf19.7662	Ortholog(s) have RNA polymerase II complex binding activity, role in DNA damage response, detection of DNA damage, mRNA 3'-end processing, negative regulation of transposition, RNA-mediated and site of double-strand break localization	1.459802	0.010658
GCR3	Functional homolog of S. cerevisiae Gcr3, which acts in regulation of glycolytic genes; no intron predicted, in contrast to intron in S. cerevisiae GCR3 gene	1.459643	0.013839
UBP6	Putative ubiquitin-specific protease of the 26S proteasome; oxidative stress-induced via Cap1p	1.459497	0.004838
orf19.3481	**Putative mitochondrial ATP-dependent RNA helicase of the DEAD-box family, transcription is activated in the presence of elevated CO2**	1.458746	0.001801
TRR1	Thioredoxin reductase; regulated by Tsa1/Tsa1B, Hap43; induced by nitric oxide, peroxide; oxidative stress-induce via Cap1; induced by human neutrophils; stationary phase enriched protein	1.458732	0.000372
orf19.5666	Ortholog(s) have chromatin binding activity	1.458104	0.007265
orf19.961.2	Has domain(s) with predicted structural constituent of ribosome activity, role in translation and ribosome localization	1.457594	0.011083
PDI1	Putative protein disulfide-isomerase; antigenic in human infection; soluble protein in hyphae; induced by filamentous growth; protein present in exponential and	1.457135	0.000606

	stationary growth phase yeast cultures; flow model biofilm repressed		
STE50	Protein with sterile alpha motif (SAM) and Ras-associated domain (RAD); similar to S. cerevisiae Rad50p, which is involved in signal transduction via interaction with and regulation of MAPKKK	1.456022	0.004393
orf19.4370	Protein of unknown function; induced by nitric oxide; oxidative stress-induced via Cap1; fungal-specific (no human or murine homolog)	1.455921	0.018531
NUO2	NADH-ubiquinone oxidoreductase subunit; Hap43p-repressed gene; repressed by nitric oxide; identified in detergent-resistant membrane fraction (possible lipid raft component)	1.455833	0.001383
orf19.3569	Protein of unknown function; S. cerevisiae YLR407W mutants have abnormal budding; constitutive expression independent of MTL or white-opaque status; Spider biofilm induced	1.455153	0.014592
CCT2	Chaperonin of the cytosolic TCP1 ring complex; protein present in exponential and stationary-phase yeast cells, but higher amounts in stationary phase; GlcNAc-induced protein	1.454177	0.001933
HSP90	Essential chaperone, regulates several signal transduction pathways and temperature-induced morphogenesis; activated by heat shock, stress; localizes to surface of hyphae, not yeast cells; mediates echinocandin and biofilm azole resistance	1.454118	0.000872
orf19.6062	Putative TIM23 translocase complex subunit; membrane-localized; Hap43-repressed	1.453509	0.010369
orf19.527	Protein of unknown function; flow model biofilm induced	1.453118	0.015005
orf19.6136	Ortholog of S. cerevisiae Mrpl4, a mitochondrial ribosomal protein of the large subunit; repressed by nitric oxide	1.451946	0.007719
WSS1	Putative metalloprotease involved in repair of DNA-protein crosslinks; interacts with SUMO (Smt3p) and Cdc48p	1.451642	0.018141
orf19.6039	Ortholog(s) have SNAP receptor activity	1.446997	0.003249
FRS2	Putative tRNA-Phe synthetase; downregulated upon phagocytosis by murine macrophage; protein present in exponential and stationary growth phase yeast cultures; Spider biofilm repressed	1.445959	0.001173
FAT1	Predicted enzyme of sphingolipid biosynthesis; upregulated in biofilm	1.445647	0.020148

orf19.891	Putative AMP deaminase; possibly an essential gene, disruptants not obtained by UAU1 method (5)	1.444509	0.018992
orf19.1617	Protein similar to S. cerevisiae Ydr282cp; transposon mutation affects filamentous growth; Hap43p-repressed gene	1.444318	0.013443
MNL1	Transcription factor; induces transcripts of stress response genes via SLE (STRE-like) elements; required for adaptation to weak acid stress; activates a subset of the genes that are repressed by Nrg1	1.443786	0.016267
CRD1	**Cardiolipin synthase; ortholog of S. cerevisiae Crd1; transcript is upregulated in clinical isolates from HIV+ patients with oral candidiasis; mutants are viable; Spider biofilm repressed**	1.443481	0.002628
ECM29	Putative scaffold protein; assists in association of the proteasome core particle with the regulatory particle; ortholog of S. cerevisiae Ecm29; transposon mutation affects filamentous growth; flow model biofilm repressed	1.441388	0.020921
DIM1	Putative 18S rRNA dimethylase; predicted role in rRNA modification and processing; Hap43-induced; likely to be essential for growth based on insertional mutagenesis strategy; F-12/CO2 early biofilm induced	1.438367	0.016367
TFA2	TFIIE small subunit; involved in RNA polymerase II transcription initiation; Spider biofilm induced	1.436893	0.007853
orf19.26	Putative peptide N-glycanase; induced by nitric oxide independent of Yhb1p (5)	1.436124	0.022168
BUD22	Protein with a predicted role in 18S rRNA maturation and small ribosomal subunit biogenesis; repressed in core stress response; repressed by prostaglandins	1.435523	0.020432
orf19.970	Protein with a predicted role in microtubule-related processes; Spider biofilm induced	1.432483	0.011461
CTA26	Putative transcription factor/activator; Med2 mediator complex ddomain; transcript is upregulated in an RHE model of oral candidiasis; member of a family of telomere-proximal genes; Efg1, Hap43-repressed	1.428734	0.007633
ZCF39	Zn(II)2Cys6 transcription factor; mutants are viable; filament induced; required for yeast cell adherence to silicone substrate; Spider biofilm induced	1.425351	0.01844
TLO11	Member of a family of telomere-proximal genes of unknown function; may be spliced in vivo	1.424919	0.006018
RPN8	Putative regulatory subunit of the 26S proteasome; mutation confers hypersensitivity to amphotericin B; regulated by Mig1, Gcn2 and Gcn4; Spider biofilm repressed	1.42244	0.002369

RPL40B	Protein with similarity to the ribosomal protein portion of S. cerevisiae Rpl40Bp; colony morphology-related gene regulation by Ssn6; positively regulated by Tbf1; Spider biofilm repressed	1.420104	0.010626
MAP1	Ortholog(s) have mRNA binding, metalloaminopeptidase activity and role in negative regulation of gene expression, protein initiator methionine removal involved in protein maturation	1.419784	0.002997
RLI1	Member of RNase L inhibitor (RLI) subfamily of ABC family; predicted not to be a transporter; regulated by Sef1p, Sfu1p, and Hap43p	1.418944	0.003929
CAR1	Arginase; arginine catabolism; transcript regulated by Nrg1, Mig1, Tup1; colony morphology-related regulation by Ssn6; alkaline induced; protein decreased in stationary phase; sumoylation target; flow model biofilm induced	1.417255	0.004111
orf19.4952	Protein of unknown function; flow model biofilm induced; Spider biofilm induced (5, 6)	1.417003	0.011146
MCM3	Putative DNA replication protein; periodic mRNA expression, peak at cell-cycle M/G1 phase; Spider biofilm induced	1.414148	0.013137
RRB1	Ortholog(s) have unfolded protein binding activity, role in ribosome biogenesis and nucleolus localization	1.412163	0.007094
orf19.4857	Protein with a dual-specificity phosphatase domain; Hap43-induced gene	1.41198	0.021062
YHM1	Putative mitochondrial carrier protein; fungal-specific (no human or murine homolog); Hap43p-repressed gene	1.407932	0.001757
orf19.1516	Ortholog(s) have thiol-dependent deubiquitinase activity and role in negative regulation of gluconeogenesis, proteasome-mediated ubiquitin-dependent protein catabolic process (5)	1.407889	0.019697
CPR6	Putative peptidyl-prolyl cis-trans isomerase; macrophage/pseudohyphal-repressed; heavy metal (cadmium) stress-induced; heterozygous null mutant displays sensitivity to virgineone; rat catheter biofilm induced	1.403817	0.01236
VPS51	Protein with a role in vacuolar function; null mutant has defect in damaging oral epithelial and vascular endothelial cells; required for normal hyphal growth and stress resistance; induced in presence of host oral or vascular cells	1.4026	0.017651
orf19.2726	Putative plasma membrane protein; Plc1-regulated; Spider biofilm induced	1.399861	0.004282
orf19.1388	**Putative 66S pre-ribosomal particle component; Hap43-induced; F-12/CO2 early biofilm induced**	1.39696	0.007948

GGA2	Protein involved in Golgi trafficking; rat catheter and Spider biofilm repressed	1.396914	0.009596
LSO2	Similar to HMG-box variant of S. pombe; Spider biofilm repressed	1.393285	0.018321
MRP17	Predicted mitochondrial ribosomal protein	1.393103	0.014836
orf19.6565	Conserved mitochondrial inner membrane insertase; mediates insertion of mitochondrial- and nuclear-encoded proteins from the matrix into the inner membrane; Spider biofilm repressed	1.391069	0.002932
SEC13	Putative protein transport factor; antigenic during murine systemic infection; macrophage-downregulated protein; protein level decreases in stationary phase cultures; Spider biofilm repressed	1.38976	0.005761
YAH1	Protein similar to oxidoreductases; induced in high iron; Hap43-repressed	1.388498	0.018362
SHP1	**Regulator of the type 1 protein phosphatase Glc7p activity, involved in control of morphogenesis, progression through the cell cycle and response to DNA damage**	1.382976	0.016226
orf19.3007.2	Ortholog of C. dubliniensis CD36 : Cd36_02950, C. parapsilosis CDC317 : CPAR2_108520, C. auris B8441 : B9J08_002035 and Candida tenuis NRRL Y-1498 : CANTEDRAFT_100285 (3)	1.381736	0.004337
orf19.2104	Ortholog(s) have ATPase activator activity, chaperone binding activity (5)	1.37819	0.011128
CUE1	Ortholog(s) have ubiquitin binding, ubiquitin-protein transferase activator activity and role in establishment of protein localization to endoplasmic reticulum membrane, ubiquitin-dependent ERAD pathway	1.375816	0.014373
FGR32	Protein similar to S. cerevisiae Swa2p; induced upon adherence to polystyrene; transposon mutation affects filamentous growth; Hap43p-repressed gene	1.373121	0.017629
orf19.3428	Protein of unknown function; flow model biofilm induced	1.367202	0.01168
SMY2	Ortholog(s) have role in endoplasmic reticulum to Golgi vesicle-mediated transport and endoplasmic reticulum membrane, extrinsic component of membrane localization	1.367131	0.005384
RMT2	Minor protein arginine methyltransferases (PRMT) involved in methylation of arginine residues	1.365837	0.01057
NFU1	Protein with a predicted role in mitochondrial iron metabolism; Hap43-repressed; expression upregulated	1.363328	0.008845

	during growth in the mouse cecum; Spider biofilm induced		
PTP1	Phosphotyrosine-specific protein phosphatase; rat catheter biofilm induced	1.363066	0.020304
orf19.2214	Ortholog(s) have structural constituent of ribosome activity and mitochondrial large ribosomal subunit localization (5)	1.360805	0.001256
FRS1	Phenylalanyl-tRNA synthetase; possible role in early cell wall biosynthesis; downregulated by phagocytosis by macrophages; possibly essential gene, disruptants not obtained by UAU1 method; protein present in exponential and stationary phase	1.359721	0.00332
TLO8	Member of a family of telomere-proximal genes of unknown function; may be spliced in vivo	1.359275	0.013605
orf19.5833	Ortholog(s) have SUMO binding, polyubiquitin modification-dependent protein binding, ubiquitin binding activity (5)	1.357935	0.01021
UBA1	Ubiquitin-activating enzyme; protein level decreases in stationary phase cultures	1.35674	0.015455
CUE5	Predicted ubiquitin-binding protein; rat catheter biofilm repressed	1.354734	0.007576
orf19.3900	Ortholog(s) have role in mitochondrial fusion and integral component of mitochondrial outer membrane localization	1.354151	0.005003
PAM17	Predicted component of the presequence translocase-associated import motor (PAM complex) involved in protein import into mitochondrial matrix	1.352415	0.003433
orf19.1970	Putative vacuole biogenesis protein	1.350666	0.009117
PGA62	Adhesin-like cell wall protein; putative GPI-anchor; fluconazole-induced; induced in high iron; induced during cell wall regeneration; Cyr1 or Ras1 repressed; Tbf1 induced	1.350029	0.003325
orf19.2866	Has domain(s) with predicted DNA binding, nucleic acid binding activity	1.349824	0.017242
TIM22	**Mitochondrial inner membrane protein; predicted role in protein import; Hap43-repressed gene; flow model biofilm induced; Spider biofilm repressed**	1.349304	0.004204
orf19.4846	GlcNAc-induced protein	1.347742	0.004315
orf19.1967	Ortholog(s) have structural constituent of ribosome activity and mitochondrial large ribosomal subunit localization	1.345204	0.0121

orf19.2653	Protein with a transient receptor potential (TRP) ion channel domain; mutants are viable; rat catheter and Spider biofilm induced; flow model biofilm repressed	1.344887	0.011839
orf19.5213	Putative protein of unknown function; transcript is upregulated in clinical isolates from HIV+ patients with oral candidiasis	1.344767	0.016598
RRP7	Ortholog(s) have rRNA binding activity, role in rRNA processing, ribosomal small subunit assembly and CURI complex, UTP-C complex localization	1.340608	0.008164
MRPL27	Putative 60S ribosomal protein L27, mitochondrial precursor	1.338595	0.012524
orf19.5616	**Ortholog of *C. dubliniensis CD36* : Cd36_63740, *C. parapsilosis CDC317* : CPAR2_503850, *Pichia stipitis Pignal* : PICST_32045 and *Candida guilliermondii ATCC 6260* : PGUG_00070**	1.336397	0.003718
CAF130	Ortholog(s) have role in positive regulation of transcription elongation from RNA polymerase II promoter and CCR4-NOT core complex localization	1.334054	0.012296
SAC7	Putative GTPase activating protein (GAP) for Rho1; repressed upon adherence to polystyrene; macrophage/pseudohyphal-repressed; transcript is upregulated in RHE model of oral candidiasis and in clinical oral candidiasis	1.32917	0.004058
TLO9	Member of a family of telomere-proximal genes of unknown function; Hap43p-repressed gene	1.32706	0.008772
orf19.2487	Protein with a predicted phosphoribulokinase/uridine kinase domain; Spider biofilm induced (5)	1.326124	0.011977
DEM1	Putative mitochondrial exonuclease; alpha-factor induced	1.325462	0.017038
KAR2	Similar to Hsp70 family chaperones; role in translocation of proteins into the ER; induced in high iron; protein present in exponential and stationary growth phase yeast cultures; flow model and Spider biofilm repressed	1.323257	0.004651
MET10	Sulfite reductase; role in sulfur amino acid metabolism; induced by human whole blood or PMNs; Hog1-induced; possibly adherence-induced; flow model, Spider model, F-12/CO2 biofilm induced	1.321716	0.020256
orf19.5694	Predicted ORF from Assembly 19; removed from Assembly 20; restored based on transcription data	1.321637	0.004075
RPL12A	**Ribosomal protein L12, 60S ribosomal subunit; downregulated by human whole blood or polymorphonuclear cells; genes encoding cytoplasmic ribosomal subunits are downregulated**	1.321129	0.00346

	**upon phagocytosis by macrophage; Tbf1p-activated; Hap43p-induced**		
EIP1	**Probable securin that interacts with and regulates cohesin protease (separase) Esp1p to ensure chromatid separation during anaphase; Spider biofilm repressed**	1.3209 35	0.0179 23
RIB5	Putative riboflavin synthase; fungal-specific (no human or murine homolog); farnesol-downregulated; protein present in exponential and stationary growth phase yeast cultures	1.3152 83	0.0073 77
HXT5	Putative sugar transporter; induced by ciclopirox olamine; Snf3-induced; alkaline repressed; colony morphology-related gene regulation by Ssn6; possibly essential gene	1.3119 15	0.0110 09
VPS4	AAA-ATPase involved in transport from MVB to the vacuole and ESCRT-III complex disassembly; mutation decreases SAP secretion and virulence in murine intravenous infection; regulated by Gcn2p, Gcn4p; required for normal Rim8p processing	1.3109 3	0.0123 88
HWP1	Hyphal cell wall protein; host transglutaminase substrate; opaque-, a-specific, alpha-factor induced; at MTLa side of conjugation tube; virulence complicated by URA3 effects; Bcr1-repressed in RPMI a/a biofilms; Spider biofilm induced	1.3095 76	0.0061 02
orf19.470 5	Ortholog(s) have ATP binding, ATP:3'-cytidine-cytidine-tRNA adenylyltransferase activity, CTP:3'-cytidine-tRNA cytidylyltransferase activity, tRNA adenylyltransferase activity, tRNA binding activity	1.3092 97	0.0217 67
GCD1	Putative translation initiation factor; transcript regulated by Mig1; repressed upon phagocytosis by murine macrophage	1.3033 55	0.0183 22
COQ5	Putative methyltransferase of ubiquinone biosynthesis; regulated by Gcn4; repressed by amino acid starvation (3-AT), Hap43; induced upon adherence to polystyrene; Spider biofilm repressed	1.3033 23	0.0140 84
orf19.618 6	Predicted protein of unknown function; overlaps orf19.6185	1.2984 12	0.0093 48
ARO4	3-deoxy-D-arabinoheptulosonate-7-phosphate synthase; aromatic amino acid biosynthesis; GCN-regulated; feedback-inhibited by tyrosine if produced in S. cerevisiae Aro3p and Aro4p catalyze same reaction; protein decreases in stationary phase	1.2976 31	0.0100 23

SAM35	Predicted component of the sorting and assembly machinery (SAM complex) of the mitochondrial outer membrane, involved in protein import into mitochondria	1.294213	0.009134
SES1	Seryl-tRNA synthetase; charges the tRNA that recognizes the CUG codon, which typically specifies Leu, but specifies Ser in C. albicans; complements S. cerevisiae ses1 mutant viability; soluble protein in hyphae; macrophage-regulated	1.293862	0.006646
RPT5	26S proteasome regulatory subunit; transcript regulated by Nrg1 and Mig1; regulated by Gcn2 and Gcn4; protein level decreases in stationary phase	1.291123	0.00896
orf19.268	Ortholog(s) have role in DNA repair, transcription-coupled nucleotide-excision repair and Ddb1-Ckn1 complex localization	1.290259	0.021616
DED81	Putative tRNA-Asn synthetase; genes encoding ribosomal subunits, translation factors, tRNA synthetases are downregulated upon phagocytosis by murine macrophage; protein enriched in stationary phase yeast cultures	1.287066	0.007845
orf19.6406	Ortholog(s) have structural constituent of ribosome activity and mitochondrial small ribosomal subunit localization	1.286114	0.010729
orf19.3130	Ortholog of S. cerevisiae Gpn3 a GTPase with a role in biogenesis of RNA pol II and polIII; possibly an essential gene, disruptants not obtained by UAU1 method	1.285352	0.007342
PIN3	Putative SH3-domain-containing protein; predicted role in actin cytoskeleton organization; Spider biofilm repressed	1.281857	0.005001
orf19.3797	Ortholog(s) have structural constituent of ribosome activity and mitochondrial large ribosomal subunit localization	1.280279	0.007084
orf19.3535	Ortholog(s) have cellular bud neck, fungal-type vacuole localization (5)	1.278747	0.005063
orf19.2343.1	Putative vacuolar H+ ATPase subunit e of the V-ATPase V0 subcomplex; added to Assembly 21 based on comparative genome analysis	1.272694	0.021212
orf19.5518	Protein of unknown function; Spider biofilm induced	1.271188	0.014721
RRN3	Protein with a predicted role in recruitment of RNA polymerase I to rDNA; caspofungin induced; flucytosine repressed; repressed in core stress response; repressed by prostaglandins	1.269011	0.014815
SDH2	Succinate dehydrogenase, Fe-S subunit; localizes to surface of yeast cells, but not hyphae; induced in high	1.263071	0.012843

	iron and during log phase aerobic growth; repressed by nitric oxide, Hap43		
YME1	Ortholog(s) have ATP-dependent peptidase activity	1.261881	0.016593
PGA4	GPI-anchored cell surface protein; beta-1,3-glucanosyltransferase with similarity to the A. fumigatus GEL family; transcript induced in RHE model of oral candidiasis; fluconazol-induced	1.25618	0.014457
NAM7	Putative role in nonsense-mediated mRNA decay; similar to S. cerevisiae Nam7p; gene induced by ciclopirox olamine treatment	1.248555	0.013008
PST3	Flavodoxin-like protein involved in oxidative stress protection and virulence; YNB biofilm induced; stationary phase enriched protein; rat catheter and Spider biofilm repressed	1.248296	0.015375
GLN4	Putative tRNA-Gln synthetase; genes encoding ribosomal subunits, translation factors, tRNA synthetases are downregulated upon phagocytosis by murine macrophage; protein present in exponential and stationary growth phase yeast cultures	1.238015	0.014552
orf19.7386	Ortholog(s) have mitochondrial ribosome binding activity and role in inner mitochondrial membrane organization, positive regulation of mitochondrial translation, protein insertion into mitochondrial inner membrane from matrix (5)	1.214124	0.019381
orf19.2275	Putative mitochondrial ribosomal protein; predicted role in aerobic respiration; Spider biofilm repressed (5)	1.210557	0.020619
GTT11	Glutathione S-transferase, localized to ER; induced in exponentially growing cells, under oxidative stress; induced by nitric oxide; Spider biofilm induced	1.197067	0.017665
UTR2	Putative GPI anchored cell wall glycosidase; role in adhesion, hyphal growth on Spider (not serum) medium; chitin-binding, glycosyl hydrolase domains; induced during cell wall regeneration; mRNA in yeast-form cells; Spider biofilm induced	1.193975	0.015954
YCK2	Plasma membrane protein similar to S. cerevisiae casein kinase I; mutation or inhibition impairs virulence and morphogenesis; transcription is activated in weak acid stress or on contact with with host cells	1.18649	0.019505
CYC1	Cytochrome c; complements defects of S. cerevisiae cyc1 cyc7 double mutant; induced in high iron; alkaline repressed; repressed by nitric oxide; Hap43-dependent repression in low iron; regulated by Sef1, Sfu1	1.176204	0.019104

orf19.4959	**Protein of unknown function; Spider biofilm repressed**	1.170553	0.019792
PIL1	Eisosome component; predicted role in endocytosis; echinocandin-binding protein; localizes to cell surface of hyphae, but not yeast-form cells; Hap43, YNB biofilm induced; rat catheter biofilm repressed	-1.00118	5.82E-08
LTP1	Putative protein phosphatase of the PTP family (tyrosine-specific), similar to S. cerevisiae Ltp1p	-1.00259	1.86E-08
FEN1	Putative fatty acid elongase; predicted role in sphingolipid biosynthesis; possibly an essential gene, disruptants not obtained by UAU1 method; Spider and flow model biofilm induced	-1.00355	7.46E-08
PTP2	Predicted protein tyrosine phosphatase; involved in regulation of MAP kinase Hog1 activity; induced by Mnl1 under weak acid stress; rat catheter and Spider biofilm induced	-1.00506	4.92E-05
orf19.787.1	Protein of unknown function; ORF added to Assembly 21 based on comparative genome analysis; protein detected by mass spec in stationary phase cultures	-1.0059	0.001615
orf19.4269	Has domain(s) with predicted ATP binding, protein kinase activity and role in protein phosphorylation	-1.00595	1.95E-05
HYR4	Putative GPI-anchored adhesin-like protein; Rim101-repressed; constitutive expression independent of MTL or white-opaque status	-1.00659	0.000268
GVP36	BAR domain protein; ocalizes to early and late Golgi vesicles; predicted role in adaptation to varying nutrient concentrations, fluid-phase endocytosis, actin cytoskeleton polarization and vacuole biogenesis; rat catheter biofilm repressed	-1.00679	2.2E-06
LAC1	Ceramide synthase; required for biosynthesis of ceramides with C18:0 fatty acids, which serve as precursors for glucosylsphingolipids; caspofungin induced	-1.00903	2.14E-05
orf19.7288	Protein with predicted oxidoreductase and dehydrogenase domains; Hap43-repressed; Spider biofilm induced	-1.00908	0.0002
orf19.4341	Ortholog(s) have role in attachment of GPI anchor to protein and GPI-anchor transamidase complex localization	-1.00971	3.14E-07
orf19.1070	Ortholog(s) have role in phospholipid translocation, retrograde transport, endosome to Golgi and endosome membrane, trans-Golgi network membrane localization	-1.01209	0.00018

orf19.6537	Ortholog(s) have role in chromosome segregation and kinetochore, nuclear MIS12/MIND complex, spindle pole localization	-1.01356	0.020411
orf19.7210	Protein of unknown function; Spider biofilm induced	-1.01391	0.000135
FCA1	Cytosine deaminase; enzyme of pyrimidine salvage; functional homolog of S. cerevisiae Fcy1p; mutation is associated with resistance to flucytosine (5-FC) in a clinical isolate; hyphal downregulated; gene has intron	-1.01518	4.65E-06
CAR2	Ornithine aminotransferase; arginine metabolism; alkaline induced; mutant sensitivite to toxic ergosterol analog, to amphotericin B; exponential and stationary phase yeast; flow model biofilm induced; rat catheter, Spider biofilm repressed	-1.01646	2.77E-06
CDC3	Septin; essential for viability; functional homolog of S. cerevisiae Cdc3p; down-regulation associated with azole resistance; macrophage/pseudohyphal-repressed; virulence-group-correlated expression; gene has intron	-1.01798	3.58E-07
orf19.518	Ortholog(s) have tRNA (cytosine-5-)-methyltransferase activity, tRNA binding activity	-1.01975	1.39E-05
orf19.5573	Protein of unknown function; expression downregulated in an ssr1 null mutant	-1.02029	0.010023
orf19.5157	Protein with a protein tyrosine phosphatase-like protein domain; putative membrane-spanning regions; rat catheter biofilm induced	-1.02212	0.001252
RTF1	Putative RNA polymerase II-associated Paf1 complex subunit; induced during the mating process	-1.02224	0.001515
GNA1	Glucosamine-6-phosphate acetyltransferase; enzyme of UDP-GlcNAc biosynthesis; required for viability in absence of GlcNAc supplementation; required for persistent infection and wild-type virulence in mouse systemic infection	-1.02331	0.000463
LAP41	Putative aminopeptidase yscI precursor; mutant is viable; protein present in exponential and stationary growth phase yeast cultures; Spider biofilm repressed	-1.02353	2.98E-07
orf19.4520	Putative gluconokinase; rat catheter biofilm induced	-1.02369	0.017715

orf19.3601	Has domain(s) with predicted ATP binding, ATPase, nucleoside-triphosphatase activity, nucleotide binding activity	-1.0259	0.001897
ADE4	Putative phosphoribosylpyrophosphate amidotransferase; flucytosine induced	-1.02799	1.75E-07
orf19.557	Protein of unknown function; Hap43-repressed; Spider biofilm repressed	-1.03536	0.002612
DAP1	Similar to mammalian membrane-associated progesterone receptors involved in DNA damage response; induced in core stress response; Hog1 regulated; clade-associated expression; Hap43-repressed	-1.03641	1.29E-06
orf19.6581	Ortholog(s) have palmitoyltransferase activity, role in protein palmitoylation and endoplasmic reticulum localization	-1.03735	2.19E-05
TIF1	Translation initiation factor; upregulated in highly virulent strain compared to less virulent strain; antigenic in human; flucytosine induced; downregulated upon phagocytosis by macrophages; Spider biofilm repressed	-1.03757	8.18E-06
orf19.3103	Ortholog(s) have RNA polymerase III activity, role in tRNA transcription by RNA polymerase III and RNA polymerase III complex localization	-1.03945	0.001004
HGH1	Putative HMG1/2-related protein; transcript regulated by Mig1	-1.04084	1.12E-07
PGK1	Phosphoglycerate kinase; localizes to cell wall and cytoplasm; antigenic in murine/human infection; flow model biofilm, Hog1-, Hap43-, GCN-induced; repressed upon phagocytosis; repressed in Spider biofilms by Bcr1, Ndt80, Rob1, Brg1	-1.04118	6.58E-07
PHR1	Cell surface glycosidase; may act on cell-wall beta-1,3-glucan prior to beta-1,6-glucan linkage; role in systemic, not vaginal virulence (neutral, not low pH); high pH or filamentation induced; Bcr1-repressed in RPMI a/a biofilm	-1.04165	7.27E-06
orf19.5041	Ortholog of C. dubliniensis CD36 : Cd36_43600, C. parapsilosis CDC317 : CPAR2_403780, Candida tenuis NRRL Y-1498 : CANTEDRAFT_93767 and Debaryomyces hansenii CBS767 : DEHA2G16984g	-1.04393	0.001059
orf19.5727	Ortholog of C. dubliniensis CD36 : Cd36_64130, C. parapsilosis CDC317 : CPAR2_601230, C. auris B8441 : B9J08_003898 and Candida tenuis NRRL Y-1498 : CANTEDRAFT_115908	-1.04395	1.82E-05

PMA1	Plasma membrane H(+)-ATPase; highly expressed, comprises 20-40% of total plasma membrane protein; levels increase at stationary phase transition; fluconazole induced; caspofungin repressed; upregulated in RHE model; Spider biofilm repressed	-1.04746	0.000161
orf19.3763	Ortholog of C. dubliniensis CD36 : Cd36_11710, C. auris B8441 : B9J08_003469, Candida tenuis NRRL Y-1498 : CANTEDRAFT_112512 and Debaryomyces hansenii CBS767 : DEHA2E16808g	-1.04786	0.000544
ERO1	Ortholog of S. cerevisiae Ero1; role in formation of disulfide bonds in the endoplasmic reticulum; fluconazole-induced; induced by Mnl1 under weak acid stress; Spider biofilm induced	-1.04904	1.91E-07
SNL1	Ribosome-associated protein predicted to function in protein synthesis; 1 predicted transmembrane domain; rat catheter biofilm repressed	-1.04936	1.43E-06
MET15	O-acetylhomoserine O-acetylserine sulfhydrylase; sulfur amino acid synthesis; immunogenic; Hog1, adherence-induced; brown color of mutant in Pb(2+) medium a visual selection; chlamydospore formation induced, F-12/CO2 biofilm induced	-1.05147	1.25E-07
orf19.7271	Protein of unknown function; transcript detected on high-resolution tiling arrays	-1.05259	1.07E-05
SER33	Predicted enzyme of amino acid biosynthesis; Gcn4p-regulated; upregulated in biofilm; protein present in exponential and stationary growth phase yeast cultures; S. cerevisiae ortholog is Gcn4p regulated	-1.05457	5.66E-07
orf19.3442	Putative oxidoreductase; Hap43-repressed gene	-1.05477	2.59E-08
orf19.4516	Ortholog(s) have tetrahydrofolylpolyglutamate synthase activity, role in one-carbon metabolic process and cytoplasm, mitochondrion localization (5)	-1.05865	0.000228
orf19.4629	Ortholog(s) have ubiquitin-ubiquitin ligase activity, role in ATP export, mitochondrion inheritance, protein monoubiquitination, protein polyubiquitination, ubiquitin-dependent endocytosis and ubiquitin ligase complex localizatio	-1.05978	0.01393
orf19.4521	S. cerevisiae ortholog Env9 has similarity to oxidoreductases and is proposed to have vacuolar functions, found in lipid particles; hyphal-induced expression	-1.06186	6.76E-05

UGA4	Putative gamma-aminobutyric acid/polyamine permease; nitrogen catabolite repressed gene, induced in absence of preferred N sources; transcriptionally induced upon phagocytosis by macrophage; gene regulation by nitrogen source requires Gat1p	-1.06405	0.011835
orf19.1239	Secreted protein; exogenously expressed protein is a substrate for Kex2 processing in vitro; fluconazole-regulated; Spider biofilm induced	-1.06457	0.010633
orf19.321	Ortholog(s) have L-methionine transmembrane transporter activity and role in methionine import across plasma membrane	-1.06462	3.33E-06
GTR1	Putative GTP-binding protein; involved in activation of TOR1C during starvation response; transcript is upregulated in clinical isolates from HIV+ patients with oral candidiasis; (see Locus History Note for Assembly 19 correction)	-1.06485	0.004085
orf19.4621	Ortholog(s) have P-body localization	-1.06592	0.000341
orf19.2269	Putative 3-phosphoserine phosphatase; induced by benomyl or in azole-resistant strain that overexpresses MDR1; early-stage flow model biofilm induced; Spider biofilm repressed	-1.06691	1.69E-07
ABG1	Vacuolar membrane protein; depletion causes abnormal vacuolar morphology, cell separation defect, sensitivity to cell wall stress, increased hyphal branching; essential, no mammalian homolog; Cyr1-regulated; rat catheter biofilm repressed	-1.06828	4.37E-08
ERG2	C-8 sterol isomerase; enzyme of ergosterol biosynthesis; converts fecosterol to episterol; mutant is hypersensitive to multiple drugs; ketoconazole-induced; flow model and Spider biofilm repressed	-1.06832	8.31E-08
RPS3	Ribosomal protein S3; Hog1, Hap43-induced; grepressed upon phagocytosis by murine macrophage; present in exponential and stationary phase cells; Spider biofilm repressed	-1.07009	6.24E-10
orf19.7149	Putative GTPase inhibitor; predicted role in endocytosis, protein targeting to vacuole; rat catheter biofilm repressed	-1.07013	3.51E-06
CBR1	Putative cytochrome B5 reductase; plasma membrane-localized	-1.07329	3.66E-09

orf19.7589	Protein of unknown function; Hap43-repressed gene; transcript induced by elevated CO2	-1.07523	3.04E-07
orf19.7670	Putative Ca2+/H+ antiporter; oral infection upregulated gene; mutants have reduced capacity to damage oral epithelial cells	-1.07792	2.63E-07
CST5	Scaffold protein for the mitogen-activated protein (MAP) kinase cascade that regulates mating; required for opaque mating or white biofilm formation in response to mating pheromone; induced in response to pheromone; Hap43p-repressed	-1.0784	8.23E-06
GOR1	Ortholog(s) have glyoxylate reductase (NAD+) activity, role in glyoxylate catabolic process and extracellular region localization	-1.08139	1.12E-06
ALD6	Putative aldehyde dehydrogenase; stationary phase enriched protein; expression regulated upon white-opaque switch; rat catheter biofilm induced; rat catheter and Spider biofilm induced	-1.0825	0.000961
orf19.499	Ortholog(s) have S-adenosylmethionine-dependent methyltransferase activity and role in translational readthrough	-1.08545	0.000112
PCL5	Putative cyclin for Pho85 kinase; Gcn4-induced; suppresses toxicity of C. albicans Gcn4 overproduction in S. cerevisiae via increased Pho85-dependent phosphorylation and degradation of Gcn4; rat catheter and Spider biofilm induced	-1.08548	6.15E-05
orf19.6604	Ortholog of S. cerevisiae Pba1 that is involved in 20S proteasome assembly; upregulated in a cyr1 null mutant; contains a 5' UTR intron	-1.08612	5.65E-09
ASG1	Gal4p family zinc-finger transcription factor with similarity to S. cerevisiae Asg1p	-1.08738	7.95E-06
orf19.6453	Ortholog of C. dubliniensis CD36 : Cd36_72300, C. parapsilosis CDC317 : CPAR2_703060, C. auris B8441 : B9J08_001030 and Candida tenuis NRRL Y-1498 : CANTEDRAFT_92002	-1.08769	0.003514
orf19.746	Has domain(s) with predicted role in protein transport, vacuolar transport	-1.08944	8.12E-05
orf19.1350	Protein with a thioredoxin domain; predicted role in cell redox homeostasis; rat catheter and Spider biofilm induced	-1.08993	0.001573

orf19.4864	Ortholog(s) have acylglycerol lipase activity, role in triglyceride metabolic process and lipid droplet, membrane localization	-1.09276	6.43E-07
orf19.1305	Ortholog(s) have tRNA (guanine-N1-)-methyltransferase activity, role in mitochondrial tRNA methylation, tRNA N1-guanine methylation and cytoplasm, mitochondrial matrix localization	-1.09622	8.39E-07
orf19.7095	Ortholog(s) have fluoride transmembrane transporter activity, role in cellular detoxification of fluoride, fluoride export across plasma membrane, fluoride transmembrane transport and plasma membrane localization	-1.0963	1.08E-07
orf19.4174	Ortholog(s) have nicotinamide riboside transmembrane transporter activity, nucleobase transmembrane transporter activity, nucleoside transmembrane transporter activity	-1.097	3.18E-08
TPS1	Trehalose-6-phosphate synthase; role in hyphal growth and virulence in mouse systemic infection; induced in presence of human neutrophils; macrophage/pseudohyphal-repressed after 16h; stationary phase enriched protein; Hap43-repressed	-1.1003	3.66E-06
ZFU2	Zn(II)2Cys6 transcription factor; regulator of yeast form adherence; mutants display increased colonization of mouse kidneys; required for yeast cell adherence to silicone substrate; Spider biofilm induced	-1.10086	9.47E-05
orf19.729	Adaptor protein required for specific mRNA transport; protein similar but not orthologous to S. cerevisiae She3; transposon mutation affects filamentous growth	-1.10227	5.62E-05
TIM9	Predicted protein of the mitochondrial intermembrane space; rat catheter biofilm induced; Spider biofilm repressed	-1.10481	0.003194
	Has domain(s) with predicted DNA binding activity, role in DNA recombination, DNA repair, DNA replication and nucleus localization	-1.10558	7.77E-07
orf19.5523	Ortholog of C. dubliniensis CD36 : Cd36_62780, C. parapsilosis CDC317 : CPAR2_601690, C. auris B8441 : B9J08_001638 and Candida tenuis NRRL Y-1498 : CANTEDRAFT_113271	-1.10659	9.86E-05
SSU72	Ortholog(s) have RNA polymerase II CTD heptapeptide repeat phosphatase activity, phosphoprotein phosphatase activity, protein serine/threonine phosphatase activity, protein tyrosine phosphatase activity	-1.11372	0.000052

APL2	Phosphorylated protein of unknown function; mutation confers hypersensitivity to toxic ergosterol analog	-1.11487	1.06E-08
DUG3	Putative glutamine amidotransferase (GATase II); role in glutathione catabolism;	-1.11564	9.13E-07
orf19.5054	Putative quinolinate phosphoribosyl transferase, involved in NAD biosynthesis; Hap43p-repressed gene	-1.11763	9.45E-10
orf19.7531	Protein of unknown function; stationary phase enriched protein; induced upon yeast-hypha transition; benomyl or caspofungin induced; Hap43-repressed; Spider biofilm induced	-1.11821	3.45E-07
orf19.7588	Ortholog of S. cerevisiae : RRG7, C. glabrata CBS138 : CAGL0I09680g, C. dubliniensis CD36 : Cd36_35140, C. parapsilosis CDC317 : CPAR2_200670 and Candida tenuis NRRL Y-1498 : CANTEDRAFT_117734	-1.12039	0.001506
orf19.2165	Predicted hydrolase; induced by nitric oxide	-1.1225	1.76E-07
orf19.1549	Plasma membrane-associated protein identified in detergent-resistant membrane fraction (possible lipid raft component); repressed by nitric oxide; predicted transmembrane helix	-1.12263	4.12E-08
orf19.5092	Has domain(s) with predicted kinetochore localization (5)	-1.12296	0.009115
orf19.4764	Ortholog(s) have poly(A)-specific ribonuclease activity, role in nuclear-transcribed mRNA poly(A) tail shortening, postreplication repair and PAN complex localization	-1.12582	8.54E-05
orf19.4756	Ortholog of S. cerevisiae : YTP1, C. dubliniensis CD36 : Cd36_08490, C. parapsilosis CDC317 : CPAR2_801590, C. auris B8441 : B9J08_004547 and Candida tenuis NRRL Y-1498 : CANTEDRAFT_109732	-1.12638	4.07E-05
ERG6	Delta(24)-sterol C-methyltransferase, converts zymosterol to fecosterol, ergosterol biosynthesis; mutation confers nystatin resistance; Hap43, GlcNAc-, fluconazole-induced; upregulated in azole-resistant strain; Spider biofilm repressed	-1.12639	2.8E-09
ARC19	Putative ARP2/3 complex subunit; Hap43-induced gene; mutation confers hypersensitivity to cytochalasin D; rat catheter biofilm repressed	-1.12686	3.18E-07
ALS4	GPI-anchored adhesin; role in adhesion, germ tube induction; growth, temperature regulated; expressed during infection of human buccal epithelial cells;	-1.12808	5.06E-05

	repressed by vaginal contact; biofilm induced; repressed during chlamydospore formation		
orf19.1387	Ortholog(s) have 4 iron, 4 sulfur cluster binding, ATPase activity, role in iron-sulfur cluster assembly, tRNA wobble uridine modification and Nbp35-Cfd1 ATPase complex, cytoplasm localization (5)	-1.12961	1.11E-08
orf19.7547	Ortholog(s) have phosphatidylinositol-3-phosphate binding, ubiquitin protein ligase activity, ubiquitin-protein transferase activity	-1.13141	1.2E-08
ERG13	3-hydroxy-3-methylglutaryl coenzyme A synthase; ergosterol biosynthesis; sumoylation target; Tn mutation affects filamentation; amphotericin B, caspofungin repressed; exponential, stationary growth phase expressed; Spider biofilm repressed	-1.13351	3.17E-10
orf19.1985	Has aminoglycoside phosphotransferase and protein kinase domains; rat catheter and flow model biofilm induced	-1.13372	0.008466
ALG5	Putative glucosyltransferase involved in cell wall mannan biosynthesis; possibly an essential gene, disruptants not obtained by UAU1 method	-1.13474	3.89E-07
orf19.6348	Predicted cysteine proteinase domain; mutants are viable	-1.13556	0.017999
EMP70	Protein with a role in endosome-to-vacuole sorting; rat catheter biofilm repressed	-1.1389	3.4E-07
orf19.3712	Protein of unknown function; induced by Mnl1 under weak acid stress; flow model biofilm induced; Spider biofilm induced	-1.13999	3.14E-07
orf19.6898.1	Ortholog of C. dubliniensis CD36 : Cd36_71020, C. parapsilosis CDC317 : CPAR2_300360, C. auris B8441 : B9J08_000715 and Candida tenuis NRRL Y-1498 : CANTEDRAFT_105022	-1.14117	4.41E-05
orf19.7131	Butyrobetaine dioxygenase, the fourth enzyme of the carnitine biosynthesis pathway	-1.14267	1.77E-10
CCT6	Putative cytosolic chaperonin Cct ring complex subunit; mutation confers hypersensitivity to cytochalasin D; GlcNAc-induced protein; Spider biofilm repressed	-1.14805	5.45E-05
RBE1	Pry family cell wall protein; Rim101, Efg1, Ssn6, alkaline repressed; O-glycosylation; no GPI anchor predicted; ketoconazol induced; regulated by Sef1, Sfu1, Hap4; flow model biofilm induced; rat catheter and Spider biofilm repressed	-1.15188	2.14E-08

CKA2	Catalytic alpha-subunit of protein kinase CK2; interaction with calcineurin pathway affects fluconazole sensitivity; synthetically lethal with CKA1; attenuated virulence in a mouse oropharyngeal candidiasis but not in a systemic mouse model	-1.15387	2.2E-06
orf19.7316	Putative phytanoyl-CoA dioxygenase family protein; mutation confers hypersensitivity to 5-fluorocytosine (5-FC), 5-fluorouracil (5-FU), and tubercidin (7-deazaadenosine); induced by nitric oxide	-1.15667	0.003199
orf19.951	Protein of unknown function; transcript repressed upon yeast-hyphal switch; fluconazole-induced; Hap43-repressed; flow model biofilm induced	-1.15704	8.11E-07
orf19.2751	Predicted membrane transporter; member of the monocarboxylate porter (MCP) family, major facilitator superfamily; Hap43-induced gene; alkaline upregulated by Rim101; possibly an essential gene, disruptants not obtained by UAU1 method	-1.15826	3.72E-06
PYC2	Putative pyruvate carboxylase; binds biotin cofactor; repressed by Ssk1 response regulator, by benomyl treatment, or in an azole-resistant strain overexpressing MDR1; stationary phase enriched protein; flow model biofilm repressed	-1.15977	1.36E-05
orf19.2835	Ortholog(s) have SUMO activating enzyme activity, role in mitotic chromosome condensation, protein sumoylation, regulation of mitotic chromosome condensation and SUMO activating enzyme complex, cytosol, nucleus localization	-1.16083	4.33E-06
SEC14	Essential protein; functional homolog of S. cerevisiae Sec14p, a Golgi phosphatidylinositol/phosphatidylcholine transfer protein that regulates choline-phosphate cytidyltransferase and thereby affects secretion; biofilm-regulated	-1.16617	8.92E-08
orf19.3151	Has domain(s) with predicted catalytic activity and role in cellular metabolic process	-1.16881	0.000792
FCY2	Purine-cytosine permease of pyrimidine salvage; mutation associated with resistance to flucytosine in clinical isolates; transposon mutation affects filamentation; farnesol-upregulated in biofilm	-1.17132	5.67E-06
orf19.6440	Ortholog(s) have ubiquitin-protein transferase activity and role in cellular response to amino acid stimulus, transcription factor catabolic process, ubiquitin-dependent protein catabolic process	-1.17174	6.94E-07

orf19.7449	Ortholog(s) have role in mitochondrial genome maintenance, plasmid maintenance	-1.17206	0.009203
HIS7	Putative imidazole glycerol phosphate synthase; histidine biosynthesis; no human/murine homolog; transcription induced by histidine starvation; regulated by Gcn2p and Gcn4p; higher protein level in stationary phase	-1.17356	5.51E-09
ERG7	2,3-epoxysqualene-lanosterol cyclase (lanosterol synthase), conversion of 2,3-oxidosqualene to lanosterol in sterol biosynthesis; fluconazole-induced; possibly essential, disruptants not obtained by UAU1 method; rat catheter biofilm induced	-1.17479	5.47E-05
orf19.7033	Putative dual specificity protein phosphatase, similar to S. cerevisiae Pps1p	-1.17729	0.017346
IMP1	Predicted subunit of the mitochondrial inner membrane peptidase complex involved in protein targeting to mitochondria	-1.17945	1.54E-06
VPS28	ESCRT I protein sorting complex subunit; involved in Rim8 processing and proteolytic activation of Rim101, which regulates pH response; role in echinocandin, azole sensitivity	-1.18007	0.001535
MET14	Putative adenylylsulfate kinase; predicted role in sulfur metabolism; possibly adherence-induced; protein present in exponential and stationary growth phase yeast; F-12/CO2 biofilm induced	-1.18197	4.08E-09
EFT2	Elongation Factor 2 (eEF2); GTPase; essential; highly expressed; target of sordarin antifungals; antigenic in human/mouse; lacks site for regulatory phosphorylation by eEF2 kinase; GCN-regulated; higher protein amount in stationary phase	-1.18581	5.4E-06
orf19.5539	Ortholog(s) have SNAP receptor activity, role in retrograde vesicle-mediated transport, Golgi to endoplasmic reticulum and SNARE complex, integral component of cytoplasmic side of endoplasmic reticulum membrane localization	-1.18896	1.43E-06
MNN11	Ortholog(s) have alpha-1,6-mannosyltransferase activity, role in protein N-linked glycosylation, protein glycosylation and mannan polymerase complex localization	-1.19051	0.000927
orf19.6861	Ortholog(s) have ubiquitin protein ligase activity, role in anaphase-promoting complex-dependent catabolic process, chromatin assembly, protein ubiquitination and anaphase-promoting complex localization	-1.19475	1.4E-06

orf19.1075	Protein of unknown function; Spider biofilm induced	-1.20122	0.001218
CYB5	Cytochrome b(5); ortholog of S. cerevisiae Cyb5; induced in high iron; fluconazole-induced; shows colony morphology-related gene regulation by Ssn6; mutants are viable	-1.20446	2.23E-08
SCS7	Putative ceramide hydroxylase; regulated by Nrg1; induced in high iron; fluconazole-induced; Hap43-repressed; Spider biofilm induced	-1.20505	4.34E-08
orf19.2893	Protein of unknown function; regulation correlates with clinical development of fluconazole resistance; transcript is upregulated in an RHE model of oral candidiasis	-1.20988	5.54E-06
GAP2	General broad specificity amino acid permease; ketoconazole, flucytosine repressed; Ssy1-dependent histidine induction; regulated by Nrg1, Tup1; colony morphology-related gene regulation by Ssn6; Spider and flow model biofilm induced	-1.21028	4.92E-07
orf19.5633	F-box domain-containing protein; flow model biofilm induced	-1.21036	5.02E-08
ERG4	Protein similar to sterol C-24 reductase; shows Mob2p-dependent hyphal regulation; fluconazole-induced; caspofungin repressed; rat catheter biofilm repressed	-1.21079	4.02E-11
PMT6	Protein mannosyltransferase; required for virulence in mice, adhesion to endothelium; role in hyphal growth signaling, hygromycin B sensitivity; no major role in cellular PMT activity; Hap4-repressed; Bcr1-repressed in RPMI a/a biofilms	-1.21389	5.1E-07
RPO21	RNA polymerase II; ortholog of S. cerevisiae Rpo21, transposon mutation affects filamentous growth; flow model biofilm repressed	-1.21469	8.17E-05
orf19.7305	Ortholog of C. dubliniensis CD36 : Cd36_34510, Candida tropicalis NEW ASSEMBLY : CTRG1_05938, Candida tropicalis MYA-3404 : CTRG_05938 and Candida albicans WO-1 : CAWG_02183	-1.21543	9.01E-07
orf19.5300	Calnexin; integral membrane ER chaperone involved in folding and quality control of glycoproteins; caspofungin induced	-1.21605	3.04E-08
orf19.6194	Ortholog of C. dubliniensis CD36 : Cd36_06640, C. parapsilosis CDC317 : CPAR2_208220, C. auris B8441 : B9J08_002282 and Candida tenuis NRRL Y-1498 : CANTEDRAFT_136902	-1.21643	0.000609

orf19.3466	Predicted methyltransferase; downregulated by fluphenazine treatment or in an azole-resistant strain that overexpresses CDR1 and CDR2	-1.21885	0.000818
orf19.5757	Ortholog(s) have FAD diphosphatase activity and role in flavin-containing compound metabolic process	-1.21897	5.07E-08
MAK32	Putative protein involved in the structural stability of L-A double-stranded RNA-containing particles; downregulated upon adherence to polystyrene	-1.21981	9.05E-05
orf19.3528	Protein of unknown function; Spider biofilm induced	-1.22149	3.77E-07
NUO1	NADH-ubiquinone oxidoreductase subunit with roles in mitochondrial respiratory chain complex I assembly	-1.22864	6.98E-09
NOP15	Nucleolar ribosome biogenesis factor; hyphal-induced expression; Hap43-induced; rat catheter biofilm induced	-1.22873	9.62E-06
COX7	Putative cytochrome c oxidase; flucytosine induced; repressed by nitric oxide	-1.22979	2.25E-05
GIT2	Putative glycerophosphoinositol permease; fungal-specific; repressed by alpha pheromone in SpiderM medium; Hap43-repressed; Spider biofilm induced	-1.23077	1.38E-08
orf19.3633	Ortholog(s) have role in purine nucleobase catabolic process	-1.23185	0.001142
orf19.1448.1	Ortholog(s) have plus-end-directed microtubule motor activity, protein-containing complex binding activity (2)	-1.23236	0.018887
orf19.1160	Ortholog(s) have COPII receptor activity and role in endoplasmic reticulum to Golgi vesicle-mediated transport, fungal-type cell wall organization, protein retention in Golgi apparatus	-1.23665	9.02E-10
AGM1	Phosphoacetylglucosamine mutase (N-acetylglucosamine-phosphate mutase); enzyme of UDP-N-acetylglucosamine (UDP-GlcNAc) biosynthesis	-1.24862	2.56E-07
CDC28	Cyclin-dependent protein kinase; interacts with regulatory subunit Cyb1; determination of cell morphology during the cell cycle; phosphorylated mostly by Swe1 and phosphorylation is regulated by Hsl1; 5'-UTR intron; Spider biofilm repressed	-1.24906	4.42E-08

orf19.4940	Putative histidine permease; fungal-specific (no human or murine homolog); Hap43p-induced gene	-1.25199	4.07E-09
orf19.6356	Ortholog(s) have role in mRNA splicing, via spliceosome and U4/U6 snRNP, U4/U6 x U5 tri-snRNP complex localization	-1.25324	0.002708
HYR3	Putative GPI-anchored adhesin-like protein; similar to Hyr1p induced in high iron; clade-specific repeat variation	-1.25561	1.49E-07
orf19.4676	Protein with homology to mitochondrial intermembrane space proteins; regulated by Sef1p-, Sfu1p-, and Hap43p	-1.25599	0.002557
orf19.5618	Ortholog(s) have SNARE binding, unfolded protein binding activity	-1.25632	0.000037
orf19.6132	Ortholog of ***C. dubliniensis CD36*** **: Cd36_33000,** ***C. parapsilosis CDC317*** **: CPAR2_701740,** ***C. auris B8441*** **: B9J08_000567 and** ***Candida tenuis NRRL Y-1498*** **: CANTEDRAFT_93887**	-1.25659	7.79E-06
orf19.4607	Possible Golgi membrane protein; Hap43-repressed; hypha induced; flow model biofilm induced; Spider biofilm induced	-1.25857	0.01352
orf19.1477	Protein of unknown function; possible ER protein; Hap43p-repressed; Spider biofilm induced	-1.26097	1.49E-06
TVP18	Putative integral membrane protein; fluconazole-induced	-1.26428	2.95E-05
orf19.3302	Putative type-1 protein phosphatase targeting subunit; transcript repressed by yeast-hyphal switch; transcript induced by Mnl1p under weak acid stress; flow model biofilm induced (5, 6, 7, 8)	-1.26781	5.73E-06
CYB2	Putative cytochrome b2 precursor; induced in high iron; alkaline repressed; colony morphology-related gene regulation by Ssn6; Hap43-repressed; pider biofilm induced	-1.26862	0.001171
ERV29	Putative SURF4 family member; plasma membrane-localized; flow model biofilm repressed	-1.27	2.82E-10
UPC2	Zn2-Cys6 transcript factor; regulator of ergosterol biosynthetic genes and sterol uptake; binds ERG2 promoter; induced by ergosterol depletion, by azoles, anaerobicity; macrophage/pseudohyphal-repressed; flow model biofilm induced	-1.27294	6.11E-08

orf19.3247	Putative ortholog of S. cerevisiae Laa1p; likely to be essential for growth, based on an insertional mutagenesis strategy	-1.27591	2.79E-05
GAP5	General amino acid permease; fungal-specific (no human or murine homolog)	-1.27623	2.14E-09
CAM1	Putative translation elongation factor eEF1 gamma; protein level decreased in stationary phase cultures; Spider biofilm repressed (1, 5)	-1.27751	1.95E-10
ARG83	GAL4-like Zn(II)2Cys6 transcription factor; clade-associated expression; null shows abnormal regulation of invasive colony growth, is unable to utilize proline as a nitrogen source; flow model biofilm induced	-1.27769	0.001595
SLC1	Putative fatty acyltransferase; protein repressed during the mating process	-1.27853	7.24E-10
orf19.4580	Protein of unknown function; Hap43-repressed gene	-1.27856	3.78E-07
orf19.1158	Ortholog of S. cerevisiae Yft2 required for normal ER membrane biosynthesis; Hap43-repressed gene	-1.28004	1.37E-05
THI13	Thiamin pyrimidine synthase; synthesis of the thiamine precursor hydroxymethylpyrimidine phosphate; single-turnover enzyme that provides histidine for HMP-P formation; induced by nitric oxide independent of Yhb1; Spider biofilm induced	-1.2809	1.08E-05
MDH1	Mitochondrial malate dehydrogenase; regulated by Mig1, Tup1, white-opaque switch, phagocytosis; induced in high iron; antigenic during murine and human infection; repressed in Spider biofilms by Bcr1, Tec1, Ndt80, Rob1, Brg1	-1.28958	6.76E-12
MNT1	Alpha-1,2-mannosyl transferase; predicted type II Golgi membrane protein; adds 2nd mannose during cell-wall mannoprotein biosynthesis; required for wild-type virulence and adherence to epithelial cells; Hap43-induced	-1.28995	1.18E-10
orf19.2306	Ortholog(s) have acireductone dioxygenase (Ni2+-requiring) activity and role in L-methionine salvage from methylthioadenosine	-1.29178	1.11E-06
CDC53	Cullin, a scaffold subunit of the SCF ubiquitin-ligase complexes; depletion leads to increased filamentous growth and premature cell death	-1.29179	8.97E-08

FTR1	High-affinity iron permease; required for mouse virulence, low-iron growth; iron, amphotericin B, caspofungin, ciclopirox, Hog1p, Sef1p, Sfu1p, and Hap43p regulated; complements S. cerevisiae ftr1 iron transport; Hap43p-repressed	-1.29183	2.11E-09
GUA1	Putative GMP synthase, involved in the final step of guanine biosynthesis; soluble protein in hyphae; flucytosine induced; macrophage-downregulated protein abundance; protein level decreases in stationary phase cultures	-1.29431	8.79E-10
ANP1	Putative mannosyltransferase of Golgi; member of Mnn9p family; similar to S. cerevisiae Anp1p; fungal-specific (no human or murine homolog)	-1.29611	2.04E-06
ARG8	Putative acetylornithine aminotransferase; Gcn2, Gcn4 regulated; rat catheter biofilm induced; Spider biofilm induced	-1.29744	2.36E-05
orf19.7198	CCR4-NOT complex component; involved in controlling mRNA initiation, elongation and degradation; rat catheter biofilm induced	-1.29842	7.75E-07
GAL102	UDP-glucose 4,6-dehydratase; role in mannosylation of cell wall proteins; mutation confers hypersensitivity to toxic ergosterol analog; overlaps orf19.3673; Spider biofilm induced	-1.29973	0.000637
orf19.1761	Predicted olichyl-diphosphooligosaccharide-protein glycotransferase; role in protein N-linked glycosylation; Spider biofilm repressed	-1.30061	1.73E-05
orf19.2890	Has domain(s) with predicted carbohydrate:proton symporter activity, pyrimidine nucleotide-sugar transmembrane transporter activity and role in carbohydrate transport, pyrimidine nucleotide-sugar transmembrane transport	-1.30239	7.56E-09
orf19.1381	Ortholog of S. cerevisiae/S. pombe Lsb5; predicted role in actin cortical patch localization, actin filament organization, endocytosis; flow model biofilm induced; Spider biofilm repressed	-1.30538	3.05E-08
orf19.4066	Putative glycerol-3-phosphate acyltransferase; Hog1-repressed	-1.30642	8.77E-07
orf19.5209	Ortholog(s) have role in cytoplasm to vacuole transport by the Cvt pathway, intra-Golgi vesicle-mediated transport and Golgi transport complex localization	-1.3069	4.91E-05
orf19.640	Integral membrane protein of the ER; role in the synthesis of beta-1,6-glucan in the cell wall; S. cerevisiae	-1.30718	5.58E-06

	ortholog required for cell viability; Spider biofilm induced		
orf19.247	Ortholog(s) have role in endosome organization, regulation of protein localization and BLOC-1 complex localization	-1.30873	0.000269
ILV6	Putative regulatory subunit of acetolacetate synthase; alkaline induced; regulated by Gcn2 and Gcn4; protein present in exponential and stationary growth phase yeast; Spider biofilm repressed	-1.31007	5.75E-11
ALG2	Putative mannosyltransferase involved in cell wall mannan biosynthesis; transcription is elevated in chk1, nik1, and sln1 homozygous null mutants	-1.31256	4.21E-09
orf19.2124	Predicted alcohol dehydrogenase; Spider biofilm induced	-1.313	8.73E-08
orf19.7487	Has domain(s) with predicted RNA binding, RNA-directed DNA polymerase activity and role in RNA-dependent DNA biosynthetic process	-1.31733	1.56E-12
BIG1	Endoplasmic reticulum (ER) protein; ortholog of S. cerevisiae Big1; required for beta-1,6-glucan synthesis, filamentation, adhesion, and virulence; rat catheter biofilm repressed	-1.32577	3.2E-09
orf19.1505	Ortholog of S. cerevisiae : YBR138C, C. dubliniensis CD36 : Cd36_16750, C. parapsilosis CDC317 : CPAR2_212450, C. auris B8441 : B9J08_002176 and Candida tenuis NRRL Y-1498 : CANTEDRAFT_92365	-1.3284	4.68E-06
TFB3	Putative C3HC4 zinc finger transcription factor; transcript positively regulated by Tbf1; Spider biofilm induced	-1.32986	4.91E-07
Putative 3-hydroxyanthranilic acid dioxygenase, involved in NAD biosynthesis; Hap43p-repressed gene		-1.33004	1.97E-08
END3	Regulated by Gcn4p; induced in response to amino acid starvation (3-aminotriazole treatment)	-1.33169	2.11E-09
orf19.6860	Ortholog(s) have CDP-diacylglycerol-inositol 3-phosphatidyltransferase activity, role in phosphatidylinositol biosynthetic process and Golgi apparatus, integral component of membrane, mitochondrial outer membrane localization	-1.33204	2.42E-11
orf19.1110	Thiamine pyrophosphokinase, phosphorylates thiamine to produce the coenzyme thiamine pyrophosphate (thiamine diphosphate); active as a homodimer	-1.33371	1.06E-06

orf19.5720	Predicted membrane transporter, member of the monocarboxylate porter (MCP) family, major facilitator superfamily (MFS); ketoconazole or caspofungin repressed; Spider biofilm induced	-1.34069	4.89E-11
orf19.4471	Ortholog(s) have role in TOR signaling, re-entry into mitotic cell cycle after pheromone arrest and endoplasmic reticulum, endoplasmic reticulum membrane, endoplasmic reticulum-Golgi intermediate compartment localization	-1.34184	0.001074
CSA1	Surface antigen on elongating hyphae and buds; strain variation in repeat number; ciclopirox, filament induced, alkaline induced by Rim101; Efg1-, Cph1, Hap43-regulated; required for WT RPMI biofilm formation; Bcr1-induced in a/a biofilms	-1.34233	8.48E-08
BGL22	Putative glucanase; induced during cell wall regeneration	-1.34307	3.11E-06
orf19.3105	Putative cytochrome P450 protein; possibly an essential gene, disruptants not obtained by UAU1 method	-1.34452	3.36E-06
BMT1	Beta-mannosyltransferase, required for addition of the 1st beta-mannose residue to acid-stable fraction of cell wall phosphopeptidomannan; 9-gene family member; mutants induce higher levels of inflammatory cytokines in mouse dendritic cells	-1.34622	9.22E-08
NCE103	Carbonic anhydrase; converts of CO2 to bicarbonate; essential for virulence in host niches with limited CO2, normal white-opaque switch; Mnl1-induced in weak acid stress; Hap43-induced gene; F-12/CO2, rat catheter, Spider biofilm induced	-1.34886	8.84E-06
orf19.4271	Predicted ORF from Assembly 19; removed from Assembly 20; subsequently reinstated in Assembly 21 based on comparative genome analysis	-1.34935	0.001861
AGO1	Putative Argonaute protein involved in RNA silencing; hyphal-induced expression; regulated by Cyr1, Ras1, Efg1, Nrg1, Tup1; rat catheter biofilm repressed	-1.34965	2.74E-08
SMC3	Protein similar to S. cerevisiae Smc3p, which is an ATPase involved in sister chromatid cohesion; likely to be essential for growth, based on an insertional mutagenesis strategy	-1.35654	0.017521
TUB2	Beta-tubulin; functional homolog of ScTub2; overproduction makes S. cerevisiae inviable; has two introns; GlcNAc, hypha fluconazole-induced; slow	-1.3664	1.45E-09

	growth, ectopic expression increases white-to opaque switch; rat catheter biofilm repressed		
orf19.4615	Ortholog(s) have histone deacetylase activity	-1.3682	7.02E-07
orf19.2452	Protein of unknown function; induced in high iron; repressed in core caspofungin response; ketoconazole-repressed; colony morphology-related gene regulation by Ssn6; possibly subject to Kex2 processing	-1.36944	5.9E-08
orf19.6809	Putative phosphomutase-like protein; protein present in exponential and stationary growth phase yeast; Hap43-repressed; Spider biofilm repressed	-1.36956	1.05E-10
orf19.6802	Ortholog(s) have palmitoyltransferase activity, role in protein palmitoylation, vacuole fusion, non-autophagic and fungal-type vacuole, fungal-type vacuole membrane localization	-1.37066	8.37E-07
orf19.7343	Ortholog(s) have role in snoRNA splicing, spliceosomal conformational changes to generate catalytic conformation and U4/U6 x U5 tri-snRNP complex localization	-1.37124	0.000038
orf19.2938	Putative mitochondrial inner membrane protein with a predicted role in the assembly of respiratory complex III; Hap43p-repressed gene; S. cerevisiae ortholog FMP25 localizes to mitochondrion	-1.37162	1.26E-07
orf19.7277	Predicted ORF in retrotransposon Zorro2 with similarity to zinc finger-containing retroviral nucleocapsid proteins; un-merged from orf19.7275 in a revision of Assembly 21 based on comparative genome analysis and new sequence data	-1.37171	2.9E-06
GAP4	High-affinity S-adenosylmethionine permease; required for SAM-induced morphogenesis; hyphal induced; regulated by Hap43, Gcn2 and Gcn4; colony morphology-related gene regulation by Ssnp	-1.3738	1.2E-12
orf19.31	Ortholog of C. parapsilosis CDC317 : CPAR2_201960, C. dubliniensis CD36 : Cd36_20900, Candida metapsilosis : CMET_3324 and Candida tropicalis NEW ASSEMBLY : CTRG1_04756 (5)	-1.37412	0.000176
HEM3	Hydroxymethylbilane synthase; converts 4-porphobilinogen to hydroxymethylbilane in heme biosynthesis; induced in high iron, CO2; alkaline repressed; regulated by Sef1, Sfu1, and Hap43; rat catheter and Spider biofilm induced	-1.37446	4.72E-10
orf19.7444	Ortholog(s) have transcription factor TFIIH holo complex localization	-1.37572	0.000089

orf19.1210	Ortholog(s) have L-arginine transmembrane transporter activity, L-aspartate transmembrane transporter activity and L-glutamate transmembrane transporter activity, more	-1.37627	1.78E-11
orf19.7227	Protein phosphatase inhibitor; Hap43-repressed; homozygous Tn insertion decreases colony wrinkling but does not block hyphal growth in liquid media; mutation confers hypersensitivity to toxic ergosterol analog; Spider biofilm induced	-1.37779	7.89E-05
NUP	Nucleoside permease; adenosine and guanosine are substrates, whereas cytidine, adenine, guanine, uridine, uracil are not; similar to a nucleoside permease of S. pombe; possibly processed by Kex2p	-1.38129	9.39E-12
DPP1	Putative diacylglycerol pyrophosphate phosphatase of diacylglycerol production for phospholipid biosynthesis; downregulation correlates with clinical development of fluconazole resistance	-1.38349	1.53E-08
orf19.3132	Predicted endoplasmic reticulum zinc transporter; induced by nitric oxide	-1.38407	5.57E-09
orf19.3984	Protein of unknown function; induced in core caspofungin response; induced in ssr1 mutant; induced by nitric oxide independent of Yhb1; Spider biofilm induced	-1.38578	5.22E-08
ALG8	Putative glucosyltransferase involved in cell wall mannan biosynthesis; transcription is elevated in nik1 and sln1 homozygous null mutants, but not in the chk1 null mutant; possibly an essential gene, disruptants not obtained by UAU1 method	-1.38823	9.29E-09
orf19.3456	Protein with a predicted serine/threonine kinase and tyrosine kinase domain; possibly an essential gene, disruptants not obtained by UAU1 method	-1.39556	0.003742
TKL1	Putative transketolase; localizes to surface of yeast cells, not hyphae; soluble protein in hyphae; transcript regulated by Nrg1, Mig1, and Tup1; antigenic in human or murine infection; possibly essential (by UAU1 method)	-1.39608	2.48E-09
OPT6	Putative oligopeptide transporter; fungal-specific (no human or murine homolog); expression of OPT6, OPT7, or OPT8 does not suppress defect of mutant lacking Opt1p, Opt2p, and Opt3p; alleles are nonidentical	-1.39656	2.42E-06
HGT19	Putative MFS glucose/myo-inositol transporter; 20 member family; 12 transmembrane segments, extended N terminus; expressed in rich medium; Hap43,	-1.40632	2.61E-09

	phagocytosis, rat catheter, Spider and flow model biofilm induced		
orf19.6266	Protein of unknown function; Spider biofilm induced	-1.40646	0.000032
orf19.1034	Protein with a predicted cytochrome b5-like Heme/Steroid binding domain; Hap43, caspofungin repressed; flow model biofilm induced (5, 6, 7)	-1.40767	1.96E-09
orf19.6411	Protein similar to S. cerevisiae Vac14p; a protein involved in regulated synthesis of PtdIns(3,5)P(2); transposon mutation affects filamentous growth	-1.40952	5.52E-08
GPH1	Putative glycogen phosphorylase; role in glycogen metabolism; regulated by Ssk1, Mig1, Tup1, Hap43; fluconazole-induced; localizes to cell surface of hyphae, not yeast; stationary phase enriched protein; Spider biofilm induced	-1.41036	7.64E-07
HGT7	Putative MFS glucose transporter; glucose, fluconazole, Snf3 induced, expressed at high glucose; 20 member C. albicans glucose transporter family; 12 TM regions predicted; flow model biofilm induced; Spider biofilm repressed	-1.41091	1.07E-11
orf19.6035	Protein of unknown function; repressed by nitric oxide	-1.41836	2.79E-06
SSB1	HSP70 family heat shock protein; mRNA in yeast and germ tubes; at yeast cell surface, not hyphae; antigenic in human/mouse infection; macrophage, GCN-induced; possibly essential; sumoylation target; Hap43-induced; Spider biofilm repressed	-1.41864	4.15E-06
orf19.5516	Ortholog(s) have role in SRP-dependent cotranslational protein targeting to membrane and signal recognition particle, endoplasmic reticulum targeting localization	-1.41873	3.44E-06
orf19.3613	Ortholog(s) have RNA polymerase II C-terminal domain phosphoserine binding, RNA polymerase II complex binding, chromatin binding activity	-1.41892	1.39E-08
orf19.6360	Protein involved in pre-mRNA splicing; Spider biofilm induced	-1.42112	0.000035
PHO114	Acid phosphatase; induced by Mnl1 under weak acid stress; Spider biofilm induced	-1.42353	0.001146
orf19.7366	Ortholog(s) have S-adenosylmethionine-dependent methyltransferase activity, rRNA (uridine-N3-)-	-1.4236	0.000266

	methyltransferase activity, role in rRNA base methylation and nucleolus localization		
orf19.6027	Ortholog of ***C. dubliniensis CD36*** **: Cd36_00740,** ***C. parapsilosis CDC317*** **: CPAR2_110170,** ***C. auris B8441*** **: B9J08_004945 and** ***Candida tenuis NRRL Y-1498*** **: CANTEDRAFT_133175**	-1.42636	0.0065
CAN1	Basic amino acid permease; complements lysine transport mutation; 10 predicted transmembrane regions, 3 predicted N-glycosylation sites; phagocytosis by macrophages induces transcript; rat catheter, Spider and flow model biofilm induced	-1.43117	1.21E-07
orf19.3223.1	Putative 12kDa subunit of mitochondrial NADH-ubiquinone oxidoreductase; gene has intron	-1.43226	3.51E-05
GLK4	Putative glucokinase; decreased expression in hyphae compared to yeast-form cells	-1.43607	9.46E-07
PGA26	GPI-anchored adhesin-like protein of the cell wall; role in cell wall integrity; required for normal virulence; induced in high iron and during cell wall regeneration; Hap43-repressed	-1.43991	0.000374
orf19.6275	Ortholog(s) have protein serine/threonine kinase activity, ribosomal protein S6 kinase activity, role in TORC1 signaling, peptidyl-serine phosphorylation, protein autophosphorylation and cytoplasm localization	-1.44612	1.38E-06
orf19.1344	Protein of unknown function; fluconazole-induced; Spider biofilm induced	-1.44682	2.07E-05
orf19.1183	Ortholog of S. cerevisiae : YNL115C, C. glabrata CBS138 : CAGL0L03938g, C. dubliniensis CD36 : Cd36_60200, C. parapsilosis CDC317 : CPAR2_603320 and C. auris B8441 : B9J08_002166	-1.44821	4.66E-08
PEX8	Putative peroxisomal biogenesis factor; expression regulated during planktonic growth	-1.44931	0.001529
orf19.35	Predicted kinase; rat catheter, flow model, Spider biofilm induced	-1.44959	0.000179
OPT7	Putative oligopeptide transporter; possibly transports GSH or related compounds; Hog1-induced; expression of OPT6, -7, or -8 does not suppress defect of mutant lacking OPT1-3; Hap43-repressed; F-12/CO2 early biofilm induced	-1.44965	3.01E-08

RAM1	Protein that acts in prenylation; transcription is alpha-factor induced; regulated in response to lovastatin and fluconazole; Hap43p-repressed gene	-1.44974	0.003118
orf19.4368	Has domain(s) with predicted hydrolase activity and role in cellular process	-1.45161	3.55E-05
AHP2	Putative thiol-specific peroxiredoxin; macrophage-downregulated gene	-1.45292	1.9E-11
orf19.7125	Protein of unknown function; Spider biofilm repressed	-1.45518	8.93E-08
SOH1	Subunit of the RNA polymerase II mediator complex; involved in control of cytokinesis, morphogenesis, biofilm formation, virulence; cell-cycle regulated periodic mRNA expression	-1.45841	0.007134
orf19.1152	Protein of unknown function; induced in core stress response; Gcn2 and Gcn4 regulated; flow model biofilm induced; Spider biofilm induced	-1.46031	2.05E-10
LEU4	Putative 2-isopropylmalate synthase; regulated by Nrg1, Mig1, Tup1, Gcn4; induced by human whole blood or PMNs; macrophage/pseudohyphal-repressed after 16h; Spider biofilm repressed	-1.46718	3.96E-09
orf19.4914	Ortholog(s) have role in cellular response to DNA damage stimulus and FANCM-MHF complex localization	-1.46748	0.013704
orf19.2827	Ortholog(s) have microtubule binding, molecular adaptor activity	-1.4691	0.000908
orf19.904	Ortholog(s) have cytoplasm, nucleus localization	-1.46947	1.75E-09
MSH3	Mismatch repair protein; predicted role in repair of insertion or deletion mutations and removal of nonhomologous DNA ends; rat catheter biofilm repressed	-1.47064	1.06E-06
ARP9	Component of the RSC chromatin remodeling complex; similar to S. cerevisiae Arp3p, a component of the Arp2/3 complex involved in actin-dependent processes; likely to be essential for growth, based on an insertional mutagenesis strategy	-1.47265	4.73E-09
RPN6	Putative 26S proteasome subunit; Hap43p-induced gene; regulated by Gcn2p and Gcn4p	-1.47351	1.13E-10

TEP1	Putative protein phosphatase of the PTP family (tyrosine-specific), similar to S. cerevisiae Tep1p	-1.47582	0.000035
orf19.1800	Protein of unknown function; Spider biofilm induced	-1.48181	4.36E-10
PGM2	Ortholog of S. cerevisiae Pgm2; induced in planktonic culture; Tye7p-regulated; flow model biofilm induced; rat catheter biofilm repressed	-1.48225	6.8E-13
RPB4	Protein similar to S. cerevisiae Rpb4p, which is a component of RNA polymerase II; transposon mutation affects filamentous growth	-1.48278	6.9E-07
RPF2	Putative pre-rRNA processing protein; Hap43p-induced gene; mutation confers hypersensitivity to 5-fluorocytosine (5-FC), 5-fluorouracil (5-FU), and tubercidin (7-deazaadenosine)	-1.48318	7.95E-10
orf19.4382	Ortholog(s) have role in retrograde vesicle-mediated transport, Golgi to endoplasmic reticulum and COPI vesicle coat localization	-1.48384	2.49E-11
orf19.7594	Protein of unknown function; Spider biofilm induced	-1.48425	0.000743
SPR28	Septin; similar to S. cerevisiae meiotic/sporulation septin; mutant has no obvious phenotype; two introns with noncanonical branch site and 5' splice site, respectively; splicing inhibited upon exposure to alpha-factor	-1.48454	0.003691
ALP1	Cystine transporter; present in pathogenic yeasts (no human or murine homolog); Spider biofilm induced	-1.48738	1.05E-10
orf19.4780	Predicted MFS family membrane transporter, member of the drug:proton antiporter (12 spanner) (DHA1) family; Spider biofilm induced	-1.48751	5.59E-11
ADE13	Adenylosuccinate lyase; enzyme of adenine biosynthesis; soluble protein in hyphae; not induced during GCN response, in contrast to the S. cerevisiae ortholog; repressed by nitric oxide	-1.48789	7.1E-10
orf19.2542	Ortholog of C. dubliniensis CD36 : Cd36_26520, C. parapsilosis CDC317 : CPAR2_802860, C. auris B8441 : B9J08_003507 and Candida tenuis NRRL Y-1498 : CANTEDRAFT_94578	-1.48932	0.00013
orf19.2708	Ortholog(s) have guanyl-nucleotide exchange factor activity and role in mature ribosome assembly	-1.48964	1.21E-08

LYS9	Saccharopine dehydrogenase; lysine biosynthesis; soluble protein in hyphae; amphotericin B repressed; Gcn4-regulated; colony morphology-related gene regulation by Ssn6; protein present in exponential and stationary growth phase yeast	-1.49052	1.06E-14
orf19.3449.2	Putative mitochondrial phosphatidylglycerophosphatase (PGP phosphatase); essential for cardiolipin biosynthesis; rat catheter biofilm induced	-1.49095	1.01E-07
POL30	Proliferating cell nuclear antigen (PCNA), forms homotrimeric sliding clamp for DNA polymerases; RNA abundance regulated by tyrosol, cell density; induced by flucytosine, interaction with macrophages; stationary phase enriched protein	-1.49135	1.4E-10
orf19.934	Predicted ORF from Assembly 19; removed from Assembly 20; subsequently reinstated in Assembly 21 and merged with orf19.2224, based on comparative genome analysis; flow model biofilm repressed	-1.49805	0.018746
orf19.4658	RING finger and CHY zinc finger domain-containing protein; mutant are viable	-1.49942	0.000167
orf19.21	Ortholog(s) have role in ethanol metabolic process and mitochondrial inner membrane localization	-1.50002	9.73E-07
orf19.1050	Ortholog of S. cerevisiae : YMR087W, C. glabrata CBS138 : CAGL0J01397g, C. dubliniensis CD36 : Cd36_03920, C. parapsilosis CDC317 : CPAR2_107150 and C. auris B8441 : B9J08_00513	-1.50303	4.87E-05
KTR4	Mannosyltransferase; induced during cell wall regeneration; fungal-specific (no human or murine homolog); Bcr1-repressed in RPMI a/a biofilms	-1.50614	2.56E-10
IPK1	Ortholog of S. cerevisiae/S. pombe Ipk1; an inositol pentakisphosphate 2-kinase, a nuclear protein required for synthesis of 1,2,3,4,5,6-hexakisphosphate; Spider biofilm induced	-1.5063	4.54E-07
orf19.639	S. cerevisiae ortholog YDR370C/DXO1 has decapping and 5′-3′ exoRNase activity; decreased transcription is observed upon fluphenazine treatment or in an azole-resistant strain that overexpresses CDR1 and CDR2	-1.50826	4.98E-06
OST1	Alpha subunit of the oligosaccharyltransferase complex of the ER lumen; catalyzes asparagine-linked glycosylation of newly synthesized proteins; Spider biofilm repressed	-1.50851	5.55E-12

orf19.641	Ortholog(s) have role in protein folding and endoplasmic reticulum localization	-1.51722	5.33E-07
orf19.7345	Ortholog(s) have ATP binding activity	-1.52339	1.2E-08
orf19.6230	Ortholog(s) have GDP binding, RNA NAD-cap (NAD-forming) hydrolase activity, RNA pyrophosphohydrolase activity, enzyme regulator activity, phosphodiesterase decapping endonuclease activity	-1.52723	2.89E-07
orf19.1483	Ortholog(s) have copper ion binding activity, role in mitochondrial cytochrome c oxidase assembly and extrinsic component of mitochondrial inner membrane, mitochondrial intermembrane space localization	-1.5274	3.26E-06
FET34	Multicopper ferroxidase; induced by low iron, ciclopirox olamine, ketoconazole, hypoxia; alkaline induced by Rim101; repressed in fluconazole-resistant isolate; Sfu1, Hog1 repressed; complements S. cerevisiae fet3; Spider biofilm induced	-1.52786	1.9E-10
orf19.4633	Ortholog(s) have carbonyl reductase (NADPH) activity, oxidoreductase activity, serine 3-dehydrogenase activity	-1.52845	1.47E-12
MCD4	Mannose-ethanolamine phosphotransferase, essential gene involved in GPI anchor biosynthesis	-1.53103	3.73E-08
ZCF19	Predicted Zn(II)2Cys6 transcription factor	-1.53172	0.020185
YRB1	Functional homolog of S. cerevisiae Yrb1p; regulates Gsp1 GTPase activity and thereby affects nucleocytoplasmic transport and cytoskeletal dynamics; transcript is not regulated by white-opaque switch or by dimorphic transition	-1.53436	2.67E-09
orf19.1406	Ortholog(s) have DNA-directed DNA polymerase activity, role in error-free translesion synthesis, error-prone translesion synthesis and mitochondrion, zeta DNA polymerase complex localization	-1.53726	8.63E-10
ARG3	Putative ornithine carbamoyltransferase; Gcn4-regulated; Hap43-induced; repressed in alkalinizing medium; rat catheter and Spider biofilm induced	-1.53788	7.73E-07
GPM1	Phosphoglycerate mutase; surface protein that binds host complement Factor H and FHL-1; antigenic; fluconazole, or amino acid starvation (3-AT) induced,	-1.53828	8.08E-11

	farnesol-repressed; Hap43, flow model biofilm induced; Spider biofilm repressed		
ENO1	Enolase, involved in glycolysis and gluconeogenesis; also has transglutaminase activity involved in assembly of cell wall polysaccharides; major cell-surface antigen; binds host plasmin/plasminogen; immunoprotective; may be essential	-1.54202	5.39E-10
ECM25	Non-essential protein involved in cell morphogenesis	-1.54432	0.000376
RHD1	Putative beta-mannosyltransferase required for the addition of beta-mannose to the acid-labile fraction of cell wall phosphopeptidomannan; 9-gene family memebr; regulated on yeast-hypha and white-opaque switches; Spider biofilm repressed	-1.54603	2.56E-08
PPT1	Putative serine/threonine phosphatase; induced in high iron	-1.54935	0.000343
DOG1	Putative 2-deoxyglucose-6-phosphatase; haloacid dehalogenase hydrolase/phosphatase superfamily; similar to S. cerevisiae Dog1, Dog2, Hor1, Rhr2; regulated by Nrg1, Tup1; Spider biofilm repressed	-1.55371	6.37E-14
HGT5	Putative glucose transporter of the major facilitator superfamily; the C. albicans glucose transporter family comprises 20 members; 12 probable membrane-spanning segments, extended N terminus; expressed in rich medium; Hap43p-repressed	-1.5541	6.33E-10
COX15	Cytochrome oxidase assembly protein; transcript regulated by Nrg1 and Tup1; alkaline repressed; Hap43-repressed; early-stage flow model biofilm induced; Spider biofilm repressed	-1.55748	3.48E-09
orf19.1301	Ortholog(s) have protein-macromolecule adaptor activity and role in proteasome regulatory particle assembly	-1.55907	3.41E-06
TPI1	Triose-phosphate isomerase; antigenic in mouse/human; mutation affects filamentation; macrophage-repressed; protein in exponential and stationary growth phase yeast; possibly essential; flow model biofilm induced; Spider biofilm repressed	-1.55987	5.42E-11
TPD3	Subunit of protein serine/threonine phosphatase PPA2, involved in regulation of cytokinesis and morphogenesis; forms complex with catalytic subunit Pph21p that dephosphorylates septin Sep7p	-1.56002	2.26E-09

orf19.7360	Ortholog(s) have enzyme activator activity, role in histone deubiquitination, regulation of transcription by RNA polymerase II and DUBm complex, SAGA complex localization	-1.56316	0.001675
OPT1	Oligopeptide transporter; transports 3-to-5-residue peptides; alleles are distinct, one has intron; suppresses S. cerevisiae ptr2-2 mutant defects; induced by BSA or peptides; Stp3p, Hog1p regulated; flow model biofilm induced	-1.56686	1.98E-15
orf19.2227	Protein of unknown function; flow model biofilm induced; nitric oxide-repressed (5, 6)	-1.57097	0.000012
orf19.1796	Putative glyoxylate reductase; acts on glyoxylate and hydroxypyruvate substrates; Spider biofilm repressed	-1.57375	6.74E-13
orf19.2335	Putative aspartyl aminopeptidase; stationary phase enriched protein; mutation confers hypersensitivity to 5-fluorouracil (5-FU); Hog1-induced; planktonic growth-induced; rat catheter and Spider biofilm repressed	-1.57595	6.97E-13
orf19.1082	Protein with an Alba DNA/RNA-binding protein domain; Spider biofilm induced	-1.57725	0.000128
orf19.4760	Putative protein-histidine N-methyltransferase; Spider biofilm induced (5)	-1.57933	7.64E-08
orf19.5267	Putative cell wall adhesin-like protein; repressed in core caspofungin response and by alpha pheromone in SpiderM medium; transcript reduced in ace2 mutant; flow model, rat catheter and Spider biofilm repressed	-1.58026	7.84E-11
orf19.5038	Predicted tRNA (guanine) methyltransferase activity; Spider biofilm induced	-1.58562	2.83E-06
orf19.7204	Has domain(s) with predicted catalytic activity, nitronate monooxygenase activity	-1.58801	3.75E-07
URA1	Dihydroorotate dehydrogenase; de novo pyrimidine biosynthesis; regulated by yeast-hypha switch, Nrg1/Mig1/Tup1; flow model biofilm induced; rat catheter and Spider biofilm repressed	-1.5882	6.15E-11
orf19.1738.1	Protein involved in microtubule morphogenesis; required for protection from excess free beta-tubulin; possibly involved the beta-tubulin folding; Spider biofilm repressed	-1.58832	0.000018

orf19.4316	Trimethyllysine dioxygenase, the first enzyme in the carnitine biosynthesis pathway; hypha-induced expression, regulated by Cyr1, Ras1, Efg1; rat catheter biofilm repressed	-1.58897	1.66E-09
LYS1	Saccharopine dehydrogenase (biosynthetic); enzyme of alpha-aminoadipate lysine biosynthesis pathway; functionally complements S. cerevisiae lys1 mutation; fungal-specific (no human or murine homolog)	-1.59245	3.97E-15
PHO84	High-affinity phosphate transporter; transcript regulated by white-opaque switch; Hog1, ciclopirox olamine or alkaline induced; caspofungin, stress repressed; required for normal TORC1 function	-1.59429	2.3E-11
orf19.2763	Protein not essential for viability; orf19.10279 possibly transcriptionally regulated upon hyphal formation	-1.59448	3.44E-08
orf19.4984	Pseudogene; has lysine motifs associated with chitin binding	-1.59833	9.06E-08
PFK2	Phosphofructokinase beta subunit; fructose 2,6-bisphosphate, AMP activated; ATP inhibited; phagocytosis, hyphal repressed; fluconazole-induced; stationary-phase enriched; flow model biofilm induced; rat catheter/Spider biofilm repressed	-1.59916	1.04E-08
orf19.2650	Protein of unknown function; rat catheter biofilm induced	-1.60415	1.68E-05
SEM1	Component of the 19S regulatory particle lid of the proteasome	-1.60471	1.99E-06
SRT1	Ortholog(s) have dehydrodolichyl diphosphate synthase activity, prenyltransferase activity, role in dolichol biosynthetic process, protein glycosylation and dehydrodolichyl diphosphate synthase complex, lipid droplet localization	-1.60554	0.000326
TUB1	Alpha-tubulin; gene has intron; complements cold-sensitivity of S. cerevisiae tub1 mutant; C. albicans has single alpha-tubulin gene, whereas S. cerevisiae has two (TUB1, TUB3); farnesol-upregulated in biofilm; sumoylation target	-1.60573	4.63E-14
orf19.2047	Putative protein of unknown function; Hap43p-repressed gene; mutation confers hypersensitivity to toxic ergosterol analog, and to amphotericin B	-1.60766	5.3E-13

SAM51	Component of the SAM complex involved in mitochondrial protein import, involved in beta-barrel protein assembly; member of the Omp85 protein family	-1.60989	1.29E-13
orf19.137	Putative transferase involved in phospholipid biosynthesis; induced by alpha pheromone in SpiderM medium	-1.61838	2.12E-09
FBA1	Fructose-bisphosphate aldolase; glycolytic enzyme; antigenic in murine/human infection; regulated by yeast-hypha switch; induced by Efg1, Gcn4, Hog1, fluconazole; phagocytosis-repressed; flow model biofilm induced; Spider biofilm repressed	-1.62089	4.52E-11
RSN1	Protein of unknown function; flow model biofilm induced; Spider biofilm induced; induced during the mating process; Hap43-repressed	-1.62395	3.44E-05
HOC1	Protein with similarity to mannosyltransferases; similar to S. cerevisiae Hoc1p and C. albicans Och1p	-1.62398	7.27E-12
orf19.6805	Protein of unknown function; Spider biofilm induced	-1.62484	9.1E-06
PTR22	Oligopeptide transporter involved in uptake of di-/tripeptides; regulated by Stp2 and Stp3; transcript induced upon phagocytosis by macrophage; repressed by Rim101 at pH 8; flow model biofilm induced	-1.62875	1.16E-12
EPT1	Diacylglycerol cholinephosphotransferase and ethanolaminephosphotransferase, catalyzes the final step in Kennedy pathway of phosphatidylcholine and phosphatidylethanolamine biosynthesis	-1.62986	5.22E-15
orf19.916	Putative protein involved in apoptosis; Spider biofilm induced	-1.6346	1.11E-13
orf19.5043	Ortholog of ***C. dubliniensis CD36*** **: Cd36_43610,** ***C. parapsilosis CDC317*** **: CPAR2_403770,** ***C. auris B8441*** **: B9J08_000308,** ***Debaryomyces hansenii CBS767*** **: DEHA2G16962g and** ***Pichia stipitis Pignal*** **: PICST_30926**	-1.64673	0.002104
TDH3	NAD-linked glyceraldehyde-3-phosphate dehydrogenase; binds fibronectin, laminin; at cell surface; antigenic in infection; farnesol-repressed; stationary phase-enriched; GlcNAc-induced; flow model biofilm induced; Spider biofilm repressed	-1.65192	8.11E-12
MTW1	Kinetochore component; amount of Nuf2p and Mtw1p protein detected at each centromere is consistent with a single kinetochore microtubule attachment site	-1.65208	5.79E-05

AYR2	Putative NADPH-dependent 1-acyl dihydroxyacetone phosphate reductase; shows colony morphology-related gene regulation by Ssn6p	-1.65642	3.47E-13
MRPL33	Putative mitochondrial ribosomal protein of the large subunit; Ssr1-repressed; rat catheter biofilm induced	-1.65657	0.002514
orf19.3352	Has domain(s) with predicted oxidoreductase activity and role in metabolic process	-1.65683	5.07E-09
orf19.192	Ortholog of C. dubliniensis CD36 : Cd36_19320, C. parapsilosis CDC317 : CPAR2_209670, C. auris B8441 : B9J08_002950 and Candida tenuis NRRL Y-1498 : CANTEDRAFT_113478	-1.65776	1.36E-05
TLG2	Putative syntaxin-like t-SNARE; macrophage/pseudohyphal-repressed	-1.66525	1.28E-06
LYS12	Homoisocitrate dehydrogenase; catalyzes 4th step in the alpha-aminoadipate pathway of lysine biosynthesis; clade-associated gene expression; protein level decreases in stationary phase cultures; Spider biofilm repressed	-1.66707	5.3E-17
orf19.2794	Putative non-specific single-domain racemase; regulated by Gcn4p; repressed in response to amino acid starvation (3-AT treatment); alkaline upregulated; macrophage-induced protein	-1.66895	2.23E-09
PPS1	Putative dual specificity protein phosphatase, similar to S. cerevisiae Pps1p; mutant shows virulence defect	-1.66946	1.97E-06
orf19.6601	Protein of unknown function; rat catheter and flow model biofilm induced	-1.66966	9.2E-08
ARF3	Similar to but not orthologous to S. cerevisae Arf3; transcript filament induced; Tup1 regulated; rat catheter biofilm repressed (see Locus History Note for Assembly 19 correction)	-1.67166	5.76E-13
AGE3	Putative ADP-ribosylation factor GTPase activating protein, functional ortholog of S. cerevisiae GCS1; mutation affects endocytosis, hyphal growth, chemical and drug resistance, and sensitivity to cell wall inhibitors	-1.67239	5.95E-14
DSE1	Essential cell wall protein involved in cell wall integrity and rigidity; periodic mRNA expression peaks at M/G1 phase; Ace2p-induced; required for virulence in a mouse model of infection	-1.67356	1.23E-07

orf19.775	Ortholog of C. dubliniensis CD36 : Cd36_04450, C. parapsilosis CDC317 : CPAR2_105460, C. auris B8441 : B9J08_002463, Debaryomyces hansenii CBS767 : DEHA2D07128g and Pichia stipitis Pignal : PICST_80203	-1.67976	4.88E-08
LHP1	Ortholog(s) have RNA binding, RNA strand annealing activity, tRNA binding activity, role in tRNA 3'-trailer cleavage, tRNA 3'-trailer cleavage, endonucleolytic, tRNA folding, tRNA processing and nucleolus, nucleoplasm, nucleus localization	-1.67984	1.03E-09
orf19.1502	Has domain(s) with predicted aminoacyl-tRNA hydrolase activity (5)	-1.69278	2.34E-05
ALG6	Putative glucosyltransferase involved in cell wall mannan biosynthesis; transcription is elevated in chk1, nik1, and sln1 homozygous null mutants; repressed by nitric oxide; possibly essential gene, disruptants not obtained by UAU1 method	-1.69494	2.76E-11
orf19.2770	Ortholog of C. dubliniensis CD36 : Cd36_42220, C. parapsilosis CDC317 : CPAR2_500340, Debaryomyces hansenii CBS767 : DEHA2G17754g and Pichia stipitis Pignal : PICST_31424	-1.70094	0.000107
orf19.3627	Ortholog of C. dubliniensis CD36 : Cd36_22640, C. parapsilosis CDC317 : CPAR2_406910, C. auris B8441 : B9J08_004017 and Candida tenuis NRRL Y-1498 : CANTEDRAFT_104937	-1.70985	0.001311
orf19.4080	Putative ribonuclease H2 subunit; required for RNase H2 activity; repressed in Spider biofilms by Bcr1, Tec1, Brg1, Rob1	-1.71384	1.42E-09
orf19.2869	Ortholog of ***C. dubliniensis CD36*** **: Cd36_46110,** ***Candida tropicalis NEW ASSEMBLY*** **: CTRG1_03792,** ***Candida tropicalis MYA-3404*** **: CTRG_03792 and** ***Candida albicans WO-1*** **: CAWG_03165**	-1.7197	7.16E-11
orf19.2838	Protein of unknown function; mutation confers hypersensitivity to amphotericin B; flow model biofilm induced	-1.72184	6.53E-06
AVT7	Ortholog of S. cerevisiae Avt7 transporter; repressed upon adherence to polystyrene; constitutive expression independent of MTL or white-opaque status; Spider biofilm induced	-1.72557	2.18E-16
UAP1	UDP-N-acetylglucosamine pyrophosphorylase, catalyzes biosynthesis of UDP-N-acetylglucosamine from UTP and N-acetylglucosamine 1-phosphate; functional homolog of S. cerevisiae Qri1p; alkaline upregulated	-1.72637	1.78E-12

TOM70	Ortholog(s) have mitochondrion targeting sequence binding, protein transmembrane transporter activity	-1.72854	2.95E-15
NIT2	Putative carbon-nitrogen hydrolase; rat catheter biofilm repressed	-1.73182	7.66E-07
orf19.4550	Predicted MFS membrane transporter, member of the drug:proton antiporter (12 spanner) (DHA1) family; flow model biofilm induced	-1.73234	1.73E-13
GPI1	Putative protein of GPI synthesis; expression is reduced in a fluconazole-resistant isolate	-1.73497	2.44E-05
orf19.1500	Ortholog of C. dubliniensis CD36 : Cd36_16720, C. parapsilosis CDC317 : CPAR2_210210, C. auris B8441 : B9J08_004860 and Candida tenuis NRRL Y-1498 : CANTEDRAFT_128446	-1.73522	2.94E-09
orf19.2669	ORF in retrotransposon Tca4; similar to Pol region of retrotransposons encoding reverse transcriptase, protease, integrase; downstream of RHD2, similar to the Gag region encoding nucleocapsid-like protein; rat catheter biofilm induced	-1.73911	1.14E-10
orf19.5620	Stationary phase enriched protein; Gcn4-regulated; induced by amino acid starvation (3-AT), benomyl or in azole-resistant strain that overexpresses MDR1; flow model biofilm induced; rat catheter biofilm repressed; overlaps orf19.5621	-1.73912	1.96E-15
PDC11	Pyruvate decarboxylase; antigenic; on hyphal not yeast cell surface; Hap43, Gcn4, Efg1, Efh1, Hsf1 regulated; fluconazole, farnesol induced; amino acid starvation repressed; flow model biofilm induced; Spider biofilm repressed	-1.74235	4.89E-11
orf19.5428	Putative Golgi membrane protein with a predicted role in manganese homeostasis; Hap43p-repressed gene; clade-associated gene expression	-1.74396	7.38E-10
HEM13	Coproporphyrinogen III oxidase; antigenic; on yeast cell surface, not hyphae; iron-regulated expression; Hap43, macrophage-repressed; farnesol-induced; possibly essential; flow model biofilm induced; rat catheter, Spider biofilm repressed	-1.74672	8.46E-15
SSN8	Ortholog of S. cerevisiae Ssn8; a component of RNA polymerase II holoenzyme; mutants are viable and are sensitive to hydrogen peroxide medium	-1.75079	4.31E-05

orf19.1491	Ortholog(s) have RNA binding activity, role in mRNA splicing, via spliceosome and U1 snRNP, U2-type prespliceosome localization	-1.75441	8.22E-09
orf19.6816	Putative xylose and arabinose reductase; flow model biofilm induced; Spider biofilm repressed	-1.7608	5.07E-11
SMC2	Protein similar to S. cerevisiae Smc2p, which is a component of the condensin complex involved in mitotic chromosome condensation; induced under hydroxyurea treatment	-1.76633	2.72E-05
orf19.4153	Ortholog(s) have NEDD8 activating enzyme activity and role in protein neddylation	-1.76912	5.79E-07
VHT1	Predicted membrane transporter, involved in biotin import; member of the anion:cation symporter (ACS) family, major facilitator superfamily (MFS); biotin-dependent transcription regulated by Vhr1p; amphotericin B, caspofungin repressed	-1.76977	4.74E-07
orf19.5611	Predicted 3-methylbutanol:NAD(P) oxidoreductase and methylglyoxal reductase (NADPH-dependent); role in ergosterol metabolic process; early stage flow model biofilm induced; Spider biofilm induced	-1.77063	0.006314
orf19.199	Protein of unknown function; hyphal-induced expression; upregulated in a cyr1 null mutant	-1.77241	1.85E-12
FOX2	3-hydroxyacyl-CoA epimerase; fatty acid beta-oxidation; induced by phagocytosis; regulated by Mig1, by white-opaque switch, by DNA methylation; transcriptional activation by oleate requires Ctf1; rat catheter and Spider biofilm induced	-1.77259	3.19E-09
orf19.4264	Protein of unknown function; induced during chlamydospore formation in both C. albicans and C. dubliniensis; flow model biofilm induced	-1.7789	1.02E-05
orf19.4751	Ortholog(s) have structural constituent of ribosome activity and mitochondrial small ribosomal subunit localization	-1.7807	8.61E-10
orf19.2006	Ortholog of S. cerevisiae : YPL162C, C. glabrata CBS138 : CAGL0M02057g, C. dubliniensis CD36 : Cd36_16070, C. parapsilosis CDC317 : CPAR2_213850 and C. auris B8441 : B9J08_003194	-1.7882	1.27E-06
orf19.3483	Putative phosphatidyl glycerol phospholipase C; Plc1-regulated; flow model biofilm induced; Spider biofilm induced	-1.78982	6.07E-08

orf19.3615	Protein of unknown function; induced in core caspofungin response; expression upregulated in an ssr1 null mutant; induced by nitric oxide independent of Yhb1p	-1.79388	1.64E-07
GPX2	Similar to glutathione peroxidase; induced in high iron; alkaline induced by Rim101; induced by alpha factor or interaction with macrophage; regulated by Efg1; caspofungin repressed; Spider biofilm induced	-1.79719	6.11E-16
PGI1	Glucose-6-phosphate isomerase; enzyme of glycolysis; antigenic; Efg1-regulated; induced upon adherence to polystyrene; repressed by phagocytosis, human neutrophils; flow model biofilm induced; rat catheter and Spider biofilm repressed	-1.81125	3.54E-12
orf19.5270	Protein of unknown function; rat catheter biofilm induced	-1.81649	0.003912
orf19.4861	Protein of unknown function; rat catheter biofilm repressed	-1.8191	0.003805
YKT6	Putative protein of the vacuolar SNARE complex; predicted role in vacuolar fusion; rat catheter biofilm repressed	-1.82372	6.87E-13
orf19.3053	Protein of unknown function; present in exponential and stationary phase yeast; identified in extracts from biofilm and planktonic cells; flow model biofilm induced gene; GlcNAc-induced protein	-1.82796	9.74E-17
REX2	Putative 3'-5' RNA exonuclease with a predicted role in 3'-end processing of U4 and U5 snRNAs, 5S and 5.8S rRNAs; rat catheter biofilm induced	-1.83119	3.18E-08
SLD1	Sphingolipid delta-8 desaturase; catalyzes desaturation at C8 in the long-chain base moiety of ceramides in glucosylceramide synthesis, important for virulence; ketoconazole and hypoxia induced; Hap43-repressed; Spider biofilm induced	-1.85262	4.6E-14
MVB12	ESCRT-I complex subunit with a role in multivesicular body (MVB) trafficking; mutant displays growth defect on alkaline medium; Hap43p-repressed gene	-1.85466	3.72E-06
ALI1	Putative NADH-ubiquinone oxidoreductase; in detergent-resistant membrane fraction (possible lipid raft component); predicted N-terminal acetylation; nitric oxide-repressed; plasma membrane-localized; protein decreases in stationary phase	-1.85679	1.27E-12
CCJ1	Protein involved in cell cycle regulation; ortholog of S. pombe SPAC1071.09c DNAJ domain protein; Hap43-induced gene	-1.86237	1.23E-10

ROT1	Similar to S. cerevisiae Rot1p, which is involved in cell wall 1,6-beta-glucan biosynthesis; has predicted transmembrane region; possibly an essential gene, disruptants not obtained by UAU1 method	-1.8632	1.7E-10
VRP1	Verprolin-related protein involved in actin cytoskeleton organization and polarized morphogenesis; interacts with Wal1p and Myo5p; downregulated upon adherence to polystyrene	-1.87011	2.43E-10
orf19.2018.1	PProtein of unknown function; gene has intron; similar to human BLOC1S2 (1)	-1.87112	0.011681
orf19.3228	Putative endosomal transmembrane protein; Hap43p-induced; mutation confers hypersensitivity to amphotericin B	-1.87336	5.72E-10
VRG4	GDP-mannose transporter; essential; required for glycosylation, hyphal growth; functional homolog of S. cerevisiae Vrg4p, which imports GDP-mannose from cytoplasm to Golgi for protein and lipid mannosylation; no mammalian homolog	-1.87814	1.61E-19
orf19.6023	Protein with a predicted multidrug transporter domain; Hap43-repressed gene	-1.87859	5.76E-12
FEN12	Putative protein with a predicted role in the elongation of fatty acids; amphotericin B, caspofungin repressed	-1.88438	1.21E-16
orf19.3758	Has domain(s) with predicted membrane localization	-1.88637	1.97E-14
orf19.5854.1	Ortholog of Candida albicans WO-1 : CAWG_02725	-1.88684	0.003654
ERG251	C-4 sterol methyl oxidase; role in ergosterol biosynthesis; Hap43-induced; ketoconazole-induced; amphotericin B, caspofungin repressed; possibly essential gene, disruptants not obtained by UAU1 method; Spider biofilm repressed	-1.89037	1.75E-14
CAM1-1	Putative translation elongation factor; downregulated upon phagocytosis by murine macrophages; Hap43-induced gene; Spider biofilm repressed	-1.8956	1.49E-16
orf19.7038	Ortholog(s) have phosphatidylinositol-3-phosphate binding activity, role in plasma membrane tubulation, protein targeting to vacuole, retrograde transport, endosome to Golgi and cytoplasm, endosome, nucleus localization	-1.89658	3.29E-09

orf19.698	Integral ER membrane protein; predicted role in maintenance of ER zinc homeostasis; Spider biofilm induced	-1.89745	4E-18
orf19.4474	Ortholog(s) have proteasome binding activity and role in cellular response to arsenic-containing substance, proteasome-mediated ubiquitin-dependent protein catabolic process	-1.90459	5.26E-09
orf19.6443	Protein with an enoyl-CoA hydratase related domain; Spider biofilm induced	-1.90577	8.18E-07
ERG1	Squalene epoxidase, epoxidation of squalene to 2,3(S)-oxidosqualene; ergosterol biosynthesis; allylamine antifungal drug target; NADH reducing cofactor but S. cerevisiae Erg1 uses NADPH; flow model biofilm induced; Spider biofilm repressed	-1.91286	9.17E-17
orf19.1137	Thymidylate kinase of unknown role; forms a dimer; potential target for antifungal drugs	-1.91644	2.89E-08
LAP3	Putative aminopeptidase; positively regulated by Sfu1; clade-associated gene expression; virulence-group-correlated expression; induced by alpha pheromone in SpiderM medium; Hap43-induced; Spider and flow model biofilm induced	-1.92387	8.39E-08
GEF2	Member of the voltage chloride channel family; Hap43p-repressed gene	-1.92428	9.74E-11
orf19.5905	Protein of unknown function; Hap43-induced; F-12/CO2 early biofilm induced	-1.92428	0.002976
orf19.2511.2	Ortholog of C. parapsilosis CDC317 : CPAR2_103040, Candida tenuis NRRL Y-1498 : CANTEDRAFT_116324, Debaryomyces hansenii CBS767 : DEHA2G01980g and Pichia stipitis Pignal : PICST_45936 (1)	-1.93256	0.002853
LPG20	Aldo-keto reductase family protein; similar to aryl alcohol dehydrogenases; osmotic stress-induced, correlates with overexpression of MDR1 in fluconazole-resistant isolate; stationary phase enriched protein	-1.93267	1.91E-12
orf19.2001	Has domain(s) with predicted 2-oxoglutarate-dependent dioxygenase activity, L-ascorbic acid binding, iron ion binding, oxidoreductase activity, acting on paired donors, with incorporation or reduction of molecular oxygen activity	-1.93384	1.02E-09

ERG25	Putative C-4 methyl sterol oxidase; C4-demethylation of ergosterol biosynthesis intermediates, based on similarity to S. cerevisiae Erg25; fluconazole-induced; induced in azole-resistant strain; rat catheter and Spider biofilm induced	-1.93385	7.03E-19
orf19.3697	Ortholog(s) have role in mitotic intra-S DNA damage checkpoint signaling, positive regulation of macroautophagy, re-entry into mitotic cell cycle after pheromone arrest and Golgi trans cisterna, endoplasmic reticulum localization	-1.93805	2.06E-07
ADH1	Alcohol dehydrogenase; oxidizes ethanol to acetaldehyde; at yeast cell surface; immunogenic in humans/mice; complements S. cerevisiae adh1 adh2 adh3 mutant; fluconazole, farnesol-induced; flow model biofilm induced; Spider biofilm repressed	-1.94211	1.17E-12
OSM2	Putative mitochondrial fumarate reductase; regulated by Ssn6p, Gcn2p, and Gcn4p; Hog1p-downregulated; stationary phase enriched protein; Hap43p-repressed gene	-1.94326	1.18E-15
orf19.4117	Ortholog of C. dubliniensis CD36 : Cd36_20450, C. parapsilosis CDC317 : CPAR2_104160, C. auris B8441 : B9J08_001214 and Candida tenuis NRRL Y-1498 : CANTEDRAFT_95516	-1.94956	4.45E-12
GLK1	Putative glucokinase; transcript regulated upon yeast-hyphal switch; Efg1 regulated; fluconazole-induced; induced in core stress response; colony morphology-related gene regulation by Ssn6; GlcNAc-induced protein	-1.95178	1.43E-14
orf19.836	Protein likely to be essential for growth, based on an insertional mutagenesis strategy	-1.95594	1.73E-08
orf19.697	Ortholog of S. cerevisiae : YSC83, C. glabrata CBS138 : CAGL0A02134g, C. dubliniensis CD36 : Cd36_32160, C. parapsilosis CDC317 : CPAR2_203070 and C. auris B8441 : B9J08_004564	-1.9584	3.04E-14
orf19.5129	Ortholog of C. dubliniensis CD36 : Cd36_72910, C. parapsilosis CDC317 : CPAR2_704100, C. auris B8441 : B9J08_002713 and Candida tenuis NRRL Y-1498 : CANTEDRAFT_113193	-1.96251	3.23E-11
orf19.3447	Ortholog(s) have mannosyltransferase activity, role in GPI anchor biosynthetic process, protein processing, ubiquitin-dependent ERAD pathway and endoplasmic reticulum, glycosylphosphatidylinositol-mannosyltransferase I complex localization	-1.96879	3.34E-10

ISY1	Putative pre-mRNA-splicing factor; mutation confers resistance to 5-fluorocytosine (5-FC); rat catheter biofilm induced	-1.97164	6.03E-05
QCE1	Mitochondrial protein required for expression of respiratory chain complex III (coenzyme Q:cytochrome c oxidoreductase); Hap43-induced gene; Spider biofilm induced	-1.97744	7.32E-08
ABP2	Putative alpha-actinin-like protein; induced by alpha pheromone in SpiderM medium	-1.98612	1.54E-07
orf19.2433	Has domain(s) with predicted flap-structured DNA binding activity and role in double-strand break repair via single-strand annealing, removal of nonhomologous ends	-1.98712	0.010208
VMA13	Predicted proton-transporting ATPase; predicted role in ATP hydrolysis coupled proton transport; rat catheter biofilm repressed	-1.99083	7.93E-17
orf19.3325	Putative glycogen synthesis initiator; regulated by Efg1 and Efh1; Hog1-repressed; colony morphology-related gene regulation by Ssn6; induced by prostaglandins; flow model biofilm induced	-1.99354	2.73E-08
MRT4	Putative mRNA turnover protein; Hap43-induced; mutation confers hypersensitivity to tubercidin (7-deazaadenosine); rat catheter biofilm induced	-2.00524	2.74E-13
CGR1	Negative regulator of yeast-form growth; HSP70 family member; induced by growth cessation at yeast-hyphal transition or in planktonic growth; physically interacts with Msi3p; similar to rat anti-aging gene, SMP30, stationary phase enriched	-2.00704	2.34E-16
orf19.4913	Ortholog(s) have ubiquitin binding activity	-2.00793	4.52E-05
TOM22	Putative mitochondrial import receptor subunit; colony morphology-related gene regulation by Ssn6	-2.00853	4.44E-13
orf19.6862	Hap43-induced gene; mutation confers hypersensitivity to 5-fluorocytosine (5-FC), 5-fluorouracil (5-FU), and tubercidin (7-deazaadenosine)	-2.00884	5.42E-14
CWH8	Putative dolichyl pyrophosphate (Dol-P-P) phosphatase; ketoconazole-induced; expression is increased in a fluconazole-resistant isolate; clade-associated gene expression; Hap43p-induced gene	-2.00918	1.16E-16

orf19.6656	Spermidine transporter; induced in strains from HIV patients with oral candidiasis; alkaline repressed; amphotericin B induced; colony morphology regulated by Ssn6; reduced oral epithelial cell damage by mutant; Spider biofilm induced	-2.01016	7.38E-15
orf19.1748	Protein of unknown function; shows colony morphology-related gene regulation by Ssn6	-2.01441	0.001175
orf19.2541	Ortholog(s) have 3'-5'-exodeoxyribonuclease activity, endonuclease activity and role in apoptotic DNA fragmentation, cellular response to oxidative stress	-2.01923	2.49E-08
orf19.2397.3	Putative aminotransferase; Hap43-repressed; homozygous Tn insertion decreases colony wrinkling in filament-inducing conditions, does not block true hypha formation in liquid media; rat catheter and Spider biofilm induced	-2.02238	3.09E-11
orf19.7443	Ortholog of C. dubliniensis CD36 : Cd36_86510, C. parapsilosis CDC317 : CPAR2_206110, C. auris B8441 : B9J08_002384 and Candida tenuis NRRL Y-1498 : CANTEDRAFT_112184	-2.02285	4.49E-11
DPP3	Protein similar to S. cerevisiae pyrophosphate phosphatase Dpp1; required for farnesol biosynthesis; repressed by 17-beta-estradiol, ethynyl estradiol; Spider biofilm induced	-2.02498	1.48E-16
orf19.2642	Ortholog(s) have myosin II heavy chain binding activity, role in mitotic actomyosin contractile ring disassembly and cellular bud neck contractile ring, incipient cellular bud site, myosin II complex localization	-2.0269	1.97E-05
DIE2	Ortholog(s) have dolichyl pyrophosphate Glc2Man9GlcNAc2 alpha-1,2-glucosyltransferase activity, role in protein N-linked glycosylation and endoplasmic reticulum membrane localization	-2.0324	2.02E-09
orf19.4639	Protein present in exponential and stationary growth phase yeast cultures	-2.04794	4.93E-14
orf19.4191.1	Ortholog(s) have ubiquitin-ubiquitin ligase activity and role in free ubiquitin chain polymerization, protein polyubiquitination, ubiquitin-dependent ERAD pathway, ubiquitin-dependent protein catabolic process	-2.05501	1.69E-10
orf19.6787	Ortholog(s) have cargo receptor activity and role in ascospore formation, axial cellular bud site selection, endoplasmic reticulum to Golgi vesicle-mediated transport	-2.05746	2.13E-10

CHT3	Major chitinase; secreted; functional homolog of S. cerevisiae Cts1p; 4 N-glycosylation motifs; possible O-mannosylation; putative signal peptide; hyphal-repressed; farnesol upregulated in biofilm; regulated by Efg1p, Cyr1p, Ras1p	-2.05827	1.11E-12
orf19.4748	Putative U2B'' component of the U2 snRNP, involved in splicing; contains an RNA recognition motif (RRM); ortholog of S. cerevisiae MSL1; Hap43p-induced gene	-2.06001	0.000382
orf19.4465	Protein of unknown function; Spider biofilm induced	-2.06635	9.48E-08
orf19.5291	Ortholog(s) have role in phospholipid biosynthetic process, phospholipid metabolic process, postreplication repair and endoplasmic reticulum localization	-2.06931	5.1E-14
GIG1	Protein induced by N-acetylglucosamine (GlcNAc); localized in cytoplasm; mutation causes increased resistance to nikkomycin Z	-2.07722	3.52E-05
orf19.305	Protein of unknown function; upregulated in a cyr1 null mutant	-2.07775	4.64E-17
DIP5	Dicarboxylic amino acid permease; mutation confers hypersensitivity to toxic ergosterol analog; induced upon phagocytosis by macrophage; Gcn4-regulated; upregulated by Rim101 at pH 8; rat catheter and Spider biofilm induced	-2.10212	1.74E-17
CDC19	Pyruvate kinase at yeast cell surface; Gcn4/Hog1/GlcNAc regulated; Hap43/polystyrene adherence induced; repressed by phagocytosis/farnesol; hyphal growth role; stationary phase enriched; flow model biofilm induced; Spider biofilm repressed	-2.10945	2.77E-12
orf19.7365	Ortholog(s) have ubiquitin-protein transferase activity and role in negative regulation of apoptotic process, negative regulation of gluconeogenesis, proteasome-mediated ubiquitin-dependent protein catabolic process	-2.11042	2.86E-06
orf19.1229	Ortholog(s) have role in protein export from nucleus, snRNA import into nucleus and nuclear envelope, nuclear periphery, nucleus localization	-2.11174	4.32E-12
PLB1	Phospholipase B; host cell penetration and virulence in mouse systemic infection; Hog1-induced; signal sequence, N-glycosylation, and Tyr phosphorylation site; induced in fluconazole-resistant strains; rat catheter biofilm repressed	-2.11548	0.004268

orf19.6920	Protein of unknown function; induced during chlamydospore formation in both C. albicans and C. dubliniensis; Hap43-induced gene; Spider biofilm induced; F-12/CO2 early biofilm induced	-2.11757	0.001061
orf19.5701	Ortholog(s) have role in DNA replication initiation, chromosome segregation, establishment of mitotic sister chromatid cohesion, mitotic spindle assembly checkpoint signaling	-2.12526	1.48E-05
LYS22	Homocitrate synthase, minor isoform; repressed by nitric oxide and by hypoxia; protein level decreases in stationary phase cultures; induced by ketoconazole, Spider biofilm induced; flow model biofilm repressed	-2.12639	2.31E-21
RPC19	Putative RNA polymerases I and III subunit AC19; Hap43-induced; rat catheter biofilm induced	-2.12869	2.16E-11
AXL1	Putative endoprotease; induced by alpha factor; transcript is upregulated in an RHE model of oral candidiasis and in clinical isolates from HIV+ patients with oral candidiasis	-2.14136	4.9E-13
orf19.3799	Ortholog(s) have role in ER-dependent peroxisome organization, endoplasmic reticulum inheritance, endoplasmic reticulum tubular network maintenance and endoplasmic reticulum tubular network organization, more	-2.14225	8.81E-25
ECM1	Putative pre-ribosomal factor; decreased mRNA abundance observed in cyr1 homozygous mutant hyphae; induced by heavy metal (cadmium) stress; Hog1p regulated	-2.14237	5.22E-09
orf19.3430	Plasma membrane-associated protein; physically interacts with TAP-tagged Nop1p	-2.14273	5.79E-11
OPT3	Oligopeptide transporter; transcript induced by macrophage phagocytosis, BSA or peptides; fluconazole-induced; induced by Rim101 at pH 8; virulence-group-correlated expression; Hap43-repressed; Spider biofilm induced	-2.14419	1.32E-15
orf19.810	Ortholog(s) have DNA helicase activity, heteroduplex DNA loop binding activity	-2.14522	3.23E-10
FRP1	Ferric reductase; alkaline-induced by Rim101; iron-chelation-induced by CCAAT-binding factor; fluconazole-repressed; ciclopirox-, hypoxia-, Hap43-induced; colony morphology-related regulation by Ssn6; Spider and flow model biofilm induced	-2.15562	5.58E-14

TEN1	Histidine kinase involved in a two-component signaling pathway that regulates cell wall biosynthesis; mutants are sensitive to growth on H2O2 medium; rat catheter and Spider biofilm induced	-2.16515	0.00821
orf19.6597	Ortholog of S. cerevisiae : YLR287C, C. glabrata CBS138 : CAGL0M09757g, C. dubliniensis CD36 : Cd36_34830, C. parapsilosis CDC317 : CPAR2_701110 and C. auris B8441 : B9J08_000560	-2.17024	1.04E-15
orf19.3250	Ortholog(s) have RNA binding activity, role in mRNA splicing, via spliceosome and U1 snRNP, U2-type prespliceosome, nucleus localization	-2.17478	5.2E-10
orf19.3916	Ortholog of C. dubliniensis CD36 : Cd36_53980, C. parapsilosis CDC317 : CPAR2_100980, C. auris B8441 : B9J08_004358 and Candida tenuis NRRL Y-1498 : CANTEDRAFT_116966	-2.17595	1.5E-10
orf19.3364	Ortholog of C. parapsilosis CDC317 : CPAR2_403360, Debaryomyces hansenii CBS767 : DEHA2D00814g, Pichia stipitis Pignal : PICST_32156 and Candida guilliermondii ATCC 6260 : PGUG_04611	-2.18552	3.33E-10
orf19.2888	Ortholog(s) have role in cellular protein-containing complex assembly, early endosome to Golgi transport and Golgi apparatus, TRAPPII protein complex localization	-2.18898	1.55E-12
orf19.2728	Ortholog(s) have DNA clamp loader activity, role in DNA damage checkpoint signaling, nucleotide-excision repair, reciprocal meiotic recombination and Rad17 RFC-like complex localization	-2.19692	7.46E-09
orf19.55	Ortholog(s) have role in cellular zinc ion homeostasis	-2.20756	2.06E-20
orf19.7022	Ortholog of C. dubliniensis CD36 : Cd36_70850, C. auris B8441 : B9J08_003277, Candida tenuis NRRL Y-1498 : cten_CGOB_00183 and Debaryomyces hansenii CBS767 : DEHA2F22462g	-2.20957	4.46E-12
orf19.3585	Putative tRNA methyltransferase complex subunit; regulated by Gcn4; repressed in response to amino acid starvation (3-aminotriazole treatment); Spider biofilm induced	-2.21944	1.05E-10
orf19.6699	Ortholog(s) have histidinol-phosphatase activity and role in histidine biosynthetic proces	-2.23122	3.13E-16
DAD3	Subunit of the Dam1 (DASH) complex, which acts in chromosome segregation by coupling kinetochores to spindle microtubules	-2.23148	2.33E-05

SEN15	Similar to delta subunit of tRNA splicing endonuclease; increased transcription is observed upon fluphenazine treatment	-2.24384	4.57E-09
orf19.773	Protein similar to S. cerevisiae Rsa3 predicted nucleolar protein involved in maturation of pre-60S ribosomal particles; rat catheter and Spider biofilm induced	-2.2491	2.91E-07
orf19.4612	Protein with a dienelactone hydrolase domain; Hap43-repressed gene	-2.26383	2.48E-11
TPS3	Predicted trehalose-phosphate synthase regulatory subunit; regulated by Efg1; regulated by Tsa1, Tsa1B under H2O2 stress conditions; flow model biofilm induced (reports differ)	-2.26984	2.41E-13
orf19.1841	Protein of unknown function; Hap43-induced gene	-2.28235	2.2E-08
RNR2	Putative ribonucleoside diphosphate reductase;colony morphology-related gene regulation by Ssn6; transcript regulated by tyrosol and cell density; Hap43-repressed; Spider biofilm induced	-2.28928	1.35E-12
RHB1	Putative small G protein from the Ras superfamily involved in cell wall integrity and control of filamentous growth under nitrogen starvation; involved in activation of TOR1C during starvation response	-2.30412	8.41E-13
orf19.3690.2	Ribosomal 60S subunit protein; Spider biofilm repressed	-2.30884	1.18E-12
AHP1	Alkyl hydroperoxide reductase; immunogenic; fluconazole-induced; amphotericin B, caspofungin, alkaline repressed; core stress response induced; Ssk1/Nrg1/Tup1/Ssn6/Hog1 regulated; flow model biofilm induced; rat catheter biofilm repressed	-2.31093	1.16E-26
SAC1	Putative phosphatidylinositol phosphate (PtdInsP) phosphatase, involved in cell wall integrity and morphogenesis	-2.31737	2.23E-17
MNN24	Alpha-1,2-mannosyltransferase; required for normal cell wall mannan content	-2.31972	5.51E-18
orf19.1708	Protein of unknown function; Spider biofilm induced (5, 6)	-2.32754	9.52E-06
orf19.3437	Has domain(s) with predicted serine-type endopeptidase activity and role in proteolysis	-2.33065	6.54E-07

BBC1	Putative SH3-domain-containing protein	-2.33559	5.02E-10
orf19.7051	Maf-like protein; rat catheter biofilm induced	-2.34343	2.87E-09
orf19.1606	Protein of unknown function; Plc1-regulated	-2.34375	1.76E-06
GRE3	Putative D-xylose reductase; antigenic in murine systemic infection; soluble protein in hyphae; induced by farnesol, macrophage interaction and by Mnl1 under weak acid stress; stationary-phase enriched protein; Spider biofilm induced	-2.35444	2.02E-26
orf19.2939	Ortholog(s) have role in mitochondrial cytochrome c oxidase assembly and mitochondrion localization	-2.36342	1.83E-09
orf19.3686	Ortholog(s) have protein domain specific binding activity, role in mitochondrial proton-transporting ATP synthase complex assembly and mitochondrion localization	-2.3682	7.65E-14
OSM1	Putative flavoprotein subunit of fumarate reductase; soluble protein in hyphae; caspofungin repressed; stationary phase enriched protein; flow model biofilm induced; Spider biofilm repressed	-2.37983	2.62E-18
RPN12	Subunit of the 19S regulatory particle lid of the proteasome	-2.38216	2.52E-17
MED8	Ortholog(s) have RNA polymerase II cis-regulatory region sequence-specific DNA binding, TBP-class protein binding, protein-macromolecule adaptor activity, transcription corepressor activity	-2.38348	3.37E-09
CDR4	Putative ABC transporter superfamily; fluconazole, Sfu1, Hog1, core stress response induced; caspofungin repressed; fluconazole resistance not affected by mutation or correlated with expression; rat catheter and flow model biofilm induced	-2.38567	9.71E-15
VMA5	Putative vacuolar H(+)-ATPase; plasma membrane localized; rat catheter biofilm repressed	-2.3862	2.05E-22
orf19.6264.3	Ortholog(s) have role in endoplasmic reticulum to Golgi vesicle-mediated transport and COPII-coated ER to Golgi transport vesicle, Golgi apparatus, endoplasmic reticulum localization	-2.39927	5.48E-19

SEC5	Predicted exocyst component; ortholog of S. cerevisiae Sec5p; merged with orf19.75 in Assembly 21	-2.40141	1.26E-12
orf19.1484	Ortholog of S, cerevisiae Spc24; a component of the conserved kinetochore-associated Ndc80 complex involved in chromosome segregation, spindle checkpoint activity and kinetochore clustering; Hap43-induced gene	-2.40447	0.002616
GLC3	Putative 1,4-glucan branching enzyme; fluconazole-induced; colony morphology-related gene regulation by Ssn6; stationary phase enriched protein	-2.40781	2.65E-19
CSP37	Hyphal cell wall protein; role in progression of mouse systemic infection; predicted P-loop, divalent cation binding, N-glycosylation sites; expressed in yeast and hyphae; hyphal downregulated; stationary-phase enriched; GlcNAc-induced	-2.41694	9.47E-25
QDR1	Putative antibiotic resistance transporter; regulated by white-opaque switch, Nrg1, Tup1; Hap43, caspofungin repressed; repressed during chlamydospore formation; flow model biofilm induced; Spider biofilm repressed	-2.42039	6.9E-20
SAM50	Predicted component of the SAM complex involved in mitochondrial protein import	-2.42963	1.13E-19
orf19.5003	Ortholog(s) have GTPase regulator activity and cytoplasm, nucleus localization	-2.43032	4.78E-13
HEM14	Putative protoporphyrinogen oxidase; involved in heme biosynthesis; predicted Kex2p substrate; iron regulated transcript; Yfh1-induced; Hap43-repressed; rat catheter biofilm repressed	-2.43452	7.78E-15
OPT2	Oligopeptide transporter; induced upon phagocytosis by macrophage; macrophage/pseudohyphal-repressed after 16h; fluconazole-induced; virulence-group-correlated expression; Hap43-repressed	-2.4366	1.07E-06
BUD16	Putative pyridoxal kinase; a key enzyme in pyridoxal 5'-phosphate synthesis, the active form of vitamin B6; involved in bud-site selection and genome integrity in S. cerevisiae; induced by alpha pheromone in SpiderM medium	-2.43706	3.68E-11
orf19.3990	Ortholog(s) have DNA-directed 5'-3' RNA polymerase activity, RNA polymerase III activity and role in tRNA transcription by RNA polymerase III, transcription initiation from RNA polymerase III promoter	-2.43987	3.79E-08

orf19.4699	Putative phospholipase of patatin family; similar to S. cerevisiae Tgl3p; predicted Kex2p substrate	-2.44287	1.01E-15
orf19.104	Protein of unknown function; induced by alpha pheromone in SpiderM medium	-2.44392	0.012406
orf19.1121	Ortholog of C. dubliniensis CD36 : Cd36_53470, Candida tenuis NRRL Y-1498 : CANTEDRAFT_93324, Candida tropicalis NEW ASSEMBLY : CTRG1_05538 and Spathaspora passalidarum NRRL Y-27907 : SPAPADRAFT_55715	-2.44478	2.33E-16
MRP7	Mitochondrial ribosomal protein of the large subunit; rat catheter biofilm induced	-2.46505	1.65E-16
MNT4	Predicted alpha-1,3-mannosyltransferase with a role in protein glycosylation; induced in low iron	-2.47089	8.73E-08
RTT101	Putative cullin subunit of E3 ubiquitin ligase complex, involved in response to DNA damage; induced by alpha pheromone in SpiderM medium	-2.4736	1.24E-06
AAT22	Aspartate aminotransferase; nitrogen metabolism; similar but not orthologous to S. cerevisiae Aat2; clade-associated gene expression; protein levels decrease in stationary phase yeast; mutant is viable; flow model biofilm repressed	-2.4835	1.13E-25
OP4	Ala- Leu- and Ser-rich protein; secreted; N-terminal hydrophobic region; possible glycosylation; opaque-specific transcript; repressed by alpha pheromone in opaque MTLa homozygotes; fluconazole-induced; Spider biofilm induced	-2.48556	3.27E-07
CRG1	Methyltransferase involved in sphingolipid homeostasis, methylates a drug cantharidin; decreased expression in hyphae compared to yeast; expression regulated during planktonic growth; flow model biofilm induced; Hap43-repressed gene	-2.48711	1.58E-18
XKS1	Putative xylulokinase; Hap43-repressed; induced by prostaglandins; rat catheter biofilm repressed	-2.48854	5.8E-19
orf19.252	Protein of unknown function; S. cerevisiae ortholog Fmp37 which localizes to mitochondria; Hap43-repressed; Spider biofilm repressed	-2.49006	2.01E-15
orf19.1314	Protein of unknown function; planktonic growth-induced gene	-2.49096	1.18E-14

orf19.6822	Ortholog(s) have NEDD8 transferase activity, cullin family protein binding, protein-macromolecule adaptor activity, ubiquitin conjugating enzyme binding, ubiquitin-like protein binding activity	-2.49507	2.97E-12
OPT4	Oligopeptide transporter; detected at germ tube plasma membrane; transcript inducede during phagocytosis by macrophages; fungal-specific; Hap43-repressed; merged with orf19.2292 in Assembly 20; rat catheter and Spider biofilm induced	-2.4981	1.85E-15
YKE2	Possible heterohexameric Gim/prefoldin protein complex subunit; role in folding alpha-tubulin, beta-tubulin, and actin; transcript induced by yeast-to-hypha switch; regulated by Nrg1, Tup1; Spider and flow model biofilm induced	-2.49866	2.79E-16
RPL35	Ribosomal protein; downregulation correlates with clinical development of fluconazole resistance; colony morphology-related gene regulation by Ssn6; Hap43-induced; Spider biofilm repressed	-2.5033	2.14E-15
orf19.1336.2	Ortholog(s) have role in mitochondrial respiratory chain complex assembly and mitochondrial intermembrane space localization	-2.50505	2.11E-06
orf19.2514	Predicted methyltransferase; rat catheter biofilm induced	-2.50791	4.93E-12
GSG1	Putative subunit of the TRAPP complex; involved in targeting of ER-to-Golgi transport vesicles; flow model biofilm induced	-2.51543	4.28E-10
orf19.3978	Protein required for maturation of 18S rRNA; rat catheter biofilm repressed	-2.51896	1.02E-15
ERG28	Ortholog(s) have protein-macromolecule adaptor activity, role in demethylation, ergosterol biosynthetic process and endoplasmic reticulum membrane localization	-2.51949	1.97E-19
orf19.3698	Protein of unknown function that may function in RNA processing; filament induced	-2.52819	3.12E-12
orf19.2828	Ortholog(s) have alpha-tubulin binding, microtubule binding activity, role in cytoplasmic microtubule organization, post-chaperonin tubulin folding pathway, protein folding and cytoplasm localization	-2.53098	7.51E-17
orf19.6966	Ortholog(s) have choline kinase activity, ethanolamine kinase activity and role in phosphatidylcholine	-2.54772	1.68E-18

	biosynthetic process, phosphatidylethanolamine biosynthetic process		
orf19.4595	Ortholog of C. dubliniensis CD36 : Cd36_41860, C. parapsilosis CDC317 : CPAR2_400440, C. auris B8441 : B9J08_002205 and Candida tenuis NRRL Y-1498 : CANTEDRAFT_103033	-2.54944	7.73E-20
FGR24	Protein encoded in retrotransposon Zorro2 with similarity to retroviral endonuclease-reverse transcriptase proteins; lacks an ortholog in S. cerevisiae; transposon mutation affects filamentous growth	-2.55005	3.29E-15
HRT2	Protein described as having a role in Ty3 transposition; repressed in hyphae; stationary phase enriched protein; rat catheter and Spider biofilm repressed	-2.55752	2E-20
orf19.2123	Predicted ORF from Assembly 19; removed from Assembly 20; restored based on comparative genome analysis	-2.56792	0.001337
SUR2	Putative ceramide hydroxylase; predicted enzyme of sphingolipid biosynthesis; regulated by Tsa1, Tsa1B under H2O2 stress conditions; Spider and flow model biofilm induced	-2.58074	9.87E-30
orf19.5125	Protein of unknown function; induced by ketoconazole; Spider, F-12/CO2 and flow model biofilm induced	-2.58496	9.86E-09
orf19.6788	Protein with a predicted role in cotranslational protein targeting to membrane; induced during chlamydospore formation in both C. albicans and C. dubliniensis	-2.59157	7.67E-14
orf19.6458.1	Ortholog(s) have U6 snRNA binding activity and role in P-body assembly, mRNA splicing, via spliceosome	-2.59448	1.1E-11
LYP1	Putative permease; amphotericin B induced; flucytosine repressed; possibly an essential gene, disruptants not obtained by UAU1 method	-2.59626	6.73E-08
orf19.5227	Chaperone component; involved in assembly of alpha subunits into the 20S proteasome; flow model biofilm induced	-2.60468	4.07E-10
orf19.1590	Ortholog of C. dubliniensis CD36 : Cd36_18970, C. parapsilosis CDC317 : CPAR2_212800, Debaryomyces hansenii CBS767 : DEHA2F08690g and Candida guilliermondii ATCC 6260 : PGUG_01488	-2.60739	1.12E-07
orf19.1296	Ortholog(s) have role in spliceosomal snRNP assembly and U4/U6 x U5 tri-snRNP complex localization	-2.61344	2.45E-09

CAN2	Basic amino acid permease; arginine metabolism; regulated by Nrg1/Tup1; caspofungin, flucytosine induced; colony morphology-related regulation by Ssn6; Hap43-repressed; rat catheter and Spider biofilm induced; promoter bound by Efg1	-2.62471	6.37E-24
ACP1	Putative mitochondrial acyl carrier protein involved in fatty acid biosynthesis; shows colony morphology-related gene regulation by Ssn6p; protein newly produced during adaptation to the serum	-2.62781	1.68E-20
orf19.3214	Alpha/beta-HydrolasE superfamily protein; membrane-localized	-2.62874	1.26E-10
orf19.119	Ortholog(s) have protein C-terminal S-isoprenylcysteine carboxyl O-methyltransferase activity	-2.63377	0.007952
orf19.3572	Ortholog of C. dubliniensis CD36 : Cd36_19880, C. auris B8441 : B9J08_003635, Candida tenuis NRRL Y-1498 : CANTEDRAFT_117672 and Debaryomyces hansenii CBS767 : DEHA2D11154g	-2.63488	9.83E-11
orf19.68.2	Ortholog of C. dubliniensis CD36 : Cd36_11670, C. parapsilosis CDC317 : CPAR2_201750, C. auris B8441 : B9J08_003474 and Candida tenuis NRRL Y-1498 : CANTEDRAFT_115679	-2.64178	6.52E-05
orf19.7297	Putative cystathionine gamma-synthase; decreased levels in stationary phase cultures; Hog1p-induced; Gcn4p-regulated	-2.64373	7.46E-25
orf19.6359	Ortholog(s) have role in mitochondrial respiratory chain complex assembly, proteolysis and mitochondrial inner membrane, mitochondrial inner membrane peptidase complex localization	-2.65513	1.6E-06
orf19.1276	Protein with a selenoprotein domain and a thioredoxin-like fold domain; similar to S. cerevisiae Vhs3p, which is a putative phosphopantothenoylcysteine decarboxylase	-2.65527	5.61E-09
PRO2	Putative gamma-glutamyl phosphate reductase with a predicted role in proline biosynthesis; regulated by Gcn2p and Gcn4p	-2.66495	6.12E-29
orf19.154	Putative ortholog of S. cerevisiae Utp30; a U3-containing 90S preribosome complex protein; Hap43-induced; Spider biofilm induced	-2.67265	3.97E-07
orf19.1266	Protein of unknown function; transcript detected on high-resolution tiling arrays	-2.68096	2.72E-06

YWP1	Secreted yeast wall protein; possible role in dispersal in host; involved in adhesion and biofilm formation; propeptide; growth phase, phosphate, Ssk1/Ssn6/Efg1/Efh1/Hap43 regulated; mRNA binds She3; flow and Spider biofilm repressed	-2.68971	7.92E-26
orf19.6480	Ortholog(s) have role in UDP-glucose transmembrane transport	-2.70923	6.63E-15
orf19.6346	Putative nuclear export protein; Hap43p-induced gene; decreased transcription is observed in an azole-resistant strain that overexpresses MDR1	-2.71777	1.2E-19
RME1	Zinc finger protein, controls asexual sporulation; white-specific transcript; upregulation correlates with clinical development of fluconazole resistance; Upc2-regulated in hypoxia; flow model biofilm induced; Spider biofil	-2.71886	3.49E-09
orf19.4340.1	Ortholog(s) have poly(U) RNA binding, splicing factor binding activity and U1 snRNP, U2 snRNP, U4/U6 x U5 tri-snRNP complex, U5 snRNP, post-mRNA release spliceosomal complex, spliceosomal complex localization	-2.71958	3.85E-08
DSL1	Protein similar to S. cerevisiae Dsl1p, which is a member of the t-SNARE complex of the endoplasmic reticulum	-2.73555	2.42E-19
orf19.6804	Ortholog(s) have role in SRP-dependent cotranslational protein targeting to membrane and signal recognition particle, endoplasmic reticulum targeting localization	-2.76563	1.29E-20
ATX1	Putative cytosolic copper metallochaperone; flucytosine induced; Ssr1-repressed; rat catheter biofilm induced	-2.77567	0.000874
orf19.2733	Putative subunit of phosphatidylinositol 3-kinase complexes I and II; transcription is activated in the presence of elevated CO2	-2.7788	2.72E-15
orf19.3351	Protein of unknown function; Hap43-induced; Spider biofilm induced	-2.78675	2.9E-09
AAT1	Aspartate aminotransferase; soluble protein in hyphae; macrophage-induced protein; alkaline upregulated; amphotericin B repressed; gene used for strain identification by multilocus sequence typing; farnesol-, Hap43p-induced; GlcNAc-induced	-2.7882	4.46E-32
ATP7	Putative subunit of the F1F0-ATPase complex; colony morphology-related gene regulation by Ssn6; farnesol, macrophage-downregulated protein abundance; protein	-2.79583	1.94E-21

	present in exponential and stationary yeast growth phases; Hap43-induced		
orf19.7032	Ortholog of C. dubliniensis CD36 : Cd36_70790, C. parapsilosis CDC317 : CPAR2_301530, C. auris B8441 : B9J08_005266 and Candida tenuis NRRL Y-1498 : CANTEDRAFT_94947	-2.8096	2.54E-19
orf19.5066	Putative pre-60S pre-ribosomal particle subunit; essential gene; S. cerevisiae ortholog RRP17 is essential; Hap43p-induced; rat catheter biofilm induced	-2.812	4.01E-13
orf19.120	Has domain(s) with predicted protein C-terminal S-isoprenylcysteine carboxyl O-methyltransferase activity, role in C-terminal protein methylation and integral component of membrane localization	-2.81876	1.71E-05
FCY21	High affinity, high capacity, hypoxanthine-adenine-guanine-cytosine/H+ symporter; similar to S. cerevisiae Fcy2; mutation confers resistance to 5-fluorocytosine (5-FC); flow model biofilm induced	-2.82056	2.35E-30
NIP1	Putative translation initiation factor; mutation confers hypersensitivity to roridin A and verrucarin A; genes encoding ribosomal subunits, translation factors, and tRNA synthetases are downregulated upon phagocytosis by murine macrophage	-2.83051	2.17E-18
QCR7	Putative ubiquinol-cytochrome-c reductase, subunit 7; Hap43p-repressed gene	-2.83052	5.85E-20
ARA1	D-Arabinose dehydrogenase; dehydro-D-arabinono-1,4-lactone synthesis; active on D-arabinose, L-fucose, L-xylose, L-galactose; inhibited by metal ions, thiol group-specific reagents; induced on polystyrene adherence; Spider biofilm induced	-2.83165	3.23E-17
orf19.6989	Putative U2 snRNP-associated protein; Spider biofilm induced (5)	-2.85244	0.004603
DAD1	Subunit of the Dam1 (DASH) complex, which acts in chromosome segregation by coupling kinetochores to spindle microtubules	-2.8585	0.001921
orf19.6315	Ortholog of C. dubliniensis CD36 : Cd36_30140, C. parapsilosis CDC317 : CPAR2_204040, C. auris B8441 : B9J08_001978 and Candida tenuis NRRL Y-1498 : CANTEDRAFT_114703	-2.87915	2.17E-16
orf19.1427	Putative transporter; fungal-specific; Spider biofilm induced	-2.87928	5.53E-33

FRE9	Ferric reductase; alkaline induced; ciclopirox olamine; Hap43-induced	-2.8807	0.004357
PGA45	Putative GPI-anchored cell wall protein; repressed in core caspofungin response; Hog1-induced; regulated by Ssn6; Mob2-dependent hyphal regulation; flow model biofilm induced	-2.89307	2.07E-34
RPB8	Putative subunit of RNA polymerases I, II, and III; regulated by Gcn4p; repressed in response to amino acid starvation (3-aminotriazole treatment); heterozygous null mutant exhibits resistance to parnafungin	-2.89787	1.59E-18
ADH5	Putative alcohol dehydrogenase; regulated by white-opaque switch; fluconazole-induced; antigenic in murine infection; regulated by Nrg1, Tup1; Hap43, macrophage repressed, flow model biofilm induced; Spider biofilm induced	-2.89808	1.02E-25
orf19.2928	Has domain(s) with predicted role in retrograde vesicle-mediated transport, Golgi to endoplasmic reticulum and integral component of membrane localization	-2.90698	3.67E-29
ZCF14	Putative Zn(II)2Cys6 transcription factor; caspofungin induced	-2.91796	2.97E-07
ACB1	Protein similar to a region of acyl-coenzyme-A-binding protein; amphotericin B repressed; regulated by Nrg1, Tup1; Hap43-induced	-2.92477	5.82E-10
CHO2	Phosphatidyl-ethanolamine N-methyltransferase; fungal-specific (no human or murine homolog); amphotericin B repressed; Hap43p-induced gene	-2.92915	9.83E-29
NUE1	Mitochondrial protein required for expression of mitochondrial respiratory chain complex I (NADH:ubiquinone oxidoreductase)	-2.93675	0.000052
GSY1	Glycogen synthase (UDP glucose/starch glucosyltransferase); transcript repressed by yeast-hyphal switch, Efg1-regulated; strong oxidative stress induced; colony morphology-related regulation by Ssn6; stationary phase enriched	-2.94191	1.47E-20
orf19.1368	Protein of unknown function; flow model biofilm induced; Spider biofilm induced	-2.95282	1.13E-10
orf19.7553	Protein of unknown function; rat catheter biofilm repressed	-2.95318	1.14E-11
orf19.4528	Ortholog(s) have role in vacuolar protein processing	-2.95785	6.62E-23

	Putative alcohol dehydrogenase; yeast-enriched transcript; Efg1-regulated; induced by prostaglandins, Hog1, fluconazole; rat catheter biofilm induced	-2.95932	3.5E-31
orf19.1611	Protein of unknown function; Spider biofilm induced	-2.96462	0.000131
MNE1	Protein required for expression of NADH:ubiquinone oxidoreductase (mitochondrial complex I)	-2.96963	8.75E-06
orf19.6020	Ortholog(s) have Atg8 ligase activity	-2.97012	2.76E-14
orf19.7556	Ortholog of C. dubliniensis CD36 : Cd36_34965, C. parapsilosis CDC317 : CPAR2_200910, C. auris B8441 : B9J08_005167 and Candida tenuis NRRL Y-1498 : CANTEDRAFT_95984	-2.97117	4.7E-11
	Ortholog(s) have role in Golgi to vacuole transport, endosomal transport, vesicle-mediated transport and AP-1 adaptor complex, Golgi apparatus, endosome localization	-2.97546	1.42E-19
orf19.5608	RNA polymerase III subunit; Spider biofilm induced	-2.9822	1.09E-12
orf19.2757	Has domain(s) with predicted role in cell redox homeostasis	-2.99426	6.67E-20
orf19.1054	Ortholog(s) have role in endoplasmic reticulum tubular network membrane organization, nuclear envelope organization, nuclear pore organization	-3.00445	1.04E-32
orf19.1045	Putative U2 snRNP protein; Hap43p-induced gene; mutation confers hypersensitivity to 5-fluorocytosine (5-FC), 5-fluorouracil (5-FU), and tubercidin (7-deazaadenosine)	-3.00749	5.75E-17
orf19.7502	Protein of unknown function; Hap43-induced gene; upregulated in a cyr1 null mutant; Spider biofilm induced	-3.01446	2.75E-23
orf19.3558	Has domain(s) with predicted role in transport and integral component of membrane localization	-3.01867	2.06E-31
orf19.530	Protein with a UV radiation resistance protein/autophagy-related protein 14 domain; Hap43p-induced gene; mutants are viable	-3.02551	1.43E-13
orf19.2516	Has domain(s) with predicted role in cell redox homeostasis	-3.02838	5.06E-09

CSI2	Putative 66S pre-ribosomal particle component; Hap43-induced; essential for growth; transposon mutation affects filamentous growth; Spider biofilm induced	-3.03664	7.48E-27
orf19.3235	Putative F-actin capping protein subunit alpha; possibly an essential gene, disruptants not obtained by UAU1 method	-3.04469	3E-28
ERD1	Putative membrane protein required for the retention of lumenal endoplasmic reticulum proteins; rat catheter biofilm induced	-3.04735	1.71E-17
PGA7	GPI-linked hyphal surface antigen; induced by ciclopirox olamine, ketoconazole, Rim101 at pH 8; Hap43, fluconazole; flow model biofilm induced; Spider biofilm induced; required for RPMI biofilm; Bcr1-induced in a/a biofilm	-3.05159	3.35E-26
orf19.1485	Mitochondrial ribosomal protein of the large subunit; rat catheter biofilm induced	-3.05829	2.09E-12
Mannosyltransferase; transcription elevated in cyr1, chk1, nik1, and sln1 homozygous null mutants; fungal-specific (no human or murine homolog)		-3.06816	7.44E-23
orf19.6342	Ortholog of C. dubliniensis CD36 : Cd36_11980, C. parapsilosis CDC317 : CPAR2_201330, C. auris B8441 : B9J08_003701 and Candida tenuis NRRL Y-1498 : CANTEDRAFT_114815 (5)	-3.08314	0.019425
orf19.4517	Protein of unknown function; Hap43-induced gene	-3.08542	3.96E-28
orf19.6813	Protein of unknown function; Hap43-induced gene	-3.08919	2.55E-13
orf19.6795	Ortholog(s) have enzyme activator activity	-3.09163	0.000102
orf19.335	Sef1p-, Sfu1p-, and Hap43p-regulated gene	-3.10195	1.87E-17
orf19.2371	Putative Gag protein of retrotransposon Tca2; separated by a stop codon from Pol protein orf19.2372; likely translated as single polyprotein that includes Gag, reverse transcriptase, protease, and integrase; rat catheter biofilm repressed	-3.11003	5.56E-23
orf19.3872	Protein of unknown function; oral infection induced; mutants have reduced capacity to damage oral epithelial cells; rat catheter biofilm repressed	-3.12118	2.58E-31

orf19.1729	Putative transcription factor with zinc finger DNA-binding motif	-3.12363	3.87E-10
orf19.7344	Ortholog(s) have DNA binding, chromatin binding, histone deacetylase activity and role in chromosome segregation, gene silencing by RNA, histone deacetylation, negative regulation of transcription by RNA polymerase II	-3.13339	3.56E-13
RPP1	Putative ortholog of S. cerevisiae Rpp1; subunit of both RNase MRP and nuclear RNase P; rat catheter and Spider biofilm induced	-3.14626	1.04E-05
MET8	Putative bifunctional dehydrogenase and ferrochelatase with a predicted role in siroheme biosynthesis; regulated by Gcn2p and Gcn4p	-3.17895	2.17E-27
orf19.50	Ortholog(s) have role in SCF-dependent proteasomal ubiquitin-dependent protein catabolic process, cellular response to DNA damage stimulus, cellular response to methylmercury	-3.18763	3.66E-12
FCY24	Putative transporter; more similar to S. cerevisiae Tpn1, which is a vitamin B6 transporter, than to purine-cytosine permeases; transcription is regulated by Nrg1; Spider biofilm induced	-3.18794	3.05E-41
orf19.5276	Putative nuclear pore-associated protein; Hap43p-induced gene; induced upon low-level peroxide stress; possibly an essential gene, disruptants not obtained by UAU1 method	-3.19089	4.98E-16
mesA	XP_723132.1 Mea1p [Candida albicans SC5314]	-3.19584	4.27E-16
orf19.7202	Ortholog(s) have role in protein retention in ER lumen, retrograde vesicle-mediated transport, Golgi to endoplasmic reticulum	-3.22502	4.62E-23
ZWF1	Glucose-6-phosphate dehydrogenase; antigenic in mice; activity induced by O2 or oxidizing agents H2O2, menadione, macrophage; caspofungin repressed; induced in core stress response; regulated by Gcn2, Gcn4; rat catheter biofilm repressed	-3.23109	3.04E-34
FAD2	Delta-12 fatty acid desaturase, involved in production of linoleic acid, which is a major component of membranes	-3.23232	5.26E-48
MED22	RNA polymerase II mediator complex subunit	-3.23346	4.71E-05

orf19.1367.1	Ortholog of C. parapsilosis CDC317 : CPAR2_407330, C. auris B8441 : B9J08_001010, Candida tenuis NRRL Y-1498 : CANTEDRAFT_114646 and Debaryomyces hansenii CBS767 : DEHA2B05654g	-3.25203	2.41E-05
orf19.2925	Ortholog(s) have role in protein folding, tubulin complex assembly	-3.26832	1.95E-07
orf19.5522	Ortholog of C. dubliniensis CD36 : Cd36_62760, C. parapsilosis CDC317 : CPAR2_601700, C. auris B8441 : B9J08_001639 and Candida tenuis NRRL Y-1498 : CANTEDRAFT_115220	-3.26886	1.72E-16
CDC43	Beta subunit of heterodimeric protein geranylgeranyltransferase type I; GGTase I enzyme binds zinc, is Mg-dependent; Cdc42p is GGTase I substrate	-3.29464	1.26E-09
orf19.2829	Ortholog(s) have role in protein transport	-3.29828	1.24E-07
orf19.5491	Ortholog of C. dubliniensis CD36 : Cd36_20670, C. parapsilosis CDC317 : CPAR2_104720, C. auris B8441 : B9J08_000085 and Candida tenuis NRRL Y-1498 : CANTEDRAFT_103989 (5)	-3.30185	2.42E-05
GUP1	Putative O-acyltransferase with a role in glycerol uptake; functionally complements growth of S. cerevisiae gup1 mutant under salt stress; required for normal ergosterol distribution, hyphal growth, biofilm formation	-3.30567	1.18E-21
HGT6	Putative high-affinity MFS glucose transporter; 20 family members; induced in core stress response; fluconazole, oralpharyngeal candidasis induced; flow model biofilm induced; Spider biofilm induced	-3.31086	4.7E-16
orf19.4953	Putative ATPase; predicted role in ER-associated protein catabolism; induced during chlamydospore formation in both C. albicans and C. dubliniensis; rat catheter biofilm repressed	-3.31143	3.14E-23
orf19.6551	Ortholog(s) have SNAP receptor activity, role in Golgi vesicle transport, vesicle fusion and Golgi medial cisterna, SNARE complex localization	-3.31734	6.65E-35
orf19.6970	Ortholog of C. dubliniensis CD36 : Cd36_85310, C. parapsilosis CDC317 : CPAR2_807370, C. auris B8441 : B9J08_004442 and Candida tenuis NRRL Y-1498 : CANTEDRAFT_115544	-3.3208	2.66E-06
ARC35	Putative ARP2/3 complex subunit; shows colony morphology-related gene regulation by Ssn6p; mutation confers hypersensitivity to cytochalasin D	-3.34907	3.69E-32

orf19.6612	Putative mitochondrial protein; Hap43p-induced gene	-3.35017	1.87E-24
ERG11	Lanosterol 14-alpha-demethylase; cytochrome P450 family; role in ergosterol biosynthesis; target of azole antifungals; may contribute to drug resistance; azole or flow model biofilm induced; drug treated biofilm induced; hypoxia regulated	-3.3519	1.82E-42
LAG1	Putative ceramide synthase component; Hap43p-repressed gene; amphotericin B repressed	-3.36665	7.56E-34
TGL99	Has domain(s) with predicted role in lipid metabolic process	-3.38351	4.1E-23
orf19.3762	Ortholog of C. dubliniensis CD36 : Cd36_11720, C. parapsilosis CDC317 : CPAR2_201890, C. auris B8441 : B9J08_003468 and Candida tenuis NRRL Y-1498 : CANTEDRAFT_91948	-3.39816	3.74E-11
orf19.2369	Ortholog(s) have ATP binding, DNA replication origin binding activity	-3.41188	2.35E-15
MET18	Putative protein with a predicted role in nucleotide excision repair (NER) and RNA polymerase II (RNAP II) transcription; Plc1p-regulated	-3.42631	6.12E-34
orf19.7304	Protein of unknown function; Hap43-induced; transcript induced early in infection of reconstituted human epithelium, while expression of the C. dubliniensis ortholog is not upregulated; mutants are viable; rat catheter biofilm repressed	-3.42921	4.23E-32
GPI14	Catalytic subunit of glycosylphosphatidylinositol-alpha 1,4 mannosyltransferase I, involved in GPI anchor biosynthesis; regulated by Tsa1p, Tsa1Bp under H2O2 stress conditions	-3.42931	5.12E-32
orf19.2237.1	Ortholog of Slx9 required for pre-rRNA processing; associated with 90S pre-ribosome and 43S small ribosomal subunit precursor, interacts with U3 snoRNA in S. cerevisiae; gene has intron; Spider biofilm induced	-3.43519	3.11E-14
UBP8	Putative ubiquitin-specific protease, involved in histone H2B deubiquitination during hyphal development	-3.44113	8.32E-14
SIT1	Transporter of ferrichrome siderophores, not ferrioxamine B; required for human epithelial cell invasion in vitro, not for mouse systemic infection;	-3.47563	2.4E-15

	regulated by iron, Sfu1, Rfg1, Tup1, Hap43; rat catheter and Spider biofilm induced		
orf19.3626	Has domain(s) with predicted asparagine synthase (glutamine-hydrolyzing) activity and role in asparagine biosynthetic process, metabolic process	-3.47723	5.38E-11
orf19.2761	Putative glycosylphosphatidylinositol (GPI) anchor assembly protein; transposon insertion causes decreased colony wrinkling but does not block true hyphal growth; induced by nitric oxide independent of Yhb1p	-3.50209	0.012777
AQY1	Aquaporin water channel; osmotic shock resistance, WT freeze tolerance; virulent in mice; flucytosine repressed; flow model/RPMI/Spider/rat catheter biofilm induced; required for RPMI biofilm formation; Bcr1-induced in a/a RPMI biofilms	-3.5228	3.25E-25
HSM3	Ortholog(s) have role in mismatch repair, proteasome regulatory particle assembly and cytosol, nucleus localization	-3.53315	5.43E-34
SSH1	Protein with a role in protein translocation across membranes	-3.53947	1.75E-35
orf19.5266	Planktonic growth-induced gene	-3.5502	0.002167
RBT5	GPI-linked cell wall protein; hemoglobin utilization; Rfg1, Rim101, Tbf1, Fe regulated; Sfu1, Hog1, Tup1, serum, alkaline pH, antifungal drugs, geldamycin repressed; Hap43 induced; required for RPMI biofilms; Spider biofilm induced	-3.57622	4.1E-43
PSF1	Putative GINS complex subunit which is localized to DNA replication origins; implicated in assembly of the DNA replication machinery; flow model biofilm induced	-3.57927	1.97E-17
PRP39	Putative component of the U1 snRNP; involved in splicing; Hap43-induced gene; Spider biofilm induced	-3.58395	2.17E-19
orf19.2510	Ortholog(s) have role in protein folding, tubulin complex assembly	-3.58922	3.45E-23
orf19.4656	Ortholog of C. dubliniensis CD36 : Cd36_41300, C. parapsilosis CDC317 : CPAR2_400390, C. auris B8441 : B9J08_000767 and Candida tenuis NRRL Y-1498 : CANTEDRAFT_94704	-3.60631	0.00412
orf19.6024	Ortholog of C. dubliniensis CD36 : Cd36_00770, C. auris B8441 : B9J08_004942, Candida tenuis NRRL Y-1498 : CANTEDRAFT_112621 and Debaryomyces hansenii CBS767 : DEHA2D09812g	-3.60724	1.5E-16

BET4	Ortholog(s) have Rab geranylgeranyltransferase activity and role in endoplasmic reticulum to Golgi vesicle-mediated transport, protein geranylgeranylation, protein targeting to membrane	-3.61426	7.6E-15
ERG9	Putative farnesyl-diphosphate farnesyl transferase (squalene synthase); sterol biosynthesis pathway; likely essential for growth; regulated by fluconazole, lovastatin; amphotericin B, caspofungin repressed; Spider biofilm repressed	-3.6232	1.87E-37
BLP1	Protein of unknown function, serum-induced	-3.642	0.000139
NAT4	Putative histone acetyltransferase; involved in regulation of white-opaque switch; early-stage flow model biofilm induced; Spider biofilm induced	-3.67399	1.46E-19
orf19.1789	Ortholog of S. cerevisiae : MRX12, C. glabrata CBS138 : CAGL0J11110g, C. dubliniensis CD36 : Cd36_44970, C. parapsilosis CDC317 : CPAR2_500860 and C. auris B8441 : B9J08_003204	-3.71375	5.18E-27
orf19.6600	Ortholog(s) have phosphatidic acid transfer activity and role in cardiolipin metabolic process, phospholipid translocation, phospholipid transport, positive regulation of phosphatidylcholine biosynthetic process	-3.72632	1.32E-19
orf19.1495	Ortholog(s) have role in L-methionine salvage from methylthioadenosine	-3.77862	4.46E-28
orf19.81	Ortholog(s) have role in exonucleolytic trimming to generate mature 3'-end of 5.8S rRNA from tricistronic rRNA transcript (SSU-rRNA, 5.8S rRNA and LSU-rRNA), more	-3.80987	1.46E-12
PET111	Mitochondrial translational activator specific for the COX2 mRNA, required for expression of mitochondrial respiratory chain complex IV (cytochrome c oxidase); Hap43p-induced gene	-3.81315	1.34E-14
orf19.6358	Ortholog(s) have ubiquitin conjugating enzyme activity, ubiquitin-protein transferase activity and role in free ubiquitin chain polymerization, postreplication repair, protein K63-linked ubiquitination, protein polyubiquitination	-3.81784	4.79E-25
orf19.5621	Putative protein of unknown function; mutation confers hypersensitivity to amphotericin B; overlaps orf19.5621	-3.85726	2.63E-29
orf19.5625	Ortholog of C. dubliniensis CD36 : Cd36_63640, C. auris B8441 : B9J08_001441, Pichia stipitis Pignal :	-3.85914	0.001637

	PICST_32878 and Candida guilliermondii ATCC 6260 : PGUG_03423		
GPM2	Putative phosphoglycerate mutase; repressed in hyphae; macrophage/pseudohyphal-repressed; induced by high levels of peroxide stress, farnesol; flow model biofilm induced; rat catheter and Spider biofilm repressed	-3.88609	3.07E-20
orf19.7473	Ortholog(s) have role in endocytosis and actin cortical patch localization	-3.89773	2E-16
orf19.4963	Ortholog(s) have protein carrier activity, unfolded protein binding activity, role in ribosomal large subunit biogenesis and cytoplasm, nucleus localization	-3.91239	9.74E-26
orf19.2368	Predicted component of U5 snRNP; Spider biofilm induced	-3.96884	4.03E-14
orf19.6316.4	Protein of unknown function; Hap43-induced; rat catheter and Spider biofilm induced	-3.99209	2.76E-06
orf19.3248	Ortholog of C. dubliniensis CD36 : Cd36_26070, C. parapsilosis CDC317 : CPAR2_803220, C. auris B8441 : B9J08_002999 and Candida tenuis NRRL Y-1498 : CANTEDRAFT_131795	-4.00604	6.97E-12
orf19.194	Ortholog of C. dubliniensis CD36 : Cd36_19300, C. parapsilosis CDC317 : CPAR2_209720, C. auris B8441 : B9J08_003188 and Candida tenuis NRRL Y-1498 : CANTEDRAFT_114035	-4.02104	1.05E-29
orf19.915	Protein of unknown function; Spider biofilm induced	-4.02628	5.17E-20
orf19.3625	Has domain(s) with predicted zinc ion binding activity	-4.07349	1.83E-11
orf19.2889	Ortholog(s) have role in ATP-dependent chromatin remodeling, chromatin remodeling, histone exchange and positive regulation of cellular response to phosphate starvation, more	-4.08015	1.39E-10
ALS2	ALS family protein; role in adhesion, biofilm formation, germ tube induction; expressed at infection of human buccal epithelial cells; putative GPI-anchor; induced by ketoconazole, low iron and at cell wall regeneration; regulated by Sfu1p	-4.08793	1.1E-22
orf19.6357	Ortholog(s) have protein-containing complex binding activity	-4.09236	2.22E-25

orf19.2230	Putative pre-mRNA-splicing factor; decreased transcription is observed upon benomyl treatment	-4.10504	1.38E-06
orf19.725	Ortholog of C. dubliniensis CD36 : Cd36_31880, C. parapsilosis CDC317 : CPAR2_702220, C. auris B8441 : B9J08_003984 and Candida tenuis NRRL Y-1498 : CANTEDRAFT_115848	-4.11151	7.85E-13
PEX11	Putative peroxisomal membrane protein; role in fatty acid oxidation; expression is Tac1-regulated; Hms1p-dependent induction by geldamycin; Spider biofilm induced	-4.11901	1.23E-18
orf19.2822	Essential component of the conserved oligomeric Golgi complex; role in fusion of transport vesicles to Golgi compartments; rat catheter biofilm repressed	-4.16552	2.05E-19
orf19.4617	Predicted peptide alpha-N-acetyltransferase; flow model biofilm induced	-4.19605	3.72E-31
orf19.3448	Protein of unknown function; ketoconazole-repressed	-4.23286	2.12E-11
orf19.6579	Ortholog of C. dubliniensis CD36 : Cd36_71340, C. parapsilosis CDC317 : CPAR2_301350, C. auris B8441 : B9J08_004871 and Candida tenuis NRRL Y-1498 : CANTEDRAFT_114098	-4.25681	7.93E-34
orf19.714	Ortholog(s) have GTPase activity	-4.26076	2.31E-14
orf19.2367	Putative protein of unknown function; Hap43-induced; repressed by Rim101; Spider biofilm induced	-4.27117	2.5E-08
orf19.3607	Ortholog of C. dubliniensis CD36 : Cd36_22860, C. parapsilosis CDC317 : CPAR2_806540, C. auris B8441 : B9J08_004792 and Candida tenuis NRRL Y-1498 : CANTEDRAFT_108823	-4.27302	1.03E-07
orf19.2442	Ortholog of S. cerevisiae Shg1; a COMPASS (Set1C) complex subunit that methylates histone H3 on lysine 4 and is required in transcriptional silencing near telomeres; repressed in an azole-resistant strain overexpressing MDR1	-4.28808	5.95E-12
orf19.6983	Protein of unknown function; Hap43-repressed gene; repressed by nitric oxide; Spider biofilm induced	-4.29347	3.14E-38

orf19.5524	Ortholog of C. dubliniensis CD36 : Cd36_62790, C. parapsilosis CDC317 : CPAR2_601680, C. auris B8441 : B9J08_001637 and Candida tenuis NRRL Y-1498 : cten_CGOB_00252	-4.33688	0.010693
orf19.7341.1	Protein of unknown function; Spider biofilm induced	-4.3489	8.96E-10
TOA2	Putative TFIIA small subunit; protein abundance decreased in CAI4 strain compared to the SC5314 strain, abundance not affected by reintegration of URA3 in CAI4; flucytosine induced; possibly an essential gene (UAU1 method)	-4.35444	2.49E-22
orf19.1769	Ortholog(s) have G-protein beta/gamma-subunit complex binding, actin binding activity, role in positive regulation of transcription from RNA polymerase II promoter by pheromones, protein folding and cytoplasm localization	-4.36111	3.35E-34
orf19.1063	Ortholog(s) have alpha-1,6-mannosyltransferase activity, mannosyltransferase activity and role in GPI anchor biosynthetic process	-4.36569	1.41E-26
VMA22	Ortholog(s) have unfolded protein binding activity and role in vacuolar acidification, vacuolar proton-transporting V-type ATPase complex assembly	-4.36696	0.00019
orf19.1776	Putative pantetheine-phosphate adenylyltransferase (PPAT); which catalyzes 4th step in coenzyme A biosynthesis from pantothenate; rat catheter biofilm repressed	-4.39133	4.24E-18
MCP1	Mitochondrial outer membrane protein, component of vacuole and mitochondria patches (vCLAMPs); involved in mitophagy; mutants are defective in mitochondrial function and virulence; regulated by Sef1p, Sfu1p, and Hap43p	-4.39314	7.31E-13
ERG3	C-5 sterol desaturase; introduces C-5(6) double bond into episterol; some clinical isolates show increased azole resistance and defects in hyphal growth and virulence; Efg1p-repressed; fluconazole-induced	-4.42216	3.74E-63
orf19.6605	Has domain(s) with predicted integral component of membrane localization	-4.42228	1.69E-21
orf19.716	Protein of unknown function; Hap43-induced; regulated by Nrg1, Tup1; repressed by alpha pheromone in SpiderM medium; Spider biofilm induced; Bcr1-repressed in RPMI a/a biofilms	-4.43761	1.03E-13

orf19.158	Ortholog of S. cerevisiae Apd1; required for normal localization of actin patches and normal tolerance of sodium ions and hydrogen peroxide; Hap43-induced; Spider biofilm induced	-4.48766	1.26E-48
SMD2	Putative Core Sm protein; Hap43p-induced gene; flucytosine induced	-4.49311	2.04E-07
orf19.2503	Ortholog of S. cerevisiae : YGL138C, C. glabrata CBS138 : CAGL0H08910g, C. dubliniensis CD36 : Cd36_80910, C. parapsilosis CDC317 : CPAR2_102230 and C. auris B8441 : B9J08_002616	-4.56704	0.00458
orf19.4903	Ortholog(s) have N-acetylglucosaminylphosphatidylinositol deacetylase activity	-4.57642	2.75E-38
CAK1	Monomeric CDK-activating kinase; functional homolog of S. cerevisiae Cak1p; phosphorylates cyclin-free human CDK2; lacks glycine loop motif; conserved lysine (K36) not required for activity; possibly essential gene (by UAU1 method)	-4.61842	1.18E-30
COI1	Secreted protein; ciclopirox olamine induced; regulated by Ssn6; induced by nitric oxide independent of Yhb1; Hap43-induced; Spider biofilm repressed	-4.62914	8.27E-47
orf19.4323	Ortholog(s) have phosphatidylinositol-3-phosphate binding activity	-4.69629	9.74E-11
orf19.7199	Ortholog(s) have role in posttranslational protein targeting to endoplasmic reticulum membrane and TRC complex, cytoplasm localization	-4.71504	8.89E-47
CCE1	Putative Holliday junction resolving enzyme; similar to S. cerevisiae Cce1p	-4.72935	2.21E-09
orf19.1049	Predicted NUDIX hydrolase domain; Hap43-induced	-4.76437	5.65E-08
orf19.1092	Dolichol-P-Man dependent alpha(1-3) mannosyltransferase; role in the synthesis of dolichol-linked oligosaccharide donor for N-linked glycosylation of proteins; rat catheter biofilm repressed	-4.8271	6.89E-33
orf19.5235	Putative mitochondrial ribosomal protein of the large subunit; Hap43-induced; mutants are viable; protein level decreases in stationary phase	-4.87611	1.51E-16
orf19.4763	Protein of unknown function; possibly membrane bound; mutants are viable; rat catheter biofilm repressed	-4.91431	2.31E-20

ERG20	Putative farnesyl pyrophosphate synthetase involved in isoprenoid and sterol biosynthesis, based on similarity to S. cerevisiae Erg20p; likely to be essential for growth, based on an insertional mutagenesis strategy	-4.98841	1.02E-79
PMM1	Phosphomannomutase; enzyme of O- and N-linked mannosylation; interconverts mannose-6-phosphate and mannose-l-phosphate; functional homolog of S. cerevisiae Sec53; antigenic in mice; Hap43-induced; flow model and Spider biofilm repressed	-5.05708	4.21E-85
orf19.2228	Ortholog(s) have actin filament binding, actin monomer binding activity	-5.10278	1.67E-38
MED11	Subunit of the RNA polymerase II mediator complex	-5.1422	0.000037
RPN9	Subunit of the 19S regulatory particle lid of the proteasome	-5.24492	7.88E-46
LYS5	Phosphopantetheinyl transferase; enzyme of lysine biosynthesis; modifies, and thereby activates, Lys2p alpha-aminoadipate reductase; functional homolog of S. cerevisiae Lys5p	-5.28411	8.02E-06
PGA10	GPI anchored membrane protein; utilization of hemin and hemoglobin for Fe in host; Rim101 at ph8/hypoxia/ketoconazole/ciclopirox/hypha-induced; required for RPMI biofilm formation, Bcr1-induced in a/a biofilm; rat catheter biofilm repressed	-5.2951	3.62E-36
orf19.1795.1	Ortholog(s) have role in protein targeting to ER, signal peptide processing and signal peptidase complex localization	-5.50158	0.000311
orf19.6272	Putative ubiquitin C-terminal hydrolase; regulated by Gcn2p and Gcn4p	-5.56611	1.61E-42
RPS27A	Ribosomal protein S27; rat catheter and Spider biofilm induced	-5.62312	0.000118
orf19.2778	Protein of unknown function; transcript is upregulated in clinical isolates from HIV+ patients with oral candidiasis	-5.73412	3.75E-11
orf19.1043	Ortholog(s) have lysophosphatidic acid acyltransferase activity, role in cellular triglyceride homeostasis, lipid droplet organization and endoplasmic reticulum, lipid droplet localization	-5.73934	1.04E-10

orf19.927	Ortholog(s) have role in DNA recombination, positive regulation of transcription by RNA polymerase II, regulation of cell aging, telomere maintenance, telomere maintenance via recombination and EKC/KEOPS complex localization	-5.88307	3.44E-08
UCF1	Upregulated by cAMP in filamentous growth; induced in high iron, decreased upon yeast-hypha switch; downregulation correlates with clinical fluconazole resistance; Ras1-regulated; Hap43-repressed; flow model biofilm induced	-5.8849	6.6E-99
SPC19	Essential subunit of the Dam1 (DASH) complex, which acts in chromosome segregation by coupling kinetochores to spindle microtubules	-5.89017	5.34E-09
orf19.4642	Protein of unknown function; Hap43-induced gene	-5.9168	9.51E-44
PEX22	Putative peroxin	-6.00938	2.62E-20
orf19.7330	Protein with a predicted heme oxygenase domain; Spider biofilm induced	-6.40439	2.63E-35
SCP1	Putative cortical actin cytoskeleton protein; constitutive expression independent of MTL or white-opaque status	-6.64914	4.58E-06
orf19.1136	Ortholog(s) have AP-2 adaptor complex, clathrin-coated vesicle localization	-6.70506	1.8E-06
orf19.3687	Ortholog(s) have unfolded protein binding activity, role in cytoskeleton organization, positive regulation of transcription elongation from RNA polymerase II promoter, protein folding and prefoldin complex localization	-6.77534	3.39E-06
orf19.4768	Protein of unknown function; Spider biofilm induced	-6.77886	4.4E-06
orf19.2756	Ortholog(s) have HDEL sequence binding activity, role in endoplasmic reticulum to Golgi vesicle-mediated transport and integral component of endoplasmic reticulum membrane localization	-6.9625	2.25E-44
orf19.6602	Ortholog(s) have role in ubiquinone biosynthetic process, ubiquinone-6 biosynthetic process and mitochondrial inner membrane, mitochondrion localization	-6.99727	5.13E-28
orf19.1691	Plasma-membrane-localized protein; filament induced; Hog1, ketoconazole, fluconazole and hypoxia-induced;	-7.2319	4.58E-91

	regulated by Nrg1, Tup1, Upc2; induced by prostaglandins; flow model biofilm induced; rat catheter and Spider biofilm repressed		
orf19.3446	Ortholog(s) have role in protein import into nucleus and nucleus localization	-7.36862	1.91E-12
RVS162	Protein containing a BAR domain, which is found in proteins involved in membrane curvature; null mutant does not display the endocytic, hyphal growth, virulence, or cell wall defects exhibited by mutants in related genes RVS161 and RVS167	-7.63628	3.73E-08
orf19.4955	Ortholog(s) have role in ascospore wall assembly and ascospore wall, prospore membrane localization (5)	-8.49185	0.021677
orf19.3600	Protein of unknown function; flow model biofilm induced	-9.58246	0.017645
orf19.2847.1	Pseudogene; formerly an ORF Predicted by Annotation Working Group that was subsequently removed from Assembly 20	-9.69116	0.009053
SCW4	Putative cell wall protein; substrate for Kex2p processing in vitro; expression regulated by white-opaque switch; alkaline repressed; possibly essential (UAU1 method); flow model biofilm induced; Spider biofilm induced	-10.1033	5.29E-05
ARG4	Argininosuccinate lyase, catalyzes the final step in the arginine biosynthesis pathway; alkaline downregulated; flow model biofilm induced; Spider biofilm induced	-10.1207	0.000171
orf19.7043	Ortholog of ***S. cerevisiae*** **: YLR050C,** ***C. glabrata CBS138*** **: CAGL0F01991g,** ***C. parapsilosis CDC317*** **: CPAR2_703230,** ***C. auris B8441*** **: B9J08_003284 and** ***Candida tenuis NRRL Y-1498*** **: CANTEDRAFT_117826**	-10.2086	0.010627
RGS2	Protein of RGS superfamily; mutants are viable; rat catheter and Spider biofilm induced	-10.4852	8.82E-05
PRM9	Protein described a similar to S. cerevisiae Prm9; not the ortholog though; mutant is viable	-10.6042	6.65E-06
orf19.5532	Protein of unknown function; Spider biofilm induced	-10.8324	0.008285
orf19.6482	Protein of unknown function; induced by nitric oxide independent of Yhb1p; regulated by Sef1, Sfu1, and Hap43; rat catheter biofilm induced	-10.9513	0.000944

orf19.3783	Protein of unknown function; rat catheter biofilm induced	-11.2208	0.001185
orf19.1707	Ortholog of Candida albicans WO-1 : CAWG_02476	-11.2208	0.006198
orf19.4861.1	Ortholog(s) have tRNA binding activity, role in tRNA export from nucleus and cytoplasm, nuclear pore localization	-11.3219	0.005428
orf19.193	Ortholog of C. dubliniensis CD36 : Cd36_19310, C. parapsilosis CDC317 : CPAR2_209710, C. auris B8441 : B9J08_002949 and Candida tenuis NRRL Y-1498 : CANTEDRAFT_113479	-11.5714	9.51E-06
RAD53	Protein involved in regulation of DNA-damage-induced filamentous growth; putative component of cell cycle checkpoint; ortholog of S. cerevisiae Rad53p, protein kinase required for cell-cycle arrest in response to DNA damage	-11.7814	8.36E-09
orf19.5446	Putative protein of unknown function; transcript is upregulated in clinical isolates from HIV+ patients with oral candidiasis; regulated by Ssn6	-11.8791	5.77E-05
orf19.5042	Ortholog(s) have role in maintenance of rDNA, regulation of exit from mitosis	-12.0258	6.88E-06
orf19.3436	Protein with a Rho GDP-dissociation inhibitor domain; macrophage-induced gene	-12.4801	8.6E-06
orf19.6222.1	Ortholog of ***C. parapsilosis CDC317*** **: CPAR2_208910,** ***Candida tenuis NRRL Y-1498*** **: CANTEDRAFT_114047,** ***Debaryomyces hansenii CBS767*** **: DEHA2D14388g and** ***Pichia stipitis Pignal*** **: PICST_37629**	-12.6625	0.000311
PEX17	Putative peroxin	-12.8803	6.21E-08
orf19.5288.1	Protein of unknown function; Spider biofilm repressed	-13.084	0.000102
DAL4	Putative allantoin permease; fungal-specific (no human or murine homolog)	-13.1287	4.52E-11
IMG2	Mitochondrial ribosomal protein of the large subunit; rat catheter biofilm induced	-13.1325	1.74E-06

orf19.1618.1	Ortholog of C. dubliniensis CD36 : Cd36_82240, C. parapsilosis CDC317 : CPAR2_405130, C. auris B8441 : B9J08_002633 and Candida tenuis NRRL Y-1498 : CANTEDRAFT_127317	-13.1394	3.99E-05
orf19.36	Component of the conserved oligomeric Golgi complex; predicted to mediate fusion of transport vesicles to Golgi compartments; clade-associated gene expression	-13.3224	1.73E-10
MOH1	Ortholog of S. cerevisiae Moh1, essential for staionary phase growth; induced by alpha pheromone in SpiderM medium and by Mnl1 under weak acid stress; possibly essential (UAU1 method); flow model biofilm induced; Spider biofilm induced	-13.4589	2.55E-09
orf19.699	Protein of unknown function; flow model, rat catheter, Spider biofilm induced	-13.8007	7.14E-08
orf19.6307	Ortholog of C. dubliniensis CD36 : Cd36_30250, C. auris B8441 : B9J08_001969, Candida tenuis NRRL Y-1498 : CANTEDRAFT_98619 and Debaryomyces hansenii CBS767 : DEHA2G22594g	-14.1044	1.33E-12
orf19.446.1	Protein with a NADH-ubiquinone oxidoreductase B18 subunit domain; gene has intron	-14.4215	9.41E-08
orf19.7200	Ortholog of C. dubliniensis CD36 : Cd36_73480, Pichia stipitis Pignal : PICST_30727, Candida tropicalis NEW ASSEMBLY : CTRG1_05165 and Candida tropicalis MYA-3404 : CTRG_05165	-14.4983	2.19E-10
orf19.3730	Protein similar to S. cerevisiae Kre27p, which has a role in resistance to killer toxin; predicted Kex2p substrate	-14.6581	7.04E-10
orf19.6308	Ortholog of Candida albicans WO-1 : CAWG_01818	-14.8088	2.64E-12
ARC18	Putative ARP2/3 complex subunit; mutation confers hypersensitivity to cytochalasin D	-14.8813	2.47E-11
LEU2	Isopropyl malate dehydrogenase; leucine biosynthesis; induced by human whole blood or PMNs; protein level decreases in stationary phase; GlcNAc-induced protein; flow model biofilm repressed	-15.6806	1.12E-15
