# Supplementary material for: DNA Damage Checkpoints Govern Global Gene Transcription and Exhibit Species-Specific Regulation on HOF1 in Candida albicans
Source: J Fungi (Basel). 2024 May 29;10(6):387. doi: 10.3390/jof10060387 (PMC11204775; doi:10.3390/jof10060387)
Supplement: Supplementary file 1 [file jof-10-00387-s001.zip › Table S4.pdf]

Gene	Description	log2(fc)	P Value
LDG3	Putative LDG family protein; F-12/CO2 early biofilm induced	13.660664	7.33E-11
orf19.6487	Ortholog of *C. parapsilosis CDC317* : CPAR2_808350, *C. dubliniensis CD36* : Cd36_72060, *Candida metapsilosis* : CMET_5893 and *Candida orthopsilosis Co 90-125* : CORT_0C00820	12.080151	2.1983E-05
orf19.3271	Ortholog of *Candida albicans WO-1* : CAWG_01442	11.978353	6.62483E-06
orf19.6484	Ortholog of *C. parapsilosis CDC317* : CPAR2_808370, *C. dubliniensis CD36* : Cd36_72070, *Candida metapsilosis* : CMET_5895 and *Candida orthopsilosis Co 90-125* : CORT_0C00800	11.554266	3.7007E-28
CSA2	Extracellular heme-binding protein involved in heme-iron acquisition; regulated by Tsa1, Tsa1B in minimal media at 37 deg; induced by ketoconazole, nitric oxide, Hap43; required for normal RPMI biofilm formation	11.344296	0.0014337
BUD20	Protein similar to S. cerevisiae Bud20p, which affects bud site selection; transposon mutation affects filamentous growth	11.053247	0.026739517
orf19.938	Ortholog of *C. dubliniensis CD36* : Cd36_50520, *C. parapsilosis CDC317* : CPAR2_304000, *C. auris B8441* : B9J08_000441 and *Candida tenuis NRRL Y-1498* : cten_CGOB_00233	10.382624	2.34339E-05
orf19.7452	Ortholog(s) have chromatin binding activity and role in DNA replication initiation, DNA unwinding involved in DNA replication, double-strand break repair via break-induced replication	9.0223678	0.0014337
PGA31	Cell wall protein; putative GPI anchor; expression regulated upon white-opaque switch; induced by Congo Red and cell wall regeneration; Bcr1-repressed in RPMI a/a biofilms	8.2394779	4.258E-146
orf19.4653	Protein similar to GPI-linked cell-wall proteins; induced in low iron; Spider biofilm induced; regulated in Spider biofilms by Bcr1, Tec1, Ndt80, Brg1	8.0202321	2.70232E-16
PBR1	Protein of unknown function; required for cohesion, adhesion, and RPMI biofilm formation; induced by alpha pheromone in white cells; fluconazole-induced; Spider biofilm induced	7.0112273	4.55356E-05
orf19.3499	Secreted potein; Hap43-repressed; fluconazole-induced; regulated by Tsa1, Tsa1B under H2O2 stress conditions; induced by Mnl1p under weak acid stress; Spider biofilm induced	6.4552479	1.24273E-98
RBR1	Glycosylphosphatidylinositol (GPI)-anchored cell wall protein; required for filamentous growth at acidic pH; expression repressed by Rim101 and activated by Nrg1; Hap43-induced	5.9404879	7.19858E-14
orf19.2103	Ortholog of *Candida albicans WO-1* : CAWG_03811	5.9118005	0.000534932
FGR46	Protein lacking an ortholog in S. cerevisiae; transposon mutation affects filamentous growth	5.489179	0.007550605
orf19.5549	Ortholog of *C. dubliniensis CD36* : Cd36_63380 and *Candida albicans WO-1* : CAWG_05071	5.1718023	9.93934E-05

PGA23	Putative GPI-anchored protein of unknown function; Rim101-repressed; Cyr1-regulated; colony morphology-related gene regulation by Ssn6	5.064912	2.522E-187
MRV6	Ortholog of *Candida albicans WO-1* : CAWG_04796	5.0370893	0.024974494
orf19.3378	Protein of unknown function; regulated by Tsa1, Tsa1B in minimal media at 37 degrees C	4.6903155	0.013069948
orf19.7077	Putative ferric reductase; induced by Mac1 under copper starvation; Plc1-regulated; Rim101-repressed	4.6022214	1.6479E-74
orf19.5814.1	Protein of unknown function	4.5935485	8.23967E-06
SCW11	Cell wall protein; repressed in ace2 mutant; repressed in core caspofungin response; induced in high iron; possibly an essential gene, disruptants not obtained by UAU1 method; rat catheter and Spider biofilm repressed	4.5780238	1.42964E-51
MCD1	Alpha-kleisin cohesin complex subunit; for sister chromatid cohesion in mitosis and meiosis; repressed by alpha pheromone in SpiderM medium; periodic cell-cycle expression; Hap43-repressed; rat catheter and Spider biofilm repressed	4.4966066	2.39699E-22
MRV8	Membrane protein involved in mycelial growth, biofilm formation and epithelial damage; Spider biofilm induced	4.2662486	1.01584E-28
TBF1	Essential transcription factor; induces ribosomal protein genes and the rDNA locus; acts with Cbf1 at subset of promoters; recruits Fhl1 and Ifh1 to promoters; role is analogous to that of S. cerevisiae Rap1; Spider biofilm induced	4.0765416	3.15737E-18
CRH11	GPI-anchored cell wall transglycosylase, putative ortholog of S. cerevisiae Crh1p; predicted glycosyl hydrolase domain; similar to Csf4p and to antigenic A. fumigatus Aspf9; predicted Kex2p substrate; caspofungin-induced	4.0524475	9.8479E-134
CFL5	Ferric reductase; induced in low iron; ciclopirox olamine, flucytosine induced; amphotericin B, Sfu1 repressed; Tbf1, Hap43 induced	3.959246	2.76421E-05
orf19.2633.1	Ortholog of *C. dubliniensis CD36* : Cd36_00150, *Lodderomyces elongisporus NRLL YB-4239* : LELG_01269 and *Candida tropicalis NEW ASSEMBLY* : CTRG1_CGOB_00075	3.9055645	1.24146E-14
HGT10	Glycerol permease involved in glycerol uptake; member of the major facilitator superfamily; induced by osmotic stress, at low glucose in rich media, during cell wall regeneration; 12 membrane spans; Hap43p-induced gene	3.8766383	3.68044E-59
HBR3	Essential protein; regulated by hemoglobin; S. cerevisiae ortholog is essential; Hap43p-induced gene	3.8227614	5.43882E-24
ART3	CHK1 checkpoint-like protein, partial [Trametes versicolor FP-101664 SS1]	3.8221765	2.1975E-113

orf19.1687	Ortholog of S. cerevisiae Prp43, an RNA helicase in the DEAH-box family that functions in both RNA polymerase I and polymerase II transcript metabolism; Hap43-induced gene	3.8191636	6.57764E-22
orf19.5704	Ortholog(s) have rRNA binding activity, role in RNA splicing, mitochondrial RNA processing, mitochondrial genome maintenance, positive regulation of rRNA processing, rRNA metabolic process and mitochondrion localization	3.8139351	1.5989E-12
orf19.3109	Ortholog of *C. dubliniensis CD36* : Cd36_46470, *C. parapsilosis CDC317* : CPAR2_500900, *Debaryomyces hansenii CBS767* : DEHA2B15730g and *Candida tropicalis MYA-3404* : CTRG_03755	3.7434737	2.58182E-05
orf19.5069	Ortholog of S. cerevisiae Sae3; meiosis specific protein involved in DMC1-dependent meiotic recombination in S. cerevisiae; Spider biofilm induced	3.73064	0.001126384
orf19.6950	Putative vacuolar membrane transporter for cationic amino acids; Spider biofilm induced	3.6981731	0.006760164
CYK3	Essential protein involved in cytokinesis; contains an SH3 domain	3.6841282	0.000902916
PGA38	Putative adhesin-like GPI-anchored protein; repressed during cell wall regeneration; possibly an essential gene, disruptants not obtained by UAU1 method; rat catheter and Spider biofilm repressed	3.6589081	3.73968E-22
orf19.6520	Putative allantoate permease; fungal-specific (no human or murine homolog)	3.6355886	0.006384472
FGR41	Putative GPI-anchored adhesin-like protein; transposon mutation affects filamentous growth; Spider biofilm repressed	3.6260075	1.55909E-73
orf19.5592	Ortholog of *C. dubliniensis CD36* : Cd36_63980, *Candida tenuis NRRL Y-1498* : CANTEDRAFT_115187, *Candida tropicalis NEW ASSEMBLY* : CTRG1_02719 and *Candida tropicalis MYA-3404* : CTRG_02719	3.5949466	6.62847E-05
orf19.5287	Ortholog of *C. dubliniensis CD36* : Cd36_30690, *C. parapsilosis CDC317* : CPAR2_204140, *Debaryomyces hansenii CBS767* : DEHA2F16940g and *Candida tropicalis MYA-3404* : CTRG_00570	3.5825081	9.6107E-28
orf19.5020	Protein of unknown function; Hap43-induced; Spider biofilm induced	3.5694631	3.51663E-17
orf19.270	Ortholog of *C. dubliniensis CD36* : Cd36_82780, *C. parapsilosis CDC317* : CPAR2_102150, *Pichia stipitis Pignal* : psti_CGOB_00155 and *Candida tropicalis MYA-3404* : CTRG_02557	3.5614554	3.62254E-13
DBP7	Putative ATP-dependent DEAD-box RNA helicase; Hap43-induced; rat catheter biofilm induced	3.5556561	1.67573E-12
orf19.6866	Putative U1-70K component of the U1 snRNP, involved in splicing; ortholog of S. cerevisiae SNP1; downregulated upon adherence to polystyrene	3.5455676	3.15247E-13

HHT21	Putative histone H3; amphotericin B repressed; regulated by Efg1, farnesol; Hap43-induced; rat catheter and Spider biofilm repressed	3.5197779	3.4297E-124
NRP1	Ortholog(s) have cytoplasmic stress granule localization	3.4757334	2.73614E-06
CFL4	C-terminus similar to ferric reductases; induced in low iron; Sfu1-repressed; ciclopirox olamine induced; colony morphology-related gene regulation by Ssn6; Hap43-repressed; Sef1-regulated	3.4709062	0.000201543
DRS1	Putative nucleolar DEAD-box protein; Hap43-induced; mutation confers hypersensitivity to 5-fluorouracil (5-FU), tubercidin (7-deazaadenosine); Tbf1-induced; repressed in core stress response	3.4637798	2.29164E-21
orf19.3337	Protein of unknown function; merged with orf19.3338; rat catheter, flow and Spider model biofilm induced; promoter bound by Bcr1, Efg1, Ndt80, and Rob1; orf19.3338 Bcr1-repressed in RPMI a/a biofilms	3.46228	0.00952779
orf19.5353	Ortholog of *C. dubliniensis CD36* : Cd36_24630, *C. parapsilosis CDC317* : CPAR2_407790, *Candida tropicalis MYA-3404* : CTRG_02045, *Candida albicans WO-1* : CAWG_06134 and *Candida metapsilosis* : CMET_2127	3.4396853	0.000810075
orf19.660	Protein of unknown function; mRNA binds She3	3.4389042	1.72872E-05
CZF1	Transcription factor; regulates white-opaque switch; hyphal growth regulator; expression in S. cerevisiae causes dominant-negative inhibition of pheromone response; required for yeast cell adherence to silicone; Spider biofilm induced	3.3970476	1.13885E-06
orf19.1577	Has domain(s) with predicted DNA-binding transcription factor activity, sequence-specific DNA binding, zinc ion binding activity and role in regulation of transcription, DNA-templated	3.3831283	0.009681396
MNN46	Possible mannosyltransferase; Spider biofilm induced	3.3825859	1.4036E-05
INO1	Inositol-1-phosphate synthase; antigenic in human; repressed by farnesol in biofilm or by caspofungin; upstream inositol/choline regulatory element; glycosylation predicted; rat catheter, flow model induced; Spider biofilm repressed	3.3679273	2.80649E-09
GRF10	Putative homeodomain transcription factor, involved in control of filamentous growth; null mutant is an adenine auxotroph; Spider biofilm induced; promoter bound by Bcr1, Tec1, Efg1, Ndt80 and Brg1	3.3451355	5.32366E-11
orf19.5802	Ortholog(s) have transferase activity, role in maturation of SSU-rRNA and cytoplasm localization	3.3423072	1.11972E-06
BRE1	Putative E3 ubiquitin ligase with RING-type zinc finger domain; involved in ubiquitination of histone H2B during hyphal development; transposon mutation affects filamentous growth	3.3324492	1.39856E-33

RHD3	GPI-anchored yeast-associated cell wall protein; induced in high iron; clade-associated gene expression; not essential for cell wall integrity; fluconazole-repressed; flow model and Spider biofilm repressed	3.3301901	1.24619E-66
orf19.2320	Putative serine/threonine-protein kinase; possibly an essential gene, disruptants not obtained by UAU1 method	3.3159497	2.11081E-11
orf19.7422	Ortholog(s) have role in endonucleolytic cleavage in 5'-ETS of tricistronic rRNA transcript (SSU-rRNA, 5.8S rRNA and LSU-rRNA)	3.3132676	3.70727E-29
orf19.4101	Ortholog of *C. parapsilosis CDC317* : CPAR2_104840, *C. auris B8441* : B9J08_001223, *Candida tenuis NRRL Y-1498* : CANTEDRAFT_116070 and *Debaryomyces hansenii CBS767* : DEHA2F04994g	3.2833892	7.34212E-06
BDF1	Essential chromatin-binding bromodomain protein; repressed upon adherence to polystyrene; reduced mRNA abundance detected in null mutant; macrophage/pseudohyphal-repressed	3.2647454	4.98138E-25
CGT1	mRNA 5' guanylyltransferase; small subunit of mRNA capping enzyme; binds an RNA Pol II C-terminal domain peptide; Cet1p and Cgt1p form a 2:1 complex; functional homolog of S. cerevisiae Ceg1p	3.2573293	7.51098E-14
BMT7	Beta-mannosyltransferase, member of a 9-gene family that includes characterized genes BMT1, BMT2, BMT3, and BMT4 with roles in beta-1,2-mannosylation of cell wall phosphopeptidomannan; downregulated in azole-resistant strain; Hap43p-induced	3.250845	4.45478E-10
CHT3	Major chitinase; secreted; functional homolog of S. cerevisiae Cts1p; 4 N-glycosylation motifs; possible O-mannosylation; putative signal peptide; hyphal-repressed; farnesol upregulated in biofilm; regulated by Efg1p, Cyr1p, Ras1p	3.2495174	2.68119E-40
SRP40	Putative chaperone of small nucleolar ribonucleoprotein particles; macrophage/pseudohyphal-induced; rat catheter biofilm induced	3.2388914	1.46997E-20
ECM331	GPI-anchored protein; mainly at plasma membrane, also at cell wall; Hap43, caspofungin-induced; Plc1-regulated; Hog1, Rim101-repressed; colony morphology-related regulated by Ssn6; induced by ketoconazole and hypoxia	3.2363399	1.23416E-42
PGA13	GPI-anchored cell wall protein involved in cell wall synthesis; required for normal cell surface properties; induced in oralpharyngeal candidasis; Spider biofilm induced; Bcr1-repressed in RPMI a/a biofilms	3.208678	4.89159E-61
CAG1	Heterotrimeric G protein alpha subunit; positive role in mating pheromone response; opaque-enriched transcript; transcript	3.1971465	0.000296462

	repressed by MTLa1-MTLalpha2; regulated by hemoglobin-responsive Hbr1 via MTL genes; rat catheter biofilm repressed		
HTA2	Putative histone H2A; farnesol regulated; rat catheter biofilm repressed; Spider biofilm repressed; Hap43-induced	3.1541961	4.12533E-98
LTV1	Putative GSE complex component; repressed by prostaglandins	3.1531851	1.68806E-25
orf19.4245	Protein with a predicted pleckstrin domain; Hap43-repressed gene	3.1413856	8.98874E-12
NOP4	Putative nucleolar protein; Hap43-induced; mutation confers hypersensitivity to 5-fluorocytosine (5-FC), 5-fluorouracil (5-FU), and tubercidin (7-deazaadenosine); represses in core stress response	3.1325668	1.46644E-15
RNR3	Putative ribonucleotide reductase large subunit; transcript induced in low iron; rat catheter and Spider biofilm induced	3.1255309	0.025761836
HHF1	Putative histone H4; repressed in fkh2 mutant; regulated by Efg1; fluconazole induced; amphotericin B repressed; farnesol regulated; colony morphology-related gene regulation by Ssn6; Hap43-induced; rat catheter and Spider biofilm repressed	3.1223765	9.91852E-86
NOG1	Putative GTPase; mutation confers hypersensitivity to 5-fluorocytosine (5-FC), 5-fluorouracil (5-FU), and tubercidin (7-deazaadenosine); repressed by prostaglandins; Hap43-induced	3.1193744	8.89315E-15
orf19.6675	Protein of unknown function; from Assembly 19; removed from Assembly 20; restored based on comparative genome analysis; F-12/CO2 early biofilm induced	3.1079648	4.75844E-13
orf19.1434	Ortholog(s) have DNA polymerase binding, protein kinase activator activity, signaling adaptor activity	3.0994247	1.47528E-24
FGR6-1	Protein lacking an ortholog in S. cerevisiae; member of a family encoded by FGR6-related genes in the RB2 repeat sequence; transposon mutation affects filamentous growth	3.0968615	0.003457504
orf19.1959	Ortholog of *S. cerevisiae* : OTU2, *C. dubliniensis CD36* : Cd36_51210, *C. parapsilosis CDC317* : CPAR2_303850, *C. auris B8441* : B9J08_000423 and *Candida tenuis NRRL Y-1498* : CANTEDRAFT_114159	3.0962711	9.58843E-15
orf19.6227	Ortholog of *C. dubliniensis CD36* : Cd36_06390, *C. parapsilosis CDC317* : CPAR2_209040, *C. auris B8441* : B9J08_003388 and *Candida tenuis NRRL Y-1498* : CANTEDRAFT_114052	3.0907848	4.93552E-12
UTP18	Putative U3 snoRNA-associated protein; Hap43-induced; repressed in core stress response; physically interacts with TAP-tagged Nop1	3.0690141	3.2128E-24
SPB1	Putative AdoMet-dependent methyltransferase; Hap43-induced; repressed by prostaglandins; possibly essential gene, disruptants not obtained by UAU1 method; Spider biofilm induced	3.0688192	2.9733E-10

orf19.6090	Putative nucleolar protein with a predicted role in pre-rRNA processing and ribosome biogenesis; repressed by nitric oxide; required for flow model biofilm formation; Spider biofilm repressed	3.0681965	1.53999E-41
orf19.4046	Putative transcription factor containing a Zn(2)-Cys(6) binuclear cluster	3.0653985	4.325E-88
orf19.7455	Ortholog of *C. dubliniensis CD36* : Cd36_86630, *Candida tropicalis NEW ASSEMBLY* : CTRG1_05698 and *Candida tropicalis MYA-3404* : CTRG_05698	3.0541889	2.51435E-07
ASK1	Essential subunit of the Dam1 (DASH) complex, which acts in chromosome segregation by coupling kinetochores to spindle microtubules	3.0513469	4.1967E-08
orf19.2319	Putative nucleolar protein with a predicted role in pre-rRNA processing; Hap43-induced gene; repressed in core stress response	3.0306154	2.4772E-13
orf19.2657	Protein of unknown function	3.0281969	7.59648E-06
NOC2	Putative nucleolar complex protein; Hap43-induced; transposon mutation affects filamentous growth; mutation confers hypersensitivity to 5-fluorouracil (5-FU), tubercidin (7-deazaadenosine); repressed in core stress response	3.0238581	2.39944E-09
orf19.1642	Ortholog of S. cerevisiae Loc1, a nuclear protein involved in asymmetric localization of ASH1 mRNA in S. cerevisiae; Hap43-induced gene; Spider biofilm induced	3.0179386	1.57351E-37
orf19.494	Putative RNA-binding protein; role in assembly of box H/ACA snoRNPs and thus pre-rRNA processing; Spider biofilm induced	3.0083635	2.51086E-12
PGA6	GPI-anchored cell wall adhesin-like protein; induced by high iron; upregulated upon Als2 depletion; mRNA binds She3 and is localized to hyphal tips; Spider biofilm repressed	3.0063258	1.96523E-65
HCM1	Protein with forkhead domain; similar to S. cerevisiae Hcm1p; Hap43p-induced gene	2.9843541	1.54859E-17
AFT2	Putative Aft domain transcription factor; role in regulation of iron metabolism, oxidative stress, adhesion, hyphal growth, colony morphology, virulence; complements S. cerevisiae aft1 mutation; Spider biofilm induced	2.9808912	1.87402E-05
MDJ1	Putative member of the HSP40 (DnaJ) family of chaperones; rat catheter and Spider biofilm induced	2.9778613	4.40424E-18
RBF1	Transcription factor; glutamine-rich activation domain; binds RPG-box DNA sequences; predominantly nuclear; mutation causes accelerated induction of filamentous growth; antigenic during human oral infection; Sko1p-repressed	2.9750358	1.27713E-17
orf19.355	Has domain(s) with predicted oxidoreductase activity	2.9748243	0.00135792
FET31	Putative multicopper oxidase; ketoconazole/caspofungin/amphotericin B repressed;	2.9703357	2.8246E-42

	Sef1/Sfu1/Hap43 regulated; reports differ if functional homolog of ScFet3; rat catheter and Spider biofilm induced		
orf19.5991	Ortholog(s) have role in assembly of large subunit precursor of preribosome, maturation of 5.8S rRNA from tricistronic rRNA transcript	2.9494511	3.08515E-15
orf19.5775	Predicted ORF overlapping the Major Repeat Sequence on chromosome 6; member of a family encoded by FGR6-related genes in the RB2 repeat sequence; rat catheter biofilm repressed	2.9489353	8.89892E-08
PRP22	Putative RNA-dependent ATPase; induced upon adherence to polystyrene; induced by Mnl1p under weak acid stress	2.9481421	2.67462E-32
HHT2	Putative histone H3; farnesol regulated; Hap43-induced; rat catheter and Spider biofilm repressed	2.9476022	4.6667E-96
orf19.3100	Protein with t-SNARE domains and a microtubule associated domain; Hap43-induced gene; repressed by alpha pheromone in SpiderM medium	2.9397272	4.42213E-08
orf19.3894	Putative inositol oxygenase; mutation confers hypersensitivity to toxic ergosterol analog; Spider biofilm induced	2.9314129	4.22554E-18
orf19.6366	Has domain(s) with predicted RNA binding activity	2.9179834	1.32511E-25
orf19.4160	Ortholog(s) have N(6)-L-threonylcarbamoyladenine synthase activity and role in mitochondrial tRNA threonylcarbamoyladenosine modification, tRNA threonylcarbamoyladenosine modification	2.9167826	7.74213E-17
orf19.6859	Ortholog of *C. dubliniensis CD36* : Cd36_44860, *Candida tropicalis NEW ASSEMBLY* : CTRG1_04054, *Candida tropicalis MYA-3404* : CTRG_04054 and *Candida albicans WO-1* : CAWG_03295	2.8977637	0.000325702
orf19.5430	Small-subunit processome component; repressed by prostaglandins	2.890927	4.91034E-16
MAK21	Putative 66S pre-ribosomal particle subunit; mutation confers hypersensitivity to tubercidin (7-deazaadenosine)	2.8868162	4.68779E-13
orf19.5952	Protein of unknown function; induced by nitric oxide independent of Yhb1; Sef1, Sfu1, and Hap43-induced; rat catheter and Spider biofilm induced	2.8859663	1.38374E-17
orf19.2386	Ortholog(s) have role in endonucleolytic cleavage in 5'-ETS of tricistronic rRNA transcript	2.8842866	8.12775E-19
UTP21	Putative U3 snoRNP protein; Hap43-induce; physically interacts with TAP-tagged Nop1; Spider biofilm induced	2.8831677	3.59923E-09
RPA135	Putative RNA polymerase I subunit A135; repressed by prostaglandins	2.8826893	3.05888E-18
orf19.1473	2-hydroxyacid dehydrogenase domain-containing protein; Hap43-repressed gene; induced by alpha pheromone in SpiderM medium	2.8782699	2.47874E-05

orf19.1562	Protein of unknown function; flow model biofilm induced; Spider biofilm induced; repressed by alpha pheromone in SpiderM medium	2.8748087	1.52387E-08
NOG2	Putative nucleolar GTPase; repressed by prostaglandins; Hap43-induced, rat catheter and Spider biofilm induced	2.8450485	7.00625E-37
orf19.2612	C2H2 zinc finger transcription factor; expression reduced in ssr1 null mutant; flow model biofilm induced	2.8430229	1.20049E-09
CRZ1	Calcineurin-regulated C2H2 transcription factor; role in maintenance of membrane integrity, azole tolerance; not required for mouse virulence; repressed by low iron; regulates Ca++ influx during alkaline pH response; Spider biofilm induced	2.811717	1.31962E-12
RER2	Putative cis-prenyltransferase involved in dolichol synthesis; participates in endoplasmic reticulum (ER) protein sorting; flow model biofilm induced	2.8098789	1.02259E-20
orf19.1948	Ortholog of *C. dubliniensis CD36* : Cd36_51110, *C. parapsilosis CDC317* : CPAR2_302560, *C. auris B8441* : B9J08_005315 and *Candida tenuis NRRL Y-1498* : CANTEDRAFT_135271	2.7977286	2.56016E-13
MCM3	Putative DNA replication protein; periodic mRNA expression, peak at cell-cycle M/G1 phase; Spider biofilm induced	2.7927465	1.50277E-21
orf19.6234	Putative U2 snRNP component; mutation confers hypersensitivity to 5-fluorocytosine (5-FC), 5-fluorouracil (5-FU), and tubercidin (7-deazaadenosine); Hap43-induced, Spider biofilm induced	2.785445	1.08729E-08
orf19.6853	Protein of unknown function; Spider biofilm repressed	2.775149	2.96901E-15
HSX11	UDP-glucose:ceramide glucosyltransferase (glucosylceramide synthase [GCS], EC 2.4.1.80); involved in glucosylceramide biosynthesis, which is important for virulence	2.7698378	5.60578E-06
GLE1	Putative nucleoporin; moderately induced at 42 degrees C	2.7624383	3.75765E-11
orf19.501	Ortholog(s) have rRNA (cytosine-C5-)-methyltransferase activity	2.7595602	3.54024E-18
orf19.4835	Ortholog(s) have role in endonucleolytic cleavage in 5'-ETS of tricistronic rRNA transcript	2.7541417	3.91636E-10
SSK1	Response regulator of two-component system; role in oxidative stress response, cell wall biosynthesis, virulence, hyphal growth on solid media; expressed in hyphae and yeast; peroxisomal targeting sequence (PTS1); Spider biofilm induced	2.753449	9.16381E-60
orf19.3932.1	Has domain(s) with predicted nucleic acid binding, nucleotide binding activity	2.7517232	0.013680072
FAR1	Protein involved in regulation of pheromone-mediated mating; repressed by A1p and Alpha2p in white-phase cells; null mutant shows no pheromone response in opaque cells; overexpression causes enhanced pheromone response and cell cycle arrest	2.7480816	5.22133E-16

RPO41	Putative mitochondrial RNA polymerase; repressed in core stress response; Spider biofilm induced	2.7464781	4.25261E-13
DAG7	Secretory protein; a-specific, alpha-factor induced; mutation confers hypersensitivity to toxic ergosterol analog; fluconazole-induced; induced during chlamydospore formation in C. albicans and C. dubliniensis	2.7418852	1.22055E-30
FET99	Multicopper oxidase family protein; similar to S. cerevisiae Fet3; does not complement S. cerevisiae fet3 mutant growth under low-iron; iron-repressed; regulated by Tup1, Rim101; flow model biofilm induced; Spider biofilm repressed	2.7237266	3.21595E-07
HTA3	Putative histone H2A; amphotericin B repressed; flucytosine induced; RNA abundance regulated by tyrosol and cell density; Spider biofilm repressed	2.7208223	3.79473E-60
orf19.2527	Putative protein of unknown function; Hap43-induced; required for normal biofilm growth; F-12/CO2 early biofilm induced	2.7196503	5.71604E-13
CCW14	Putative mannoprotein of cell wall with role in response to stress; increased mRNA abundance observed in cyr1 homozygous mutant (hyphal or yeast-form cells) and in ras1 homozygous mutant (yeast-form cells)	2.71863	3.86369E-07
orf19.267	Protein required for normal filamentous growth; mRNA binds She3	2.7177177	6.63882E-08
NOT4	Putative E3 ubiquitin-protein ligase; required for maintenance, but not induction, of hyphal development; homozygous null mutant is avirulent in mouse systemic infection despite persistence in host; repressed in rat oral candidiasis	2.712312	5.05344E-17
DIP2	Putative small ribonucleoprotein complex; Tn mutation affects filamentous growth; physically interacts with TAP-tagged Nop1; heterozygous null mutant exhibits resistance to parnafungin; Hap43-induced gene; Spider biofilm induced	2.7050563	1.77522E-09
UTP9	Small-subunit processome protein; Ssr1-induced; repressed by prostaglandins; physically interacts with TAP-tagged Nop1	2.7020982	3.67671E-30
orf19.5267	Putative cell wall adhesin-like protein; repressed in core caspofungin response and by alpha pheromone in SpiderM medium; transcript reduced in ace2 mutant; flow model, rat catheter and Spider biofilm repressed	2.6939292	9.38341E-07
NIP7	Putative nucleolar protein with role in ribosomal assembly; hyphal-induced; Hap43-induced; Spider biofilm induced	2.6931839	0.010244256
orf19.389	Protein of unknown function; Hap43-induced gene	2.6882638	6.82366E-07
orf19.6175	Putative 35S rRNA processing protein; Hap43-induced; repressed by prostaglandins; Spider biofilm induced	2.6793592	6.85957E-17
PEL1	Predicted enzyme of mitochondrial phospholipid biosynthesis; rat catheter and flow model biofilm induced	2.6788755	1.54329E-11

orf19.1825	Protein of unknown function; mutants are viable; filament induced; regulated by Nrg1, Rfg1, Tup1	2.6774087	6.53337E-05
orf19.7574	Ortholog(s) have ubiquitin ligase complex localization	2.6717463	3.02593E-08
NSA1	Putative 66S pre-ribosomal particles conmponent; Hap43-induced; repressed by prostaglandins	2.6695044	2.26162E-16
HSL1	Probable protein kinase involved in determination of morphology during the cell cycle of both yeast-form and hyphal cells via regulation of Swe1p and Cdc28p; required for full virulence and kidney colonization in mouse systemic infection	2.6693118	8.44119E-07
EAF7	Subunit of the NuA4 histone acetyltransferase complex	2.6681698	2.47933E-08
EXO1	Putative exodeoxyribonuclease; cell-cycle regulated periodic mRNA expression	2.6667203	9.64844E-14
RPC53	Ortholog(s) have RNA polymerase III activity, role in tRNA transcription by RNA polymerase III and RNA polymerase III complex localization	2.6665742	4.70595E-11
MRPL8	Mitochondrial 60S ribosomal protein subunit; Hap43p-repressed gene	2.6572982	2.16658E-31
EXG2	GPI-anchored cell wall protein, similar to S. cerevisiae exo-1,3-beta-glucosidase Exg2p; predicted Kex2p substrate; induced during cell wall regeneration; possibly an essential gene, disruptants not obtained by UAU1 method; Hap43p-repressed	2.6497328	4.11048E-08
orf19.7450	Ortholog(s) have myosin II tail binding, protein-macromolecule adaptor activity, role in protein localization to cell division site, septin ring assembly and cellular bud neck, cellular bud neck septin ring localization	2.642203	7.47274E-17
orf19.7397.1	Predicted ORF identical to NSA2	2.6270489	1.15674E-23
NSA2	Putative protein constituent of 66S pre-ribosomal particles; Hap43-induced; repressed by prostaglandins	2.6270489	1.15674E-23
orf19.4029	Ortholog(s) have unfolded protein binding activity, role in ribosomal large subunit assembly, ribosomal large subunit biogenesis and cytosol localization	2.6269913	2.78494E-21
PGA58	Putative GPI-anchored protein; transcription is positively regulated by Tbf1p	2.6261434	3.27639E-14
orf19.4030	Ortholog(s) have DNA primase activity, single-stranded DNA binding activity and role in DNA replication, DNA replication, synthesis of RNA primer	2.6247788	2.93908E-21
orf19.6723	Protein of unknown function; Spider biofilm induced	2.6240623	3.64221E-07
orf19.4011	Similar to S. pombe mug180, a predicted esterase/lipase; highly induced during chlamydospore formation in both C. albicans and C. dubliniensis; flow model biofilm induced	2.6232325	0.01457896
orf19.3431	Ortholog(s) have 3'-5' exonuclease activity, DNA-directed DNA polymerase activity, role in mitochondrial DNA catabolic	2.6147098	0.024631518

	process, mitochondrial DNA replication, mitochondrial genome maintenance and mitochondrion localization		
RFX1	Transcription factor; involved in DNA damage responses, morphogenesis, and virulence; Spider biofilm induced	2.6078993	0.002141149
orf19.4563	Protein of unknown function; repressed by prostaglandins; Hap43-induced, Spider biofilm induced	2.5861819	1.24311E-15
orf19.344	Protein of unknown function; upregulated by fluphenazine treatment or in an azole-resistant strain that overexpresses CDR1 and CDR2; transcript possibly regulated by Tac1	2.5849625	1.07837E-07
UTP8	Essential nucleolar protein; involved in tRNA export from the nucleus and ribosomal small subunit biogenesis; physically interacts with TAP-tagged Nop1; Spider biofilm induced	2.5837598	7.27363E-28
orf19.2639	Ortholog(s) have structural constituent of ribosome activity and mitochondrial large ribosomal subunit localization	2.5754745	2.08461E-42
POP4	Ortholog of S. cerevisiae Pop4; a subunit of both RNase MRP and nuclear RNase P; filament induced; regulated by Nrg1, Tup1; likely essential, based on UAU1 strategy; rat catheter and Spider biofilm induced	2.5737554	4.03059E-06
RBT4	Pry family protein; required for virulence in mouse systemic/rabbit corneal infections; not filamentation; mRNA binds She3, is localized to hyphal tips; Hap43-induced; in both yeast and hyphal culture supernatants; Spider biofilm induced	2.5723096	2.95375E-50
MSS116	Putative DEAD-box protein; required for efficient splicing of mitochondrial Group I and II introns; Hap43-induced; rat catheter biofilm induced	2.5615273	1.63957E-14
orf19.6326	Ortholog of *C. dubliniensis CD36* : Cd36_60130, *C. parapsilosis CDC317* : CPAR2_603380, *C. auris B8441* : B9J08_002731 and *Candida tenuis NRRL Y-1498* : CANTEDRAFT_94402	2.561136	1.58643E-28
MEP2	Ammonium permease and regulator of nitrogen starvation-induced filamentation; 11 predicted transmembrane regions; in low nitrogen cytoplasmic C-terminus activates Ras/cAMP and MAPK signal transduction pathways to induce filamentation	2.5576084	2.52174E-48
orf19.429	Putative non-canonical poly(A) polymerase; repressed by nitric oxide; Spider biofilm induced	2.5567489	3.07186E-06
ARX1	Putative ribosomal large subunit biogenesis protein; repressed in core stress response; repressed by prostaglandins	2.5501421	2.60395E-19
MPP10	Putative SSU processome and 90S preribosome component; repressed in core stress response; repressed by prostaglandins	2.5485296	1.79477E-17
RSC8	Component of the RSC chromatin remodeling complex; possibly an essential gene, disruptants not obtained by UAU1 method	2.5435226	9.69979E-15
MAK5	Putative nucleolar DEAD-box RNA helicase; oxidative stress-repressed via Cap1; repressed by prostaglandins	2.5389573	2.90025E-05

orf19.6639	Ortholog of S. cerevisiae Mdm36; mitochondrial distribution and morphology protein; Hap43-repressed gene	2.5362379	8.20146E-12
CRK1	Protein kinase of the Cdc2 subfamily involved in hyphal development, virulence; promotes hyphal development independently of Cph1 and Efg1; functionally complements pheromone hypersensitivity of S. cerevisiae sgv1 mutant; Hap43p-repressed	2.5319305	3.24286E-07
orf19.4068	Protein of unknown function; flow model biofilm induced	2.5251694	0.000552535
CCT3	Putative cytosolic chaperonin Cct ring complex subunit; mutation confers hypersensitivity to cytochalasin D	2.5142775	1.22893E-23
NCS2	Putative cytosolic thiouridylase subunit; Spider biofilm induced	2.5087699	1.23436E-07
orf19.7552	Putative U3-containing small subunit processome complex protein; Hap43-induced gene; repressed in core stress response; Spider biofilm induced	2.5085318	1.31894E-07
orf19.279	Protein with a predicted D-Tyr-tRNA(Tyr) deacylase domain; Hap43-repressed gene	2.5083966	0.004368037
DBF4	Regulatory subunit of Cdc7p-Dbf4p protein kinase complex that acts as negative regulator of hyphal development; cell-cycle regulated periodic mRNA expression; S. cerevisiae ortholog is not cell-cycle regulated	2.5067624	1.14396E-24
orf19.4301	Ortholog(s) have role in chromatin silencing at telomere, rDNA heterochromatin assembly, regulation of transcription by RNA polymerase II, silent mating-type cassette heterochromatin assembly	2.502195	1.85869E-08
PHO4	bHLH transcription factor of the myc-family; required for phosphate acquisition and for resistance to stresses; induced by Mnl1 under weak acid stress	2.5011309	2.073E-09
orf19.5225	Ortholog of *Candida albicans WO-1* : CAWG_00201	2.4964258	0.000488464
YOX1	Putative homeodomain-containing transcription factor; transcriptional repressor; periodic mRNA expression, peak at cell-cycle G1/S phase	2.4959438	1.05858E-22
orf19.4330	Ortholog of *C. dubliniensis CD36* : Cd36_52790, *Candida tropicalis NEW ASSEMBLY* : CTRG1_05968, *Candida tropicalis MYA-3404* : CTRG_05968 and *Candida albicans WO-1* : CAWG_04684	2.4929842	0.005798978
RSC4	Component of the RSC chromatin remodeling complex	2.4922805	7.43735E-15
orf19.3295	Has domain(s) with predicted role in cell division, chromosome segregation and MIS12/MIND type complex localization	2.4905837	9.74531E-06
orf19.6252	Ortholog(s) have RNA polymerase II complex binding activity	2.4890527	1.68679E-14
orf19.7104	Adhesin-like cell wall protein; similar to mucins and to a Litomosoides microfilarial sheath protein; N-terminal secretion signal; rat catheter and Spider biofilm repressed	2.4885676	1.33011E-30

CHS1	Chitin synthase; essential; for primary septum synthesis in yeast and hyphae; 1 of several chitin synthases; enzymatically activated by proteolytic processing; complements defects of S. cerevisiae chs1 or chs2; Spider biofilm repressed	2.487819	1.38564E-12
PES1	Pescadillo homolog required for dispersal of biofilm cells into planktonic yeast cells; essential in yeast cells, not in hyphal cells; mutation confers hypersensitivity to 5-fluorocytosine, 5-fluorouracil, tubercidin	2.487359	4.5227E-12
NOP58	Ortholog of S. cerevisiae Nop58; involved in pre-rRNA process; Tn mutation affects filamentous growth; macrophage/pseudohyphal-induced; physically interacts with TAP-tagged Nop1; Spider biofilm repressed	2.4840448	2.26938E-30
CDC47	Phosphorylated protein described as having role in control of cell division; RNA abundance regulated by tyrosol and cell density; merged with orf19.201 in Assembly 20; unmerged from orf19.201 in a revision of Assembly 21	2.4836983	2.97806E-14
orf19.6742	Ortholog(s) have RNA polymerase II CTD heptapeptide repeat phosphatase activity, protein serine/threonine phosphatase activity	2.4809648	0.000335219
CNS1	Putative co-chaperone; Hap43p-induced gene; mutation confers hypersensitivity to radicicol	2.4733171	4.99674E-14
MSM1	Mitochondrial methionyl-tRNA synthetase (MetRS); functionally complements methionine auxotrophy of an E. coli MetRS mutant; transcript regulated by Nrg1; flow model biofilm induced	2.4664321	5.31517E-07
orf19.2917	Putative GTPase; heterozygous null mutant exhibits resistance to parnafungin in the C. albicans fitness test; Hap43p-induced gene	2.4663734	2.3153E-19
CSE4	Centromeric histone H3 variant; role in structural changes of centromeric nucleosomes during cell cycle; 4 nucleosomes bind 1 centromere, each has 1 Cse4 molecule during most of cell cycle, 2 molecules in anaphase; Spider biofilm repressed	2.4591693	4.59227E-10
orf19.2481	Protein of unknown function; Spider biofilm induced	2.4523883	0.024659714
SDA1	Predicted nuclear protein involved in actin cytoskeleton organization, passage through Start, 60S ribosome biogenesis; rat catheter biofilm induced; Hap43-induced	2.4493484	2.34024E-05
FHL1	Fork-head transcription factor; forms a heterodimer with Ifh1 that is tethered to promoters by Tbf1; positively regulates rRNA and ribosomal protein gene transcription	2.4404456	8.91957E-08
orf19.6418	Ortholog(s) have unfolded protein binding activity and role in protein import into nucleus, ribosomal large subunit biogenesis	2.4402415	7.25591E-11
PRR2	Putative serine/threonine protein kinase; mutation confers resistance to 5-fluorocytosine (5-FC)	2.4381575	0.000406905

orf19.3275	Ortholog of *C. dubliniensis CD36* : Cd36_25870, *C. parapsilosis CDC317* : CPAR2_804000, *C. auris B8441* : B9J08_001023 and *Candida tenuis NRRL Y-1498* : CANTEDRAFT_115661	2.4344051	1.28768E-05
TAF12	Essential component of transcription factor TFIID complex, involved in transcription regulation	2.4334388	2.1281E-11
HCA4	Putative role in regulation of cell wall biogenesis; Hap43p-induced gene; possibly an essential gene, disruptants not obtained by UAU1 method; flow model and rat catheter biofilm induced	2.4313958	6.49239E-06
ARH2	Putative adrenodoxin-NADPH oxidoreductase; role in heme biosynthesis	2.4261904	0.000168177
orf19.6712	Ortholog of *S. cerevisiae* : SGM1, *C. glabrata CBS138* : CAGL0M00462g, *C. dubliniensis CD36* : Cd36_87610, *C. parapsilosis CDC317* : CPAR2_808090 and *C. auris B8441* : B9J08_004134	2.4229057	2.79938E-09
MET4	Putative transcription coactivator; predicted role in sulfur amino acid metabolism; required for yeast cell adherence to silicone substrate; Spider biofilm induced	2.417514	3.68287E-07
MTG1	Putative mitochondrial GTPase; likely essential for respiratory competence and in large ribosomal subunit assembly; mitochondrial translation; Spider biofilm induced	2.4112993	2.24995E-09
orf19.3563	Ortholog of *C. dubliniensis CD36* : Cd36_19810, *C. parapsilosis CDC317* : CPAR2_206450, *C. auris B8441* : B9J08_005014 and *Candida tenuis NRRL Y-1498* : CANTEDRAFT_113905	2.4099284	0.000479155
SGD1	Predicted small ribosomal subunit biogenesis protein; repressed in core stress response; transcript increases in populations of cells exposed to fluconazole over multiple generations; Spider biofilm induced	2.4074243	3.53226E-05
MSH6	Protein similar to S. cerevisiae Msh6p, which is involved in mismatch repair; repressed under Cdc5p depletion; Hap43p-induced gene	2.4020184	1.95625E-14
PAM16	Putative maltase; regulated by Gcn4; repressed by amino acid starvation (3-AT); rat catheter biofilm induced	2.401489	1.12837E-37
SET1	Lysine histone methyltransferase; methylates histone H3 K4; regulates of white-opaque switch, epithelial cell adhesion, agar-embedded filamentation, virulence in mice; unique N-terminus immunogenic in human; rat catheter biofilm repressed	2.4012505	0.000475253
orf19.2604	S. pombe ortholog SPAC2C4.06c is a predicted tRNA (cytosine-5-)-methyltransferase; Spider biofilm induced	2.3975477	0.002114605
orf19.7624	Ortholog(s) have role in maturation of SSU-rRNA from tricistronic rRNA transcript (SSU-rRNA, 5.8S rRNA, LSU-rRNA), rRNA processing and nucleolus, small-subunit processome localization	2.3965439	2.00526E-08

orf19.5929	Putative protein of unknown function, transcription is positively regulated by Tbf1p	2.3955571	6.49812E-07
MBP1	Putative component of the MBF transcription complex involved in G1/S cell-cycle progression; non-periodic mRNA expression; predicted, conserved MBF binding sites upstream of G1/S-regulated genes	2.391331	0.000412765
orf19.6940	Protein of unknown function	2.3794375	0.005694277
PGA48	Putative GPI-anchored adhesin-like protein; similar to S. cerevisiae Spi1p, which is induced at stationary phase; transcript induced in high iron; flow model biofilm induced; Spider biofilm repressed	2.3782082	9.06916E-33
CHS5	Putative chitin biosynthesis protein; fungal-specific; repressed upon yeast-to-hypha switch; rat catheter biofilm repressed	2.3774896	9.62249E-15
RPL29	Ribosomal protein L29; induced upon germ tube formation; colony morphology-related gene regulation by Ssn6; intron in 5'-UTR; Spider biofilm repressed	2.3746902	1.04102E-36
HHF22	Putative histone H4; regulated by Efg1; flucytosine, fluconazole-induced; amphotericin B, caspofungin repressed; colony morphology-related gene regulation by Ssn6; Hap43-induced; rat catheter and Spider biofilm repressed	2.3682952	4.03288E-45
orf19.512	Ortholog of S. cerevisiae Kre33; essential; S. cerevisiae ortholog is essential and is required for biogenesis of the small ribosomal subunit	2.366905	5.94334E-08
APM1	Ortholog of S. cerevisiae/S. pombe Apm1; a clathrin-associated protein complex (AP-1) subunit; phosphorylated protein; Tn mutation affects filamentous growth; Spider biofilm repressed	2.355143	5.17574E-33
RAT1	5'-->3' exoribonuclease; similar to S. cerevisiae nuclear exoribonuclease Rat1p; suppresses S. cerevisiae kem1 mutant slow growth, mating defect, and haploid invasive growth defect	2.3517874	1.93245E-06
orf19.1426	Ortholog of S. cerevisiae Skg6; localizes to the cell cortex, cell bud neck, cell bud tip, incipient cellular bud site, and is membrane-bound; Spider biofilm induced	2.3461321	1.33612E-06
CDC11	Septin; cell and hyphal morphology, agar-invasive growth, full virulence and kidney tissue invasion in mouse, but not kidney colonization, immunogenicity; hyphal and cell-cycle-regulated phosphorylation; rat catheter biofilm repressed	2.345997	1.75648E-21
RVB2	Putative trancription modulator; RuvB-like protein family member; heterozygous null mutant displays sensitivity to virgineone	2.3419639	5.09803E-16
orf19.6896	Predicted ORF overlapping the Major Repeat Sequence on chromosome 7; member of a family encoded by FGR6-related genes in the RB2 repeat sequence	2.3414055	0.000119545

FRP5	Protein with a GPR1/FUN34/yaaH family domain; flucytosine repressed	2.3405438	0.024601782
orf19.962	Protein with a fungal RNA polymerase I subunit RPA14 domain; proposed to play a role in the recruitment of pol I to the promoter; Hap43-induced gene	2.3378183	1.62443E-18
UTP5	Putative U3 snoRNA-associated protein; Hap43p-induced gene; mutation confers resistance to 5-fluorocytosine (5-FC), 5-fluorouracil (5-FU), and tubercidin (7-deazaadenosine); physically interacts with TAP-tagged Nop1p	2.333479	3.39499E-12
PGA44	Putative GPI-anchored protein	2.3318498	1.74367E-06
orf19.2040	Ortholog(s) have oxidoreductase activity, acting on NAD(P)H activity	2.3307177	1.33796E-06
GTS1	Putative Arf3p GTPase activating protein; Hap43p-repressed gene; possibly an essential gene, disruptants not obtained by UAU1 method	2.3294256	7.19925E-10
SMC4	Ortholog(s) have ATPase, DNA/DNA annealing activity, chromatin binding, double-stranded DNA binding, single-stranded DNA binding activity	2.3266176	1.86709E-06
orf19.3301	Putative ubiquitin ligase complex component; induced by heavy metal (cadmium) stress; Hog1-induced; transcript induced by Mnl1p under weak acid stress; flow model biofilm induced; Spider biofilm induced	2.3266122	2.23775E-05
orf19.4721	Ortholog(s) have RNA binding activity, role in mRNA processing, mitochondrial translation and mitochondrion localization	2.3257702	6.87257E-08
orf19.536	Ortholog(s) have chromatin binding, molecular adaptor activity, ubiquitin binding activity and role in chromatin organization, histone acetylation, transcription by RNA polymerase II	2.3240862	3.85996E-05
ISW2	Ortholog of S. cerevisiae Isw2; an ATPase involved in chromatin remodeling; required for chlamydospore formation; Hap43-induced gene; repressed by high-level peroxide stress	2.3229366	0.000750093
CHL4	Protein described as having role in chromosome segregation; RNA abundance regulated by tyrosol and cell density	2.3219281	0.004001113
SOL1	Cell cycle regulator; partial functional homolog of S. cerevisiae Sic1p; protein degradation regulated by SCF(CDC4) pathway; CDK phosphorylation predicted	2.3154333	6.92203E-13
orf19.7193	Specificity factor required for ubiquitination; role in protein targeting to vacuole; involved in ubiquitin-dependent protein catabolism via the multivesicular body sorting pathway; Spider biofilm induced	2.3078271	5.9474E-07
orf19.1052	Predicted histone H2B; Hap43-induced gene; Spider biofilm repressed	2.3069149	8.24507E-56
RSC58	Component of the RSC chromatin remodeling complex	2.2995199	4.85234E-08

PAC1	Ortholog(s) have microtubule binding, microtubule plus-end binding activity	2.2944165	0.000356926
orf19.928	Ortholog of *C. dubliniensis CD36* : Cd36_50610, *C. parapsilosis CDC317* : CPAR2_303690, *C. auris B8441* : B9J08_002049 and *Candida tenuis NRRL Y-1498* : CANTEDRAFT_93989	2.2934217	9.71005E-05
SOF1	Putative protein with a predicted role in 40S ribosomal subunit biogenesis; rat catheter biofilm induced	2.2889384	8.92928E-13
orf19.7067	Ortholog(s) have RNA polymerase II C-terminal domain phosphoserine binding, RNA polymerase II complex binding, transcription coregulator activity, triplex DNA binding activity	2.2869943	1.32177E-06
RRP6	Putative nuclear exosome exonuclease component; Hap43p-induced gene; mutation confers hypersensitivity to 5-fluorocytosine (5-FC), 5-fluorouracil (5-FU), and tubercidin (7-deazaadenosine)	2.2868353	1.79862E-07
orf19.5439	Ortholog of *C. dubliniensis CD36* : Cd36_80270, *C. parapsilosis CDC317* : CPAR2_504010, *Pichia stipitis Pignal* : PICST_29581, *Candida tropicalis MYA-3404* : CTRG_06175 and *Candida albicans WO-1* : CAWG_02365	2.2842169	1.37186E-20
orf19.1447	Has domain(s) with predicted DNA-binding transcription factor activity, sequence-specific DNA binding activity and role in regulation of transcription, DNA-templated	2.2809563	0.000338438
orf19.711	Protein of unknown function; induced by nitric oxide; predicted ORF from Assembly 19; removed from Assembly 20; restored based on transcription data	2.2794247	6.28652E-10
orf19.7601	Putative mitochondrial 2' O-ribose methyltransferase; predicted role in methylation of U(2791) in 21S rRNA; Spider biofilm induced	2.2792109	1.68166E-05
orf19.5051	Has domain(s) with predicted protein heterodimerization activity	2.274175	0.003124936
PRN1	Protein with similarity to pirins; induced by benomyl and in response to alpha pheromone in SpiderM medium; transcript induced by Mnl1 in weak acid stress; rat catheter and Spider biofilm induced	2.2736315	0.000285651
orf19.7103	Predicted exonuclease; Spider biofilm induced	2.2730714	1.53776E-07
orf19.1666	Ortholog of Dig2, a MAP kinase-responsive inhibitor of Ste12; regulates mating-specific genes and invasive growth in S. cerevisiae; induced by alpha pheromone in SpiderM medium	2.272419	1.50337E-05
KCH1	Ortholog of Kch1 a potassium transporter; mediates K+ influx and activates high-affinity Ca2+ influx system during mating pheromone response in S. cerevisiae; induced by alpha pheromone in SpiderM medium	2.2722159	6.01989E-06
AHC1	Ortholog(s) have histone acetyltransferase activity, role in histone acetylation, replication-born double-strand break	2.2700782	2.09618E-13

	repair via sister chromatid exchange and Ada2/Gcn5/Ada3 transcription activator complex localization		
SUA5	Ortholog(s) have N(6)-L-threonylcarbamoyladenine synthase activity, single-stranded telomeric DNA binding activity	2.2683442	2.69773E-20
MCM2	Phosphorylated protein of unknown function; transcription is periodic with a peak at M/G1 phase of the cell cycle	2.2666782	1.67729E-07
orf19.4895	Ortholog of *C. dubliniensis CD36* : Cd36_09650, *C. parapsilosis CDC317* : CPAR2_805060, *C. auris B8441* : B9J08_004034 and *Candida tenuis NRRL Y-1498* : CANTEDRAFT_95780	2.2660975	4.84729E-07
ELF1	Putative mRNA export protein; Walker A and B (ATP/GTP binding) motifs; required for wild-type morphology, growth; expressed in hyphal, pseudohyphal, and yeast form; Hap43-induced; Spider and flow model biofilm induced	2.265777	2.7471E-08
SFL1	Transcription factor involved in negative regulation of morphogenesis, flocculation and virulence; induced in core caspofungin response; Spider biofilm induced	2.2647032	2.78122E-06
MEF1	Ortholog(s) have role in mitochondrial translation and mitochondrion localization	2.2638251	1.97408E-19
orf19.7581	Protein with a predicted role in assembly of U2 snRNP into the spliceosome; Spider biofilm induced	2.2613812	5.76947E-08
orf19.2594	Ortholog(s) have RNA polymerase I activity and role in nucleolar large rRNA transcription by RNA polymerase I, transcription by RNA polymerase I, transcription initiation from RNA polymerase I promoter	2.259364	1.37834E-08
CTR1	Copper transporter; transcribed in low copper; induced Mac1, Tye7, macrophage interaction, alkaline pH via Rim101; 17-beta-estradiol repressed; complements S. cerevisiae ctr1 ctr3 copper transport mutant; flow model/Spider biofilm induced	2.2562592	1.76184E-27
RPA34	Putative RNA polymerase I subunit; rat catheter biofilm induced	2.2518384	6.59595E-07
TOM6	Predicted component of the TOM (translocase of outer membrane) complex, involved in protein import into mitochondria	2.2455329	6.45085E-08
orf19.5987	Ortholog(s) have tRNA (guanine-N2-)-methyltransferase activity, role in tRNA methylation and cytoplasm, tRNA (m2G10) methyltransferase complex localization	2.2438107	1.85339E-07
YRB2	Ortholog(s) have role in protein export from nucleus, regulation of chromatin silencing at telomere, ribosomal small subunit export from nucleus and cytosol, nucleus localization	2.2411637	2.02005E-09
NDT80	Ortholog of Ndt80; meiosis-specific transcription factor; activator of CDR1 induction by antifungal drugs; required for wild-type drug resistance and for Spider biofilm formation; transcript induced by antifungal drug treatment	2.2392533	1.3003E-23

RRP15	Putative nucleolar protein; constituent of pre-60S ribosomal particles; Hap43-induced; repressed by prostaglandins	2.2354668	3.00991E-21
orf19.6049	Ortholog(s) have protein kinase activator activity and role in chromosome segregation, protein phosphorylation, regulation of cytokinesis	2.2354257	6.30488E-05
orf19.3170	Ortholog(s) have role in RNA polymerase I assembly, RNA polymerase II core complex assembly, RNA polymerase III assembly, formation of translation preinitiation complex and cytoplasm localization	2.229191	0.000190644
MGT1	Putative DNA repair methyltransferase; induced by nitric oxide independent of Yhb1; Spider biofilm induced	2.2259563	4.3682E-30
BRR2	Predicted RNA-dependent ATPase RNA helicase; Hap43-induced gene	2.2168114	9.01289E-07
CEF1	Putative mRNA splicing factor; ortholog is essential in S. cerevisiae; Spider biofilm induced	2.2129937	0.001077512
orf19.2506	Protein of unknown function; opaque-specific transcript; induced during chlamydospore formation in both C. albicans and C. dubliniensis; Hog1-repressed; Spider biofilm induced	2.210545	3.72043E-07
HTA1	Histone H2A; repressed in fkh2 mutant; amphotericin B repressed; farnesol regulated; RNA abundance regulated by tyrosol and cell density; Hap43-induced gene; Spider biofilm repressed	2.2090315	1.58145E-46
CFL2	Oxidoreductase; iron utilization; Sfu1/Sef1/Hap43/Nrg1/Tup1/Rim101 regulated; alkaline/low iron/fluphenazine/ciclopirox olamine, flucytosine, fluconazole, Spider/flow model/rat catheter biofilm induced; caspofungin/amphotericin B repressed	2.2080433	0.00013281
URA7	CTP synthase 1; flucytosine induced; protein present in exponential and stationary growth phase yeast cultures	2.2079297	1.23651E-14
FGR50	Protein lacking an ortholog in S. cerevisiae; transposon mutation affects filamentous growth; Spider biofilm repressed	2.2031605	2.07146E-05
orf19.2520	Ortholog(s) have structural constituent of ribosome activity and mitochondrial small ribosomal subunit localization	2.1984858	8.44276E-20
EVP1	Putative plasma membrane protein; predicted role in cell wall integrity; regulated by Nrg1, Tup1; induced during chlamydospore formation in both C. albicans and C. dubliniensis	2.1965354	3.73269E-08
orf19.932	Putative aminophospholipid translocase (flippase); merged with orf19.2226 in Assembly 21; possibly an essential gene, disruptants not obtained by UAU1 method	2.1951476	5.19877E-06
DEP1	Ortholog(s) have role in histone deacetylation, negative regulation of chromatin silencing at telomere and negative regulation of ribosomal DNA heterochromatin assembly	2.1946021	1.01925E-06

NGG1	Ortholog(s) have histone acetyltransferase activity and role in chromatin organization, histone acetylation, regulation of transcription by RNA polymerase II	2.1943051	3.95635E-07
NTO1	Putative histone acetyltransferase complex subunit; induced upon low-level peroxide stress; Spider biofilm induced	2.1929802	6.71368E-05
orf19.3751	Putative serine/threonine protein kinase; possibly an essential gene, disruptants not obtained by UAU1 method	2.1929417	0.000226379
CTA8	Essential transcription factor, mediates heat shock transcriptional induction; in the absence of heat stress, Cta8p levels are modulated by growth temperature to regulate basal expression of genes involved in protein folding	2.1920032	1.98537E-05
MSW1	Protein similar to S. cerevisiae Msw1p, which is mitochondrial tryptophanyl-tRNA synthetase; Hap43p-repressed gene; likely to be essential for growth, based on an insertional mutagenesis strategy	2.1849483	5.36308E-14
orf19.5126	Putative adhesin-like protein	2.1843618	1.38821E-10
ECE1	Candidalysin, cytolytic peptide toxin essential for mucosal infection; hypha-specific protein; regulated by Rfg1, Nrg1, Tup1, Cph1, Efg1, Hog1, farnesol, phagocytosis; fluconazole-induced; rat catheter and Spider biofilm induced	2.1812888	6.74917E-21
MAC1	Copper fist transcription factor; regulator of CTR1 copper transporter; induces CTR1 in low-copper conditions; required for copper regulation of SOD1 and SOD3; Spider biofilm induced	2.1799845	2.54092E-12
orf19.1600	Ortholog of *C. dubliniensis CD36* : Cd36_23530, *C. parapsilosis CDC317* : CPAR2_406140, *C. auris B8441* : B9J08_003025 and *Candida tenuis NRRL Y-1498* : CANTEDRAFT_116829	2.1790368	4.36945E-12
orf19.7376	Ortholog of *C. dubliniensis CD36* : Cd36_85880, *C. parapsilosis CDC317* : CPAR2_806870, *C. auris B8441* : B9J08_002007 and *Candida tenuis NRRL Y-1498* : CANTEDRAFT_96983	2.1770848	0.003368775
orf19.2260	Putative transcription factor with zinc finger DNA-binding motif	2.1770319	1.02833E-06
VAM3	Predicted syntaxin-like vacuolar t-SNARE, involved in vacuolar inheritance	2.1708982	1.10846E-09
SEF1	Zn2-Cys6 transcription factor; regulates iron uptake; negatively regulated by Sfu1p, positively regulated by Tbf1; promotes virulence in mice; mutants display decreased colonization of mouse kidneys; Spider biofilm induced	2.169925	4.17324E-08
orf19.6017	Protein of unknown function; Spider biofilm induced	2.169925	0.019690458
SOD3	Cytosolic manganese-containing superoxide dismutase; protects against oxidative stress; repressed by ciclopirox olamine, induced during stationary phase when SOD1 expression is low; Hap43-repressed; Spider and flow model biofilm induced	2.1697089	8.94593E-39

DBP3	Putative ATP-dependent DEAD-box RNA helicase; Hap43-induced; repressed by prostaglandins; Spider biofilm induced	2.1677727	4.31264E-10
DST1	Ortholog(s) have RNA polymerase II complex binding, RNA polymerase II complex recruiting activity	2.166452	2.9964E-14
orf19.5566	Ortholog(s) have role in protein lipoylation and mitochondrion localization	2.166221	0.002171425
orf19.4455	Protein of unknown function; Spider biofilm induced	2.162583	5.92611E-05
orf19.6871	Protein of unknown function; Hap43-induced; flow model biofilm repressed	2.1620198	0.000241678
orf19.985	Ortholog of *S. cerevisiae* : YEL043W, *C. glabrata CBS138* : CAGL0L01221g, *C. dubliniensis CD36* : Cd36_09830, *C. parapsilosis CDC317* : CPAR2_804210 and *C. auris B8441* : B9J08_004773	2.1572139	4.43548E-06
HAP5	Component of CCAAT-binding transcription factor; roles in filamentous growth, low-iron induction of FRP1; regulates genes involved in respiratory growth; functional homolog of S. cerevisiae Hap5p; Cap1p-dependent expression in low iron	2.15591	1.17785E-10
SKO1	bZIP transcription factor involved in cell wall damage response; represses the yeast-to-hypha transition; mutants are caspofungin sensitive; induced by osmotic stress via Hog1; activated by Rlm1p; induced by Mnl1 under weak acid stress	2.1558048	4.89147E-15
SET6	Ortholog of S. cerevisiae Set6, a SET domain protein of unknown function; Hap43-induced; rat catheter and Spider biofilm induced	2.1548396	6.40408E-06
RSM24	Ortholog(s) have structural constituent of ribosome activity and mitochondrial small ribosomal subunit localization	2.15406	2.39211E-23
TOS4	Putative fork-head transcription factor; rat catheter and Spider biofilm repressed	2.1514117	1.13114E-08
DBP9	Putative DEAD-box helicase; Hap43-induced; Spider biofilm induced	2.1499238	6.49469E-08
orf19.675	Cell wall protein; induced in core stress response and core caspofungin response; iron-regulated; amphotericin B, ketoconazole, and hypoxia induced; regulated by Cyr1, Ssn6; induced in oralpharyngeal candidasis; Spider biofilm repressed	2.147783	1.03556E-43
STE4	Beta subunit of heterotrimeric G protein of mating signal transduction pathway; required for mating; transcript is specific to cells homozygous at MTL; induced by alpha pheromone; ortholog of S. cerevisiae Ste4	2.1473824	0.021299731
HST3	Histone H3K56 deacetylase; reduced copy number increases opaque cell formation; repressed by MMS, hydroxyurea and high-levels of hydrogen peroxide; Hap43p-induced; ectopic expression blocks genotoxin-induced switching; nicotinamide target	2.145721	8.53443E-06

TAZ1	Putative lyso-phosphatidylcholine acyltransferase, required for normal phospholipid content of mitochondrial membranes; rat catheter biofilm induced	2.1450394	2.42393E-07
HOT1	Putative transcription factor; required for inhibition of filamentous growth by farnesoic acid and for expression of PHO81; filament induced	2.1420413	9.44107E-05
orf19.1955	Ortholog(s) have palmitoyltransferase activity, role in protein palmitoylation, protein targeting to membrane and endoplasmic reticulum palmitoyltransferase complex, extrinsic component of endoplasmic reticulum membrane localization	2.1399182	0.00604641
CDC5	Polo-like kinase; member of conserved Mcm1 regulon; depletion causes defects in spindle elongation and Cdc35-dependent filamentation; virulence-group-correlated expression; likely essential (UAU1 method); Spider biofilm repressed	2.1397657	1.06161E-12
TFG1	Protein similar to S. cerevisiae Tfg1p, which is part of transcription factor TFIIF; transposon mutation affects filamentous growth; possibly an essential gene, disruptants not obtained by UAU1 method	2.1383852	4.47373E-08
orf19.7291	Ortholog(s) have tRNA (adenine-N1-)-methyltransferase activity, role in tRNA methylation and nucleus, tRNA (m1A) methyltransferase complex localization	2.1349114	9.19666E-05
DBP8	Protein similar to S. cerevisiae Dbp8p, an ATP-dependent helicase involved in rRNA processing; oxidative stress-repressed via Cap1p; flucytosine repressed; likely to be essential for growth, based on insertional mutagenesis	2.1344561	4.34358E-10
CDC54	Putative pre-replication complex helicase subunit; transcript regulated by Nrg1 and Mig1; periodic mRNA expression, peak at cell-cycle M/G1 phase; Hap43-induced	2.1323282	1.91779E-05
BUD6	Protein required for Spitzenkorper formation in hyphal cells (wild-type localization of Mlc1p to the Spitzenkorper); localizes to polarisome	2.1303288	7.11911E-06
orf19.5831	Ortholog of *C. dubliniensis CD36* : Cd36_17620, *C. parapsilosis CDC317* : CPAR2_212260, *Candida tropicalis MYA-3404* : CTRG_01348, *Candida albicans WO-1* : CAWG_04043 and *Candida metapsilosis* : CMET_2351	2.1259817	0.016618579
BRF1	Component of the general transcription factor for RNA polymerase III (TFIIIB); possibly an essential gene, disruptants not obtained by UAU1 method	2.1231907	5.24781E-07
orf19.2610	Putative tRNA binding protein; intron-containing gene; Spider biofilm induced	2.1155049	9.55973E-27
orf19.4596	Protein of unknown function; Spider biofilm induced	2.1139599	1.08225E-05
RIO2	Putative serine kinase with a predicted role in the processing of the 20S pre-rRNA into mature 18S rRNA; null mutants are hypersensitive to caspofungin	2.1076547	5.351E-09

NOP14	Putative nucleolar protein; Hap43-induced; mutation confers resistance to 5-fluorocytosine (5-FC), 5-fluorouracil (5-FU), and tubercidin (7-deazaadenosine); heterozygous mutant is resistant to parnafungin; Spider biofilm induced	2.1039994	3.4058E-07
PBS2	MAPK kinase (MAPKK); role in osmotic and oxidative stress responses, oxidative stress adaptation; required for stress regulation of Hog1p localization and activity; functional homolog of S. cerevisiae Pbs2p	2.1030907	3.02828E-14
orf19.6119	Protein of unknown function; Spider biofilm repressed	2.0969786	1.26574E-05
HAS1	Functional homolog of S. cerevisiae Has1p, which is a nucleolar protein of the DEAD-box ATP-dependent RNA helicase family that is involved in biogenesis of the ribosome, particularly the small (40S) subunit; caspofungin-downregulated	2.0963501	9.28636E-15
orf19.6195	Ortholog(s) have 3'-5'-exoribonuclease activity	2.0912268	4.75488E-07
ENP2	Putative nucleolar protein; essential; heterozygous mutation confers resistance to 5-fluorocytosine (5-FC), 5-fluorouracil (5-FU), and tubercidin (7-deazaadenosine); Hap43-induced; Spider biofilm induced	2.0909085	1.42517E-05
CWC25	Putative splicing factor required for the first step of pre-mRNA splicing; Spider biofilm induced	2.0899409	7.42159E-11
DES1	Putative delta-4 sphingolipid desaturase; planktonic growth-induced gene	2.0871368	3.1143E-42
orf19.954	Putative DnaJ-like chaperone; Hap43-repressed gene	2.0835102	8.40299E-10
EST1	Telomerase subunit; allosteric activator of catalytic activity, but not required for catalytic activity; has TPR domain	2.0788044	3.86931E-38
CDC14	Protein involved in exit from mitosis and morphogenesis; ortholog of S. cerevisiae Cdc14p, which is a dual-specificity phosphatase and cell-cycle regulator; suppresses S. cerevisiae cdc15-lyt1, dbf2-2, and (partially) tem1 mutant phenotypes	2.0784887	1.95612E-15
CUP2	Putative copper-binding transcription factor; required for normal resistance to copper; activates transcription of metallothionein genes; Hap43-repressed; Spider biofilm induced	2.0782829	2.7454E-07
YTM1	Protein similar to S. cerevisiae Ytm1p, which is involved in biogenesis of the large ribosomal subunit; transposon mutation affects filamentous growth; protein level decreases in stationary phase cultures; Hap43p-induced gene	2.0757457	4.08356E-09
CAF130	Ortholog(s) have role in positive regulation of transcription elongation from RNA polymerase II promoter and CCR4-NOT core complex localization	2.0753286	1.56878E-09
MCM6	Putative MCM DNA replication initiation complex component; mRNA expression peak at cell-cycle M/G1 phase; regulated by	2.0747678	8.96474E-08

	tyrosol and cell density; repressed by alpha pheromone in SpiderM medium; Hap43-induced gene		
orf19.6919	Ortholog of *C. dubliniensis CD36* : Cd36_71210, *C. parapsilosis CDC317* : CPAR2_702710, *C. auris B8441* : B9J08_002141 and *Candida tenuis NRRL Y-1498* : CANTEDRAFT_116256	2.0733877	0.005274226
MTD1	Ortholog(s) have methylenetetrahydrofolate dehydrogenase (NAD+) activity, role in folic acid-containing compound biosynthetic process, one-carbon metabolic process, purine nucleobase biosynthetic process and cytosol localization	2.073249	2.85605E-11
POP3	Putative RNase MRP and nuclear RNase P component; decreased repressed by prostaglandins; Spider biofilm induced	2.0717502	5.69135E-11
MTO1	Putative mitochondrial protein; rat catheter biofilm induced	2.0703893	0.012692747
orf19.5425	Ortholog(s) have 3'-tRNA processing endoribonuclease activity	2.0698292	2.42408E-05
orf19.1535	Protein of unknown function; Hap43-induced gene	2.0681413	0.010123125
PIR1	1,3-beta-glucan-linked cell wall protein; N-mannosylated, O-glycosylated by Pmt1; cell wall defect in het mutant; Hog1/fluconazole/hypoxia induced; iron/Efg1/Plc1/temp regulated; flow model biofilm induced; hyphal, Spider biofilm repressed	2.0681359	8.36327E-26
LIP5	Cold-activated secreted lipase, differentially expressed lipase gene family member with possible roles in nutrition and acidic microenvironment; LIP5 and LIP8 expressed at all stages of mucosal and systemic infection; affects filamentation	2.067941	0.000661953
TPT1	tRNA 2'-phosphotransferase; enzyme of tRNA splicing; functionally complements viability of S. cerevisiae tpt1 null mutant	2.0648365	1.46833E-11
RFC1	Putative DNA replication factor C subunit; ortholog of S. cerviaie Rfc1; likely essential, based on an insertional mutagenesis strategy; rat catheter biofilm repressed	2.0592423	1.66966E-08
orf19.4628	Putative cleavage and polyadenylation factor; heterozygous null mutant exhibits hypersensitivity to parnafungin and cordycepin in the C. albicans fitness test; possibly an essential gene, disruptants not obtained by UAU1 method	2.0589769	4.8547E-09
ESA1	Subunit of the NuA4 histone acetyltransferase complex; member of MYST family; Spider biofilm induced	2.0575047	2.69637E-08
ELP3	Predicted histone acetyltransferase; role in regulation of transcription, tRNA wobble uridine modification; Spider biofilm induced	2.0573429	6.05656E-14
orf19.4787	Ortholog of *S. cerevisiae* : JJJ2, *C. glabrata CBS138* : CAGL0I06226g, *C. dubliniensis CD36* : Cd36_08750, *C. parapsilosis CDC317* : CPAR2_803690 and *C. auris B8441* : B9J08_004047	2.0544478	0.004238907

SWI6	Putative component of the MBF and SBF transcription complexes involved in G1/S cell-cycle progression; periodic mRNA expression, peak at cell-cycle G1/S phase	2.0528612	0.000235172
RPA12	Putative DNA-directed RNA polymerase I; induced upon adherence to polystyrene	2.0517406	0.001099576
BRN1	Putative condensin complex subunit; cell-cycle regulated periodic mRNA expression	2.0489727	9.78152E-05
DOT1	Putative modulator of white-opaque switching	2.0482303	4.29795E-06
CST20	Protein kinase of Ste20p/p65PAK family, required for wild-type mating efficiency and virulence in a mouse model; Cst20p-Hst7p-Cek1p-Cph1p MAPK pathway regulates some hyphal growth; involved in Cdc42p growth regulation	2.0481986	4.5117E-05
GUT1	Putative glycerol kinase; downregulated upon adherence to polystyrene; greater mRNA abundance observed in a cyr1 homozygous null mutant than in wild type	2.0471871	5.35211E-09
orf19.323	Putative haloacid dehalogenase; localized to plasma membrane	2.0471239	3.1152E-05
orf19.1873	Protein of unknown function; Hap43-repressed gene; repressed by nitric oxide	2.045083	2.84092E-17
RCK2	Predicted MAP kinase-activated protein kinase, similar to S. cerevisiae serine/threonine protein kinase Rck2p; induced by osmotic stress via Hog1p; macrophage/pseudohyphal-repressed; mutants are sensitive to rapamycin	2.0436639	8.98355E-13
CCP2	Has domain(s) with predicted heme binding, peroxidase activity and role in response to oxidative stress	2.0412927	0.001620421
YTA7	Ortholog(s) have ATPase, chromatin binding, histone binding activity	2.035545	1.54391E-05
orf19.6580	Protein of unknown function; Spider biofilm induced	2.0335897	4.47685E-09
NMA111	Putative serine protease and general molecular chaperone; macrophage-induced gene; repressed in core stress response; merged with orf19.3288.1 in Assembly 21	2.0281969	1.86947E-08
IFV1	Predicted ORF overlapping the Major Repeat Sequence on chromosome R; member of a family encoded by FGR6-related genes in the RB2 repeat sequence	2.0272598	0.009040281
orf19.1956	Ortholog(s) have role in reciprocal meiotic recombination and mitochondrial matrix, mitochondrion localization	2.0259298	5.57553E-10
HDA1	Histone deacetylase; inducer of filamentation; conserved deacetylation motif; regulates white-to-opaque switch frequency but not opaque-to-white switch; greater expression in white cells than opaque cells; inhibited by trichostatin-A	2.0241539	0.000117403
UTP6	Putative U3 snoRNA-associated protein; Hap43-induced; transposon mutation affects filamentous growth; repressed by prostaglandins	2.0214094	4.19364E-14

TAF7	Putative TFIID subunit involved in RNA polymerase II transcription initiation; possibly an essential gene, disruptants not obtained by UAU1 method	2.0152668	0.002949441
orf19.445	Protein of unknown function; repressed by prostaglandins	2.0145177	3.9462E-17
PUS4	Putative pseudouridine synthase; transcript regulated by Nrg1, Mig1, and Tup1	2.0128174	3.75148E-10
YJU2	Ortholog(s) have first spliceosomal transesterification activity and role in generation of catalytic spliceosome for first transesterification step	2.0127362	2.24812E-17
orf19.6083	Has domain(s) with predicted nucleic acid binding, nucleotide binding activity	2.0101128	9.72598E-10
TUB4	Putative gamma-tubulin; induced upon adherence to polystyrene; transcript regulated by Nrg1 and Mig1; periodic mRNA expression, peak at cell-cycle S/G2 phase	2.0096353	0.000368953
SNF12	Component of the RSC chromatin remodeling complex	2.0070104	3.35471E-11
orf19.6660	Protein of unknown function; mRNA binds to She3; Hap43-repressed; rat catheter and flow model biofilm induced	2.0051553	3.97795E-12
NMD3	Putative nonsense-mediated mRNA decay protein; repressed in core stress response; repressed by prostaglandins	2.0019314	8.63118E-13
FIP1	Puttaive pre-mRNA polyadenylation factor; heterozygous null mutant exhibits hypersensitivity to parnafungin and cordycepin in the C. albicans fitness test	1.9995551	2.41993E-16
PGA37	Putative GPI-anchored protein; Hap43-repressed; Spider biofilm induced	1.9982284	0.008161156
PRP5	Putative pre-mRNA processing RNA-helicase; induced upon adherence to polystyrene; rat catheter and Spider biofilm induced	1.9979197	0.000250254
orf19.537	Ortholog of *C. dubliniensis CD36* : Cd36_29810, *C. parapsilosis CDC317* : CPAR2_206030, *C. auris B8441* : B9J08_002565 and *Candida tenuis NRRL Y-1498* : CANTEDRAFT_102086	1.9959548	0.005155432
ARG11	Putative ornithine transporter of the mitochondrial inner membrane; induced during the mating process	1.9942374	0.003759001
JJJ3	Protein with a predicted DnaJ chaperone domain and a CSL-type zinc finger; Spider biofilm induced	1.9903405	3.00883E-05
SAT4	Ortholog of S. cerevisiae Sat4; amphotericin B induced; clade-associated gene expression; Spider biofilm induced	1.9895452	1.07529E-07
INT1	Protein structurally similar to alpha-subunit of human leukocyte integrin; role in morphogenesis, adhesion, and mouse cecal colonization and systemic virulence; similar to S. cerevisiae Bud4; Hap43-induced gene	1.9884947	4.20341E-08
orf19.4846	GlcNAc-induced protein	1.9825036	3.41038E-25
GLN3	GATA transcription factor, involved in regulation of nitrogen starvation-induced filamentous growth; regulates transcription	1.9811788	1.74256E-08

	of Mep2 ammonium permease; regulated by Gcn2 and Gcn4; mRNA binds She3; Spider biofilm induced		
orf19.871	Ortholog(s) have GTPase activator activity, role in positive regulation of GTPase activity, positive regulation of TORC1 signaling and Lst4-Lst7 complex, cytoplasm, vacuolar membrane localization	1.9797314	0.00018924
PCL1	Cyclin homolog; transcript induced by filamentous growth; induced by alpha pheromone in SpiderM medium	1.978423	7.4847E-12
SGO1	Component of the spindle checkpoint; cell-cycle regulated periodic mRNA expression	1.9781582	4.41776E-05
SRP101	Signal recognition particle (SRP) receptor alpha subunit; involved in SRP-dependent protein targeting; rat catheter biofilm repressed	1.9730732	9.21661E-09
FAL1	eIF4A subfamily of DEAD-box ATP-dependent RNA helicases; predicted nucleolar protein required for maturation of 18S rRNA; Spider biofilm induced	1.9729681	4.24697E-05
SPT7	Putative SAGA transcriptional regulatory complex subunit; mutation confers hypersensitivity to toxic ergosterol analog, and to amphotericin B	1.9711797	0.000351171
FRE7	Copper-regulated cupric reductase; repressed by ciclopirox olamine or 17-beta-estradiol; induced by alkaline conditions or interaction with macrophage; Spider biofilm induced	1.9706162	0.003182305
orf19.4702	Possible similarity to mutator-like element (MULE) transposase; flow model biofilm induced; expression regulated during planktonic growth	1.9637565	0.01755211
HHO1	Putative histone H1; farnesol regulated; Hap43-induced; contains 5' UTR intron; Spider biofilm repressed	1.9622552	1.62743E-35
HTB1	Histone H2B; induced by adherence to polystyrene; amphotericin B, caspofungin repressed; flucytosine, fluconazole induced; Efg1 regulated; slow growth, increased white-to opaque switch in ectopic expression strains; Spider biofilm repressed	1.961411	5.01124E-37
ASE1	Putative microtubule-associated protein; member of conserved Mcm1p regulon; periodic mRNA expression, peak at cell-cycle G2/M phase	1.9578745	0.007423626
HAP41	Putative Hap4-like transcription factor; Hap43-repressed; not required for response to low iron; induced by Mnl1 under weak acid stress; Spider biofilm induced	1.9573681	8.91447E-20
orf19.6855	Ortholog of *C. dubliniensis CD36* : Cd36_04370, *C. parapsilosis CDC317* : CPAR2_105410, *C. auris B8441* : B9J08_000021 and *Candida tenuis NRRL Y-1498* : CANTEDRAFT_114891	1.9516812	1.76607E-10
orf19.6166	Ortholog of *C. dubliniensis CD36* : Cd36_80810, *C. parapsilosis CDC317* : CPAR2_101800, *C. auris B8441* : B9J08_002362 and *Candida tenuis NRRL Y-1498* : CANTEDRAFT_103482	1.950247	0.003972605

orf19.3404	Protein of unknown function; transcription repressed by fluphenazine treatment	1.9488213	0.002045788
orf19.5442	Ortholog of *C. dubliniensis CD36* : Cd36_80240, *C. parapsilosis CDC317* : CPAR2_504040, *C. auris B8441* : B9J08_005021 and *Candida tenuis NRRL Y-1498* : CANTEDRAFT_130872	1.9459373	2.86365E-12
RSM7	Ortholog(s) have structural constituent of ribosome activity and mitochondrial small ribosomal subunit localization	1.9456736	3.67761E-24
RPF1	Putative nucleolar protein with a predicted role in the assembly and export of the large ribosomal subunit; essential for growth; rat catheter and Spider biofilm induced	1.944984	9.34626E-14
orf19.1582	Predicted membrane transporter; member of the drug:proton antiporter (12 spanner) (DHA1) family, major facilitator superfamily (MFS); mRNA binds She3	1.9446765	0.002954734
orf19.1897	Ortholog of *C. dubliniensis CD36* : Cd36_21640, *C. parapsilosis CDC317* : CPAR2_105040, *Candida tenuis NRRL Y-1498* : CANTEDRAFT_112352 and *Debaryomyces hansenii CBS767* : DEHA2C08008g	1.9440873	7.70774E-10
orf19.2066	Ortholog(s) have protein-lysine N-methyltransferase activity and role in peptidyl-lysine trimethylation	1.9428416	3.0913E-07
UTP22	Putative U3 snoRNP protein; Ssr1-induced; repressed by prostaglandins; heterozygous null mutant is resistant to parnafungin	1.9413295	5.8914E-07
REP1	Putative transcription factor involved in transcription of N-acetylglucosamine-inducible genes; involved in negative regulation of MDR1 transcription; mutants show increased resistance to azole drugs	1.9402667	0.009867289
CLB4	B-type mitotic cyclin; nonessential; negative regulator of pseudohyphal growth; dispensible for mitotic exit, cytokinesis; Fkh2-represed; flow model biofilm repressed; farnesol-upregulated in biofilm; reduced total RNA in clb4 mutant	1.9354597	6.90154E-05
UBP7	Ortholog(s) have thiol-dependent deubiquitinase activity and role in protein deubiquitination	1.9351346	4.94954E-07
POP5	Ortholog(s) have RNA binding, ribonuclease MRP activity, ribonuclease P activity	1.9350107	0.006215327
DUR4	Putative urea permease; fungal-specific (no human or murine homolog); possibly an essential gene, disruptants not obtained by UAU1 method	1.9332712	6.30268E-05
GYP5	Putative Rab GTPase activator; role in ER to Golgi vesicle-mediated transport; Spider biofilm induced	1.9320658	8.53986E-16
orf19.3088	bZIP transcription factor; possibly transcriptionally regulated upon hyphal formation; Hap43; F-12/CO2 early biofilm induced; Spider biofilm induced	1.9311028	2.13954E-09

orf19.3970	Putative ribosome biogenesis factor; possibly essential, disruptants not obtained by UAU1 method; rat catheter and Spider biofilm induced	1.9293091	0.000103788
orf19.3449	Ortholog(s) have ubiquitin protein ligase activity and role in histone catabolic process, histone ubiquitination, mRNA destabilization, negative regulation of gene expression	1.9289169	1.22675E-06
FGR15	Putative transcription factor with zinc finger DNA-binding motif; lacks an ortholog in S. cerevisiae; transposon mutation affects filamentous growth; caspofungin induced; mutation causes marginal increase in caspofungin sensitivity	1.9270685	0.017155438
IQG1	Actomyosin ring component at bud neck; cell-cycle regulated ser phosphorylation at CDK sites regulate association with Bni1/Bnr1, Iqg1 degradation, and ring disassembly; mutation causes cytokinetic defects; rat catheter biofilm repressed	1.9266141	5.01733E-05
NUP85	Ortholog of S. cerevisiae Nup85; a structural constituent of the nuclear pore; required for alkaline-induced hyphal morphogenesis and for SD or Spider media biofilm formation	1.925259	3.44848E-08
orf19.6113	Protein of unknown function; transcript detected on high-resolution tiling arrays	1.9236045	1.16758E-14
SPP1	Putative histone methyltransferase; induced by nitric oxide independent of Yhb1p; cell-cycle regulated periodic mRNA expression	1.9228812	0.000122676
orf19.4844	Ortholog(s) have 3'-5' RNA helicase activity, RNA binding, polynucleotide adenylyltransferase activity, protein-macromolecule adaptor activity	1.9209315	1.21757E-10
UTP13	Putative U3 snoRNA-associated protein; Hap43-induced; repressed in core stress response; physically interacts with TAP-tagged Nop1	1.9197104	1.68988E-06
orf19.1639	Has domain(s) with predicted oxidoreductase activity	1.919702	9.46344E-05
RLM1	Transcription factor required for wild-type resistance to cell wall perturbation caused by caspofungin treatment; regulates caspofungin-induced transcription of SKO1	1.9165954	3.3335E-19
FLO8	Transcription factor; required for hyphal formation and CO2 induced white-opaque switching; regulates hyphal gene expression; required for virulence in mouse systemic infection; binds Efg1p; binds Mss11p via LisH motif; has LUFS domain	1.9156702	7.78541E-12
MIF2	Centromere-associated protein; similar to CENP-C proteins; Cse4p and Mif2p colocalize at C. albicans centromeres	1.9154147	1.36853E-06
SPT2	Ortholog(s) have DNA binding, DNA secondary structure binding activity	1.9088521	3.74785E-10
DBP6	Ortholog(s) have role in maturation of 5.8S rRNA from tricistronic rRNA transcript (SSU-rRNA, 5.8S rRNA, LSU-rRNA), maturation of LSU-rRNA from tricistronic rRNA transcript (SSU-rRNA, 5.8S rRNA, LSU-rRNA)	1.9087835	1.49351E-06

SSP2	Protein similar to S. cerevisiae meiosis-specific, spore-wall-localized protein Ssp2p, which is required for wild-type outer spore wall formation in S. cerevisiae; possibly an essential gene (UAU1 method); Hap43p-repressed	1.908232	0.020181108
GCV2	Glycine decarboxylase P subunit; protein of glycine catabolism; repressed by Efg1; Hog1-induced; induced by Rim101 at acid pH; transcript induced in elevated CO2; stationary phase enriched protein	1.9058771	5.4197E-15
SPA2	Protein involved in cell polarity, Spitzenkorper formation; required for mouse virulence; localizes to hyphal tip; cell-cycle regulated localization in yeast-form cells; functional domains conserved with S. cerevisiae ; Hap43p-induced gene	1.9054755	3.96498E-06
RIA1	Putative translation elongation factor; genes encoding ribosomal subunits, translation factors, and tRNA synthetases are downregulated upon phagocytosis by murine macrophage	1.9020693	3.02669E-07
ZCF35	Zn(II)2Cys6 transcription factor; Hap43-induced; Spider biofilm induced	1.9013311	0.000511837
orf19.2547	Has domain(s) with predicted RNA binding, ribonuclease activity	1.9010694	0.009843502
SCH9	Protein kinase; involved in growth control, ribosomal protein synthesis, cell size, resistance to rapamycin,, chlamydospore formation, filamentous growth, and virulence; prevents hyphal growth in hypoxia at high CO2	1.8992997	8.93099E-07
orf19.1806	Ortholog of *C. dubliniensis CD36* : Cd36_30340, *C. parapsilosis CDC317* : CPAR2_203900, *Candida tenuis NRRL Y-1498* : CANTEDRAFT_113094 and *Debaryomyces hansenii CBS767* : DEHA2G22374g	1.8985028	0.014012414
DIM1	Putative 18S rRNA dimethylase; predicted role in rRNA modification and processing; Hap43-induced; likely to be essential for growth based on insertional mutagenesis strategy; F-12/CO2 early biofilm induced	1.8966628	1.20197E-07
orf19.6240	SH3-domain protein; role in barrier septum assembly involved in cell cycle cytokinesis; activates the Chs2 chitin synthase activity during cytokinesis; Spider biofilm repressed	1.8961642	0.010578066
orf19.3831	Ortholog(s) have enzyme activator activity, telomerase inhibitor activity, role in box C/D RNA 3'-end processing, negative regulation of telomere maintenance via telomerase and nucleolus, nucleoplasm localization	1.8958521	5.67533E-06
orf19.4792	Protein with a regulator of G-protein signaling domain; Plc1-regulated; Spider biofilm induced; rat catheter biofilm repressed	1.8928887	2.06618E-06
ATP14	Putative mitochondrial F1F0 ATP synthase subunit; macrophage/pseudohyphal-induced	1.8922878	9.10564E-19

RPL37	Ribosomal protein L37; Hap43-induced; Spider biofilm repressed	1.8922179	3.62484E-27
BNR1	Formin; probable role in hyphal cytoskeletal polarity; synthetic lethality if Bnr1p and Bni1p are absent	1.8917651	2.11353E-10
orf19.1250	Ortholog(s) have role in maturation of SSU-rRNA from tricistronic rRNA transcript (SSU-rRNA, 5.8S rRNA, LSU-rRNA) and nucleolus localization	1.8902736	1.75641E-08
ZPR1	Protein with putative zinc finger; regulated by Gcn4p; repressed in response to amino acid starvation (3-aminotriazole treatment); upregulation correlates with clinical development of fluconazole resistance	1.8880678	3.86379E-11
orf19.4414	Ortholog of *C. dubliniensis CD36* : Cd36_45590, *C. parapsilosis CDC317* : CPAR2_501450, *C. auris B8441* : B9J08_003351 and *Candida tenuis NRRL Y-1498* : CANTEDRAFT_105024	1.8869299	0.017157004
RAD52	Required for homologous DNA recombination, repair of UV- or MMS-damaged DNA, telomere length, UV-induced LOH; constitutive expression, MMS-induced; weakly complements S. cerevisiae rad52 mutant; slow growth, increased white-to-opaque switch	1.8835352	4.28081E-06
GCD11	Gamma subunit of translation initiation factor eIF2; involved in identification of the start codon; likely essential for growth, based on an insertional mutagenesis strategy; Spider biofilm repressed	1.8823795	7.62543E-22
RSM22	Predicted mitochondrial small ribosomal subunit; rat catheter and Spider biofilm induced	1.8755329	2.49592E-05
orf19.3956	Ortholog(s) have glutaminyl-tRNA synthase (glutamine-hydrolyzing) activity and role in endoplasmic reticulum organization, glutaminyl-tRNAGln biosynthesis via transamidation	1.8738815	1.40773E-12
FKH2	Forkhead transcription factor; morphogenesis regulator; required for wild-type hyphal transcription, cell separation, and for virulence in cell culture; mutant lacks true hyphae, is constitutively pseudohyphal; upregulated in RHE model	1.8733211	1.90661E-06
SAK1	Serine/threonine protein kinase, acts as an upstream activating factor for the SNF1 complex that regulates responses to nutrient stress; Spider biofilm induced	1.8719504	1.33993E-07
orf19.341	Putative spermidine export pump; fungal-specific	1.8713682	6.46203E-07
GST1	Putative glutathione S-transferase; upregulated in the presence of human neutrophils; expression is regulated upon white-opaque switching; increased transcription is observed upon benomyl treatment	1.870397	4.3626E-14
JIP5	Ortholog of S. cerevisiae Jip5; predicted role in biogenesis of the large ribosomal subunit; repressed in core stress response; Hap43-induced gene	1.8669118	4.60867E-06

RNH35	Putative ribonuclease H2 catalytic subunit; flucytosine induced; Spider biofilm repressed	1.8654233	1.21944E-11
FLO9	Putative adhesin-like cell wall mannoprotein; repressed during the mating process; mutation confers hypersensitivity to toxic ergosterol analog; decreased transcription is observed upon fluphenazine treatment	1.8626013	5.72632E-06
NPL6	Component of the RSC chromatin remodeling complex; Hap43-induced; Spider biofilm repressed	1.8623004	5.82692E-08
TOP2	DNA topoisomerase II; catalyzes ATP-dependent DNA relaxation and decatenation in vitro; Y842 predicted to be catalytic; functional homolog of S. cerevisiae Top2p; sensitive to amsacrine or doxorubicin; farnesol-upregulated in biofilm	1.8607304	0.000164549
OPI1	Leucine zipper transcription factor; involved in regulation of filamentous growth; has putative Opi1-Sin3 interaction domain; interacts with ScSin3, but not CaSin3	1.8606479	0.002707158
RAD30	DNA polymerase eta with role in DNA repair; required for transition to hyphal growth; down-regulation associated with azole resistance; Hap43p-repressed gene	1.8595348	3.54988E-07
CWC2	Ortholog(s) have U6 snRNA binding activity and role in mRNA cis splicing, via spliceosome, mRNA splicing, via spliceosome	1.8590931	0.000115442
HYS2	Putative DNA polymerase III (delta) subunit with a predicted role in DNA replication and DNA repair; cell-cycle regulated periodic mRNA expression	1.8588365	2.02595E-06
RET2	Delta subunit of the coatomer complex (COPI); coats Golgi-derived transport vesicles; involved in retrograde transport between Golgi and ER; interacts with Crk1 in the two-hybrid system; Spider biofilm repressed	1.8588044	1.15258E-09
orf19.6456	Ortholog of *C. dubliniensis CD36* : Cd36_72280, *C. parapsilosis CDC317* : CPAR2_703040, *C. auris B8441* : B9J08_001028 and *Candida tenuis NRRL Y-1498* : CANTEDRAFT_125842	1.8557434	7.57949E-06
FGR13	Protein encoded in retrotransposon Zorro3 with a potential zinc finger; lacks an ortholog in S. cerevisiae; transposon mutation affects filamentous growth	1.8527979	5.82916E-12
HTS1	Putative tRNA-His synthetase; downregulated upon phagocytosis by murine macrophage; stationary phase enriched protein; Spider biofilm repressed	1.8505321	2.01224E-23
MAK16	Putative constituent of 66S pre-ribosomal particles; Hap43-induced; repressed by prostaglandins; Spider biofilm induced	1.8468485	2.19658E-08
SMM1	Putative dihydrouridine synthase; Hap43-induced gene; rat catheter biofilm induced; Spider biofilm induced	1.8408607	0.000391252
orf19.5026	C2H2 transcription factor; Spider biofilm induced	1.8394913	0.002272734
orf19.2326	Ortholog(s) have role in double-strand break repair via homologous recombination, mitotic intra-S DNA damage checkpoint signaling and mitotic sister chromatid cohesion	1.8392444	0.000108656

FGR6-10	Protein lacking an ortholog in S. cerevisiae; member of a family encoded by FGR6-related genes in the RB2 repeat sequence; transposon mutation affects filamentous growth	1.8383467	0.001626091
orf19.7107	Ortholog(s) have role in ribosomal large subunit biogenesis and cytoplasm, nucleus localization	1.8363925	1.77956E-15
BRG1	Transcription factor; recruits Hda1 to hypha-specific promoters; Tn mutation affects filamentation; Hap43-repressed; Spider and flow model biofilm induced; required for Spider biofilm formation; Bcr1-repressed in RPMI a/a biofilms	1.8314359	5.59969E-19
ash2	Ortholog(s) have histone methyltransferase activity (H3-K4 specific) activity	1.8313277	1.29798E-11
WAR1	Zn(II)2Cys6 transcription factor; plays a role in resistance to weak organic acids; required for yeast cell adherence to silicone substrate; Spider biofilm induced	1.8308727	0.022603856
RPS25A	Ribosomal protein; macrophage/pseudohyphal-induced after 16 h; repressed upon phagocytosis by murine macrophage; transcript positively regulated by Tbf1; 5'-UTR intron; Hap43-induced; Spider biofilm repressed	1.8307342	3.96828E-37
MRPL40	Putative mitochondrial ribosomal protein; Spider biofilm repressed	1.8296727	3.83422E-20
KSP1	Putative serine/threonine protein kinase; mRNA binds She3 and is localized to hyphal tips; mutation confers hypersensitivity to amphotericin B	1.8270373	0.000530423
GUF1	Ortholog(s) have GTPase activity, mitochondrial ribosome binding activity, role in positive regulation of translation and mitochondrial matrix localization	1.8184526	0.002367973
orf19.4878	Protein of unknown function; Hap43-repressed; rat catheter biofilm repressed	1.8180538	0.000118993
orf19.5688	Predicted ORF from Assembly 19; removed from Assembly 20; subsequently reinstated in Assembly 21 based on comparative genome analysis	1.8131006	9.67571E-30
SNF2	Protein involved in transcriptional regulation; ortholog of S. cerevisiae Snf2p, which is the catalytic subunit of the SWI/SNF chromatin remodeling complex; interacts with Swi1p; SWI/SNF complex is essential for hyphal growth and virulence	1.8128551	0.000592374
orf19.3501	S. cerevisiae ortholog Pxl1 localizes to sites of polarized growth and is required for selection and/or maintenance of polarized growth sites; Hog1p-repressed	1.8116423	0.001962023
KAR3	Kinesin-like microtubule motor protein; required for nuclear fusion during mating; C-terminal motor domain; mutants are viable; null has high-frequency white-opaque switching phenotype	1.8114018	0.001830621

POL1	Putative DNA directed DNA polymerase alpha; RNA abundance regulated by cell cycle, tyrosol and cell density; rat catheter biofilm induced	1.8086345	1.59212E-06
AGE2	Ortholog(s) have GTPase activator activity and role in endoplasmic reticulum to Golgi vesicle-mediated transport, intra-Golgi vesicle-mediated transport	1.8051341	2.70356E-06
DUS1	Predicted tRNA dihydrouridine synthase; Spider biofilm induced	1.8040892	2.97799E-07
PKH2	Putative serine/threonine protein kinase; predicted role in sphingolipid-mediated signaling pathway that controls endocytosis; mRNA binds She3 and is localized to hyphal tips	1.7995161	0.001538346
RPN5	Putative COP9 signalosome component; macrophage/pseudohyphal-repressed	1.7977149	4.92106E-19
MPH1	Protein similar to S. cerevisiae Mph1p, which is a DNA helicase involved in DNA repair; induced under hydroxyurea treatment	1.7975739	0.010206446
orf19.3289	Phosphorylated protein of unknown function	1.7942929	7.01516E-07
orf19.3087.2	Ortholog of *C. dubliniensis CD36* : Cd36_26300, *Candida tenuis NRRL Y-1498* : cten_CGOB_00015, *Debaryomyces hansenii CBS767* : DEHA2G16104g and *Pichia stipitis Pignal* : psti_CGOB_00212	1.792658	0.019936561
orf19.6681	Protein of unknown function; Spider biofilm induced	1.7926012	0.000795328
orf19.3470	Putative flavodoxin; similar to S. cerevisiae Tyw1, an iron-sulfur protein required for synthesis of wybutosine modified tRNA; predicted Kex2p substrate; Spider biofilm induced	1.7916109	2.35879E-05
ALS1	Cell-surface adhesin; adhesion, virulence, immunoprotective roles; band at hyphal base; Rfg1, Ssk1, Spider biofilm induced; flow model biofilm repressed; CAI-4 strain background effects; promoter bound Bcr1, Tec1, Efg1, Ndt80, and Brg1	1.7865964	1.33343E-05
DUR1,2	Urea amidolyase; hydrolyzes urea to CO2; use of urea as N source and for hyphal switch in macrophage; regulated by Nrg1/Hap43; required for virulence; promotes mouse kidney and brain colonization; rat catheter and flow model biofilm induced	1.7857249	0.000896803
ECM32	Ortholog(s) have DNA helicase activity, role in regulation of translational termination and cytoplasmic stress granule, polysome localization	1.7843544	0.000144022
LIG1	tRNA ligase; functional homolog of S. cerevisiae Trl1	1.7838847	1.88433E-05
CDH1	Protein involved in regulation of mitosis; similar to S. cerevisiae Cdh1, which is an APC/C component; transcriptionally induced by Mnl1 under weak acid stress	1.7834932	2.90951E-05
ZCF3	Zn(II)2Cys6 domain transcription factor; required for filamentous growth, resistance to rapamycin and flucytosine; possibly an essential gene, disruptants not obtained by UAU1	1.7832969	8.45423E-19

	method; Hap43-repressed; Spider and flow model biofilm induced		
SMC5	Protein similar to S. cerevisiae Smc5p, which is involved in DNA repair; transposon mutation affects filamentous growth	1.7831413	0.000824297
orf19.5274	Ortholog of *S. cerevisiae* : SKG3, *C. dubliniensis CD36* : Cd36_11180, *C. parapsilosis CDC317* : CPAR2_206250, *C. auris B8441* : B9J08_005263 and *Debaryomyces hansenii CBS767* : DEHA2E13794g	1.7819038	2.10235E-05
CAS4	RAM cell wall integrity signaling network protein; cell separation, azole sensitivity; needed for hyphal growth; insertion mutation near 3' end of gene increases caspofungin sensitivity; pheromone/hyphal induced; flow biofilm repressed	1.7815512	4.73426E-05
NCL1	Ortholog(s) have tRNA (cytosine-5-)-methyltransferase activity, tRNA binding activity	1.7787272	4.30148E-07
ECM2	Putative pre-mRNA splicing factor; intron in 5'-UTR; possibly an essential gene, disruptants not obtained by UAU1 method	1.7751849	1.44724E-08
orf19.4706	Protein of unknown function; induced in cyr1 or ras1 mutant; induced by fluconazole, by alpha pheromone in SpiderM medium and during oralpharyngeal candidasis; Spider biofilm induced	1.7735912	2.98553E-15
PWP1	Putative rRNA processing protein; Hap43-induced; repressed in core stress response	1.7735804	7.8713E-05
orf19.2314	Protein with a role in nucleolar integrity and processing of pre-rRNA; mutation confers hypersensitivity to 5-fluorocytosine (5-FC), 5-fluorouracil (5-FU), and tubercidin (7-deazaadenosine); Hap43-induced; Spider biofilm induced	1.7735567	9.78686E-05
orf19.5681	Ortholog of *C. dubliniensis CD36* : Cd36_50190, *C. parapsilosis CDC317* : CPAR2_302680, *C. auris B8441* : B9J08_004528 and *Candida tenuis NRRL Y-1498* : CANTEDRAFT_109949	1.7726792	1.37905E-15
orf19.6769	Has domain(s) with predicted intracellular anatomical structure localization	1.770228	0.001086024
orf19.5365	S. cerevisiae ortholog YMR259C interacts with Trm7 for 2'-O-methylation of C32 of substrate tRNAs; downregulated by fluphenazine treatment or in an azole-resistant strain that overexpresses CDR1 and CDR2	1.7688497	1.01903E-06
orf19.4574	Ortholog(s) have lysophospholipase activity, role in lipid homeostasis and lipid droplet localization	1.7687951	0.021202313
orf19.3473	Ortholog(s) have enzyme activator activity, histone acetyltransferase activity, structural molecule activity	1.766913	6.52379E-05
ATG18	Ortholog(s) have phosphatidylinositol-3,5-bisphosphate binding, phosphatidylinositol-3-phosphate binding, phosphatidylinositol-4-phosphate binding, ubiquitin binding activity	1.7652679	1.33931E-05
BUD23	Putative methyltransferase; Hap43-induced; repressed by prostaglandins	1.7649216	1.16168E-09

REX4	Putative RNA exonuclease; induced in a ssr1 null mutant	1.7641366	0.002768554
orf19.6843	Ortholog(s) have role in CENP-A containing nucleosome assembly, chromatin maintenance and nucleoplasm localization	1.7636733	2.42038E-06
ENT2	Epsin, involved in endocytosis; mutants are defective in hyphal growth and virulence	1.763342	0.000123792
NOC4	Putative nucleolar protein; Hap43-induced; mutation confers resistance to 5-fluorocytosine (5-FC), 5-fluorouracil (5-FU), and tubercidin (7-deazaadenosine); Spider biofilm induced	1.7620065	3.42768E-07
PGA4	GPI-anchored cell surface protein; beta-1,3-glucanosyltransferase with similarity to the A. fumigatus GEL family; transcript induced in RHE model of oral candidiasis; fluconazol-induced	1.7614947	7.91806E-27
MIS12	Mitochondrial C1-tetrahydrofolate synthase precursor	1.7596185	1.94939E-27
orf19.7254	Ortholog(s) have role in mRNA metabolic process, mitochondrial translational initiation and extrinsic component of membrane, mitochondrial inner membrane localization	1.757455	3.10575E-11
SNT1	Ortholog of S. cerevisiae Snt1; an NAD-independent histone deacetylase; heterozygous transposon mutation affects filamentous growth; null mutants are viable	1.7572623	7.64232E-06
ULP3	SUMO deconjugation enzyme that removes the small ubiquitin-like modifier (SUMO) from proteins	1.7564134	0.016303195
orf19.1285	Plasma membrane-localized protein of unknown function; Hap43p-repressed gene	1.755194	6.16005E-06
orf19.4021	Ortholog of *C. dubliniensis CD36* : Cd36_54870, *C. parapsilosis CDC317* : CPAR2_100250, *Debaryomyces hansenii CBS767* : DEHA2D13992g and *Pichia stipitis Pignal* : PICST_40400	1.7549141	3.67749E-26
orf19.1479	Ortholog of the mitochondria localized S. cerevisiae Pib2 protein of unknown function; has a FYVE zinc finger domain; Spider biofilm induced	1.7548875	0.002569406
ATG15	Putative lipase; fungal-specific (no human or murine homolog); Hap43p-repressed gene	1.7544635	2.17293E-11
orf19.3826	Predicted transmembrane protein with a role in cell wall polymer composition; Plc1-regulated; Spider biofilm induced	1.7523085	1.62417E-05
orf19.3635	Ortholog of *C. dubliniensis CD36* : Cd36_22580, *C. parapsilosis CDC317* : CPAR2_406960, *C. auris B8441* : B9J08_005066 and *Candida tenuis NRRL Y-1498* : CANTEDRAFT_113677	1.7519159	0.003276059
NUP49	Nuclear pore protein	1.7511922	6.3098E-05
orf19.6170	Ortholog(s) have microtubule binding, microtubule plus-end binding activity	1.7506332	4.74199E-05
CYC3	Mitochondrial cytochrome c heme lyase; antigenic cell-wall protein; filament induced; induced on polystyrene adherence, interaction with macrophage; N-glycosylation, 2 heme-binding motifs; rat catheter, Spider biofilm induced	1.7476341	3.70988E-20

SIZ1	Possible SUMO/Smt3 ligase; Rim101-repressed	1.7466009	0.000125662
RML2	Ortholog(s) have structural constituent of ribosome activity, role in mitochondrial translation and mitochondrial large ribosomal subunit localization	1.7456655	6.38559E-17
RRN3	Protein with a predicted role in recruitment of RNA polymerase I to rDNA; caspofungin induced; flucytosine repressed; repressed in core stress response; repressed by prostaglandins	1.7454977	7.19624E-22
ARP5	Ortholog(s) have 3'-5' DNA helicase activity	1.743714	1.0844E-07
orf19.90	Ortholog of *C. dubliniensis CD36* : Cd36_60900, *C. parapsilosis CDC317* : CPAR2_603130, *C. auris B8441* : B9J08_003439 and *Candida tenuis NRRL Y-1498* : CANTEDRAFT_125757	1.7408514	0.008754471
YTA6	Protein similar to S. cerevisiae Yta6p ATPase but ortholog of S. cervisiae Sap1; transposon mutation affects filamentous growth; induced by Mnl1 under weak acid stress	1.7397096	0.000329873
orf19.53	Ortholog(s) have role in mRNA cis splicing, via spliceosome and U2-type prespliceosome, U2AF complex, commitment complex localization	1.7389218	0.000831101
orf19.2478	Ortholog of *C. dubliniensis CD36* : Cd36_05410, *C. parapsilosis CDC317* : CPAR2_107480, *C. auris B8441* : B9J08_004575 and *Candida tenuis NRRL Y-1498* : CANTEDRAFT_94695	1.7387953	0.008199966
orf19.6414	Ortholog of *C. dubliniensis CD36* : Cd36_34040, *C. parapsilosis CDC317* : CPAR2_205850, *C. auris B8441* : B9J08_000124 and *Candida tenuis NRRL Y-1498* : CANTEDRAFT_132607	1.735997	1.93249E-05
NRM1	Transcriptional regulator of cell cycle gene expression; regulates expression of genes involved in DNA replication stress; interacts with Swi4 and Swi6; repressed during core stress response; Spider biofilm repressed	1.7335851	0.001685654
GDE1	Glycerophosphocholine phosphodiesterase; mutation confers hypersensitivity to 5-fluorouracil (5-FU); F-12/CO2 early biofilm induced	1.7302254	0.003096261
DOT4	Protein similar to ubiquitin C-terminal hydrolase; localizes to cell surface of hyphal cells, but not yeast-form cells; repressed upon high-level peroxide; Hap43p-induced; rat catheter biofilm induced	1.7294112	0.000108404
orf19.5879	Has domain(s) with predicted oxidoreductase activity and role in metabolic process	1.7289391	0.002059109
IRS4	Protein with roles in cell wall integrity, systemic (but not oral) murine infection, adherence, hyphal growth, and agar-embedded filamentous growth; antigenic in human infection; similar to S. cerevisiae Irs4p	1.7287049	5.85333E-06
orf19.3660	Ortholog of *C. dubliniensis CD36* : Cd36_60600, *C. parapsilosis CDC317* : CPAR2_602890, *C. auris B8441* : B9J08_001866 and *Candida tenuis NRRL Y-1498* : CANTEDRAFT_135512	1.7212971	0.005475787

NAM9	Putative mitochondrial ribosomal component of the small subunit; possibly an essential gene, disruptants not obtained by UAU1 method; Spider biofilm repressed	1.7209559	3.13294E-14
orf19.5722	Ortholog(s) have DNA binding activity, role in rDNA heterochromatin assembly, termination of RNA polymerase I transcription and nucleolus, rDNA heterochromatin localization	1.7186973	0.001679358
SSQ1	Protein involved in intracellular sequestering of iron ion and mitochondrial iron-sulfur cluster assembly; repression leads to defects in respiratory growth, activation of autophagy, attenuated virulence	1.7163529	0.000195404
PRA1	Cell surface protein that sequesters zinc from host tissue; enriched at hyphal tips; released extracellularly; binds to host complement regulators; mediates leukocyte adhesion and migration; immunogenic in mouse; produced at ambient pH	1.7161351	0.025891855
orf19.4818	Protein of unknown function; Spider biofilm induced	1.7154754	1.31547E-09
KRE30	YEF3-subfamily ABC family protein; predicted not to be a transporter; repressed in core stress response; mutation confers hypersensitivity to amphotericin B	1.7126628	1.28428E-17
orf19.3048	Protein of unknown function; repressed by alpha pheromone in SpiderM medium	1.7120744	0.011044965
orf19.4946	Ortholog of *C. dubliniensis CD36* : Cd36_12240, *C. parapsilosis CDC317* : CPAR2_801750, *C. auris B8441* : B9J08_002031, *Candida tenuis NRRL Y-1498* : CANTEDRAFT_135403 and *Pichia stipitis Pignal* : PICST_29111	1.710354	0.000849628
orf19.6506	Ortholog(s) have role in histone deacetylation, negative regulation of antisense RNA transcription and positive regulation of transcription by RNA polymerase II	1.7103396	0.002910624
CTF1	Putative zinc-finger transcription factor, similar to A. nidulans FarA and FarB; activates genes required for fatty acid degradation; induced by oleate; null mutant displays carbon source utilization defects and slightly reduced virulence	1.7081107	0.005195746
orf19.3928	Putative transcription factor with zinc finger DNA-binding motif	1.7079637	6.36193E-05
orf19.828	Putative ribosomal protein, large subunit, mitochondrial precursor; repressed by prostaglandins; Spider biofilm repressed	1.7079597	1.01418E-20
orf19.1360	Ortholog(s) have role in mitochondrial genome maintenance, mitochondrion organization	1.7064866	0.000625994
WOR4	Predicted C2H2 zinc finger protein, involved in transcriptional regulation of white-opaque phenotypic switching; activator of the opaque cell type	1.7056491	5.82095E-17
orf19.2143	Ortholog(s) have tRNA (guanosine-2'-O-)-methyltransferase activity, role in endocytic recycling, tRNA methylation, wobble	1.7052876	6.7974E-18

	position ribose methylation and cytoplasm, endosome localization		
PIM1	ATP-dependent Lon protease; role in degradation of misfolded proteins in mitochondria, biogenesis and maintenance of mitochondria; rat catheter biofilm induced	1.7015381	3.17528E-07
orf19.7006	Ortholog(s) have role in reciprocal meiotic recombination	1.7012322	0.001277467
APM3	Phosphorylated protein of unknown function; transcript is upregulated clinical isolates from HIV positive patients with oral candidiasis	1.6975163	0.00019886
GCD2	Putative translation initiation factor; genes encoding ribosomal subunits, translation factors, and tRNA synthetases are downregulated upon phagocytosis by murine macrophage	1.6940399	1.39815E-10
IMP2	Predicted subunit of the mitochondrial inner membrane peptidase complex involved in protein targeting to mitochondria	1.6913931	1.33056E-10
orf19.5356	Protein with a predicted role in cell wall integrity; repressed in core stress response	1.6846001	3.59787E-10
RPA190	Putative RNA polymerase I subunit A190; Hap43p-induced gene; flucytosine induced	1.6838159	5.50365E-07
RLR1	Putative THO complex subunit; possibly an essential gene, disruptants not obtained by UAU1 method; protein newly produced during adaptation to the serum	1.6829523	0.002511375
ELG1	Putative subunit of an alternative replication factor C complex; role in DNA replication, genome integrity, homologous recombination-mediated repair and telomere homeostasis	1.6816666	0.004745715
PSR1	Predicted plasma membrane associated protein phosphatase; required for normal filamentous growth; mRNA binds She3 and is localized to hyphal tips	1.6799299	2.49888E-06
ELP2	Similar to S. cerevisiae Elp2, an Elongator complex subunit required for modification of wobble nucleosides in tRNA; repressed in core stress response	1.6796647	2.35365E-06
GRR1	F-box protein component of the SCF ubiquitin-ligase complex required for cell cycle progression; involved in negative control of pseudohyphal growth; regulates stability of Ume6p in response to CO2	1.6796212	2.70845E-05
RPO26	Putative RNA polymerase subunit; heterozygous null mutant exhibits resistance to parnafungin in the C. albicans fitness test	1.6757669	8.30921E-17
orf19.1864	Ortholog(s) have role in proteasome-mediated ubiquitin-dependent protein catabolic process, rescue of stalled ribosome, ribosome-associated ubiquitin-dependent protein catabolic process and RQC complex localization	1.6756814	0.000174828
POL3	Large subunit of DNA polymerase III; partially complements defects of an S. cerevisiae cdc2 mutant; differing reports about	1.6752116	0.000140093

	periodic (G1/S) or non-periodic mRNA expression through cell cycle; Hap43p-repressed		
orf19.216.1	Ortholog of *C. parapsilosis CDC317* : CPAR2_806510, *Candida tenuis NRRL Y-1498* : CANTEDRAFT_109089, *Debaryomyces hansenii CBS767* : DEHA2B13508g and *Pichia stipitis Pignal* : PICST_59544	1.6724416	0.001584176
HOF1	Protein involved in cytokinesis and DNA damage response; interacts genetically with Rad53p-dependent checkpoint; mutant is viable	1.6713309	2.40984E-08
BUB1	Putative cell cycle checkpoint kinase; mutation confers increased sensitivity to nocodazole	1.6706477	6.24321E-05
BSD2	Ortholog(s) have role in metal ion transport, protein targeting to vacuole, ubiquitin-dependent protein catabolic process and endoplasmic reticulum, fungal-type vacuole, fungal-type vacuole membrane localization	1.6703204	5.96633E-06
FGR44	Protein lacking an ortholog in S. cerevisiae; transposon mutation affects filamentous growth	1.6676327	2.7644E-07
orf19.2333	Ortholog(s) have role in retrograde transport, endosome to Golgi and cytoplasm, late endosome localization	1.6674524	4.91547E-10
SHE3	mRNA-binding protein that localizes specific mRNAs to daughter yeast cells and to hyphal tips; required for normal filamentation and host epithelial cell damage; ortholog of S. cerevisiae She3 but target mRNAs differs	1.6671664	0.001586218
SME1	Ortholog(s) have splicing factor binding activity, role in mRNA splicing, via spliceosome and U1 snRNP, U2 snRNP, U2-type prespliceosome, U4/U6 x U5 tri-snRNP complex, U5 snRNP, post-mRNA release spliceosomal complex localization	1.6661687	2.38981E-05
GLO3	Putative ARF GTPase activator; role in COPI coating of Golgi vesicle, ER to Golgi vesicle-mediated transport, retrograde Golgi to ER vesicle-mediated transport; Spider biofilm repressed	1.6643199	8.39709E-17
PBP2	Putative RNA binding protein; transcript regulated by Nrg1, Mig1, and Tup1	1.6635081	2.49968E-12
IFH1	Transcription factor; forms a heterodimer with Fhl11 that is tethered to promoters by Tbf1; positively regulates rRNA and ribosomal protein gene transcription; Spider biofilm induced	1.6623754	6.65102E-05
orf19.3578	Ortholog of *C. dubliniensis CD36* : Cd36_19950, *C. parapsilosis CDC317* : CPAR2_104135, *Candida tenuis NRRL Y-1498* : cten_CGOB_00075 and *Debaryomyces hansenii CBS767* : DEHA2F07964g	1.6620616	0.016535756
RLI1	Member of RNase L inhibitor (RLI) subfamily of ABC family; predicted not to be a transporter; regulated by Sef1p, Sfu1p, and Hap43p	1.6590151	6.28533E-15
SSN6	Functional homolog of S. cerevisiae Cyc8/Ssn6; hyphal growth regulator; repressed during hyphal growth; Ssn6 and Tup1	1.6587827	1.41985E-05

	regulate distinct sets of genes; overexpression or mutation causes avirulence in mouse IV infection; TPR motifs		
orf19.4349	Protein of unknown function; transcript repressed by elevated CO2; Spider biofilm induced	1.6584998	0.000383062
LRG1	GTPase activator (GAP) that negatively controls small GTPases Cdc42p and Ras1p, involved in signaling pathway that controls morphogenesis in response to environmental signals	1.6566004	0.001224704
SIM1	Adhesin-like protein; involved in cell wall maintenance, redundant with Sun41; possibly secreted; macrophage-repressed; repressed by Rim101, Cyr1, Ras1; Spider biofilm induced	1.6557009	2.71891E-29
PGA56	Regulator of sorbose utilization; putative GPI-anchor; hypha and cell wall regeneration induced; colony morphology-related gene regulation by Ssn6; induced by Tbf1, Hap43; Spider biofilm induced	1.6536643	3.36308E-15
NOP13	Ortholog of S. cerevisiae Nop13; a nucleolar protein found in preribosomal complexes; Hap43-induced gene; rat catheter biofilm induced	1.6523682	5.48602E-07
TCO89	Putative homolog of *S. cerevisiae* Tco89p, which is a component of the TOR C1 complex that is involved in the regulation of growth and cell wall integrity; heterozygous null mutant displays sensitivity to rapamycin	1.6511342	0.004902206
APL1	Putative beta-adaptin, large subunit of the clathrin associated protein complex (AP-2); mutation causes decreased colony wrinkling in filamentous growth-inducing conditions, but does not block hyphal formation in liquid media	1.6492516	2.03043E-05
KIP4	S. cerevisiae Smy1 ortholog; Tn mutation affects filamentous growth; filament induced; has Mob2-dependent hyphal regulation; regulated by Nrg1, Tup1; oralpharyngeal candidasis induced; Spider biofilm induced; flow model biofilm repressed	1.6486338	0.000409122
orf19.6939	Predicted ORF from Assembly 19; removed from Assembly 20; restored based on comparative genome analysis	1.6473558	3.42359E-06
MEX67	Nuclear export protein; has NTF2-like domain; interacts with Mtr2p via the NTF2-like domain	1.6470532	1.70591E-07
APC1	Putative Anaphase-Promoting Complex/Cyclosome subunit; essential for growth; periodic mRNA expression, peak at cell-cycle S/G2 phase	1.6467785	0.009002101
orf19.1697	Ortholog(s) have role in cytoplasmic translation, poly(A)+ mRNA export from nucleus and cytoplasm localization	1.6463959	1.50384E-05
SNM1	Protein similar to RNase MRP RNA binding protein; ciclopirox olamine induced; regulated by Sef1, Sfu1, and Hap43; Spider biofilm induced	1.6450123	0.003463792

CAC2	Component of the chromatin assembly factor I (CAF-1); involved in regulation of white-opaque switching frequency; macrophage-induced	1.6444424	0.000602515
PHO80	Ortholog(s) have cyclin-dependent protein serine/threonine kinase regulator activity	1.6430655	0.005745476
orf19.500	Ortholog(s) have tRNA (adenine-N1-)-methyltransferase activity, role in tRNA methylation and nucleus, tRNA (m1A) methyltransferase complex localization	1.6424128	6.19387E-05
DED81	Putative tRNA-Asn synthetase; genes encoding ribosomal subunits, translation factors, tRNA synthetases are downregulated upon phagocytosis by murine macrophage; protein enriched in stationary phase yeast cultures	1.6418458	4.19492E-12
orf19.7245	Ortholog(s) have tRNA-5-taurinomethyluridine 2-sulfurtransferase activity, role in mitochondrial tRNA wobble position uridine thiolation and mitochondrion localization	1.6412945	1.33897E-09
KIP2	Putative kinesin-related motor protein involved in mitotic spindle positioning; repressed by alpha pheromone in SpiderM medium; periodic mRNA expression, peak at cell-cycle G2/M phase	1.6405041	0.008012705
PGA55	GPI-anchored adhesin-like protein; filament induced; regulated by Nrg1, Tup1; regulated upon hyphal formation; mRNA binds to She3 and is localized to yeast-form buds and hyphal tips; induced during chlamydospore formation	1.638512	0.012514012
PUT2	Putative delta-1-pyrroline-5-carboxylate dehydrogenase; alkaline upregulated; protein present in exponential and stationary growth phase yeast cultures; flow model biofilm induced; Spider biofilm induced	1.6382348	3.36506E-05
CRZ2	C2H2 transcription factor, involved in regulation of early adaptation to murine GI tract; Rim101-repressed at pH 8; required for yeast cell adherence to silicone substrate; Spider biofilm induced	1.6371079	0.000328678
CDC60	Cytosolic leucyl tRNA synthetase; conserved amino acid and ATP binding class I signature, tRNA binding, proofreading motifs; likely essential for growth; interacts with benzoxaborole antifungals; present in exponential and stationary phase	1.6369161	4.01137E-12
RVB1	Putative chromatin remodelling complex protein; heterozygous null mutant displays sensitivity to virgineone; Spider biofilm repressed; sumoylation regulated by Hsp90p	1.6364663	2.23577E-09
CDC45	Putative DNA replication initiation factor; transcriptionally regulated by interaction with macrophage	1.6359197	0.000138969
GOA1	Protein required for respiratory growth, resistance to oxidants, chlamydospore formation, hyphal growth under some conditions, and virulence; relocalizes from the cytoplasm to the mitochondrion during oxidative or osmotic stress	1.6357617	0.000234746

PRP24	Ortholog(s) have U6 snRNA binding, snRNA binding activity, role in spliceosomal complex assembly, spliceosomal tri-snRNP complex assembly and U6 snRNP localization	1.6348877	0.01133604
GCR3	Functional homolog of S. cerevisiae Gcr3, which acts in regulation of glycolytic genes; no intron predicted, in contrast to intron in S. cerevisiae GCR3 gene	1.633461	0.000108048
TAF145	Protein similar to S. cerevisiae Taf145p, a component of RNA polymerase II transcription factor TFIID; flucytosine repressed; likely to be essential for growth, based on an insertional mutagenesis strategy	1.6333892	0.001303498
IFU2	Ortholog(s) have ribosomal large subunit binding, tRNA binding activity and role in peptide biosynthetic process, rescue of stalled ribosome, ribosome-associated ubiquitin-dependent protein catabolic process	1.6320623	0.001490488
orf19.4575	Ortholog of *S. cerevisiae* : YPL109C, *C. glabrata CBS138* : CAGL0J07018g, *C. dubliniensis CD36* : Cd36_42050, *C. parapsilosis CDC317*: CPAR2_403320 and *C. auris B8441* : B9J08_001404	1.6311521	0.006126957
orf19.5213	Putative protein of unknown function; transcript is upregulated in clinical isolates from HIV+ patients with oral candidiasis	1.6310877	3.5459E-06
BFR1	Protein involved in the maintenance of normal ploidy; S. cerevisiae ortholog cofers Brefeldin A resistance; stationary phase enriched protein; Spider biofilm repressed	1.630646	3.18977E-09
orf19.4133	Protein wth a predicted role in transcription from RNA polymerase II promoters; Spider biofilm induced	1.6299539	0.004144246
STU2	Ortholog(s) have microtubule binding, microtubule plus end polymerase, microtubule plus-end binding activity	1.629462	0.000587153
DHR2	DEAH-box ATP-dependent RNA helicase, required for 18S rRNA synthesis; rat catheter biofilm induced	1.6271085	0.008698767
orf19.989	Mitochondrial ribosomal protein of the small subunit; Spider biofilm repressed	1.6267177	7.58927E-18
orf19.3296	Ortholog of *C. dubliniensis CD36* : Cd36_01020, *C. parapsilosis CDC317* : CPAR2_110130, *C. auris B8441* : B9J08_003399 and *Candida tenuis NRRL Y-1498* : CANTEDRAFT_93515	1.6263289	0.000992396
BOI2	Putative SH3-domain-containing protein; mutation confers hypersensitivity to toxic ergosterol analog; Spider biofilm induced	1.6253855	0.000292697
ZRG17	Ortholog(s) have zinc ion transmembrane transporter activity, role in zinc ion transport and endoplasmic reticulum localization	1.6248552	0.006044586
orf19.4355	Ortholog of *C. dubliniensis CD36* : Cd36_29080, *C. parapsilosis CDC317* : CPAR2_202490, *Candida tenuis NRRL Y-1498* :	1.6244154	0.010026815

	CANTEDRAFT_112323 and *Debaryomyces hansenii CBS767* : DEHA2F19184g		
SFP1	C2H2 transcription factor involved in regulation of biofilm formation; regulates carbon source-dependent stress response; regulates response to oxidative stress; acts as repressor of START; rat catheter biofilm induced	1.621968	1.49549E-08
TRM2	Putative tRNA methyltransferase; repressed by prostaglandins; Spider biofilm induced	1.6219113	1.15993E-06
WOR3	Transcription factor; modulator of white-opaque switch; induced in opaque cells; promoter bound by Wor1; overexpression at 25 degr shifts cells to opaque state; deletion stabilizes opaque cells at higher temperatures; Spider biofilm induced	1.6204733	0.000949722
orf19.5698	Putative mitochondrial ribosomal protein of the large subunit; transcript is upregulated in clinical isolates from HIV+ patients with oral candidiasis; Spider biofilm repressed	1.6199363	2.15228E-20
CFT2	Putative mRNA cleavage and polyadenylation factor; heterozygous null mutant exhibits hypersensitivity to parnafungin and cordycepin in the C. albicans fitness test	1.6180112	5.9653E-07
REG1	Putative protein phosphatase regulatory subunit; Hap43-repressed gene; macrophage/pseudohyphal-induced; possibly regulated upon hyphal formation; flow model biofilm induced	1.614864	7.76958E-05
SUA7	Transcription factor TFIIB; required for transcription initiation and start site selection by RNA polymerase II; downregulated during planktonic growth, whereas related SUA72 is induced; induced by Tbf1; rat catheter, Spider biofilm induced	1.6137921	3.85819E-10
orf19.3547	Ortholog(s) have mRNA 3'-UTR binding, mRNA 5'-UTR binding, translation repressor activity, mRNA regulatory element binding activity and role in negative regulation of translation, protein stabilization, ribosomal large subunit biogenesis	1.6110438	1.03261E-07
orf19.4252	Ortholog(s) have protein serine/threonine kinase activity	1.611036	6.17869E-07
orf19.4668	Protein with a glycoside hydrolase domain; mutants are viable	1.611036	0.000941347
KIS1	Snf1p complex scaffold protein; similar to S. cerevisiae Gal83p and Sip2p with regions of similarity to Sip1p (ASC and KIS domain); interacts with Snf4p; mutants are hypersensitive to caspofungin and hydrogen peroxide; Hap43p-repressed gene	1.6102043	7.40891E-06
NOP10	Small nucleolar ribonucleoprotein; flucytosine induced	1.609033	0.00034026
DEM1	Putative mitochondrial exonuclease; alpha-factor induced	1.608531	3.62007E-12
SWC4	Subunit of the NuA4 histone acetyltransferase complex	1.6081633	6.06956E-06
PGA57	Putative GPI-anchored protein; Hap43p-induced gene	1.6070563	0.018720599
orf19.2512	Ortholog of *C. dubliniensis CD36* : Cd36_81000, *C. parapsilosis CDC317* : CPAR2_103050, *C. auris B8441* : B9J08_002621 and *Candida tenuis NRRL Y-1498* : CANTEDRAFT_116326	1.6059366	0.001460842

ZCF22	Predicted Zn(II)2Cys6 transcription factor	1.605374	0.023818819
orf19.2459	Protein of unknown function; mRNA binds to She3; Hap43 repressed gene; Spider biofilm induced	1.6027738	0.014204725
orf19.2850	Protein of unknown function; induced by nitric oxide independent of Yhb1p	1.6026645	0.001157311
orf19.7441	Ortholog(s) have role in lipid homeostasis, nuclear envelope organization and nuclear envelope localization	1.6025107	0.000593828
NHP6A	Putative non-histone chromatin component; RNA abundance regulated by tyrosol,cell density; Hap43-induced; amphotericin B, caspofungin repressed; 5'-UTR intron; rat catheter and Spider biofilm repressed	1.6007357	5.61379E-17
RSM18	Ortholog(s) have structural constituent of ribosome activity and mitochondrial small ribosomal subunit localization	1.5999318	2.00261E-13
orf19.1939	Ortholog(s) have mRNA 3'-UTR binding activity, role in regulation of mRNA stability, regulation of phosphate metabolic process and cytoplasm localization	1.5998779	3.06654E-07
orf19.2387	Putative tRNA-Pro synthetase; genes encoding ribosomal subunits, translation factors, and tRNA synthetases are downregulated upon phagocytosis by murine macrophage	1.5978163	1.96926E-06
orf19.841	Ortholog(s) have SNARE binding activity, role in Golgi vesicle docking, Golgi vesicle transport and Golgi membrane localization	1.5970814	0.000385181
orf19.6056	Ortholog(s) have phosphatase activity and role in dephosphorylation	1.5970472	1.65672E-07
CDC24	GDP-GTP exchange factor for Cdc42p; phosphorylated; required for maintenance of hyphal growth; misexpression blocks hyphal growth and causes avirulence in a mouse model of systemic infection; antigenic during human oral infection	1.5958219	4.47516E-05
orf19.4159	Ortholog(s) have magnesium ion transmembrane transporter activity, role in magnesium ion export from mitochondrion, magnesium ion transport and mitochondrial inner membrane localization	1.5941116	2.89081E-07
UBP12	Ubiquitin-specific protease; cleaves ubiquitin from ubiquitinated proteins; Spider biofilm induced	1.5912215	0.001608456
orf19.6730	Ortholog of *S. cerevisiae* : YCR016W, *C. glabrata CBS138* : CAGL0L07832g, *C. dubliniensis CD36* : Cd36_87480, *C. parapsilosis CDC317* : CPAR2_808240 and *C. auris B8441* : B9J08_001753	1.5892156	8.93028E-06
EFG1	bHLH transcription factor; required for white-phase cell type, RPMI and Spider biofilm formation, hyphal growth, cell-wall gene regulation; roles in adhesion, virulence; Cph1 and Efg1 have role in host cytokine response; binds E-box	1.5872857	1.23081E-06
PIN4	Protein with similarity to S. cerevisiae Pin4p; transposon mutation affects filamentous growth	1.5849625	0.001281504

MAP1	Ortholog(s) have mRNA binding, metalloaminopeptidase activity and role in negative regulation of gene expression, protein initiator methionine removal involved in protein maturation	1.5842493	6.71912E-15
orf19.4225.1	Ortholog(s) have oxoglutarate dehydrogenase (succinyl-transferring) activity, structural constituent of ribosome activity and role in 2-oxoglutarate metabolic process, tricarboxylic acid cycle	1.5819422	6.32975E-07
RIX7	Putative ATPase of the AAA family; role in ribosomal subunit export from the nucleus; mutation impairs hyphal growth and biofilm formation	1.5811157	5.49518E-10
ARL3	Putative Ras superfamily GTPase; induced by nitric oxide independent of Yhb1p	1.5794998	0.001248981
orf19.4055	Protein similar to S. cerevisiae Ybr075wp; transposon mutation affects filamentous growth; clade-associated gene expression	1.5787969	1.29518E-09
AAF1	Possible regulatory protein; possible adhesin-like; Glu-rich domain; production in S. cerevisiae increases endothelial cell adherence and flocculence; flow model biofilm, alkaline or caspofungin induced	1.5738831	8.47459E-05
TRY2	Transcription factor; regulator of yeast form adherence; required for yeast cell adherence to silicone substrate; Spider biofilm induced	1.5737984	0.000159725
orf19.6899	Putative oxidoreductase; mutation confers hypersensitivity to toxic ergosterol analog; rat catheter and Spider biofilm induced	1.5709701	1.50752E-06
orf19.6152	Ortholog of *C. dubliniensis CD36* : Cd36_32830, *C. parapsilosis CDC317* : CPAR2_205930, *C. auris B8441* : B9J08_003982 and *Candida tenuis NRRL Y-1498* : CANTEDRAFT_115846	1.5694419	0.010504305
orf19.6156	Ortholog of *S. cerevisiae* : AIM11, *C. glabrata CBS138* : CAGL0I04928g, *C. dubliniensis CD36* : Cd36_80770, *C. parapsilosis CDC317* : CPAR2_102260 and *C. auris B8441* : B9J08_002841	1.5692676	1.47309E-10
SPT8	Ortholog(s) have TBP-class protein binding, transcription coregulator activity	1.5687705	0.004170501
orf19.426	Ortholog of *C. dubliniensis CD36* : Cd36_05120, *C. parapsilosis CDC317* : CPAR2_107740, *C. auris B8441* : B9J08_004520 and *Candida tenuis NRRL Y-1498* : CANTEDRAFT_136277	1.5674041	0.014056416
DIA4	Ortholog(s) have serine-tRNA ligase activity, role in mitochondrial seryl-tRNA aminoacylation, spore germination and mitochondrion localization	1.5620474	8.10929E-05
PRP18	snRNP U5 splicing factor component; involved in positioning the 3' splice site during the 2nd catalytic step of splicing; Spider biofilm induced	1.5585676	2.35227E-08

UBA1	Ubiquitin-activating enzyme; protein level decreases in stationary phase cultures	1.5583455	1.76988E-09
MRD1	Ortholog(s) have rRNA primary transcript binding activity	1.558029	0.000110658
QCR8	Putative ubiquinol cytochrome c reductase; macrophage and pseudohyphal-induced protein; colony morphology-related gene regulation by Ssn6; Hap43-repressed; Spider biofilm repressed	1.5579285	1.30235E-13
SCD6	Ortholog(s) have eukaryotic initiation factor 4G binding, mRNA binding activity, role in P-body assembly, negative regulation of translational initiation, stress granule assembly and P-body, cytoplasmic stress granule localization	1.5553517	2.54318E-10
CAB3	Putative phosphopantothenoylcysteine decarboxylase, binds to protein phosphatase Ppz1p and regulates its activity	1.5552654	0.000439028
orf19.4893	Ortholog of *C. dubliniensis CD36* : Cd36_09630, *C. parapsilosis CDC317* : CPAR2_805080, *C. auris B8441* : B9J08_004031 and *Candida tenuis NRRL Y-1498* : CANTEDRAFT_136811	1.5514099	0.004117599
CRL1	Predicted GTPase of RHO family; CAAX motif geranylgeranylated; expression in S. cerevisiae causes dominant-negative inhibition of pheromone response	1.5511582	1.85755E-13
orf19.3778	Protein with a predicted role in ribosome biogenesis; mutation confers hypersensitivity to 5-fluorocytosine (5-FC), 5-fluorouracil (5-FU); repressed in core stress response; repressed by prostaglandins; Hap43-induced	1.5493686	7.78403E-11
CAP1	AP-1 bZIP transcription factor; apoptotic, oxidative stress response/resistance, multidrug resistance; nuclear in oxidative stress; complements S. cerevisiae yap1 mutant; oralpharyngeal candidasis-, human neutrophil, Spider biofilm induced	1.5480336	3.33073E-20
KTI12	Protein similar to S. cerevisiae Kti12p, which associates with Elongator complex; has a role in resistance to killer toxin; predicted Kex2p substrate; Hap43p-induced gene	1.5472455	0.000448331
orf19.1388	Putative 66S pre-ribosomal particle component; Hap43-induced; F-12/CO2 early biofilm induced	1.5454727	4.96242E-08
orf19.3836	Ortholog(s) have protein-lysine N-methyltransferase activity and role in peptidyl-lysine trimethylation	1.5439886	2.41863E-07
ZUO1	Ortholog of S. cerevisiae Zuo1; a cytosolic ribosome-associated chaperone; likely to be essential for growth, based on an insertional mutagenesis strategy; Spider biofilm repressed	1.5434267	1.93976E-22
PRT1	Putative translation initiation factor eIF3; mutation confers hypersensitivity to roridin A, verrucarin A; genes encoding ribosomal subunits, translation factors, tRNA synthetases are downregulated upon phagocytosis by murine macrophages	1.5432941	3.1299E-08
orf19.2826	Ortholog of *C. dubliniensis CD36* : Cd36_27680, *C. parapsilosis CDC317* : CPAR2_801140, *C. auris B8441* : B9J08_004087 and *Candida tenuis NRRL Y-1498* : CANTEDRAFT_129982	1.5405551	2.27778E-05

orf19.3991	Ortholog(s) have acylglycerol lipase activity, role in cellular lipid metabolic process and cytoplasm, nucleus localization	1.5392545	1.93724E-06
EIP1	Probable securin that interacts with and regulates cohesin protease (separase) Esp1p to ensure chromatid separation during anaphase; Spider biofilm repressed	1.5355946	2.31501E-07
orf19.3461	Protein of unknown function; oxidative stress-induced via Cap1; induced by alpha pheromone in SpiderM medium	1.5355072	5.59465E-10
orf19.813	Protein of unknown function; mutants are viable; Hap43-induced gene; oxidative stress-induced via Cap1; rat catheter and Spider biofilm induced	1.5349827	0.000221777
orf19.5856	Membrane-localized protein of unknown function	1.5339087	7.47314E-08
MRP2	Protein similar to S. cerevisiae Mrp2p, which is a component of the small subunit of the mitochondrial ribosome; transposon mutation affects filamentous growth	1.5331168	1.71391E-19
RRB1	Ortholog(s) have unfolded protein binding activity, role in ribosome biogenesis and nucleolus localization	1.5314675	3.40458E-08
orf19.7489.3	Ortholog(s) have 2 iron, 2 sulfur cluster binding, iron-sulfur cluster binding activity	1.5308044	0.00602415
HFI1	Ortholog(s) have transcription coactivator activity, transcription coregulator activity and role in chromatin organization, histone acetylation, transcription by RNA polymerase II	1.5306066	0.000673006
orf19.7011	Ortholog(s) have role in maturation of SSU-rRNA from tricistronic rRNA transcript (SSU-rRNA, 5.8S rRNA, LSU-rRNA) and nucleus, preribosome, small subunit precursor localization	1.5302719	0.002238113
orf19.4239	Planktonic growth-induced gene	1.5291093	0.002547424
orf19.5269	Ortholog of *C. dubliniensis CD36* : Cd36_11210, *C. parapsilosis CDC317* : CPAR2_805180, *C. auris B8441* : B9J08_002727 and *Candida tenuis NRRL Y-1498* : CANTEDRAFT_116279	1.5262401	0.001952955
orf19.3858	Protein of unknown function; rat catheter and Spider biofilm induced	1.5227734	0.004134147
MUB1	Predicted protein required for ubiquitination; role in meiosis, regulation of cell budding in S. cereviae; Spider biofilm induced	1.5213671	0.002433903
SPT4	Ortholog(s) have RNA polymerase II complex binding, protein-containing complex binding, rDNA binding, single-stranded RNA binding activity	1.5207492	8.62199E-12
orf19.6665	Ortholog(s) have structural constituent of nuclear pore activity	1.5204617	4.90925E-09
TRM9	Putative tRNA methyltransferase; repressed during the mating process	1.5201678	7.83735E-12
RGA2	Putative GTPase-activating protein (GAP) for Rho-type GTPase Cdc42; involved in cell signaling pathways controlling cell polarity; induced by low-level peroxide stress; flow model biofilm induced	1.5197448	0.001321552

MSB1	Putative regulator of transcription; expression in S. cerevisiae flo8 and flo11 mutants suggests a role in regulation of adhesion factors	1.5178483	2.80405E-05
PRR1	Putative serine/threonine protein kinase; Hog1p-induced	1.5178483	0.001939152
FRS1	Phenylalanyl-tRNA synthetase; possible role in early cell wall biosynthesis; downregulated by phagocytosis by macrophages; possibly essential gene, disruptants not obtained by UAU1 method; protein present in exponential and stationary phase	1.5144474	1.88067E-10
HRD3	Ortholog(s) have ubiquitin-protein transferase activity and role in negative regulation of protein autoubiquitination, retrograde protein transport, ER to cytosol, ubiquitin-dependent ERAD pathway	1.5137822	3.81869E-05
UTR1	Predicted NAD+/NADH kinase; possible role in cellular iron ion homeostasis; Spider biofilm induced	1.5132398	5.71467E-07
YND1	Ortholog(s) have ATPase, GTPase activity, adenosine-diphosphatase activity, cytidine-diphosphatase activity, guanosine-diphosphatase activity and nucleoside-diphosphatase activity	1.5120996	0.002533273
SMC6	Putative structural maintenance of chromosomes (SMC) protein; Hap43-induced; cell-cycle regulated periodic mRNA expression; S. cerevisiae ortholog not cell-cycle regulated; Spider biofilm induced	1.5119831	0.001001265
orf19.4278	Ortholog(s) have chromatin binding, methylated histone binding activity and role in negative regulation of transcription by RNA polymerase II	1.5111984	0.000447325
HMO1	HMG-box transcription factor; binds upstream of hexose and ergosterol metabolism and cell cycle genes; acts as repressor of START; activates pseudohyphal growth when expressed in S. cerevisiae; repressed in hyphae	1.5093495	4.89917E-17
orf19.5547	Protein of unknown function; Hap43-repressed gene	1.5084822	1.0872E-11
orf19.4017	Ortholog of *C. dubliniensis CD36* : Cd36_54840, *C. parapsilosis CDC317* : CPAR2_100270, *C. auris B8441* : B9J08_003974 and *Candida tenuis NRRL Y-1498* : cten_CGOB_00242	1.5081235	0.018313132
PDX1	Pyruvate dehydrogenase complex protein X; essential component of the mitochondrial pyruvate dehydrogenase complex; role in the respiratory pathway; protein present in exponential and stationary growth phase yeast; Spider biofilm repressed	1.5077165	3.81389E-15
NAM2	Mitochondrial leucyl-tRNA synthetase	1.5068832	0.004663786
KES1	Putative oxysterol binding protein family; probable peripheral membrane protein of the Golgi complex; flow model and Spider biofilm repressed	1.5053698	4.74854E-07

orf19.7341	Protein of unknown function; flow model biofilm induced; ketoconazole-repressed	1.5039835	6.2921E-05
FZO1	Mitochondrial biogenesis protein; rat catheter and Spider biofilm induced	1.5021137	0.000166803
orf19.3644	Protein of unknown function; Cyr1-repressed; rat catheter and Spider biofilm induced	1.501531	3.18789E-05
PRP3	Predicted splicing factor, component of the U4/U6-U5 snRNP complex; Hap43-induced gene; rat catheter biofilm induced	1.5013869	0.002010267
TAF3	Protein with a histone fold domain; similar to TAFII47 proteins from S. cerevisiae, Danio rerio, Drosophila melanogaster, human, and mouse	1.5000736	0.000462844
GLN4	Putative tRNA-Gln synthetase; genes encoding ribosomal subunits, translation factors, tRNA synthetases are downregulated upon phagocytosis by murine macrophage; protein present in exponential and stationary growth phase yeast cultures	1.4994095	1.45008E-07
orf19.7618	Putative nucleolar protein with a predicted role in pre-18S rRNA processing; Plc1p-regulated; Spider biofilm induced	1.4972524	7.63098E-13
orf19.2575	Putative S-adenosylmethionine-dependent methyltransferase; Hap43p-induced gene	1.4972131	3.54292E-05
CRN1	Coronin; cortical actin cytoskeletal component; predicted role in regulation of actin patch assembly; rat catheter and Spider biofilm repressed	1.4966066	4.85004E-05
LAS1	Putative bud formation and morphogenesis protein; mutation confers hypersensitivity to 5-fluorocytosine (5-FC), 5-fluorouracil (5-FU); macrophage-induced; Spider biofilm induced	1.4964095	0.00022333
orf19.5070	Similar to cell-wall mannoproteins; induced in low iron; induced in cyr1 homozygous null; regulated by osmotic and oxidative stress via Hog1; Spider biofilm induced	1.4950519	2.02104E-08
TIM50	Predicted component of the Translocase of the Inner Mitochondrial membrane (TIM23 complex); involved in mitochondrial protein import; Spider biofilm repressed	1.4940259	2.23613E-07
CTA9	Protein required for normal filamentous growth; induced during the mating process; mRNA binds She3; activates transcription in 1-hybrid assay in S. cerevisiae	1.493379	0.000253836
orf19.2051	Ortholog of *C. dubliniensis CD36* : Cd36_15670, *C. parapsilosis CDC317* : CPAR2_213100, *C. auris B8441* : B9J08_001290 and *Candida tenuis NRRL Y-1498* : CANTEDRAFT_135034	1.4933736	0.000513274
orf19.2266	Ortholog(s) have ATPase, DNA binding, nucleosome binding activity, role in sister chromatid cohesion and Isw1a complex localization	1.4919594	3.2436E-05

orf19.1998	Ortholog of *C. dubliniensis CD36* : Cd36_16140, *C. parapsilosis CDC317* : CPAR2_213870, *C. auris B8441* : B9J08_003044 and *Candida tenuis NRRL Y-1498* : CANTEDRAFT_92202	1.4911187	5.2554E-10
orf19.4830	Has domain(s) with predicted DNA binding activity, role in DNA recombination, DNA repair, DNA replication and nucleus localization	1.4901719	5.45679E-10
SVL3	Protein of unknown funtion; S. cerevisiae ortholog Svl3 plays a role in endocytosis and is localized to the bud neck; Spider biofilm induced	1.4887148	0.000118802
orf19.6832	Protein of unknown function; regulated by Sef1p-, Sfu1p-, and Hap43p	1.4878333	0.006408292
TSR1	Component of 20S pre-rRNA processing unit; repressed by prostaglandins	1.4876307	2.52114E-06
MSS1	Ortholog(s) have role in mitochondrial tRNA wobble uridine modification and mitochondrial inner membrane localization	1.4856433	0.00252462
PET112	Ortholog(s) have glutaminyl-tRNA synthase (glutamine-hydrolyzing) activity, role in glutaminyl-tRNAGln biosynthesis via transamidation, mitochondrial translation and glutamyl-tRNA(Gln) amidotransferase complex, mitochondrion localization	1.4852481	0.000641289
PCP1	Ortholog(s) have serine-type endopeptidase activity, role in regulation of mitochondrion organization, signal peptide processing and mitochondrial inner membrane localization	1.4847591	1.50569E-13
PRP2	Ortholog(s) have ATP-dependent activity, acting on RNA activity, role in generation of catalytic spliceosome for first transesterification step, snoRNA splicing and U2-type catalytic step 1 spliceosome localization	1.4821141	0.011955776
MNN1	Putative alpha-1,3-mannosyltransferase; of the mannosyltransferase complex; negatively regulated by Rim101; transcript elevated in chk1 and nik1 mutants, but not in sln1 mutant; Spider and flow model biofilm induced	1.4819294	0.000163546
BDP1	Putative RNA polymerase III transcription factor (TFIIIB) subunit; flucytosine repressed	1.4813861	0.00010763
CKB1	Regulatory subunit of protein kinase CK2 (casein kinase II), beta subunit; null mutants are hypersensitive to caspofungin and hydrogen peroxide medium	1.4804177	0.000171521
VHR1	Transcriptional activator of genes involved in biotin metabolism; required for survival and proliferation in macrophages; expression upregulated during growth in the mouse cecum; Spider biofilm induced	1.4757637	0.005623666
CSU57	Protein involved in utilization of L-sorbose as carbon source	1.4740071	0.000108011
orf19.970	Protein with a predicted role in microtubule-related processes; Spider biofilm induced	1.4734979	1.03772E-05
SEC26	Secretory vesicles coatomer complex protein	1.4732963	3.73914E-05

MED1	RNA polymerase II mediator complex subunit; RNA polymerase II transcription cofactor	1.4706539	2.66458E-06
TRM22	Has domain(s) with predicted RNA methyltransferase activity and role in RNA processing	1.469931	0.001912662
RFG1	HMG domain transcriptional repressor of filamentous growth and hyphal genes; in Tup1-dependent and -independent pathways; binds DNA; transcript not regulated by oxygen or serum; not responsible for hypoxic repression; Spider biofilm induced	1.4696532	0.0029618
ADE2	Phosphoribosylaminoimadazole carboxylase; role in adenine biosynthesis; required for normal growth and virulence in immunosuppressed mouse infection; not induced in GCN response, in contrast to S. cerevisiae ADE2; stationary phase-enriched	1.466973	4.5454E-09
COR1	Putative ubiquinol-cytochrome-c reductase; amphotericin B induced; repressed by nitric oxide; protein level decreases in stationary phase cultures; Hap43-repressed; Spider biofilm repressed	1.4663616	3.48929E-13
ATP18	F1F0 ATP synthase complex subunit; fungal-specific; gene has intron	1.4637676	3.07327E-06
orf19.6205	Ortholog of *C. dubliniensis CD36* : Cd36_06550, *C. parapsilosis CDC317* : CPAR2_209030, *C. auris B8441* : B9J08_005439 and *Candida tenuis NRRL Y-1498* : CANTEDRAFT_111035	1.4633847	7.63972E-11
SEN2	Putative tRNA splicing endonuclease subunit; mutation confers hypersensitivity to toxic ergosterol analog and to amphotericin B; 5'-UTR intron; Hap43-induced; Spider biofilm induced	1.4628478	4.36367E-05
ADR1	C2H2 transcription factor; ortholog of S. cerevisiae Adr1 but mutant phenotype suggests a different set of target genes; transposon mutation affects filamentous growth; Spider biofilm induced	1.4576582	0.023154745
orf19.6503	Ortholog(s) have structural constituent of ribosome activity, role in mitochondrial translation and mitochondrial large ribosomal subunit localization	1.4568582	6.94894E-10
NRD1	Putative RNA-binding protein; transcript is upregulated in an RHE model of oral candidiasis	1.4549784	1.97303E-10
GCN5	Histone acetyltransferase; required for hyphen elongation and cell wall organization; mutation confers hypersensitivity to 5-fluorouracil (5-FU)	1.4527566	0.001093881
BUD14	Putative SH3-domain-containing protein; predicted role in bud-site selection; Spider biofilm induced	1.4518305	0.012456297
orf19.6628	Protein of unknown function; rat catheter biofilm repressed	1.4499081	0.00068408
MTG2	Putative Obg family GTPase member; peripheral protein of the mitochondrial inner membrane; associates with the large	1.4489318	0.002082275

	ribosomal subunit; required for mitochondrial translation; rat catheter biofilm repressed		
CCT2	Chaperonin of the cytosolic TCP1 ring complex; protein present in exponential and stationary-phase yeast cells, but higher amounts in stationary phase; GlcNAc-induced protein	1.4476731	6.62289E-13
SEC1	Ortholog(s) have SNARE binding activity, role in exocytosis, positive regulation of vesicle fusion, vesicle docking involved in exocytosis and cellular bud neck, cellular bud tip, plasma membrane, prospore membrane localization	1.4438965	5.94703E-11
orf19.5569	Ortholog(s) have role in establishment of mitotic sister chromatid cohesion, maintenance of rDNA, mitotic sister chromatid segregation and chromosome, telomeric region, nuclear envelope localization	1.4412717	3.43809E-05
COX12	Ortholog(s) have cytochrome-c oxidase activity, role in mitochondrial cytochrome c oxidase assembly and mitochondrial intermembrane space, mitochondrial respiratory chain complex IV localization	1.4388867	1.5676E-13
orf19.6705	Putative guanyl nucleotide exchange factor with Sec7 domain; required for normal filamentous growth; regulated by yeast-hyphal switch; filament induced; regulated by Nrg1, Tup1, Mob2, Hap43; mRNA binds She3; Spider biofilm induced	1.4385534	0.00285037
orf19.3517	Ortholog(s) have role in positive regulation of cellular response to hypoxia, regulation of protein glycosylation, sterol regulatory element binding protein cleavage	1.4382725	8.87227E-06
TIF3	Putative translation initiation factor; genes encoding ribosomal subunits, translation factors, and tRNA synthetases are downregulated upon phagocytosis by murine macrophage	1.4382431	5.24072E-08
VPS4	AAA-ATPase involved in transport from MVB to the vacuole and ESCRT-III complex disassembly; mutation decreases SAP secretion and virulence in murine intravenous infection; regulated by Gcn2p, Gcn4p; required for normal Rim8p processing	1.4374296	1.82678E-08
orf19.3115	Protein of unknown function; rat catheter and Spider biofilm induced	1.4365916	0.000839815
AES4	Protein of unknown function; Hap43-repressed gene	1.4349941	0.008155111
orf19.475	Putative rRNA processing protein; rat catheter biofilm induced	1.4349243	1.69327E-06
orf19.1830	Protein of unknown function; Hap43-induced; rat catheter and Spider biofilm induced	1.4344028	0.008013824
orf19.6923	Ortholog(s) have chromatin binding activity, role in RNA polymerase II preinitiation complex assembly, transcription by RNA polymerase II and transcription factor TFIID complex localization	1.4343649	3.44609E-05
CHS8	Chitin synthase required for synthesis of long-chitin fibrils; nonessential; 8 or 9 membrane spanning regions; mRNA	1.4338483	3.69201E-07

	present in yeast and hyphae; induced during cell wall regeneration; flow model biofilm repressed		
orf19.284	Ortholog of *C. dubliniensis CD36* : Cd36_82890, *Debaryomyces hansenii CBS767* : DEHA2G01034g, *Pichia stipitis Pignal* : PICST_32242 and *Candida tropicalis NEW ASSEMBLY* : CTRG1_02580	1.4294922	7.00405E-16
orf19.292	Ortholog of *C. dubliniensis CD36* : Cd36_82960, *C. parapsilosis CDC317* : CPAR2_102390, *C. auris B8441* : B9J08_001199, *Candida tenuis NRRL Y-1498* : CANTEDRAFT_133198 and *Candida albicans WO-1* : CAWG_02618	1.4294922	7.00405E-16
orf19.3349	Putative RNA polymerase II subunit B150; heterozygous null mutant exhibits resistance to parnafungin in the C. albicans fitness test	1.4281511	0.000143623
SCJ1	Ortholog(s) have chaperone binding activity, role in protein folding in endoplasmic reticulum, response to unfolded protein, ubiquitin-dependent ERAD pathway and endoplasmic reticulum lumen localization	1.427809	4.94384E-05
GCD6	Ortholog of S. cerevisiae Gcd6; catalytic epsilon subunit of the translation initiation factor eIF2B; genes encoding translation factors are repressed by phagocytosis by murine macrophages	1.4269818	0.000479375
ZCF29	Zn(II)2Cys6 transcription factor; mutants sensitive to caffeine, menadione, beauvericin;resistant to fenpropimorph; Hap43-repressed; mutants have decreased CFU in mouse kidneys and defects in filamentous growth; Spider biofilm induced	1.426554	4.95028E-05
orf19.6244	Ortholog of *C. dubliniensis CD36* : Cd36_06250, *C. parapsilosis CDC317* : CPAR2_206820, *C. auris B8441* : B9J08_003766 and *Candida tenuis NRRL Y-1498* : CANTEDRAFT_125137	1.4261862	0.006747665
orf19.6923.1	Ortholog of *C. parapsilosis CDC317* : CPAR2_502850, *Candida tenuis NRRL Y-1498* : CANTEDRAFT_135869, *Debaryomyces hansenii CBS767* : DEHA2F18260g and *Pichia stipitis Pignal* : psti_CGOB_00119	1.4254101	0.000581645
NMD2	Ortholog(s) have role in DNA recombination, nuclear-transcribed mRNA catabolic process, 3'-5' exonucleolytic nonsense-mediated decay, nuclear-transcribed mRNA catabolic process, nonsense-mediated decay and cytoplasm, polysome localization	1.419714	0.022982756
SXM1	Ortholog(s) have role in mRNA export from nucleus, protein import into nucleus and cytoplasm, nucleus localization	1.418646	1.84958E-06
CSF1	Ortholog(s) have role in fermentation, protein maturation	1.4159042	0.007077578
GCD7	Putative translation initiator; downregulated in the presence of human whole blood or polymorphonuclear (PMN) cells	1.4154568	2.36686E-06

orf19.6658	Stationary phase enriched protein; predicted ORF from Assembly 19; removed from Assembly 20; subsequently reinstated in Assembly 21 based on comparative genome analysis	1.4126936	2.18282E-08
ESC4	Protein similar to S. cerevisiae Esc4; a protein that represses transposition; transposon mutation affects filamentation; rat catheter biofilm repressed	1.4123491	0.023828106
orf19.4959	Protein of unknown function; Spider biofilm repressed	1.4113756	4.70906E-08
KAR4	Transcription factor; required for gene regulation in response to pheromones; ortholog of S. cerevisiae Kar4; role in karyogamy; opaque-specific, a-specific; induced by alpha factor	1.408218	3.55672E-12
TOD6	Protein with a predicted role in telomeric gene silencing and filamentation; repressed by high-level peroxide stress; Spider biofilm induced	1.4073073	2.21806E-05
SAM2	S-adenosylmethionine synthetase; localizes to surface of hyphae, not yeast cells; alkaline, Hog1-induced; farnesol-downregulated; F-12/CO2 early biofilm induced; Spider biofilm repressed	1.4062822	3.60482E-18
ADE8	Putative phosphoribosylglycinamide formyl-transferase, enzyme of amino acid biosynthesis pathway; upregulated in biofilm; S. cerevisiae ortholog is Gcn4p regulated; protein enriched in stationary phase yeast-form cultures	1.404559	2.90216E-11
PUS1	Putative pseudouridine synthase; predicted role in snRNA pseudouridine synthesis, tRNA pseudouridine synthesis; Spider biofilm induced	1.4029647	0.00213945
IFM1	Putative mitochondrial translation initiation factor; transcript regulated by Nrg1, Mig1, and Tup1	1.3999936	0.015199044
RPS5	Ribosomal protein S5; macrophage/pseudohyphal-induced after 16 h; downregulated upon phagocytosis by murine macrophage; Hap43-induced; Spider biofilm repressed	1.3991846	4.77713E-18
SPT20	Putative transcription factor; downregulated upon adherence to polystyrene; flucytosine repressed	1.3983909	3.42939E-05
TGS1	Ortholog(s) have RNA methyltransferase activity and role in 7-methylguanosine cap hypermethylation, RNA methylation, meiotic cell cycle, nucleologenesis, regulation of telomere maintenance via telomerase, tRNA processing	1.3972125	0.000397309
MED21	Ortholog(s) have transcription coactivator activity, transcription corepressor activity and role in negative regulation of transcription by RNA polymerase II, positive regulation of transcription by RNA polymerase II	1.3964691	0.016450492
SCM3	Centromeric chromatin (CENP-A) chaperone, involved in nuclear DNA replication; expression downregulated in an ssr1 null mutant	1.3954133	0.001514548

DEF1	RNA polymerase II regulator; role in filamentation, epithelial cell escape, dissemination in RHE model; induced by fluconazole, high cell density; Efg1/hyphal regulated; role in adhesion, hyphal growth on solid media; Spider biofilm induced	1.3937381	4.81536E-06
orf19.6679	Has domain(s) with predicted metal ion binding activity	1.3930507	0.002029292
orf19.6186	Predicted protein of unknown function; overlaps orf19.6185	1.3902846	3.54951E-16
SLD2	Ortholog(s) have DNA replication origin binding, single-stranded DNA binding activity	1.3898087	0.000251097
SEC34	Ortholog(s) have cargo adaptor activity	1.3880379	0.000708621
ECM16	Ortholog of S. cerevisiae Ecm16, an essential DEAH-box ATP-dependent RNA helicase specific to the U3 snoRNP required for 18S rRNA synthesis; Hap43-induced; Spider biofilm induced	1.3879257	0.015124044
orf19.4441	Ortholog(s) have DNA replication origin binding, double-stranded DNA binding, single-stranded DNA binding activity	1.3873797	2.49236E-05
orf19.6008.4	Ortholog(s) have structural constituent of ribosome activity and mitochondrial large ribosomal subunit, mitochondrion localization	1.3873161	4.88747E-06
ARP1	Putative centractin; induced upon adherence to polystyrene	1.3872226	0.004392667
ARO4	3-deoxy-D-arabinoheptulosonate-7-phosphate synthase; aromatic amino acid biosynthesis; GCN-regulated; feedback-inhibited by tyrosine if produced in S. cerevisiae Aro3p and Aro4p catalyze same reaction; protein decreases in stationary phase	1.3872153	4.50254E-16
orf19.2564	Putative nucleolar protein; implicated in ribosome biogenesis; rat catheter biofilm repressed	1.3849832	0.001896939
MAS2	Putative processing peptidase, catalytic (alpha) subunit; protein level decreases in stationary phase cultures	1.3845257	3.11797E-05
ALS5	ALS family adhesin; highly variable; expression in S. cerevisiae causes adhesion to human epithelium, endothelium or ECM, endothelial invasiveness by endocytosis and, at high abundance, ECM-induced aggregation; can form amyloid fibrils	1.3834477	0.018909247
orf19.2325	Ortholog(s) have RNA polymerase III general transcription initiation factor activity and RNA polymerase III type 1 promoter sequence-specific DNA binding	1.3833994	0.012941972
CTA1	Protein similar to S. cerevisiae Mos10p, which affects S. cerevisiae filamentous growth; activates transcription in 1-hybrid assay in S. cerevisiae; protein levels increase under weak acid stress; nonessential	1.3832326	0.000539738
PTH2	Ortholog(s) have aminoacyl-tRNA hydrolase activity and role in negative regulation of proteasomal ubiquitin-dependent protein catabolic process	1.383107	0.01305417

TFA1	Protein with polyglutamate motifs and abundant Ser/Thr residues; described as a subunit of TFIIE, which is a basal transcription initiation factor of RNA Polymerase II; possibly an essential gene, disruptants not obtained by UAU1 method	1.3819008	2.04439E-05
orf19.952	Has domain(s) with predicted DNA binding activity	1.3806279	0.002155599
BUD22	Protein with a predicted role in 18S rRNA maturation and small ribosomal subunit biogenesis; repressed in core stress response; repressed by prostaglandins	1.3791487	5.40336E-05
TFG2	Ortholog(s) have RNA polymerase II complex binding activity	1.3787477	1.81686E-08
SEC7	Putative guanine nucleotide exchange factor (GEF); mutation confers hypersensitivity to Brefeldin A; caspofungin repressed	1.3724179	0.000552612
orf19.4092	Trimeric GatFAB AmidoTransferase(AdT) complex subunit; involved in the formation of Q-tRNAQ; repressed by fluphenazine treatment or in an azole-resistant strain that overexpresses CDR1; Spider biofilm induced	1.3721777	1.98005E-08
TOP3	Ortholog(s) have DNA topoisomerase activity, DNA topoisomerase type I (single strand cut, ATP-independent) activity	1.3663918	0.002689217
orf19.6754	Protein of unknown function; Spider biofilm induced	1.3654104	8.99668E-09
NAN1	Putative U3 snoRNP protein; Hap43p-induced gene; physically interacts with TAP-tagged Nop1p	1.3649676	1.2841E-05
ARC1	Putative G4 nucleic acid binding protein; macrophage/pseudohyphal-repressed; protein enriched in stationary phase yeast-form cultures; Spider biofilm repressed	1.3639141	4.199E-10
orf19.7085	Protein of unknown function; induced in core stress response; induced by cadmium stress via Hog1; oxidative stress-induced via Cap1; induced by Mnl1 under weak acid stress; macrophage-repressed; rat catheter and Spider biofilm induced	1.3633099	2.80457E-08
SDC1	Putative COMPASS/SET1C histone methyltransferase complex subunit	1.3630985	0.00978654
NOT3	Transcriptional regulator; not required for buccal epithelial cell adherence or virulence in mouse systemic infection; null mutant colonies exhibit slightly decreased filamentation ratio; required for yeast adherence to silicone substrate	1.3630254	4.65186E-06
RPN2	Putative 26S proteasome subunit; transcript regulated by Mig1; caspofungin repressed; regulated by Gcn2 and Gcn4; gene used for strain identification by multilocus sequence typing	1.3625701	2.03769E-05
orf19.4148	Ortholog of *C. dubliniensis CD36* : Cd36_50820, *C. parapsilosis CDC317* : CPAR2_501670, *C. auris B8441* : B9J08_002922 and *Candida tenuis NRRL Y-1498* : CANTEDRAFT_115294	1.3621671	0.00126889
SER2	Ortholog(s) have phosphoserine phosphatase activity and role in L-serine biosynthetic process	1.3575215	5.20854E-07

orf19.1393	Ortholog(s) have NAD transmembrane transporter activity, pyruvate secondary active transmembrane transporter activity, role in NAD transmembrane transport, mitochondrial pyruvate transmembrane transport and mitochondrion localization	1.3570306	5.44151E-06
RTG1	RNA polymerase II transcription factor involved in regulation of galactose catabolism genes; as result of transcriptional rewiring, performs role analogous to Gal4p in S. cerevisiae; involved in regulation of sphingolipid homeostasis	1.3531408	2.95667E-06
TRI1	Has domain(s) with predicted DNA binding activity	1.3527564	0.003139977
orf19.5201.1	Has domain(s) with predicted ATPase inhibitor activity, role in negative regulation of ATPase activity, negative regulation of nucleotide metabolic process and mitochondrion localization	1.3506001	3.73598E-08
SIN3	Protein similar to S. cerevisiae Sin3p (transcriptional corepressor involved in histone deacetylase recruitment); has paired amphipathic helix PAH1 domain; interacts with ScOpi1p, not CaOpi1p; transposon mutation affects filamentous growth	1.3496641	0.000671424
orf19.3481	Putative mitochondrial ATP-dependent RNA helicase of the DEAD-box family, transcription is activated in the presence of elevated CO2	1.3492762	1.42954E-06
VIP1	Predicted inositol polyphosphate kinase, involved in autophagy, energy metabolism, virulence	1.348747	1.72045E-08
SMY2	Ortholog(s) have role in endoplasmic reticulum to Golgi vesicle-mediated transport and endoplasmic reticulum membrane, extrinsic component of membrane localization	1.347146	5.26705E-10
ACE2	Transcription factor; similar to S. cerevisiae Ace2 and Swi5; regulates morphogenesis, cell separation, adherence, virulence in a mice; mutant is hyperfilamentous; rat catheter and Spider biofilm induced	1.3452786	0.000904103
RTA2	Flippase involved in sphingolipid long chain base release; mediates calcineurin-dependent ER stress response and resistance to azoles; Plc1p, Ca2+, calcineurin-regulated	1.3449681	2.18879E-14
orf19.3921	Ortholog of *S. cerevisiae* : FSH3, *C. glabrata CBS138* : CAGL0L11044g, *C. dubliniensis CD36* : Cd36_54010, *C. parapsilosis CDC317* : CPAR2_100920 and *C. auris B8441* : B9J08_004360	1.3443395	3.47802E-11
orf19.843	Putative DNA repair exonuclease; fungal-specific	1.3441934	0.002569669
PRP42	Putative component of the U1 snRNP, involved in splicing; ortholog of S. cerevisiae PRP42	1.3398835	0.001312546
RIM8	Beta-arrestin-like protein; involved in pH response; required for pathogenesis, activation of Rim101 and alkaline pH-induced hyphal growth; colony morphology-related gene regulation by Ssn6p negative feedback regulation target	1.3386145	5.60767E-06

KCS1	Predicted inositol polyphosphate kinase; Spider biofilm induced	1.3359493	0.003468426
PET127	Protein with a predicted role in 5'-end processing of mitochondrial RNAs; ortholog of S. cerevisiae Pet127; Hap43-induced; rat catheter and Spider biofilm induced	1.3341883	0.009615581
GUS1	Putative glutamine-tRNA ligase; stationary phase enriched protein; Spider biofilm repressed	1.333743	1.06182E-09
KAP114	Ortholog(s) have role in NLS-bearing protein import into nucleus, protein import into nucleus and cytoplasm, nucleus localization	1.3301261	9.12404E-06
CWT1	Zn2Cys6 transcription factor involved in negative regulation of nitrosative stress response; regulates formation of mating projections and same-sex mating; mutant has cell wall defects; transcription increased at stationary phase	1.3283696	3.2859E-05
orf19.4906	Putative adhesin-like protein; positively regulated by Tbf1; Spider biofilm induced	1.3279375	4.0218E-07
IMP4	Putative SSU processome component; Hap43-induced; repressed by prostaglandins; Spider biofilm induce	1.3276002	1.5885E-07
orf19.6907	Ortholog(s) have DNA binding activity, role in regulation of DNA damage checkpoint and cytoplasm, nuclear periphery localization	1.3270383	0.010442403
orf19.4150	Putative glutaredoxin; induced by nitric oxide; Spider biofilm induced	1.3256754	1.41414E-06
VPS72	Ortholog(s) have histone binding activity, role in chromatin remodeling, histone exchange and Swr1 complex localization	1.3255913	0.001515556
ORC1	Putative origin recognition complex large subunit; essential for viability; similar to S. cerevisiae Orc1p origin recognition complex subunit	1.3255262	0.000668622
TAC1	Zn(2)-Cys(6) transcriptional activator of drug-responsive genes (CDR1 and CDR2); binds DRE element; gene in zinc cluster region near MTL locus; resequencing indicates that TAC1 spans orf19.3188 and orf19.3189; Spider biofilm induced	1.324177	0.001287232
BFA1	Ortholog(s) have GDP-dissociation inhibitor activity, GTPase activator activity, role in mitotic spindle orientation checkpoint signaling, negative regulation of exit from mitosis and Bfa1-Bub2 complex, spindle pole body localization	1.3234827	0.020047389
FUN30	Ortholog(s) have ATP-dependent activity, acting on DNA, DNA binding, chromatin binding activity	1.3232664	0.003673294
PUT3	Zn(II)2Cys6 transcription factor; has similarity to S. cerevisiae Put3, a transcription factor involved in the regulation of proline utilization genes	1.3202496	0.000653461
ALG1	Protein similar to S. cerevisiae Alg1p, a mannosyltransferase involved in N-linked protein glycosylation; likely to be essential for growth, based on an insertional mutagenesis strategy	1.3200654	0.010164719

DIS3	Ortholog(s) have 3'-5'-exoribonuclease activity, endoribonuclease activity, exoribonuclease activity, ribonuclease activity, tRNA binding activity	1.3157759	0.001468603
WSS1	Putative metalloprotease involved in repair of DNA-protein crosslinks; interacts with SUMO (Smt3p) and Cdc48p	1.3144457	4.84946E-07
MED4	RNA polymerase II core mediator complex subunit	1.3139875	0.001679441
NFU1	Protein with a predicted role in mitochondrial iron metabolism; Hap43-repressed; expression upregulated during growth in the mouse cecum; Spider biofilm induced	1.3121807	1.00852E-09
orf19.7662	Ortholog(s) have RNA polymerase II complex binding activity, role in DNA damage response, detection of DNA damage, mRNA 3'-end processing, negative regulation of transposition, RNA-mediated and site of double-strand break localization	1.3117599	1.93277E-08
GSC1	Essential beta-1,3-glucan synthase subunit; gsc1 allele determines resistance/sensitivity to echinocandins; 16 predicted membrane-spanning regions; mRNA abundance declines after yeast-to-hypha transition; Spider biofilm induced	1.311217	2.67089E-11
PGA52	GPI-anchored cell surface protein of unknown function; Hap43p-repressed gene; fluconazole-induced; possibly an essential gene, disruptants not obtained by UAU1 method	1.3103796	8.43377E-19
DUS4	Ortholog(s) have tRNA dihydrouridine synthase activity and role in tRNA modification	1.3095823	5.91471E-07
orf19.4779	Putative transporter; slightly similar to the Sit1p siderophore transporter; Gcn4p-regulated; fungal-specific; induced by Mnl1p under weak acid stress	1.3059673	9.40062E-09
NUO4	Subunit of mitochondrial respiratory chain complex I; Hap43-repressed gene; repressed by nitric oxide	1.3052281	1.22346E-10
orf19.5019	Ortholog of *C. dubliniensis CD36* : Cd36_12800, *C. parapsilosis CDC317* : CPAR2_203460, *C. auris B8441* : B9J08_004645 and *Candida tenuis NRRL Y-1498* : CANTEDRAFT_94106	1.304984	4.89764E-05
NOP12	Ortholog(s) have rRNA binding activity, role in maturation of LSU-rRNA from tricistronic rRNA transcript (SSU-rRNA, 5.8S rRNA, LSU-rRNA) and nucleolus, preribosome localization	1.3039423	0.000943856
orf19.2095	Ortholog(s) have role in negative regulation of transcription from RNA polymerase II promoter in response to iron and cytosol localization	1.3038417	7.3719E-07
orf19.7506	Ortholog(s) have ATPase, DNA binding, nucleosome binding activity, role in chromatin remodeling and Isw1b complex localization	1.3026191	0.01850794
RPP2B	Conserved acidic ribosomal protein; possibly involved in regulation of translation elongation; interacts with Rpp1A; 1 of 4 similar C. albicans proteins (Rpp1A, Rpp1B, Rpp2A, Rpp2B); macrophage/pseudohyphal-induced; Spider biofilm repressed	1.3023659	1.68181E-12

RIB2	Ortholog(s) have 2,5-diamino-6-ribitylamino-4(3H)-pyrimidinone 5'-phosphate deaminase activity, pseudouridine synthase activity and role in riboflavin biosynthetic process, tRNA pseudouridine synthesis	1.3021123	6.04798E-06
orf19.687	Ortholog of *C. dubliniensis CD36* : Cd36_62090, *C. parapsilosis CDC317* : CPAR2_602150, *C. auris B8441* : B9J08_005503 and *Candida tenuis NRRL Y-1498* : CANTEDRAFT_112751	1.3015014	0.0005328
SET5	Ortholog(s) have S-adenosylmethionine-dependent methyltransferase activity and role in gene silencing, histone lysine methylation, telomere maintenance	1.3014475	0.00067121
orf19.862	Protein of unknown function; Spider biofilm induced	1.3006978	0.001057957
SAS10	Putative U3-containing small subunit processome complex subunit; Hap43p-induced gene; mutation confers resistance to 5-fluorocytosine (5-FC); repressed upon high-level peroxide stress	1.2990459	0.004255115
RBD1	Rhomboid-like protein; similar to putative intramembrane serine proteases; role in filamentous growth; Gcn4-regulated; caspofungin repressed; Spider biofilm induced	1.2986583	0.000210667
CTA24	Putative transcription factor; positive regulator of gene expression; Efg1-repressed; member of a family of telomere-proximal genes; transcript upregulated in RHE model of oral candidiasis	1.2980043	7.1564E-13
PSE1	Ortholog(s) have nuclear localization sequence binding activity	1.295248	1.64767E-05
orf19.7091	Protein of unknown function; induced by nitric oxide; Spider biofilm repressed	1.2924105	1.7185E-08
MNN14	Predicted alpha-1,3-mannosyltransferase activity with a role in protein glycosylation; Hap43-repressed; Spider biofilm induced	1.2919155	0.017869678
orf19.6137	Has domain(s) with predicted ATP binding, nucleic acid binding activity	1.2915966	0.001698044
TRM7	Ortholog(s) have tRNA (cytosine-2'-O-)-methyltransferase activity, tRNA (guanosine-2'-O-)-methyltransferase activity, tRNA 2'-O-methyltransferase activity	1.2906534	0.000646471
NUO2	NADH-ubiquinone oxidoreductase subunit; Hap43p-repressed gene; repressed by nitric oxide; identified in detergent-resistant membrane fraction (possible lipid raft component)	1.2902049	7.40055E-13
orf19.332.1	Ortholog of *C. dubliniensis CD36* : Cd36_83290, *C. parapsilosis CDC317* : CPAR2_102640, *Candida tenuis NRRL Y-1498* : CANTEDRAFT_98613 and *Debaryomyces hansenii CBS767* : DEHA2D07282g	1.2897897	0.00032397
orf19.1195	Ortholog(s) have metalloendopeptidase activity, role in cellular iron ion homeostasis, protein processing involved in protein	1.2892659	0.017075504

	targeting to mitochondrion, protein stabilization and mitochondrial matrix localization		
orf19.529	Ortholog of *C. dubliniensis CD36* : Cd36_29750, *C. parapsilosis CDC317* : CPAR2_205260, *C. auris B8441* : B9J08_005543 and *Candida tenuis NRRL Y-1498* : CANTEDRAFT_91782	1.2879222	0.0020393
HRP1	Putative cleavage factor I subunit; required for the cleavage and polyadenylation of pre-mRNA 3' ends; Spider biofilm repressed	1.2842409	2.98087E-14
PKC1	Protein kinase C; functional homolog of S. cerevisiae Pkc1p; mutant has abnormal yeast-form cell morphology and increased cell lysis; activated by phosphatidylserine; target of antifungal, cercosporamide; R400P mutant is activated	1.2840752	0.000247964
orf19.3793	Protein of unknown function; mRNA binds She3; regulated by Nrg1; upregulated in a cyr1 or ras1 mutant	1.2840295	8.35485E-05
orf19.7116	Protein of unknown function; transcript induced in RHE model of oral candidiasis; Spider biofilm repressed	1.2810982	2.48536E-07
SUP35	Translation factor eRF3; shows prion-like aggregation in some, not all, studies; partially complements S. cerevisiae sup35 mutant translation defect; species barrier with S. cerevisiae Sup35p prion; gene not regulated by yeast-hyphal switch	1.2785834	4.24332E-09
orf19.7406	Ortholog of *C. dubliniensis CD36* : Cd36_86220, *C. parapsilosis CDC317* : CPAR2_404490, *C. auris B8441* : B9J08_000040 and *Candida tenuis NRRL Y-1498* : CANTEDRAFT_94473	1.2784096	0.001016412
orf19.6413	Protein of unknown function; rat catheter biofilm induced	1.2776296	0.000777754
CCL1	Ortholog(s) have cyclin-dependent protein kinase activating kinase regulator activity, cyclin-dependent protein serine/threonine kinase activator activity, cyclin-dependent protein serine/threonine kinase regulator activity	1.2770492	0.012694022
orf19.3161	Ortholog(s) have ATPase activator activity, RNA binding activity	1.2764636	0.000260456
NAB3	Putative nuclear polyadenylated RNA-binding protein; flucytosine repressed	1.2763152	4.14931E-09
orf19.2418	Ortholog of *C. dubliniensis CD36* : Cd36_28190, *C. parapsilosis CDC317* : CPAR2_802530, *Candida tenuis NRRL Y-1498* : CANTEDRAFT_109615 and *Debaryomyces hansenii CBS767* : DEHA2A12650g	1.2757251	0.000412521
orf19.5412	Ortholog of *S. cerevisiae* : ECM9, *C. glabrata CBS138* : CAGL0L02255g, *C. dubliniensis CD36* : Cd36_80470, *C. parapsilosis CDC317* : CPAR2_503390 and *C. auris B8441* : B9J08_003812	1.2753486	0.00654434
CDC37	Chaperone for Crk1p; interacts with Crk1p kinase domain and with Sti1p; putative phosphorylation site at Ser14; functional homolog of S. cerevisiae Cdc37p; likely to be essential for growth; regulated by Gcn2p and Gcn4p	1.2751602	0.001079536
RPL33A	Ribosomal 60S subunit protein; Spider biofilm repressed	1.2751581	1.95143E-09

MKT1	Ortholog(s) have mRNA 3'-UTR binding activity and role in biological process involved in interspecies interaction between organisms, cellular response to DNA damage stimulus	1.2743624	0.009327503
orf19.4728	Ortholog(s) have role in histone deacetylation and Set3 complex localization	1.2723003	0.000777016
BUR2	Protein with similarity to S. cerevisiae Bur2p, contains a cyclin domain; not required for wild-type hyphal growth, adherence to buccal epithelial cells, or virulence in mouse systemic infection	1.2710149	0.002803255
orf19.2995	Protein of unknown function; induced by nitric oxide; rat catheter and Spider biofilm induced	1.2691946	0.000643317
orf19.3264.1	Protein of unknown function; Spider biofilm repressed	1.2676339	0.01310048
orf19.7648	Has domain(s) with predicted antiporter activity, xenobiotic transmembrane transporter activity, role in xenobiotic transmembrane transport and membrane localization	1.2669791	4.95417E-08
ZCF10	Putative transcription factor with zinc cluster DNA-binding motif	1.2667018	0.024051183
NAM7	Putative role in nonsense-mediated mRNA decay; similar to S. cerevisiae Nam7p; gene induced by ciclopirox olamine treatment	1.2638324	8.67348E-07
SFL2	Transcription factor involved in regulation of morphogenesis; regulates transcription in response to carbon dioxide levels; required for filamentous growth, for virulence in RHE model but not in mice; Spider biofilm induced	1.2624059	0.000230184
orf19.5541	Protein with similarity to S. pombe Nrd1p; transcription induced upon induction of hyphal growth; regulated by Cph1p, Efg1p, Cph2p; low-level expression; alkaline upregulated; fungal-specific (no human or murine homolog)	1.2613338	1.94549E-06
OYE22	Putative NADPH dehydrogenase; rat catheter biofilm induced	1.2604917	9.01711E-07
CUE1	Ortholog(s) have ubiquitin binding, ubiquitin-protein transferase activator activity and role in establishment of protein localization to endoplasmic reticulum membrane, ubiquitin-dependent ERAD pathway	1.2601966	1.03983E-08
FGR32	Protein similar to S. cerevisiae Swa2p; induced upon adherence to polystyrene; transposon mutation affects filamentous growth; Hap43p-repressed gene	1.2601837	5.14909E-06
RAD4	Protein similar to S. cerevisiae Rad4p; down-regulation associated with azole resistance	1.2601104	0.000592825
orf19.6054	Protein with a Bul1 domain; binds the ubiquitin ligase Rsp5 and is involved in intracellular trafficking of a general amino acid permease Gap1; repressed in an azole-resistant strain overexpressing MDR1; Spider biofilm induced	1.2598671	0.005796617
FRS2	Putative tRNA-Phe synthetase; downregulated upon phagocytosis by murine macrophage; protein present in	1.2590217	7.89331E-11

	exponential and stationary growth phase yeast cultures; Spider biofilm repressed		
orf19.3469	S. cerevisiae ortholog Stb1 has a role in regulation of MBF-specific transcription at Start; induced in a cyr1 null mutant; Spider biofilm induced	1.2583822	2.92491E-05
PAB1	Putative poly(A)-binding protein; regulated by Gcn4p; induced in response to amino acid starvation (3-AT treatment); protein present in exponential and stationary growth phase yeast cultures	1.2565584	2.94431E-13
SEN1	Putative helicase; repressed by prostaglandins	1.2560402	0.001842118
orf19.551	Ortholog of *C. dubliniensis CD36* : Cd36_29980, *C. parapsilosis CDC317* : CPAR2_204170, *C. auris B8441* : B9J08_004836 and *Candida tenuis NRRL Y-1498* : CANTEDRAFT_110235	1.2558588	0.000564816
LIG4	DNA ligase; mRNA detected in yeast-form and pseudohyphal cells, induced upon hyphal induction; suppresses S. cerevisiae ime1-1 mutant IME2 transcription defect; required for wild-type filamentous growth and wild-type pathogenesis	1.2540054	0.009775007
orf19.6676	Has domain(s) with predicted diphthine synthase activity, methyltransferase activity and role in metabolic process, peptidyl-diphthamide biosynthetic process from peptidyl-histidine	1.2539281	2.26035E-07
orf19.1124.2	Ortholog(s) have diphthine synthase activity and role in peptidyl-diphthamide biosynthetic process from peptidyl-histidine	1.2539281	2.26035E-07
RSN1	Putative membrane protein; induced by alpha pheromone in SpiderM medium; Hap4-induced gene; Spider biofilm induced	1.2534728	0.002420089
ISW1	Ortholog(s) have ATPase, DNA binding, nucleosome binding, rDNA binding activity	1.2519725	0.000104641
ISA1	Putative mitochondrial iron-sulfur protein; alkaline repressed; induced in high iron; regulated by Sef1, Sfu1, Hap43; Spider biofilm induced	1.2484795	4.3389E-05
orf19.6636	Protein of unknown function; rat catheter biofilm repressed	1.2467603	0.011389001
RPS27B	Putative ribosomal protein; repressed upon phagocytosis by murine macrophage; Spider biofilm repressed	1.2463475	4.51539E-05
PTK2	Putative protein kinase of polyamine import; mutation confers hypersensitivity to high concentrations of tunicamycin; YPD flow model biofilm induced; rat catheter and Spider biofilm induced	1.2463191	4.18566E-06
PGA1	Putative GPI-anchored protein; induced during cell wall regeneration; required for normal adhesion to host cells and for adherence during Sabouraud biofilm formation; Spider biofilm induced	1.2463171	1.1003E-07
GCF1	HMG box mitochondrial protein; binds to mt DNA and the HWP1 promoter; mutant phenotype and functional	1.2462083	8.16327E-07

	complementation of an S. cerevisiae abf2 mutation suggest role in mt genome replication, maintenance; flow and Spider biofilm repressed		
MDM31	Ortholog(s) have role in cellular ion homeostasis, mitochondrion inheritance, mitochondrion organization, regulation of cardiolipin metabolic process and mitochondrial inner membrane, mitochondrion localization	1.2459945	0.004725283
orf19.4820	Ortholog of *S. cerevisiae* : YKL162C, *C. dubliniensis CD36* : Cd36_09010, *C. parapsilosis CDC317* : CPAR2_214170, *C. auris B8441* : B9J08_005393 and *Candida tenuis NRRL Y-1498* : CANTEDRAFT_95066	1.2454132	0.000271249
orf19.6476	Putative protein with a predicted role in exocytic transport from the Golgi; filament induced	1.2444966	0.019200576
orf19.7527	Ortholog of *C. dubliniensis CD36* : Cd36_25130, *C. parapsilosis CDC317* : CPAR2_800100, *C. auris B8441* : B9J08_002680 and *Candida tenuis NRRL Y-1498* : CANTEDRAFT_107256	1.2423554	0.000802079
STP2	Amino-acid-regulated transcription factor; activates transcription of amino acid permease genes; activated by amino-acid-induced proteolytic processing (Ssy1, Csh3 dependent); required for alkalinization of medium; Spider biofilm induced	1.2412088	0.001762222
orf19.4207	Predicted ORF from Assembly 19; removed from Assembly 20; restored based on comparative genome analysis	1.2406204	0.004390247
orf19.4204	Ortholog(s) have structural constituent of ribosome activity and mitochondrial small ribosomal subunit localization	1.2397782	2.65917E-05
orf19.7012	Ortholog(s) have structural constituent of ribosome activity and mitochondrial small ribosomal subunit localization	1.2380342	7.79677E-10
RAX1	Protein with a predicted role in bud site selection; hypha-induced expression; Spider biofilm induced	1.2361906	5.98378E-05
orf19.425	Ortholog(s) have mitochondrial large ribosomal subunit localization	1.2352024	0.00458251
orf19.863	Ortholog(s) have structural constituent of ribosome activity and mitochondrial large ribosomal subunit localization	1.2331897	6.37674E-08
SKY1	Predicted protein serine/threonine kinase; Spider biofilm induced	1.2322621	0.021356506
RFA1	Putative DNA replication factor A; RNA abundance regulated by cell cycle, tyrosol and cell density	1.2320788	7.76924E-10
RAD27	Ortholog(s) have 5'-3' exonuclease activity, 5'-flap endonuclease activity, double-stranded DNA 5'-3' exodeoxyribonuclease activity, single-stranded DNA 5'-3' exodeoxyribonuclease activity	1.2318044	2.81891E-05
RAD50	Putative DNA double-strand break repair factor; involved in response to oxidative stress and drug resistance; flow model biofilm repressed	1.231676	0.011747801

orf19.6233	Has domain(s) with predicted oxidoreductase activity and role in metabolic process	1.2315444	9.62596E-08
RRP9	Ribosomal protein; mutation confers resistance to 5-fluorocytosine (5-FC), 5-fluorouracil (5-FU), and tubercidin (7-deazaadenosine); physically interacts with TAP-tagged Nop1; Hap43-induced; Spider biofilm induced	1.2312289	0.003294047
DCR1	Putative Dicer RNAse involved in RNA interference, similar to S. cerevisiae Rnt1p but orthologous to S. castellii Dcr1p, which is not conserved in S. cerevisiae	1.230777	0.001324883
ATS1	Protein required for modification of wobble nucleosides in tRNA; induced upon adherence to polystyrene; regulated by Sef1, Sfu1, and Hap43	1.2305514	2.78454E-05
SEC63	Endoplasmic reticulum (ER) protein-translocation complex subunit	1.2295534	0.000363068
ENT3	Putative epsin; induced during the mating process; transcript is upregulated in an RHE model of oral candidiasis	1.2292385	2.05608E-06
orf19.1576	Ortholog(s) have nuclear localization sequence binding, ubiquitin binding activity and role in response to ethanol	1.2278311	0.01498868
FUM12	Putative fumarate hydratase; enzyme of citric acid cycle; fluconazole, Efg1 repressed; induced in high iron; protein present in exponential and stationary growth phase	1.2269509	1.79152E-13
SNF5	SWI/SNF chromatin remodeling complex subunit involved in transcriptional regulation; mutants have defects in silicone adherence, biofilm formation, hyphal morphogenesis, cell wall defects; increased cell aggregation during yeast form growth	1.2255361	0.000568203
SPC34	Subunit of the Dam1 (DASH) complex, which acts in chromosome segregation by coupling kinetochores to spindle microtubules	1.2241103	0.000926421
COG5	Ortholog(s) have role in cytoplasm to vacuole transport by the Cvt pathway, intra-Golgi vesicle-mediated transport and Golgi transport complex localization	1.2225107	0.00401394
ARO1	Putative pentafunctional arom enzyme; required for aromatic amino acid biosynthesis; involved in cell wall integrity and formation of biofilm; fungal-specific (no human or murine homolog); Gcn2p-, Gcn4p-regulated	1.2222432	7.8477E-05
NNF1	Ortholog(s) have role in chromosome segregation and kinetochore, nuclear MIS12/MIND complex localization	1.2220785	0.003360705
BUB2	Ortholog(s) have GTPase activator activity	1.2215141	0.016946355
SGT1A	Putative co-chaperone protein with a predicted role in kinetochore assembly; mutation confers hypersensitivity to radicicol; sumoylation target	1.2213466	1.17985E-06
ARP7	Component of the RSC chromatin remodeling complex; putative DNA translocase; Spider biofilm repressed	1.2207085	8.09042E-07
HPC2	Ortholog(s) have DNA binding, nucleosome binding activity	1.2204297	0.026002559

SMC1	Protein similar to chromosomal ATPases; RNA abundance regulated by tyrosol and cell density; cell-cycle regulated periodic mRNA expression	1.2203121	0.025712685
ISU1	Protein with similarity to NifU; possible role in iron-sulfur cluster biogenesis; induced in low iron; rat catheter biofilm induced; rat catheter and Spider biofilm induced	1.2187327	1.35935E-09
DAM1	Essential subunit of the Dam1 (DASH) complex, which acts in chromosome segregation by coupling kinetochores to spindle microtubules	1.2183598	4.00903E-06
MNL1	Transcription factor; induces transcripts of stress response genes via SLE (STRE-like) elements; required for adaptation to weak acid stress; activates a subset of the genes that are repressed by Nrg1	1.2142526	0.003583891
orf19.2019	Ortholog(s) have structural constituent of ribosome activity, role in mitochondrial translation and mitochondrial large ribosomal subunit localization	1.2126824	1.84416E-10
LHS1	Protein similar to S. cerevisiae Hsp70p; predicted Kex2p substrate; possibly essential, disruptants not obtained by UAU1 method; flow model biofilm repressed	1.2124855	0.008466199
TFA2	TFIIE small subunit; involved in RNA polymerase II transcription initiation; Spider biofilm induced	1.2111908	0.000102254
RAD57	Putative DNA recombination and repair protein; induced by interaction with macrophage; transcript is regulated by Nrg1, Mig1, and Tup1; essential protein; S. cerevisiae ortholog is essential	1.2109782	0.003267729
orf19.6736	Protein required for mitochondrial ribosome small subunit biogenesis; role in maturation of SSU-rRNA; Spider biofilm induced	1.2100948	0.000624001
COX8	Putative cytochrome c oxidase; flucytosine induced; caspofungin repressed	1.209478	5.39117E-11
orf19.5694	Predicted ORF from Assembly 19; removed from Assembly 20; restored based on transcription data	1.2074968	2.87027E-12
PRP46	Ortholog(s) have role in mRNA splicing, via spliceosome and Prp19 complex, spliceosomal complex localization	1.2063294	1.79E-06
FPR3	Putative peptidyl-prolyl cis-trans isomerase	1.2011253	1.76704E-07
ATG13	Predicted regulatory subunit of the Atg1 signaling complex; required for vesicle formation during autophagy and the cytoplasm-to-vacuole targeting (Cvt) pathway; Spider biofilm induced	1.2008446	0.002613508
YHC1	Ortholog(s) have mRNA binding, pre-mRNA 5'-splice site binding activity and role in mRNA 5'-splice site recognition, mRNA splicing, via spliceosome, positive regulation of RNA binding	1.2007845	0.001001387

CDL1	Putative RNAse III, ortholog of S. cerevisiae RNT1; merged with orf19.3772 in Assembly 21	1.200253	0.003700014
CTK1	Putative kinase subunit of RNA polymerase II carboxy-terminal domain kinase I; possibly an essential gene, disruptants not obtained by UAU1 method	1.1998459	0.012783145
FGR16	Protein lacking an ortholog in S. cerevisiae; transposon mutation affects filamentous growth; fluconazole-downregulated	1.1997482	0.006842294
orf19.1876	Ortholog(s) have mRNA binding activity and role in mRNA splice site selection, mRNA splicing, via spliceosome, positive regulation of mRNA splicing, via spliceosome	1.1996885	1.07682E-05
orf19.5772	Ortholog(s) have role in chromatin remodeling, histone exchange and Swr1 complex, cytosol, nucleus localization	1.1979394	0.002199994
RPL12A	Ribosomal protein L12, 60S ribosomal subunit; downregulated by human whole blood or polymorphonuclear cells; genes encoding cytoplasmic ribosomal subunits are downregulated upon phagocytosis by macrophage; Tbf1p-activated; Hap43p-induced	1.1978991	2.51061E-14
FCR1	Transcription factor; repressor of fluconazole/ketoconazole/brefeldin A resistance; Tn mutation enhances filamentation; partially rescues S. cerevisiae pdr1 pdr3 fluconazole sensitivity; rat catheter biofilm induced/Spider biofilm repressed	1.1964677	7.59573E-06
orf19.2866	Has domain(s) with predicted DNA binding, nucleic acid binding activity	1.1959972	0.000155935
TIM11	Ortholog(s) have proton-transporting ATP synthase activity, rotational mechanism, structural molecule activity and role in ATP synthesis coupled proton transport, cristae formation, protein-containing complex assembly	1.1936054	6.30625E-07
orf19.3724	Ortholog(s) have small ribosomal subunit rRNA binding activity	1.1933644	0.013410745
LEU3	Zn(II)2Cys6 transcription factor; predicted regulator branched-c ofhain amino acid biosynthesis genes; alkaline induced; induced by Mnl1 under weak acid stress; required for yeast cell adherence to silicone substrate; Spider biofilm induced	1.1922681	8.30548E-05
orf19.384	Ortholog of *S. cerevisiae* : YPL247C, *C. glabrata CBS138* : CAGL0H00781g, *C. dubliniensis CD36* : Cd36_07900, *C. parapsilosis CDC317* : CPAR2_207070 and *C. auris B8441* : B9J08_003608	1.1919578	0.000205983
YLF2	Has domain(s) with predicted GTP binding activity	1.1917272	0.002409238
MEU1	Putative methylthioadenosine phosphorylase; protein level decreases in stationary phase cultures	1.1901838	3.67319E-07
PDS5	Putative protein with a predicted role in establishment and maintenance of sister chromatid condensation and cohesion; cell-cycle regulated periodic mRNA expression	1.1893908	0.002623803

PEA2	Putative coiled-coil polarisome; predicted role in polarized morphogenesis, cell fusion, and low affinity Ca2+ influx; rat catheter biofilm induced	1.1881296	0.005423584
ARO80	Zn(II)2Cys6 transcription factor; transcriptional activator of aromatic amino acid catabolism; regulator of aromatic alcohol biosynthesis via the Ehrlich pathway; mutant is viable	1.1869261	0.001688937
orf19.7102	Has domain(s) with predicted exonuclease activity and intracellular anatomical structure localization	1.1855783	0.000795373
orf19.2973	Ortholog(s) have RNA binding, flap-structured DNA binding activity and role in nuclear-transcribed mRNA catabolic process, nuclear-transcribed mRNA poly(A) tail shortening, positive regulation of endodeoxyribonuclease activity	1.1841134	0.000317301
orf19.6134	Ortholog(s) have role in ER-dependent peroxisome organization, retrograde vesicle-mediated transport, Golgi to endoplasmic reticulum, vesicle-mediated transport	1.1825581	0.005500939
CHT1	Chitinase; putative N-terminal catalytic domain; has secretory signal sequence; lacks S/T region and N-glycosylation motifs of Chs2p and Chs3p; alkaline downregulated; expression not detected in yeast-form or hyphal cells	1.180666	1.2658E-08
orf19.6701	Protein with similarity to amino acid-tRNA ligase; stationary phase enriched protein; GlcNAc-induced protein	1.1806466	8.83792E-10
VPS9	Ortholog(s) have guanyl-nucleotide exchange factor activity, ubiquitin binding activity	1.1805331	0.022096991
RPL38	60S ribosomal ribosomal protein subunit; genes encoding cytoplasmic ribosomal subunits, translation factors, tRNA synthetases are downregulated upon phagocytosis by murine macrophage	1.1797661	1.48648E-10
TRM10	Ortholog(s) have tRNA (guanine(9)-N(1))-methyltransferase activity, tRNA (guanine) methyltransferase activity and role in tRNA N1-guanine methylation, tRNA methylation	1.1784269	0.000874408
orf19.6558	Ortholog(s) have GTPase activator activity and cytosol localization	1.1776928	1.30714E-05
STE50	Protein with sterile alpha motif (SAM) and Ras-associated domain (RAD); similar to S. cerevisiae Rad50p, which is involved in signal transduction via interaction with and regulation of MAPKKK	1.1768224	4.45529E-10
orf19.5880	Putative voltage-gated chloride channel; predicted role in copper ion and iron ion homeostasis; flow model biofilm induced	1.1768032	0.017192164
orf19.949	Ortholog of *C. dubliniensis CD36* : Cd36_50420, *C. parapsilosis CDC317* : CPAR2_304100, *C. auris B8441* : B9J08_001314 and *Candida tenuis NRRL Y-1498* : CANTEDRAFT_98299	1.1754674	0.005247461
NAT1	Ortholog(s) have acetyltransferase activator activity, peptide alpha-N-acetyltransferase activity, ribosome binding activity	1.1732324	6.85388E-05

orf19.6586	Protein of unknown function; transcript induced by benomyl or in azole-resistant strain overexpressing MDR1; Ssn6 colony morphology-related regulation; induced by NO; Hap43-repressed; rat catheter and flow model biofilm induced	1.1705646	0.000278689
orf19.4518	Protein kinase of unknown function; mutants are viable	1.1691111	0.011541752
orf19.6039	Ortholog(s) have SNAP receptor activity	1.1690614	1.58675E-05
YUH2	Putative ubiquitin C-terminal hydrolase; sumoylation target	1.1666506	1.86503E-06
orf19.6818	Has domain(s) with predicted ATP binding, helicase activity, nucleic acid binding activity	1.1649494	0.000553127
MPS1	Monopolar spindle protein, putative spindle assembly checkpoint kinase; essential for growth; involved in oxidative stress response; periodic mRNA expression, peak at cell-cycle S/G2 phase	1.1635795	0.006966715
UFE1	Protein interacting with Sec20p, possibly involved in retrograde transport between the Golgi and the endoplasmic reticulum; functional homolog of S. cerevisiae Ufe1p, which is an ER t-SNARE that mediates the retrograde traffic	1.1624178	4.55518E-07
RPT2	Putative ATPase of the 19S regulatory particle of the 26S proteasome; oxidative stress-induced via Cap1; Spider biofilm repressed	1.1623793	1.05069E-08
orf19.1163	Has domain(s) with predicted GTP binding activity	1.161272	0.000222992
PAN3	Ortholog(s) have RNA binding, poly(A) binding, poly(A)-specific ribonuclease activity, role in DNA repair, nuclear-transcribed mRNA poly(A) tail shortening, postreplication repair and PAN complex localization	1.159561	0.00127023
CAF17	Ortholog(s) have role in protein maturation by [2Fe-2S] cluster transfer, protein maturation by [4Fe-4S] cluster transfer and mitochondrial matrix localization	1.1595334	3.88357E-05
THG1	tRNA guanylyltransferase, with role in tRNA modification; catalyzes reverse (3'-5') nucleotide polymerization	1.1593196	0.017691094
TIM22	Mitochondrial inner membrane protein; predicted role in protein import; Hap43-repressed gene; flow model biofilm induced; Spider biofilm repressed	1.157749	2.61272E-07
orf19.3806	Ortholog(s) have role in negative regulation of gluconeogenesis, proteasome-mediated ubiquitin-dependent protein catabolic process, traversing start control point of mitotic cell cycle and GID complex localization	1.1561475	0.019279863
orf19.5808	Ortholog(s) have S-adenosylmethionine-dependent methyltransferase activity, protein-lysine N-methyltransferase activity	1.1556434	0.002022199
FMP40	Putative protein of unknown function, transcription is positively regulated by Tbf1p	1.1525101	0.00325854

RIM1	Putative single-stranded DNA-binding protein; protein level decreases in stationary phase cultures; rat catheter biofilm repressed	1.1519713	2.51755E-10
ZDS1	Nonessential protein; similar to S. cerevisiae Zds1p	1.1513093	0.005326466
NAB2	Ortholog(s) have 5S rRNA binding, 7S RNA binding, poly(A) binding, ribonuclease P RNA binding, tRNA binding activity	1.1510579	6.33858E-06
orf19.1625	Putative ubiquinone oxidoreductase; repressed by nitric oxide; Hap43p-repressed	1.1501617	4.37464E-05
orf19.1460	Putative glutamine-dependent NAD synthetase, involved in NAD salvage pathway	1.1499188	0.001256398
orf19.1893	Protein of unknown function; Spider biofilm repressed	1.1494551	0.000859782
SAC3	Putative nuclear pore-associated protein, required for small ribosomal subunit biogenesis; possibly an essential gene, disruptants not obtained by UAU1 method	1.1491365	0.010276001
CLG1	Putative cyclin-like protein; transcription is regulated upon yeast-hyphal switch	1.1485824	1.36556E-10
orf19.7458	Ortholog of *C. dubliniensis CD36* : Cd36_86660, *Candida tenuis NRRL Y-1498* : cten_CGOB_00028, *Candida tropicalis NEW ASSEMBLY*: CTRG1_05695 and *Candida tropicalis MYA-3404* : CTRG_05695	1.1481796	0.010935261
RMT2	Minor protein arginine methyltransferases (PRMT) involved in methylation of arginine residues	1.1442069	4.04829E-05
orf19.397	Ortholog(s) have structural constituent of ribosome activity and mitochondrial large ribosomal subunit localization	1.1435182	1.54879E-08
orf19.5495	Putative RNA-binding protein; induced by alpha pheromone in SpiderM medium	1.1427118	0.0114073
CBK1	Ser/Thr kinase of cell wall integrity pathway; mutants show abnormal morphology and aggregation; Mob2p associated; required for wild-type hyphal growth and transcriptional regulation of cell-wall-associated genes	1.1422878	0.003089636
orf19.688	Mitochondrial ribosomal protein of the small subunit; S. cerevisiae ortholog is essential for viability; Spider biofilm repressed	1.1398003	2.35585E-07
TUS1	Putative guanine nucleotide exchange factor; induced by Mnl1 under weak acid stress; Spider biofilm induced	1.1370974	2.347E-05
CHD1	Ortholog(s) have ATP-dependent activity, acting on DNA, DNA binding, chromatin DNA binding, methylated histone binding, nucleosome-dependent ATPase activity, rDNA binding activity	1.1366625	0.003665425
orf19.6416	Protein involved in N-glycosylation; Spider biofilm induced; rat catheter biofilm repressed	1.1365897	0.000263527
MRS7	Member of the LETM1-like protein family, mitochondrial membrane protein	1.1358145	0.000395096
orf19.2091	Putative NADH dehydrogenase; repressed by nitric oxide, Hap43p-repressed	1.1348957	2.19576E-06

orf19.4931	Putative tRNA-Cys synthetase; induced by alpha pheromone in SpiderM medium; ribosomal subunits, translation factors, tRNA synthetases are downregulated upon phagocytosis by murine macrophage	1.1329264	0.001332933
MRE11	Putative DNA double-strand break repair factor; involved in response to oxidative stress and drug resistance	1.1320501	0.011997163
CRD1	Cardiolipin synthase; ortholog of S. cerevisiae Crd1; transcript is upregulated in clinical isolates from HIV+ patients with oral candidiasis; mutants are viable; Spider biofilm repressed	1.1320062	6.6191E-12
orf19.6118	Putative guanine nucleotide dissociation stimulator; transcription is positively regulated by Tbf1p	1.1318266	6.61118E-06
orf19.3175	Putative ortholog of human electron transfer flavoprotein dehydrogenase (ETF-dH); alkaline repressed; repressed by nitric oxide; virulence-group-correlated expression; Spider biofilm induced	1.1312689	8.10652E-08
OBPA	Similar to oxysterol binding protein; non-sex gene located within the MTLa mating-type-like locus; Plc1p-regulated	1.1278832	0.001952329
UME1	Ortholog of *C. dubliniensis CD36* : Cd36_73630, *C. parapsilosis CDC317* : CPAR2_805330, *C. auris B8441* : B9J08_000281 and *Candida tenuis NRRL Y-1498* : CANTEDRAFT_119330	1.1255994	0.000287676
RPN8	Putative regulatory subunit of the 26S proteasome; mutation confers hypersensitivity to amphotericin B; regulated by Mig1, Gcn2 and Gcn4; Spider biofilm repressed	1.1252245	7.51895E-10
FUN31	Putative PAS kinase involved in cell wall damage response; similar to S. cerevisiae Psk1p, a putative serine/threonine protein kinase; induced by Mnl1 under weak acid stress; rat catheter and Spider biofilm induced	1.1241606	0.020164374
orf19.1682	Membrane protein; Hap43p-repressed gene; repressed by nitric oxide	1.1235601	7.7231E-11
SES1	Seryl-tRNA synthetase; charges the tRNA that recognizes the CUG codon, which typically specifies Leu, but specifies Ser in C. albicans; complements S. cerevisiae ses1 mutant viability; soluble protein in hyphae; macrophage-regulated	1.1228863	6.77439E-08
orf19.4193	Ortholog(s) have Arp2/3 complex binding activity, role in actin filament debranching, negative regulation of Arp2/3 complex-mediated actin nucleation and actin cortical patch, cytoplasm localization	1.122021	6.85162E-05
COG4	Ortholog(s) have role in autophagy of peroxisome, cytoplasm to vacuole transport by the Cvt pathway, establishment of cell polarity and filamentous growth of a population of unicellular organisms	1.1218161	0.022829734
SRP54	Putative signal recognition particle (SRP) subunit; induced in ssr1 null; Spider biofilm repressed	1.121542	0.002269691

TSR2	Protein with a predicted role in pre-rRNA processing; repressed by prostaglandins	1.1214995	0.000982337
STI1	Protein that interacts with Cdc37 and Crk1 in two-hybrid; may be involved in Cdc37 chaperone activity; soluble protein in hyphae; protein in exponential and stationary phase yeast cultures; YNB biofilm induced; Spider biofilm induced	1.1201494	3.47739E-05
orf19.4293	Ortholog(s) have role in protein maturation by iron-sulfur cluster transfer, tRNA wobble uridine modification and CIA complex, cytosol, nucleus localization	1.1199645	0.00033293
SDH4	Succinate dehydrogenase, membrane subunit; induced in high iron	1.1187233	1.90218E-10
SFU1	GATA-type transcription factor; regulator of iron-responsive genes; represses iron utilization genes if iron is present; Hap43-repressed; promotes gastrointestinal commensalism in mice; Spider biofilm induced	1.1172092	0.001891279
RPN3	Putative non-ATPase regulatory subunit of the 26S proteasome lid; amphotericin B repressed; oxidative stress-induced via Cap1p	1.1151797	8.14116E-06
orf19.259	Protein with a predicted role in 60S ribosomal subunit assembly; flow model biofilm induced	1.1150904	0.0091182
QCR2	Ubiquinol-cytochrome-c reductase; antigenic; induced by interaction with macrophage; repressed by nitric oxide; in detergent-resistant membrane fraction (possible lipid raft component); levels decrease in stationary phase; Hap43p-repressed	1.1145336	1.13725E-06
ISM1	Protein similar to isoleucyl-tRNA synthetase; isoleucyl-tRNA synthetase is the target of drugs including the cyclic beta-amino acid icofungipen/PLD-118/BAY-10-8888 and mupirocin	1.113139	0.025324457
CIT1	Citrate synthase; induced by phagocytosis; induced in high iron; Hog1-repressed; Efg1-regulated under yeast, not hyphal growth conditions; present in exponential and stationary phase; Spider biofilm repressed; rat catheter biofilm induced	1.1125983	1.36024E-06
ZCF2	Zn(II)2Cys6 transcription factor, required for adaptation to reactive sulfur species; regulates sulfite tolerance through expression of SSU1 and CDG1; Hap43-repressed; Spider biofilm induced	1.1124747	0.001692175
KAR2	Similar to Hsp70 family chaperones; role in translocation of proteins into the ER; induced in high iron; protein present in exponential and stationary growth phase yeast cultures; flow model and Spider biofilm repressed	1.1117424	1.23167E-06
SWE1	Putative protein kinase with a role in control of growth and morphogenesis, required for full virulence; mutant cells are	1.1091988	0.026484473

	small, rounded, and sometimes binucleate; not required for filamentous growth; mutant is hypersensitive to caspofungin		
orf19.5678	Has domain(s) with predicted role in peptidyl-diphthamide biosynthetic process from peptidyl-histidine and cytoplasm localization	1.1083166	0.001087016
MRR1	Putative Zn(II)2Cys6 transcription factor; regulator of MDR1 transcription; gain-of-function mutations cause upregulation of MDR1 (a plasma membrane multidrug efflux pump) and multidrug resistance; Hap43-induced	1.107313	0.002684053
orf19.2398	Protein of unknown function; Hap43-repressed; transcript increased in azole-resistant strain overexpressing CDR1 and CDR2; possibly regulated by Tac1; induced by Mnl1 in weak acid stress; flow model biofilm induced; Spider biofilm induced	1.1070984	9.80967E-06
MES1	Cytoplasmic methionyl-tRNA synthetase; zinc-binding motif; ribosomal subunits, translation factors, tRNA synthetases are downregulated upon phagocytosis by murine macrophage; protein present in exponential and stationary phase yeast	1.1070705	5.1705E-08
PMI1	Phosphomannose isomerase; cell wall biosynthesis enzyme; drug target; functional homolog of S. cerevisiae, E. coli phosphomannose isomerase; Gcn4-regulated; induced on adherence to polystyrene, phagocytosis; 3-AT, Spider biofilm repressed	1.1060599	5.6593E-10
RSC2	Component of the RSC chromatin remodeling complex	1.1060052	0.000222458
UTP4	Putative U3 snoRNA-associated protein; Hap43-induced; physically interacts with TAP-tagged Nop1; Spider biofilm induced	1.1051012	0.004418336
ORC4	Phosphorylated protein similar to S. cerevisiae Orc4, subunit of the origin recognition complex (ORC); induced by alpha pheromone in SpiderM medium	1.103951	0.007643652
orf19.5782	Ortholog(s) have phospholipase activity, role in cardiolipin metabolic process, phosphatidylethanolamine metabolic process, phospholipid metabolic process and mitochondrial matrix localization	1.1014401	0.002539467
DBP2	Putative DEAD-box family ATP-dependent RNA helicase; flucytosine induced; repressed in core stress response	1.1009427	2.11804E-06
orf19.6903	Predicted RNA polymerase III subunit C37; Spider biofilm induced	1.0997645	0.000268783
orf19.5824	Protein of unknown function; rat catheter and Spider biofilm induced	1.0993412	0.001490347
YAR1	Ortholog(s) have unfolded protein binding activity	1.097047	0.001781581
orf19.4370	Protein of unknown function; induced by nitric oxide; oxidative stress-induced via Cap1; fungal-specific (no human or murine homolog)	1.0963454	0.000862803

YSH1	Putative endoribonuclease; heterozygous null mutant exhibits hypersensitivity to parnafungin and cordycepin in the C. albicans fitness test	1.0947248	0.020460687
STB2	Ortholog of *S. cerevisiae* : STB6, *C. glabrata CBS138* : CAGL0L05016g, *C. dubliniensis CD36* : Cd36_83110, *C. parapsilosis CDC317* : CPAR2_103210 and *C. auris B8441* : B9J08_001101	1.0943446	0.024296374
COQ5	Putative methyltransferase of ubiquinone biosynthesis; regulated by Gcn4; repressed by amino acid starvation (3-AT), Hap43; induced upon adherence to polystyrene; Spider biofilm repressed	1.093822	3.90778E-06
orf19.1630	Ortholog(s) have P-body localization	1.0917876	1.21455E-05
FOL2	Ortholog(s) have GTP cyclohydrolase I activity, role in folic acid-containing compound biosynthetic process and nucleus localization	1.0911973	0.000106906
SDH2	Succinate dehydrogenase, Fe-S subunit; localizes to surface of yeast cells, but not hyphae; induced in high iron and during log phase aerobic growth; repressed by nitric oxide, Hap43	1.0907489	3.40526E-09
CDC23	Similar to anaphase-promoting complex component; possibly transcriptionally regulated by Tac1p	1.0904905	0.008291139
CHT2	GPI-linked chitinase; required for normal filamentous growth; repressed in core caspofungin response; fluconazole, Cyr1, Efg1, pH-regulated; mRNA binds She3 and is localized to yeast-form buds and hyphal tips; Spider biofilm repressed	1.090459	2.37854E-09
RSR1	RAS-related protein; GTP/GDP cycling required for wild-type polar bud site selection, hyphal growth guidance; role in systemic virulence in mouse; geranylgeranylation predicted; suppresses S. cerevisiae cdc24-4 mutant heat sensitivity	1.0891387	3.75686E-06
orf19.2222	Putative casein kinase; plasma membrane-localized	1.0890482	0.027256779
PGA54	GPI-anchored protein; Hog1-repressed; induced in cyr1 or efg1 mutant or in hyphae; colony morphology-related gene regulation by Ssn6; induced in RHE model; mRNA binds She3; regulated in Spider biofilms by Tec1, Egf1, Ntd80, Rob1, Brg1	1.0878504	1.74825E-07
orf19.6555	Ortholog(s) have zinc ion binding activity, role in protein import into mitochondrial intermembrane space and mitochondrial intermembrane space localization	1.0875656	0.006279031
SEC13	Putative protein transport factor; antigenic during murine systemic infection; macrophage-downregulated protein; protein level decreases in stationary phase cultures; Spider biofilm repressed	1.0866884	5.17908E-06
orf19.6847	Putative tRNA U44 2'-O-methyltransferase; virulence-group-correlated expression; induced during oral infection; mutants have reduced ability to damage oral epithelial cells; early-stage flow model biofilm induced	1.0850721	0.004002619

CEF3	Translation elongation factor 3; antigenic in humans; predicted C-term nucleotide-binding active site; protein on surface of yeast, not hyphae; polystyrene adherence induced; higher protein amount in stationary phase; possibly essential	1.0838979	3.16973E-06
PTP1	Phosphotyrosine-specific protein phosphatase; rat catheter biofilm induced	1.0834866	1.23111E-07
orf19.804.1	Ortholog of *S. cerevisiae* : YPR010C-A, *C. dubliniensis CD36* : Cd36_02990, *C. parapsilosis CDC317* : CPAR2_211020, *C. auris B8441* : B9J08_000865 and *Candida tenuis NRRL Y-1498* : CANTEDRAFT_116148	1.0811137	9.47416E-06
GPR1	Plasma membrane G-protein-coupled receptor of the cAMP-PKA pathway; detects lactate and triggers signaling pathway that regulates beta-glucan masking and immune evasion; binds Gpa2; regulates HWP1 and ECE1; required for WT hyphal growth	1.079868	0.003535623
HIR3	Putative nucleosome assembly protein; homozygous transposon insertion causes decreased colony wrinkling under filamentous growth-inducing conditions, but does not block true hyphal formation in liquid media	1.0790412	0.007932022
orf19.5114.1	Ortholog of *C. dubliniensis CD36* : Cd36_02630, *C. parapsilosis CDC317* : CPAR2_206910, *Candida tenuis NRRL Y-1498* : cten_CGOB_00051 and *Pichia stipitis Pignal* : psti_CGOB_00173	1.0776258	2.16318E-07
RPN4	C2H2 transcription factor; putative regulator of proteasome genes; DNA recognition sequence (GAAGGCAAAA) enriched in regions upstream of proteasome genes; induced in core stress response; Hap43-induced; Spider biofilm induced	1.0766774	0.000448866
SSL2	Ortholog(s) have DNA helicase activity, DNA translocase activity	1.0764981	0.022563047
CAT5	Ortholog(s) have 2-octoprenyl-3-methyl-6-methoxy-1,4-benzoquinone hydroxylase activity, role in ubiquinone biosynthetic process and mitochondrial inner membrane, mitochondrion localization	1.0759887	5.2696E-06
GIN4	Autophosphorylated kinase; role in pseudohyphal-hyphal switch and cytokinesis; phosphorylates Cdc11p on S395; necessary for septin ring within germ tube but not for septin band at mother cell junction; physically associates with septins	1.0752469	0.001133598
CPR6	Putative peptidyl-prolyl cis-trans isomerase; macrophage/pseudohyphal-repressed; heavy metal (cadmium) stress-induced; heterozygous null mutant displays sensitivity to virgineone; rat catheter biofilm induced	1.0749447	4.92229E-09
orf19.5431	Protein of unknown function; Hap43-repressed; Spider biofilm induced	1.073373	0.001404204

KRS1	Putative tRNA-Lys synthetase; repressed upon phagocytosis by murine macrophages; stationary phase enriched protein; Spider biofilm repressed	1.0706194	2.494E-05
PHO89	Putative phosphate permease; transcript regulated upon white-opaque switch; alkaline induced by Rim101; possibly adherence-induced; F-12/CO2 model, rat catheter and Spider biofilm induced	1.0692013	0.002630118
orf19.4396	Mitochondrial inner membrane protein; mammalian mitofilin domain; Spider biofilm repressed	1.0684639	1.41583E-06
orf19.5468	Predicted ORF from Assembly 19; removed from Assembly 20; restored based on transcription data; almost identical to orf19.6113, orf19.4055, orf19.5370	1.0683959	0.015242727
TAF9	Ortholog(s) have chromatin binding, molecular adaptor activity and role in RNA polymerase II preinitiation complex assembly, chromatin organization, histone acetylation, transcription by RNA polymerase II	1.0680564	0.002817358
CSL4	Ortholog(s) have role in exonucleolytic trimming to generate mature 3'-end of 5.8S rRNA from tricistronic rRNA transcript (SSU-rRNA, 5.8S rRNA, LSU-rRNA) and mRNA processing	1.0676387	0.004216477
HAP43	CCAAT-binding factor-dependent transcription factor; repressor; also called CAP2; required for low iron response; similar to bZIP transcription factor AP-1; repressed by Sfu1; ciclopirox olamine induced; rat catheter, Spider biofilm induced	1.0675816	0.021455406
NAR1	Putative cytosolic iron-sulfur (FeS) protein assembly machinery protein; induced by nitric oxide; oxidative stress-induced via Cap1p	1.0672064	6.51496E-05
orf19.4398	Protein of unknown function; rat catheter biofilm induced	1.0659173	0.010092063
orf19.2101	Ortholog(s) have protein-membrane adaptor activity, role in mitophagy, protein insertion into ER membrane, retrograde vesicle-mediated transport, Golgi to endoplasmic reticulum and GET complex localization	1.0653417	4.66922E-08
CAR1	Arginase; arginine catabolism; transcript regulated by Nrg1, Mig1, Tup1; colony morphology-related regulation by Ssn6; alkaline induced; protein decreased in stationary phase; sumoylation target; flow model biofilm induced	1.0646505	9.49151E-07
RPN10	Putative 19S regulatory particle of the 26S proteasome; macrophage/pseudohyphal-repressed; regulated by Gcn2 and Gcn4; Spider biofilm repressed	1.0623505	2.29396E-06
orf19.5848	RING-type zinc finger protein; upregulated during oral infection; mutants have reduced ability to damage oral epithelial cells; Spider and flow model biofilm induced	1.0622342	0.020623357
NAG3	Putative MFS transporter; similar to Nag4; required for wild-type mouse virulence and cycloheximide resistance; in gene	1.0621926	3.28226E-10

	cluster that includes genes encoding enzymes of GlcNAc catabolism; Spider biofilm repressed		
GRS1	Putative tRNA-Gly synthetase; genes encoding ribosomal subunits, translation factors, tRNA synthetases are downregulated upon phagocytosis by murine macrophage; stationary phase enriched protein	1.0614457	3.89075E-07
orf19.3977	Protein with a role in translation; flow model biofilm repressed	1.0610934	3.12392E-05
VAN1	Member of Mnn9 family of mannosyltransferases; ortholog of S. cerevisiae Van1p; fungal-specific (no human or murine homolog)	1.0594043	0.003178821
orf19.1144	Protein with SEL-1 like protein domain; early-stage flow model biofilm induced	1.0588937	0.011143813
HFL1	HAP5-like; ortholog of S. cerevisiae Dpb3; third-largest subunit of DNA polymerase II (DNA polymerase epsilon); phosphorylated protein; mutants have a growth defect	1.0560403	0.000301507
MNR2	Putative ion transporter; fungal-specific (no human or murine homolog)	1.0550255	0.000766192
LAP4	Protein similar to aminopeptidase I; mutation confers hypersensitivity to amphotericin B; transcript regulated in macrophage response; flow model biofilm induced	1.0545089	0.000550339
NUP	Nucleoside permease; adenosine and guanosine are substrates, whereas cytidine, adenine, guanine, uridine, uracil are not; similar to a nucleoside permease of S. pombe; possibly processed by Kex2p	1.054484	0.007105401
DCK2	Protein similar to S. cerevisiae Ylr422wp; transposon mutation affects filamentous growth; induced by Mnl1p under weak acid stress	1.0543461	0.001450677
RPS10	Ribosomal protein S10; downregulated in the presence of human whole blood or PMNs; Spider biofilm repressed	1.0515323	2.61728E-07
orf19.3569	Protein of unknown function; S. cerevisiae YLR407W mutants have abnormal budding; constitutive expression independent of MTL or white-opaque status; Spider biofilm induced	1.0502775	0.017489677
orf19.1181	Has domain(s) with predicted catalytic activity and membrane localization	1.049695	7.98845E-05
TIF35	Putative translation initiation factor; repressed upon phagocytosis by murine macrophage; Spider biofilm repressed	1.0493027	8.73121E-09
GDA1	Golgi membrane GDPase, required for wild-type O-mannosylation, not N-glycosylation; required for wild-type hyphal induction, cell wall, and cell surface charge; not required for HeLa cell adherence; functional homolog of S. cerevisiae Gda1p	1.0481283	0.000688523
SHP1	Regulator of the type 1 protein phosphatase Glc7p activity, involved in control of morphogenesis, progression through the cell cycle and response to DNA damage	1.0476888	3.95445E-05

GCN20	YEF3-subfamily ABC family protein, predicted not to be a transporter	1.0468542	0.00023352
TRP5	Predicted tryptophan synthase; identified in detergent-resistant membrane fraction (possible lipid raft component); predicted N-terminal acetylation; Gcn4p-regulated; S. cerevisiae ortholog is Gcn4p regulated; upregulated in biofilm	1.0450817	9.67586E-07
GZF3	GATA-type transcription factor; oxidative stress-induced via Cap1; mutant has abnormal colony morphology and altered sensitivity to fluconazole, LiCl, and copper; Spider biofilm induced	1.0442872	0.004502088
CTA7	Zn(II)2Cys6 transcription factor; activates transcription in 1-hybrid assay in S. cerevisiae; has similarity to S. cerevisiae Stb4	1.0442773	0.00867448
orf19.268	Ortholog(s) have role in DNA repair, transcription-coupled nucleotide-excision repair and Ddb1-Ckn1 complex localization	1.0441105	0.000376067
orf19.6407	Ortholog(s) have role in ribosomal large subunit biogenesis	1.0429086	0.015328022
SAP8	Secreted aspartyl protease; regulated by growth phase, temperature, white-opaque switch; highly expressed in opaque cells and upon deep epidermal invasion; greater expression in vaginal than oral infection; prominent role in biofilms	1.042383	0.003556088
orf19.4912	Ortholog(s) have ubiquitin protein ligase binding activity, role in positive regulation of receptor internalization, protein ubiquitination, ubiquitin-dependent endocytosis and Golgi apparatus, cytosol, plasma membrane localization	1.0413643	0.024279619
orf19.3163	Ortholog of *C. dubliniensis CD36* : Cd36_51910, *C. parapsilosis CDC317* : CPAR2_101650, *C. auris B8441* : B9J08_001942 and *Candida tenuis NRRL Y-1498* : CANTEDRAFT_94797	1.0413408	0.007883661
GDH3	NADP-glutamate dehydrogenase; Nrg1, Plc1 regulated; hypha, hypoxia, Efg1-repressed; Rim101-induced at pH 8; GlcNAc, ciclopirox, ketoconazole induced; exp and stationary phase protein; Spider biofilm repressed; rat catheter biofilm induced	1.0410159	4.54853E-08
UBI3	Fusion of ubiquitin with the S34 protein of the small ribosomal subunit; mRNA decreases upon heat shock, appears to be degraded; functional homolog of S. cerevisiae RPS31; Hap43-induced; Spider biofilm repressed	1.0400057	3.7238E-11
DCP2	Ortholog(s) have chromatin binding, hydrolase activity, m7G(5')pppN diphosphatase activity, mRNA binding activity	1.0375757	0.000705811
CYM1	Putative metalloprotease of the mitochondrial intermembrane space; rat catheter biofilm induced	1.0373012	0.005194118
PRP9	Ortholog(s) have RNA binding activity, role in mRNA 5'-splice site recognition, mRNA splicing, via spliceosome and U2-type prespliceosome localization	1.0341691	0.025721083

RPS21B	Ribosomal protein S21; regulated by Nrg1, Tup1; colony morphology-related gene regulation by Ssn6; positively regulated by Tbf1, Hap43; Spider biofilm repressed	1.0341502	1.14699E-05
MDL2	Putative mitochondrial, half-size MDR-subfamily ABC transporter	1.0330019	0.00314658
orf19.3817	Has domain(s) with predicted nucleic acid binding, zinc ion binding activity	1.0329619	0.008087131
GLC7	Putative catalytic subunit of type 1 serine/threonine protein phosphatase; regulated by Shp1; induced in high iron; alternatively spliced intron in 5' UTR	1.0315516	9.88446E-08
orf19.5963	Putative prenyltransferase; essential gene in S. cerevisiae; Spider biofilm induced	1.0315095	0.014407222
orf19.5422	Ortholog of *C. dubliniensis CD36* : Cd36_80400, *Candida tropicalis NEW ASSEMBLY* : CTRG1_06188, *Candida tropicalis MYA-3404* : CTRG_06188 and *Candida albicans WO-1* : CAWG_02379	1.0284048	0.023075809
orf19.4857	Protein with a dual-specificity phosphatase domain; Hap43-induced gene	1.0274117	0.017997127
TPO4	Putative sperimidine transporter; fungal-specific (no human or murine homolog); Spider biofilm induced; promoter bound by Tec1 and Ndt80; Bcr1-repressed in RPMI a/a biofilms	1.0263144	0.002085318
HNT2	Putative dinucleoside triphosphate hydrolase; induced upon low-level peroxide stress	1.0237936	0.002385316
ENA21	Predicted P-type ATPase sodium pump; Gcn4p-regulated; flucytosine, amphotericin B, or ketoconazole-induced; osmotic stress-induced; overlaps orf19.5170.1, which is annotated as a blocked reading frame; Spider biofilm induced	1.0236581	3.80876E-06
KRE1	Cell wall glycoprotein; beta glucan synthesis; increases glucan content in S. cerevisiae kre1, complements killer toxin sensitivity; caspofungin induced; Spider/rat catheter/flow model biofilm induced; Bcr1-repressed in RPMI a/a biofilms	1.0236079	0.026868427
orf19.5238	Ortholog of *C. dubliniensis CD36* : Cd36_11490, *C. parapsilosis CDC317* : CPAR2_701040, *C. auris B8441* : B9J08_003931 and *Candida tenuis NRRL Y-1498* : CANTEDRAFT_104365	1.0220745	0.001648791
TMA19	Cell wall protein, ortholog of S. cerevisiae Tma19p (Ykl065cp)	1.0219887	1.62481E-08
orf19.6160	Ortholog(s) have role in eisosome assembly and eisosome, membrane raft localization	1.0213285	0.001278615
ZNC1	Zn(2)-Cys(6) transcription factor; regulated by Gcn2 and Gcn4; gene located in zinc cluster region of Chromosome 5, near the MTL locus; required for yeast cell adherence to silicone substrate; Spider biofilm induced	1.0183885	0.005933628
orf19.1544	Putative cis-golgi localized protein involved in ER to Golgi transport; Spider biofilm repressed	1.0182998	0.005050186

orf19.7664	Ortholog of *S. cerevisiae* : YCR087C-A, *C. glabrata CBS138* : CAGL0E00517g, *C. dubliniensis CD36* : Cd36_35524, *C. parapsilosis CDC317* : CPAR2_200040 and *C. auris B8441* : B9J08_001263	1.0173994	2.43645E-06
WOR2	Zn(II)2Cys6 transcription factor; regulator of white-opaque switching; required for maintenance of opaque state; Hap43-induce	1.0151242	0.002221932
MIS11	Predicted mitochondrial C1-tetrahydrofolate synthase precursor; putative protein of glycine catabolism; repressed by Efg1; fluconazole-induced; stationary phase enriched protein; rat catheter and Spider biofilm repressed	1.0130562	0.000235588
CDC50	Predicted non-catalytic subunit of phospholipid flippase; involved in endocytosis, hyphal development, drug resistance; mutants show attenuated virulence in mouse model; induced by Mnl1p under weak acid stress	1.0113868	0.001323291
orf19.2343.1	Putative vacuolar H+ ATPase subunit e of the V-ATPase V0 subcomplex; added to Assembly 21 based on comparative genome analysis	1.0113414	1.74164E-05
orf19.229	Ortholog(s) have ubiquitin-protein transferase activity, role in ubiquitin-dependent protein catabolic process and chromosome, centromeric region, site of double-strand break localization	1.0111851	0.000521154
ORC3	Protein similar to S. cerevisiae Orc3p, which is a component of the origin recognition complex involved in DNA replication; mutation confers hypersensitivity to toxic ergosterol analog; induced under hydroxyurea treatment	1.010861	0.007914736
orf19.549	Ortholog(s) have structural constituent of ribosome activity and mitochondrial large ribosomal subunit localization	1.0063314	1.41144E-06
orf19.1872	Plasma membrane protein; repressed by nitric oxide	1.006056	2.14296E-05
LEM3	Putative membrane protein; mutation increases resistance to miltefosine; early-stage flow model biofilm induced	1.0048527	0.004418486
RRP7	Ortholog(s) have rRNA binding activity, role in rRNA processing, ribosomal small subunit assembly and CURI complex, UTP-C complex localization	1.0031458	1.859E-05
JEM1	Functional homolog of S. cerevisiae Jem1p, which acts with Scj1p and Kar2p (BiP) in protein folding and ER-associated degradation of misfolded proteins, and also has a role in karyogamy; has J domain and 4 tetratricopeptide repeats	1.002815	0.025600267
orf19.6556	Protein of unknown function; rat catheter, flow model and Spider biofilm induced	1.0026652	0.007511604
NOP1	Nucleolar protein; flucytosine induced; Hap43-induced; Spider biofilm repressed	1.0023468	8.3827E-09
CWC24	Putative pre-mRNA-splicing factor; possibly an essential gene, disruptants not obtained by UAU1 method	1.0021711	0.004728196

RPS23A	Putative ribosomal protein; repressed upon phagocytosis by murine macrophage; Spider biofilm repressed	1.0020717	2.62595E-10
orf19.2007	Ortholog(s) have role in Golgi to vacuole transport, ascospore wall assembly, cellular sphingolipid homeostasis, retrograde transport, endosome to Golgi and GARP complex, Golgi apparatus localization	1.0020281	0.012541384
SSL1	Putative RNA polymerase transcription factor TFIIH core component; possibly an essential gene, disruptants not obtained by UAU1 method	1.0006836	0.000991513
orf19.4400	Has domain(s) with predicted role in anaphase-promoting complex-dependent catabolic process, regulation of mitotic metaphase/anaphase transition and anaphase-promoting complex localization	1.0005321	0.021152196
URA1	Dihydroorotate dehydrogenase; de novo pyrimidine biosynthesis; regulated by yeast-hypha switch, Nrg1/Mig1/Tup1; flow model biofilm induced; rat catheter and Spider biofilm repressed	-1.000672	4.46952E-09
PRE2	Putative proteasome beta-5 subunit; macrophage-induced protein	-1.002455	3.05557E-11
orf19.1794	Ortholog(s) have mRNA 5'-UTR binding, pre-mRNA intronic binding, translation regulator activity and role in Group I intron splicing, mitochondrial mRNA processing, positive regulation of mitochondrial translation	-1.002749	0.001473516
orf19.4678	Predicted triglyceride lipase; Spider biofilm induced	-1.003165	0.000437622
orf19.2499	Putative peptidyl-prolyl cis-trans isomerase	-1.003212	0.000205028
orf19.6311	Protein of unknown function; Hap43-induced; rat catheter and Spider biofilm induced	-1.004112	0.016434247
RBR2	Cell wall protein; expression repressed by Rim101; transcript regulated upon white-opaque switching; repressed by alpha pheromone in SpiderM medium; macrophage-induced gene	-1.005798	9.9277E-06
IFF3	Putative GPI-anchored protein	-1.006283	0.000905982
SNX4	Putative sorting nexin; induced during the mating process	-1.008529	2.66379E-05
HGH1	Putative HMG1/2-related protein; transcript regulated by Mig1	-1.009896	9.42184E-09
AGC1	Putative mitochondrial carrier protein; transcript is alkaline upregulated rat catheter biofilm induced	-1.012257	2.42319E-06
MRPL10	Putative mitochondrial large subunit ribosomal protein; colony morphology-related gene regulation by Ssn6	-1.012429	1.69349E-08
orf19.1037	Protein of unknown function; rat catheter biofilm repressed	-1.012893	0.012079017
orf19.1527	Protein of unknown function; Spider biofilm induced	-1.013048	0.002181135
MRF1	Putative mitochondrial respiratory protein; induced by farnesol, benomyl, nitric oxide, core stress response; oxidative stress-induced via Cap1; stationary-phase enriched protein; Spider biofilm induced	-1.015648	7.69935E-13

orf19.2244	Similar to oxidoreductases and to S. cerevisiae Yjr096wp; Sfu1 repressed; induced by benomyl treatment, Ssr1; Hap43-repressed; flow model biofilm repressed	-1.016489	7.52156E-14
ARO9	Aromatic transaminase; Ehrlich fusel oil pathway of aromatic alcohol biosynthesis; Rim101-dependent pH-regulation (alkaline induced); Hap43-induced gene	-1.016531	3.12568E-05
orf19.6348	Predicted cysteine proteinase domain; mutants are viable	-1.019365	0.011271133
BZZ1	Protein similar to S. cerevisiae Bzz1p, which is an SH3 domain protein involved in the regulation of actin polymerization	-1.019662	1.42135E-06
CHO1	Putative phosphatidylserine synthase; ortholog of S. cerevisiae CHO1; transposon mutation affects filamentous growth; regulated by Nrg1, Tup1	-1.02245	8.90595E-07
IMP1	Predicted subunit of the mitochondrial inner membrane peptidase complex involved in protein targeting to mitochondria	-1.022803	2.96074E-05
orf19.4357	Putative protein similar to S. cerevisiae Mgr3p, a subunit of the i-AAA protease supercomplex that degrades misfolded mitochondrial proteins	-1.023797	2.48375E-08
orf19.2305	Ortholog(s) have tubulin binding activity, role in tubulin complex assembly and cytoplasm, prefoldin complex localization	-1.024542	0.008645244
IDP2	Isocitrate dehydrogenase; white-opaque switch regulated; morphology-regulation by Ssn6; protein in exponential and stationary phase yeast; Hap43-repressed; Spider biofilm repressed by Bcr1, Tec1, Ndt80, Rob1, Brg1; Spider biofilm induced	-1.024769	1.31405E-07
orf19.4615	Ortholog(s) have histone deacetylase activity	-1.025193	2.84435E-05
orf19.6008	S. cerevisiae ortholog YLL032C interacts with ribosomes; repressed by alpha pheromone in SpiderM medium	-1.02605	0.001709703
orf19.4380.1	Ortholog(s) have role in mitochondrial genome maintenance and integral component of mitochondrial inner membrane localization	-1.02703	1.80628E-05
orf19.2043	Has domain(s) with predicted ATP binding, ATPase, iron-sulfur cluster binding activity and role in iron-sulfur cluster assembly	-1.029907	0.010122431
orf19.2835	Ortholog(s) have SUMO activating enzyme activity, role in mitotic chromosome condensation, protein sumoylation, regulation of mitotic chromosome condensation and SUMO activating enzyme complex, cytosol, nucleus localization	-1.030351	0.000408792
orf19.2106	Putative protein of unknown function; Hap43p-repressed gene	-1.03117	2.22217E-06
RIB4	Lumazine synthase (6,7-dimethyl-8-ribityllumazine synthase, DMRL synthase); catalyzes the penultimate step in the synthesis of riboflavin; Hap43-induced; rat catheter and Spider biofilm repressed	-1.032468	8.06493E-15

MYO1	Component of actomyosin ring at neck of newly-emerged bud	-1.033947	0.000330852
SAM37	Protein of the mitochondrial outer membrane, component of the Sorting and Assembly Machinery (SAM) involved in insertion of proteins into the outer membrane; mutants show loss of mtDNA	-1.033967	0.000890139
orf19.6530	Similar to bacterial DnaJ; transcript upregulated in low iron; flow model biofilm induced; Spider biofilm induced	-1.034026	0.003629225
PCL5	Putative cyclin for Pho85 kinase; Gcn4-induced; suppresses toxicity of C. albicans Gcn4 overproduction in S. cerevisiae via increased Pho85-dependent phosphorylation and degradation of Gcn4; rat catheter and Spider biofilm induced	-1.036311	0.00223086
NOP8	Ortholog of S. cereviiae Nop8; has a role in ribosomal large subunit biogenesis; rat catheter and Spider biofilm induced	-1.038819	0.001133295
PCL2	Cyclin homolog; reduced expression observed upon depletion of Cln3; farnesol regulated; periodic mRNA expression, peak at cell-cycle G1/S phase; Hap43-induced; rat catheter biofilm repressed	-1.039245	0.012103442
SOU1	Enzyme involved in utilization of L-sorbose; has sorbitol dehydrogenase, fructose reductase, and sorbose reductase activities; NAD-binding site motif; transcriptional regulation affected by chromosome 5 copy number; Hap43p-induced gene	-1.041156	1.83783E-05
orf19.4583	Protein with a mitochondrial carrier protein domain; possibly an essential gene, disruptants not obtained by UAU1 method; Spider biofilm repressed	-1.042118	1.18214E-05
BIG1	Endoplasmic reticulum (ER) protein; ortholog of S. cerevisiae Big1; required for beta-1,6-glucan synthesis, filamentation, adhesion, and virulence; rat catheter biofilm repressed	-1.042454	4.23709E-08
orf19.91	Protein of unknown function; flow model biofilm induced; Hap43-repressed	-1.043361	0.001355202
RNH1	Ribonuclease H (RNAse H); hyphal-induced; flucytosine induced; similar to orf19.5564 (see Locus History); possibly essential (UAU1 method); rat catheter biofilm induced; flow model biofilm repressed	-1.043513	1.33798E-13
orf19.2921	Ortholog(s) have alpha-tubulin binding activity, role in protein folding, tubulin complex assembly and cytoplasm localization	-1.043641	0.001047691
CRP1	Copper transporter; CPx P1-type ATPase; mediates Cu resistance; similar to Menkes and Wilson disease proteins; copper-induced; Tbf1-activated; suppresses Cu sensitivity of S. cerevisiae cup1 mutant; flow model biofilm induced	-1.044934	0.001149565
MLS1	Malate synthase; glyoxylate cycle enzyme; no mammalian homolog; regulated upon white-opaque switch; phagocytosis, strong oxidative stress induced; stationary phase enriched;	-1.04608	4.3631E-05

	flow model biofilm repressed; rat catheter, Spider biofilm induced		
ALG11	Alpha-1,2-mannosyltransferase; catalyzes sequential addition of 2 terminal alpha 1,2-mannose residues to the Man5GlcNAc2-PP-dolichol intermediate during asparagine-linked glycosylation in the ER; Spider biofilm induced	-1.049306	9.14129E-05
ERG4	Protein similar to sterol C-24 reductase; shows Mob2p-dependent hyphal regulation; fluconazole-induced; caspofungin repressed; rat catheter biofilm repressed	-1.049875	9.34145E-10
orf19.164	Ortholog(s) have triglyceride lipase activity, role in triglyceride catabolic process and peroxisomal matrix localization	-1.054067	1.51337E-08
orf19.4382	Ortholog(s) have role in retrograde vesicle-mediated transport, Golgi to endoplasmic reticulum and COPI vesicle coat localization	-1.055659	2.70168E-08
orf19.3887	Ortholog of S. cerevisiae : YML108W, C. glabrata CBS138 : CAGL0J06666g, C. dubliniensis CD36 : Cd36_31830, C. parapsilosis CDC317 : CPAR2_204870 and C. auris B8441 : B9J08_000809	-1.055874	0.000530767
PHO84	High-affinity phosphate transporter; transcript regulated by white-opaque switch; Hog1, ciclopirox olamine or alkaline induced; caspofungin, stress repressed; required for normal TORC1 function	-1.056366	2.46361E-08
PLB2	Putative phospholipase B; conserved catalytic region; 6 putative N-glycosylation motifs; predicted secretion signal; no GPI anchor predicted; fungal-specific (no human or murine homol	-1.058268	0.000207912
orf19.1083	Putative protein of unknown function; macrophage-induced gene	-1.060315	0.000322357
TPS2	Trehalose-6-phosphate (Tre6P) phosphatase; mutant heat sensitive, accumulates Tre6P, decreased mouse virulence; possible drug target; 2 conserved phospohydrolase motifs; no mammalian homolog; Hap43-repressed; flow model biofilm induced	-1.060622	2.17796E-06
orf19.5553	Ortholog(s) have methionine-R-sulfoxide reductase activity and role in cellular response to oxidative stress	-1.062129	4.56711E-07
FRP6	Putative ammonia transport protein; regulated by Nrg1 and Tup1; regulated by Ssn6; induced by human neutrophils	-1.062911	4.48306E-13
orf19.1723	Ortholog(s) have role in response to purine-containing compound	-1.064596	5.46014E-07
orf19.2670	Ortholog(s) have 3-hydroxyacyl-[acyl-carrier-protein] dehydratase activity and mitochondrion localization	-1.065522	3.18216E-08
orf19.2682	Ortholog(s) have TBP-class protein binding, transcription coregulator activity and role in RNA polymerase II preinitiation	-1.067158	2.84915E-06

	complex assembly, transcription initiation from RNA polymerase II promoter		
MNT1	Alpha-1,2-mannosyl transferase; predicted type II Golgi membrane protein; adds 2nd mannose during cell-wall mannoprotein biosynthesis; required for wild-type virulence and adherence to epithelial cells; Hap43-induced	-1.069593	5.93375E-15
YSA1	Predicted Nudix hydrolase family member with ADP-ribose pyrophosphatase activity; role in metabolism of O-acetyl-ADP-ribose to AMP and acetylated ribose 5'-phosphate; rat catheter biofilm repressed	-1.069763	2.14644E-08
BBC1	Putative SH3-domain-containing protein	-1.070556	1.03634E-05
orf19.4172	Has domain(s) with predicted hydrolase activity and role in metabolic process	-1.071622	7.33179E-05
TDH3	NAD-linked glyceraldehyde-3-phosphate dehydrogenase; binds fibronectin, laminin; at cell surface; antigenic in infection; farnesol-repressed; stationary phase-enriched; GlcNAc-induced; flow model biofilm induced; Spider biofilm repressed	-1.072555	2.60287E-14
ERG10	Acetyl-CoA acetyltransferase; role in ergosterol biosynthesis; soluble in hyphae; changes in protein abundance associated with azole resistance; fluconazole or ketoconazole induced; macrophage-downregulated protein; GlcNAc-induced protein	-1.07373	2.50436E-10
FGR14	Protein encoded in retrotransposon Zorro3 with similarity to retroviral endonuclease-reverse transcriptase proteins; lacks an ortholog in S. cerevisiae; transposon mutation affects filamentous growth	-1.077074	1.06639E-09
orf19.993	Protein of unknown function; rat catheter biofilm repressed	-1.080617	7.11721E-09
orf19.5041	Ortholog of C. dubliniensis CD36 : Cd36_43600, C. parapsilosis CDC317 : CPAR2_403780, Candida tenuis NRRL Y-1498 : CANTEDRAFT_93767 and Debaryomyces hansenii CBS767 : DEHA2G16984g	-1.082255	0.004468078
orf19.1632	Has domain(s) with predicted catalytic activity and role in carbohydrate metabolic process	-1.084928	5.79777E-07
GST2	Glutathione S transferase; induced by benomyl and in populations of cells exposed to fluconazole over multiple generations; regulated by Nrg1, Tup1; induced by nitric oxide; stationary phase enriched; Spider biofilm induced	-1.084982	1.23218E-13
RAD3	Ortholog of S. cerevisiae Rad3; 5' to 3' DNA helicase, nucleotide excision repair and transcription, subunit of RNA polII initiation factor TFIIH and Nucleotide Excision Repair Factor 3	-1.085373	6.70247E-13
orf19.1800	Protein of unknown function; Spider biofilm induced	-1.089344	8.01789E-06
orf19.4529	Ortholog of Srp21, signal recognition particle subunit, functions in protein targeting to the endoplasmic reticulum membrane; predicted adhesin-like protein; mutants are viable	-1.090557	7.34972E-05

GAL7	Putative galactose-1-phoshphate uridyl transferase; downregulated by hypoxia, upregulated by ketoconazole; macrophage/pseudohyphal-repressed	-1.091296	1.81763E-09
ARV1	Lipid transporter involved in sterol trafficking and transport of glycosylphosphatidylinositol and sphingolipid precursors	-1.091412	0.011609424
orf19.1533	Possible vacuolar protein; Hap43-induced gene	-1.092291	1.26835E-05
orf19.4171	Has domain(s) with predicted ATP binding activity	-1.092558	0.003718352
ATG11	Adapter protein for pexophagy and the cytoplasm-to-vacuole targeting (Cvt) pathway; Spider biofilm induced	-1.093918	0.007607366
orf19.1772	Ortholog of S. cerevisiae : MRX1, C. glabrata CBS138 : CAGL0J03278g, C. dubliniensis CD36 : Cd36_24130, C. parapsilosis CDC317 : CPAR2_407430 and C. auris B8441 : B9J08_003802	-1.093976	0.007736461
orf19.4530.1	Protein of unknown function; regulated by Nrg1, Tup1; Spider and flow model biofilm induced	-1.095917	1.88584E-08
HMI1	ATP-dependent 3' - 5' helicase involved in maintenance of mitochondrial DNA; ortholog of S. cerevisiae Hmi1; rat catheter biofilm repressed	-1.095969	0.007802288
orf19.4657	Ortholog(s) have phosphoprotein phosphatase activity	-1.096567	0.007427991
orf19.6973	ATP-dependent LON protease family member; Hap43-repressed gene; regulated by Gcn2 and Gcn4; Spider biofilm induced	-1.097269	0.00559885
RPF2	Putative pre-rRNA processing protein; Hap43p-induced gene; mutation confers hypersensitivity to 5-fluorocytosine (5-FC), 5-fluorouracil (5-FU), and tubercidin (7-deazaadenosine)	-1.101168	9.18075E-09
POR1	Mitochondrial outer membrane porin; in detergent-resistant membrane fraction (possible lipid raft component); antigenic in human, mouse; Hap43p-induced; flucytosine-, macrophage-, farnesol- induced; fluconazole, caspofungin repressed	-1.104606	8.95818E-19
orf19.4894	Protein with similarity to S. cerevisiae Yer010cp, a protein of unknown function belonging to the prokaryotic RraA family; repressed by benomyl; Hap43-induced; Spider biofilm induced	-1.104999	0.000460128
GAD1	Putative glutamate decarboxylase; alkaline, macrophage-downregulated gene; amphotericin B induced; induced by Mnl1 under weak acid stress; stationary phase enriched protein; rat catheter biofilm repressed	-1.108152	1.44231E-10
orf19.5523	Ortholog of C. dubliniensis CD36 : Cd36_62780, C. parapsilosis CDC317 : CPAR2_601690, C. auris B8441 : B9J08_001638 and Candida tenuis NRRL Y-1498 : CANTEDRAFT_113271	-1.109051	0.000327953
FBA1	Fructose-bisphosphate aldolase; glycolytic enzyme; antigenic in murine/human infection; regulated by yeast-hypha switch; induced by Efg1, Gcn4, Hog1, fluconazole; phagocytosis-	-1.110663	6.03733E-14

	repressed; flow model biofilm induced; Spider biofilm repressed		
MRP20	Component of mitochondrial ribosome; decreased expression in hyphae compared to yeast-form cells	-1.111314	1.07084E-08
GEM1	Putative outer mitochondrial membrane GTPase, subunit of the ERMES complex; required for mitochondrial morphology and invasive growth	-1.11574	0.000120454
HOM6	Putative homoserine dehydrogenase; Gcn4-regulated; induced by amino acid starvation (3-ATtreatment); macrophage-induced protein; protein level decreases in stationary phase cultures; flow model biofilm repressed	-1.115859	3.50162E-18
PDC11	Pyruvate decarboxylase; antigenic; on hyphal not yeast cell surface; Hap43, Gcn4, Efg1, Efh1, Hsf1 regulated; fluconazole, farnesol induced; amino acid starvation repressed; flow model biofilm induced; Spider biofilm repressed	-1.116529	1.63808E-12
ILV5	Ketol-acid reductoisomerase; antigenic; regulated by Gcn4; GlcNAc, amino acid starvation (3-AT)-induced; macrophage-repressed protein; protein present in exponential and stationary phase; flow model and Spider biofilm repressed	-1.117304	1.93885E-12
orf19.6198.1	Ortholog of S. cerevisiae : YIL156W-B, C. glabrata CBS138 : CAGL0H06732g, C. dubliniensis CD36 : Cd36_06600, C. parapsilosis CDC317 : CPAR2_208850 and C. auris B8441 : B9J08_005435	-1.117648	0.000605684
orf19.4368	Has domain(s) with predicted hydrolase activity and role in cellular process	-1.118379	0.001290246
orf19.6732	Ortholog(s) have thiamine phosphate phosphatase activity and role in phosphate ion transport	-1.118671	0.002801428
ZRC1	Putative zinc transporter essential for tolerance to zinc; plays a role in zincosome formation; Hap43-induced; required for normal filamentous growth; mRNA binds She3 and is localized to hyphal tips	-1.119292	5.05896E-06
orf19.5773	Putative dipeptidyl-peptidase III; protein detected by mass spec in exponential and stationary phase cultures; Hog1p-induced; clade-associated gene expression	-1.121801	2.21004E-09
PTC4	Type PP2C serine/threonine phosphatase; localized to mitochondria; mutation causes sensitivity to sodium, potassium and azole drugs; decreased expression in hyphae compared to yeast-form cells	-1.123715	0.000100818
orf19.5730	Putative phenylacrylic acid decarboxylase; clade-associated gene expression	-1.124178	1.13773E-09
RCE1	Putative Type II CAAX prenyl protease; induced during the mating process	-1.125828	4.41289E-12
orf19.4901	Predicted methyltransferase; Spider biofilm induced	-1.12673	0.0072672

orf19.6596	Putative esterase; possibly transcriptionally regulated by Tac1; induced by Mnl1 under weak acid stress; protein present in exponential and stationary growth phase yeast cultures; Spider biofilm repressed	-1.127151	8.818E-14
CDC53	Cullin, a scaffold subunit of the SCF ubiquitin-ligase complexes; depletion leads to increased filamentous growth and premature cell death	-1.130045	8.75604E-08
USO1	Ortholog(s) have role in Golgi vesicle docking, SNARE complex assembly, endoplasmic reticulum to Golgi vesicle-mediated transport and ER to Golgi transport vesicle membrane, Golgi membrane localization	-1.133062	0.017063953
orf19.4523	Ortholog(s) have 5-formyltetrahydrofolate cyclo-ligase activity and role in folic acid-containing compound biosynthetic process	-1.133165	0.000632593
orf19.719	Ortholog(s) have ubiquitin protein ligase activity, ubiquitin-protein transferase activity	-1.133878	0.000904998
ASG1	Gal4p family zinc-finger transcription factor with similarity to S. cerevisiae Asg1p	-1.136659	1.56205E-05
orf19.5826	Predicted amino acid transmembrane transporter; rat catheter biofilm repressed	-1.13801	8.63697E-06
COX19	Putative cytochrome c oxidase assembly protein; Plc1-regulated; rat catheter biofilm induced	-1.139246	0.006601998
CSH3	Functional homolog of S. cerevisiae Shr3p, which is a chaperone specific for amino acid permeases; localized to ER; required for wild-type amino-acid responsive hyphal growth and for mouse systemic virulence; regulated by Gcn2p and Gcn4p	-1.142721	5.58887E-12
ZCF13	Predicted Zn(II)2Cys6 transcription factor; similar to but not the true ortholog of S. cerevisiae Hap1; mutants display decreased colonization of mouse kidneys	-1.14439	0.000204251
SMC3	Protein similar to S. cerevisiae Smc3p, which is an ATPase involved in sister chromatid cohesion; likely to be essential for growth, based on an insertional mutagenesis strategy	-1.145677	0.016594606
VPS15	Protein involved in retrograde endosome-to-Golgi protein transport; required for normal virulence	-1.147226	0.017908659
orf19.3689	Putative protein similar to 6-phosphofructo-2-kinase/fructose-2,6-bisphosphatase; expression downregulated in an ssr1 null mutant	-1.147629	1.0178E-08
orf19.3763	Ortholog of C. dubliniensis CD36 : Cd36_11710, C. auris B8441 : B9J08_003469, Candida tenuis NRRL Y-1498 : CANTEDRAFT_112512 and Debaryomyces hansenii CBS767 : DEHA2E16808g	-1.149957	0.000735478
orf19.1855	Predicted membrane transporter, member of the anion:cation symporter (ACS) family, major facilitator superfamily (MFS);	-1.152003	0.006869335

	Gcn4p-regulated; flucytosine induced; ketoconazole-repressed; oxidative stress-induced via Cap1p		
TPS1	Trehalose-6-phosphate synthase; role in hyphal growth and virulence in mouse systemic infection; induced in presence of human neutrophils; macrophage/pseudohyphal-repressed after 16h; stationary phase enriched protein; Hap43-repressed	-1.152084	2.15135E-08
SSU72	Ortholog(s) have RNA polymerase II CTD heptapeptide repeat phosphatase activity, phosphoprotein phosphatase activity, protein serine/threonine phosphatase activity, protein tyrosine phosphatase activity	-1.152463	7.69403E-05
orf19.247	Ortholog(s) have role in endosome organization, regulation of protein localization and BLOC-1 complex localization	-1.15317	0.00140603
orf19.2263	Ortholog of C. dubliniensis CD36 : Cd36_21360, C. parapsilosis CDC317 : CPAR2_406560, C. auris B8441 : B9J08_002799 and Candida tenuis NRRL Y-1498 : CANTEDRAFT_103408	-1.15846	3.95382E-05
GLO1	Putative monomeric glyoxalase I; oxidative stress-induced via Cap1; flow model and rat catheter biofilm repressed	-1.160367	4.49762E-11
orf19.4793	Putative ribosome-associated protein; ortholog of S. cerevisiae Tma16; Hap43-induced gene; Spider biofilm induced	-1.165818	0.01222172
orf19.4898	Putative protein of unknown function; induced by prostaglandins	-1.166134	6.98271E-19
orf19.5516	Ortholog(s) have role in SRP-dependent cotranslational protein targeting to membrane and signal recognition particle, endoplasmic reticulum targeting localization	-1.166812	3.03203E-07
orf19.2008	Ortholog(s) have S-methyl-5-thioribose-1-phosphate isomerase activity and role in L-methionine salvage from methylthioadenosine	-1.168881	5.19384E-10
OST1	Alpha subunit of the oligosaccharyltransferase complex of the ER lumen; catalyzes asparagine-linked glycosylation of newly synthesized proteins; Spider biofilm repressed	-1.170907	9.22322E-15
orf19.4428	Ortholog of C. dubliniensis CD36 : Cd36_06910, C. parapsilosis CDC317 : CPAR2_208560, C. auris B8441 : B9J08_001617 and Candida tenuis NRRL Y-1498 : CANTEDRAFT_95854	-1.173276	0.004565529
orf19.3679	Putative protein of unknown function; stationary phase enriched protein	-1.17579	5.21453E-07
orf19.1300	Putative mitochondrial membrane protein; homozygous transposon insertion causes decreased colony wrinkling under filamentous growth-inducing conditions, but does not block true hyphal formation in liquid media	-1.179765	9.07472E-07
CPA1	Putative carbamoyl-phosphate synthase subunit; alkaline repressed; rat catheter, Spider and flow model biofilm induced	-1.180514	1.04522E-06
orf19.1534	Ortholog of S. cerevisiae Zrt3, vacuolar membrane zinc transporter; predicted Kex2 substrate; induced in	-1.181946	0.000297107

	oralpharyngeal candidasis; flow model biofilm induced; Spider biofilm induced		
ALP1	Cystine transporter; present in pathogenic yeasts (no human or murine homolog); Spider biofilm induced	-1.182085	4.8411E-09
PHHB	Putative 4a-hydroxytetrahydrobiopterin dehydratase; transposon mutation affects filamentous growth; flow model biofilm induced; Spider biofilm induced	-1.18243	6.98281E-06
orf19.5295	Protein with a predicted endonuclease/exonuclease/phosphatase family domain and a carbon catabolite repressor protein 4 domain; induced by alpha pheromone in SpiderM medium	-1.18884	0.007750029
HEM4	Putative uroporphyrinogen III synthase; induced in high iron or elevated CO2; alkaline, Hap43-repressed	-1.189063	8.7299E-05
orf19.1160	Ortholog(s) have COPII receptor activity and role in endoplasmic reticulum to Golgi vesicle-mediated transport, fungal-type cell wall organization, protein retention in Golgi apparatus	-1.189922	1.78594E-16
orf19.6807	Predicted ORF in retrotransposon Tca17 with similarity to parts of the Gag-Pol region of retrotransposons; clade-associated gene expression	-1.190753	4.87631E-10
orf19.4511	Ortholog of Candida albicans WO-1 : CAWG_04197	-1.190997	0.001633001
orf19.2515	ZZ-type zinc finger protein; rat catheter and Spider biofilm induced	-1.192645	6.25008E-05
PEP1	Type I transmembrane sorting receptor for multiple vacuolar hydrolases; cycles between late-Golgi and prevacuolar endosome-like compartments; rat catheter biofilm repressed	-1.194333	1.02704E-05
orf19.2890	Has domain(s) with predicted carbohydrate:proton symporter activity, pyrimidine nucleotide-sugar transmembrane transporter activity and role in carbohydrate transport, pyrimidine nucleotide-sugar transmembrane transport	-1.195551	2.14052E-06
orf19.4580	Protein of unknown function; Hap43-repressed gene	-1.196464	3.7321E-07
COX15	Cytochrome oxidase assembly protein; transcript regulated by Nrg1 and Tup1; alkaline repressed; Hap43-repressed; early-stage flow model biofilm induced; Spider biofilm repressed	-1.197587	3.02035E-08
PUP3	Putative beta 3 subunit of the 20S proteasome; macrophage/pseudohyphal-repressed	-1.198355	7.63138E-13
orf19.4626	Ortholog(s) have role in TOR signaling, positive regulation of transcription by RNA polymerase I and cytosol, extrinsic component of membrane localization	-1.19864	1.31923E-05
orf19.6990	Putative peptidyl-prolyl cis-trans isomerase; predicted endoplasmic reticulum (ER) localization	-1.198799	1.34075E-05
orf19.7306	Aldo-keto reductase; increased transcript associated with MDR1 overexpression, benomyl or long-term fluconazole treatment; overexpression does not affect drug or oxidative	-1.199897	4.01257E-13

	stress sensitivity; stationary phase enriched; flow biofim repressed		
RAV2	Protein similar to S. cerevisiae Rav2; a regulator of (H+)-ATPase in vacuolar membrane; transposon mutation affects filamentous growth	-1.204489	3.01747E-06
orf19.5626	Protein of unknown function; Plc1-regulated; induced by Mnl1 under weak acid stress; flow model biofilm induced	-1.204569	2.2742E-06
LYS1	Saccharopine dehydrogenase (biosynthetic); enzyme of alpha-aminoadipate lysine biosynthesis pathway; functionally complements S. cerevisiae lys1 mutation; fungal-specific (no human or murine homolog)	-1.209424	1.34781E-10
SNZ1	Stationary phase protein; vitamin B synthesis; induced byyeast-hypha switch, 3-AT or in azole-resistant strain overexpressing MDR1; soluble in hyphae; regulated by Gcn4, macrophage; Spider biofilm induced; rat catheter biofilm repressed	-1.211926	8.18403E-16
orf19.3220	Putative rRNA processing protein; Spider biofilm induced	-1.213803	4.66826E-05
OPT7	Putative oligopeptide transporter; possibly transports GSH or related compounds; Hog1-induced; expression of OPT6, -7, or -8 does not suppress defect of mutant lacking OPT1-3; Hap43-repressed; F-12/CO2 early biofilm induced	-1.214803	7.55331E-10
EBP1	NADPH oxidoreductase; interacts with phenolic substrates (17beta-estradiol); possible role in estrogen response; induced by oxidative, weak acid stress, NO, benomyl, GlcNAc; Cap1, Mnl1 induced; Hap43-repressed; rat catheter biofilm induced	-1.216281	5.57743E-16
orf19.3442	Putative oxidoreductase; Hap43-repressed gene	-1.218061	2.12892E-13
RDH54	Putative DNA-dependent ATPase with a predicted role in DNA recombination and repair; transcriptionally induced by interaction with macrophages	-1.218997	1.09132E-09
SNL1	Ribosome-associated protein predicted to function in protein synthesis; 1 predicted transmembrane domain; rat catheter biofilm repressed	-1.219015	2.25258E-09
IFA14	Putative LPF family protein; Plc1-regulated; induced by alpha pheromone in SpiderM medium	-1.220702	0.00059742
TAL1	Transaldolase; protein present in exponential and stationary growth phase yeast cultures; oxidative stress-induced via Cap1; induced by nitric oxide independent of Yhb1p; sumoylation target; rat catheter biofilm repressed	-1.221891	4.22175E-18
orf19.6867	Protein with a predicted cytochrome b5-like heme/steroid binding domain; repressed by alpha pheromone in SpiderM medium	-1.222091	3.21231E-13
orf19.1381	Ortholog of S. cerevisiae/S. pombe Lsb5; predicted role in actin cortical patch localization, actin filament organization,	-1.222392	6.46637E-11

	endocytosis; flow model biofilm induced; Spider biofilm repressed		
orf19.6077	Putative protein of unknown function; shows colony morphology-related gene regulation by Ssn6p	-1.223801	3.76722E-17
AMO2	Protein similar to A. niger predicted peroxisomal copper amino oxidase; mutation confers hypersensitivity to toxic ergosterol analog; F-12/CO2 early biofilm induced	-1.226716	3.65607E-06
orf19.3357	Ortholog(s) have structural constituent of ribosome activity and mitochondrial small ribosomal subunit, mitochondrion localization	-1.228222	2.66507E-06
orf19.3515	Putative 3-hydroxyanthranilic acid dioxygenase, involved in NAD biosynthesis; Hap43p-repressed gene	-1.228565	4.1576E-06
orf19.5502	Ortholog of C. dubliniensis CD36 : Cd36_73420, C. auris B8441 : B9J08_004732, Debaryomyces hansenii CBS767 : DEHA2A05456g and Pichia stipitis Pignal : PICST_30999	-1.229554	1.3748E-05
PGI1	Glucose-6-phosphate isomerase; enzyme of glycolysis; antigenic; Efg1-regulated; induced upon adherence to polystyrene; repressed by phagocytosis, human neutrophils; flow model biofilm induced; rat catheter and Spider biofilm repressed	-1.22993	1.03386E-14
MCP1	Mitochondrial outer membrane protein, component of vacuole and mitochondria patches (vCLAMPs); involved in mitophagy; mutants are defective in mitochondrial function and virulence; regulated by Sef1p, Sfu1p, and Hap43p	-1.230439	3.38295E-05
MRPL33	Putative mitochondrial ribosomal protein of the large subunit; Ssr1-repressed; rat catheter biofilm induced	-1.231068	0.00840413
orf19.1764	Protein of unknown function; rat catheter and Spider biofilm induced	-1.233074	3.35638E-06
orf19.3528	Protein of unknown function; Spider biofilm induced	-1.234152	3.31855E-07
orf19.6860	Ortholog(s) have CDP-diacylglycerol-inositol 3-phosphatidyltransferase activity, role in phosphatidylinositol biosynthetic process and Golgi apparatus, integral component of membrane, mitochondrial outer membrane localization	-1.234766	2.95044E-15
orf19.6522	Putative allantoate permease; Gcn4-regulated; Spider biofilm induced	-1.236405	0.001070843
ERG251	C-4 sterol methyl oxidase; role in ergosterol biosynthesis; Hap43-induced; ketoconazole-induced; amphotericin B, caspofungin repressed; possibly essential gene, disruptants not obtained by UAU1 method; Spider biofilm repressed	-1.239755	1.11327E-21
orf19.2231	Protein of unknown function; Spider biofilm induced	-1.241614	5.8542E-08
DUG3	Putative glutamine amidotransferase (GATase II); role in glutathione catabolism;	-1.243173	2.86888E-07
TFB3	Putative C3HC4 zinc finger transcription factor; transcript positively regulated by Tbf1; Spider biofilm induced	-1.250384	2.69115E-08

FRP2	Putative ferric reductase; alkaline induced by Rim101; fluconazole-downregulated; upregulated in the presence of human neutrophils; possibly adherence-induced; regulated by Sef1, Sfu1, and Hap43	-1.251279	6.39222E-11
orf19.7329	Ortholog(s) have ubiquitin conjugating enzyme activity, ubiquitin-protein transferase activity	-1.253377	8.33919E-22
SLA2	Actin binding protein with roles in growth control and morphogenesis; required for alkaline pH-induced hyphal formation; localized to actin patches; rat catheter biofilm repressed	-1.253481	6.39931E-07
orf19.1096	Has domain(s) with predicted ion channel activity, voltage-gated chloride channel activity, role in chloride transport, transmembrane transport and membrane localization	-1.254606	1.24072E-05
orf19.5038	Predicted tRNA (guanine) methyltransferase activity; Spider biofilm induced	-1.257024	8.34355E-05
orf19.1785	Protein with a PI31 proteasome regulator domain; Hap43-repressed; flow model biofilm induced	-1.259991	5.521E-16
CBP1	Corticosteroid binding protein; transcription induced at late log-phase or upon adherence to polystyrene; not induced by corticosterone; contains a possible NAD/FAD binding region; regulated by Nrg1, Tup1; Spider biofilm induced	-1.261614	6.31363E-18
RPS42	Predicted ribosomal protein S4, component of the small ribosomal subunit; has paralog RPS41	-1.263505	2.83346E-19
CYB5	Cytochrome b(5); ortholog of S. cerevisiae Cyb5; induced in high iron; fluconazole-induced; shows colony morphology-related gene regulation by Ssn6; mutants are viable	-1.267976	2.32994E-15
ALK8	Alkane-inducible cytochrome P450; catalyzes hydroxylation of lauric acid to hydroxylauric acid; overproduction causes fluconazole resistance in WT and causes multidrug resistance in a cdr1 cdr2 double mutant; rat catheter biofilm repressed	-1.269416	0.004131749
VRP1	Verprolin-related protein involved in actin cytoskeleton organization and polarized morphogenesis; interacts with Wal1p and Myo5p; downregulated upon adherence to polystyrene	-1.270264	1.17309E-13
orf19.6048	Protein of unknown function; Spider biofilm induced	-1.270615	3.76856E-06
CDC19	Pyruvate kinase at yeast cell surface; Gcn4/Hog1/GlcNAc regulated; Hap43/polystyrene adherence induced; repressed by phagocytosis/farnesol; hyphal growth role; stationary phase enriched; flow model biofilm induced; Spider biofilm repressed	-1.275375	4.35002E-17
RAD6	Functional ortholog of S. cerevisiae Rad6; negative regulator of hyphal growth; required for wild-type UV resistance; acts in pathway with Efg1; ; transcript is UV-induced, slightly repressed during hyphal growth; gene has two introns	-1.280731	3.29727E-14

RPS3	Ribosomal protein S3; Hog1, Hap43-induced; grepressed upon phagocytosis by murine macrophage; present in exponential and stationary phase cells; Spider biofilm repressed	-1.28261	6.04216E-18
orf19.6921	Ortholog(s) have RNA polymerase I general transcription initiation factor activity and role in chromatin organization, nucleolar large rRNA transcription by RNA polymerase I	-1.282765	0.007681501
RTF1	Putative RNA polymerase II-associated Paf1 complex subunit; induced during the mating process	-1.283836	8.2049E-06
orf19.1267.1	Ortholog(s) have cysteine desulfurase activity, role in iron-sulfur cluster assembly and L-cysteine desulfurase complex, extrinsic component of mitochondrial inner membrane, mitochondrial matrix localization	-1.284286	0.002875076
orf19.7445	Ortholog of S.c. Vid24; a peripheral membrane protein located at Vid (vacuole import and degradation) vesicles; regulated by Sef1, Sfu1, and Hap43; Spider biofilm induced	-1.28482	6.74895E-05
AGM1	Phosphoacetylglucosamine mutase (N-acetylglucosamine-phosphate mutase); enzyme of UDP-N-acetylglucosamine (UDP-GlcNAc) biosynthesis	-1.284853	3.04517E-12
orf19.640	Integral membrane protein of the ER; role in the synthesis of beta-1,6-glucan in the cell wall; S. cerevisiae ortholog required for cell viability; Spider biofilm induced	-1.287144	5.61565E-05
ATG9	Protein similar to S. cerevisiae Atg9; required for early step in autophagy; required for cytoplasm to vacuole trafficking of Lap41; Spider biofilm induced	-1.287882	0.00141231
orf19.5428	Putative Golgi membrane protein with a predicted role in manganese homeostasis; Hap43p-repressed gene; clade-associated gene expression	-1.291071	2.34621E-07
orf19.850	Ortholog(s) have protein-N-terminal asparagine amidohydrolase activity, protein-N-terminal glutamine amidohydrolase activity and role in N-terminal protein amino acid modification, protein catabolic process	-1.293145	0.015622245
GCN2	Translation initiation factor 2-alpha (eIF2alpha) kinase; has nonessential role in amino acid starvation response, in contrast to S. cerevisiae homolog; similar to S. cerevisiae Gcn2p	-1.294743	0.015752773
EMP70	Protein with a role in endosome-to-vacuole sorting; rat catheter biofilm repressed	-1.295003	5.93852E-09
CKA2	Catalytic alpha-subunit of protein kinase CK2; interaction with calcineurin pathway affects fluconazole sensitivity; synthetically lethal with CKA1; attenuated virulence in a mouse oropharyngeal candidiasis but not in a systemic mouse model	-1.295731	5.45973E-08
GNA1	Glucosamine-6-phosphate acetyltransferase; enzyme of UDP-GlcNAc biosynthesis; required for viability in absence of	-1.300245	4.18797E-06

	GlcNAc supplementation; required for persistent infection and wild-type virulence in mouse systemic infection		
orf19.3458	Ortholog(s) have role in late endosome to vacuole transport via multivesicular body sorting pathway and Vps55/Vps68 complex, fungal-type vacuole membrane localization	-1.300477	1.32373E-10
orf19.3051	Protein of unknown function; S. pombe ortholog SPAC17A2.02c plays a role in resistance to cadmium; colony morphology-related gene regulation by Ssn6; Spider biofilm repressed	-1.300685	9.88252E-19
ENO1	Enolase, involved in glycolysis and gluconeogenesis; also has transglutaminase activity involved in assembly of cell wall polysaccharides; major cell-surface antigen; binds host plasmin/plasminogen; immunoprotective; may be essential	-1.301077	1.75043E-24
RBP1	Peptidyl-prolyl cis-trans isomerase; rapamycin-binding protein; homozygous null mutation confers rapamycin resistance; regulated by Gcn4p; macrophage-induced protein; repressed in response to 3-AT; functional homolog of S. cerevisiae Rbp1p	-1.301343	7.38547E-15
orf19.3411	Ortholog of S. cerevisiae : BUD17, C. glabrata CBS138 : CAGL0M10725g, C. dubliniensis CD36 : Cd36_61840, C. parapsilosis CDC317 : CPAR2_602630 and C. auris B8441 : B9J08_005378	-1.301818	7.67797E-06
CPR3	Putative peptidyl-prolyl cis-trans isomerase; macrophage-induced protein; protein levels decrease in stationary phase yeast cultures; predicted mitochondrial localization; overlaps orf19.1551	-1.303197	3.07447E-15
RNR21	Ribonucleoside-diphosphate reductase; regulated by tyrosol and cell density; ciclopirox olamine, fluconazole or flucytosine induced; regulated by Sef1, Sfu1, and Hap43	-1.303387	4.05486E-26
FGR17	Putative DNA-binding transcription factor; has zinc cluster DNA-binding motif; lacks an ortholog in S. cerevisiae; transposon mutation affects filamentous growth; Hap43p-repressed gene	-1.305534	0.008350939
APN2	Putative class II abasic (AP) endonuclease; flucytosine induced	-1.31013	3.18023E-05
CDR4	Putative ABC transporter superfamily; fluconazole, Sfu1, Hog1, core stress response induced; caspofungin repressed; fluconazole resistance not affected by mutation or correlated with expression; rat catheter and flow model biofilm induced	-1.311461	2.69173E-10
orf19.5239	Predicted alanine-tRNA ligase; oxidative stress-induced via Cap1	-1.313617	9.22676E-11
orf19.5426	Putative patatin-like phospholipase; macrophage-regulated gene; fungal-specific (no human or murine homolog)	-1.313728	0.002432394
ACS1	Acetyl-CoA synthetase; induced by human neutrophils; fluconazole-repressed; regulated by Nrg1/Mig1; colony	-1.318707	4.52121E-23

	morphology-related gene regulation by Ssn6; only in stationary phase cultures; rat catheter biofilm repressed, Spider biofilm induced		
orf19.6518	Predicted aldehyde dehydrogenase [NAD(P)+]; Spider biofilm induced	-1.321711	2.0908E-07
orf19.3508	Putative protein of unknown function; stationary phase enriched protein	-1.323965	7.36909E-10
PNC1	Putative nicotinamidase, involved in NAD salvage pathway; decreased transcription is observed in an azole-resistant strain that overexpresses MDR1	-1.331757	1.36381E-06
orf19.2650.1	Mitochondrial ribosomal protein of the small subunit; Spider biofilm repressed	-1.332525	0.002621429
CDC3	Septin; essential for viability; functional homolog of S. cerevisiae Cdc3p; down-regulation associated with azole resistance; macrophage/pseudohyphal-repressed; virulence-group-correlated expression; gene has intron	-1.333817	2.09621E-15
orf19.4621	Ortholog(s) have P-body localization	-1.335085	1.70803E-05
orf19.5757	Ortholog(s) have FAD diphosphatase activity and role in flavin-containing compound metabolic process	-1.335753	6.62392E-10
orf19.5727	Ortholog of C. dubliniensis CD36 : Cd36_64130, C. parapsilosis CDC317 : CPAR2_601230, C. auris B8441 : B9J08_003898 and Candida tenuis NRRL Y-1498 : CANTEDRAFT_115908	-1.337951	1.05477E-06
ARF3	Similar to but not orthologous to S. cerevisae Arf3; transcript filament induced; Tup1 regulated; rat catheter biofilm repressed (see Locus History Note for Assembly 19 correction)	-1.338551	1.04164E-10
PFY1	Profilin; functional homolog of S. cerevisiae Pfy1; hyphae, macrophage/pseudohyphal-induced; regulated by Nrg1, Tup1; gene lacks intron (unlike S. cerevisiae PFY1); complements growth of S. cerevisiae srv2 mutant; nonessential	-1.339506	2.57797E-18
orf19.1106	Protein with Mob2p-dependent hyphal regulation; fluconazole-induced	-1.339576	0.00563449
orf19.7531	Protein of unknown function; stationary phase enriched protein; induced upon yeast-hypha transition; benomyl or caspofungin induced; Hap43-repressed; Spider biofilm induced	-1.340858	8.69143E-16
PRE7	Subunit of the 20S core particle of the proteasome	-1.340912	9.04561E-14
orf19.3482	Ortholog(s) have NAD+ diphosphatase activity, role in NADH metabolic process and peroxisome localization	-1.341839	6.98681E-14
HOL4	Putative ion transporter; alkaline induced by Rim101; Plc1-regulated; caspofungin repressed; rat catheter and Spider biofilm induced	-1.342835	4.17246E-13
AVT4	Putative vacuolar transporter of large neutral amino acids; induced by alpha pheromone in SpiderM medium	-1.346909	0.005641465

PDR16	Phosphatidylinositol transfer protein; induction correlates with CDR1, CDR2 overexpression/azole resistance; fluphenazine, 17-beta-estradiol, ethynyl estradiol, NO induced; farnesol-downregulated in biofilm; rat catheter biofilm induced	-1.347169	2.09436E-12
orf19.2278	Putative 20S proteasome assembly protein; filament induced; induced by alpha pheromone in SpiderM medium	-1.34751	9.80761E-15
FRP3	Putative ammonium transporter; upregulated in the presence of human neutrophils; fluconazole-downregulated; repressed by nitric oxide; Spider biofilm induced; rat catheter biofilm repressed	-1.352714	1.34662E-13
orf19.836.1	Ortholog(s) have U1 snRNP, U2 snRNP, U2-type prespliceosome, U4/U6 x U5 tri-snRNP complex, U5 snRNP, post-mRNA release spliceosomal complex localization	-1.354767	0.025238745
orf19.6898.1	Ortholog of C. dubliniensis CD36 : Cd36_71020, C. parapsilosis CDC317 : CPAR2_300360, C. auris B8441 : B9J08_000715 and Candida tenuis NRRL Y-1498 : CANTEDRAFT_105022	-1.358767	0.000285834
orf19.5777	Protein of unknown function; F-12/CO2 early biofilm induced	-1.361694	5.26384E-07
AMS1	Putative alpha-mannosidase; transcript regulated by Nrg1; induced during cell wall regeneration; flow model biofilm induced; Spider biofilm induced	-1.363568	0.000797967
orf19.7589	Protein of unknown function; Hap43-repressed gene; transcript induced by elevated CO2	-1.364582	7.26728E-09
orf19.6554	Regulator of calcineurin; regulated by calcineurin-Crz1 pathway; feedback regulator of calcineurin-dependent signaling; Hap43-repressed; induced by ketoconazole, hypoxia, during growth in the mouse cecum; Spider biofilm induced	-1.364847	7.64441E-17
MCD4	Mannose-ethanolamine phosphotransferase, essential gene involved in GPI anchor biosynthesis	-1.370866	1.12699E-05
orf19.4666	Protein of unknown function; hyphal-induced expression, regulated by Cyr1, Ras1, Efg1; Spider biofilm induced	-1.371594	1.09953E-07
ARP9	Component of the RSC chromatin remodeling complex; similar to S. cerevisiae Arp3p, a component of the Arp2/3 complex involved in actin-dependent processes; likely to be essential for growth, based on an insertional mutagenesis strategy	-1.371939	3.73319E-11
VHT1	Predicted membrane transporter, involved in biotin import; member of the anion:cation symporter (ACS) family, major facilitator superfamily (MFS); biotin-dependent transcription regulated by Vhr1p; amphotericin B, caspofungin repressed	-1.372839	0.002099318
orf19.7288	Protein with predicted oxidoreductase and dehydrogenase domains; Hap43-repressed; Spider biofilm induced	-1.372944	3.27832E-06

CLN3	G1 cyclin; depletion abolishes budding and causes hyphal growth defects; farnesol regulated, functional in S. cerevisiae; other biofilm induced; Spider biofilm induced	-1.374165	6.15614E-08
orf19.6266	Protein of unknown function; Spider biofilm induced	-1.374204	1.11556E-05
ECM1	Putative pre-ribosomal factor; decreased mRNA abundance observed in cyr1 homozygous mutant hyphae; induced by heavy metal (cadmium) stress; Hog1p regulated	-1.377937	0.018690424
CHS7	Protein required for wild-type chitin synthase III activity; similar to (but not functional homolog of) S. cerevisiae Chs7p, which effects ER export of Chs3p; induced cyr1 mutant hyphae and ras1 yeast-form cells; Spider biofilm induced	-1.378069	1.02142E-16
orf19.6986	Has domain(s) with predicted intracellular anatomical structure localization	-1.378389	1.98585E-05
THI13	Thiamin pyrimidine synthase; synthesis of the thiamine precursor hydroxymethylpyrimidine phosphate; single-turnover enzyme that provides histidine for HMP-P formation; induced by nitric oxide independent of Yhb1; Spider biofilm induced	-1.379628	6.08158E-05
GVP36	BAR domain protein; ocalizes to early and late Golgi vesicles; predicted role in adaptation to varying nutrient concentrations, fluid-phase endocytosis, actin cytoskeleton polarization and vacuole biogenesis; rat catheter biofilm repressed	-1.381621	1.53175E-14
TKL1	Putative transketolase; localizes to surface of yeast cells, not hyphae; soluble protein in hyphae; transcript regulated by Nrg1, Mig1, and Tup1; antigenic in human or murine infection; possibly essential (by UAU1 method)	-1.383066	1.31934E-17
orf19.3360	Protein of unknown function; flow model biofilm induced; Spider biofilm induced	-1.384409	7.31962E-06
orf19.2763	Protein not essential for viability; orf19.10279 possibly transcriptionally regulated upon hyphal formation	-1.384785	3.40725E-05
orf19.2397.3	Putative aminotransferase; Hap43-repressed; homozygous Tn insertion decreases colony wrinkling in filament-inducing conditions, does not block true hypha formation in liquid media; rat catheter and Spider biofilm induced	-1.386587	2.0317E-07
orf19.6608	Predicted DDE superfamily endonuclease domain; repression correlates with clinical development of fluconazole resistance; Spider biofilm induced	-1.387476	2.43143E-07
MED10	RNA polymerase II mediator complex subunit; possibly an essential gene, disruptants not obtained by UAU1 method	-1.38773	0.020991082
orf19.5720	Predicted membrane transporter, member of the monocarboxylate porter (MCP) family, major facilitator superfamily (MFS); ketoconazole or caspofungin repressed; Spider biofilm induced	-1.388161	2.01739E-12

YCF1	Putative glutathione S-conjugate transporter; MRP/CFTR-subfamily, ABC type transporter; human neutrophil-induced; oxidative stress-induced via Cap1; possible association with multidrug resistance; possibly essential; Spider biofilm induced	-1.38863	7.16271E-10
orf19.3228	Putative endosomal transmembrane protein; Hap43p-induced; mutation confers hypersensitivity to amphotericin B	-1.388667	1.84419E-07
orf19.5279	Ortholog(s) have structural constituent of ribosome activity and role in cellular respiration, regulation of mitochondrial DNA metabolic process, response to oxidative stress	-1.388979	5.2022E-06
FGR24	Protein encoded in retrotransposon Zorro2 with similarity to retroviral endonuclease-reverse transcriptase proteins; lacks an ortholog in S. cerevisiae; transposon mutation affects filamentous growth	-1.389748	1.17059E-06
orf19.6916	Ortholog(s) have unfolded protein binding activity, role in mitochondrial proton-transporting ATP synthase complex assembly, mitochondrion organization and mitochondrion localization	-1.390422	3.01563E-05
BGL22	Putative glucanase; induced during cell wall regeneration	-1.392957	1.03537E-08
IFG3	Putative D-amino acid oxidase; Spider biofilm induced	-1.393741	5.6063E-09
orf19.419	Protein of unknown function; flow model biofilm induced; Spider biofilm induced	-1.393857	0.000298091
REX2	Putative 3'-5' RNA exonuclease with a predicted role in 3'-end processing of U4 and U5 snRNAs, 5S and 5.8S rRNAs; rat catheter biofilm induced	-1.396088	3.50732E-08
SEC14	Essential protein; functional homolog of S. cerevisiae Sec14p, a Golgi phosphatidylinositol/phosphatidylcholine transfer protein that regulates choline-phosphate cytidyltransferase and thereby affects secretion; biofilm-regulated	-1.3965	7.71743E-13
BMT1	Beta-mannosyltransferase, required for addition of the 1st beta-mannose residue to acid-stable fraction of cell wall phosphopeptidomannan; 9-gene family member; mutants induce higher levels of inflammatory cytokines in mouse dendritic cells	-1.39758	3.35678E-07
DBF2	Essential serine/threonine protein kinase involved in mitotic spindle formation and cytokinesis; required for septum formation, exit from mitosis, and normal hyphal morphogenesis; virulence-group-correlated expression	-1.398845	0.00017421
orf19.3158	Ortholog of S. cerevisiae : RMD1, C. glabrata CBS138 : CAGL0M10483g, C. dubliniensis CD36 : Cd36_81090, C. parapsilosis CDC317 : CPAR2_101950 and C. auris B8441 : B9J08_002355	-1.400521	3.31887E-08
ERV29	Putative SURF4 family member; plasma membrane-localized; flow model biofilm repressed	-1.401189	5.91559E-15

MAL31	Putative high-affinity maltose transporter; transcript is upregulated in clinical isolates from HIV+ patients with oral candidiasis; alkaline induced; Spider biofilm induced	-1.403124	0.023466976
orf19.1549	Plasma membrane-associated protein identified in detergent-resistant membrane fraction (possible lipid raft component); repressed by nitric oxide; predicted transmembrane helix	-1.405918	7.16446E-16
orf19.5576	Putative pantothenate kinase; ortholog of S. cerevisiae Cab1; transposon mutation affects filamentous growth; repressed in core stress response	-1.40776	7.83671E-12
orf19.7615	Protein involved in endoplasmic reticulum (ER) to Golgi vesicle-mediated transport; putative subunit of the transport protein particle (TRAPP) complex of the cis-Golgi; Spider biofilm induced	-1.41177	2.26564E-06
orf19.2888	Ortholog(s) have role in cellular protein-containing complex assembly, early endosome to Golgi transport and Golgi apparatus, TRAPPII protein complex localization	-1.412803	3.18469E-06
TRP99	Putative thioredoxin peroxidase/alkyl hydroperoxide reductase; induced in low iron; regulated by Gcn4; induced in response to amino acid starvation (3-AT treatment)	-1.419784	1.94652E-24
BIO2	Putative biotin synthase; induced by high iron; repressed by ciclopirox olamine; upregulated in clinical isolates from HIV+ patients with oral candidiasis; Spider biofilm induced; biotin-dependent transcription regulated by Vhr1p	-1.421839	1.46907E-09
orf19.6007	Predicted fatty acid acyl transferase-related protein domain; repressed by prostaglandins	-1.425272	9.96262E-17
STF2	Protein involved in ATP biosynthesis; repressed in hyphae; repressed by Efg1, Hap43; transcript upregulated in clinical isolates from HIV+ patients with oral candidiasis; rat catheter, flow model and Spider biofilm induced	-1.425685	3.53081E-17
orf19.4341	Ortholog(s) have role in attachment of GPI anchor to protein and GPI-anchor transamidase complex localization	-1.42625	2.26888E-10
DAP1	Similar to mammalian membrane-associated progesterone receptors involved in DNA damage response; induced in core stress response; Hog1 regulated; clade-associated expression; Hap43-repressed	-1.426678	1.91816E-19
orf19.6656	Spermidine transporter; induced in strains from HIV patients with oral candidiasis; alkaline repressed; amphotericin B induced; colony morphology regulated by Ssn6; reduced oral epithelial cell damage by mutant; Spider biofilm induced	-1.426685	6.80243E-11
YRB1	Functional homolog of S. cerevisiae Yrb1p; regulates Gsp1 GTPase activity and thereby affects nucleocytoplasmic transport and cytoskeletal dynamics; transcript is not regulated by white-opaque switch or by dimorphic transition	-1.426777	9.80361E-20
TVP18	Putative integral membrane protein; fluconazole-induced	-1.426915	1.67868E-12

HIS7	Putative imidazole glycerol phosphate synthase; histidine biosynthesis; no human/murine homolog; transcription induced by histidine starvation; regulated by Gcn2p and Gcn4p; higher protein level in stationary phase	-1.43069	1.33614E-16
ALG6	Putative glucosyltransferase involved in cell wall mannan biosynthesis; transcription is elevated in chk1, nik1, and sln1 homozygous null mutants; repressed by nitric oxide; possibly essential gene, disruptants not obtained by UAU1 method	-1.43318	3.42888E-09
orf19.4570	Ortholog of C. dubliniensis CD36 : Cd36_42090, Debaryomyces hansenii CBS767 : DEHA2C14872g, Pichia stipitis Pignal : PICST_74821 and Candida tropicalis NEW ASSEMBLY : CTRG1_00174	-1.433805	0.000101698
orf19.349	Ortholog(s) have role in aerobic respiration, mRNA metabolic process	-1.435747	1.6648E-06
AGO1	Putative Argonaute protein involved in RNA silencing; hyphal-induced expression; regulated by Cyr1, Ras1, Efg1, Nrg1, Tup1; rat catheter biofilm repressed	-1.438035	6.51856E-09
NUP84	Ortholog(s) have structural constituent of nuclear pore activity	-1.4387	5.39292E-10
orf19.994	Protein of unknown function; induced by Mnl1 under weak acid stress; flow model biofilm repressed	-1.438953	0.000219242
orf19.5449	Predicted integral membrane protein; Spider biofilm induced	-1.439517	2.08922E-13
GST3	Glutathione S-transferase; expression regulated upon white-opaque switch; induced by human neutrophils; peroxide-induced; induced by alpha pheromone in SpiderM medium; Spider biofilm induced	-1.440086	1.45652E-08
orf19.7277	Predicted ORF in retrotransposon Zorro2 with similarity to zinc finger-containing retroviral nucleocapsid proteins; un-merged from orf19.7275 in a revision of Assembly 21 based on comparative genome analysis and new sequence data	-1.440536	4.93911E-07
WH11	White-phase yeast transcript; expression in opaques increases virulence/switching; mutant switches as WT; Hap43, hypoxia, ketoconazol induced; required for RPMI biofilm; Bcr1-induced in RPMI a/a biofilm; rat catheter, Spider biofilm induced	-1.443245	4.5361E-11
CSH1	Aldo-keto reductase; role in fibronectin adhesion, cell surface hydrophobicity; regulated by temperature, growth phase, benomyl, macrophage interaction; azole resistance associated; Spider biofilm induced; rat catheter biofilm repressed	-1.443416	3.48315E-17
GAP4	High-affinity S-adenosylmethionine permease; required for SAM-induced morphogenesis; hyphal induced; regulated by Hap43, Gcn2 and Gcn4; colony morphology-related gene regulation by Ssnp	-1.444087	3.5476E-28
orf19.4539	Protein with a Rho GDP-dissociation inhibitor domain; Hap43-repressed gene; Spider biofilm induced	-1.445613	7.28621E-11

orf19.7449	Ortholog(s) have role in mitochondrial genome maintenance, plasmid maintenance	-1.446457	0.003392675
MED5	RNA polymerase II mediator complex subunit; transcription positively regulated by Tbf1p	-1.447801	2.07905E-06
orf19.4756	Ortholog of S. cerevisiae : YTP1, C. dubliniensis CD36 : Cd36_08490, C. parapsilosis CDC317 : CPAR2_801590, C. auris B8441 : B9J08_004547 and Candida tenuis NRRL Y-1498 : CANTEDRAFT_109732	-1.448252	7.7074E-06
TUB1	Alpha-tubulin; gene has intron; complements cold-sensitivity of S. cerevisiae tub1 mutant; C. albicans has single alpha-tubulin gene, whereas S. cerevisiae has two (TUB1, TUB3); farnesol-upregulated in biofilm; sumoylation target	-1.450246	1.80076E-17
PCD1	Ortholog(s) have 8-oxo-7,8-dihydroguanosine triphosphate pyrophosphatase activity, pyrophosphatase activity, role in DNA repair and peroxisome localization	-1.466608	3.80244E-09
PRB1	Endoprotease B; regulated by heat, carbon source (GlcNAc-induced), nitrogen, macrophage response, human neutrophils; similar to (does not replace) S. cerevisiae vacuolar B protease Prb1p; flow model biofilm induced; Spider biofilm induced	-1.4674	2.6211E-11
orf19.6585	Ortholog(s) have role in protein maturation by [4Fe-4S] cluster transfer and mitochondrial matrix localization	-1.467516	0.011823283
orf19.3615	Protein of unknown function; induced in core caspofungin response; expression upregulated in an ssr1 null mutant; induced by nitric oxide independent of Yhb1p	-1.469485	1.40145E-09
orf19.3053	Protein of unknown function; present in exponential and stationary phase yeast; identified in extracts from biofilm and planktonic cells; flow model biofilm induced gene; GlcNAc-induced protein	-1.471417	1.25116E-17
GRE2	Putative reductase; Nrg1 and Tup1-regulated; benomyl- and hyphal-induced; macrophage/pseudohyphal-repressed; repressed by low iron; possibly involved in osmotic stress response; stationary phase enriched protein; Spider biofilm induced	-1.471463	2.54077E-28
MON2	Peripheral membrane protein; role in endocytosis and vacuole integrity; flow model and rat catheter biofilm repressed	-1.472472	1.59517E-06
DSE1	Essential cell wall protein involved in cell wall integrity and rigidity; periodic mRNA expression peaks at M/G1 phase; Ace2p-induced; required for virulence in a mouse model of infection	-1.473043	0.000172661
ULP2	SUMO deconjugation enzyme; removes the small ubiquitin-like modifier (SUMO) from proteins; Spider biofilm induced	-1.47465	1.82531E-06
orf19.1564	Plasma membrane-localized protein of unknown function	-1.475672	2.7163E-21
orf19.4965	Protein of unknown function; rat catheter biofilm induced	-1.475792	0.0057625

orf19.1158	Ortholog of S. cerevisiae Yft2 required for normal ER membrane biosynthesis; Hap43-repressed gene	-1.476254	4.4056E-06
orf19.3613	Ortholog(s) have RNA polymerase II C-terminal domain phosphoserine binding, RNA polymerase II complex binding, chromatin binding activity	-1.477726	3.22136E-10
GEA2	Putative ARF GTP/GDP exchange factor; induced in low iron; flow model biofilm repressed	-1.482832	4.76367E-07
orf19.1183	Ortholog of S. cerevisiae : YNL115C, C. glabrata CBS138 : CAGL0L03938g, C. dubliniensis CD36 : Cd36_60200, C. parapsilosis CDC317 : CPAR2_603320 and C. auris B8441 : B9J08_002166	-1.484079	3.76013E-16
orf19.2006	Ortholog of S. cerevisiae : YPL162C, C. glabrata CBS138 : CAGL0M02057g, C. dubliniensis CD36 : Cd36_16070, C. parapsilosis CDC317 : CPAR2_213850 and C. auris B8441 : B9J08_003194	-1.484506	4.17441E-05
orf19.2237.1	Ortholog of Slx9 required for pre-rRNA processing; associated with 90S pre-ribosome and 43S small ribosomal subunit precursor, interacts with U3 snoRNA in S. cerevisiae; gene has intron; Spider biofilm induced	-1.485013	0.005782386
PGA49	Putative GPI-anchored protein	-1.485496	2.58544E-08
PRE10	Alpha7 (C8) subunit of the 20S proteasome; multiple phosphorylated residues; transcript induced upon filamentous growth; Spider biofilm repressed	-1.488253	1.63697E-23
LAC1	Ceramide synthase; required for biosynthesis of ceramides with C18:0 fatty acids, which serve as precursors for glucosylsphingolipids; caspofungin induced	-1.490975	7.79483E-08
orf19.6581	Ortholog(s) have palmitoyltransferase activity, role in protein palmitoylation and endoplasmic reticulum localization	-1.492041	5.72161E-07
orf19.413	Protein of unknown function; induced by Sfu1; Spider biofilm induced	-1.492239	0.003518048
PEX6	Ortholog(s) have ATPase activity, role in fatty acid metabolic process, protein import into peroxisome matrix, receptor recycling, protein targeting to peroxisome, protein unfolding and cytosol, peroxisome localization	-1.492252	0.0001548
orf19.5469	Protein with a predicted DEAD-like DNA/RNA helicase domain; shows colony morphology-related gene regulation by Ssn6; overlaps orf19.5472; Spider biofilm repressed	-1.493331	2.39029E-22
orf19.7131	Butyrobetaine dioxygenase, the fourth enzyme of the carnitine biosynthesis pathway	-1.49335	1.67147E-24
TPI1	Triose-phosphate isomerase; antigenic in mouse/human; mutation affects filamentation; macrophage-repressed; protein in exponential and stationary growth phase yeast; possibly essential; flow model biofilm induced; Spider biofilm repressed	-1.494039	3.68238E-23

orf19.5616	Ortholog of C. dubliniensis CD36 : Cd36_63740, C. parapsilosis CDC317 : CPAR2_503850, Pichia stipitis Pignal : PICST_32045 and Candida guilliermondii ATCC 6260 : PGUG_00070	-1.494109	0.00696121
ILV6	Putative regulatory subunit of acetolacetate synthase; alkaline induced; regulated by Gcn2 and Gcn4; protein present in exponential and stationary growth phase yeast; Spider biofilm repressed	-1.494673	1.56255E-10
ADH1	Alcohol dehydrogenase; oxidizes ethanol to acetaldehyde; at yeast cell surface; immunogenic in humans/mice; complements S. cerevisiae adh1 adh2 adh3 mutant; fluconazole, farnesol-induced; flow model biofilm induced; Spider biofilm repressed	-1.495794	4.18541E-20
TRX1	Thioredoxin; involved in response to reactive oxygen species; biofilm, benomyl, flucytosine, peroxide, Hap43 induced; amphotericin B, caspofungin repressed; induced by human neutrophils; macrophage-repressed gene	-1.496558	1.70012E-23
RHD1	Putative beta-mannosyltransferase required for the addition of beta-mannose to the acid-labile fraction of cell wall phosphopeptidomannan; 9-gene family memebr; regulated on yeast-hypha and white-opaque switches; Spider biofilm repressed	-1.498673	1.09826E-05
orf19.2751	Predicted membrane transporter; member of the monocarboxylate porter (MCP) family, major facilitator superfamily; Hap43-induced gene; alkaline upregulated by Rim101; possibly an essential gene, disruptants not obtained by UAU1 method	-1.504558	9.39822E-08
orf19.5921	Ortholog of C. dubliniensis CD36 : Cd36_84580, C. parapsilosis CDC317 : CPAR2_404290, C. auris B8441 : B9J08_003750 and Candida tenuis NRRL Y-1498 : CANTEDRAFT_115338	-1.5064	2.51644E-06
orf19.516	S. cerevisiae ortholog Rft1p has role in glycolipid translocation, protein N-linked glycosylation and localizes to endoplasmic reticulum membrane; regulated by Sef1p-, Sfu1p-, and Hap43p	-1.509843	4.4503E-07
MDH1-3	Predicted malate dehydrogenase; farnesol regulated; protein present in exponential and stationary growth phase yeast; Hap43p-repressed gene	-1.510181	1.10634E-26
VPH2	Protein required for for proper vacuolar ATPase assembly and vacuolar functions	-1.512983	5.08357E-05
orf19.1994	Ortholog(s) have phosphatidylinositol binding, phosphatidylinositol-3-phosphate binding activity, role in retrograde transport, endosome to Golgi and endosome localization	-1.513159	3.70541E-11
orf19.1624	Non-catalytic subunit of N-terminal acetyltransferase of the NatC type; flow model biofilm repressed	-1.514105	4.41132E-09

orf19.7365	Ortholog(s) have ubiquitin-protein transferase activity and role in negative regulation of apoptotic process, negative regulation of gluconeogenesis, proteasome-mediated ubiquitin-dependent protein catabolic process	-1.514857	7.4181E-06
SST2	Predicted regulator of G-protein signaling in mating pathway; null mutation causes alpha-factor hypersensitivity and mating defect (in opaque MTLa/MTLa background); transcript induced by alpha factor	-1.515302	0.016685356
orf19.3712	Protein of unknown function; induced by Mnl1 under weak acid stress; flow model biofilm induced; Spider biofilm induced	-1.515835	1.36065E-12
DRE2	Putative cytosolic Fe-S protein assembly protein; a-specific transcript; regulated by Sef1, Sfu1, and Hap43; rat catheter and Spider biofilm induced	-1.517129	3.62555E-08
orf19.1305	Ortholog(s) have tRNA (guanine-N1-)-methyltransferase activity, role in mitochondrial tRNA methylation, tRNA N1-guanine methylation and cytoplasm, mitochondrial matrix localization	-1.51807	8.74916E-12
orf19.5293	Ortholog of C. dubliniensis CD36 : Cd36_43710, C. parapsilosis CDC317 : CPAR2_402940, C. auris B8441 : B9J08_005206 and Candida tenuis NRRL Y-1498 : CANTEDRAFT_114940	-1.523781	1.83496E-15
APL5	Ortholog of S. cerevisiae and S. pombe Apl5; subunit of the AP-3 adaptor complex involved in Golgi-to-vacuole transport; phosphorylated protein; mutant is viable;	-1.524191	3.76479E-05
ARE2	Acyl CoA:sterol acyltransferase; uses cholesterol and oleoyl-CoA substrates; protoberberine derivative drug inhibits enzyme activity; ketoconazole-induced; Hap43-repressed; flow model biofilm induced; Spider biofilm induced	-1.526584	0.000490637
orf19.951	Protein of unknown function; transcript repressed upon yeast-hyphal switch; fluconazole-induced; Hap43-repressed; flow model biofilm induced	-1.527329	2.44249E-10
orf19.2335	Putative aspartyl aminopeptidase; stationary phase enriched protein; mutation confers hypersensitivity to 5-fluorouracil (5-FU); Hog1-induced; planktonic growth-induced; rat catheter and Spider biofilm repressed	-1.532574	9.23844E-16
orf19.3310	Protein of unknown function; Hap43-repressed; rat catheter and Spider biofilm induced	-1.537682	0.00672262
HGT18	Putative glucose transporter of the major facilitator superfamily; the C. albicans glucose transporter family comprises 20 members; 12 probable membrane-spanning segments; expressed in rich medium with 2% glucose	-1.538714	7.67519E-24
orf19.6117	S. pombe ortholog SPAC5D6.04 is a predicted auxin family transmembrane transporter; ketoconazole and hypoxia induced	-1.539174	8.58382E-22
orf19.94	Protein of unknown function; Spider biofilm induced	-1.541993	1.03308E-09

PHO114	Acid phosphatase; induced by Mnl1 under weak acid stress; Spider biofilm induced	-1.542452	0.0056276
orf19.2455	Ortholog(s) have glutathione hydrolase activity, omega peptidase activity, peptidase activity, role in glutathione catabolic process and cytoplasm, glutathione hydrolase complex, nuclear periphery localization	-1.542681	1.13104E-05
CBR1	Putative cytochrome B5 reductase; plasma membrane-localized	-1.547146	1.64284E-20
RIM9	Protein required for alkaline pH response via the Rim101 signaling pathway; ortholog of S. cerevisiae Rim9 and A. nidulans palI; Spider biofilm induced	-1.549961	0.000107118
orf19.7547	Ortholog(s) have phosphatidylinositol-3-phosphate binding, ubiquitin protein ligase activity, ubiquitin-protein transferase activity	-1.552488	2.00489E-18
orf19.5586	Ortholog(s) have phosphatidylinositol-3,5-bisphosphate 5-phosphatase activity, role in phosphatidylinositol dephosphorylation and PAS complex, extrinsic component of membrane, fungal-type vacuole membrane, nuclear periphery localization	-1.552541	0.012494704
orf19.649	Ortholog of C. dubliniensis CD36 : Cd36_30530, C. parapsilosis CDC317 : CPAR2_203340, C. auris B8441 : B9J08_000376 and Candida tenuis NRRL Y-1498 : CANTEDRAFT_117008	-1.553876	7.65262E-07
orf19.1777	Ortholog(s) have deubiquitinase activity, endopeptidase activity, thiol-dependent deubiquitinase activity	-1.556362	8.2618E-10
orf19.2446	Has domain(s) with predicted 2-dehydropantoate 2-reductase activity, NADP binding, oxidoreductase activity, oxidoreductase activity, acting on the CH-OH group of donors, NAD or NADP as acceptor activity	-1.559388	6.76979E-14
orf19.7196	Putative vacuolar protease; upregulated in the presence of human neutrophils; Spider biofilm induced	-1.559844	1.78807E-32
ADH2	Alcohol dehydrogenase; soluble in hyphae; expression regulated by white-opaque switching; regulated by Ssn6; indued by Mnl1 in weak acid stress; protein enriched in stationary phase yeast cultures; Spider biofilm induced	-1.561658	1.35226E-08
orf19.1406	Ortholog(s) have DNA-directed DNA polymerase activity, role in error-free translesion synthesis, error-prone translesion synthesis and mitochondrion, zeta DNA polymerase complex localization	-1.562387	6.18186E-19
NIT2	Putative carbon-nitrogen hydrolase; rat catheter biofilm repressed	-1.5642	0.000324785
orf19.7310	Protein with a role in directing meiotic recombination events to homologous chromatids; induced by ciclopirox olamine; positively regulated by Sfu1; Hog1, fluconazole-repressed; Hap43-induced; Spider biofilm induced	-1.56666	6.87232E-09

GCA1	Extracellular/plasma membrane-associated glucoamylase; expressed in rat oral infection; regulated by carbohydrates, pH, galactose; promotes biofilm matrix formation; flow model biofilm induced; Bcr1 repressed in RPMI a/a biofilms	-1.567989	0.001238586
ABG1	Vacuolar membrane protein; depletion causes abnormal vacuolar morphology, cell separation defect, sensitivity to cell wall stress, increased hyphal branching; essential, no mammalian homolog; Cyr1-regulated; rat catheter biofilm repressed	-1.569509	1.45119E-16
ERG6	Delta(24)-sterol C-methyltransferase, converts zymosterol to fecosterol, ergosterol biosynthesis; mutation confers nystatin resistance; Hap43, GlcNAc-, fluconazole-induced; upregulated in azole-resistant strain; Spider biofilm repressed	-1.569784	3.1799E-21
TUB2	Beta-tubulin; functional homolog of ScTub2; overproduction makes S. cerevisiae inviable; has two introns; GlcNAc, hypha fluconazole-induced; slow growth, ectopic expression increases white-to opaque switch; rat catheter biofilm repressed	-1.571438	1.7592E-18
RIC1	Ortholog of S. cerevisiae Ric1 guanyl-nucleotide exchange factor; mutant is viable; rat catheter biofilm repressed	-1.572495	9.01647E-06
HGT3	Putative glucose transporter of the major facilitator superfamily; the C. albicans glucose transporter family comprises 20 members; 12 probable membrane-spanning segments, extended C terminus; expressed in rich medium with 2% glucose	-1.573467	0.000241045
orf19.6869	Putative lipid raft associated protein; Spider biofilm induced	-1.573911	1.0266E-09
RAS1	RAS signal transduction GTPase; regulates cAMP and MAP kinase pathways; role in hyphal induction, virulence, apoptosis, heat-shock sensitivity; nonessential; plasma membrane-localized; complements viability of S. cerevisiae ras1 ras2 mutant	-1.576999	9.41176E-15
orf19.6627	Protein of unknown function; possibly transcriptionally regulated upon hyphal formation	-1.578112	3.96728E-15
CAN3	Predicted amino acid transmembrane transporter; transcript regulated by white-opaque switch; Hap43-repressed gene	-1.578337	8.33308E-21
PXP2	Putative acyl-CoA oxidase; enzyme of fatty acid beta-oxidation; induced during macrophage infection; opaque specific transcript; putative peroxisome targeting signal; Spider biofilm induced	-1.579609	0.003102278
RPC19	Putative RNA polymerases I and III subunit AC19; Hap43-induced; rat catheter biofilm induced	-1.579912	2.80934E-05
HOS1	Histone deacetylase; similar to S. cerevisiae Hos1; has conserved deacetylation motif; slightly greater expression in white cells than in opaque cells	-1.582341	3.06219E-05

orf19.6861	Ortholog(s) have ubiquitin protein ligase activity, role in anaphase-promoting complex-dependent catabolic process, chromatin assembly, protein ubiquitination and anaphase-promoting complex localization	-1.587369	1.85741E-07
ARC19	Putative ARP2/3 complex subunit; Hap43-induced gene; mutation confers hypersensitivity to cytochalasin D; rat catheter biofilm repressed	-1.588373	2.56296E-10
RRD1	Putative peptidyl-prolyl cis/trans-isomerase; caspofungin induced	-1.589032	2.52927E-08
orf19.5054	Putative quinolinate phosphoribosyl transferase, involved in NAD biosynthesis; Hap43p-repressed gene	-1.589165	1.61328E-25
ZCF27	Putative Zn(II)2Cys6 transcription factor	-1.593022	3.87632E-05
orf19.4842	Protein of unknown function; Spider biofilm induced	-1.594706	0.003453731
orf19.2642	Ortholog(s) have myosin II heavy chain binding activity, role in mitotic actomyosin contractile ring disassembly and cellular bud neck contractile ring, incipient cellular bud site, myosin II complex localization	-1.594761	0.002291491
orf19.3982	Maltase; induced during growth on sucrose; induced by alpha pheromone in SpiderM medium; early-stage flow model biofilm indced	-1.596661	1.27476E-05
ASR2	Adenylyl cyclase and stress responsive protein; induced in cyr1 or ras1 mutant; stationary phase enriched protein; Spider biofilm induced	-1.599364	6.78186E-08
orf19.6458	Ortholog of C. dubliniensis CD36 : Cd36_72260, C. parapsilosis CDC317 : CPAR2_702840, Candida tenuis NRRL Y-1498 : CANTEDRAFT_115664 and Debaryomyces hansenii CBS767 : DEHA2B05940g	-1.599462	0.016055009
IHD2	Protein of unknown function; induced during hyphae development; induced in low iron; Spider biofilm induced	-1.600621	1.78942E-05
orf19.21	Ortholog(s) have role in ethanol metabolic process and mitochondrial inner membrane localization	-1.600819	3.43848E-06
orf19.4629	Ortholog(s) have ubiquitin-ubiquitin ligase activity, role in ATP export, mitochondrion inheritance, protein monoubiquitination, protein polyubiquitination, ubiquitin-dependent endocytosis and ubiquitin ligase complex localizatio	-1.601451	0.01106458
orf19.1002	Protein of unknown function; Hap43-repressed gene	-1.605414	0.009854199
ATO2	Putative fungal-specific transmembrane protein; fluconazole repressed, Hap43-repressed; flow model biofilm induced; Spider biofilm induced	-1.606362	2.33101E-24
orf19.6356	Ortholog(s) have role in mRNA splicing, via spliceosome and U4/U6 snRNP, U4/U6 x U5 tri-snRNP complex localization	-1.607056	0.000320146

RAM1	Protein that acts in prenylation; transcription is alpha-factor induced; regulated in response to lovastatin and fluconazole; Hap43p-repressed gene	-1.607889	2.94768E-05
orf19.5278	Protein of unknown function; Spider biofilm induced	-1.609396	7.47954E-13
orf19.4550	Predicted MFS membrane transporter, member of the drug:proton antiporter (12 spanner) (DHA1) family; flow model biofilm induced	-1.609436	6.31624E-12
GYP7	Protein similar to S. cerevisiae Gyp7p (GTPase-activating protein for Ypt1p); caspofungin-induced	-1.611371	1.00646E-14
DIP5	Dicarboxylic amino acid permease; mutation confers hypersensitivity to toxic ergosterol analog; induced upon phagocytosis by macrophage; Gcn4-regulated; upregulated by Rim101 at pH 8; rat catheter and Spider biofilm induced	-1.611761	2.22141E-11
orf19.4780	Predicted MFS family membrane transporter, member of the drug:proton antiporter (12 spanner) (DHA1) family; Spider biofilm induced	-1.613813	9.12975E-12
orf19.1483	Ortholog(s) have copper ion binding activity, role in mitochondrial cytochrome c oxidase assembly and extrinsic component of mitochondrial inner membrane, mitochondrial intermembrane space localization	-1.617947	6.56322E-06
FGR38	Protein lacking an ortholog in S. cerevisiae; member of a family of related proteins; transposon mutation affects filamentous growth; macrophage-induced gene; Hap43p-repressed gene	-1.617978	6.37002E-05
orf19.6709	Predicted alpha/beta hydrolase; Spider biofilm induced	-1.622731	1.24423E-14
LTP1	Putative protein phosphatase of the PTP family (tyrosine-specific), similar to S. cerevisiae Ltp1p	-1.624862	9.72613E-18
orf19.5633	F-box domain-containing protein; flow model biofilm induced	-1.625157	4.40884E-20
orf19.7210	Protein of unknown function; Spider biofilm induced	-1.625166	1.30484E-16
FEN12	Putative protein with a predicted role in the elongation of fatty acids; amphotericin B, caspofungin repressed	-1.625571	5.22914E-19
orf19.3456	Protein with a predicted serine/threonine kinase and tyrosine kinase domain; possibly an essential gene, disruptants not obtained by UAU1 method	-1.625787	0.000396822
orf19.729	Adaptor protein required for specific mRNA transport; protein similar but not orthologous to S. cerevisiae She3; transposon mutation affects filamentous growth	-1.626015	1.61211E-10
FRP1	Ferric reductase; alkaline-induced by Rim101; iron-chelation-induced by CCAAT-binding factor; fluconazole-repressed; ciclopirox-, hypoxia-, Hap43-induced; colony morphology-related regulation by Ssn6; Spider and flow model biofilm induced	-1.626903	2.1644E-10

orf19.2794	Putative non-specific single-domain racemase; regulated by Gcn4p; repressed in response to amino acid starvation (3-AT treatment); alkaline upregulated; macrophage-induced protein	-1.628282	2.21796E-14
RPN6	Putative 26S proteasome subunit; Hap43p-induced gene; regulated by Gcn2p and Gcn4p	-1.629137	5.23668E-19
MAE1	Malic enzyme, mitochondrial; transcription regulated by Mig1, Tup1; colony morphology-related gene regulation by Ssn6; Hap43-repressed; Spider biofilm repressed	-1.630562	2.21193E-14
RBK1	Has domain(s) with predicted ribokinase activity and role in D-ribose metabolic process	-1.63309	1.64546E-19
VPS11	Protein involved in protein trafficking; putative role in vesicle-target membrane fusion; mutant lacks vacuole; role in hyphal growth, possibly via vacuole expansion into hypha; role in killing of and survival within macrophage	-1.635015	5.08835E-05
RRP42	Putative exosome non-catalytic core component; involved in 3'-5' RNA processing; rat catheter biofilm induced	-1.635351	3.11583E-08
orf19.3223.1	Putative 12kDa subunit of mitochondrial NADH-ubiquinone oxidoreductase; gene has intron	-1.635929	0.000960031
orf19.261	Ortholog(s) have dolichol kinase activity, role in dolichyl monophosphate biosynthetic process and endoplasmic reticulum membrane localization	-1.635969	6.84803E-07
PGA7	GPI-linked hyphal surface antigen; induced by ciclopirox olamine, ketoconazole, Rim101 at pH 8; Hap43, fluconazole; flow model biofilm induced; Spider biofilm induced; required for RPMI biofilm; Bcr1-induced in a/a biofilm	-1.638366	2.86E-15
TSC2	Putative GTPase-activating protein; similar to mammalian tuberin; involved in control of filamentous growth; mutants are viable	-1.639531	0.000233524
ALI1	Putative NADH-ubiquinone oxidoreductase; in detergent-resistant membrane fraction (possible lipid raft component); predicted N-terminal acetylation; nitric oxide-repressed; plasma membrane-localized; protein decreases in stationary phase	-1.64131	1.23569E-17
orf19.4904	Ortholog(s) have nucleosome binding activity, role in chromatin remodeling, histone exchange and Swr1 complex, cytoplasm, nuclear periphery localization	-1.643521	1.53965E-09
orf19.73	Putative metalloprotease; associates with ribosomes and is involved in ribosome biogenesis; Spider biofilm induced	-1.644381	1.58805E-10
SLD1	Sphingolipid delta-8 desaturase; catalyzes desaturation at C8 in the long-chain base moiety of ceramides in glucosylceramide synthesis, important for virulence; ketoconazole and hypoxia induced; Hap43-repressed; Spider biofilm induced	-1.645623	3.62377E-12

orf19.3364	Ortholog of C. parapsilosis CDC317 : CPAR2_403360, Debaryomyces hansenii CBS767 : DEHA2D00814g, Pichia stipitis Pignal : PICST_32156 and Candida guilliermondii ATCC 6260 : PGUG_04611	-1.646732	1.3253E-08
ALK2	N-Alkane inducible cytochrome P450	-1.648506	3.72527E-09
SMD2	Putative Core Sm protein; Hap43p-induced gene; flucytosine induced	-1.649073	0.003555099
YAK1	Predicted serine-threonine protein kinase; involved in hyphal growth regulation and biofilm formation; flow model biofilm induced; induced in core caspofungin response	-1.650316	2.32497E-07
orf19.1210	Ortholog(s) have L-arginine transmembrane transporter activity, L-aspartate transmembrane transporter activity and L-glutamate transmembrane transporter activity, more	-1.650721	8.6637E-19
LAP41	Putative aminopeptidase yscI precursor; mutant is viable; protein present in exponential and stationary growth phase yeast cultures; Spider biofilm repressed	-1.651911	4.53169E-19
OSM2	Putative mitochondrial fumarate reductase; regulated by Ssn6p, Gcn2p, and Gcn4p; Hog1p-downregulated; stationary phase enriched protein; Hap43p-repressed gene	-1.653623	3.48016E-13
OPT2	Oligopeptide transporter; induced upon phagocytosis by macrophage; macrophage/pseudohyphal-repressed after 16h; fluconazole-induced; virulence-group-correlated expression; Hap43-repressed	-1.655089	0.01177799
CAM1-1	Putative translation elongation factor; downregulated upon phagocytosis by murine macrophages; Hap43-induced gene; Spider biofilm repressed	-1.657674	1.26399E-22
orf19.4471	Ortholog(s) have role in TOR signaling, re-entry into mitotic cell cycle after pheromone arrest and endoplasmic reticulum, endoplasmic reticulum membrane, endoplasmic reticulum-Golgi intermediate compartment localization	-1.661402	7.55204E-05
CST5	Scaffold protein for the mitogen-activated protein (MAP) kinase cascade that regulates mating; required for opaque mating or white biofilm formation in response to mating pheromone; induced in response to pheromone; Hap43p-repressed	-1.662517	5.5155E-11
orf19.4264	Protein of unknown function; induced during chlamydospore formation in both C. albicans and C. dubliniensis; flow model biofilm induced	-1.664636	2.25943E-07
YKT6	Putative protein of the vacuolar SNARE complex; predicted role in vacuolar fusion; rat catheter biofilm repressed	-1.666759	3.96854E-16
HMX1	Heme oxygenase; utilization of hemin iron; transcript induced by heat, low iron, or hemin; repressed by Efg1; induced by low iron; upregulated by Rim101 at pH 8; Hap43-induced; Spider and flow model biofilm induced	-1.667291	6.86368E-05

HGT5	Putative glucose transporter of the major facilitator superfamily; the C. albicans glucose transporter family comprises 20 members; 12 probable membrane-spanning segments, extended N terminus; expressed in rich medium; Hap43p-repressed	-1.672296	7.71584E-08
HGT2	Putative MFS glucose transporter; 20 member C. albicans glucose transporter family; 12 probable membrane-spanning segments; expressed in rich medium with 2% glucose; rat catheter and Spider biofilm induced	-1.673239	8.88152E-10
orf19.698	Integral ER membrane protein; predicted role in maintenance of ER zinc homeostasis; Spider biofilm induced	-1.680415	2.91139E-17
orf19.641	Ortholog(s) have role in protein folding and endoplasmic reticulum localization	-1.681077	1.9841E-12
orf19.899	Ortholog of C. dubliniensis CD36 : Cd36_18040, C. parapsilosis CDC317 : CPAR2_211790, C. auris B8441 : B9J08_004373 and Candida tenuis NRRL Y-1498 : CANTEDRAFT_109928	-1.681824	5.53265E-09
orf19.2038	Protein of unknown function; Spider biofilm induced	-1.68223	0.005440534
GPM1	Phosphoglycerate mutase; surface protein that binds host complement Factor H and FHL-1; antigenic; fluconazole, or amino acid starvation (3-AT) induced, farnesol-repressed; Hap43, flow model biofilm induced; Spider biofilm repressed	-1.68271	1.92875E-43
orf19.1761	Predicted olichyl-diphosphooligosaccharide-protein glycotransferase; role in protein N-linked glycosylation; Spider biofilm repressed	-1.684033	2.29317E-11
orf19.2933	Ortholog(s) have thiol-dependent deubiquitinase activity and role in protein deubiquitination, regulation of transcription, DNA-templated	-1.684658	2.63539E-12
orf19.3480	Ortholog(s) have structural constituent of ribosome activity and mitochondrial small ribosomal subunit localization	-1.686392	2.26022E-15
orf19.1595	Ortholog(s) have clathrin binding activity, role in clathrin-dependent endocytosis, histone H2B ubiquitination, sterol regulatory element binding protein cleavage and Dsc E3 ubiquitin ligase complex, clathrin-coated vesicle localization	-1.687779	5.10272E-12
orf19.1082	Protein with an Alba DNA/RNA-binding protein domain; Spider biofilm induced	-1.689342	0.001528052
orf19.3226	Ortholog(s) have sterol binding activity, role in intracellular sterol transport, sterol transport and extracellular region, fungal-type vacuole lumen localization	-1.692032	8.15903E-18
orf19.4174	Ortholog(s) have nicotinamide riboside transmembrane transporter activity, nucleobase transmembrane transporter activity, nucleoside transmembrane transporter activity	-1.693172	1.57976E-28
FOX2	3-hydroxyacyl-CoA epimerase; fatty acid beta-oxidation; induced by phagocytosis; regulated by Mig1, by white-opaque switch, by DNA methylation; transcriptional activation	-1.693426	1.46491E-10

	by oleate requires Ctf1; rat catheter and Spider biofilm induced		
KRE62	Putative subunit of glucan synthase; macrophage-induced gene; Bcr1-regulated in a/a RPMI biofilms	-1.696384	0.007272747
SAM51	Component of the SAM complex involved in mitochondrial protein import, involved in beta-barrel protein assembly; member of the Omp85 protein family	-1.699933	4.86039E-19
PIL1	Eisosome component; predicted role in endocytosis; echinocandin-binding protein; localizes to cell surface of hyphae, but not yeast-form cells; Hap43, YNB biofilm induced; rat catheter biofilm repressed	-1.70226	7.61752E-40
orf19.1477	Protein of unknown function; possible ER protein; Hap43p-repressed; Spider biofilm induced	-1.703194	1.80689E-21
orf19.4128	Ortholog of C. dubliniensis CD36 : Cd36_19390, C. parapsilosis CDC317 : CPAR2_209600, C. auris B8441 : B9J08_004606 and Candida tenuis NRRL Y-1498 : CANTEDRAFT_134010	-1.704114	1.53803E-07
orf19.5209	Ortholog(s) have role in cytoplasm to vacuole transport by the Cvt pathway, intra-Golgi vesicle-mediated transport and Golgi transport complex localization	-1.70581	1.33903E-06
SFT1	Putative Golgi v-SNARE; Plc1-regulated; Spider biofilm induced	-1.710734	0.0238342
orf19.1274	Has domain(s) with predicted DNA binding, zinc ion binding activity, role in transcription, DNA-templated and nucleus localization	-1.711468	0.000766777
XYL2	D-xylulose reductase; immunogenic in mice; soluble protein in hyphae; induced by caspofungin, fluconazole, Hog1 and during cell wall regeneration; Mnl1-induced in weak acid stress; stationary phase enriched; flow model biofilm induced	-1.712428	8.47235E-34
orf19.639	S. cerevisiae ortholog YDR370C/DXO1 has decapping and 5′-3′ exoRNase activity; decreased transcription is observed upon fluphenazine treatment or in an azole-resistant strain that overexpresses CDR1 and CDR2	-1.714301	4.46164E-07
orf19.3649	Ortholog(s) have adenyl-nucleotide exchange factor activity, role in cytoplasm protein quality control by the ubiquitin-proteasome system and cytosol localization	-1.716853	9.45254E-20
orf19.2686	Ortholog(s) have carboxypeptidase activity, role in nitrogen compound metabolic process, proteolysis involved in cellular protein catabolic process and fungal-type vacuole lumen localization	-1.717024	1.51208E-16
orf19.1258	Adhesin-like protein; regulated by Tsa1, Tsa1B in minimal media at 37 deg; clade-associated gene expression; induced by alpha pheromone in SpiderM medium; Hap43-induced; Spider biofilm repressed	-1.718325	3.88767E-13

HEM3	Hydroxymethylbilane synthase; converts 4-porphobilinogen to hydroxymethylbilane in heme biosynthesis; induced in high iron, CO2; alkaline repressed; regulated by Sef1, Sfu1, and Hap43; rat catheter and Spider biofilm induced	-1.722885	1.37574E-16
orf19.2708	Ortholog(s) have guanyl-nucleotide exchange factor activity and role in mature ribosome assembly	-1.723428	6.90279E-11
FMP27	Putative mitochondrial protein; mRNA binds She3	-1.72379	0.026885633
LYS22	Homocitrate synthase, minor isoform; repressed by nitric oxide and by hypoxia; protein level decreases in stationary phase cultures; induced by ketoconazole, Spider biofilm induced; flow model biofilm repressed	-1.724282	2.24817E-27
FGR22	Putative phosphatidylinositol-specific phospholipase C (PI-PLC); predicted type 2 membrane protein; no S. cerevisiae ortholog; role in, and regulated by, filamentation, Hap43p; almost identical to orf19.5797	-1.72431	0.016931718
orf19.7271	Protein of unknown function; transcript detected on high-resolution tiling arrays	-1.724452	6.94318E-24
orf19.3565	Ortholog of C. dubliniensis CD36 : Cd36_19830, C. parapsilosis CDC317 : CPAR2_206470, C. auris B8441 : B9J08_005009 and Candida tenuis NRRL Y-1498 : CANTEDRAFT_113899	-1.72476	0.001597018
HEM13	Coproporphyrinogen III oxidase; antigenic; on yeast cell surface, not hyphae; iron-regulated expression; Hap43, macrophage-repressed; farnesol-induced; possibly essential; flow model biofilm induced; rat catheter, Spider biofilm repressed	-1.727602	3.52769E-32
orf19.3216	Similar to Rab GTPase activators; Hap43p-induced gene	-1.730942	3.2054E-13
ERG25	Putative C-4 methyl sterol oxidase; C4-demethylation of ergosterol biosynthesis intermediates, based on similarity to S. cerevisiae Erg25; fluconazole-induced; induced in azole-resistant strain; rat catheter and Spider biofilm induced	-1.731929	8.68924E-19
orf19.1070	Ortholog(s) have role in phospholipid translocation, retrograde transport, endosome to Golgi and endosome membrane, trans-Golgi network membrane localization	-1.733287	2.1492E-07
orf19.7487	Has domain(s) with predicted RNA binding, RNA-directed DNA polymerase activity and role in RNA-dependent DNA biosynthetic process	-1.733937	1.01019E-37
VTI1	Ortholog(s) have SNAP receptor activity and role in Golgi to vacuole transport, intra-Golgi vesicle-mediated transport, macroautophagy, vacuole fusion, non-autophagic, vesicle fusion	-1.734447	2.72305E-17
PAD1	Putative phenylacrylic acid decarboxylase; repressed by Rgt1p	-1.736562	1.50247E-09
orf19.3204	Ortholog of C. dubliniensis CD36 : Cd36_51610, C. parapsilosis CDC317 : CPAR2_303630, C. auris B8441 : B9J08_001459 and Candida tenuis NRRL Y-1498 : CANTEDRAFT_135125	-1.736966	0.000995731

orf19.6035	Protein of unknown function; repressed by nitric oxide	-1.742083	8.73241E-07
orf19.1427	Putative transporter; fungal-specific; Spider biofilm induced	-1.745694	3.29773E-12
QDR1	Putative antibiotic resistance transporter; regulated by white-opaque switch, Nrg1, Tup1; Hap43, caspofungin repressed; repressed during chlamydospore formation; flow model biofilm induced; Spider biofilm repressed	-1.752373	1.00361E-14
orf19.6809	Putative phosphomutase-like protein; protein present in exponential and stationary growth phase yeast; Hap43-repressed; Spider biofilm repressed	-1.752657	7.4715E-22
orf19.1797	D-arabinose 5-phosphate isomerase; has GutQ domain which is associated with phosphosugar binding; other biofilm induced; rat catheter and Spider biofilm induced; F-12/CO2 early biofilm induced	-1.752811	3.71881E-18
NUE2	Mitochondrial protein required for expression of mitochondrial respiratory chain complex I (NADH:ubiquinone oxidoreductase)	-1.763323	4.29006E-06
orf19.1504	Putative patatin-like phospholipase; fungal-specific (no human or murine homolog)	-1.767755	4.36022E-10
orf19.2724	Protein of unknown function; flow model, rat catheter and Spider biofilm induced; Hap43-repressed	-1.767852	1.6905E-14
orf19.1087	Ortholog of C. dubliniensis CD36 : Cd36_64620, C. parapsilosis CDC317 : CPAR2_601130, Candida tropicalis MYA-3404 : CTRG_02757 and Candida albicans WO-1 : CAWG_04949	-1.770389	0.017274638
POL93	Predicted ORF in retrotransposon Tca8 with similarity to the Pol region of retrotransposons encoding reverse transcriptase, protease and integrase; downregulated in response to ciclopirox olamine; F-12/CO2 early biofilm induced	-1.773978	8.81118E-09
orf19.411	Protein similar to GTPase regulators; induced in low iron; transcript activated by Mnl1 under weak acid stress; Hap43-, Sfu1- and Sef1-regulated; flow model biofilm induced, Spider biofilm induced	-1.776096	4.12742E-09
orf19.2770	Ortholog of C. dubliniensis CD36 : Cd36_42220, C. parapsilosis CDC317 : CPAR2_500340, Debaryomyces hansenii CBS767 : DEHA2G17754g and Pichia stipitis Pignal : PICST_31424	-1.77657	7.36771E-05
orf19.733	Has domain(s) with predicted role in transmembrane transport and integral component of membrane localization	-1.777783	3.33105E-11
orf19.775	Ortholog of C. dubliniensis CD36 : Cd36_04450, C. parapsilosis CDC317 : CPAR2_105460, C. auris B8441 : B9J08_002463, Debaryomyces hansenii CBS767 : DEHA2D07128g and Pichia stipitis Pignal : PICST_80203	-1.779138	4.1371E-07
orf19.6703	Ortholog of C. dubliniensis CD36 : Cd36_73260 and Candida albicans WO-1 : CAWG_05699	-1.783013	1.26157E-06

ECI1	Protein similar to S. cerevisiae Eci1p, which is involved in fatty acid oxidation; transposon mutation affects filamentous growth; expression is regulated upon white-opaque switching	-1.783627	6.20089E-25
KTR2	Mannosyltransferase; transcription elevated in chk1, nik1, and sln1 homozygous null mutants; fungal-specific (no human or murine homolog); possibly an essential gene, disruptants not obtained by UAU1 method	-1.785885	2.05052E-14
PRC3	Putative carboxypeptidase Y precursor; transcript regulated by Nrg1 and Mig1; regulated by Gcn2 and Gcn4	-1.788524	5.61327E-16
orf19.5205	Protein of unknown function; Hap43-repressed gene	-1.788938	7.77454E-06
orf19.4984	Pseudogene; has lysine motifs associated with chitin binding	-1.789262	3.81224E-05
ATO1	Putative fungal-specific transmembrane protein; induced by Rgt1; Spider biofilm induced	-1.792235	3.72392E-07
orf19.7073	Ortholog of S. cerevisiae : YCL002C, C. dubliniensis CD36 : Cd36_70180, C. parapsilosis CDC317 : CPAR2_805360, C. auris B8441 : B9J08_000224 and Candida tenuis NRRL Y-1498 : cten_CGOB_00114	-1.792449	4.59783E-05
orf19.6023	Protein with a predicted multidrug transporter domain; Hap43-repressed gene	-1.795028	3.1598E-11
orf19.3376	Ortholog of Candida albicans WO-1 : CAWG_03459	-1.800341	0.005357923
orf19.1834	Has domain(s) with predicted role in mitotic sister chromatid cohesion	-1.804257	4.97448E-05
CWC22	Predicted spliceosome-associated protein; role in pre-mRNA splicing; Spider biofilm induced	-1.80449	0.000135919
RPB4	Protein similar to S. cerevisiae Rpb4p, which is a component of RNA polymerase II; transposon mutation affects filamentous growth	-1.804658	1.62373E-08
RPP1	Putative ortholog of S. cerevisiae Rpp1; subunit of both RNase MRP and nuclear RNase P; rat catheter and Spider biofilm induced	-1.805651	0.006986622
orf19.4340.1	Ortholog(s) have poly(U) RNA binding, splicing factor binding activity and U1 snRNP, U2 snRNP, U4/U6 x U5 tri-snRNP complex, U5 snRNP, post-mRNA release spliceosomal complex, spliceosomal complex localization	-1.807048	0.000248907
orf19.2452	Protein of unknown function; induced in high iron; repressed in core caspofungin response; ketoconazole-repressed; colony morphology-related gene regulation by Ssn6; possibly subject to Kex2 processing	-1.811665	5.83597E-14
PGA5	Putative GPI-anchored beta-1,3-glucanosyltransferase with similarity to the A. fumigatus GEL family; fungal-specific (no human or murine homolog)	-1.814598	0.00495285
orf19.1748	Protein of unknown function; shows colony morphology-related gene regulation by Ssn6	-1.814673	0.000123

FTH1	Protein similar to S. cerevisiae Fth1p, a high affinity iron transporter for intravacuolar stores of iron; repressed by Sfu1p, amphotericin B, caspofungin; induced by alkaline pH, ciclopirox olamine; regulated by Sef1p, Sfu1p, and Hap43p	-1.819206	0.00051593
orf19.1433	Protein of unknown function; Hap43-repressed; colony morphology-related gene regulation by Ssn6; Spider biofilm induced	-1.819931	4.83009E-16
orf19.6360	Protein involved in pre-mRNA splicing; Spider biofilm induced	-1.823413	2.84163E-08
FCY21	High affinity, high capacity, hypoxanthine-adenine-guanine-cytosine/H+ symporter; similar to S. cerevisiae Fcy2; mutation confers resistance to 5-fluorocytosine (5-FC); flow model biofilm induced	-1.824666	3.91822E-25
orf19.2438	Ortholog(s) have structural constituent of ribosome activity and mitochondrial small ribosomal subunit localization	-1.825143	4.69944E-11
orf19.4269	Has domain(s) with predicted ATP binding, protein kinase activity and role in protein phosphorylation	-1.826229	3.59006E-15
ALG8	Putative glucosyltransferase involved in cell wall mannan biosynthesis; transcription is elevated in nik1 and sln1 homozygous null mutants, but not in the chk1 null mutant; possibly an essential gene, disruptants not obtained by UAU1 method	-1.82748	7.48917E-14
MNN26	Putative alpha-1,2-mannosyltransferase; mutant sensitive to calcofluor, SDS and growth at 42 deg; present in detergent-resistant membrane fraction (possible lipid raft component); predicted N-terminal acetylation and transmembrane helix	-1.829629	1.31319E-10
orf19.1792	Ortholog(s) have ubiquitin protein ligase activity	-1.832195	5.83639E-10
orf19.1336.2	Ortholog(s) have role in mitochondrial respiratory chain complex assembly and mitochondrial intermembrane space localization	-1.832734	0.000682139
CDC28	Cyclin-dependent protein kinase; interacts with regulatory subunit Cyb1; determination of cell morphology during the cell cycle; phosphorylated mostly by Swe1 and phosphorylation is regulated by Hsl1; 5'-UTR intron; Spider biofilm repressed	-1.833764	1.38372E-17
orf19.7490	Predicted membrane transporter; fucose:proton symporter family member, MFS superfamily; flow model biofilm induced	-1.834556	5.6311E-08
orf19.4153	Ortholog(s) have NEDD8 activating enzyme activity and role in protein neddylation	-1.836334	3.98056E-09
FGR51	Protein lacking an ortholog in S. cerevisiae; transposon mutation affects filamentous growth; Hap43p-repressed gene	-1.837222	1.09567E-11
VRG4	GDP-mannose transporter; essential; required for glycosylation, hyphal growth; functional homolog of S. cerevisiae Vrg4p, which imports GDP-mannose from	-1.83835	5.39441E-24

	cytoplasm to Golgi for protein and lipid mannosylation; no mammalian homolog		
orf19.7305	Ortholog of C. dubliniensis CD36 : Cd36_34510, Candida tropicalis NEW ASSEMBLY : CTRG1_05938, Candida tropicalis MYA-3404 : CTRG_05938 and Candida albicans WO-1 : CAWG_02183	-1.839523	5.56633E-11
UGA2	Predicted succinate semialdehyde dehydrogenase; predicted role in glutamate catabolism; transcription regulated by Mig1, Tup1, Gcn4; mutants are viable	-1.841108	1.93464E-29
orf19.2124	Predicted alcohol dehydrogenase; Spider biofilm induced	-1.841975	1.47231E-21
orf19.6816	Putative xylose and arabinose reductase; flow model biofilm induced; Spider biofilm repressed	-1.84519	1.6266E-28
ESP1	Separase, caspase-like cysteine protease that cleaves cohesins to allow chromatid separation during anaphase; mutation confers increased sensitivity to nocodazole; periodic mRNA expression, peak at cell-cycle S/G2 phase	-1.849603	2.73646E-05
DOG1	Putative 2-deoxyglucose-6-phosphatase; haloacid dehalogenase hydrolase/phosphatase superfamily; similar to S. cerevisiae Dog1, Dog2, Hor1, Rhr2; regulated by Nrg1, Tup1; Spider biofilm repressed	-1.851702	4.47872E-20
CAN1	Basic amino acid permease; complements lysine transport mutation; 10 predicted transmembrane regions, 3 predicted N-glycosylation sites; phagocytosis by macrophages induces transcript; rat catheter, Spider and flow model biofilm induced	-1.852827	4.24143E-11
KTR4	Mannosyltransferase; induced during cell wall regeneration; fungal-specific (no human or murine homolog); Bcr1-repressed in RPMI a/a biofilms	-1.853478	2.67018E-14
orf19.3978	Protein required for maturation of 18S rRNA; rat catheter biofilm repressed	-1.854297	9.2266E-10
PRC2	Putative carboxypeptidase; induced by human neutrophils; Spider biofilm induced	-1.855541	3.67231E-16
orf19.1551	Predicted protein of unknown function; overlaps CPR3/orf19.1552	-1.856406	9.13622E-09
FCA1	Cytosine deaminase; enzyme of pyrimidine salvage; functional homolog of S. cerevisiae Fcy1p; mutation is associated with resistance to flucytosine (5-FC) in a clinical isolate; hyphal downregulated; gene has intron	-1.85965	2.66665E-11
orf19.2650	Protein of unknown function; rat catheter biofilm induced	-1.861433	4.30407E-08
TPD3	Subunit of protein serine/threonine phosphatase PPA2, involved in regulation of cytokinesis and morphogenesis; forms complex with catalytic subunit Pph21p that dephosphorylates septin Sep7p	-1.861903	3.6038E-19
orf19.1465	Has domain(s) with predicted N-acetyltransferase activity	-1.863295	2.95786E-07

orf19.2769	Putative protease B inhibitor; hyphal-induced expression; Cyr1p- and Ras1p-repressed	-1.864192	1.80559E-49
APL2	Phosphorylated protein of unknown function; mutation confers hypersensitivity to toxic ergosterol analog	-1.864507	7.27012E-19
orf19.2788	Has domain(s) with predicted RNA binding, pseudouridine synthase activity and role in pseudouridine synthesis	-1.868182	0.000106147
orf19.7051	Maf-like protein; rat catheter biofilm induced	-1.878932	0.00011368
IFF8	Putative GPI-anchored adhesin-like protein; decreased transcription is observed in an azole-resistant strain that overexpresses MDR1	-1.880636	0.000179665
OSM1	Putative flavoprotein subunit of fumarate reductase; soluble protein in hyphae; caspofungin repressed; stationary phase enriched protein; flow model biofilm induced; Spider biofilm repressed	-1.882637	8.29427E-27
LHP1	Ortholog(s) have RNA binding, RNA strand annealing activity, tRNA binding activity, role in tRNA 3'-trailer cleavage, tRNA 3'-trailer cleavage, endonucleolytic, tRNA folding, tRNA processing and nucleolus, nucleoplasm, nucleus localization	-1.884888	1.6834E-27
EPT1	Diacylglycerol cholinephosphotransferase and ethanolaminephosphotransferase, catalyzes the final step in Kennedy pathway of phosphatidylcholine and phosphatidylethanolamine biosynthesis	-1.887004	4.03398E-25
orf19.931	Ortholog of Candida albicans WO-1 : CAWG_04452	-1.893171	2.54347E-12
orf19.1266	Protein of unknown function; transcript detected on high-resolution tiling arrays	-1.894553	7.61883E-09
orf19.2201	Ortholog(s) have ribosome binding activity, role in mitochondrial respiratory chain complex III assembly, positive regulation of mitochondrial translation and Cbp3p-Cbp6 complex, mitochondrial ribosome localization	-1.896348	4.512E-09
MDJ2	Predicted component of the mitochondrial import motor; involved in protein import into mitochondrial matrix; early-stage flow model biofilm induced	-1.905899	4.5343E-05
YHB5	Flavohemoglobin-related protein; not required for normal NO resistance; predicted globin/FAD-binding/NAD(P)-binding domains but lacks some conserved residues of flavohemoglobins; filament induced; rat catheter and Spider biofilm induced	-1.908088	1.25707E-08
COX7	Putative cytochrome c oxidase; flucytosine induced; repressed by nitric oxide	-1.90969	4.51386E-07
SLC1	Putative fatty acyltransferase; protein repressed during the mating process	-1.911972	5.5717E-22
CSI2	Putative 66S pre-ribosomal particle component; Hap43-induced; essential for growth; transposon mutation affects filamentous growth; Spider biofilm induced	-1.913825	1.32176E-14

orf19.6537	Ortholog(s) have role in chromosome segregation and kinetochore, nuclear MIS12/MIND complex, spindle pole localization	-1.913867	0.006496257
ACP1	Putative mitochondrial acyl carrier protein involved in fatty acid biosynthesis; shows colony morphology-related gene regulation by Ssn6p; protein newly produced during adaptation to the serum	-1.914117	5.21451E-15
orf19.1110	Thiamine pyrophosphokinase, phosphorylates thiamine to produce the coenzyme thiamine pyrophosphate (thiamine diphosphate); active as a homodimer	-1.914962	2.45846E-09
FDH1	Formate dehydrogenase; oxidizes formate to CO2; Mig1 regulated; induced by macrophages; fluconazole-repressed; repressed by Efg1 in yeast, not hyphal conditions; stationary phase enriched; rat catheter and Spider biofilm induced	-1.918368	1.64776E-05
orf19.2076	Protein of unknown function; S. pombe ortholog SPAC7D4.05 encodes a predicted hydrolase; Hap43-repressed; Spider biofilm induced	-1.920574	2.29623E-15
NUO1	NADH-ubiquinone oxidoreductase subunit with roles in mitochondrial respiratory chain complex I assembly	-1.925792	5.63073E-18
orf19.2786	Ortholog(s) have AP-2 adaptor complex, cellular bud neck localization	-1.926511	3.87789E-09
TEA1	Telomerase subunit; allosteric activator of catalytic activity, but not required for catalytic activity; has TPR domain	-1.927564	4.35522E-05
SPR28	Septin; similar to S. cerevisiae meiotic/sporulation septin; mutant has no obvious phenotype; two introns with noncanonical branch site and 5' splice site, respectively; splicing inhibited upon exposure to alpha-factor	-1.931243	0.000296883
MNN11	Ortholog(s) have alpha-1,6-mannosyltransferase activity, role in protein N-linked glycosylation, protein glycosylation and mannan polymerase complex localization	-1.936138	0.00103236
orf19.3799	Ortholog(s) have role in ER-dependent peroxisome organization, endoplasmic reticulum inheritance, endoplasmic reticulum tubular network maintenance and endoplasmic reticulum tubular network organization, more	-1.937013	9.40036E-44
orf19.5539	Ortholog(s) have SNAP receptor activity, role in retrograde vesicle-mediated transport, Golgi to endoplasmic reticulum and SNARE complex, integral component of cytoplasmic side of endoplasmic reticulum membrane localization	-1.937143	2.697E-12
MRT4	Putative mRNA turnover protein; Hap43-induced; mutation confers hypersensitivity to tubercidin (7-deazaadenosine); rat catheter biofilm induced	-1.940018	4.32963E-15
orf19.4735	Ornithine cyclodeaminase family protein; Sef1, Sfu1, and Hap43-regulated; ortholog of S. cerevisiae YGL159W and S. pombe SPAP11E10.01; rat catheter biofilm induced	-1.940198	0.000134023

TPS3	Predicted trehalose-phosphate synthase regulatory subunit; regulated by Efg1; regulated by Tsa1, Tsa1B under H2O2 stress conditions; flow model biofilm induced (reports differ)	-1.942031	2.55543E-25
MET14	Putative adenylylsulfate kinase; predicted role in sulfur metabolism; possibly adherence-induced; protein present in exponential and stationary growth phase yeast; F-12/CO2 biofilm induced	-1.942717	2.09139E-25
SRB8	Putative RNA polymerase II mediator complex subunit; early-stage flow model biofilm induced	-1.954966	3.36019E-07
VPS70	Has domain(s) with predicted peptidase activity and role in proteolysis	-1.956239	6.82461E-20
orf19.1075	Protein of unknown function; Spider biofilm induced	-1.956411	0.001517275
PST1	Flavodoxin-like protein involved in oxidative stress protection and virulence; putative 1,4-benzoquinone reductase; hyphal-induced; regulated by Cyr1, Ras1, Efg1, Nrg1, Rfg1, Tup1; Hap43-induced; Spider biofilm induced	-1.962135	3.03826E-27
GLK4	Putative glucokinase; decreased expression in hyphae compared to yeast-form cells	-1.963048	2.21193E-10
orf19.154	Putative ortholog of S. cerevisiae Utp30; a U3-containing 90S preribosome complex protein; Hap43-induced; Spider biofilm induced	-1.965457	0.000180976
PPS1	Putative dual specificity protein phosphatase, similar to S. cerevisiae Pps1p; mutant shows virulence defect	-1.965938	1.2419E-08
orf19.2269	Putative 3-phosphoserine phosphatase; induced by benomyl or in azole-resistant strain that overexpresses MDR1; early-stage flow model biofilm induced; Spider biofilm repressed	-1.966393	1.43379E-25
orf19.2853	Protein of unknown function; rat catheter biofilm induced	-1.968253	6.43595E-06
orf19.2048	Proten of unknown function; transcript positively regulated by Sfu1; Hap43 repressed; Spider biofilm induced	-1.968519	5.26351E-24
MET3	TP sulfurlyase; sulfate assimilation; repressed by Met, Cys, Sfu1, or in fluconazole-resistant isolate; Hog1, caspofungin, white phase-induced; induced on biofilm formation, even in presence of Met and Cys; Spider, F-12/CO2 biofilm induced	-1.977121	3.63964E-25
PEX12	Ortholog(s) have ubiquitin protein ligase activity, role in protein import into peroxisome matrix, protein monoubiquitination and integral component of peroxisomal membrane, peroxisomal importomer complex localization	-1.979581	1.02815E-07
orf19.1344	Protein of unknown function; fluconazole-induced; Spider biofilm induced	-1.983077	8.43679E-06
UGA4	Putative gamma-aminobutyric acid/polyamine permease; nitrogen catabolite repressed gene, induced in absence of preferred N sources; transcriptionally induced upon phagocytosis by macrophage; gene regulation by nitrogen source requires Gat1p	-1.983087	0.001031484

orf19.2175	Mitochondrial apoptosis-inducing factor; induced by nitric oxide; Spider biofilm induced; rat catheter biofilm repressed	-1.983319	1.1569E-25
orf19.2938	Putative mitochondrial inner membrane protein with a predicted role in the assembly of respiratory complex III; Hap43p-repressed gene; S. cerevisiae ortholog FMP25 localizes to mitochondrion	-1.988652	2.83014E-10
orf19.5625	Ortholog of C. dubliniensis CD36 : Cd36_63640, C. auris B8441 : B9J08_001441, Pichia stipitis Pignal : PICST_32878 and Candida guilliermondii ATCC 6260 : PGUG_03423	-1.995557	0.024802198
DPP1	Putative diacylglycerol pyrophosphate phosphatase of diacylglycerol production for phospholipid biosynthesis; downregulation correlates with clinical development of fluconazole resistance	-1.999205	4.02686E-13
ISY1	Putative pre-mRNA-splicing factor; mutation confers resistance to 5-fluorocytosine (5-FC); rat catheter biofilm induced	-1.999232	0.00134838
orf19.7225	Ortholog of C. dubliniensis CD36 : Cd36_13150, C. parapsilosis CDC317 : CPAR2_700610, C. auris B8441 : B9J08_003318 and Candida tenuis NRRL Y-1498 : CANTEDRAFT_135418	-2.007783	4.51699E-10
orf19.1301	Ortholog(s) have protein-macromolecule adaptor activity and role in proteasome regulatory particle assembly	-2.008135	1.15927E-07
PGA12	Putative GPI-anchored protein	-2.010196	0.013432596
MDH1	Mitochondrial malate dehydrogenase; regulated by Mig1, Tup1, white-opaque switch, phagocytosis; induced in high iron; antigenic during murine and human infection; repressed in Spider biofilms by Bcr1, Tec1, Ndt80, Rob1, Brg1	-2.011477	6.54902E-37
orf19.7366	Ortholog(s) have S-adenosylmethionine-dependent methyltransferase activity, rRNA (uridine-N3-)-methyltransferase activity, role in rRNA base methylation and nucleolus localization	-2.012926	2.98103E-05
orf19.3352	Has domain(s) with predicted oxidoreductase activity and role in metabolic process	-2.016309	3.61064E-07
ARG3	Putative ornithine carbamoyltransferase; Gcn4-regulated; Hap43-induced; repressed in alkalinizing medium; rat catheter and Spider biofilm induced	-2.016752	2.13922E-23
orf19.1738.1	Protein involved in microtubule morphogenesis; required for protection from excess free beta-tubulin; possibly involved the beta-tubulin folding; Spider biofilm repressed	-2.018937	2.56507E-07
IFR1	Predicted oxidoreductase/dehydrogenase; induced by nitric oxide; rat catheter and Spider biofilm induced	-2.03143	1.25255E-19
orf19.2001	Has domain(s) with predicted 2-oxoglutarate-dependent dioxygenase activity, L-ascorbic acid binding, iron ion binding, oxidoreductase activity, acting on paired donors, with incorporation or reduction of molecular oxygen activity	-2.031851	1.02898E-12

orf19.5300	Calnexin; integral membrane ER chaperone involved in folding and quality control of glycoproteins; caspofungin induced	-2.031868	5.46499E-26
orf19.4861	Protein of unknown function; rat catheter biofilm repressed	-2.032336	0.009016354
orf19.1297	Ortholog(s) have phosphatidylinositol-3-phosphate binding activity and role in autophagy of mitochondrion, cytoplasm to vacuole transport by the Cvt pathway, early endosome to Golgi transport, macroautophagy	-2.035226	1.16554E-05
UAP1	UDP-N-acetylglucosamine pyrophosphorylase, catalyzes biosynthesis of UDP-N-acetylglucosamine from UTP and N-acetylglucosamine 1-phosphate; functional homolog of S. cerevisiae Qri1p; alkaline upregulated	-2.035526	7.86753E-32
orf19.1500	Ortholog of C. dubliniensis CD36 : Cd36_16720, C. parapsilosis CDC317 : CPAR2_210210, C. auris B8441 : B9J08_004860 and Candida tenuis NRRL Y-1498 : CANTEDRAFT_128446	-2.03777	3.58992E-15
orf19.3466	Predicted methyltransferase; downregulated by fluphenazine treatment or in an azole-resistant strain that overexpresses CDR1 and CDR2	-2.039896	4.35799E-07
orf19.773	Protein similar to S. cerevisiae Rsa3 predicted nucleolar protein involved in maturation of pre-60S ribosomal particles; rat catheter and Spider biofilm induced	-2.043194	3.32877E-05
GOR1	Ortholog(s) have glyoxylate reductase (NAD+) activity, role in glyoxylate catabolic process and extracellular region localization	-2.048737	2.40773E-19
ALG2	Putative mannosyltransferase involved in cell wall mannan biosynthesis; transcription is elevated in chk1, nik1, and sln1 homozygous null mutants	-2.051497	2.76275E-23
orf19.4498	Ortholog of C. dubliniensis CD36 : Cd36_19120, C. parapsilosis CDC317 : CPAR2_103880, C. auris B8441 : B9J08_003718 and Candida tenuis NRRL Y-1498 : CANTEDRAFT_115581	-2.053185	0.000867069
CEK2	MAP kinase required for wild-type efficiency of mating; component of the signal transduction pathway that regulates mating; ortholog of S. cerevisiae Fus3; induced by Cph1, pheromone; transposon mutation affects filamentous growth	-2.058032	0.005345876
orf19.6346	Putative nuclear export protein; Hap43p-induced gene; decreased transcription is observed in an azole-resistant strain that overexpresses MDR1	-2.06397	4.03018E-17
TOM70	Ortholog(s) have mitochondrion targeting sequence binding, protein transmembrane transporter activity	-2.068613	2.78268E-32
orf19.51	Ortholog(s) have RNA binding activity, role in cellular response to osmotic stress and cytoplasmic stress granule localization	-2.06937	6.49839E-16
FTH2	Ortholog(s) have DNA binding activity, role in regulation of DNA damage checkpoint and cytoplasm, nuclear periphery localization	-2.070449	2.43902E-07

SNU114	Protein similar to S. cerevisiae Snu114p, which is an RNA helicase involved in pre-mRNA splicing; likely to be essential for growth, based on an insertional mutagenesis strategy	-2.071326	1.71159E-06
END3	Regulated by Gcn4p; induced in response to amino acid starvation (3-aminotriazole treatment)	-2.071409	4.72963E-24
orf19.192	Ortholog of C. dubliniensis CD36 : Cd36_19320, C. parapsilosis CDC317 : CPAR2_209670, C. auris B8441 : B9J08_002950 and Candida tenuis NRRL Y-1498 : CANTEDRAFT_113478	-2.075634	6.72898E-08
orf19.3250	Ortholog(s) have RNA binding activity, role in mRNA splicing, via spliceosome and U1 snRNP, U2-type prespliceosome, nucleus localization	-2.077053	8.02331E-16
orf19.1296	Ortholog(s) have role in spliceosomal snRNP assembly and U4/U6 x U5 tri-snRNP complex localization	-2.077674	8.35774E-12
orf19.6597	Ortholog of S. cerevisiae : YLR287C, C. glabrata CBS138 : CAGL0M09757g, C. dubliniensis CD36 : Cd36_34830, C. parapsilosis CDC317 : CPAR2_701110 and C. auris B8441 : B9J08_000560	-2.082034	7.97994E-15
orf19.3585	Putative tRNA methyltransferase complex subunit; regulated by Gcn4; repressed in response to amino acid starvation (3-aminotriazole treatment); Spider biofilm induced	-2.08216	7.77996E-07
MET15	O-acetylhomoserine O-acetylserine sulfhydrylase; sulfur amino acid synthesis; immunogenic; Hog1, adherence-induced; brown color of mutant in Pb(2+) medium a visual selection; chlamydospore formation induced, F-12/CO2 biofilm induced	-2.085419	1.36431E-33
PTP2	Predicted protein tyrosine phosphatase; involved in regulation of MAP kinase Hog1 activity; induced by Mnl1 under weak acid stress; rat catheter and Spider biofilm induced	-2.086602	1.35857E-11
ROT1	Similar to S. cerevisiae Rot1p, which is involved in cell wall 1,6-beta-glucan biosynthesis; has predicted transmembrane region; possibly an essential gene, disruptants not obtained by UAU1 method	-2.08982	2.66126E-09
orf19.4316	Trimethyllysine dioxygenase, the first enzyme in the carnitine biosynthesis pathway; hypha-induced expression, regulated by Cyr1, Ras1, Efg1; rat catheter biofilm repressed	-2.091082	8.37659E-24
orf19.3758	Has domain(s) with predicted membrane localization	-2.092033	6.17016E-26
orf19.2733	Putative subunit of phosphatidylinositol 3-kinase complexes I and II; transcription is activated in the presence of elevated CO2	-2.092856	2.9837E-15
SAP3	Secreted aspartyl proteinase, acts in utilization of protein as nitrogen source; assessment of virulence role complicated by URA3 effects; regulated by growth phase; produced by opaque phase cells; alpha-pheromone repressed	-2.096423	8.05546E-08

MSH3	Mismatch repair protein; predicted role in repair of insertion or deletion mutations and removal of nonhomologous DNA ends; rat catheter biofilm repressed	-2.107068	1.07994E-07
orf19.3325	Putative glycogen synthesis initiator; regulated by Efg1 and Efh1; Hog1-repressed; colony morphology-related gene regulation by Ssn6; induced by prostaglandins; flow model biofilm induced	-2.107365	5.52047E-17
orf19.2962	Protein of unknown function; Spider biofilm induced	-2.109805	4.48558E-10
GPH1	Putative glycogen phosphorylase; role in glycogen metabolism; regulated by Ssk1, Mig1, Tup1, Hap43; fluconazole-induced; localizes to cell surface of hyphae, not yeast; stationary phase enriched protein; Spider biofilm induced	-2.110593	5.08495E-35
XKS1	Putative xylulokinase; Hap43-repressed; induced by prostaglandins; rat catheter biofilm repressed	-2.114126	1.25354E-31
orf19.7343	Ortholog(s) have role in snoRNA splicing, spliceosomal conformational changes to generate catalytic conformation and U4/U6 x U5 tri-snRNP complex localization	-2.115265	3.83642E-09
orf19.3219	Ortholog of S. cerevisiae Sia1; involved in activation of the Pma1 plasma membrane H+-ATPase by glucose in S. cerevisiae; Spider biofilm induced	-2.119536	3.42098E-24
orf19.2542	Ortholog of C. dubliniensis CD36 : Cd36_26520, C. parapsilosis CDC317 : CPAR2_802860, C. auris B8441 : B9J08_003507 and Candida tenuis NRRL Y-1498 : CANTEDRAFT_94578	-2.121917	7.21044E-05
orf19.904	Ortholog(s) have cytoplasm, nucleus localization	-2.124045	5.34326E-22
GEF2	Member of the voltage chloride channel family; Hap43p-repressed gene	-2.12435	6.22855E-20
orf19.1618.1	Ortholog of C. dubliniensis CD36 : Cd36_82240, C. parapsilosis CDC317 : CPAR2_405130, C. auris B8441 : B9J08_002633 and Candida tenuis NRRL Y-1498 : CANTEDRAFT_127317	-2.12811	0.014064254
orf19.3572	Ortholog of C. dubliniensis CD36 : Cd36_19880, C. auris B8441 : B9J08_003635, Candida tenuis NRRL Y-1498 : CANTEDRAFT_117672 and Debaryomyces hansenii CBS767 : DEHA2D11154g	-2.14439	5.13399E-11
GPI1	Putative protein of GPI synthesis; expression is reduced in a fluconazole-resistant isolate	-2.1456	1.20665E-07
orf19.7227	Protein phosphatase inhibitor; Hap43-repressed; homozygous Tn insertion decreases colony wrinkling but does not block hyphal growth in liquid media; mutation confers hypersensitivity to toxic ergosterol analog; Spider biofilm induced	-2.153972	4.49205E-05
MED8	Ortholog(s) have RNA polymerase II cis-regulatory region sequence-specific DNA binding, TBP-class protein binding,	-2.155891	3.35016E-07

	protein-macromolecule adaptor activity, transcription corepressor activity		
orf19.4764	Ortholog(s) have poly(A)-specific ribonuclease activity, role in nuclear-transcribed mRNA poly(A) tail shortening, postreplication repair and PAN complex localization	-2.15686	4.54822E-09
GLK1	Putative glucokinase; transcript regulated upon yeast-hyphal switch; Efg1 regulated; fluconazole-induced; induced in core stress response; colony morphology-related gene regulation by Ssn6; GlcNAc-induced protein	-2.157775	1.01035E-33
orf19.3105	Putative cytochrome P450 protein; possibly an essential gene, disruptants not obtained by UAU1 method	-2.158725	1.52876E-12
orf19.4324	Has domain(s) with predicted NADH dehydrogenase (ubiquinone) activity, role in mitochondrial respiratory chain complex I assembly and membrane localization	-2.160037	5.71852E-08
orf19.4633	Ortholog(s) have carbonyl reductase (NADPH) activity, oxidoreductase activity, serine 3-dehydrogenase activity	-2.164835	2.39502E-30
AGE3	Putative ADP-ribosylation factor GTPase activating protein, functional ortholog of S. cerevisiae GCS1; mutation affects endocytosis, hyphal growth, chemical and drug resistance, and sensitivity to cell wall inhibitors	-2.166273	3.21864E-31
GLX3	Glutathione-independent glyoxalase; binds human immunoglobulin E; alkaline, fluconazole, Hog1 repressed; hypoxia, oxidative stress via Cap1, Hap43 induced; stationary-phase enriched; rat catheter, Spider biofilm induced	-2.169836	2.28316E-46
TLG2	Putative syntaxin-like t-SNARE; macrophage/pseudohyphal-repressed	-2.171406	6.96127E-10
IHD1	GPI-anchored protein; alkaline, hypha-induced; regulated by Nrg1, Rfg1, Tup1 and Tsa1, Tsa1B in minimal media at 37; oralpharyngeal candidasis induced ; Spider biofilm induced; regulated in Spider biofilms by Tec1, Efg1, Ndt80, Rob1, Brg1	-2.17729	2.18487E-56
HOC1	Protein with similarity to mannosyltransferases; similar to S. cerevisiae Hoc1p and C. albicans Och1p	-2.177837	1.6984E-19
orf19.6354	Ortholog of C. dubliniensis CD36 : Cd36_11870, C. parapsilosis CDC317 : CPAR2_201650, C. auris B8441 : B9J08_002515 and Candida tenuis NRRL Y-1498 : cten_CGOB_00224	-2.179118	1.43036E-07
orf19.4914	Ortholog(s) have role in cellular response to DNA damage stimulus and FANCM-MHF complex localization	-2.179334	0.000847641
RME1	Zinc finger protein, controls asexual sporulation; white-specific transcript; upregulation correlates with clinical development of fluconazole resistance; Upc2-regulated in hypoxia; flow model biofilm induced; Spider biofil	-2.180949	2.55298E-06
orf19.6862	Hap43-induced gene; mutation confers hypersensitivity to 5-fluorocytosine (5-FC), 5-fluorouracil (5-FU), and tubercidin (7-deazaadenosine)	-2.181353	7.27579E-13

HEM14	Putative protoporphyrinogen oxidase; involved in heme biosynthesis; predicted Kex2p substrate; iron regulated transcript; Yfh1-induced; Hap43-repressed; rat catheter biofilm repressed	-2.182129	2.92407E-14
LYS9	Saccharopine dehydrogenase; lysine biosynthesis; soluble protein in hyphae; amphotericin B repressed; Gcn4-regulated; colony morphology-related gene regulation by Ssn6; protein present in exponential and stationary growth phase yeast	-2.183422	2.56315E-40
MNN22	Alpha-1,2-mannosyltransferase; required for normal cell wall mannan; regulated by Tsa1, Tsa1B at 37 deg; repressed in core stress response; NO, Hog1 induced; confers sensitivity to cell wall perturbing agents; Spider biofilm repressed	-2.185249	2.62023E-27
orf19.6305	Hydroxytrimethyllysine aldolase, the second enzyme in the carnitine biosynthesis pathway; rat catheter biofilm repressed	-2.185805	5.53539E-27
orf19.5618	Ortholog(s) have SNARE binding, unfolded protein binding activity	-2.187883	5.45851E-15
orf19.3690.2	Ribosomal 60S subunit protein; Spider biofilm repressed	-2.190218	1.19773E-22
orf19.305	Protein of unknown function; upregulated in a cyr1 null mutant	-2.199564	9.3799E-19
orf19.6797	Ortholog of C. dubliniensis CD36 : Cd36_86960, C. parapsilosis CDC317 : CPAR2_808800, C. auris B8441 : B9J08_004750 and Candida tenuis NRRL Y-1498 : CANTEDRAFT_113621	-2.206451	1.53267E-06
orf19.6453	Ortholog of C. dubliniensis CD36 : Cd36_72300, C. parapsilosis CDC317 : CPAR2_703060, C. auris B8441 : B9J08_001030 and Candida tenuis NRRL Y-1498 : CANTEDRAFT_92002	-2.206573	3.15536E-07
GYP8	Ortholog(s) have GTPase activator activity, role in vesicle-mediated transport and cytoplasm localization	-2.21209	1.42303E-07
orf19.2165	Predicted hydrolase; induced by nitric oxide	-2.214113	1.08505E-29
orf19.4287	Putative oxidoreductase; Hap43-repressed gene; clade-associated gene expression	-2.214281	8.77087E-28
orf19.1491	Ortholog(s) have RNA binding activity, role in mRNA splicing, via spliceosome and U1 snRNP, U2-type prespliceosome localization	-2.216738	1.03736E-13
orf19.2371	Putative Gag protein of retrotransposon Tca2; separated by a stop codon from Pol protein orf19.2372; likely translated as single polyprotein that includes Gag, reverse transcriptase, protease, and integrase; rat catheter biofilm repressed	-2.219753	1.62063E-33
orf19.7594	Protein of unknown function; Spider biofilm induced	-2.232517	0.00010521
orf19.3439	Protein of unknown function; Cyr1-repressed; rat catheter and Spider biofilm induced	-2.236742	8.32034E-06
VPS22	ESCRT-II complex protein with a role in multivesicular body (MVB) trafficking; required for processing of Rim8p; Hap43p-repressed gene	-2.241564	1.40756E-09

TIM9	Predicted protein of the mitochondrial intermembrane space; rat catheter biofilm induced; Spider biofilm repressed	-2.241727	1.45339E-05
orf19.3633	Ortholog(s) have role in purine nucleobase catabolic process	-2.243992	4.36225E-06
RNR2	Putative ribonucleoside diphosphate reductase;colony morphology-related gene regulation by Ssn6; transcript regulated by tyrosol and cell density; Hap43-repressed; Spider biofilm induced	-2.251724	1.76123E-59
GAL102	UDP-glucose 4,6-dehydratase; role in mannosylation of cell wall proteins; mutation confers hypersensitivity to toxic ergosterol analog; overlaps orf19.3673; Spider biofilm induced	-2.262969	3.34487E-07
orf19.4504	Has domain(s) with predicted oxidoreductase activity, zinc ion binding activity	-2.26381	1.6703E-12
orf19.4066	Putative glycerol-3-phosphate acyltransferase; Hog1-repressed	-2.266117	2.38802E-21
orf19.7553	Protein of unknown function; rat catheter biofilm repressed	-2.267426	0.00016582
orf19.836	Protein likely to be essential for growth, based on an insertional mutagenesis strategy	-2.268279	1.21838E-07
PRO2	Putative gamma-glutamyl phosphate reductase with a predicted role in proline biosynthesis; regulated by Gcn2p and Gcn4p	-2.268709	2.38248E-37
SEM1	Component of the 19S regulatory particle lid of the proteasome	-2.268713	2.86138E-11
SOD1	Cytosolic copper- and zinc-containing superoxide dismutase; role in protection from oxidative stress; required for full virulence; alkaline induced by Rim101; induced by human blood; rat catheter, flow model and Spider biofilm repressed	-2.269035	5.01794E-59
RPN12	Subunit of the 19S regulatory particle lid of the proteasome	-2.284232	7.33861E-44
PEX8	Putative peroxisomal biogenesis factor; expression regulated during planktonic growth	-2.288936	3.05803E-05
orf19.6604	Ortholog of S. cerevisiae Pba1 that is involved in 20S proteasome assembly; upregulated in a cyr1 null mutant; contains a 5' UTR intron	-2.292647	4.39749E-27
AHP2	Putative thiol-specific peroxiredoxin; macrophage-downregulated gene	-2.292859	1.15255E-30
OPT5	Oligopeptide transporter; fungal-specific (no human or murine homolog); induced by BSA, but not by tetrapeptide or pentapeptide	-2.294542	0.006439583
SIT1	Transporter of ferrichrome siderophores, not ferrioxamine B; required for human epithelial cell invasion in vitro, not for mouse systemic infection; regulated by iron, Sfu1, Rfg1, Tup1, Hap43; rat catheter and Spider biofilm induced	-2.295217	1.03556E-08
orf19.2936	Ortholog of C. dubliniensis CD36 : Cd36_02330, C. parapsilosis CDC317 : CPAR2_106450, Candida tropicalis MYA-3404 : CTRG_04653 and Candida albicans WO-1 : CAWG_01134	-2.295985	0.000154919

orf19.8	Ortholog of C. dubliniensis CD36 : Cd36_32690, C. parapsilosis CDC317 : CPAR2_204290, Candida tenuis NRRL Y-1498 : CANTEDRAFT_121459 and Debaryomyces hansenii CBS767 : DEHA2E03938g	-2.298156	8.37671E-09
HSP70	Putative hsp70 chaperone; role in entry into host cells; heat-shock, amphotericin B, cadmium, ketoconazole-induced; surface localized in yeast and hyphae; antigenic in host; farnesol-downregulated in biofilm; Spider biofilm induced	-2.300584	6.13906E-57
orf19.1449	Protein of unknown function; induced in azole-resistant strain that overexpresses MDR1; protein present in exponential and stationary growth phase yeast cultures; Spider biofilm induced	-2.309554	1.66019E-14
orf19.3916	Ortholog of C. dubliniensis CD36 : Cd36_53980, C. parapsilosis CDC317 : CPAR2_100980, C. auris B8441 : B9J08_004358 and Candida tenuis NRRL Y-1498 : CANTEDRAFT_116966	-2.309855	4.14623E-19
orf19.6275	Ortholog(s) have protein serine/threonine kinase activity, ribosomal protein S6 kinase activity, role in TORC1 signaling, peptidyl-serine phosphorylation, protein autophosphorylation and cytoplasm localization	-2.317341	5.97293E-11
orf19.4639	Protein present in exponential and stationary growth phase yeast cultures	-2.320266	1.18108E-17
orf19.3214	Alpha/beta-HydrolasE superfamily protein; membrane-localized	-2.323919	1.98619E-11
LEU4	Putative 2-isopropylmalate synthase; regulated by Nrg1, Mig1, Tup1, Gcn4; induced by human whole blood or PMNs; macrophage/pseudohyphal-repressed after 16h; Spider biofilm repressed	-2.328618	2.56587E-48
ECO1	Putative acetyltransferase	-2.329574	1.03376E-11
RTT101	Putative cullin subunit of E3 ubiquitin ligase complex, involved in response to DNA damage; induced by alpha pheromone in SpiderM medium	-2.329956	7.23271E-08
orf19.4612	Protein with a dienelactone hydrolase domain; Hap43-repressed gene	-2.332019	2.47048E-22
orf19.792	Ortholog(s) have protein serine/threonine kinase activity, protein serine/threonine/tyrosine kinase activity, protein tyrosine kinase activity	-2.336812	0.008595789
IFF4	Adhesin-like cell surface protein; putative GPI-anchor; null mutant germ tubes show decreased adhesion to plastic substrate; mutants are viable; Hap43-repressed gene	-2.343359	0.000116522
SUR2	Putative ceramide hydroxylase; predicted enzyme of sphingolipid biosynthesis; regulated by Tsa1, Tsa1B under H2O2 stress conditions; Spider and flow model biofilm induced	-2.346358	2.65835E-28
QCR7	Putative ubiquinol-cytochrome-c reductase, subunit 7; Hap43p-repressed gene	-2.347699	8.12001E-21

HSP12	Heat-shock protein; induced by osmotic/oxidative/cadmium stress, fluphenazine treatment, low iron, CDR1 and CDR2 overexpression, or ssn6 or ssk1 null mutation; overexpression increases resistance to farnesol and azoles	-2.350329	2.06502E-46
orf19.4216	Putative heat shock protein; decreased expression in hyphae; transcription is increased in populations of cells exposed to fluconazole over multiple generations; overexpression increases resistance to farnesol and azoles	-2.350329	2.06502E-46
PLB1	Phospholipase B; host cell penetration and virulence in mouse systemic infection; Hog1-induced; signal sequence, N-glycosylation, and Tyr phosphorylation site; induced in fluconazole-resistant strains; rat catheter biofilm repressed	-2.355481	0.008678064
orf19.6310	Has domain(s) with predicted myosin binding activity	-2.355687	3.67313E-07
GTR1	Putative GTP-binding protein; involved in activation of TOR1C during starvation response; transcript is upregulated in clinical isolates from HIV+ patients with oral candidiasis; (see Locus History Note for Assembly 19 correction)	-2.356379	1.43106E-05
orf19.6230	Ortholog(s) have GDP binding, RNA NAD-cap (NAD-forming) hydrolase activity, RNA pyrophosphohydrolase activity, enzyme regulator activity, phosphodiesterase decapping endonuclease activity	-2.362825	4.81472E-12
PPT1	Putative serine/threonine phosphatase; induced in high iron	-2.364399	2.27706E-12
MAK32	Putative protein involved in the structural stability of L-A double-stranded RNA-containing particles; downregulated upon adherence to polystyrene	-2.371989	3.46233E-10
orf19.6637	Predicted glycosyl hydrolase; hypoxia induced; flow model biofilm induced	-2.375162	6.57677E-44
KNS1	Protein kinase involved in negative regulation of PolIII transcription; effector kinase of the TOR signaling pathway, phosphorylates Rpc53p to regulate ribosome and tRNA biosynthesis; Spider and flow model biofilm induced	-2.375244	1.57051E-06
orf19.100	Alpha/beta hydrolase and lipase domain protein; Hap43-repressed; Spider and flow model biofilm induced	-2.377474	8.31087E-06
orf19.1368	Protein of unknown function; flow model biofilm induced; Spider biofilm induced	-2.377902	2.28443E-06
AVT7	Ortholog of S. cerevisiae Avt7 transporter; repressed upon adherence to polystyrene; constitutive expression independent of MTL or white-opaque status; Spider biofilm induced	-2.380403	1.52976E-27
orf19.3247	Putative ortholog of S. cerevisiae Laa1p; likely to be essential for growth, based on an insertional mutagenesis strategy	-2.38608	3.68563E-16
orf19.4751	Ortholog(s) have structural constituent of ribosome activity and mitochondrial small ribosomal subunit localization	-2.393963	1.57366E-14
TOM22	Putative mitochondrial import receptor subunit; colony morphology-related gene regulation by Ssn6	-2.397633	2.10116E-18

MLH1	Putative mismatch repair protein; cell-cycle regulated periodic mRNA expression	-2.400207	4.64723E-05
NCE103	Carbonic anhydrase; converts of CO2 to bicarbonate; essential for virulence in host niches with limited CO2, normal white-opaque switch; Mnl1-induced in weak acid stress; Hap43-induced gene; F-12/CO2, rat catheter, Spider biofilm induced	-2.401711	1.57599E-11
EFH1	APSES transcription factor; homodimer; minor role in transcriptional regulation vs Efg1; regulates filamentous growth, phenotypic switch; EFG1 and EFH1 genetically interact; expression interferes with mouse intestinal tract colonization	-2.409282	1.19808E-32
YWP1	Secreted yeast wall protein; possible role in dispersal in host; involved in adhesion and biofilm formation; propeptide; growth phase, phosphate, Ssk1/Ssn6/Efg1/Efh1/Hap43 regulated; mRNA binds She3; flow and Spider biofilm repressed	-2.410927	9.74869E-52
orf19.4676	Protein with homology to mitochondrial intermembrane space proteins; regulated by Sef1p-, Sfu1p-, and Hap43p	-2.418813	2.53574E-07
orf19.137	Putative transferase involved in phospholipid biosynthesis; induced by alpha pheromone in SpiderM medium	-2.419616	3.63717E-31
BUD16	Putative pyridoxal kinase; a key enzyme in pyridoxal 5'-phosphate synthesis, the active form of vitamin B6; involved in bud-site selection and genome integrity in S. cerevisiae; induced by alpha pheromone in SpiderM medium	-2.420876	6.85219E-13
orf19.2889	Ortholog(s) have role in ATP-dependent chromatin remodeling, chromatin remodeling, histone exchange and positive regulation of cellular response to phosphate starvation, more	-2.448701	5.91054E-05
orf19.6194	Ortholog of C. dubliniensis CD36 : Cd36_06640, C. parapsilosis CDC317 : CPAR2_208220, C. auris B8441 : B9J08_002282 and Candida tenuis NRRL Y-1498 : CANTEDRAFT_136902	-2.454373	5.06151E-07
VMA13	Predicted proton-transporting ATPase; predicted role in ATP hydrolysis coupled proton transport; rat catheter biofilm repressed	-2.457721	1.66841E-30
GSY1	Glycogen synthase (UDP glucose/starch glucosyltransferase); transcript repressed by yeast-hyphal switch, Efg1-regulated; strong oxidative stress induced; colony morphology-related regulation by Ssn6; stationary phase enriched	-2.470192	3.25133E-47
orf19.1367.1	Ortholog of C. parapsilosis CDC317 : CPAR2_407330, C. auris B8441 : B9J08_001010, Candida tenuis NRRL Y-1498 : CANTEDRAFT_114646 and Debaryomyces hansenii CBS767 : DEHA2B05654g	-2.473085	0.000259986
MAL2	Alpha-glucosidase; hydrolyzes sucrose for sucrose utilization; transcript regulated by Suc1, induced by maltose, repressed	-2.476938	4.36289E-24

	by glucose; Tn mutation affects filamentous growth; upregulated in RHE model; rat catheter and Spider biofilm induced		
RPL35	Ribosomal protein; downregulation correlates with clinical development of fluconazole resistance; colony morphology-related gene regulation by Ssn6; Hap43-induced; Spider biofilm repressed	-2.477504	3.80925E-08
orf19.120	Has domain(s) with predicted protein C-terminal S-isoprenylcysteine carboxyl O-methyltransferase activity, role in C-terminal protein methylation and integral component of membrane localization	-2.477626	0.000176134
PSA2	Mannose-1-phosphate guanyltransferase; Hap43, macrophage-repressed; stationary phase enriched protein; Spider biofilm induced; rat catheter biofilm repressed	-2.481406	4.88688E-15
HPD1	3-hydroxypropionate dehydrogenase; involved in degradation of toxic propionyl-CoA; rat catheter and Spider biofilm induced	-2.48226	0.000661789
orf19.2728	Ortholog(s) have DNA clamp loader activity, role in DNA damage checkpoint signaling, nucleotide-excision repair, reciprocal meiotic recombination and Rad17 RFC-like complex localization	-2.482995	6.2738E-14
orf19.4117	Ortholog of C. dubliniensis CD36 : Cd36_20450, C. parapsilosis CDC317 : CPAR2_104160, C. auris B8441 : B9J08_001214 and Candida tenuis NRRL Y-1498 : CANTEDRAFT_95516	-2.48571	1.12139E-20
SAM50	Predicted component of the SAM complex involved in mitochondrial protein import	-2.488434	1.16134E-17
orf19.6802	Ortholog(s) have palmitoyltransferase activity, role in protein palmitoylation, vacuole fusion, non-autophagic and fungal-type vacuole, fungal-type vacuole membrane localization	-2.493186	4.92527E-10
FMO1	Putative oxidoreductase; mutation confers hypersensitivity to toxic ergosterol analog, and to amphotericin B	-2.5025	0.001671472
OPT6	Putative oligopeptide transporter; fungal-specific (no human or murine homolog); expression of OPT6, OPT7, or OPT8 does not suppress defect of mutant lacking Opt1p, Opt2p, and Opt3p; alleles are nonidentical	-2.502821	2.14178E-10
orf19.5522	Ortholog of C. dubliniensis CD36 : Cd36_62760, C. parapsilosis CDC317 : CPAR2_601700, C. auris B8441 : B9J08_001639 and Candida tenuis NRRL Y-1498 : CANTEDRAFT_115220	-2.503706	4.17788E-16
orf19.2047	Putative protein of unknown function; Hap43p-repressed gene; mutation confers hypersensitivity to toxic ergosterol analog, and to amphotericin B	-2.515445	1.13764E-50
orf19.5620	Stationary phase enriched protein; Gcn4-regulated; induced by amino acid starvation (3-AT), benomyl or in azole-resistant	-2.51581	9.75939E-53

	strain that overexpresses MDR1; flow model biofilm induced; rat catheter biofilm repressed; overlaps orf19.5621		
orf19.1862	Possible stress protein; increased transcription associated with CDR1 and CDR2 overexpression or fluphenazine treatment; regulated by Sfu1, Nrg1, Tup1; stationary phase enriched protein; Spider biofilm induced	-2.524895	2.16281E-15
orf19.5291	Ortholog(s) have role in phospholipid biosynthetic process, phospholipid metabolic process, postreplication repair and endoplasmic reticulum localization	-2.52557	8.88332E-18
orf19.6699	Ortholog(s) have histidinol-phosphatase activity and role in histidine biosynthetic proces	-2.525789	9.18727E-21
PGM2	Ortholog of S. cerevisiae Pgm2; induced in planktonic culture; Tye7p-regulated; flow model biofilm induced; rat catheter biofilm repressed	-2.529588	1.74932E-60
orf19.1796	Putative glyoxylate reductase; acts on glyoxylate and hydroxypyruvate substrates; Spider biofilm repressed	-2.531152	1.24542E-35
orf19.2368	Predicted component of U5 snRNP; Spider biofilm induced	-2.532055	9.16495E-08
orf19.2836	Protein with similarity to carbonic anhydrases	-2.534272	0.013720283
orf19.4528	Ortholog(s) have role in vacuolar protein processing	-2.534998	2.99338E-13
RPB8	Putative subunit of RNA polymerases I, II, and III; regulated by Gcn4p; repressed in response to amino acid starvation (3-aminotriazole treatment); heterozygous null mutant exhibits resistance to parnafungin	-2.544062	6.29309E-19
ZFU2	Zn(II)2Cys6 transcription factor; regulator of yeast form adherence; mutants display increased colonization of mouse kidneys; required for yeast cell adherence to silicone substrate; Spider biofilm induced	-2.544757	1.40703E-21
orf19.4913	Ortholog(s) have ubiquitin binding activity	-2.552151	8.78569E-11
orf19.2306	Ortholog(s) have acireductone dioxygenase (Ni2+-requiring) activity and role in L-methionine salvage from methylthioadenosine	-2.552684	9.91269E-17
AYR2	Putative NADPH-dependent 1-acyl dihydroxyacetone phosphate reductase; shows colony morphology-related gene regulation by Ssn6p	-2.557503	7.53734E-45
orf19.4116	Ortholog(s) have N-acetyltransferase activity, role in response to drug and nuclear envelope, plasma membrane localization	-2.559501	8.78148E-19
orf19.2503	Ortholog of S. cerevisiae : YGL138C, C. glabrata CBS138 : CAGL0H08910g, C. dubliniensis CD36 : Cd36_80910, C. parapsilosis CDC317 : CPAR2_102230 and C. auris B8441 : B9J08_002616	-2.559634	0.001967483
MAM33	Putative mitochondrial acidic matrix protein; regulated by Ssn6p; protein present in exponential and stationary growth phase yeast cultures	-2.562439	1.78782E-36

orf19.6316.4	Protein of unknown function; Hap43-induced; rat catheter and Spider biofilm induced	-2.567278	0.018499135
MODF	Has domain(s) with predicted ATP binding, ATPase, nucleoside-triphosphatase activity	-2.567528	6.16381E-13
orf19.4132	Protein of unknown function; UPF0057 protein family member; localizes to the plasma membrane; Spider biofilm induced	-2.569255	6.14308E-12
SEN15	Similar to delta subunit of tRNA splicing endonuclease; increased transcription is observed upon fluphenazine treatment	-2.57453	2.9308E-08
PRM9	Protein described a similar to S. cerevisiae Prm9; not the ortholog though; mutant is viable	-2.57465	0.001367468
CCJ1	Protein involved in cell cycle regulation; ortholog of S. pombe SPAC1071.09c DNAJ domain protein; Hap43-induced gene	-2.58147	9.809E-23
AQY1	Aquaporin water channel; osmotic shock resistance, WT freeze tolerance; virulent in mice; flucytosine repressed; flow model/RPMI/Spider/rat catheter biofilm induced; required for RPMI biofilm formation; Bcr1-induced in a/a RPMI biofilms	-2.581768	8.11682E-32
orf19.1152	Protein of unknown function; induced in core stress response; Gcn2 and Gcn4 regulated; flow model biofilm induced; Spider biofilm induced	-2.58673	2.29285E-77
orf19.3447	Ortholog(s) have mannosyltransferase activity, role in GPI anchor biosynthetic process, protein processing, ubiquitin-dependent ERAD pathway and endoplasmic reticulum, glycosylphosphatidylinositol-mannosyltransferase I complex localization	-2.59304	6.07613E-22
SAC1	Putative phosphatidylinositol phosphate (PtdInsP) phosphatase, involved in cell wall integrity and morphogenesis	-2.597927	1.36833E-27
AAT22	Aspartate aminotransferase; nitrogen metabolism; similar but not orthologous to S. cerevisiae Aat2; clade-associated gene expression; protein levels decrease in stationary phase yeast; mutant is viable; flow model biofilm repressed	-2.601001	2.64151E-46
orf19.5763	Has domain(s) with predicted oxidoreductase activity and role in metabolic process	-2.6096	1.36387E-16
MVB12	ESCRT-I complex subunit with a role in multivesicular body (MVB) trafficking; mutant displays growth defect on alkaline medium; Hap43p-repressed gene	-2.611201	3.64335E-11
orf19.7170	Ortholog of C. dubliniensis CD36 : Cd36_73740, Candida tropicalis NEW ASSEMBLY : CTRG1_05151, Candida tropicalis MYA-3404 : CTRG_05151 and Candida albicans WO-1 : CAWG_05736	-2.617068	0.000411366
ACB1	Protein similar to a region of acyl-coenzyme-A-binding protein; amphotericin B repressed; regulated by Nrg1, Tup1; Hap43-induced	-2.617664	5.3492E-10

ARG83	GAL4-like Zn(II)2Cys6 transcription factor; clade-associated expression; null shows abnormal regulation of invasive colony growth, is unable to utilize proline as a nitrogen source; flow model biofilm induced	-2.618745	7.57985E-09
orf19.5524	Ortholog of C. dubliniensis CD36 : Cd36_62790, C. parapsilosis CDC317 : CPAR2_601680, C. auris B8441 : B9J08_001637 and Candida tenuis NRRL Y-1498 : cten_CGOB_00252	-2.620773	0.014218415
SSN8	Ortholog of S. cerevisiae Ssn8; a component of RNA polymerase II holoenzyme; mutants are viable and are sensitive to hydrogen peroxide medium	-2.623785	1.91407E-10
orf19.4864	Ortholog(s) have acylglycerol lipase activity, role in triglyceride metabolic process and lipid droplet, membrane localization	-2.628669	2.35209E-28
orf19.6264.3	Ortholog(s) have role in endoplasmic reticulum to Golgi vesicle-mediated transport and COPII-coated ER to Golgi transport vesicle, Golgi apparatus, endoplasmic reticulum localization	-2.631704	1.16362E-35
NAT4	Putative histone acetyltransferase; involved in regulation of white-opaque switch; early-stage flow model biofilm induced; Spider biofilm induced	-2.633878	1.36564E-19
orf19.4521	S. cerevisiae ortholog Env9 has similarity to oxidoreductases and is proposed to have vacuolar functions, found in lipid particles; hyphal-induced expression	-2.636854	6.11045E-12
orf19.5854.1	Ortholog of Candida albicans WO-1 : CAWG_02725	-2.640183	1.36473E-05
DAL5	Allantoate permease; nitrogen catabolite repressed, induced in absence of preferred N sources; nitrogen source regulation requires Gat1; possibly essential gene (by UAU1 method); Hap43-repressed	-2.642296	2.78645E-05
orf19.916	Putative protein involved in apoptosis; Spider biofilm induced	-2.649627	8.45255E-45
orf19.2838	Protein of unknown function; mutation confers hypersensitivity to amphotericin B; flow model biofilm induced	-2.650013	1.97714E-09
TRY6	Helix-loop-helix transcription factor; regulator of yeast form adherence; required for yeast cell adherence to silicone substrate; Spider and F-12/CO2 biofilm induced; repressed by alpha pheromone in SpiderM medium	-2.652667	0.000416941
orf19.4080	Putative ribonuclease H2 subunit; required for RNase H2 activity; repressed in Spider biofilms by Bcr1, Tec1, Brg1, Rob1	-2.657577	3.50811E-26
orf19.2893	Protein of unknown function; regulation correlates with clinical development of fluconazole resistance; transcript is upregulated in an RHE model of oral candidiasis	-2.660965	2.18997E-11
orf19.5066	Putative pre-60S pre-ribosomal particle subunit; essential gene; S. cerevisiae ortholog RRP17 is essential; Hap43p-induced; rat catheter biofilm induced	-2.662708	4.39806E-10
HGT19	Putative MFS glucose/myo-inositol transporter; 20 member family; 12 transmembrane segments, extended N terminus;	-2.669584	2.07019E-36

	expressed in rich medium; Hap43, phagocytosis, rat catheter, Spider and flow model biofilm induced		
orf19.6480	Ortholog(s) have role in UDP-glucose transmembrane transport	-2.67502	1.35514E-15
orf19.1539	Protein of unknown function; F-12/CO2 early biofilm induced	-2.677907	2.64571E-06
orf19.6315	Ortholog of C. dubliniensis CD36 : Cd36_30140, C. parapsilosis CDC317 : CPAR2_204040, C. auris B8441 : B9J08_001978 and Candida tenuis NRRL Y-1498 : CANTEDRAFT_114703	-2.677955	1.248E-07
FCY24	Putative transporter; more similar to S. cerevisiae Tpn1, which is a vitamin B6 transporter, than to purine-cytosine permeases; transcription is regulated by Nrg1; Spider biofilm induced	-2.683901	2.75599E-42
OPT1	Oligopeptide transporter; transports 3-to-5-residue peptides; alleles are distinct, one has intron; suppresses S. cerevisiae ptr2-2 mutant defects; induced by BSA or peptides; Stp3p, Hog1p regulated; flow model biofilm induced	-2.68431	3.5172E-89
GAP2	General broad specificity amino acid permease; ketoconazole, flucytosine repressed; Ssy1-dependent histidine induction; regulated by Nrg1, Tup1; colony morphology-related gene regulation by Ssn6; Spider and flow model biofilm induced	-2.691827	1.34967E-31
orf19.7345	Ortholog(s) have ATP binding activity	-2.69202	2.45681E-17
PGA26	GPI-anchored adhesin-like protein of the cell wall; role in cell wall integrity; required for normal virulence; induced in high iron and during cell wall regeneration; Hap43-repressed	-2.6935	2.39426E-06
orf19.50	Ortholog(s) have role in SCF-dependent proteasomal ubiquitin-dependent protein catabolic process, cellular response to DNA damage stimulus, cellular response to methylmercury	-2.695219	2.4285E-16
SEC5	Predicted exocyst component; ortholog of S. cerevisiae Sec5p; merged with orf19.75 in Assembly 21	-2.702586	3.63735E-13
orf19.4791	Protein of unknown function; Spider biofilm induced	-2.706269	7.5179E-05
orf19.7316	Putative phytanoyl-CoA dioxygenase family protein; mutation confers hypersensitivity to 5-fluorocytosine (5-FC), 5-fluorouracil (5-FU), and tubercidin (7-deazaadenosine); induced by nitric oxide	-2.710782	6.78655E-07
HSP21	Small heat shock protein; role in stress response and virulence; fluconazole-downregulated; induced in cyr1 or ras1 mutant; stationary phase enriched protein; detected in some, not all, biofilm extracts; Spider biofilm induced	-2.715836	8.89943E-27
PGA45	Putative GPI-anchored cell wall protein; repressed in core caspofungin response; Hog1-induced; regulated by Ssn6; Mob2-dependent hyphal regulation; flow model biofilm induced	-2.71743	2.73848E-76

orf19.5129	Ortholog of C. dubliniensis CD36 : Cd36_72910, C. parapsilosis CDC317 : CPAR2_704100, C. auris B8441 : B9J08_002713 and Candida tenuis NRRL Y-1498 : CANTEDRAFT_113193	-2.718718	1.07232E-14
orf19.1229	Ortholog(s) have role in protein export from nucleus, snRNA import into nucleus and nuclear envelope, nuclear periphery, nucleus localization	-2.73681	2.97446E-05
orf19.5003	Ortholog(s) have GTPase regulator activity and cytoplasm, nucleus localization	-2.742019	1.22756E-32
ALD6	Putative aldehyde dehydrogenase; stationary phase enriched protein; expression regulated upon white-opaque switch; rat catheter biofilm induced; rat catheter and Spider biofilm induced	-2.742903	0.007130234
VMA5	Putative vacuolar H(+)-ATPase; plasma membrane localized; rat catheter biofilm repressed	-2.743067	5.59718E-46
ALD5	NAD-aldehyde dehydrogenase; decreased expression in fluconazole-resistant isolate, or in hyphae; biofilm induced; fluconazole-downregulated; protein abundance is affected by URA3 expression in the CAI-4 strain; stationary phase enriched	-2.748801	2.66375E-46
AXL1	Putative endoprotease; induced by alpha factor; transcript is upregulated in an RHE model of oral candidiasis and in clinical isolates from HIV+ patients with oral candidiasis	-2.752246	1.33383E-18
orf19.6316	Predicted membrane transporter, member of the L-amino acid transporter-3 (LAT3) family, major facilitator superfamily (MFS)	-2.762894	4.51494E-09
MNN24	Alpha-1,2-mannosyltransferase; required for normal cell wall mannan content	-2.766044	7.4835E-30
orf19.1054	Ortholog(s) have role in endoplasmic reticulum tubular network membrane organization, nuclear envelope organization, nuclear pore organization	-2.76792	3.47627E-35
orf19.2514	Predicted methyltransferase; rat catheter biofilm induced	-2.771584	7.67133E-17
LPG20	Aldo-keto reductase family protein; similar to aryl alcohol dehydrogenases; osmotic stress-induced, correlates with overexpression of MDR1 in fluconazole-resistant isolate; stationary phase enriched protein	-2.772576	1.08319E-38
orf19.4748	Putative U2B'' component of the U2 snRNP, involved in splicing; contains an RNA recognition motif (RRM); ortholog of S. cerevisiae MSL1; Hap43p-induced gene	-2.77259	0.000430313
orf19.1137	Thymidylate kinase of unknown role; forms a dimer; potential target for antifungal drugs	-2.778042	4.223E-17
orf19.3151	Has domain(s) with predicted catalytic activity and role in cellular metabolic process	-2.77961	1.00877E-16
orf19.1476	Ortholog(s) have mRNA (N6-adenosine)-methyltransferase activity and role in mRNA methylation, meiotic DNA	-2.779746	1.57013E-05

	replication initiation, meiotic cell cycle, negative regulation of pseudohyphal growth		
RHB1	Putative small G protein from the Ras superfamily involved in cell wall integrity and control of filamentous growth under nitrogen starvation; involved in activation of TOR1C during starvation response	-2.780316	5.80481E-15
ALS2	ALS family protein; role in adhesion, biofilm formation, germ tube induction; expressed at infection of human buccal epithelial cells; putative GPI-anchor; induced by ketoconazole, low iron and at cell wall regeneration; regulated by Sfu1p	-2.792433	1.90464E-25
IPK1	Ortholog of S. cerevisiae/S. pombe Ipk1; an inositol pentakisphosphate 2-kinase, a nuclear protein required for synthesis of 1,2,3,4,5,6-hexakisphosphate; Spider biofilm induced	-2.794301	7.09196E-35
orf19.746	Has domain(s) with predicted role in protein transport, vacuolar transport	-2.796747	1.18525E-10
orf19.5608	RNA polymerase III subunit; Spider biofilm induced	-2.802748	6.35741E-12
orf19.6804	Ortholog(s) have role in SRP-dependent cotranslational protein targeting to membrane and signal recognition particle, endoplasmic reticulum targeting localization	-2.808881	1.17099E-36
SMC2	Protein similar to S. cerevisiae Smc2p, which is a component of the condensin complex involved in mitotic chromosome condensation; induced under hydroxyurea treatment	-2.811524	6.20307E-13
MRP7	Mitochondrial ribosomal protein of the large subunit; rat catheter biofilm induced	-2.817177	5.75673E-31
QCE1	Mitochondrial protein required for expression of respiratory chain complex III (coenzyme Q:cytochrome c oxidoreductase); Hap43-induced gene; Spider biofilm induced	-2.819834	6.73347E-10
orf19.7341.1	Protein of unknown function; Spider biofilm induced	-2.820575	3.71518E-09
orf19.1276	Protein with a selenoprotein domain and a thioredoxin-like fold domain; similar to S. cerevisiae Vhs3p, which is a putative phosphopantothenoylcysteine decarboxylase	-2.822235	1.39571E-10
ERD1	Putative membrane protein required for the retention of lumenal endoplasmic reticulum proteins; rat catheter biofilm induced	-2.828015	3.91323E-20
HGT1	High-affinity MFS glucose transporter; induced by progesterone, chloramphenicol, benomyl; likely essential for growth; protein newly produced during adaptation to the serum; rat catheter and Spider biofilm induced	-2.832146	8.7546E-32
orf19.1050	Ortholog of S. cerevisiae : YMR087W, C. glabrata CBS138 : CAGL0J01397g, C. dubliniensis CD36 : Cd36_03920, C. parapsilosis CDC317 : CPAR2_107150 and C. auris B8441 : B9J08_00513	-2.83284	1.01988E-09

CGR1	Negative regulator of yeast-form growth; HSP70 family member; induced by growth cessation at yeast-hyphal transition or in planktonic growth; physically interacts with Msi3p; similar to rat anti-aging gene, SMP30, stationary phase enriched	-2.835638	2.77978E-38
orf19.3444	Predicted membrane transporter, member of the drug:proton antiporter (14 spanner) (DHA2) family, major facilitator superfamily (MFS); Hap43p-repressed gene	-2.839327	8.59019E-05
orf19.3430	Plasma membrane-associated protein; physically interacts with TAP-tagged Nop1p	-2.843719	4.93269E-10
orf19.2749	BTB/POZ domain protein; induced by Mnl1 under weak acid stress; flow model biofilm induced; Spider biofilm induced	-2.844545	0.001444961
orf19.4517	Protein of unknown function; Hap43-induced gene	-2.862108	8.1144E-54
orf19.2928	Has domain(s) with predicted role in retrograde vesicle-mediated transport, Golgi to endoplasmic reticulum and integral component of membrane localization	-2.871009	6.95958E-31
orf19.670.2	Protein of unknown function; hypoxia, Hap43-repressed; ketoconazole induced; induced in oralpharyngeal candidasis; 16h flow model biofilm repressed, late-stage flow model biofilm induced; rat catheter and Spider biofilm induced	-2.872089	3.39008E-32
orf19.5250	Ortholog of C. dubliniensis CD36 : Cd36_11370, C. parapsilosis CDC317 : CPAR2_207480, C. auris B8441 : B9J08_001721 and Candida tenuis NRRL Y-1498 : CANTEDRAFT_120384	-2.873569	1.86059E-33
orf19.3437	Has domain(s) with predicted serine-type endopeptidase activity and role in proteolysis	-2.889946	3.98161E-06
orf19.55	Ortholog(s) have role in cellular zinc ion homeostasis	-2.891511	3.88105E-39
ERG28	Ortholog(s) have protein-macromolecule adaptor activity, role in demethylation, ergosterol biosynthetic process and endoplasmic reticulum membrane localization	-2.902255	2.20513E-29
ATP7	Putative subunit of the F1F0-ATPase complex; colony morphology-related gene regulation by Ssn6; farnesol, macrophage-downregulated protein abundance; protein present in exponential and stationary yeast growth phases; Hap43-induced	-2.905549	5.01341E-36
orf19.6822	Ortholog(s) have NEDD8 transferase activity, cullin family protein binding, protein-macromolecule adaptor activity, ubiquitin conjugating enzyme binding, ubiquitin-like protein binding activity	-2.91089	2.73931E-25
orf19.1505	Ortholog of S. cerevisiae : YBR138C, C. dubliniensis CD36 : Cd36_16750, C. parapsilosis CDC317 : CPAR2_212450, C. auris B8441 : B9J08_002176 and Candida tenuis NRRL Y-1498 : CANTEDRAFT_92365	-2.921507	2.86997E-29
AAT1	Aspartate aminotransferase; soluble protein in hyphae; macrophage-induced protein; alkaline upregulated;	-2.921951	2.24616E-53

	amphotericin B repressed; gene used for strain identification by multilocus sequence typing; farnesol-, Hap43p-induced; GlcNAc-induced		
orf19.2669	ORF in retrotransposon Tca4; similar to Pol region of retrotransposons encoding reverse transcriptase, protease, integrase; downstream of RHD2, similar to the Gag region encoding nucleocapsid-like protein; rat catheter biofilm induced	-2.927319	1.04103E-46
orf19.762	Ortholog of C. parapsilosis CDC317 : CPAR2_105580, C. dubliniensis CD36 : Cd36_04550, Lodderomyces elongisporus NRLL YB-4239 : LELG_03202 and Candida metapsilosis : CMET_5254	-2.929611	0.004129335
PGA10	GPI anchored membrane protein; utilization of hemin and hemoglobin for Fe in host; Rim101 at ph8/hypoxia/ketoconazole/ciclopirox/hypha-induced; required for RPMI biofilm formation, Bcr1-induced in a/a biofilm; rat catheter biofilm repressed	-2.934131	4.60343E-60
PET111	Mitochondrial translational activator specific for the COX2 mRNA, required for expression of mitochondrial respiratory chain complex IV (cytochrome c oxidase); Hap43p-induced gene	-2.941393	6.30304E-12
orf19.3460	Protein of unknown function; mRNA binds She3; transcript regulated upon yeast-hypha switch; induced in oralpharyngeal candidasis	-2.945841	7.03519E-09
orf19.5701	Ortholog(s) have role in DNA replication initiation, chromosome segregation, establishment of mitotic sister chromatid cohesion, mitotic spindle assembly checkpoint signaling	-2.945875	1.20361E-05
IFD6	Aldo-keto reductase; similar to aryl alcohol dehydrogenases; protein increase correlates with MDR1 overexpression (not CDR1 or CDR2) in fluconazole-resistant clinical isolates; farnesol regulated; possibly essential; Spider biofilm induced	-2.955235	2.94957E-24
orf19.2442	Ortholog of S. cerevisiae Shg1; a COMPASS (Set1C) complex subunit that methylates histone H3 on lysine 4 and is required in transcriptional silencing near telomeres; repressed in an azole-resistant strain overexpressing MDR1	-2.956163	6.72015E-11
TNA1	Putative nicotinic acid transporter; detected at germ tube plasma membrane by mass spectrometry; transcript induced upon phagocytosis by macrophage; rat catheter biofilm induced	-2.958318	6.55795E-16
orf19.1314	Protein of unknown function; planktonic growth-induced gene	-2.961378	1.38058E-38
orf19.104	Protein of unknown function; induced by alpha pheromone in SpiderM medium	-2.963616	0.001650758

orf19.199	Protein of unknown function; hyphal-induced expression; upregulated in a cyr1 null mutant	-2.966138	1.02859E-27
IPL1	Putative Aurora kinase; Hap43-induced; induced during planktonic growth; possibly an essential gene, disruptants not obtained by UAU1 method	-2.966286	4.9079E-16
SCP1	Putative cortical actin cytoskeleton protein; constitutive expression independent of MTL or white-opaque status	-2.982337	4.64356E-05
orf19.4595	Ortholog of C. dubliniensis CD36 : Cd36_41860, C. parapsilosis CDC317 : CPAR2_400440, C. auris B8441 : B9J08_002205 and Candida tenuis NRRL Y-1498 : CANTEDRAFT_103033	-2.9826	1.24307E-25
CAN2	Basic amino acid permease; arginine metabolism; regulated by Nrg1/Tup1; caspofungin, flucytosine induced; colony morphology-related regulation by Ssn6; Hap43-repressed; rat catheter and Spider biofilm induced; promoter bound by Efg1	-2.985339	1.14861E-47
orf19.6920	Protein of unknown function; induced during chlamydospore formation in both C. albicans and C. dubliniensis; Hap43-induced gene; Spider biofilm induced; F-12/CO2 early biofilm induced	-2.994202	0.001124655
orf19.5573	Protein of unknown function; expression downregulated in an ssr1 null mutant	-3.001483	0.000565839
orf19.6359	Ortholog(s) have role in mitochondrial respiratory chain complex assembly, proteolysis and mitochondrial inner membrane, mitochondrial inner membrane peptidase complex localization	-3.001524	2.83914E-07
DSL1	Protein similar to S. cerevisiae Dsl1p, which is a member of the t-SNARE complex of the endoplasmic reticulum	-3.001755	5.23406E-34
orf19.1590	Ortholog of C. dubliniensis CD36 : Cd36_18970, C. parapsilosis CDC317 : CPAR2_212800, Debaryomyces hansenii CBS767 : DEHA2F08690g and Candida guilliermondii ATCC 6260 : PGUG_01488	-3.003062	7.74592E-18
orf19.3351	Protein of unknown function; Hap43-induced; Spider biofilm induced	-3.008226	0.000105459
orf19.446.1	Protein with a NADH-ubiquinone oxidoreductase B18 subunit domain; gene has intron	-3.024027	6.6167E-06
orf19.2516	Has domain(s) with predicted role in cell redox homeostasis	-3.030296	3.7383E-12
orf19.2541	Ortholog(s) have 3'-5'-exodeoxyribonuclease activity, endonuclease activity and role in apoptotic DNA fragmentation, cellular response to oxidative stress	-3.037849	1.25113E-16
orf19.3762	Ortholog of C. dubliniensis CD36 : Cd36_11720, C. parapsilosis CDC317 : CPAR2_201890, C. auris B8441 : B9J08_003468 and Candida tenuis NRRL Y-1498 : CANTEDRAFT_91948	-3.038933	1.04501E-10
MNT4	Predicted alpha-1,3-mannosyltransferase with a role in protein glycosylation; induced in low iron	-3.043741	5.98998E-17

orf19.376	Protein of unknown function; Hap43-repressed; Spider biofilm induced	-3.05555	4.35463E-86
orf19.7022	Ortholog of C. dubliniensis CD36 : Cd36_70850, C. auris B8441 : B9J08_003277, Candida tenuis NRRL Y-1498 : cten_CGOB_00183 and Debaryomyces hansenii CBS767 : DEHA2F22462g	-3.055689	6.83437E-17
orf19.697	Ortholog of S. cerevisiae : YSC83, C. glabrata CBS138 : CAGL0A02134g, C. dubliniensis CD36 : Cd36_32160, C. parapsilosis CDC317 : CPAR2_203070 and C. auris B8441 : B9J08_004564	-3.057258	1.44735E-48
orf19.4191.1	Ortholog(s) have ubiquitin-ubiquitin ligase activity and role in free ubiquitin chain polymerization, protein polyubiquitination, ubiquitin-dependent ERAD pathway, ubiquitin-dependent protein catabolic process	-3.069258	5.363E-21
NIP1	Putative translation initiation factor; mutation confers hypersensitivity to roridin A and verrucarin A; genes encoding ribosomal subunits, translation factors, and tRNA synthetases are downregulated upon phagocytosis by murine macrophage	-3.0836	2.58756E-67
GPI14	Catalytic subunit of glycosylphosphatidylinositol-alpha 1,4 mannosyltransferase I, involved in GPI anchor biosynthesis; regulated by Tsa1p, Tsa1Bp under H2O2 stress conditions	-3.088385	6.92225E-37
orf19.3558	Has domain(s) with predicted role in transport and integral component of membrane localization	-3.089597	5.1046E-40
TEP1	Putative protein phosphatase of the PTP family (tyrosine-specific), similar to S. cerevisiae Tep1p	-3.103951	3.15333E-10
orf19.6805	Protein of unknown function; Spider biofilm induced	-3.106369	9.63458E-11
orf19.1121	Ortholog of C. dubliniensis CD36 : Cd36_53470, Candida tenuis NRRL Y-1498 : CANTEDRAFT_93324, Candida tropicalis NEW ASSEMBLY : CTRG1_05538 and Spathaspora passalidarum NRRL Y-27907 : SPAPADRAFT_55715	-3.108955	6.31621E-22
GLC3	Putative 1,4-glucan branching enzyme; fluconazole-induced; colony morphology-related gene regulation by Ssn6; stationary phase enriched protein	-3.111985	2.63835E-73
CDC43	Beta subunit of heterodimeric protein geranylgeranyltransferase type I; GGTase I enzyme binds zinc, is Mg-dependent; Cdc42p is GGTase I substrate	-3.112053	2.05767E-10
GSG1	Putative subunit of the TRAPP complex; involved in targeting of ER-to-Golgi transport vesicles; flow model biofilm induced	-3.117238	1.3825E-15
orf19.4656	Ortholog of C. dubliniensis CD36 : Cd36_41300, C. parapsilosis CDC317 : CPAR2_400390, C. auris B8441 : B9J08_000767 and Candida tenuis NRRL Y-1498 : CANTEDRAFT_94704	-3.126692	5.44617E-08
RBT5	GPI-linked cell wall protein; hemoglobin utilization; Rfg1, Rim101, Tbf1, Fe regulated; Sfu1, Hog1, Tup1, serum, alkaline	-3.128952	3.14415E-43

	pH, antifungal drugs, geldamycin repressed; Hap43 induced; required for RPMI biofilms; Spider biofilm induced		
orf19.6966	Ortholog(s) have choline kinase activity, ethanolamine kinase activity and role in phosphatidylcholine biosynthetic process, phosphatidylethanolamine biosynthetic process	-3.130573	2.5231E-37
DPP3	Protein similar to S. cerevisiae pyrophosphate phosphatase Dpp1; required for farnesol biosynthesis; repressed by 17-beta-estradiol, ethynyl estradiol; Spider biofilm induced	-3.13128	1.04969E-51
JEN2	Dicarboxylic acid transporter; regulated by glucose repression; induced by Rgt1; disruptants not obtained by UAU1 method; rat catheter and Spider biofilm induced	-3.131406	9.93295E-07
ERG11	Lanosterol 14-alpha-demethylase; cytochrome P450 family; role in ergosterol biosynthesis; target of azole antifungals; may contribute to drug resistance; azole or flow model biofilm induced; drug treated biofilm induced; hypoxia regulated	-3.134114	1.8345E-68
orf19.3686	Ortholog(s) have protein domain specific binding activity, role in mitochondrial proton-transporting ATP synthase complex assembly and mitochondrion localization	-3.136501	3.67474E-39
orf19.7202	Ortholog(s) have role in protein retention in ER lumen, retrograde vesicle-mediated transport, Golgi to endoplasmic reticulum	-3.143061	1.43672E-19
orf19.7297	Putative cystathionine gamma-synthase; decreased levels in stationary phase cultures; Hog1p-induced; Gcn4p-regulated	-3.144316	2.90255E-46
orf19.4474	Ortholog(s) have proteasome binding activity and role in cellular response to arsenic-containing substance, proteasome-mediated ubiquitin-dependent protein catabolic process	-3.154673	4.03623E-32
DAL52	Putative allantoate permease; mutant is viable; similar but not orthologous to S. cerevisiae Dal5	-3.155795	0.02179403
CHO2	Phosphatidyl-ethanolamine N-methyltransferase; fungal-specific (no human or murine homolog); amphotericin B repressed; Hap43p-induced gene	-3.158295	2.28727E-37
orf19.3601	Has domain(s) with predicted ATP binding, ATPase, nucleoside-triphosphatase activity, nucleotide binding activity	-3.160773	4.81087E-11
DIE2	Ortholog(s) have dolichyl pyrophosphate Glc2Man9GlcNAc2 alpha-1,2-glucosyltransferase activity, role in protein N-linked glycosylation and endoplasmic reticulum membrane localization	-3.166116	1.57211E-13
orf19.7038	Ortholog(s) have phosphatidylinositol-3-phosphate binding activity, role in plasma membrane tubulation, protein targeting to vacuole, retrograde transport, endosome to Golgi and cytoplasm, endosome, nucleus localization	-3.177186	5.52844E-23

MET8	Putative bifunctional dehydrogenase and ferrochelatase with a predicted role in siroheme biosynthesis; regulated by Gcn2p and Gcn4p	-3.17953	1.17846E-06
orf19.725	Ortholog of C. dubliniensis CD36 : Cd36_31880, C. parapsilosis CDC317 : CPAR2_702220, C. auris B8441 : B9J08_003984 and Candida tenuis NRRL Y-1498 : CANTEDRAFT_115848	-3.181682	2.36667E-11
orf19.714	Ortholog(s) have GTPase activity	-3.195245	4.59985E-11
orf19.4569	Ortholog of C. dubliniensis CD36 : Cd36_42100, Debaryomyces hansenii CBS767 : DEHA2C14850g, Pichia stipitis Pignal : PICST_52615 and Candida tropicalis MYA-3404 : CTRG_00173	-3.195551	0.000367765
SGA1	Putative glucoamylase; induced in oralpharyngeal candidasis; rat catheter and Spider biofilm induced	-3.196583	2.16783E-10
orf19.1841	Protein of unknown function; Hap43-induced gene	-3.207778	8.46919E-08
LAP3	Putative aminopeptidase; positively regulated by Sfu1; clade-associated gene expression; virulence-group-correlated expression; induced by alpha pheromone in SpiderM medium; Hap43-induced; Spider and flow model biofilm induced	-3.20779	3.28262E-16
orf19.252	Protein of unknown function; S. cerevisiae ortholog Fmp37 which localizes to mitochondria; Hap43-repressed; Spider biofilm repressed	-3.21425	2.67504E-46
UBP8	Putative ubiquitin-specific protease, involved in histone H2B deubiquitination during hyphal development	-3.214977	1.04373E-12
orf19.7344	Ortholog(s) have DNA binding, chromatin binding, histone deacetylase activity and role in chromosome segregation, gene silencing by RNA, histone deacetylation, negative regulation of transcription by RNA polymerase II	-3.223077	1.11599E-23
GPM2	Putative phosphoglycerate mutase; repressed in hyphae; macrophage/pseudohyphal-repressed; induced by high levels of peroxide stress, farnesol; flow model biofilm induced; rat catheter and Spider biofilm repressed	-3.232199	6.25881E-52
VMA22	Ortholog(s) have unfolded protein binding activity and role in vacuolar acidification, vacuolar proton-transporting V-type ATPase complex assembly	-3.236234	7.29336E-05
orf19.2369	Ortholog(s) have ATP binding, DNA replication origin binding activity	-3.243732	4.52665E-20
orf19.2870	Protein of unknown function; rat catheter and Spider biofilm induced	-3.249525	2.46217E-07
orf19.6551	Ortholog(s) have SNAP receptor activity, role in Golgi vesicle transport, vesicle fusion and Golgi medial cisterna, SNARE complex localization	-3.255928	5.29713E-11
orf19.6553	Membrane-localized protein of unknown function; possibly secreted; fluconazole-induced	-3.262551	4.69389E-58

ABP2	Putative alpha-actinin-like protein; induced by alpha pheromone in SpiderM medium	-3.263176	5.82186E-11
orf19.6601	Protein of unknown function; rat catheter and flow model biofilm induced	-3.263922	5.50016E-26
orf19.2829	Ortholog(s) have role in protein transport	-3.264894	7.65947E-11
orf19.2939	Ortholog(s) have role in mitochondrial cytochrome c oxidase assembly and mitochondrion localization	-3.265648	3.15207E-20
orf19.2828	Ortholog(s) have alpha-tubulin binding, microtubule binding activity, role in cytoplasmic microtubule organization, post-chaperonin tubulin folding pathway, protein folding and cytoplasm localization	-3.269546	8.94858E-36
orf19.4617	Predicted peptide alpha-N-acetyltransferase; flow model biofilm induced	-3.277827	6.81433E-50
orf19.6813	Protein of unknown function; Hap43-induced gene	-3.290471	1.49489E-13
CRG1	Methyltransferase involved in sphingolipid homeostasis, methylates a drug cantharidin; decreased expression in hyphae compared to yeast; expression regulated during planktonic growth; flow model biofilm induced; Hap43-repressed gene	-3.30111	2.73287E-24
orf19.5611	Predicted 3-methylbutanol:NAD(P) oxidoreductase and methylglyoxal reductase (NADPH-dependent); role in ergosterol metabolic process; early stage flow model biofilm induced; Spider biofilm induced	-3.316369	1.34368E-07
orf19.3627	Ortholog of C. dubliniensis CD36 : Cd36_22640, C. parapsilosis CDC317 : CPAR2_406910, C. auris B8441 : B9J08_004017 and Candida tenuis NRRL Y-1498 : CANTEDRAFT_104937	-3.3494	0.017942006
orf19.6458.1	Ortholog(s) have U6 snRNA binding activity and role in P-body assembly, mRNA splicing, via spliceosome	-3.349526	3.18592E-18
orf19.5227	Chaperone component; involved in assembly of alpha subunits into the 20S proteasome; flow model biofilm induced	-3.356069	0.00023994
IFE2	Putative alcohol dehydrogenase; yeast-enriched transcript; Efg1-regulated; induced by prostaglandins, Hog1, fluconazole; rat catheter biofilm induced	-3.418676	1.23235E-54
ARG8	Putative acetylornithine aminotransferase; Gcn2, Gcn4 regulated; rat catheter biofilm induced; Spider biofilm induced	-3.420911	1.50036E-24
BET4	Ortholog(s) have Rab geranylgeranyltransferase activity and role in endoplasmic reticulum to Golgi vesicle-mediated transport, protein geranylgeranylation, protein targeting to membrane	-3.422151	1.3541E-18
ZCF14	Putative Zn(II)2Cys6 transcription factor; caspofungin induced	-3.422409	3.03321E-08
YKE2	Possible heterohexameric Gim/prefoldin protein complex subunit; role in folding alpha-tubulin, beta-tubulin, and actin; transcript induced by yeast-to-hypha switch; regulated by Nrg1, Tup1; Spider and flow model biofilm induced	-3.42339	4.69498E-53

FAD2	Delta-12 fatty acid desaturase, involved in production of linoleic acid, which is a major component of membranes	-3.424073	7.26385E-95
CCE1	Putative Holliday junction resolving enzyme; similar to S. cerevisiae Cce1p	-3.439599	1.07505E-05
orf19.4953	Putative ATPase; predicted role in ER-associated protein catabolism; induced during chlamydospore formation in both C. albicans and C. dubliniensis; rat catheter biofilm repressed	-3.451206	7.37373E-49
orf19.6443	Protein with an enoyl-CoA hydratase related domain; Spider biofilm induced	-3.466287	1.56677E-05
orf19.3235	Putative F-actin capping protein subunit alpha; possibly an essential gene, disruptants not obtained by UAU1 method	-3.491005	2.98313E-54
MET18	Putative protein with a predicted role in nucleotide excision repair (NER) and RNA polymerase II (RNAP II) transcription; Plc1p-regulated	-3.504532	5.73657E-58
DAD1	Subunit of the Dam1 (DASH) complex, which acts in chromosome segregation by coupling kinetochores to spindle microtubules	-3.510118	0.007707999
orf19.1529	Protein of unknown function; macrophage-induced gene	-3.511253	0.011081936
CWH8	Putative dolichyl pyrophosphate (Dol-P-P) phosphatase; ketoconazole-induced; expression is increased in a fluconazole-resistant isolate; clade-associated gene expression; Hap43p-induced gene	-3.513646	1.47502E-67
orf19.7204	Has domain(s) with predicted catalytic activity, nitronate monooxygenase activity	-3.515293	4.26221E-29
orf19.5042	Ortholog(s) have role in maintenance of rDNA, regulation of exit from mitosis	-3.520982	0.001563371
orf19.7502	Protein of unknown function; Hap43-induced gene; upregulated in a cyr1 null mutant; Spider biofilm induced	-3.544877	3.19491E-46
orf19.35	Predicted kinase; rat catheter, flow model, Spider biofilm induced	-3.544945	5.43468E-18
orf19.3872	Protein of unknown function; oral infection induced; mutants have reduced capacity to damage oral epithelial cells; rat catheter biofilm repressed	-3.549199	7.95532E-59
orf19.1729	Putative transcription factor with zinc finger DNA-binding motif	-3.561325	1.4053E-08
GPX2	Similar to glutathione peroxidase; induced in high iron; alkaline induced by Rim101; induced by alpha factor or interaction with macrophage; regulated by Efg1; caspofungin repressed; Spider biofilm induced	-3.564618	5.53264E-75
orf19.6020	Ortholog(s) have Atg8 ligase activity	-3.577044	2.85422E-27
orf19.5033	Ortholog(s) have Atg8 ligase activity, enzyme activator activity, protein tag activity	-3.586723	2.35783E-06
ERG9	Putative farnesyl-diphosphate farnesyl transferase (squalene synthase); sterol biosynthesis pathway; likely essential for	-3.604274	1.53041E-55

	growth; regulated by fluconazole, lovastatin; amphotericin B, caspofungin repressed; Spider biofilm repressed		
orf19.1495	Ortholog(s) have role in L-methionine salvage from methylthioadenosine	-3.609186	5.68998E-23
orf19.7556	Ortholog of C. dubliniensis CD36 : Cd36_34965, C. parapsilosis CDC317 : CPAR2_200910, C. auris B8441 : B9J08_005167 and Candida tenuis NRRL Y-1498 : CANTEDRAFT_95984	-3.610458	0.000289813
orf19.1485	Mitochondrial ribosomal protein of the large subunit; rat catheter biofilm induced	-3.617101	5.83243E-20
orf19.6612	Putative mitochondrial protein; Hap43p-induced gene	-3.627457	8.13855E-61
mesA	XP_723132.1 Mea1p [Candida albicans SC5314]	-3.627889	2.08813E-25
orf19.6787	Ortholog(s) have cargo receptor activity and role in ascospore formation, axial cellular bud site selection, endoplasmic reticulum to Golgi vesicle-mediated transport	-3.632731	3.25978E-25
MNT3	Mannosyltransferase; transcription elevated in cyr1, chk1, nik1, and sln1 homozygous null mutants; fungal-specific (no human or murine homolog)	-3.641826	3.02947E-24
orf19.3687	Ortholog(s) have unfolded protein binding activity, role in cytoskeleton organization, positive regulation of transcription elongation from RNA polymerase II promoter, protein folding and prefoldin complex localization	-3.64802	8.76124E-07
orf19.2757	Has domain(s) with predicted role in cell redox homeostasis	-3.650996	2.4588E-18
orf19.4658	RING finger and CHY zinc finger domain-containing protein; mutant are viable	-3.653362	1.18486E-16
orf19.335	Sef1p-, Sfu1p-, and Hap43p-regulated gene	-3.655352	1.3066E-29
ERG3	C-5 sterol desaturase; introduces C-5(6) double bond into episterol; some clinical isolates show increased azole resistance and defects in hyphal growth and virulence; Efg1p-repressed; fluconazole-induced	-3.663453	1.48776E-53
orf19.2925	Ortholog(s) have role in protein folding, tubulin complex assembly	-3.666502	3.84973E-07
orf19.7588	Ortholog of S. cerevisiae : RRG7, C. glabrata CBS138 : CAGL0I09680g, C. dubliniensis CD36 : Cd36_35140, C. parapsilosis CDC317 : CPAR2_200670 and Candida tenuis NRRL Y-1498 : CANTEDRAFT_117734	-3.670478	1.98879E-11
ADH5	Putative alcohol dehydrogenase; regulated by white-opaque switch; fluconazole-induced; antigenic in murine infection; regulated by Nrg1, Tup1; Hap43, macrophage repressed, flow model biofilm induced; Spider biofilm induced	-3.682566	2.024E-114
orf19.6606	Ortholog of C. parapsilosis CDC317 : CPAR2_201040, C. auris B8441 : B9J08_001657, Candida tenuis NRRL Y-1498 : CANTEDRAFT_112677 and Debaryomyces hansenii CBS767 : DEHA2E02420g	-3.683154	4.62133E-23

CTN1	Carnitine acetyl transferase; required for growth on nonfermentable carbon sources, not for hyphal growth or virulence in mice; induced in macrophage; macrophage/pseudohyphal-repressed after 16 hr; rat catheter, Spider biofilm induced	-3.686141	0.003962525
orf19.934	Predicted ORF from Assembly 19; removed from Assembly 20; subsequently reinstated in Assembly 21 and merged with orf19.2224, based on comparative genome analysis; flow model biofilm repressed	-3.688457	9.06097E-05
GRE3	Putative D-xylose reductase; antigenic in murine systemic infection; soluble protein in hyphae; induced by farnesol, macrophage interaction and by Mnl1 under weak acid stress; stationary-phase enriched protein; Spider biofilm induced	-3.691563	3.1082E-87
CAK1	Monomeric CDK-activating kinase; functional homolog of S. cerevisiae Cak1p; phosphorylates cyclin-free human CDK2; lacks glycine loop motif; conserved lysine (K36) not required for activity; possibly essential gene (by UAU1 method)	-3.706459	3.77606E-52
GIG1	Protein induced by N-acetylglucosamine (GlcNAc); localized in cytoplasm; mutation causes increased resistance to nikkomycin Z	-3.718952	1.31062E-07
PRP39	Putative component of the U1 snRNP; involved in splicing; Hap43-induced gene; Spider biofilm induced	-3.720222	2.97856E-26
orf19.158	Ortholog of S. cerevisiae Apd1; required for normal localization of actin patches and normal tolerance of sodium ions and hydrogen peroxide; Hap43-induced; Spider biofilm induced	-3.741268	3.47477E-43
orf19.3248	Ortholog of C. dubliniensis CD36 : Cd36_26070, C. parapsilosis CDC317 : CPAR2_803220, C. auris B8441 : B9J08_002999 and Candida tenuis NRRL Y-1498 : CANTEDRAFT_131795	-3.741295	5.77939E-12
orf19.5254	Protein of unknown function; repressed by nitric oxide	-3.750574	6.94902E-05
TGL99	Has domain(s) with predicted role in lipid metabolic process	-3.751321	5.50036E-25
orf19.7032	Ortholog of C. dubliniensis CD36 : Cd36_70790, C. parapsilosis CDC317 : CPAR2_301530, C. auris B8441 : B9J08_005266 and Candida tenuis NRRL Y-1498 : CANTEDRAFT_94947	-3.770173	5.26475E-33
CSP37	Hyphal cell wall protein; role in progression of mouse systemic infection; predicted P-loop, divalent cation binding, N-glycosylation sites; expressed in yeast and hyphae; hyphal downregulated; stationary-phase enriched; GlcNAc-induced	-3.770405	1.3851E-103
orf19.3697	Ortholog(s) have role in mitotic intra-S DNA damage checkpoint signaling, positive regulation of macroautophagy, re-entry into mitotic cell cycle after pheromone arrest and Golgi trans cisterna, endoplasmic reticulum localization	-3.77961	2.86995E-19

COI1	Secreted protein; ciclopirox olamine induced; regulated by Ssn6; induced by nitric oxide independent of Yhb1; Hap43-induced; Spider biofilm repressed	-3.804192	6.68626E-51
orf19.4963	Ortholog(s) have protein carrier activity, unfolded protein binding activity, role in ribosomal large subunit biogenesis and cytoplasm, nucleus localization	-3.828167	2.2924E-23
HSM3	Ortholog(s) have role in mismatch repair, proteasome regulatory particle assembly and cytosol, nucleus localization	-3.828846	8.06468E-66
orf19.1350	Protein with a thioredoxin domain; predicted role in cell redox homeostasis; rat catheter and Spider biofilm induced	-3.832123	3.92691E-14
orf19.2367	Putative protein of unknown function; Hap43-induced; repressed by Rim101; Spider biofilm induced	-3.839919	6.68517E-09
HGT12	Glucose, fructose, mannose transporter; major facilitator superfamily; role in macrophage-induced hyphal growth; detected at germ tube plasma membrane by mass spectrometry; Snf3p-induced; 12 probable transmembrane segments	-3.844221	6.63707E-34
OPT4	Oligopeptide transporter; detected at germ tube plasma membrane; transcript inducede during phagocytosis by macrophages; fungal-specific; Hap43-repressed; merged with orf19.2292 in Assembly 20; rat catheter and Spider biofilm induced	-3.853278	1.84462E-33
OPT3	Oligopeptide transporter; transcript induced by macrophage phagocytosis, BSA or peptides; fluconazole-induced; induced by Rim101 at pH 8; virulence-group-correlated expression; Hap43-repressed; Spider biofilm induced	-3.86555	5.00957E-30
orf19.4680	Possile protease; mutation confers hypersensitivity to toxic ergosterol analog	-3.876087	2.57219E-07
ZWF1	Glucose-6-phosphate dehydrogenase; antigenic in mice; activity induced by O2 or oxidizing agents H2O2, menadione, macrophage; caspofungin repressed; induced in core stress response; regulated by Gcn2, Gcn4; rat catheter biofilm repressed	-3.881247	1.5122E-119
AHP1	Alkyl hydroperoxide reductase; immunogenic; fluconazole-induced; amphotericin B, caspofungin, alkaline repressed; core stress response induced; Ssk1/Nrg1/Tup1/Ssn6/Hog1 regulated; flow model biofilm induced; rat catheter biofilm repressed	-3.889607	3.3261E-178
HGT6	Putative high-affinity MFS glucose transporter; 20 family members; induced in core stress response; fluconazole, oralpharyngeal candidasis induced; flow model biofilm induced; Spider biofilm induced	-3.893653	1.22692E-72
orf19.5532	Protein of unknown function; Spider biofilm induced	-3.922077	0.024534759
orf19.5266	Planktonic growth-induced gene	-3.946939	1.20161E-05

PSF1	Putative GINS complex subunit which is localized to DNA replication origins; implicated in assembly of the DNA replication machinery; flow model biofilm induced	-3.950357	5.82872E-20
PEX11	Putative peroxisomal membrane protein; role in fatty acid oxidation; expression is Tac1-regulated; Hms1p-dependent induction by geldamycin; Spider biofilm induced	-3.978955	1.40792E-27
BMT9	Beta-mannosyltransferase, 9-gene family that includes characterized genes BMT1, BMT2, BMT3, and BMT4 with roles in beta-1,2-mannosylation of cell wall phosphopeptidomannan; regulated by Sef1, Sfu1, Hap43; rat catheter biofilm repressed	-3.982129	4.03311E-08
orf19.6600	Ortholog(s) have phosphatidic acid transfer activity and role in cardiolipin metabolic process, phospholipid translocation, phospholipid transport, positive regulation of phosphatidylcholine biosynthetic process	-3.990117	0.000312192
orf19.810	Ortholog(s) have DNA helicase activity, heteroduplex DNA loop binding activity	-4.011164	1.68429E-27
HRT2	Protein described as having a role in Ty3 transposition; repressed in hyphae; stationary phase enriched protein; rat catheter and Spider biofilm repressed	-4.018051	1.7974E-101
LYP1	Putative permease; amphotericin B induced; flucytosine repressed; possibly an essential gene, disruptants not obtained by UAU1 method	-4.021789	2.13442E-13
TOA2	Putative TFIIA small subunit; protein abundance decreased in CAI4 strain compared to the SC5314 strain, abundance not affected by reintegration of URA3 in CAI4; flucytosine induced; possibly an essential gene (UAU1 method)	-4.027182	2.31793E-28
orf19.3990	Ortholog(s) have DNA-directed 5'-3' RNA polymerase activity, RNA polymerase III activity and role in tRNA transcription by RNA polymerase III, transcription initiation from RNA polymerase III promoter	-4.029747	3.60306E-10
orf19.1049	Predicted NUDIX hydrolase domain; Hap43-induced	-4.049705	1.09318E-06
orf19.4323	Ortholog(s) have phosphatidylinositol-3-phosphate binding activity	-4.0803	0.000730404
ARA1	D-Arabinose dehydrogenase; dehydro-D-arabinono-1,4-lactone synthesis; active on D-arabinose, L-fucose, L-xylose, L-galactose; inhibited by metal ions, thiol group-specific reagents; induced on polystyrene adherence; Spider biofilm induced	-4.083311	3.80425E-67
LAG1	Putative ceramide synthase component; Hap43p-repressed gene; amphotericin B repressed	-4.084329	3.90634E-30
CIP1	Possible oxidoreductase; transcript induced by cadmium but not other heavy metals, heat shock, yeast-hypha switch,	-4.1031	1.95094E-76

	oxidative stress (via Cap1), or macrophage interaction; stationary phase enriched protein; Spider biofilm induced		
orf19.4996	Ortholog of S. cerevisiae : YBL029W, C. glabrata CBS138 : CAGL0F04125g, C. parapsilosis CDC317 : CPAR2_201430, C. auris B8441 : B9J08_000642 and Candida tenuis NRRL Y-1498 : CANTEDRAFT_136724	-4.112005	0.018612756
orf19.4903	Ortholog(s) have N-acetylglucosaminylphosphatidylinositol deacetylase activity	-4.121776	3.31054E-35
orf19.4699	Putative phospholipase of patatin family; similar to S. cerevisiae Tgl3p; predicted Kex2p substrate	-4.134318	1.38424E-32
orf19.1136	Ortholog(s) have AP-2 adaptor complex, clathrin-coated vesicle localization	-4.150152	3.75835E-09
orf19.2797	Has domain(s) with predicted ATP binding, DNA binding, helicase activity, hydrolase activity, nucleic acid binding activity	-4.172061	2.60918E-05
orf19.3698	Protein of unknown function that may function in RNA processing; filament induced	-4.17434	9.63342E-16
orf19.3626	Has domain(s) with predicted asparagine synthase (glutamine-hydrolyzing) activity and role in asparagine biosynthetic process, metabolic process	-4.180938	1.00371E-10
orf19.1789	Ortholog of S. cerevisiae : MRX12, C. glabrata CBS138 : CAGL0J11110g, C. dubliniensis CD36 : Cd36_44970, C. parapsilosis CDC317 : CPAR2_500860 and C. auris B8441 : B9J08_003204	-4.222273	1.81297E-08
orf19.6970	Ortholog of C. dubliniensis CD36 : Cd36_85310, C. parapsilosis CDC317 : CPAR2_807370, C. auris B8441 : B9J08_004442 and Candida tenuis NRRL Y-1498 : CANTEDRAFT_115544	-4.224966	2.32438E-06
orf19.1045	Putative U2 snRNP protein; Hap43p-induced gene; mutation confers hypersensitivity to 5-fluorocytosine (5-FC), 5-fluorouracil (5-FU), and tubercidin (7-deazaadenosine)	-4.227377	5.23109E-21
orf19.1769	Ortholog(s) have G-protein beta/gamma-subunit complex binding, actin binding activity, role in positive regulation of transcription from RNA polymerase II promoter by pheromones, protein folding and cytoplasm localization	-4.258367	6.17947E-43
ARC35	Putative ARP2/3 complex subunit; shows colony morphology-related gene regulation by Ssn6p; mutation confers hypersensitivity to cytochalasin D	-4.271587	2.01962E-76
orf19.5621	Putative protein of unknown function; mutation confers hypersensitivity to amphotericin B; overlaps orf19.5621	-4.282159	3.66483E-50
orf19.1012	Ortholog(s) have role in Golgi to vacuole transport, endosomal transport, vesicle-mediated transport and AP-1 adaptor complex, Golgi apparatus, endosome localization	-4.296881	1.22822E-29
orf19.81	Ortholog(s) have role in exonucleolytic trimming to generate mature 3'-end of 5.8S rRNA from tricistronic rRNA transcript (SSU-rRNA, 5.8S rRNA and LSU-rRNA), more	-4.300527	2.02384E-15

orf19.7443	Ortholog of C. dubliniensis CD36 : Cd36_86510, C. parapsilosis CDC317 : CPAR2_206110, C. auris B8441 : B9J08_002384 and Candida tenuis NRRL Y-1498 : CANTEDRAFT_112184	-4.300914	1.26979E-21
orf19.2228	Ortholog(s) have actin filament binding, actin monomer binding activity	-4.332587	6.97819E-09
orf19.6579	Ortholog of C. dubliniensis CD36 : Cd36_71340, C. parapsilosis CDC317 : CPAR2_301350, C. auris B8441 : B9J08_004871 and Candida tenuis NRRL Y-1498 : CANTEDRAFT_114098	-4.363804	1.11616E-36
orf19.1611	Protein of unknown function; Spider biofilm induced	-4.386396	7.06894E-07
orf19.2761	Putative glycosylphosphatidylinositol (GPI) anchor assembly protein; transposon insertion causes decreased colony wrinkling but does not block true hyphal growth; induced by nitric oxide independent of Yhb1p	-4.402023	4.67567E-31
orf19.3446	Ortholog(s) have role in protein import into nucleus and nucleus localization	-4.402626	5.27459E-13
orf19.5125	Protein of unknown function; induced by ketoconazole; Spider, F-12/CO2 and flow model biofilm induced	-4.409432	6.2406E-20
orf19.2168	Putative sterol deacetylase; flow model biofilm induced; rat catheter biofilm repressed	-4.441317	1.35222E-05
orf19.7360	Ortholog(s) have enzyme activator activity, role in histone deubiquitination, regulation of transcription by RNA polymerase II and DUBm complex, SAGA complex localization	-4.520422	0.00016935
SSH1	Protein with a role in protein translocation across membranes	-4.593941	4.88671E-67
orf19.3483	Putative phosphatidyl glycerol phospholipase C; Plc1-regulated; flow model biofilm induced; Spider biofilm induced	-4.62605	7.51948E-14
orf19.4763	Protein of unknown function; possibly membrane bound; mutants are viable; rat catheter biofilm repressed	-4.638237	1.33398E-30
orf19.68.2	Ortholog of C. dubliniensis CD36 : Cd36_11670, C. parapsilosis CDC317 : CPAR2_201750, C. auris B8441 : B9J08_003474 and Candida tenuis NRRL Y-1498 : CANTEDRAFT_115679	-4.661337	8.88839E-05
MED11	Subunit of the RNA polymerase II mediator complex	-4.684078	3.20853E-22
orf19.6357	Ortholog(s) have protein-containing complex binding activity	-4.685817	1.89806E-39
orf19.5270	Protein of unknown function; rat catheter biofilm induced	-4.689012	3.58511E-16
orf19.6605	Has domain(s) with predicted integral component of membrane localization	-4.689351	6.46314E-41
orf19.1664	Protein of unknown function; expression downregulated in an ssr1 null mutant	-4.695499	1.79998E-10
orf19.915	Protein of unknown function; Spider biofilm induced	-4.697282	9.76852E-31
orf19.6983	Protein of unknown function; Hap43-repressed gene; repressed by nitric oxide; Spider biofilm induced	-4.718079	1.94162E-55
HRQ2	Protein of unknown function; mutants are viable; rat catheter and Spider biofilm induced	-4.738718	9.43232E-22

MTW1	Kinetochore component; amount of Nuf2p and Mtw1p protein detected at each centromere is consistent with a single kinetochore microtubule attachment site	-4.787358	7.33632E-09
orf19.530	Protein with a UV radiation resistance protein/autophagy-related protein 14 domain; Hap43p-induced gene; mutants are viable	-4.895552	5.81096E-16
orf19.716	Protein of unknown function; Hap43-induced; regulated by Nrg1, Tup1; repressed by alpha pheromone in SpiderM medium; Spider biofilm induced; Bcr1-repressed in RPMI a/a biofilms	-4.903992	8.43572E-11
orf19.1484	Ortholog of S, cerevisiae Spc24; a component of the conserved kinetochore-associated Ndc80 complex involved in chromosome segregation, spindle checkpoint activity and kinetochore clustering; Hap43-induced gene	-4.911944	0.003474267
HNM4	Putative choline permease; fungal-specific (no human or murine homolog)	-4.955747	1.39101E-05
orf19.927	Ortholog(s) have role in DNA recombination, positive regulation of transcription by RNA polymerase II, regulation of cell aging, telomere maintenance, telomere maintenance via recombination and EKC/KEOPS complex localization	-4.966888	3.56061E-09
orf19.3625	Has domain(s) with predicted zinc ion binding activity	-4.97456	1.08769E-05
orf19.5905	Protein of unknown function; Hap43-induced; F-12/CO2 early biofilm induced	-4.991207	0.002038612
orf19.4465	Protein of unknown function; Spider biofilm induced	-5.013199	0.000114844
GUP1	Putative O-acyltransferase with a role in glycerol uptake; functionally complements growth of S. cerevisiae gup1 mutant under salt stress; required for normal ergosterol distribution, hyphal growth, biofilm formation	-5.034646	8.61197E-11
SPC19	Essential subunit of the Dam1 (DASH) complex, which acts in chromosome segregation by coupling kinetochores to spindle microtubules	-5.056268	5.14071E-08
PEX22	Putative peroxin	-5.086111	2.26643E-29
MED22	RNA polymerase II mediator complex subunit	-5.094122	5.24714E-06
orf19.2510	Ortholog(s) have role in protein folding, tubulin complex assembly	-5.127168	1.82674E-41
orf19.3448	Protein of unknown function; ketoconazole-repressed	-5.130212	2.52058E-14
orf19.4837.1	Ortholog of C. parapsilosis CDC317 : CPAR2_804880, Candida tenuis NRRL Y-1498 : CANTEDRAFT_111283, Debaryomyces hansenii CBS767 : DEHA2B10604g and Pichia stipitis Pignal : PICST_40454	-5.147899	0.001278609
LIP7	Probable lipase, part of a gene family whose members are differentially expressed during infection; lacks a signal sequence for secretion, unlike other family members; may	-5.147899	0.001381264

	have a role in nutrition or in creating an acidic microenvironment		
orf19.6272	Putative ubiquitin C-terminal hydrolase; regulated by Gcn2p and Gcn4p	-5.149758	1.76173E-56
orf19.1092	Dolichol-P-Man dependent alpha(1-3) mannosyltransferase; role in the synthesis of dolichol-linked oligosaccharide donor for N-linked glycosylation of proteins; rat catheter biofilm repressed	-5.210664	1.85935E-35
orf19.7199	Ortholog(s) have role in posttranslational protein targeting to endoplasmic reticulum membrane and TRC complex, cytoplasm localization	-5.217235	1.30056E-71
orf19.194	Ortholog of C. dubliniensis CD36 : Cd36_19300, C. parapsilosis CDC317 : CPAR2_209720, C. auris B8441 : B9J08_003188 and Candida tenuis NRRL Y-1498 : CANTEDRAFT_114035	-5.299393	5.42108E-39
orf19.699	Protein of unknown function; flow model, rat catheter, Spider biofilm induced	-5.302741	1.55967E-06
ERG20	Putative farnesyl pyrophosphate synthetase involved in isoprenoid and sterol biosynthesis, based on similarity to S. cerevisiae Erg20p; likely to be essential for growth, based on an insertional mutagenesis strategy	-5.340871	1.3623E-127
orf19.2433	Has domain(s) with predicted flap-structured DNA binding activity and role in double-strand break repair via single-strand annealing, removal of nonhomologous ends	-5.398845	1.11233E-06
MOH1	Ortholog of S. cerevisiae Moh1, essential for staionary phase growth; induced by alpha pheromone in SpiderM medium and by Mnl1 under weak acid stress; possibly essential (UAU1 method); flow model biofilm induced; Spider biofilm induced	-5.542076	1.06603E-21
orf19.7473	Ortholog(s) have role in endocytosis and actin cortical patch localization	-5.561733	4.28975E-25
orf19.193	Ortholog of C. dubliniensis CD36 : Cd36_19310, C. parapsilosis CDC317 : CPAR2_209710, C. auris B8441 : B9J08_002949 and Candida tenuis NRRL Y-1498 : CANTEDRAFT_113479	-5.565979	0.000522348
PMM1	Phosphomannomutase; enzyme of O- and N-linked mannosylation; interconverts mannose-6-phosphate and mannose-l-phosphate; functional homolog of S. cerevisiae Sec53; antigenic in mice; Hap43-induced; flow model and Spider biofilm repressed	-5.567053	4.7575E-128
IMG2	Mitochondrial ribosomal protein of the large subunit; rat catheter biofilm induced	-5.604391	0.000262121
orf19.2756	Ortholog(s) have HDEL sequence binding activity, role in endoplasmic reticulum to Golgi vesicle-mediated transport and integral component of endoplasmic reticulum membrane localization	-5.710075	4.27064E-46

RPS27A	Ribosomal protein S27; rat catheter and Spider biofilm induced	-5.710522	2.68806E-07
orf19.3730	Protein similar to S. cerevisiae Kre27p, which has a role in resistance to killer toxin; predicted Kex2p substrate	-5.715129	1.43195E-10
UCF1	Upregulated by cAMP in filamentous growth; induced in high iron, decreased upon yeast-hypha switch; downregulation correlates with clinical fluconazole resistance; Ras1-regulated; Hap43-repressed; flow model biofilm induced	-5.721543	3.76205E-18
orf19.6358	Ortholog(s) have ubiquitin conjugating enzyme activity, ubiquitin-protein transferase activity and role in free ubiquitin chain polymerization, postreplication repair, protein K63-linked ubiquitination, protein polyubiquitination	-5.76052	1.70907E-42
orf19.4642	Protein of unknown function; Hap43-induced gene	-5.835533	5.19743E-55
ATO5	Putative fungal-specific transmembrane protein	-5.905516	1.22908E-07
orf19.7304	Protein of unknown function; Hap43-induced; transcript induced early in infection of reconstituted human epithelium, while expression of the C. dubliniensis ortholog is not upregulated; mutants are viable; rat catheter biofilm repressed	-5.984764	1.9228E-122
RPN9	Subunit of the 19S regulatory particle lid of the proteasome	-6.071687	2.4E-16
orf19.1606	Protein of unknown function; Plc1-regulated	-6.119069	9.83462E-12
orf19.1776	Putative pantetheine-phosphate adenylyltransferase (PPAT); which catalyzes 4th step in coenzyme A biosynthesis from pantothenate; rat catheter biofilm repressed	-6.162559	5.04152E-15
orf19.7330	Protein with a predicted heme oxygenase domain; Spider biofilm induced	-6.209555	4.45322E-23
orf19.5276	Putative nuclear pore-associated protein; Hap43p-induced gene; induced upon low-level peroxide stress; possibly an essential gene, disruptants not obtained by UAU1 method	-6.212898	4.33847E-16
orf19.119	Ortholog(s) have protein C-terminal S-isoprenylcysteine carboxyl O-methyltransferase activity	-6.68961	4.42976E-10
BLP1	Protein of unknown function, serum-induced	-6.785865	4.5254E-06
orf19.1043	Ortholog(s) have lysophosphatidic acid acyltransferase activity, role in cellular triglyceride homeostasis, lipid droplet organization and endoplasmic reticulum, lipid droplet localization	-6.848101	3.24389E-06
orf19.6788	Protein with a predicted role in cotranslational protein targeting to membrane; induced during chlamydospore formation in both C. albicans and C. dubliniensis	-6.891171	1.09875E-19
orf19.6602	Ortholog(s) have role in ubiquinone biosynthetic process, ubiquinone-6 biosynthetic process and mitochondrial inner membrane, mitochondrion localization	-6.909508	1.30505E-28
orf19.2230	Putative pre-mRNA-splicing factor; decreased transcription is observed upon benomyl treatment	-6.935853	2.82507E-06

orf19.6024	Ortholog of C. dubliniensis CD36 : Cd36_00770, C. auris B8441 : B9J08_004942, Candida tenuis NRRL Y-1498 : CANTEDRAFT_112621 and Debaryomyces hansenii CBS767 : DEHA2D09812g	-7.020836	2.19056E-11
orf19.7200	Ortholog of C. dubliniensis CD36 : Cd36_73480, Pichia stipitis Pignal : PICST_30727, Candida tropicalis NEW ASSEMBLY : CTRG1_05165 and Candida tropicalis MYA-3404 : CTRG_05165	-7.040684	2.09632E-11
orf19.6307	Ortholog of C. dubliniensis CD36 : Cd36_30250, C. auris B8441 : B9J08_001969, Candida tenuis NRRL Y-1498 : CANTEDRAFT_98619 and Debaryomyces hansenii CBS767 : DEHA2G22594g	-7.078003	2.32326E-11
orf19.1691	Plasma-membrane-localized protein; filament induced; Hog1, ketoconazole, fluconazole and hypoxia-induced; regulated by Nrg1, Tup1, Upc2; induced by prostaglandins; flow model biofilm induced; rat catheter and Spider biofilm repressed	-7.339988	6.03594E-32
orf19.1063	Ortholog(s) have alpha-1,6-mannosyltransferase activity, mannosyltransferase activity and role in GPI anchor biosynthetic process	-7.631492	4.17066E-13
orf19.5235	Putative mitochondrial ribosomal protein of the large subunit; Hap43-induced; mutants are viable; protein level decreases in stationary phase	-7.769187	6.12016E-08
orf19.2822	Essential component of the conserved oligomeric Golgi complex; role in fusion of transport vesicles to Golgi compartments; rat catheter biofilm repressed	-7.885278	5.64502E-08
LYS5	Phosphopantetheinyl transferase; enzyme of lysine biosynthesis; modifies, and thereby activates, Lys2p alpha-aminoadipate reductase; functional homolog of S. cerevisiae Lys5p	-8.088877	2.27384E-08
orf19.6687	Protein of unknown function; rat catheter biofilm repressed	-8.97537	0.005739989
orf19.1215	Ortholog of C. dubliniensis CD36 : Cd36_64550, Candida tropicalis NEW ASSEMBLY : CTRG1_02747, Candida tropicalis MYA-3404 : CTRG_02747 and Candida albicans WO-1 : CAWG_04972	-9.146357	0.017426324
ZCF25	Putative Zn(II)2Cys6 transcription factor	-9.321928	0.000779392
orf19.4189	Has domain(s) with predicted hydrolase activity	-9.336878	0.023490644
SPR3	Septin; mutant has no obvious phenotype	-9.819115	0.001558924
orf19.4834	Ortholog of C. dubliniensis CD36 : Cd36_09140, C. parapsilosis CDC317 : CPAR2_804920, Debaryomyces hansenii CBS767 : DEHA2B10516g and Pichia stipitis Pignal : PICST_30058	-10.0453	0.023630108
orf19.2781	Protein with predicted serine/threonine kinase and tyrosine kinase domains; possibly an essential gene, disruptants not obtained by UAU1 method; Spider biofilm induced	-10.09891	6.56932E-05

GBU1	Guanidinobutyrase (Gbase), enzyme involved in metabolism of guanidinobutyrate	-10.19229	0.001927249
orf19.3600	Protein of unknown function; flow model biofilm induced	-10.20864	0.015279553
orf19.3713	Protein of unknown function; flow model biofilm induced; Spider biofilm induced; induced by Mnl1 under weak acid stress; transcript detected in high-resolution tiling arrays	-10.34799	0.016341401
ARG4	Argininosuccinate lyase, catalyzes the final step in the arginine biosynthesis pathway; alkaline downregulated; flow model biofilm induced; Spider biofilm induced	-10.37721	0.000305943
orf19.1302	Ortholog of C. dubliniensis CD36 : Cd36_43500, Candida tropicalis NEW ASSEMBLY : CTRG1_00335, Spathaspora passalidarum NRRL Y-27907 : SPAPADRAFT_64619 and Candida tropicalis MYA-3404 : CTRG_00335	-10.44432	0.008487169
PTR2	Oligopeptide transporter involved in uptake of di-/tripeptides; highly induced during chlamydospore formation in both C. albicans and C. dubliniensis	-10.65881	1.87922E-05
orf19.1620	Protein of unknown function; induced by alpha pheromone in SpiderM mediumC4_02250C_AC. albicans SC5314BGL2C4_02250C_AORFVerifiedorf19.12034, orf6.3097, BGL21, IPF22613.1, IPF1046.1, Contig4-3104_0006, CA1541, CaO19.4565, CaO19.12034, orf19.4565, C4_02250C_B, C4_02250C, CAWG_03569Ca22chr4A_C_albicans_SC5314:445555 to 444629 | GBrowse	-10.71425	0.01229778
orf19.11	Protein of unknown function; transcript regulated by Mig1 and Tup1; rat catheter biofilm induced	-11.05438	0.00082601
orf19.1707	Ortholog of Candida albicans WO-1 : CAWG_02476	-11.18198	0.019416749
orf19.1026.1	Ortholog of C. dubliniensis CD36 : Cd36_03580, C. parapsilosis CDC317 : CPAR2_105090, C. auris B8441 : B9J08_004984 and Candida tenuis NRRL Y-1498 : CANTEDRAFT_106351	-11.21675	0.027223984
RGS2	Protein of RGS superfamily; mutants are viable; rat catheter and Spider biofilm induced	-11.44087	6.77374E-06
orf19.3436	Protein with a Rho GDP-dissociation inhibitor domain; macrophage-induced gene	-11.51833	0.000610397
orf19.4768	Protein of unknown function; Spider biofilm induced	-11.54271	1.4038E-06
orf19.6482	Protein of unknown function; induced by nitric oxide independent of Yhb1p; regulated by Sef1, Sfu1, and Hap43; rat catheter biofilm induced	-11.60424	0.000782239
TEF4	Putative translation elongation factor; genes encoding ribosomal subunits, translation factors, and tRNA synthetases are downregulated upon phagocytosis by murine macrophage	-11.75238	9.7443E-05
orf19.2778	Protein of unknown function; transcript is upregulated in clinical isolates from HIV+ patients with oral candidiasis	-11.82443	5.9439E-08

MNE1	Protein required for expression of NADH:ubiquinone oxidoreductase (mitochondrial complex I)	-11.82972	5.15164E-08
orf19.4861.1	Ortholog(s) have tRNA binding activity, role in tRNA export from nucleus and cytoplasm, nuclear pore localization	-12.02583	0.002988752
orf19.3783	Protein of unknown function; rat catheter biofilm induced	-12.08569	0.000241096
orf19.3607	Ortholog of C. dubliniensis CD36 : Cd36_22860, C. parapsilosis CDC317 : CPAR2_806540, C. auris B8441 : B9J08_004792 and Candida tenuis NRRL Y-1498 : CANTEDRAFT_108823	-12.11309	2.02796E-07
orf19.1360.1	Putative adhesin-like protein	-12.12175	0.015047313
orf19.1105	Ortholog of C. dubliniensis CD36 : Cd36_53630, C. parapsilosis CDC317 : CPAR2_302980, Pichia stipitis Pignal : PICST_32155, Candida tropicalis MYA-3404 : CTRG_03184 and Candida albicans WO-1 : CAWG_04763	-12.42347	5.88224E-05
orf19.685.1	Ortholog of C. parapsilosis CDC317 : CPAR2_602135, Candida tenuis NRRL Y-1498 : cten_CGOB_00042, Debaryomyces hansenii CBS767 : DEHA2F11858g and Pichia stipitis Pignal : PICST_60705	-12.47083	0.001653498
orf19.5724	Ortholog of C. dubliniensis CD36 : Cd36_64110, C. parapsilosis CDC317 : CPAR2_500910, Candida tenuis NRRL Y-1498 : CANTEDRAFT_115913 and Pichia stipitis Pignal : PICST_32870	-12.53625	2.67558E-06
DAL4	Putative allantoin permease; fungal-specific (no human or murine homolog)	-12.73415	3.85361E-09
NUE1	Mitochondrial protein required for expression of mitochondrial respiratory chain complex I (NADH:ubiquinone oxidoreductase)	-13.14157	3.10421E-07
orf19.36	Component of the conserved oligomeric Golgi complex; predicted to mediate fusion of transport vesicles to Golgi compartments; clade-associated gene expression	-13.24228	2.55483E-09
orf19.1795.1	Ortholog(s) have role in protein targeting to ER, signal peptide processing and signal peptidase complex localization	-13.35856	4.35039E-05
orf19.6185	Putative pseudouridine-5'-phosphate glycosidase; overlaps orf19.6186 and orf19.6187; Spider biofilm repressed	-13.39071	1.47648E-08
orf19.6795	Ortholog(s) have enzyme activator activity	-13.69986	1.54473E-06
orf19.2123	Predicted ORF from Assembly 19; removed from Assembly 20; restored based on comparative genome analysis	-13.74959	2.18996E-06
orf19.6308	Ortholog of Candida albicans WO-1 : CAWG_01818	-14.08901	1.38171E-09
PEX17	Putative peroxin	-14.12901	1.6897E-09
RVS162	Protein containing a BAR domain, which is found in proteins involved in membrane curvature; null mutant does not display the endocytic, hyphal growth, virulence, or cell wall defects exhibited by mutants in related genes RVS161 and RVS167	-14.22807	9.53994E-11
ARC18	Putative ARP2/3 complex subunit; mutation confers hypersensitivity to cytochalasin D	-15.38981	2.73851E-11

LEU2	Isopropyl malate dehydrogenase; leucine biosynthesis; induced by human whole blood or PMNs; protein level decreases in stationary phase; GlcNAc-induced protein; flow model biofilm repressed	-16.94211	1.12692E-17
