# Supplementary material for: DNA Damage Checkpoints Govern Global Gene Transcription and Exhibit Species-Specific Regulation on HOF1 in Candida albicans
Source: J Fungi (Basel). 2024 May 29;10(6):387. doi: 10.3390/jof10060387 (PMC11204775; doi:10.3390/jof10060387)
Supplement: Supplementary file 1 [file jof-10-00387-s001.zip › Table S5.pdf]

No MMS	With MMS
gene-CAALFM_C401340WA	gene-CAALFM_C702270WA
gene-CAALFM_C702260WA	gene-CAALFM_C702260WA
gene-CAALFM_C702270WA	gene-CAALFM_CR00840CA
gene-CAALFM_CR07740WA	gene-CAALFM_C702280WA
gene-CAALFM_C505180WA	gene-CAALFM_C406920CA
gene-CAALFM_C406920CA	gene-CAALFM_C208880WA
gene-CAALFM_C702280WA	gene-CAALFM_C500510WA
gene-CAALFM_C404080CA	gene-CAALFM_C306630WA
gene-CAALFM_CR02280WA	gene-CAALFM_C404080CA
gene-CAALFM_C600470CA	gene-CAALFM_C401340WA
gene-CAALFM_C504250WA	gene-CAALFM_C106370CA
gene-CAALFM_C504480CA	gene-CAALFM_C602100WA
gene-CAALFM_C101510WA	gene-CAALFM_CR04440CA
gene-CAALFM_C306630WA	gene-CAALFM_C200310WA
gene-CAALFM_CR04440CA	gene-CAALFM_CR09850CA
gene-CAALFM_C501380WA	gene-CAALFM_C602760WA
gene-CAALFM_C602100WA	gene-CAALFM_CR02280WA
gene-CAALFM_C702360WA	gene-CAALFM_C504230WA
gene-CAALFM_C303460CA	gene-CAALFM_C403500CA
gene-CAALFM_C210730WA	gene-CAALFM_C700430WA
gene-CAALFM_C304660CA	gene-CAALFM_C202910WA
gene-CAALFM_C303450CA	gene-CAALFM_C504110WA
gene-CAALFM_CR07060CA	gene-CAALFM_CR10540CA
gene-CAALFM_C603440WA	gene-CAALFM_C504250WA
gene-CAALFM_C700430WA	gene-CAALFM_C204200WA
gene-CAALFM_CR08080WA	gene-CAALFM_C402900CA
gene-CAALFM_C603090WA	gene-CAALFM_C501380WA
gene-CAALFM_CR05360CA	gene-CAALFM_CR07740WA
gene-CAALFM_C505220WA	gene-CAALFM_C603790CA
gene-CAALFM_C306660CA	gene-CAALFM_C303650WA
gene-CAALFM_C503600WA	MSTRG.7648
gene-CAALFM_C102630CA	gene-CAALFM_C301560WA
gene-CAALFM_C502430WA	gene-CAALFM_C603440WA
gene-CAALFM_C601810WA	gene-CAALFM_C407000WA
gene-CAALFM_C307650CA	gene-CAALFM_C107980CA
gene-CAALFM_C401000CA	gene-CAALFM_C303690WA
gene-CAALFM_C505460CA	gene-CAALFM_C106690WA
gene-CAALFM_C208380CA	gene-CAALFM_C402440CA
gene-CAALFM_C500810CA	gene-CAALFM_C702000CA
gene-CAALFM_C303650WA	gene-CAALFM_C110400CA

gene-CAALFM_C602840CA	gene-CAALFM_C603090WA
gene-CAALFM_C502630CA	gene-CAALFM_CR05360CA
gene-CAALFM_C504120CA	gene-CAALFM_C113820CA
gene-CAALFM_CR06550CA	gene-CAALFM_C302790WA
gene-CAALFM_C500200CA	gene-CAALFM_C701200CA
gene-CAALFM_C401220CA	gene-CAALFM_C205540CA
gene-CAALFM_C501170WA	gene-CAALFM_C104260WA
gene-CAALFM_C110950CA	gene-CAALFM_C108000WA
gene-CAALFM_C105500WA	gene-CAALFM_C501360WA
gene-CAALFM_C700330CA	gene-CAALFM_CR10550WA
gene-CAALFM_C205090WA	gene-CAALFM_C101510WA
gene-CAALFM_C204200WA	gene-CAALFM_C210730WA
gene-CAALFM_C505080WA	gene-CAALFM_C111430WA
gene-CAALFM_C403500CA	gene-CAALFM_C406820CA
gene-CAALFM_C403470CA	gene-CAALFM_C202530WA
gene-CAALFM_C400810CA	gene-CAALFM_C406990WA
gene-CAALFM_C302790WA	gene-CAALFM_CR10100CA
gene-CAALFM_C504840CA	gene-CAALFM_C505080WA
gene-CAALFM_C303690WA	gene-CAALFM_C203000CA
gene-CAALFM_CR10470CA	gene-CAALFM_C500210CA
gene-CAALFM_C504370CA	gene-CAALFM_C404050CA
gene-CAALFM_C202430WA	gene-CAALFM_C110950CA
gene-CAALFM_C207670CA	gene-CAALFM_C306370CA
gene-CAALFM_C300280CA	gene-CAALFM_C206160WA
gene-CAALFM_C402800WA	gene-CAALFM_C500200CA
gene-CAALFM_C603790CA	gene-CAALFM_C405760WA
gene-CAALFM_C500300CA	gene-CAALFM_C303450CA
gene-CAALFM_C503050CA	gene-CAALFM_CR10110WA
gene-CAALFM_C406210CA	gene-CAALFM_CR02980CA
gene-CAALFM_C501140CA	gene-CAALFM_C502460CA
gene-CAALFM_C306210CA	gene-CAALFM_CR08510WA
gene-CAALFM_CR04110WA	gene-CAALFM_C505220WA
gene-CAALFM_C500210CA	gene-CAALFM_C104170CA
gene-CAALFM_C113640WA	gene-CAALFM_CR10650WA
gene-CAALFM_C209350WA	gene-CAALFM_C502370CA
gene-CAALFM_CR10100CA	gene-CAALFM_C104390CA
gene-CAALFM_C113930WA	gene-CAALFM_CR05570CA
gene-CAALFM_C114150CA	gene-CAALFM_C104240CA
gene-CAALFM_C405760WA	gene-CAALFM_C306030WA
gene-CAALFM_C108630WA	gene-CAALFM_C503630CA
gene-CAALFM_C403440CA	gene-CAALFM_C208380CA
gene-CAALFM_C502370CA	gene-CAALFM_C702750WA
gene-CAALFM_C700110WA	gene-CAALFM_C501110WA

gene-CAALFM_C106820WA	gene-CAALFM_C106820WA
gene-CAALFM_C305160CA	gene-CAALFM_C704190CA
gene-CAALFM_CR05660WA	gene-CAALFM_C604160CA
gene-CAALFM_C304900WA	gene-CAALFM_C100160CA
gene-CAALFM_C402830CA	gene-CAALFM_C505460CA
gene-CAALFM_C107090CA	gene-CAALFM_C306660CA
gene-CAALFM_C505330CA	gene-CAALFM_C401150WA
gene-CAALFM_C304350CA	gene-CAALFM_C110970WA
gene-CAALFM_C107380CA	gene-CAALFM_C503210CA
gene-CAALFM_C700180WA	gene-CAALFM_CR05520WA
gene-CAALFM_C204140WA	gene-CAALFM_C302040CA
gene-CAALFM_C106670WA	gene-CAALFM_CR04110WA
gene-CAALFM_C404520WA	gene-CAALFM_C109080CA
gene-CAALFM_C204210WA	gene-CAALFM_C109870WA
gene-CAALFM_C304840CA	gene-CAALFM_C207170CA
gene-CAALFM_C111270WA	gene-CAALFM_C503600WA
gene-CAALFM_CR01370CA	gene-CAALFM_C602840CA
gene-CAALFM_C303780WA	gene-CAALFM_C303570CA
gene-CAALFM_C107130CA	gene-CAALFM_C600480CA
gene-CAALFM_C104600CA	gene-CAALFM_C305160CA
gene-CAALFM_C601740CA	gene-CAALFM_C603990CA
gene-CAALFM_C703890CA	gene-CAALFM_C505370CA
gene-CAALFM_C502460CA	gene-CAALFM_CR06810WA
gene-CAALFM_C203000CA	gene-CAALFM_C407080CA
gene-CAALFM_C504650CA	gene-CAALFM_C504120CA
gene-CAALFM_C405200CA	gene-CAALFM_CR08080WA
gene-CAALFM_C100640CA	gene-CAALFM_C400810CA
gene-CAALFM_CR04340WA	gene-CAALFM_C405200CA
gene-CAALFM_C704190CA	gene-CAALFM_C300370CA
gene-CAALFM_C500510WA	gene-CAALFM_C304560WA
gene-CAALFM_C108810CA	gene-CAALFM_C304840CA
gene-CAALFM_CR04130CA	gene-CAALFM_CR03360WA
gene-CAALFM_C307800CA	gene-CAALFM_C202430WA
gene-CAALFM_C100200CA	gene-CAALFM_C700570WA
gene-CAALFM_C110970WA	gene-CAALFM_C201630WA
gene-CAALFM_C404240CA	gene-CAALFM_C202390WA
gene-CAALFM_C205580WA	gene-CAALFM_C603640WA
gene-CAALFM_C501640WA	gene-CAALFM_CR02120CA
gene-CAALFM_C503030WA	gene-CAALFM_C305780CA
gene-CAALFM_C603450CA	gene-CAALFM_C505330CA
gene-CAALFM_C205540CA	gene-CAALFM_C501220WA
gene-CAALFM_C503170CA	gene-CAALFM_C207350WA

gene-CAALFM_C502840CA	gene-CAALFM_C106760CA
gene-CAALFM_C110250CA	gene-CAALFM_C104600CA
gene-CAALFM_C300370CA	gene-CAALFM_C402010CA
gene-CAALFM_C209320CA	gene-CAALFM_C207670CA
gene-CAALFM_C106760CA	gene-CAALFM_CR04170WA
gene-CAALFM_C304540CA	gene-CAALFM_C109710CA
gene-CAALFM_C209100CA	gene-CAALFM_C113930WA
gene-CAALFM_CR03650WA	gene-CAALFM_C504480CA
gene-CAALFM_C111430WA	gene-CAALFM_C700180WA
gene-CAALFM_C305950WA	gene-CAALFM_C100640CA
gene-CAALFM_CR01500WA	gene-CAALFM_C401010CA
gene-CAALFM_CR02050CA	gene-CAALFM_C600470CA
gene-CAALFM_C500350CA	gene-CAALFM_C303280CA
gene-CAALFM_C108000WA	gene-CAALFM_CR01410CA
gene-CAALFM_C402000CA	gene-CAALFM_C501010WA
gene-CAALFM_C209890WA	gene-CAALFM_C302760CA
gene-CAALFM_C406980WA	gene-CAALFM_CR03420CA
gene-CAALFM_C301560WA	gene-CAALFM_C108290CA
gene-CAALFM_C302070CA	gene-CAALFM_C307750WA
gene-CAALFM_C505370CA	gene-CAALFM_C111990WA
gene-CAALFM_C401960CA	gene-CAALFM_C702360WA
gene-CAALFM_C701380WA	gene-CAALFM_C108440CA
gene-CAALFM_C603640WA	gene-CAALFM_C307800CA
gene-CAALFM_C105840WA	gene-CAALFM_C700470CA
gene-CAALFM_CR02980CA	gene-CAALFM_C106250WA
gene-CAALFM_C701170CA	gene-CAALFM_CR09990WA
gene-CAALFM_C500160WA	gene-CAALFM_C207960CA
gene-CAALFM_CR03420CA	gene-CAALFM_C502840CA
gene-CAALFM_C105050CA	gene-CAALFM_CR04130CA
gene-CAALFM_C206160WA	gene-CAALFM_C500630CA
gene-CAALFM_C306720WA	gene-CAALFM_C402800WA
gene-CAALFM_C702830CA	gene-CAALFM_CR07390CA
gene-CAALFM_C300890CA	gene-CAALFM_C102630CA
gene-CAALFM_C603810WA	gene-CAALFM_C306610WA
gene-CAALFM_C402260CA	gene-CAALFM_C306160CA
gene-CAALFM_C106590CA	gene-CAALFM_C306380WA
gene-CAALFM_C502440CA	gene-CAALFM_C505340WA
gene-CAALFM_C300530CA	gene-CAALFM_C503050CA
gene-CAALFM_C404260CA	gene-CAALFM_C505350WA
gene-CAALFM_C305910WA	gene-CAALFM_C307650CA
gene-CAALFM_C114240WA	gene-CAALFM_C505180WA
gene-CAALFM_C113060CA	gene-CAALFM_C601550CA

gene-CAALFM_C103280WA
gene-CAALFM_C503650CA
gene-CAALFM_C109370WA
gene-CAALFM_C306370CA
gene-CAALFM_C302000WA
gene-CAALFM_C301520CA
gene-CAALFM_C500630CA
gene-CAALFM_C109210CA
gene-CAALFM_C501360WA
gene-CAALFM_C306610WA
gene-CAALFM_CR03790CA
gene-CAALFM_C112620WA
gene-CAALFM_C109320CA
gene-CAALFM_C700520WA
gene-CAALFM_C111070WA
gene-CAALFM_C704010WA
gene-CAALFM_CR03360WA
gene-CAALFM_C207170CA
gene-CAALFM_C305780CA
gene-CAALFM_C602310WA
gene-CAALFM_C304560WA
gene-CAALFM_C502480WA
gene-CAALFM_C500860WA
gene-CAALFM_C306030WA
gene-CAALFM_C108870CA
gene-CAALFM_CR10260WA
gene-CAALFM_C205330CA
gene-CAALFM_C406990WA
gene-CAALFM_CR05650WA
gene-CAALFM_C406190CA
gene-CAALFM_C100560WA
gene-CAALFM_C504670WA
gene-CAALFM_C501430CA
gene-CAALFM_CR10550WA
gene-CAALFM_C307740WA
gene-CAALFM_C101130WA
gene-CAALFM_CR02120CA
gene-CAALFM_C300560CA
gene-CAALFM_C400860CA
gene-CAALFM_C501340WA

gene-CAALFM_CR06110CA
gene-CAALFM_C402260CA
gene-CAALFM_C303460CA
gene-CAALFM_C300330WA
gene-CAALFM_C503410CA
gene-CAALFM_C602910WA
gene-CAALFM_C107030CA
gene-CAALFM_C108810CA
gene-CAALFM_C600110CA
gene-CAALFM_C400430WA
gene-CAALFM_C105360CA
gene-CAALFM_C103230CA
gene-CAALFM_C200070CA
gene-CAALFM_C114240WA
gene-CAALFM_C205090WA
gene-CAALFM_CR05730CA
gene-CAALFM_C204930CA
gene-CAALFM_C209100CA
gene-CAALFM_C505120WA
gene-CAALFM_CR03550WA
gene-CAALFM_CR09800CA
gene-CAALFM_C302880WA
gene-CAALFM_C702950CA
gene-CAALFM_C502780WA
gene-CAALFM_C405680WA
gene-CAALFM_C112400CA
gene-CAALFM_C700970CA
gene-CAALFM_C503030WA
gene-CAALFM_C200830CA
gene-CAALFM_C101070CA
gene-CAALFM_C106590CA
gene-CAALFM_C700190WA
gene-CAALFM_C702770WA
gene-CAALFM_C209320CA
gene-CAALFM_C600370CA
gene-CAALFM_C209020WA
gene-CAALFM_C307460WA
gene-CAALFM_C100560WA
gene-CAALFM_C504650CA
gene-CAALFM_C406210CA

gene-CAALFM_CR00400CA
gene-CAALFM_C404000WA
gene-CAALFM_C106530CA
gene-CAALFM_C104570CA
gene-CAALFM_C201890WA
gene-CAALFM_C502620CA
gene-CAALFM_C302760CA
gene-CAALFM_CR04170WA
gene-CAALFM_C701100CA
gene-CAALFM_CR06110CA
gene-CAALFM_C701430CA
gene-CAALFM_C105900WA
gene-CAALFM_C501110WA
gene-CAALFM_C602290CA
gene-CAALFM_CR02030CA
gene-CAALFM_C401650CA
gene-CAALFM_CR01700CA
gene-CAALFM_CR04450CA
gene-CAALFM_C307750WA
gene-CAALFM_C201390WA
gene-CAALFM_C300360WA
gene-CAALFM_C104330WA
gene-CAALFM_C110380CA
gene-CAALFM_CR05520WA
gene-CAALFM_CR07750CA
gene-CAALFM_C111350CA
gene-CAALFM_CR01820WA
gene-CAALFM_CR04140WA
gene-CAALFM_C703270WA
gene-CAALFM_CR04460CA
gene-CAALFM_C203500WA
gene-CAALFM_C504280CA
gene-CAALFM_C505120WA
gene-CAALFM_C504740CA
gene-CAALFM_C600790CA
gene-CAALFM_CR06810WA
gene-CAALFM_CR05220CA
gene-CAALFM_CR03350CA
gene-CAALFM_C505250CA

gene-CAALFM_C300860WA
gene-CAALFM_C105690CA
gene-CAALFM_CR05660WA
gene-CAALFM_C206830CA
gene-CAALFM_CR08500WA
gene-CAALFM_C703340CA
gene-CAALFM_CR00810WA
gene-CAALFM_C306820CA
gene-CAALFM_C402830CA
gene-CAALFM_C204880CA
gene-CAALFM_C307740WA
gene-CAALFM_C404000WA
gene-CAALFM_CR03380WA
gene-CAALFM_C205330CA
gene-CAALFM_CR03650WA
gene-CAALFM_C113170CA
gene-CAALFM_C114150CA
gene-CAALFM_C100960CA
gene-CAALFM_CR02030CA
gene-CAALFM_CR10470CA
gene-CAALFM_C304690CA
gene-CAALFM_C304110CA
gene-CAALFM_C303780WA
gene-CAALFM_C600160WA
gene-CAALFM_C204140WA
gene-CAALFM_C111040WA
gene-CAALFM_CR06800CA
gene-CAALFM_CR04240CA
gene-CAALFM_C505400WA
gene-CAALFM_C401080WA
gene-CAALFM_C404240CA
gene-CAALFM_C500070WA
gene-CAALFM_C701810WA
gene-CAALFM_C701100CA
gene-CAALFM_C601440CA
gene-CAALFM_C500310CA
gene-CAALFM_CR10240WA
gene-CAALFM_C301370CA
gene-CAALFM_C200840WA

gene-CAALFM_C700470CA	gene-CAALFM_CR08290WA
gene-CAALFM_CR01600CA	gene-CAALFM_C500300CA
gene-CAALFM_C301800CA	gene-CAALFM_C101130WA
gene-CAALFM_CR06680CA	gene-CAALFM_C108630WA
gene-CAALFM_C501390CA	gene-CAALFM_CR04450CA
gene-CAALFM_C307460WA	gene-CAALFM_C306310CA
gene-CAALFM_C500070WA	gene-CAALFM_C104570CA
gene-CAALFM_C404560CA	gene-CAALFM_C303850CA
gene-CAALFM_C502060WA	gene-CAALFM_C703890CA
gene-CAALFM_CR02100CA	gene-CAALFM_C104180WA
gene-CAALFM_C701810WA	gene-CAALFM_C200570WA
gene-CAALFM_C206470WA	gene-CAALFM_C300530CA
gene-CAALFM_C209370CA	gene-CAALFM_C500610CA
gene-CAALFM_C702060WA	gene-CAALFM_C300560CA
gene-CAALFM_C203800CA	gene-CAALFM_C700520WA
gene-CAALFM_C505290CA	gene-CAALFM_C105050CA
gene-CAALFM_C203200WA	gene-CAALFM_C300300WA
gene-CAALFM_C108670WA	gene-CAALFM_C201420CA
gene-CAALFM_C602210WA	gene-CAALFM_CR06550CA
gene-CAALFM_C103290WA	gene-CAALFM_CR10260WA
gene-CAALFM_C106090CA	gene-CAALFM_C107810CA
gene-CAALFM_C209230CA	gene-CAALFM_C105840WA
gene-CAALFM_C501130WA	gene-CAALFM_C700200WA
gene-CAALFM_C207960CA	gene-CAALFM_C301800CA
gene-CAALFM_CR02210WA	gene-CAALFM_C701620CA
gene-CAALFM_C209310CA	gene-CAALFM_CR10230WA
gene-CAALFM_C402060CA	gene-CAALFM_C700330CA
gene-CAALFM_C202450CA	gene-CAALFM_CR03830CA
gene-CAALFM_C304490WA	gene-CAALFM_C110250CA
gene-CAALFM_C107260CA	gene-CAALFM_CR09370WA
gene-CAALFM_CR08940WA	gene-CAALFM_CR05990CA
gene-CAALFM_C107990CA	gene-CAALFM_C113060CA
gene-CAALFM_C502300CA	gene-CAALFM_CR10060WA
gene-CAALFM_C209380WA	gene-CAALFM_CR01950WA
gene-CAALFM_C300420WA	gene-CAALFM_C600790CA
gene-CAALFM_C111040WA	gene-CAALFM_C110260CA
gene-CAALFM_C504080CA	gene-CAALFM_C301980CA
gene-CAALFM_C504640CA	gene-CAALFM_C305140CA
gene-CAALFM_C306310CA	gene-CAALFM_C205530CA
gene-CAALFM_C112410CA	gene-CAALFM_C200140WA
gene-CAALFM_CR07460CA	gene-CAALFM_C209380WA
gene-CAALFM_C502900WA	gene-CAALFM_C100570CA

gene-CAALFM_C104390CA	gene-CAALFM_C502010CA
gene-CAALFM_C300450CA	gene-CAALFM_C407010CA
gene-CAALFM_C702950CA	gene-CAALFM_C407100CA
gene-CAALFM_C701580WA	gene-CAALFM_C109370WA
gene-CAALFM_C104990CA	gene-CAALFM_C301020WA
gene-CAALFM_C201200CA	gene-CAALFM_C303910WA
gene-CAALFM_C303260WA	gene-CAALFM_C405780CA
gene-CAALFM_C601040CA	gene-CAALFM_C504570CA
gene-CAALFM_C401080WA	gene-CAALFM_CR06300CA
gene-CAALFM_CR09980WA	gene-CAALFM_CR01370CA
gene-CAALFM_CR00630WA	gene-CAALFM_C307470WA
gene-CAALFM_C500380WA	gene-CAALFM_C500570WA
gene-CAALFM_C701030CA	gene-CAALFM_C204130WA
gene-CAALFM_C600480CA	gene-CAALFM_C103290WA
gene-CAALFM_C702110WA	gene-CAALFM_C304540CA
gene-CAALFM_C601350WA	gene-CAALFM_CR02210WA
gene-CAALFM_C104620WA	gene-CAALFM_C109170WA
gene-CAALFM_C207130CA	gene-CAALFM_C502770WA
gene-CAALFM_C109710CA	gene-CAALFM_C703270WA
gene-CAALFM_C601550CA	gene-CAALFM_C403470CA
gene-CAALFM_C600510CA	gene-CAALFM_C700510WA
gene-CAALFM_CR02330CA	gene-CAALFM_C209420WA
gene-CAALFM_C600550WA	gene-CAALFM_C305950WA
gene-CAALFM_CR01240CA	gene-CAALFM_C207040WA
gene-CAALFM_CR10230WA	gene-CAALFM_C304320WA
gene-CAALFM_C501820WA	gene-CAALFM_CR02190CA
gene-CAALFM_C502770WA	gene-CAALFM_C100880WA
gene-CAALFM_CR10650WA	gene-CAALFM_C700110WA
gene-CAALFM_CR00810WA	gene-CAALFM_C110030WA
gene-CAALFM_C206200CA	gene-CAALFM_C101860WA
gene-CAALFM_C501180WA	gene-CAALFM_C602900CA
gene-CAALFM_C205720CA	gene-CAALFM_C107130CA
gene-CAALFM_C205260WA	gene-CAALFM_C205580WA
gene-CAALFM_C207540WA	gene-CAALFM_C110440WA
gene-CAALFM_CR08510WA	gene-CAALFM_C500940CA
gene-CAALFM_C113550CA	gene-CAALFM_C103770WA
gene-CAALFM_C204130WA	gene-CAALFM_C102080WA
gene-CAALFM_C406820CA	gene-CAALFM_C103280WA

gene-CAALFM_C505350WA	gene-CAALFM_C111350CA
gene-CAALFM_C302030WA	gene-CAALFM_C601890CA
gene-CAALFM_C108290CA	gene-CAALFM_C111270WA
gene-CAALFM_C202520WA	gene-CAALFM_C204210WA
gene-CAALFM_C107810CA	gene-CAALFM_C501340WA
gene-CAALFM_C200720CA	gene-CAALFM_C209350WA
gene-CAALFM_C105610WA	gene-CAALFM_C101390CA
gene-CAALFM_C503690WA	gene-CAALFM_C501170WA
gene-CAALFM_C105140WA	gene-CAALFM_C100950CA
gene-CAALFM_C111280WA	gene-CAALFM_C402060CA
gene-CAALFM_C502010CA	gene-CAALFM_CR08940WA
gene-CAALFM_C200830CA	gene-CAALFM_CR05630WA
gene-CAALFM_C300490WA	gene-CAALFM_C112550CA
gene-CAALFM_C504830WA	gene-CAALFM_C108120WA
gene-CAALFM_C202760WA	gene-CAALFM_C202750CA
gene-CAALFM_C200820WA	gene-CAALFM_CR05650WA
gene-CAALFM_C702940CA	gene-CAALFM_CR02100CA
gene-CAALFM_C204930CA	gene-CAALFM_C401960CA
gene-CAALFM_CR04010CA	gene-CAALFM_CR08100CA
gene-CAALFM_CR01410CA	gene-CAALFM_C304900WA
gene-CAALFM_C100570CA	gene-CAALFM_C306070CA
gene-CAALFM_C107800WA	gene-CAALFM_CR07460CA
gene-CAALFM_C402010CA	gene-CAALFM_C504750CA
gene-CAALFM_C110580CA	gene-CAALFM_C107090CA
gene-CAALFM_C305140CA	gene-CAALFM_C703540CA
gene-CAALFM_C505240CA	gene-CAALFM_C504080CA
gene-CAALFM_C502650CA	gene-CAALFM_C505480WA
gene-CAALFM_C701200CA	gene-CAALFM_C500350CA
gene-CAALFM_C110010CA	gene-CAALFM_C505470WA
gene-CAALFM_C701020CA	gene-CAALFM_C600670WA
gene-CAALFM_CR08180CA	gene-CAALFM_C113640WA
gene-CAALFM_C602910WA	gene-CAALFM_C109510WA
gene-CAALFM_C703220CA	gene-CAALFM_C106910CA
gene-CAALFM_C500090CA	gene-CAALFM_CR02110WA
gene-CAALFM_C304760CA	gene-CAALFM_C701380WA
gene-CAALFM_C113820CA	gene-CAALFM_C404720WA
gene-CAALFM_CR02190CA	gene-CAALFM_CR10690WA
gene-CAALFM_C503900WA	gene-CAALFM_C107800WA
gene-CAALFM_C205200CA	gene-CAALFM_C300420WA
gene-CAALFM_C112010CA	gene-CAALFM_C202190CA
gene-CAALFM_C304400CA	gene-CAALFM_C208870CA
gene-CAALFM_C300840CA	gene-CAALFM_C702830CA
gene-CAALFM_C300330WA	gene-CAALFM_C300350WA
gene-CAALFM_C205790CA	gene-CAALFM_C205790CA

gene-CAALFM_C200100CA	gene-CAALFM_C401650CA
gene-CAALFM_C109010WA	gene-CAALFM_C300490WA
gene-CAALFM_C505470WA	gene-CAALFM_C306060WA
gene-CAALFM_C104490WA	gene-CAALFM_C109280WA
gene-CAALFM_C112440WA	gene-CAALFM_C108670WA
gene-CAALFM_CR03550WA	gene-CAALFM_C207300CA
gene-CAALFM_CR10060WA	gene-CAALFM_C405660CA
gene-CAALFM_C105210CA	gene-CAALFM_C306300WA
gene-CAALFM_C204080WA	gene-CAALFM_C502340CA
gene-CAALFM_CR03400WA	gene-CAALFM_CR05220CA
gene-CAALFM_C302040CA	gene-CAALFM_C303250WA
gene-CAALFM_C307290WA	gene-CAALFM_C207540WA
gene-CAALFM_C503580CA	gene-CAALFM_C207130CA
gene-CAALFM_C500030WA	gene-CAALFM_C500810CA
gene-CAALFM_C703940CA	gene-CAALFM_C504640CA
gene-CAALFM_CR10240WA	gene-CAALFM_C701490WA
gene-CAALFM_C206360CA	gene-CAALFM_C100990CA
gene-CAALFM_C503630CA	gene-CAALFM_CR06380CA
gene-CAALFM_C702750WA	gene-CAALFM_C501140CA
gene-CAALFM_CR06800CA	gene-CAALFM_CR02050CA
gene-CAALFM_C504600CA	gene-CAALFM_C110880WA
gene-CAALFM_C505380WA	gene-CAALFM_C202500WA
gene-CAALFM_C105640CA	gene-CAALFM_C105210CA
gene-CAALFM_C306690CA	gene-CAALFM_C501180WA
gene-CAALFM_C200200WA	gene-CAALFM_C300100WA
gene-CAALFM_C502180CA	gene-CAALFM_C100200CA
gene-CAALFM_C307630CA	gene-CAALFM_C405570CA
gene-CAALFM_C106600WA	gene-CAALFM_C207100WA
gene-CAALFM_C104810WA	gene-CAALFM_C503510CA
gene-CAALFM_C107110WA	gene-CAALFM_CR06720WA
gene-CAALFM_CR00440CA	gene-CAALFM_C502480WA
gene-CAALFM_C203950WA	gene-CAALFM_C504370CA
gene-CAALFM_C304230WA	gene-CAALFM_C306720WA
gene-CAALFM_C307550CA	gene-CAALFM_CR04460CA
gene-CAALFM_C303770CA	gene-CAALFM_C304660CA
gene-CAALFM_CR04240CA	gene-CAALFM_C201060CA
gene-CAALFM_C304110CA	gene-CAALFM_CR06040WA
gene-CAALFM_C701210CA	gene-CAALFM_C502470WA
gene-CAALFM_C109170WA	gene-CAALFM_C109810WA
gene-CAALFM_C504780WA	gene-CAALFM_C504280CA

gene-CAALFM_C303280CA	gene-CAALFM_C203500WA
gene-CAALFM_C103230CA	gene-CAALFM_C503310CA
gene-CAALFM_C503310CA	gene-CAALFM_C205200CA
gene-CAALFM_C101320WA	gene-CAALFM_C504670WA
gene-CAALFM_C404640CA	gene-CAALFM_C105640CA
gene-CAALFM_C107030CA	gene-CAALFM_CR09980WA
gene-CAALFM_CR09860WA	gene-CAALFM_CR07290WA
gene-CAALFM_C104240CA	gene-CAALFM_C400920CA
gene-CAALFM_C306060WA	gene-CAALFM_C703180CA
gene-CAALFM_CR00430CA	gene-CAALFM_C303900CA
gene-CAALFM_C500570WA	gene-CAALFM_C305960WA
gene-CAALFM_C406040WA	gene-CAALFM_C405110CA
gene-CAALFM_CR00140WA	gene-CAALFM_C104620WA
gene-CAALFM_C110470WA	gene-CAALFM_C300890CA
gene-CAALFM_C200840WA	gene-CAALFM_C601810WA
gene-CAALFM_C300300WA	gene-CAALFM_C300270CA
gene-CAALFM_C503670CA	gene-CAALFM_C505250CA
gene-CAALFM_C400430WA	gene-CAALFM_C403270WA
gene-CAALFM_C505340WA	gene-CAALFM_C202570WA
gene-CAALFM_C604160CA	gene-CAALFM_C207370WA
gene-CAALFM_C702790CA	gene-CAALFM_C200620CA
gene-CAALFM_CR05250CA	gene-CAALFM_C202450CA
gene-CAALFM_C101390CA	gene-CAALFM_CR00140WA
gene-CAALFM_CR10540CA	gene-CAALFM_C703940CA
gene-CAALFM_C500310CA	gene-CAALFM_C404660CA
gene-CAALFM_C108440CA	gene-CAALFM_C302840WA
gene-CAALFM_C109870WA	gene-CAALFM_C400480WA
gene-CAALFM_C406600WA	gene-CAALFM_C210610WA
gene-CAALFM_C601890CA	gene-CAALFM_C407150WA
gene-CAALFM_C204360WA	gene-CAALFM_C504840CA
gene-CAALFM_C505500CA	gene-CAALFM_C601350WA
gene-CAALFM_CR04050CA	gene-CAALFM_C200720CA
gene-CAALFM_C111770CA	gene-CAALFM_C701840WA
gene-CAALFM_C703540CA	gene-CAALFM_C304400CA
gene-CAALFM_C113830CA	gene-CAALFM_C100030CA
gene-CAALFM_C407150WA	gene-CAALFM_C103250CA
gene-CAALFM_C501840CA	gene-CAALFM_C109790CA
gene-CAALFM_C303850CA	gene-CAALFM_C502550CA
gene-CAALFM_C100030CA	gene-CAALFM_C302070CA
gene-CAALFM_CR07600WA	gene-CAALFM_C401260WA
gene-CAALFM_C306820CA	gene-CAALFM_C604350CA

gene-CAALFM_C302020WA
gene-CAALFM_C102850WA
gene-CAALFM_C205530CA
gene-CAALFM_C300350WA
gene-CAALFM_CR10690WA
gene-CAALFM_CR05630WA
gene-CAALFM_C703480WA
gene-CAALFM_CR10440WA
gene-CAALFM_C100980WA
gene-CAALFM_C500950CA
gene-CAALFM_C103740WA
gene-CAALFM_C301980CA
gene-CAALFM_C701620CA
gene-CAALFM_CR04300WA
gene-CAALFM_C505510CA
gene-CAALFM_CR03660CA
gene-CAALFM_C501530CA
gene-CAALFM_C503510CA
gene-CAALFM_C405110CA
gene-CAALFM_C602520WA
gene-CAALFM_C405650WA
gene-CAALFM_C110440WA
gene-CAALFM_C500790CA
gene-CAALFM_CR00280CA
gene-CAALFM_C600370CA
gene-CAALFM_C503150WA
gene-CAALFM_C200610CA
gene-CAALFM_C502310CA
gene-CAALFM_C109540WA
gene-CAALFM_CR01950WA
gene-CAALFM_C206290CA
gene-CAALFM_C501250WA
gene-CAALFM_C202500WA
gene-CAALFM_C203980CA
gene-CAALFM_C108700WA
gene-CAALFM_C101560WA
gene-CAALFM_C402160CA
gene-CAALFM_C600390WA
gene-CAALFM_C402270CA

gene-CAALFM_C602780CA
gene-CAALFM_C307630CA
gene-CAALFM_CR07750CA
gene-CAALFM_C210320CA
gene-CAALFM_C108400CA
gene-CAALFM_C112620WA
gene-CAALFM_C107140CA
gene-CAALFM_C305910WA
gene-CAALFM_CR01600CA
gene-CAALFM_C203940CA
gene-CAALFM_CR04940WA
gene-CAALFM_C113730CA
gene-CAALFM_C106700WA
gene-CAALFM_C404520WA
gene-CAALFM_C109320CA
gene-CAALFM_C206290CA
gene-CAALFM_C111360WA
gene-CAALFM_CR00070WA
gene-CAALFM_C405650WA
gene-CAALFM_C101110CA
gene-CAALFM_C406040WA
gene-CAALFM_C600510CA
gene-CAALFM_C502170CA
gene-CAALFM_C105500WA
gene-CAALFM_C504710WA
gene-CAALFM_C300670CA
gene-CAALFM_C404460CA
gene-CAALFM_C303440CA
gene-CAALFM_C406900WA
gene-CAALFM_C108870CA
gene-CAALFM_C701630WA
gene-CAALFM_C300580WA
gene-CAALFM_C200710WA
gene-CAALFM_C406600WA
gene-CAALFM_C202250CA
gene-CAALFM_C203540WA
gene-CAALFM_C111280WA
gene-CAALFM_C504740CA
gene-CAALFM_C502300CA

gene-CAALFM_C105360CA	gene-CAALFM_C702540WA
gene-CAALFM_CR01200WA	gene-CAALFM_CR05250CA
gene-CAALFM_C113170CA	gene-CAALFM_C505490CA
gene-CAALFM_C403290WA	gene-CAALFM_C206360CA
gene-CAALFM_C300510WA	gene-CAALFM_C209890WA
gene-CAALFM_CR02850CA	gene-CAALFM_C113880CA
gene-CAALFM_C203560CA	gene-CAALFM_C110910CA
gene-CAALFM_C702120CA	gene-CAALFM_C405530WA
gene-CAALFM_CR06380CA	gene-CAALFM_C700160CA
gene-CAALFM_C200300CA	gene-CAALFM_C105140WA
gene-CAALFM_C201400CA	gene-CAALFM_C504780WA
gene-CAALFM_C603700WA	gene-CAALFM_C103740WA
gene-CAALFM_C302980CA	gene-CAALFM_C503540CA
gene-CAALFM_C302910WA	gene-CAALFM_CR04010CA
gene-CAALFM_C201420CA	gene-CAALFM_C107380CA
gene-CAALFM_CR02930WA	gene-CAALFM_C206200CA
gene-CAALFM_C201130WA	gene-CAALFM_C110090CA
gene-CAALFM_C114330WA	gene-CAALFM_C500090CA
gene-CAALFM_C209500WA	gene-CAALFM_C202100WA
gene-CAALFM_C300850CA	gene-CAALFM_C602090CA
gene-CAALFM_C305960WA	gene-CAALFM_C209370CA
gene-CAALFM_C403270WA	gene-CAALFM_C304300CA
gene-CAALFM_C307600WA	gene-CAALFM_C209580WA
gene-CAALFM_C603550CA	gene-CAALFM_C209500WA
gene-CAALFM_C109860CA	gene-CAALFM_C112410CA
gene-CAALFM_C100160CA	gene-CAALFM_C505380WA
gene-CAALFM_C700310CA	gene-CAALFM_C406190CA
gene-CAALFM_CR04180CA	gene-CAALFM_C101000CA
gene-CAALFM_C502550CA	gene-CAALFM_C407160WA
gene-CAALFM_C207610CA	gene-CAALFM_C503690WA
gene-CAALFM_C100960CA	gene-CAALFM_C602290CA
gene-CAALFM_C112550CA	gene-CAALFM_C603700WA
gene-CAALFM_C501850CA	gene-CAALFM_C104660WA
gene-CAALFM_C405660CA	gene-CAALFM_CR06680CA
gene-CAALFM_C112050WA	gene-CAALFM_C702060WA
gene-CAALFM_C306380WA	gene-CAALFM_C200450CA
gene-CAALFM_C306160CA	gene-CAALFM_C111520CA
gene-CAALFM_C306520CA	gene-CAALFM_CR03100WA
gene-CAALFM_C201680CA	gene-CAALFM_C111930WA
gene-CAALFM_C110260CA	gene-CAALFM_C301530CA
gene-CAALFM_C304320WA	gene-CAALFM_CR04300WA
gene-CAALFM_C104260WA	gene-CAALFM_C210810WA

gene-CAALFM_C110730WA
gene-CAALFM_C105090WA
gene-CAALFM_CR03380WA
gene-CAALFM_C701630WA
gene-CAALFM_C700200WA
gene-CAALFM_C603140CA
gene-CAALFM_C502780WA
gene-CAALFM_C503490CA
gene-CAALFM_C111360WA
gene-CAALFM_C109570WA
gene-CAALFM_CR08500WA
gene-CAALFM_CR05460WA
gene-CAALFM_C504050WA
gene-CAALFM_C104370CA
gene-CAALFM_C110880WA
gene-CAALFM_CR00980CA
gene-CAALFM_C602780CA
gene-CAALFM_C204870CA
gene-CAALFM_CR03100WA
gene-CAALFM_C501060CA
gene-CAALFM_C601850WA
gene-CAALFM_C303190CA
gene-CAALFM_C306070CA
gene-CAALFM_C700970CA
gene-CAALFM_C407080CA
gene-CAALFM_C404860WA
gene-CAALFM_C104360CA
gene-CAALFM_C600340CA
gene-CAALFM_CR09520CA
gene-CAALFM_C603950CA
gene-CAALFM_C503540CA
gene-CAALFM_C504510WA
gene-CAALFM_C101420CA
gene-CAALFM_C602930WA
gene-CAALFM_C401150WA
gene-CAALFM_C502190CA
gene-CAALFM_C505480WA
gene-CAALFM_C110640CA
gene-CAALFM_C602350CA
gene-CAALFM_C302220WA

gene-CAALFM_C400860CA
gene-CAALFM_C401450WA
gene-CAALFM_C111000CA
gene-CAALFM_C500160WA
gene-CAALFM_C307290WA
gene-CAALFM_C210820CA
gene-CAALFM_C402170CA
gene-CAALFM_C602310WA
gene-CAALFM_C100430WA
gene-CAALFM_C501060CA
gene-CAALFM_C112440WA
gene-CAALFM_C104490WA
gene-CAALFM_C201390WA
gene-CAALFM_C207340WA
gene-CAALFM_C505390CA
gene-CAALFM_CR00080WA
gene-CAALFM_C114380CA
gene-CAALFM_C112230WA
gene-CAALFM_C300280CA
gene-CAALFM_C504050WA
gene-CAALFM_C505270CA
gene-CAALFM_C201680CA
gene-CAALFM_C601740CA
gene-CAALFM_C404560CA
gene-CAALFM_C208450WA
gene-CAALFM_C113550CA
gene-CAALFM_C307770CA
gene-CAALFM_C501130WA
gene-CAALFM_C101560WA
gene-CAALFM_C300450CA
gene-CAALFM_C200030WA
gene-CAALFM_CR04180CA
gene-CAALFM_C600890WA
gene-CAALFM_CR09860WA
gene-CAALFM_C104990CA
gene-CAALFM_C105740CA
gene-CAALFM_CR08450CA
gene-CAALFM_C100840CA
gene-CAALFM_C504510WA
gene-CAALFM_C403440CA

gene-CAALFM_C103830CA
gene-CAALFM_C207040WA
gene-CAALFM_C206320WA
gene-CAALFM_C108520CA
gene-CAALFM_C200570WA
gene-CAALFM_C101860WA
gene-CAALFM_C112230WA
gene-CAALFM_CR01110WA
gene-CAALFM_C111560CA
gene-CAALFM_C603100WA
gene-CAALFM_C106250WA
gene-CAALFM_C109790CA
gene-CAALFM_C501560CA
gene-CAALFM_C101380CA
gene-CAALFM_C209020WA
gene-CAALFM_C700510WA
gene-CAALFM_C701760CA
gene-CAALFM_C306230WA
gene-CAALFM_C702910WA
gene-CAALFM_C306300WA
gene-CAALFM_C601630WA
gene-CAALFM_C109510WA
gene-CAALFM_C303660WA
gene-CAALFM_C300670CA
gene-CAALFM_C200410CA
gene-CAALFM_C404050CA
gene-CAALFM_C300290WA
gene-CAALFM_C504580CA
gene-CAALFM_C304820CA
gene-CAALFM_C500940CA
gene-CAALFM_C307640CA
gene-CAALFM_C202720WA
gene-CAALFM_C700570WA
gene-CAALFM_C602610CA
gene-CAALFM_C503360WA
gene-CAALFM_C701340WA
gene-CAALFM_C404660CA
gene-CAALFM_C206830CA
gene-CAALFM_C601980CA
gene-CAALFM_C305920WA
gene-CAALFM_C200710WA
gene-CAALFM_C108640WA
gene-CAALFM_C110840CA

gene-CAALFM_C304350CA
gene-CAALFM_C303660WA
gene-CAALFM_C600690WA
gene-CAALFM_CR04140WA
gene-CAALFM_C603550CA
gene-CAALFM_C704010WA
gene-CAALFM_C406980WA
gene-CAALFM_C109540WA
gene-CAALFM_C208000CA
gene-CAALFM_C103460CA
gene-CAALFM_C113180WA
gene-CAALFM_C702110WA
gene-CAALFM_C201890WA
gene-CAALFM_C504410CA
gene-CAALFM_C203950WA
gene-CAALFM_C209920WA
gene-CAALFM_C307730WA
gene-CAALFM_C604530CA
gene-CAALFM_CR04340WA
gene-CAALFM_C701070CA
gene-CAALFM_C600430CA
gene-CAALFM_C307250WA
gene-CAALFM_C500860WA
gene-CAALFM_C210740CA
gene-CAALFM_C110670CA
gene-CAALFM_C204870CA
gene-CAALFM_CR10440WA
gene-CAALFM_C300570CA
gene-CAALFM_C402850WA
gene-CAALFM_C504600CA
gene-CAALFM_C503360WA
gene-CAALFM_C207610CA
gene-CAALFM_C702790CA
gene-CAALFM_C208900WA
gene-CAALFM_C400370WA
gene-CAALFM_C403120CA
gene-CAALFM_C304260WA
gene-CAALFM_C304990WA
gene-CAALFM_C110840CA
gene-CAALFM_C603100WA
gene-CAALFM_C600530CA
gene-CAALFM_C300240CA
gene-CAALFM_C603940CA

gene-CAALFM_C110090CA	gene-CAALFM_C502650CA
gene-CAALFM_C200220CA	gene-CAALFM_C205490WA
gene-CAALFM_CR04580WA	gene-CAALFM_C103350CA
gene-CAALFM_C304990WA	gene-CAALFM_C307020WA
gene-CAALFM_C600530CA	gene-CAALFM_C503170CA
gene-CAALFM_C504350CA	gene-CAALFM_CR00440CA
gene-CAALFM_C502150CA	gene-CAALFM_C113940WA
gene-CAALFM_C403120CA	gene-CAALFM_C503900WA
gene-CAALFM_C306260CA	gene-CAALFM_C307300WA
gene-CAALFM_C101070CA	gene-CAALFM_C104810WA
gene-CAALFM_C305170WA	gene-CAALFM_C114040WA
gene-CAALFM_C500400CA	gene-CAALFM_C112010CA
gene-CAALFM_C113940WA	gene-CAALFM_C303770CA
gene-CAALFM_CR02710WA	gene-CAALFM_CR04050CA
gene-CAALFM_C207520CA	gene-CAALFM_C100600WA
gene-CAALFM_C501220WA	gene-CAALFM_C301520CA
gene-CAALFM_C600690WA	gene-CAALFM_C501390CA
gene-CAALFM_C101000CA	gene-CAALFM_C503580CA
gene-CAALFM_CR09370WA	gene-CAALFM_C603810WA
gene-CAALFM_C701450CA	gene-CAALFM_CR04160CA
gene-CAALFM_CR04380CA	gene-CAALFM_C703670WA
gene-CAALFM_C504570CA	gene-CAALFM_C114330WA
gene-CAALFM_C105340CA	gene-CAALFM_C210310CA
gene-CAALFM_CR05730CA	gene-CAALFM_C208980CA
gene-CAALFM_C301530CA	gene-CAALFM_C504880CA
gene-CAALFM_C110030WA	gene-CAALFM_CR07060CA
gene-CAALFM_C501870WA	gene-CAALFM_CR01690CA
gene-CAALFM_C600660CA	gene-CAALFM_C406800WA
gene-CAALFM_C404500CA	gene-CAALFM_CR07720CA
gene-CAALFM_C402900CA	gene-CAALFM_C404640CA
gene-CAALFM_C205160CA	gene-CAALFM_CR01700CA
gene-CAALFM_C307370WA	gene-CAALFM_C108420WA
gene-CAALFM_C209580WA	gene-CAALFM_C107110WA
gene-CAALFM_C113350WA	gene-CAALFM_CR01820WA
gene-CAALFM_C201030WA	gene-CAALFM_C402160CA
gene-CAALFM_C111220CA	gene-CAALFM_C205910WA
gene-CAALFM_C300270CA	gene-CAALFM_C402270CA
gene-CAALFM_C400370WA	gene-CAALFM_C501430CA
gene-CAALFM_C401450WA	gene-CAALFM_CR09520CA
gene-CAALFM_C407100CA	gene-CAALFM_C601040CA
gene-CAALFM_C202100WA	gene-CAALFM_C110470WA

gene-CAALFM_C300710WA	gene-CAALFM_C101080WA
gene-CAALFM_C300570CA	gene-CAALFM_CR01240CA
gene-CAALFM_C105400CA	gene-CAALFM_C405080CA
gene-CAALFM_C401260WA	gene-CAALFM_CR03840CA
gene-CAALFM_C110020WA	gene-CAALFM_C304860WA
gene-CAALFM_C304120CA	gene-CAALFM_C101380CA
gene-CAALFM_C500330CA	gene-CAALFM_CR03890WA
gene-CAALFM_C601830WA	gene-CAALFM_C500030WA
gene-CAALFM_C404650WA	gene-CAALFM_C303260WA
gene-CAALFM_C303100CA	gene-CAALFM_C201200CA
gene-CAALFM_C400820WA	gene-CAALFM_C103560CA
gene-CAALFM_C300240CA	gene-CAALFM_C205160CA
gene-CAALFM_CR03340CA	gene-CAALFM_C502440CA
gene-CAALFM_C303150WA	gene-CAALFM_C401220CA
gene-CAALFM_C307250WA	gene-CAALFM_C209230CA
gene-CAALFM_C600800CA	gene-CAALFM_CR08020CA
gene-CAALFM_C108120WA	gene-CAALFM_CR03490WA
gene-CAALFM_CR03080CA	gene-CAALFM_CR00400CA
gene-CAALFM_C305150WA	gene-CAALFM_C401000CA
gene-CAALFM_C402790CA	gene-CAALFM_C301100WA
gene-CAALFM_C202350CA	gene-CAALFM_C502430WA
gene-CAALFM_CR05620CA	gene-CAALFM_C105900WA
gene-CAALFM_C600110CA	gene-CAALFM_CR02930WA
gene-CAALFM_C304260WA	gene-CAALFM_C306520CA
gene-CAALFM_C404750WA	gene-CAALFM_C401700CA
gene-CAALFM_C504750CA	gene-CAALFM_C104370CA
gene-CAALFM_C304470WA	gene-CAALFM_C501300CA
gene-CAALFM_CR05260CA	gene-CAALFM_CR03350CA
gene-CAALFM_C112350WA	gene-CAALFM_C203800CA
gene-CAALFM_C602630CA	gene-CAALFM_C100520WA
gene-CAALFM_C109670CA	gene-CAALFM_C501970CA
gene-CAALFM_C504710WA	gene-CAALFM_C400820WA
gene-CAALFM_C210610WA	gene-CAALFM_C106530CA
gene-CAALFM_C203570CA	gene-CAALFM_C307600WA
gene-CAALFM_C108730WA	gene-CAALFM_CR07890WA
gene-CAALFM_C701850CA	gene-CAALFM_CR01500WA
gene-CAALFM_C203140CA	gene-CAALFM_C406850CA
gene-CAALFM_CR10480WA	gene-CAALFM_C502190CA
gene-CAALFM_C110350CA	gene-CAALFM_C502120CA
gene-CAALFM_C111000CA	gene-CAALFM_C111190WA
gene-CAALFM_C303440CA	gene-CAALFM_C505510CA
gene-CAALFM_C304300CA	gene-CAALFM_C306470WA
gene-CAALFM_C207340WA	gene-CAALFM_C105090WA

gene-CAALFM_C303910WA	gene-CAALFM_C701170CA
gene-CAALFM_C103770WA	gene-CAALFM_CR07200WA
gene-CAALFM_C210740CA	gene-CAALFM_C300840CA
gene-CAALFM_C504900CA	gene-CAALFM_C502900WA
gene-CAALFM_C203940CA	gene-CAALFM_C105390CA
gene-CAALFM_C504880CA	gene-CAALFM_C600390WA
gene-CAALFM_C403030CA	gene-CAALFM_C200200WA
gene-CAALFM_C504430CA	gene-CAALFM_C306500WA
gene-CAALFM_C701660CA	gene-CAALFM_C302020WA
gene-CAALFM_C102960CA	gene-CAALFM_C204590CA
gene-CAALFM_C206480WA	gene-CAALFM_C106600WA
gene-CAALFM_C600230WA	gene-CAALFM_CR00980CA
gene-CAALFM_C300580WA	gene-CAALFM_C110230CA
gene-CAALFM_CR09880WA	gene-CAALFM_C101420CA
gene-CAALFM_C502730CA	gene-CAALFM_C405010WA
gene-CAALFM_C505270CA	gene-CAALFM_C302220WA
gene-CAALFM_C505400WA	gene-CAALFM_CR03370CA
gene-CAALFM_C406800WA	gene-CAALFM_C209660WA
gene-CAALFM_C306120CA	gene-CAALFM_C404500CA
gene-CAALFM_C306620WA	gene-CAALFM_C402870CA
gene-CAALFM_C103520WA	gene-CAALFM_C701450CA
gene-CAALFM_C201530CA	gene-CAALFM_CR02670CA
gene-CAALFM_C303750CA	gene-CAALFM_C505000CA
gene-CAALFM_C111520CA	gene-CAALFM_C500340WA
gene-CAALFM_CR04080CA	gene-CAALFM_C602210WA
gene-CAALFM_C404830WA	gene-CAALFM_C204080WA
gene-CAALFM_C203990WA	gene-CAALFM_C304120CA
gene-CAALFM_C106770WA	gene-CAALFM_C505290CA
gene-CAALFM_C113030CA	gene-CAALFM_C112680WA
gene-CAALFM_C113810WA	gene-CAALFM_CR00430CA
gene-CAALFM_C500290WA	gene-CAALFM_C303100CA
gene-CAALFM_C101470WA	gene-CAALFM_C701030CA
gene-CAALFM_C110670CA	gene-CAALFM_C502310CA
gene-CAALFM_C307770CA	gene-CAALFM_C111970CA
gene-CAALFM_C601960WA	gene-CAALFM_CR06060WA
gene-CAALFM_C604350CA	gene-CAALFM_C306210CA
gene-CAALFM_C302130WA	gene-CAALFM_C504630WA
gene-CAALFM_C503070WA	gene-CAALFM_C503550WA
gene-CAALFM_C104170CA	gene-CAALFM_CR08180CA
gene-CAALFM_C207100WA	gene-CAALFM_C402000CA
gene-CAALFM_C300500CA	gene-CAALFM_C103710CA
gene-CAALFM_C304860WA	gene-CAALFM_C503150WA
gene-CAALFM_CR10730CA	gene-CAALFM_CR01760CA

gene-CAALFM_C108420WA	gene-CAALFM_C600310WA
gene-CAALFM_C602380WA	gene-CAALFM_C306110CA
gene-CAALFM_C302100WA	gene-CAALFM_C406230CA
gene-CAALFM_CR03710CA	gene-CAALFM_C701580WA
gene-CAALFM_C302940CA	gene-CAALFM_C502620CA
gene-CAALFM_C205380WA	gene-CAALFM_CR05670CA
gene-CAALFM_C209520CA	gene-CAALFM_C602740WA
gene-CAALFM_C302970CA	gene-CAALFM_C505240CA
gene-CAALFM_C100660CA	gene-CAALFM_C113830CA
gene-CAALFM_C102710WA	gene-CAALFM_C603450CA
gene-CAALFM_CR07450CA	gene-CAALFM_C205720CA
gene-CAALFM_C100100CA	gene-CAALFM_CR09440CA
gene-CAALFM_C113730CA	gene-CAALFM_C404260CA
gene-CAALFM_C110120CA	gene-CAALFM_C600800CA
gene-CAALFM_C406850CA	gene-CAALFM_C203200WA
gene-CAALFM_C103350CA	gene-CAALFM_CR03460WA
gene-CAALFM_C504490CA	gene-CAALFM_C700550CA
gene-CAALFM_C207350WA	gene-CAALFM_CR10410CA
gene-CAALFM_C112680WA	gene-CAALFM_CR01780WA
gene-CAALFM_C109910CA	gene-CAALFM_C701850CA
gene-CAALFM_CR10670WA	gene-CAALFM_C206000WA
gene-CAALFM_C209660WA	gene-CAALFM_C107990CA
gene-CAALFM_C303170WA	gene-CAALFM_C111220CA
gene-CAALFM_CR01610CA	gene-CAALFM_C101270WA
gene-CAALFM_C503570WA	gene-CAALFM_C200730CA
gene-CAALFM_CR08030CA	gene-CAALFM_C207110CA
gene-CAALFM_C406680CA	gene-CAALFM_C201260WA
gene-CAALFM_C500680WA	gene-CAALFM_C109670CA
gene-CAALFM_C200600CA	gene-CAALFM_C111770CA
gene-CAALFM_CR08070WA	gene-CAALFM_C306710WA
gene-CAALFM_CR01760CA	gene-CAALFM_CR08490WA
gene-CAALFM_C306500WA	gene-CAALFM_C200820WA
gene-CAALFM_C406660WA	gene-CAALFM_C105610WA
gene-CAALFM_C208310WA	gene-CAALFM_C302030WA
gene-CAALFM_C109810WA	gene-CAALFM_C304230WA
gene-CAALFM_C205910WA	gene-CAALFM_C502630CA
gene-CAALFM_C202710CA	gene-CAALFM_CR01420WA
gene-CAALFM_C106910CA	gene-CAALFM_C200300CA
gene-CAALFM_CR03490WA	gene-CAALFM_CR00630WA
gene-CAALFM_C201120WA	gene-CAALFM_C504580CA
gene-CAALFM_C500050WA	gene-CAALFM_C500250CA

gene-CAALFM_C302110WA
gene-CAALFM_C500340WA
gene-CAALFM_C503090WA
gene-CAALFM_C205470WA
gene-CAALFM_C307900CA
gene-CAALFM_C105110CA
gene-CAALFM_C302780WA
gene-CAALFM_C302770CA
gene-CAALFM_C703670WA
gene-CAALFM_C110750CA
gene-CAALFM_CR05180CA
gene-CAALFM_C505390CA
gene-CAALFM_C700550CA
gene-CAALFM_C113320CA

represents common genes

gene-CAALFM_CR04380CA
gene-CAALFM_C203980CA
gene-CAALFM_CR03660CA
gene-CAALFM_CR02640WA
gene-CAALFM_C304520CA
gene-CAALFM_C505230CA
gene-CAALFM_C200610CA
gene-CAALFM_C107010WA
gene-CAALFM_C402640CA
gene-CAALFM_C402500CA
gene-CAALFM_C702120CA
gene-CAALFM_C500790CA
gene-CAALFM_CR06710CA
gene-CAALFM_C205260WA
gene-CAALFM_CR05840WA
gene-CAALFM_CR05620CA
gene-CAALFM_C209520CA
gene-CAALFM_C702320WA
gene-CAALFM_C602930WA
gene-CAALFM_C604250WA
gene-CAALFM_C703480WA
gene-CAALFM_CR05460WA
gene-CAALFM_C601630WA
gene-CAALFM_C503090WA
gene-CAALFM_C406940CA
gene-CAALFM_C503650CA
gene-CAALFM_CR03940WA
gene-CAALFM_C110580CA
gene-CAALFM_C303930WA
gene-CAALFM_C300710WA
gene-CAALFM_C302910WA
gene-CAALFM_C302980CA
gene-CAALFM_C101590CA
gene-CAALFM_C601460CA
gene-CAALFM_C108600CA
gene-CAALFM_C703220CA
gene-CAALFM_C106670WA
gene-CAALFM_C303920WA
gene-CAALFM_C203550CA
gene-CAALFM_C202720WA
gene-CAALFM_C500330CA
gene-CAALFM_C203990WA
gene-CAALFM_C503490CA
gene-CAALFM_C201400CA

gene-CAALFM_C113320CA
gene-CAALFM_CR02330CA
gene-CAALFM_CR01580CA
gene-CAALFM_C111450CA
gene-CAALFM_C203090CA
gene-CAALFM_C602350CA
gene-CAALFM_C702940CA
gene-CAALFM_C503070WA
gene-CAALFM_C105430WA
gene-CAALFM_C207360WA
gene-CAALFM_CR09760WA
gene-CAALFM_C301770CA
gene-CAALFM_CR09880WA
gene-CAALFM_C503670CA
gene-CAALFM_C307900CA
gene-CAALFM_CR03340CA
gene-CAALFM_C107270WA
gene-CAALFM_C200410CA
gene-CAALFM_C107260CA
gene-CAALFM_C100980WA
gene-CAALFM_C503380WA
gene-CAALFM_C105110CA
gene-CAALFM_CR01710WA
gene-CAALFM_C501640WA
gene-CAALFM_C603690WA
gene-CAALFM_C110920WA
gene-CAALFM_C601830WA
gene-CAALFM_CR01570WA
gene-CAALFM_C109860CA
gene-CAALFM_C500380WA
gene-CAALFM_CR00680WA
gene-CAALFM_C200220CA
gene-CAALFM_C304820CA
gene-CAALFM_C209310CA
gene-CAALFM_C304740CA
gene-CAALFM_C307370WA
gene-CAALFM_C403030CA
gene-CAALFM_CR03060WA
gene-CAALFM_C700350CA
gene-CAALFM_C504160WA
gene-CAALFM_C201130WA
gene-CAALFM_C112050WA
gene-CAALFM_C501530CA
gene-CAALFM_C202690WA

gene-CAALFM_C209610WA
gene-CAALFM_C108640WA
gene-CAALFM_C100910WA
gene-CAALFM_C104360CA
gene-CAALFM_C100930CA
gene-CAALFM_C602380WA
gene-CAALFM_C500180WA
gene-CAALFM_C406680CA
gene-CAALFM_CR07440WA
gene-CAALFM_C206470WA
gene-CAALFM_C504350CA
gene-CAALFM_C203780CA
gene-CAALFM_CR03750CA
gene-CAALFM_C100150CA
gene-CAALFM_CR05260CA
gene-CAALFM_C111070WA
gene-CAALFM_C700620WA
gene-CAALFM_C700340CA
gene-CAALFM_CR05530CA
gene-CAALFM_C110350CA
gene-CAALFM_CR07950WA
gene-CAALFM_C701270CA
gene-CAALFM_C501560CA
gene-CAALFM_C601850WA
gene-CAALFM_C103070CA
gene-CAALFM_C501840CA
gene-CAALFM_C100230CA
gene-CAALFM_CR07600WA
gene-CAALFM_C107020CA
gene-CAALFM_C406000WA
gene-CAALFM_C112350WA
gene-CAALFM_C302970CA
gene-CAALFM_C207500CA
gene-CAALFM_C200600CA
gene-CAALFM_CR10730CA
gene-CAALFM_C102420CA
gene-CAALFM_C200100CA
gene-CAALFM_C500290WA
gene-CAALFM_C109210CA
gene-CAALFM_C108050WA
gene-CAALFM_C113810WA
gene-CAALFM_C204120CA
gene-CAALFM_C200360CA
gene-CAALFM_CR00280CA

gene-CAALFM_C304680WA
gene-CAALFM_C501940WA
gene-CAALFM_C601960WA
gene-CAALFM_C500950CA
gene-CAALFM_C203570CA
gene-CAALFM_C402790CA
gene-CAALFM_C112290CA
gene-CAALFM_C505500CA
gene-CAALFM_C108110WA
gene-CAALFM_C700310CA
gene-CAALFM_C305610WA
gene-CAALFM_CR07310WA
gene-CAALFM_C307400WA
gene-CAALFM_C302940CA
gene-CAALFM_C303340CA
gene-CAALFM_C600340CA
gene-CAALFM_CR04390CA
gene-CAALFM_C305150WA
gene-CAALFM_C304470WA
gene-CAALFM_C404860WA
gene-CAALFM_C700070CA
gene-CAALFM_C209720WA
gene-CAALFM_C306260CA
gene-CAALFM_CR08440WA
gene-CAALFM_C101820CA
gene-CAALFM_C502070CA
gene-CAALFM_C602600WA
gene-CAALFM_CR03080CA
gene-CAALFM_C300510WA
gene-CAALFM_C602610CA
gene-CAALFM_C205710CA
gene-CAALFM_C403290WA
gene-CAALFM_C108700WA
gene-CAALFM_C502670WA
gene-CAALFM_C103040WA
gene-CAALFM_CR00910WA
gene-CAALFM_CR10640WA
gene-CAALFM_C207230CA
gene-CAALFM_C500500WA
gene-CAALFM_C504830WA
gene-CAALFM_C602690CA
gene-CAALFM_CR01200WA
gene-CAALFM_C503570WA
gene-CAALFM_CR08030CA

gene-CAALFM_C307660WA
gene-CAALFM_C100540CA
gene-CAALFM_C102710WA
gene-CAALFM_C602280WA
gene-CAALFM_C103370WA
gene-CAALFM_C304760CA
gene-CAALFM_CR04600WA
gene-CAALFM_C203030WA
gene-CAALFM_C503640WA
gene-CAALFM_C503700CA
gene-CAALFM_C109430WA
gene-CAALFM_C107340WA
gene-CAALFM_C602520WA
gene-CAALFM_CR05760CA
gene-CAALFM_CR07630CA
gene-CAALFM_C601710CA
gene-CAALFM_CR10480WA
gene-CAALFM_C108550CA
gene-CAALFM_C102960CA
gene-CAALFM_C109570WA
gene-CAALFM_C702350CA
gene-CAALFM_CR00110WA
gene-CAALFM_C113350WA
gene-CAALFM_C600520WA
gene-CAALFM_C600550WA
gene-CAALFM_C701020CA
gene-CAALFM_C304490WA
gene-CAALFM_C105400CA
gene-CAALFM_C203560CA
gene-CAALFM_C106090CA
gene-CAALFM_C200380CA
gene-CAALFM_CR04560CA
gene-CAALFM_C302000WA
gene-CAALFM_C106770WA
gene-CAALFM_CR02710WA
gene-CAALFM_C404830WA
gene-CAALFM_CR08320WA
gene-CAALFM_CR04080CA
gene-CAALFM_C202340CA
gene-CAALFM_C202520WA
gene-CAALFM_C307640CA
gene-CAALFM_C304290CA
gene-CAALFM_C404910CA
gene-CAALFM_C108730WA

gene-CAALFM_C400890WA
gene-CAALFM_CR01360WA
gene-CAALFM_C202780CA
gene-CAALFM_C209270CA
gene-CAALFM_C103310WA
gene-CAALFM_C113970CA
gene-CAALFM_CR03680CA
gene-CAALFM_C701760CA
gene-CAALFM_C109730WA
gene-CAALFM_CR07450CA
gene-CAALFM_C201030WA
gene-CAALFM_C202760WA
gene-CAALFM_C110120CA
gene-CAALFM_C208110WA
gene-CAALFM_C307550CA
gene-CAALFM_C205920CA
gene-CAALFM_C500050WA
gene-CAALFM_C300500CA
gene-CAALFM_C103790CA
gene-CAALFM_CR02910WA
gene-CAALFM_C206300WA
gene-CAALFM_C405060WA
gene-CAALFM_C302260CA
gene-CAALFM_C111130WA
gene-CAALFM_C207520CA
gene-CAALFM_C603950CA
gene-CAALFM_C302110WA
gene-CAALFM_C306850WA
gene-CAALFM_C406660WA
gene-CAALFM_C400330CA
gene-CAALFM_CR02420WA
gene-CAALFM_C502180CA
gene-CAALFM_C108390CA
gene-CAALFM_C604450WA
gene-CAALFM_C303790WA
gene-CAALFM_C207660WA
gene-CAALFM_C110640CA
gene-CAALFM_C103200CA
gene-CAALFM_C700220WA
gene-CAALFM_C102850WA
gene-CAALFM_CR07350WA
gene-CAALFM_CR00180CA
gene-CAALFM_C703660CA
gene-CAALFM_C602630CA

gene-CAALFM_C200210WA
gene-CAALFM_C206480WA
gene-CAALFM_C701660CA
gene-CAALFM_C302100WA
gene-CAALFM_C304360WA
gene-CAALFM_C500400CA
gene-CAALFM_C501870WA
gene-CAALFM_C701430CA
gene-CAALFM_C204360WA
gene-CAALFM_C100660CA
gene-CAALFM_C504430CA
gene-CAALFM_C306830CA
gene-CAALFM_CR08960CA
gene-CAALFM_C704270CA
gene-CAALFM_C300290WA
gene-CAALFM_C111560CA
gene-CAALFM_C505170WA
gene-CAALFM_C303190CA
gene-CAALFM_C700560CA
gene-CAALFM_C208310WA
gene-CAALFM_C404750WA
gene-CAALFM_C202960CA
gene-CAALFM_C110730WA
gene-CAALFM_C105680CA
gene-CAALFM_C404580WA
gene-CAALFM_C101470WA
gene-CAALFM_C302190CA
gene-CAALFM_C201530CA
gene-CAALFM_C207390CA
gene-CAALFM_C202350CA
gene-CAALFM_CR07240CA
gene-CAALFM_C306690CA
gene-CAALFM_C500680WA
gene-CAALFM_C108520CA
gene-CAALFM_C206320WA
gene-CAALFM_C110380CA
gene-CAALFM_C601980CA
gene-CAALFM_C104480CA
gene-CAALFM_C103360WA
gene-CAALFM_CR08470WA
gene-CAALFM_C101320WA
gene-CAALFM_C200400CA
gene-CAALFM_C113030CA
gene-CAALFM_C701340WA

gene-CAALFM_C100100CA
gene-CAALFM_CR07470WA
gene-CAALFM_C501960CA
gene-CAALFM_C501770CA
gene-CAALFM_C703960CA
gene-CAALFM_C502030WA
gene-CAALFM_C306620WA
gene-CAALFM_C301610WA
gene-CAALFM_C302780WA
gene-CAALFM_C600660CA
gene-CAALFM_C205680WA
gene-CAALFM_CR01110WA
gene-CAALFM_C103060CA
gene-CAALFM_C501820WA
gene-CAALFM_C502730CA
gene-CAALFM_C505280CA
gene-CAALFM_C110020WA
gene-CAALFM_C103520WA
gene-CAALFM_C302670WA
gene-CAALFM_C503350WA
gene-CAALFM_CR03400WA
gene-CAALFM_CR03500WA
gene-CAALFM_C105340CA
gene-CAALFM_C201120WA
gene-CAALFM_C110010CA
gene-CAALFM_C400490WA
gene-CAALFM_C305920WA
gene-CAALFM_CR03250CA
gene-CAALFM_C504700CA
gene-CAALFM_C209640WA
gene-CAALFM_C102750CA
gene-CAALFM_C302130WA
gene-CAALFM_C502150CA
gene-CAALFM_C203140CA
gene-CAALFM_CR02530WA
gene-CAALFM_C701210CA
gene-CAALFM_C202820CA
gene-CAALFM_C704140CA
gene-CAALFM_CR03710CA
gene-CAALFM_C206230WA
gene-CAALFM_C303150WA
gene-CAALFM_C205470WA
gene-CAALFM_C302160CA
gene-CAALFM_C504720CA

gene-CAALFM_CR05180CA
gene-CAALFM_CR08630WA
gene-CAALFM_C504130CA
gene-CAALFM_CR02140WA
gene-CAALFM_C208270CA
gene-CAALFM_C403070WA
gene-CAALFM_C701690WA
gene-CAALFM_C303170WA
gene-CAALFM_C104530CA
gene-CAALFM_C501580CA
gene-CAALFM_CR08070WA
gene-CAALFM_C204170CA
gene-CAALFM_C501250WA
gene-CAALFM_C307680WA
gene-CAALFM_C108350CA
gene-CAALFM_C104330WA
gene-CAALFM_CR02970CA
gene-CAALFM_C501290CA
gene-CAALFM_C111400CA
gene-CAALFM_CR10670WA
gene-CAALFM_C300360WA
gene-CAALFM_C307410CA
gene-CAALFM_C401940WA
gene-CAALFM_CR03530WA
gene-CAALFM_C300050CA
gene-CAALFM_C503830CA
gene-CAALFM_C103830CA
gene-CAALFM_C111210CA
gene-CAALFM_C109010WA
gene-CAALFM_CR03540WA
gene-CAALFM_C200320WA
gene-CAALFM_C504490CA
gene-CAALFM_C206150CA
gene-CAALFM_CR05540CA
gene-CAALFM_C604610CA
gene-CAALFM_C105290WA
gene-CAALFM_C504900CA
gene-CAALFM_C307500WA
gene-CAALFM_C111730WA
gene-CAALFM_C103640CA
gene-CAALFM_C400400WA
gene-CAALFM_C503590WA
gene-CAALFM_C701560CA
gene-CAALFM_C204040CA

gene-CAALFM_C208040CA
gene-CAALFM_C205380WA
gene-CAALFM_C600230WA
gene-CAALFM_C114260CA
gene-CAALFM_C306120CA
gene-CAALFM_CR01610CA
gene-CAALFM_C100480CA
gene-CAALFM_C406110CA
gene-CAALFM_CR02850CA
gene-CAALFM_C502700WA
gene-CAALFM_C302770CA
gene-CAALFM_CR08410WA
gene-CAALFM_C302510CA
gene-CAALFM_C110420CA
gene-CAALFM_C502060WA
gene-CAALFM_C406120WA
gene-CAALFM_C407180WA
gene-CAALFM_CR08120CA
gene-CAALFM_C306230WA
gene-CAALFM_C501930WA
gene-CAALFM_C101370CA
gene-CAALFM_C603140CA
gene-CAALFM_C404650WA
gene-CAALFM_CR07650WA
gene-CAALFM_C304940WA
gene-CAALFM_C300440WA
gene-CAALFM_C109910CA
gene-CAALFM_CR03920CA
gene-CAALFM_C306340WA
gene-CAALFM_C702910WA
gene-CAALFM_CR03790CA
gene-CAALFM_C112250CA
gene-CAALFM_CR00860CA
gene-CAALFM_C300850CA
gene-CAALFM_C501850CA
gene-CAALFM_C202260WA
gene-CAALFM_CR10750CA
gene-CAALFM_C305170WA
gene-CAALFM_CR07010WA
gene-CAALFM_C603660CA
gene-CAALFM_C110750CA
gene-CAALFM_C302410CA
gene-CAALFM_C303750CA
gene-CAALFM_CR04580WA

gene-CAALFM_C207550WA
gene-CAALFM_C205110WA
gene-CAALFM_C202710CA
gene-CAALFM_C208790WA
gene-CAALFM_C701680CA
gene-CAALFM_C406720WA
gene-CAALFM_C200280CA
gene-CAALFM_C106580WA
gene-CAALFM_C201170CA
gene-CAALFM_C201490CA
gene-CAALFM_CR03560WA
