# Supplementary material for: DNA Damage Checkpoints Govern Global Gene Transcription and Exhibit Species-Specific Regulation on HOF1 in Candida albicans
Source: J Fungi (Basel). 2024 May 29;10(6):387. doi: 10.3390/jof10060387 (PMC11204775; doi:10.3390/jof10060387)
Supplement: Supplementary file 1 [file jof-10-00387-s001.zip › Table S6.pdf]

| No MMS | With MMS |
| --- | --- |
| gene-CAALFM_C104680WA | gene-CAALFM_C109720WA |
| gene-CAALFM_C108260CA | gene-CAALFM_C206820CA |
| gene-CAALFM_C112790CA | gene-CAALFM_C405360CA |
| gene-CAALFM_C403890WA | gene-CAALFM_C401120CA |
| gene-CAALFM_C403960WA | gene-CAALFM_C300950CA |
| gene-CAALFM_C502560CA | gene-CAALFM_CR04820WA |
| gene-CAALFM_CR00760CA | gene-CAALFM_CR04420CA |
| gene-CAALFM_C405550CA | gene-CAALFM_CR03630WA |
| gene-CAALFM_C305730CA | gene-CAALFM_C201350CA |
| gene-CAALFM_CR08920WA | gene-CAALFM_C402050WA |
| gene-CAALFM_C503120WA | gene-CAALFM_C113400CA |
| gene-CAALFM_C104340CA | gene-CAALFM_C205000CA |
| gene-CAALFM_C701830WA | gene-CAALFM_C103720CA |
| gene-CAALFM_C114030WA | gene-CAALFM_C202110CA |
| gene-CAALFM_C600620WA | gene-CAALFM_C111700CA |
| gene-CAALFM_C400160CA | gene-CAALFM_C206890CA |
| gene-CAALFM_C104210CA | gene-CAALFM_C405560CA |
| gene-CAALFM_C704300WA | gene-CAALFM_C112740WA |
| gene-CAALFM_C602950CA | gene-CAALFM_C301500CA |
| gene-CAALFM_C703040WA | gene-CAALFM_C111240CA |
| gene-CAALFM_C700720WA | gene-CAALFM_C103610CA |
| gene-CAALFM_C203870WA | gene-CAALFM_CR03860CA |
| gene-CAALFM_C302170CA | gene-CAALFM_C111110CA |
| gene-CAALFM_C204340CA | gene-CAALFM_CR02360WA |
| gene-CAALFM_C208740WA | gene-CAALFM_C401780CA |
| gene-CAALFM_C107710CA | gene-CAALFM_C305290CA |
| gene-CAALFM_CR05210WA | gene-CAALFM_CR03620CA |
| gene-CAALFM_CR04060CA | gene-CAALFM_C200810CA |
| gene-CAALFM_C701480WA | gene-CAALFM_CR02770CA |
| gene-CAALFM_C101350CA | gene-CAALFM_C200270CA |
| gene-CAALFM_C407060WA | gene-CAALFM_C105560WA |
| gene-CAALFM_C402050WA | gene-CAALFM_C501650CA |
| gene-CAALFM_C600750CA | gene-CAALFM_C202160WA |
| gene-CAALFM_C404530CA | gene-CAALFM_C701910CA |
| gene-CAALFM_C403860CA | gene-CAALFM_C505190WA |
| gene-CAALFM_C603590CA | gene-CAALFM_C604330WA |
| gene-CAALFM_C300720WA | gene-CAALFM_C108570CA |
| gene-CAALFM_C112530CA | gene-CAALFM_C406390WA |
| gene-CAALFM_C110040WA | gene-CAALFM_C402080WA |
| gene-CAALFM_C110530WA | gene-CAALFM_C110830WA |
| gene-CAALFM_C400200CA | gene-CAALFM_C600900CA |
| gene-CAALFM_C114530WA | gene-CAALFM_C602870WA |
| gene-CAALFM_C112030WA | gene-CAALFM_C406160WA |

gene-CAALFM_C601420CA
gene-CAALFM_C204380CA
gene-CAALFM_C401560CA
gene-CAALFM_C204330CA
gene-CAALFM_C105710CA
gene-CAALFM_C405580CA
gene-CAALFM_C303230CA
gene-CAALFM_C208600WA
gene-CAALFM_C401730CA
gene-CAALFM_C207140WA
gene-CAALFM_C209390WA
gene-CAALFM_C100800CA
gene-CAALFM_CR04810WA
gene-CAALFM_C704240CA
gene-CAALFM_C405450CA
gene-CAALFM_CR10130WA
gene-CAALFM_CR10800CA
gene-CAALFM_C604430WA
gene-CAALFM_C102980WA
gene-CAALFM_C405130CA
gene-CAALFM_CR04150WA
gene-CAALFM_C505190WA
gene-CAALFM_CR09580CA
gene-CAALFM_CR02560CA
gene-CAALFM_C702560WA
gene-CAALFM_C405160CA
gene-CAALFM_C208330WA
gene-CAALFM_C109980CA
gene-CAALFM_C403730CA
gene-CAALFM_C700270WA
gene-CAALFM_C400680WA
gene-CAALFM_CR05720WA
gene-CAALFM_C307200CA
gene-CAALFM_CR07570WA
gene-CAALFM_C703630CA
gene-CAALFM_C109680WA
gene-CAALFM_C602540CA
gene-CAALFM_CR03210CA
gene-CAALFM_C302430WA
gene-CAALFM_C703020CA
gene-CAALFM_C107840WA
gene-CAALFM_CR00090CA
gene-CAALFM_CR10120CA
gene-CAALFM_C208200WA
gene-CAALFM_C109250WA
gene-CAALFM_C109690WA
gene-CAALFM_C602270CA
gene-CAALFM_C300760WA
gene-CAALFM_CR02570CA
gene-CAALFM_CR03600CA
gene-CAALFM_CR06330CA
gene-CAALFM_C111480WA
gene-CAALFM_C602000WA
gene-CAALFM_C604260CA
gene-CAALFM_C103380WA
gene-CAALFM_C602800WA
gene-CAALFM_C601430CA
gene-CAALFM_C301280WA
gene-CAALFM_C403200CA
gene-CAALFM_C403090WA
gene-CAALFM_C301810CA
gene-CAALFM_C307040CA
gene-CAALFM_C107540CA
gene-CAALFM_C400700CA
gene-CAALFM_C306870WA
gene-CAALFM_C204310WA
gene-CAALFM_CR05240CA
gene-CAALFM_C110500WA
gene-CAALFM_C403860CA
gene-CAALFM_C302140CA
gene-CAALFM_C402990CA
gene-CAALFM_C700050CA
gene-CAALFM_C405440CA
gene-CAALFM_C101940CA
gene-CAALFM_C102180WA
gene-CAALFM_C304710WA
gene-CAALFM_C202170WA
gene-CAALFM_C400710WA
gene-CAALFM_C401790WA
gene-CAALFM_C210090CA
gene-CAALFM_C101930WA
gene-CAALFM_CR10720WA
gene-CAALFM_C401300WA
gene-CAALFM_C305360CA
gene-CAALFM_C205230CA
gene-CAALFM_C104100CA
gene-CAALFM_C110240CA
gene-CAALFM_C111660WA

gene-CAALFM_C202310WA
gene-CAALFM_C108150CA
gene-CAALFM_C109070WA
gene-CAALFM_C109000WA
gene-CAALFM_C302150CA
gene-CAALFM_CR01040CA
gene-CAALFM_C604130CA
gene-CAALFM_C209670CA
gene-CAALFM_CR09750CA
gene-CAALFM_CR09160CA
gene-CAALFM_CR07700WA
gene-CAALFM_CR02790CA
gene-CAALFM_C112740WA
gene-CAALFM_C402550CA
gene-CAALFM_CR07830CA
gene-CAALFM_C701160CA
gene-CAALFM_C704310CA
gene-CAALFM_C406830CA
gene-CAALFM_C114120CA
gene-CAALFM_C204980CA
gene-CAALFM_CR09190CA
gene-CAALFM_C500390CA
gene-CAALFM_C402510WA
gene-CAALFM_C403940CA
gene-CAALFM_CR02770CA
gene-CAALFM_C500480CA
gene-CAALFM_C206710WA
gene-CAALFM_C303360WA
gene-CAALFM_CR08650CA
gene-CAALFM_C306600CA
gene-CAALFM_C703720CA
gene-CAALFM_C202460WA
gene-CAALFM_C700840CA
gene-CAALFM_C103610CA
gene-CAALFM_C208940CA
gene-CAALFM_C500430WA
gene-CAALFM_C203100WA
gene-CAALFM_C602680WA
gene-CAALFM_C102400CA
gene-CAALFM_C405220CA
gene-CAALFM_C604190CA
gene-CAALFM_C700700WA
gene-CAALFM_C404590WA
gene-CAALFM_C406410WA

gene-CAALFM_C602540CA
gene-CAALFM_C401750CA
gene-CAALFM_C101600WA
gene-CAALFM_C100890WA
gene-CAALFM_C102620CA
gene-CAALFM_C406570CA
gene-CAALFM_C600870CA
gene-CAALFM_C107060CA
gene-CAALFM_CR03690WA
gene-CAALFM_C307570CA
gene-CAALFM_C202200WA
gene-CAALFM_C603960WA
gene-CAALFM_CR05740CA
gene-CAALFM_C603620CA
gene-CAALFM_C404570WA
gene-CAALFM_C110310WA
gene-CAALFM_CR09670CA
gene-CAALFM_C301700WA
gene-CAALFM_C405280WA
gene-CAALFM_C102020WA
gene-CAALFM_CR06470WA
gene-CAALFM_CR02560CA
gene-CAALFM_C202790CA
gene-CAALFM_C113410WA
gene-CAALFM_C403390WA
gene-CAALFM_C503330CA
gene-CAALFM_C302700WA
gene-CAALFM_C601190CA
gene-CAALFM_C102320CA
gene-CAALFM_C112530CA
gene-CAALFM_CR06790CA
gene-CAALFM_CR05720WA
gene-CAALFM_CR03210CA
gene-CAALFM_C302580CA
gene-CAALFM_C207080CA
gene-CAALFM_C100500CA
gene-CAALFM_C109330WA
gene-CAALFM_C110280CA
gene-CAALFM_C602470WA
gene-CAALFM_C201160WA
gene-CAALFM_C305530WA
gene-CAALFM_C107400CA
gene-CAALFM_C102220CA
gene-CAALFM_C403780CA

| | |
|---|---|
| gene-CAALFM_C305580CA | gene-CAALFM_C401550CA |
| gene-CAALFM_C400100CA | gene-CAALFM_C202180WA |
| gene-CAALFM_C300760WA | gene-CAALFM_C110800CA |
| gene-CAALFM_C404780WA | gene-CAALFM_C200480CA |
| gene-CAALFM_CR10680WA | gene-CAALFM_C404140WA |
| gene-CAALFM_CR09090CA | gene-CAALFM_C210340WA |
| gene-CAALFM_C404090CA | gene-CAALFM_C111600WA |
| gene-CAALFM_C107100CA | gene-CAALFM_C306940WA |
| gene-CAALFM_C602250WA | gene-CAALFM_C204410WA |
| gene-CAALFM_C603820CA | gene-CAALFM_C301130CA |
| gene-CAALFM_C101770WA | gene-CAALFM_C112490WA |
| gene-CAALFM_C204960CA | gene-CAALFM_C406440CA |
| gene-CAALFM_CR09550CA | gene-CAALFM_C402110WA |
| gene-CAALFM_C700690WA | gene-CAALFM_C600720CA |
| gene-CAALFM_C302400CA | gene-CAALFM_C703390CA |
| gene-CAALFM_C500890CA | gene-CAALFM_C401670CA |
| gene-CAALFM_C208460CA | gene-CAALFM_C305550CA |
| gene-CAALFM_C201440CA | gene-CAALFM_CR09100CA |
| gene-CAALFM_C111600WA | gene-CAALFM_C602850WA |
| gene-CAALFM_C113760WA | gene-CAALFM_C603370WA |
| gene-CAALFM_CR06050WA | gene-CAALFM_C405320WA |
| gene-CAALFM_C113130CA | gene-CAALFM_C102590CA |
| gene-CAALFM_C112670CA | gene-CAALFM_C503920CA |
| gene-CAALFM_C500730WA | gene-CAALFM_C400440CA |
| gene-CAALFM_C401140CA | gene-CAALFM_C601180CA |
| gene-CAALFM_C603300CA | gene-CAALFM_C601420CA |
| gene-CAALFM_CR07370WA | gene-CAALFM_C210840WA |
| gene-CAALFM_C401860CA | gene-CAALFM_C110530WA |
| gene-CAALFM_C201660CA | gene-CAALFM_CR09780CA |
| gene-CAALFM_C109800CA | gene-CAALFM_CR03720WA |
| gene-CAALFM_C101140CA | gene-CAALFM_C205550WA |
| gene-CAALFM_C113630WA | gene-CAALFM_C209710CA |
| gene-CAALFM_C402120WA | gene-CAALFM_C100310WA |
| gene-CAALFM_C108460CA | gene-CAALFM_C206700WA |
| gene-CAALFM_CR01080WA | gene-CAALFM_C101680CA |
| gene-CAALFM_C405430CA | gene-CAALFM_CR05440WA |
| gene-CAALFM_C306010WA | gene-CAALFM_C703760WA |
| gene-CAALFM_C402530WA | gene-CAALFM_CR06340CA |
| gene-CAALFM_C302600CA | gene-CAALFM_C701750WA |
| gene-CAALFM_C402110WA | gene-CAALFM_C301080WA |
| gene-CAALFM_C111620WA | gene-CAALFM_C210170CA |
| gene-CAALFM_CR09290WA | gene-CAALFM_C204960CA |
| gene-CAALFM_CR00540CA | gene-CAALFM_C405210WA |
| gene-CAALFM_C301810CA | gene-CAALFM_C701990CA |

gene-CAALFM_C111100WA
gene-CAALFM_C301700WA
gene-CAALFM_C114130WA
gene-CAALFM_C109490CA
gene-CAALFM_C208570WA
gene-CAALFM_C405070CA
gene-CAALFM_C703840WA
gene-CAALFM_C102170CA
gene-CAALFM_C210200WA
gene-CAALFM_C406440CA
gene-CAALFM_C209710CA
gene-CAALFM_C209070CA
gene-CAALFM_C205850CA
gene-CAALFM_CR05140WA
gene-CAALFM_C302580CA
gene-CAALFM_C401370WA
gene-CAALFM_C604030WA
gene-CAALFM_C604410CA
gene-CAALFM_CR00470WA
gene-CAALFM_C110830WA
gene-CAALFM_C201930CA
gene-CAALFM_C500690CA
gene-CAALFM_CR05440WA
gene-CAALFM_C301400WA
gene-CAALFM_C405210WA
gene-CAALFM_C503840WA
gene-CAALFM_C603540WA
gene-CAALFM_C103960CA
gene-CAALFM_C700090CA
gene-CAALFM_CR09420CA
gene-CAALFM_C407040WA
gene-CAALFM_C307180CA
gene-CAALFM_C301300CA
gene-CAALFM_C502580WA
gene-CAALFM_C406340WA
gene-CAALFM_C302700WA
gene-CAALFM_C100710CA
gene-CAALFM_C401780CA
gene-CAALFM_C105950CA
gene-CAALFM_C306920WA
gene-CAALFM_C306990WA
gene-CAALFM_CR09480WA
gene-CAALFM_C102490CA
gene-CAALFM_C114580CA

gene-CAALFM_C401530CA
gene-CAALFM_C206800CA
gene-CAALFM_C703020CA
gene-CAALFM_C500690CA
gene-CAALFM_C700100WA
gene-CAALFM_CR09340WA
gene-CAALFM_C703810WA
gene-CAALFM_C604370WA
gene-CAALFM_C403830WA
gene-CAALFM_C209980WA
gene-CAALFM_CR09270CA
gene-CAALFM_C101640WA
gene-CAALFM_C700700WA
gene-CAALFM_CR07130CA
gene-CAALFM_C207930CA
gene-CAALFM_C100580WA
gene-CAALFM_C205460WA
gene-CAALFM_C703870WA
gene-CAALFM_CR04810WA
gene-CAALFM_C701400CA
gene-CAALFM_C700720WA
gene-CAALFM_C405820WA
gene-CAALFM_C306560WA
gene-CAALFM_C113760WA
gene-CAALFM_CR05140WA
gene-CAALFM_C305040CA
gene-CAALFM_C300390WA
gene-CAALFM_C203700WA
gene-CAALFM_C701330CA
gene-CAALFM_C402550CA
gene-CAALFM_C204980CA
gene-CAALFM_C203870WA
gene-CAALFM_C602180WA
gene-CAALFM_C103490WA
gene-CAALFM_C108500CA
gene-CAALFM_C702570CA
gene-CAALFM_C601750CA
gene-CAALFM_C202320CA
gene-CAALFM_C203010CA
gene-CAALFM_C603610WA
gene-CAALFM_C110650WA
gene-CAALFM_C108070WA
gene-CAALFM_C112240CA
gene-CAALFM_C300400CA

gene-CAALFM_C107120WA
gene-CAALFM_C206550WA
gene-CAALFM_C210360CA
gene-CAALFM_C306550CA
gene-CAALFM_C604110WA
gene-CAALFM_C114190CA
gene-CAALFM_C701560CA
gene-CAALFM_C111470CA
gene-CAALFM_C406770WA
gene-CAALFM_C504960WA
gene-CAALFM_C301880WA
gene-CAALFM_C602160WA
gene-CAALFM_C108320WA
gene-CAALFM_C401320CA
gene-CAALFM_C300220WA
gene-CAALFM_C106440CA
gene-CAALFM_C103750WA
gene-CAALFM_CR08430WA
gene-CAALFM_C700930WA
gene-CAALFM_C201000WA
gene-CAALFM_C100700WA
gene-CAALFM_CR08090WA
gene-CAALFM_C602470WA
gene-CAALFM_C208630CA
gene-CAALFM_C112630CA
gene-CAALFM_C402070WA
gene-CAALFM_C305860CA
gene-CAALFM_C100790WA
gene-CAALFM_C600960WA
gene-CAALFM_CR01300WA
gene-CAALFM_CR07490CA
gene-CAALFM_C105760CA
gene-CAALFM_C106360WA
gene-CAALFM_C703310WA
gene-CAALFM_C600250WA
gene-CAALFM_C304030CA
gene-CAALFM_C206600WA
gene-CAALFM_C400440CA
gene-CAALFM_C403900CA
gene-CAALFM_CR03690WA
gene-CAALFM_C702390WA
gene-CAALFM_C700010CA
gene-CAALFM_C404210CA
gene-CAALFM_C111670WA

gene-CAALFM_C210350CA
gene-CAALFM_C702010CA
gene-CAALFM_C602030CA
gene-CAALFM_C703520WA
gene-CAALFM_C503290CA
gene-CAALFM_C104210CA
gene-CAALFM_C401730CA
gene-CAALFM_C603820CA
gene-CAALFM_C603590CA
gene-CAALFM_C301470WA
gene-CAALFM_C108030WA
gene-CAALFM_C503880CA
gene-CAALFM_CR00090CA
gene-CAALFM_C402470CA
gene-CAALFM_C602410WA
gene-CAALFM_C101780CA
gene-CAALFM_C111750WA
gene-CAALFM_C103820WA
gene-CAALFM_C207220WA
gene-CAALFM_C206680WA
gene-CAALFM_C203880CA
gene-CAALFM_C701160CA
gene-CAALFM_C203170WA
gene-CAALFM_C402360WA
gene-CAALFM_CR10130WA
gene-CAALFM_C701700WA
gene-CAALFM_C112200WA
gene-CAALFM_C401230CA
gene-CAALFM_C301030WA
gene-CAALFM_CR03270WA
gene-CAALFM_CR08920WA
gene-CAALFM_C501100CA
gene-CAALFM_C106440CA
gene-CAALFM_C404100CA
gene-CAALFM_C106010WA
gene-CAALFM_C305510WA
gene-CAALFM_CR09290WA
gene-CAALFM_C405550CA
gene-CAALFM_C108320WA
gene-CAALFM_C101710WA
gene-CAALFM_C402400CA
gene-CAALFM_CR03260WA
gene-CAALFM_CR09530CA
gene-CAALFM_C111980WA

gene-CAALFM_C100170WA
gene-CAALFM_C112840WA
gene-CAALFM_CR02680WA
gene-CAALFM_C203260WA
gene-CAALFM_C208680WA
gene-CAALFM_C301030WA
gene-CAALFM_C403790WA
gene-CAALFM_C702650WA
gene-CAALFM_C405440CA
gene-CAALFM_CR02820WA
gene-CAALFM_C204650CA
gene-CAALFM_C205230CA
gene-CAALFM_CR03600CA
gene-CAALFM_CR10180WA
gene-CAALFM_C502540CA
gene-CAALFM_C110800CA
gene-CAALFM_C109220WA
gene-CAALFM_CR06150CA
gene-CAALFM_CR01560WA
gene-CAALFM_C402880CA
gene-CAALFM_C306590WA
gene-CAALFM_C601340CA
gene-CAALFM_C401770WA
gene-CAALFM_C500550CA
gene-CAALFM_C401290WA
gene-CAALFM_C206430CA
gene-CAALFM_C104160CA
gene-CAALFM_C104000CA
gene-CAALFM_C403330WA
gene-CAALFM_CR05160CA
gene-CAALFM_C305530WA
gene-CAALFM_CR05130CA
gene-CAALFM_CR09500CA
gene-CAALFM_C106800WA
gene-CAALFM_C201720CA
gene-CAALFM_C600440CA
gene-CAALFM_C401510WA
gene-CAALFM_C112200WA
gene-CAALFM_CR06240CA
gene-CAALFM_CR00580WA
gene-CAALFM_C209480WA
gene-CAALFM_C603230WA
gene-CAALFM_C203270WA
gene-CAALFM_C108500CA
gene-CAALFM_C603540WA
gene-CAALFM_C702330WA
gene-CAALFM_CR01260WA
gene-CAALFM_C111880WA
gene-CAALFM_C114570CA
gene-CAALFM_C701350CA
gene-CAALFM_CR09420CA
gene-CAALFM_C500450CA
gene-CAALFM_C105440CA
gene-CAALFM_C201580WA
gene-CAALFM_C500480CA
gene-CAALFM_C307180CA
gene-CAALFM_C206670CA
gene-CAALFM_C301190CA
gene-CAALFM_C402120WA
gene-CAALFM_C504930CA
gene-CAALFM_C202310WA
gene-CAALFM_C602980CA
gene-CAALFM_CR10390WA
gene-CAALFM_C406460CA
gene-CAALFM_C105410CA
gene-CAALFM_CR01930CA
gene-CAALFM_C305280CA
gene-CAALFM_C200250WA
gene-CAALFM_C503120WA
gene-CAALFM_CR04060CA
gene-CAALFM_C503480CA
gene-CAALFM_CR00580WA
gene-CAALFM_C109800CA
gene-CAALFM_C703720CA
gene-CAALFM_CR06900CA
gene-CAALFM_C402200CA
gene-CAALFM_C303510CA
gene-CAALFM_C406340WA
gene-CAALFM_C403800CA
gene-CAALFM_C110510WA
gene-CAALFM_C300210CA
gene-CAALFM_CR06460WA
gene-CAALFM_C114580CA
gene-CAALFM_C205180WA
gene-CAALFM_C104020CA
gene-CAALFM_C107120WA
gene-CAALFM_C101840CA
gene-CAALFM_C306600CA

gene-CAALFM_C113310WA
gene-CAALFM_C105000WA
gene-CAALFM_C301710CA
gene-CAALFM_C601900CA
gene-CAALFM_C305270CA
gene-CAALFM_C600720CA
gene-CAALFM_C403770WA
gene-CAALFM_C307440WA
gene-CAALFM_C306910CA
gene-CAALFM_C305790CA
gene-CAALFM_CR02020CA
gene-CAALFM_C206760CA
gene-CAALFM_C405390WA
gene-CAALFM_C110820CA
gene-CAALFM_C604240WA
gene-CAALFM_C109040CA
gene-CAALFM_C111990WA
gene-CAALFM_C403830WA
gene-CAALFM_C703780CA
gene-CAALFM_C109720WA
gene-CAALFM_CR04650WA
gene-CAALFM_C502920WA
gene-CAALFM_C405320WA
gene-CAALFM_C111480WA
gene-CAALFM_C402400CA
gene-CAALFM_C113530WA
gene-CAALFM_C701800CA
gene-CAALFM_C503300CA
gene-CAALFM_C406880CA
gene-CAALFM_C112270WA
gene-CAALFM_CR09120CA
gene-CAALFM_C200770WA
gene-CAALFM_C500640CA
gene-CAALFM_C601300WA
gene-CAALFM_C401750CA
gene-CAALFM_C303490WA
gene-CAALFM_C601390WA
gene-CAALFM_C306950WA
gene-CAALFM_C303800WA
gene-CAALFM_C702690CA
gene-CAALFM_CR06560CA
gene-CAALFM_C403880WA
gene-CAALFM_C306870WA
gene-CAALFM_C209840WA
gene-CAALFM_CR04950WA
gene-CAALFM_C109000WA
gene-CAALFM_CR09120CA
gene-CAALFM_CR09710WA
gene-CAALFM_C206880CA
gene-CAALFM_C701440WA
gene-CAALFM_C208620WA
gene-CAALFM_C103510CA
gene-CAALFM_C206720WA
gene-CAALFM_C113120CA
gene-CAALFM_C208490WA
gene-CAALFM_CR03820CA
gene-CAALFM_C202410WA
gene-CAALFM_C113390WA
gene-CAALFM_C111620WA
gene-CAALFM_C208630CA
gene-CAALFM_C301390CA
gene-CAALFM_C600250WA
gene-CAALFM_C201190CA
gene-CAALFM_C206850WA
gene-CAALFM_C405920CA
gene-CAALFM_C701470CA
gene-CAALFM_C305730CA
gene-CAALFM_C701480WA
gene-CAALFM_C105520WA
gene-CAALFM_C205440WA
gene-CAALFM_C300030CA
gene-CAALFM_C704310CA
gene-CAALFM_C307440WA
gene-CAALFM_C603280WA
gene-CAALFM_C401370WA
gene-CAALFM_C505050WA
gene-CAALFM_CR10350CA
gene-CAALFM_C105000WA
gene-CAALFM_C402510WA
gene-CAALFM_C304630WA
gene-CAALFM_CR04280CA
gene-CAALFM_C210480WA
gene-CAALFM_C112260WA
gene-CAALFM_C201310WA
gene-CAALFM_C302210CA
gene-CAALFM_C305850WA
gene-CAALFM_C502160WA
gene-CAALFM_CR07830CA

| | |
|---|---|
| gene-CAALFM_C603270CA | gene-CAALFM_CR02650CA |
| gene-CAALFM_C301080WA | gene-CAALFM_C403730CA |
| gene-CAALFM_C101620CA | gene-CAALFM_C404160WA |
| gene-CAALFM_C204750WA | gene-CAALFM_C302590WA |
| gene-CAALFM_C202210CA | gene-CAALFM_C206940CA |
| gene-CAALFM_CR01400WA | gene-CAALFM_C500390CA |
| gene-CAALFM_C107510WA | gene-CAALFM_C110820CA |
| gene-CAALFM_C405940WA | gene-CAALFM_C101220CA |
| gene-CAALFM_CR09610CA | gene-CAALFM_CR03040CA |
| gene-CAALFM_C301470WA | gene-CAALFM_CR07480WA |
| gene-CAALFM_C102260CA | gene-CAALFM_C600930CA |
| gene-CAALFM_C208490WA | gene-CAALFM_C402070WA |
| gene-CAALFM_C104700CA | gene-CAALFM_C105930CA |
| gene-CAALFM_C107500CA | gene-CAALFM_C405450CA |
| gene-CAALFM_C201910WA | gene-CAALFM_C600990WA |
| gene-CAALFM_CR06900CA | gene-CAALFM_CR09750CA |
| gene-CAALFM_C402330CA | gene-CAALFM_C603040CA |
| gene-CAALFM_C208510WA | gene-CAALFM_CR05030WA |
| gene-CAALFM_C209180WA | gene-CAALFM_C210050WA |
| gene-CAALFM_C406490CA | gene-CAALFM_C105990CA |
| gene-CAALFM_CR02800CA | gene-CAALFM_C703860WA |
| gene-CAALFM_C203250WA | gene-CAALFM_C108330CA |
| gene-CAALFM_C502530WA | gene-CAALFM_C209480WA |
| gene-CAALFM_C702640WA | gene-CAALFM_CR08840CA |
| gene-CAALFM_CR08840CA | gene-CAALFM_CR09140CA |
| gene-CAALFM_C101750WA | gene-CAALFM_C110290WA |
| gene-CAALFM_C305390CA | gene-CAALFM_C209390WA |
| gene-CAALFM_C201900CA | gene-CAALFM_C302150CA |
| gene-CAALFM_C403230CA | gene-CAALFM_C100710CA |
| gene-CAALFM_C603320WA | gene-CAALFM_C100690WA |
| gene-CAALFM_C406570CA | gene-CAALFM_CR03850WA |
| gene-CAALFM_C300390WA | gene-CAALFM_C205570CA |
| gene-CAALFM_C304060CA | gene-CAALFM_C210210CA |
| gene-CAALFM_C305740CA | gene-CAALFM_CR05860WA |
| gene-CAALFM_C201820CA | gene-CAALFM_C600830CA |
| gene-CAALFM_C306860CA | gene-CAALFM_C301930WA |
| gene-CAALFM_C208560WA | gene-CAALFM_CR02520WA |
| gene-CAALFM_C501590WA | gene-CAALFM_C406010CA |
| gene-CAALFM_CR03270WA | gene-CAALFM_C405220CA |
| gene-CAALFM_C603240WA | gene-CAALFM_CR01040CA |
| gene-CAALFM_C209050CA | gene-CAALFM_C307080WA |
| gene-CAALFM_C300810CA | gene-CAALFM_C107840WA |
| gene-CAALFM_C502520WA | gene-CAALFM_C401380WA |
| gene-CAALFM_C108920WA | gene-CAALFM_C109770WA |

| | |
|---|---|
| gene-CAALFM_C201190CA | gene-CAALFM_C503370CA |
| gene-CAALFM_C602420WA | gene-CAALFM_C504940WA |
| gene-CAALFM_C208620WA | gene-CAALFM_CR08890CA |
| gene-CAALFM_C600840WA | gene-CAALFM_C702520WA |
| gene-CAALFM_CR06340CA | gene-CAALFM_C100850WA |
| gene-CAALFM_C111950WA | gene-CAALFM_C206430CA |
| gene-CAALFM_C109930WA | gene-CAALFM_C401560CA |
| gene-CAALFM_C102860CA | gene-CAALFM_C110560CA |
| gene-CAALFM_C103510CA | gene-CAALFM_C300930WA |
| gene-CAALFM_C201580WA | gene-CAALFM_C112670CA |
| gene-CAALFM_C302680CA | gene-CAALFM_C403900CA |
| gene-CAALFM_CR04790WA | gene-CAALFM_C111890WA |
| gene-CAALFM_C301410CA | gene-CAALFM_C101750WA |
| gene-CAALFM_C406590WA | gene-CAALFM_C703490WA |
| gene-CAALFM_C100770CA | gene-CAALFM_C102530CA |
| gene-CAALFM_C207930CA | gene-CAALFM_C109220WA |
| gene-CAALFM_C201040WA | gene-CAALFM_C201720CA |
| gene-CAALFM_CR01260WA | gene-CAALFM_C101760WA |
| gene-CAALFM_C107700CA | gene-CAALFM_C307760CA |
| gene-CAALFM_C100830WA | gene-CAALFM_C108260CA |
| gene-CAALFM_C203230CA | gene-CAALFM_C400100CA |
| gene-CAALFM_C112580WA | gene-CAALFM_C114030WA |
| gene-CAALFM_C304100WA | gene-CAALFM_C203230CA |
| gene-CAALFM_C401530CA | gene-CAALFM_C602160WA |
| gene-CAALFM_C503280WA | gene-CAALFM_CR07570WA |
| gene-CAALFM_C700790WA | gene-CAALFM_C400110CA |
| gene-CAALFM_CR06640CA | gene-CAALFM_C107510WA |
| gene-CAALFM_C103990WA | gene-CAALFM_C403790WA |
| gene-CAALFM_CR08670CA | gene-CAALFM_C601670WA |
| gene-CAALFM_C108590CA | gene-CAALFM_C112780WA |
| gene-CAALFM_C111790WA | gene-CAALFM_C405910CA |
| gene-CAALFM_CR04480CA | gene-CAALFM_C403840CA |
| gene-CAALFM_CR02350CA | gene-CAALFM_CR01300WA |
| gene-CAALFM_C304510WA | gene-CAALFM_C302690CA |
| gene-CAALFM_C301090WA | gene-CAALFM_C400120WA |
| gene-CAALFM_C104740WA | gene-CAALFM_C405420CA |
| gene-CAALFM_C201230WA | gene-CAALFM_C301410CA |
| gene-CAALFM_CR02370WA | gene-CAALFM_C110330CA |
| gene-CAALFM_C702670WA | gene-CAALFM_C604180WA |
| gene-CAALFM_C505050WA | gene-CAALFM_C302680CA |
| gene-CAALFM_C113670WA | gene-CAALFM_C403370CA |
| gene-CAALFM_C206030WA | gene-CAALFM_CR00210WA |
| gene-CAALFM_CR07150WA | gene-CAALFM_C206240WA |
| gene-CAALFM_C203890WA | gene-CAALFM_C204660CA |

gene-CAALFM_CR06620WA
gene-CAALFM_C703260CA
gene-CAALFM_C601370WA
gene-CAALFM_C703530CA
gene-CAALFM_C701370WA
gene-CAALFM_C204970WA
gene-CAALFM_C106120CA
gene-CAALFM_CR05080WA
gene-CAALFM_C101360CA
gene-CAALFM_C602770WA
gene-CAALFM_C402760CA
gene-CAALFM_C112830CA
gene-CAALFM_C702680WA
gene-CAALFM_C405230CA
gene-CAALFM_C102250WA
gene-CAALFM_C503480CA
gene-CAALFM_C210300CA
gene-CAALFM_CR01550CA
gene-CAALFM_CR03260WA
gene-CAALFM_C306540CA
gene-CAALFM_C702500CA
gene-CAALFM_C503370CA
gene-CAALFM_C305340WA
gene-CAALFM_C401460CA
gene-CAALFM_C400500WA
gene-CAALFM_C307130WA
gene-CAALFM_CR10110WA
gene-CAALFM_C108890CA
gene-CAALFM_C103910CA
gene-CAALFM_C404180CA
gene-CAALFM_C104310CA
gene-CAALFM_C303080WA
gene-CAALFM_C102530CA
gene-CAALFM_C205460WA
gene-CAALFM_C305850WA
gene-CAALFM_C107690CA
gene-CAALFM_C601990WA
gene-CAALFM_C701390WA
gene-CAALFM_C603420WA
gene-CAALFM_C204460WA
gene-CAALFM_CR02520WA
gene-CAALFM_CR09470WA
gene-CAALFM_C404800WA
gene-CAALFM_C404100CA

gene-CAALFM_C604110WA
gene-CAALFM_C302170CA
gene-CAALFM_C113670WA
gene-CAALFM_CR02240CA
gene-CAALFM_C503280WA
gene-CAALFM_C103960CA
gene-CAALFM_C604430WA
gene-CAALFM_C502520WA
gene-CAALFM_C102860CA
gene-CAALFM_C100350CA
gene-CAALFM_C305270CA
gene-CAALFM_C102110CA
gene-CAALFM_CR06640CA
gene-CAALFM_CR05130CA
gene-CAALFM_C203290WA
gene-CAALFM_C200860CA
gene-CAALFM_C203270WA
gene-CAALFM_C210200WA
gene-CAALFM_C102440CA
gene-CAALFM_C602370CA
gene-CAALFM_C403920WA
gene-CAALFM_C604240WA
gene-CAALFM_CR01280CA
gene-CAALFM_C400680WA
gene-CAALFM_C300810CA
gene-CAALFM_C500470CA
gene-CAALFM_C500640CA
gene-CAALFM_C104680WA
gene-CAALFM_C201660CA
gene-CAALFM_C204830WA
gene-CAALFM_C205850CA
gene-CAALFM_CR01190CA
gene-CAALFM_C405870CA
gene-CAALFM_CR10840CA
gene-CAALFM_CR05160CA
gene-CAALFM_C600760WA
gene-CAALFM_C403050CA
gene-CAALFM_C405730WA
gene-CAALFM_C210360CA
gene-CAALFM_C402880CA
gene-CAALFM_C601650CA
gene-CAALFM_C204460WA
gene-CAALFM_C202600CA
gene-CAALFM_C114530WA

gene-CAALFM_C601560WA
gene-CAALFM_CR02220CA
gene-CAALFM_C204110WA
gene-CAALFM_C400110CA
gene-CAALFM_CR01010WA
gene-CAALFM_CR09660WA
gene-CAALFM_CR01050CA
gene-CAALFM_C504310WA
gene-CAALFM_C403370CA
gene-CAALFM_C406460CA
gene-CAALFM_C402700WA
gene-CAALFM_C105010CA
gene-CAALFM_C700920CA
gene-CAALFM_C208840WA
gene-CAALFM_C703650WA
gene-CAALFM_CR06160CA
gene-CAALFM_C103900WA
gene-CAALFM_C104710CA
gene-CAALFM_C401800WA
gene-CAALFM_C210690WA
gene-CAALFM_CR06920WA
gene-CAALFM_C207570WA
gene-CAALFM_C305180WA
gene-CAALFM_C102330CA
gene-CAALFM_C402410CA
gene-CAALFM_C109990WA
gene-CAALFM_C201300CA
gene-CAALFM_C301430WA
gene-CAALFM_C601470WA
gene-CAALFM_C107540CA
gene-CAALFM_C700680WA
gene-CAALFM_C302390WA
gene-CAALFM_C502930CA
gene-CAALFM_C102500WA
gene-CAALFM_C102290CA
gene-CAALFM_C205700WA
gene-CAALFM_C208930WA
gene-CAALFM_C204530WA
gene-CAALFM_C108070WA
gene-CAALFM_C208190WA
gene-CAALFM_C106480CA
gene-CAALFM_C604170CA
gene-CAALFM_C201730WA
gene-CAALFM_C603340CA

gene-CAALFM_C205350CA
gene-CAALFM_C304060CA
gene-CAALFM_C503950WA
gene-CAALFM_CR02370WA
gene-CAALFM_C104340CA
gene-CAALFM_CR00470WA
gene-CAALFM_C303400CA
gene-CAALFM_C603630WA
gene-CAALFM_C501710CA
gene-CAALFM_C100700WA
gene-CAALFM_C404230WA
gene-CAALFM_CR04210CA
gene-CAALFM_C306920WA
gene-CAALFM_C405400CA
gene-CAALFM_C103930WA
gene-CAALFM_C201920CA
gene-CAALFM_C402740WA
gene-CAALFM_C604300WA
gene-CAALFM_C100290WA
gene-CAALFM_C105540CA
gene-CAALFM_C402330CA
gene-CAALFM_CR07160CA
gene-CAALFM_C104700CA
gene-CAALFM_C703680WA
gene-CAALFM_CR08690CA
gene-CAALFM_C204560WA
gene-CAALFM_C105770CA
gene-CAALFM_C205820WA
gene-CAALFM_C113530WA
gene-CAALFM_C300920WA
gene-CAALFM_C700460WA
gene-CAALFM_C100830WA
gene-CAALFM_C403490CA
gene-CAALFM_C110630CA
gene-CAALFM_C210100WA
gene-CAALFM_C204650CA
gene-CAALFM_C103800WA
gene-CAALFM_C503110CA
gene-CAALFM_C105950CA
gene-CAALFM_C102360CA
gene-CAALFM_C210300CA
gene-CAALFM_C109400CA
gene-CAALFM_C208390WA
gene-CAALFM_C112630CA

gene-CAALFM_CR01470WA
gene-CAALFM_CR04210CA
gene-CAALFM_C305770CA
gene-CAALFM_C113650CA
gene-CAALFM_C108880WA
gene-CAALFM_CR02240CA
gene-CAALFM_C106210WA
gene-CAALFM_C504990WA
gene-CAALFM_C400950CA
gene-CAALFM_C601020WA
gene-CAALFM_C503740WA
gene-CAALFM_C703790WA
gene-CAALFM_CR04800WA
gene-CAALFM_C106040WA
gene-CAALFM_C401200CA
gene-CAALFM_C113080WA
gene-CAALFM_CR04960CA
gene-CAALFM_C405300WA
gene-CAALFM_C302620CA
gene-CAALFM_C403600CA
gene-CAALFM_C306800CA
gene-CAALFM_CR02490WA
gene-CAALFM_CR09600CA
gene-CAALFM_C304960WA
gene-CAALFM_C703380WA
gene-CAALFM_C301120WA
gene-CAALFM_C112520WA
gene-CAALFM_C504910WA
gene-CAALFM_C201090CA
gene-CAALFM_C702660CA
gene-CAALFM_CR02690WA
gene-CAALFM_C305300CA
gene-CAALFM_C401970WA
gene-CAALFM_C114570CA
gene-CAALFM_C401690CA
gene-CAALFM_C604400WA
gene-CAALFM_C202860WA
gene-CAALFM_C703280CA
gene-CAALFM_C307120WA
gene-CAALFM_C702510WA
gene-CAALFM_CR05020WA
gene-CAALFM_C112370WA
gene-CAALFM_C204320WA
gene-CAALFM_C206650CA

gene-CAALFM_C209950WA
gene-CAALFM_C106070WA
gene-CAALFM_C502560CA
gene-CAALFM_C301880WA
gene-CAALFM_C703600WA
gene-CAALFM_C405340WA
gene-CAALFM_C703380WA
gene-CAALFM_CR06050WA
gene-CAALFM_CR00420WA
gene-CAALFM_C501590WA
gene-CAALFM_C204720CA
gene-CAALFM_C107700CA
gene-CAALFM_CR09090CA
gene-CAALFM_C101810CA
gene-CAALFM_C604410CA
gene-CAALFM_C306860CA
gene-CAALFM_C101670CA
gene-CAALFM_C601900CA
gene-CAALFM_C600960WA
gene-CAALFM_C104000CA
gene-CAALFM_C504910WA
gene-CAALFM_C501450WA
gene-CAALFM_C202330WA
gene-CAALFM_C600620WA
gene-CAALFM_C503300CA
gene-CAALFM_C306910CA
gene-CAALFM_C201570WA
gene-CAALFM_C402340WA
gene-CAALFM_C302430WA
gene-CAALFM_C107570CA
gene-CAALFM_C700680WA
gene-CAALFM_C500710WA
gene-CAALFM_C205700WA
gene-CAALFM_C107500CA
gene-CAALFM_C702690CA
gene-CAALFM_C500580WA
gene-CAALFM_C405800CA
gene-CAALFM_C207800WA
gene-CAALFM_C205450CA
gene-CAALFM_CR07810WA
gene-CAALFM_C302400CA
gene-CAALFM_C302600CA
gene-CAALFM_C112310CA
gene-CAALFM_C701830WA

gene-CAALFM_C601060CA
gene-CAALFM_C106060CA
gene-CAALFM_C503970WA
gene-CAALFM_C601120CA
gene-CAALFM_C205400WA
gene-CAALFM_C112480WA
gene-CAALFM_CR09010CA
gene-CAALFM_C112640WA
gene-CAALFM_C405890WA
gene-CAALFM_C307220CA
gene-CAALFM_C204700CA
gene-CAALFM_C405800CA
gene-CAALFM_C208590WA
gene-CAALFM_C702310CA
gene-CAALFM_C112760WA
gene-CAALFM_C107330WA
gene-CAALFM_C503110CA
gene-CAALFM_CR06940WA
gene-CAALFM_C306970WA
gene-CAALFM_CR06950CA
gene-CAALFM_C402670WA
gene-CAALFM_C101610CA
gene-CAALFM_C205250CA
gene-CAALFM_C107600WA
gene-CAALFM_C700850WA
gene-CAALFM_C107950CA
gene-CAALFM_C601130WA
gene-CAALFM_C209950WA
gene-CAALFM_C401490WA
gene-CAALFM_C603400CA
gene-CAALFM_C208130WA
gene-CAALFM_C305540CA
gene-CAALFM_C113710CA
gene-CAALFM_CR04770CA
gene-CAALFM_C404230WA
gene-CAALFM_C205070WA
gene-CAALFM_C105960WA
gene-CAALFM_CR04780WA
gene-CAALFM_CR02070CA
gene-CAALFM_C102410CA
gene-CAALFM_C503320CA
gene-CAALFM_C700750WA
gene-CAALFM_CR02540WA
gene-CAALFM_CR02600WA

gene-CAALFM_C106060CA
gene-CAALFM_C503840WA
gene-CAALFM_CR05170CA
gene-CAALFM_C200510WA
gene-CAALFM_CR09550CA
gene-CAALFM_C107590CA
gene-CAALFM_C305470WA
gene-CAALFM_C502540CA
gene-CAALFM_C102400CA
gene-CAALFM_C404800WA
gene-CAALFM_C602680WA
gene-CAALFM_C602770WA
gene-CAALFM_C108770WA
gene-CAALFM_C210690WA
gene-CAALFM_C500430WA
gene-CAALFM_C405090CA
gene-CAALFM_C700170WA
gene-CAALFM_C604190CA
gene-CAALFM_C206870CA
gene-CAALFM_CR07490CA
gene-CAALFM_C204700CA
gene-CAALFM_C405940WA
gene-CAALFM_C207140WA
gene-CAALFM_CR02960WA
gene-CAALFM_C200760CA
gene-CAALFM_C113870WA
gene-CAALFM_C201150WA
gene-CAALFM_C703310WA
gene-CAALFM_C105710CA
gene-CAALFM_C208100WA
gene-CAALFM_C102490CA
gene-CAALFM_C603360CA
gene-CAALFM_C111470CA
gene-CAALFM_C703530CA
gene-CAALFM_C114180WA
gene-CAALFM_C403770WA
gene-CAALFM_CR10210WA
gene-CAALFM_CR00540CA
gene-CAALFM_C305860CA
gene-CAALFM_C101620CA
gene-CAALFM_C603230WA
gene-CAALFM_CR04650WA
gene-CAALFM_C210180WA
gene-CAALFM_C201230WA

gene-CAALFM_CR00780CA
gene-CAALFM_C209820WA
gene-CAALFM_CR09810WA
gene-CAALFM_C101950CA
gene-CAALFM_CR05340CA
gene-CAALFM_C302330CA
gene-CAALFM_C301110CA
gene-CAALFM_C100860WA
gene-CAALFM_CR09840CA
gene-CAALFM_CR05310WA
gene-CAALFM_C603210CA
gene-CAALFM_C402450WA
gene-CAALFM_C104200CA
gene-CAALFM_C104120CA
gene-CAALFM_CR00310CA
gene-CAALFM_C205290CA
gene-CAALFM_CR04410WA
gene-CAALFM_C301140WA
gene-CAALFM_C112310CA
gene-CAALFM_CR01180WA
gene-CAALFM_C107930CA
gene-CAALFM_C400120WA
gene-CAALFM_C201740CA
gene-CAALFM_CR05290WA
gene-CAALFM_C112800WA
gene-CAALFM_C204370WA
gene-CAALFM_C306880WA
gene-CAALFM_C307050WA
gene-CAALFM_C303380WA
gene-CAALFM_C702610CA
gene-CAALFM_CR06170WA
gene-CAALFM_C301220WA
gene-CAALFM_CR09490WA
gene-CAALFM_C103800WA
gene-CAALFM_C307210WA
gene-CAALFM_C104960CA
gene-CAALFM_CR09360WA
gene-CAALFM_C111910WA
gene-CAALFM_C304610WA
gene-CAALFM_C703800WA
gene-CAALFM_C108980CA
gene-CAALFM_C601110WA
gene-CAALFM_C101280CA
gene-CAALFM_C209830CA
gene-CAALFM_C404090CA
gene-CAALFM_C109930WA
gene-CAALFM_C403810WA
gene-CAALFM_C502530WA
gene-CAALFM_C201900CA
gene-CAALFM_C602250WA
gene-CAALFM_C104710CA
gene-CAALFM_C102980WA
gene-CAALFM_C604030WA
gene-CAALFM_C204520CA
gene-CAALFM_CR05940WA
gene-CAALFM_C112760WA
gene-CAALFM_C702640WA
gene-CAALFM_C104970WA
gene-CAALFM_CR01270CA
gene-CAALFM_C204640CA
gene-CAALFM_C301400WA
gene-CAALFM_C204750WA
gene-CAALFM_CR01050CA
gene-CAALFM_C206650CA
gene-CAALFM_CR09660WA
gene-CAALFM_C208840WA
gene-CAALFM_C400200CA
gene-CAALFM_C403890WA
gene-CAALFM_C100770CA
gene-CAALFM_C502920WA
gene-CAALFM_C112580WA
gene-CAALFM_C402670WA
gene-CAALFM_C305230WA
gene-CAALFM_C208680WA
gene-CAALFM_C101360CA
gene-CAALFM_C102730WA
gene-CAALFM_C700930WA
gene-CAALFM_C405300WA
gene-CAALFM_CR09480WA
gene-CAALFM_C503930CA
gene-CAALFM_CR01560WA
gene-CAALFM_C203260WA
gene-CAALFM_CR02350CA
gene-CAALFM_C302270WA
gene-CAALFM_C205400WA
gene-CAALFM_C305390CA
gene-CAALFM_C114190CA
gene-CAALFM_C204530WA

| | |
|---|---|
| gene-CAALFM_C102380CA | gene-CAALFM_C109070WA |
| gene-CAALFM_C602530CA | gene-CAALFM_CR07150WA |
| gene-CAALFM_CR04910WA | gene-CAALFM_C407040WA |
| gene-CAALFM_CR02700WA | gene-CAALFM_C502990WA |
| gene-CAALFM_C206280CA | gene-CAALFM_C401510WA |
| gene-CAALFM_C113540WA | gene-CAALFM_C102260CA |
| gene-CAALFM_C201020WA | gene-CAALFM_C302610CA |
| gene-CAALFM_C113270WA | gene-CAALFM_C202210CA |
| gene-CAALFM_C701710WA | gene-CAALFM_C603850CA |
| gene-CAALFM_C305330CA | gene-CAALFM_C601390WA |
| gene-CAALFM_C106080CA | gene-CAALFM_C112690CA |
| gene-CAALFM_CR09510CA | gene-CAALFM_C112840WA |
| gene-CAALFM_C500660CA | gene-CAALFM_C107330WA |
| gene-CAALFM_CR01060WA | gene-CAALFM_C405230CA |
| gene-CAALFM_C113510CA | gene-CAALFM_C108880WA |
| gene-CAALFM_C112540WA | gene-CAALFM_C306590WA |
| gene-CAALFM_CR06960WA | gene-CAALFM_C404770CA |
| gene-CAALFM_C301450CA | gene-CAALFM_CR04880WA |
| gene-CAALFM_CR09070CA | gene-CAALFM_C603300CA |
| gene-CAALFM_C402100CA | gene-CAALFM_C102330CA |
| gene-CAALFM_C206850WA | gene-CAALFM_C303080WA |
| gene-CAALFM_C210140WA | gene-CAALFM_C307030CA |
| gene-CAALFM_C208050CA | gene-CAALFM_C702560WA |
| gene-CAALFM_C208530CA | gene-CAALFM_C502910CA |
| gene-CAALFM_C402420CA | gene-CAALFM_C208200WA |
| gene-CAALFM_CR02920CA | gene-CAALFM_C502690WA |
| gene-CAALFM_C703450CA | gene-CAALFM_C201820CA |
| gene-CAALFM_C105530CA | gene-CAALFM_C702610CA |
| gene-CAALFM_C112000CA | gene-CAALFM_CR10180WA |
| gene-CAALFM_C400130WA | gene-CAALFM_C601450CA |
| gene-CAALFM_C701300CA | gene-CAALFM_C501620CA |
| gene-CAALFM_C201830WA | gene-CAALFM_C703630CA |
| gene-CAALFM_C301070CA | gene-CAALFM_C208460CA |
| gene-CAALFM_C401310WA | gene-CAALFM_C207570WA |
| gene-CAALFM_C100820WA | gene-CAALFM_C102170CA |
| gene-CAALFM_C104070CA | gene-CAALFM_C204480WA |
| gene-CAALFM_C208610WA | gene-CAALFM_C209070CA |
| gene-CAALFM_C112850WA | gene-CAALFM_CR09810WA |
| gene-CAALFM_C401250WA | gene-CAALFM_C203890WA |
| gene-CAALFM_C405310WA | gene-CAALFM_C307220CA |
| gene-CAALFM_CR09620CA | gene-CAALFM_C406880CA |
| gene-CAALFM_C201860CA | gene-CAALFM_C402320CA |
| gene-CAALFM_C206660WA | gene-CAALFM_C208930WA |
| gene-CAALFM_C302420CA | gene-CAALFM_C304030CA |

| | |
|---|---|
| gene-CAALFM_C112650CA | gene-CAALFM_CR09580CA |
| gene-CAALFM_C603330CA | gene-CAALFM_C702390WA |
| gene-CAALFM_C603360CA | gene-CAALFM_C208360CA |
| gene-CAALFM_C104320WA | gene-CAALFM_C208050CA |
| gene-CAALFM_CR00600CA | gene-CAALFM_C102470WA |
| gene-CAALFM_C113370WA | gene-CAALFM_CR07110CA |
| gene-CAALFM_CR06970CA | gene-CAALFM_C113480WA |
| gene-CAALFM_CR04750WA | gene-CAALFM_C201450CA |
| gene-CAALFM_CR01070WA | gene-CAALFM_C504310WA |
| gene-CAALFM_C204730WA | gene-CAALFM_C106360WA |
| gene-CAALFM_CR06570CA | gene-CAALFM_C401460CA |
| gene-CAALFM_C208540CA | gene-CAALFM_C503970WA |
| gene-CAALFM_C406450WA | gene-CAALFM_C100170WA |
| gene-CAALFM_C604380WA | gene-CAALFM_C107830CA |
| gene-CAALFM_C112660WA | gene-CAALFM_C106040WA |
| gene-CAALFM_C206790WA | gene-CAALFM_C401800WA |
| gene-CAALFM_CR06430WA | gene-CAALFM_C204280WA |
| gene-CAALFM_C604310WA | gene-CAALFM_CR00610WA |
| gene-CAALFM_CR02630CA | gene-CAALFM_C202860WA |
| gene-CAALFM_C401760WA | gene-CAALFM_C603400CA |
| gene-CAALFM_C601360WA | gene-CAALFM_C502080CA |
| gene-CAALFM_C701500WA | gene-CAALFM_C502110WA |
| gene-CAALFM_CR06520CA | gene-CAALFM_C601990WA |
| gene-CAALFM_CR06980WA | gene-CAALFM_CR04830CA |
| gene-CAALFM_C208690CA | gene-CAALFM_C208600WA |
| gene-CAALFM_C106030CA | gene-CAALFM_C106800WA |
| gene-CAALFM_C305450CA | gene-CAALFM_C301710CA |
| gene-CAALFM_C602550WA | gene-CAALFM_C101770WA |
| gene-CAALFM_CR09460CA | gene-CAALFM_CR05750WA |
| gene-CAALFM_C401680WA | gene-CAALFM_C113470WA |
| gene-CAALFM_C210120WA | gene-CAALFM_C600980CA |
| gene-CAALFM_C104280CA | gene-CAALFM_C209820WA |
| gene-CAALFM_CR09720WA | gene-CAALFM_C203250WA |
| gene-CAALFM_C210060CA | gene-CAALFM_CR01080WA |
| gene-CAALFM_C701740CA | gene-CAALFM_C108920WA |
| gene-CAALFM_C104770CA | gene-CAALFM_C702680WA |
| gene-CAALFM_CR09570WA | gene-CAALFM_C400790CA |
| gene-CAALFM_CR06500CA | gene-CAALFM_C301300CA |
| gene-CAALFM_C301150CA | gene-CAALFM_C206340WA |
| gene-CAALFM_C206240WA | gene-CAALFM_C208590WA |
| gene-CAALFM_C300990CA | gene-CAALFM_C401140CA |
| gene-CAALFM_C110320WA | gene-CAALFM_C601300WA |
| gene-CAALFM_C204270WA | gene-CAALFM_C106210WA |
| gene-CAALFM_C107900WA | gene-CAALFM_C406450WA |

| | |
|---|---|
| gene-CAALFM_C502980CA | gene-CAALFM_C107100CA |
| gene-CAALFM_C703830CA | gene-CAALFM_CR05080WA |
| gene-CAALFM_CR00920WA | gene-CAALFM_CR00780CA |
| gene-CAALFM_C104150CA | gene-CAALFM_C209830CA |
| gene-CAALFM_C604340WA | gene-CAALFM_CR10790WA |
| gene-CAALFM_C112280CA | gene-CAALFM_C304960WA |
| gene-CAALFM_C109060CA | gene-CAALFM_C601130WA |
| gene-CAALFM_C204580WA | gene-CAALFM_C113160WA |
| gene-CAALFM_C102480WA | gene-CAALFM_C602890CA |
| gene-CAALFM_C206770WA | gene-CAALFM_C402700WA |
| gene-CAALFM_C701290WA | gene-CAALFM_C206030WA |
| gene-CAALFM_C201320WA | gene-CAALFM_C305770CA |
| gene-CAALFM_CR04890WA | gene-CAALFM_C306990WA |
| gene-CAALFM_C400450CA | gene-CAALFM_C202060CA |
| gene-CAALFM_C405380CA | gene-CAALFM_C401320CA |
| gene-CAALFM_C106390WA | gene-CAALFM_C602530CA |
| gene-CAALFM_C105510CA | gene-CAALFM_C200770WA |
| gene-CAALFM_C107660WA | gene-CAALFM_C603320WA |
| gene-CAALFM_C104110WA | gene-CAALFM_C207630CA |
| gene-CAALFM_C500620WA | gene-CAALFM_C404180CA |
| gene-CAALFM_C208290CA | gene-CAALFM_C703650WA |
| gene-CAALFM_C103980WA | gene-CAALFM_CR02820WA |
| gene-CAALFM_C401430CA | gene-CAALFM_C405390WA |
| gene-CAALFM_C206690CA | gene-CAALFM_CR06970CA |
| gene-CAALFM_CR09350CA | gene-CAALFM_CR02780WA |
| gene-CAALFM_C206370CA | gene-CAALFM_C101950CA |
| gene-CAALFM_C111800CA | gene-CAALFM_CR04780WA |
| gene-CAALFM_C102300WA | gene-CAALFM_C307200CA |
| gene-CAALFM_C109110WA | gene-CAALFM_C112830CA |
| gene-CAALFM_C402460WA | gene-CAALFM_C111100WA |
| gene-CAALFM_CR09590WA | gene-CAALFM_C603270CA |
| gene-CAALFM_C301540WA | gene-CAALFM_C206040CA |
| gene-CAALFM_C601380CA | gene-CAALFM_C300990CA |
| gene-CAALFM_C112730WA | gene-CAALFM_C703930CA |
| gene-CAALFM_C113290WA | gene-CAALFM_CR04750WA |
| gene-CAALFM_C208750WA | gene-CAALFM_C113910CA |
| gene-CAALFM_CR02900WA | gene-CAALFM_C501420WA |
| gene-CAALFM_C102520WA | gene-CAALFM_C103900WA |
| gene-CAALFM_C703570WA | gene-CAALFM_C301040CA |
| gene-CAALFM_C700760CA | gene-CAALFM_C406590WA |
| gene-CAALFM_CR06590CA | gene-CAALFM_CR02920CA |
| gene-CAALFM_C301040CA | gene-CAALFM_C111670WA |
| gene-CAALFM_C602620CA | gene-CAALFM_C601370WA |
| gene-CAALFM_C702290WA | gene-CAALFM_C109990WA |

gene-CAALFM_C404950WA
gene-CAALFM_C301440CA
gene-CAALFM_C109940WA
gene-CAALFM_C204740CA
gene-CAALFM_C303810WA
gene-CAALFM_C300230CA
gene-CAALFM_C403870CA
gene-CAALFM_C601480WA
gene-CAALFM_C106870CA
gene-CAALFM_C208710WA
gene-CAALFM_CR05330WA
gene-CAALFM_C303140CA
gene-CAALFM_C405020WA
gene-CAALFM_C302270WA
gene-CAALFM_C206620WA
gene-CAALFM_C403430WA
gene-CAALFM_CR06650CA
gene-CAALFM_CR04860CA
gene-CAALFM_C105190CA
gene-CAALFM_C703820CA
gene-CAALFM_CR02380CA
gene-CAALFM_CR04850CA
gene-CAALFM_C601140CA
gene-CAALFM_C700400WA

represents common genes

gene-CAALFM_C401200CA
gene-CAALFM_C603880WA
gene-CAALFM_CR04790WA
gene-CAALFM_C704070CA
gene-CAALFM_C700750WA
gene-CAALFM_C402530WA
gene-CAALFM_C602550WA
gene-CAALFM_C305740CA
gene-CAALFM_C109980CA
gene-CAALFM_C106480CA
gene-CAALFM_C401250WA
gene-CAALFM_C204330CA
gene-CAALFM_C304100WA
gene-CAALFM_C303120CA
gene-CAALFM_CR06560CA
gene-CAALFM_CR02800CA
gene-CAALFM_C306790WA
gene-CAALFM_C209180WA
gene-CAALFM_C406410WA
gene-CAALFM_C107950CA
gene-CAALFM_C300220WA
gene-CAALFM_C702310CA
gene-CAALFM_C702220CA
gene-CAALFM_CR04770CA
gene-CAALFM_CR09360WA
gene-CAALFM_CR02020CA
gene-CAALFM_C305580CA
gene-CAALFM_CR09500CA
gene-CAALFM_C105760CA
gene-CAALFM_C104960CA
gene-CAALFM_C604170CA
gene-CAALFM_C109310CA
gene-CAALFM_CR09190CA
gene-CAALFM_C204010CA
gene-CAALFM_C105960WA
gene-CAALFM_C703260CA
gene-CAALFM_C107690CA
gene-CAALFM_C113650CA
gene-CAALFM_C405130CA
gene-CAALFM_C208190WA
gene-CAALFM_C202970CA
gene-CAALFM_CR09470WA
gene-CAALFM_CR04760CA
gene-CAALFM_C201300CA

gene-CAALFM_C104200CA
gene-CAALFM_C301120WA
gene-CAALFM_C104740WA
gene-CAALFM_C108890CA
gene-CAALFM_C111790WA
gene-CAALFM_C206710WA
gene-CAALFM_C201650WA
gene-CAALFM_C305180WA
gene-CAALFM_C604380WA
gene-CAALFM_C403330WA
gene-CAALFM_C405160CA
gene-CAALFM_C603210CA
gene-CAALFM_C306970WA
gene-CAALFM_C208560WA
gene-CAALFM_C703790WA
gene-CAALFM_C701370WA
gene-CAALFM_CR09460CA
gene-CAALFM_C405890WA
gene-CAALFM_C107930CA
gene-CAALFM_C101980WA
gene-CAALFM_C104160CA
gene-CAALFM_C402760CA
gene-CAALFM_C601400WA
gene-CAALFM_C601560WA
gene-CAALFM_C402520CA
gene-CAALFM_C204370WA
gene-CAALFM_C102410CA
gene-CAALFM_C111320CA
gene-CAALFM_C112140WA
gene-CAALFM_C601470WA
gene-CAALFM_C105010CA
gene-CAALFM_C201090CA
gene-CAALFM_C107600WA
gene-CAALFM_C306800CA
gene-CAALFM_C201930CA
gene-CAALFM_C205250CA
gene-CAALFM_C403230CA
gene-CAALFM_C104800CA
gene-CAALFM_C400450CA
gene-CAALFM_C302420CA
gene-CAALFM_C602200CA
gene-CAALFM_C603420WA
gene-CAALFM_C104140WA
gene-CAALFM_C106030CA

gene-CAALFM_C503060CA
gene-CAALFM_C403600CA
gene-CAALFM_C601020WA
gene-CAALFM_C209050CA
gene-CAALFM_C602320CA
gene-CAALFM_C206370CA
gene-CAALFM_C401970WA
gene-CAALFM_C601060CA
gene-CAALFM_C701390WA
gene-CAALFM_C602950CA
gene-CAALFM_C112640WA
gene-CAALFM_CR06940WA
gene-CAALFM_C204320WA
gene-CAALFM_C101610CA
gene-CAALFM_C105190CA
gene-CAALFM_C301140WA
gene-CAALFM_CR01550CA
gene-CAALFM_C112540WA
gene-CAALFM_CR04800WA
gene-CAALFM_C400080CA
gene-CAALFM_C700920CA
gene-CAALFM_CR06620WA
gene-CAALFM_C400500WA
gene-CAALFM_C401490WA
gene-CAALFM_C402100CA
gene-CAALFM_C205290CA
gene-CAALFM_C702650WA
gene-CAALFM_C306950WA
gene-CAALFM_C503740WA
gene-CAALFM_C603340CA
gene-CAALFM_CR04910WA
gene-CAALFM_C112520WA
gene-CAALFM_C401310WA
gene-CAALFM_C400130WA
gene-CAALFM_C305300CA
gene-CAALFM_C702500CA
gene-CAALFM_C404030WA
gene-CAALFM_C500660CA
gene-CAALFM_C102290CA
gene-CAALFM_C703800WA
gene-CAALFM_CR09010CA
gene-CAALFM_C103990WA
gene-CAALFM_C501670WA
gene-CAALFM_CR02540WA

gene-CAALFM_C208740WA
gene-CAALFM_C305340WA
gene-CAALFM_C700790WA
gene-CAALFM_C307210WA
gene-CAALFM_CR06430WA
gene-CAALFM_CR06520CA
gene-CAALFM_C402210WA
gene-CAALFM_C301320CA
gene-CAALFM_CR06920WA
gene-CAALFM_CR04480CA
gene-CAALFM_C302620CA
gene-CAALFM_C210140WA
gene-CAALFM_CR09490WA
gene-CAALFM_C104320WA
gene-CAALFM_CR09720WA
gene-CAALFM_CR06960WA
gene-CAALFM_C406620CA
gene-CAALFM_C701740CA
gene-CAALFM_C701710WA
gene-CAALFM_C204970WA
gene-CAALFM_CR09610CA
gene-CAALFM_CR02700WA
gene-CAALFM_C102500WA
gene-CAALFM_CR02690WA
gene-CAALFM_C401760WA
gene-CAALFM_C306880WA
gene-CAALFM_CR04960CA
gene-CAALFM_C603240WA
gene-CAALFM_C208510WA
gene-CAALFM_C702510WA
gene-CAALFM_C112370WA
gene-CAALFM_CR05340CA
gene-CAALFM_C405070CA
gene-CAALFM_C104070CA
gene-CAALFM_C503320CA
gene-CAALFM_CR09600CA
gene-CAALFM_C601110WA
gene-CAALFM_CR00920WA
gene-CAALFM_C113270WA
gene-CAALFM_CR08670CA
gene-CAALFM_CR01180WA
gene-CAALFM_C301450CA
gene-CAALFM_C113710CA
gene-CAALFM_C202130CA

gene-CAALFM_C102250WA
gene-CAALFM_C703780CA
gene-CAALFM_C403870CA
gene-CAALFM_CR00310CA
gene-CAALFM_C206600WA
gene-CAALFM_CR06170WA
gene-CAALFM_C301220WA
gene-CAALFM_C600840WA
gene-CAALFM_C100860WA
gene-CAALFM_C113950CA
gene-CAALFM_C208610WA
gene-CAALFM_C201860CA
gene-CAALFM_CR09840CA
gene-CAALFM_C201740CA
gene-CAALFM_CR09510CA
gene-CAALFM_C304610WA
gene-CAALFM_C307130WA
gene-CAALFM_CR05290WA
gene-CAALFM_C102300WA
gene-CAALFM_C402450WA
gene-CAALFM_C401290WA
gene-CAALFM_C303380WA
gene-CAALFM_C104770CA
gene-CAALFM_C102380CA
gene-CAALFM_CR10120CA
gene-CAALFM_CR02070CA
gene-CAALFM_CR09560CA
gene-CAALFM_C101740WA
gene-CAALFM_C500550CA
gene-CAALFM_C502930CA
gene-CAALFM_C204270WA
gene-CAALFM_C104310CA
gene-CAALFM_C201830WA
gene-CAALFM_C301150CA
gene-CAALFM_CR01070WA
gene-CAALFM_C112110CA
gene-CAALFM_C113510CA
gene-CAALFM_C700850WA
gene-CAALFM_CR01470WA
gene-CAALFM_C702670WA
gene-CAALFM_C107900WA
gene-CAALFM_C113370WA
gene-CAALFM_C703450CA
gene-CAALFM_C208330WA

gene-CAALFM_CR06980WA
gene-CAALFM_C700280WA
gene-CAALFM_CR02490WA
gene-CAALFM_CR02220CA
gene-CAALFM_C401090CA
gene-CAALFM_C108980CA
gene-CAALFM_C402410CA
gene-CAALFM_C201020WA
gene-CAALFM_C602620CA
gene-CAALFM_C112000CA
gene-CAALFM_C701300CA
gene-CAALFM_C604310WA
gene-CAALFM_C401170CA
gene-CAALFM_CR09620CA
gene-CAALFM_C204110WA
gene-CAALFM_C401690CA
gene-CAALFM_CR05020WA
gene-CAALFM_C401680WA
gene-CAALFM_C504990WA
gene-CAALFM_C104150CA
gene-CAALFM_C502980CA
gene-CAALFM_C208130WA
gene-CAALFM_CR01060WA
gene-CAALFM_C601070CA
gene-CAALFM_C113600WA
gene-CAALFM_C110320WA
gene-CAALFM_C400950CA
gene-CAALFM_C111800CA
gene-CAALFM_C107480CA
gene-CAALFM_C702660CA
gene-CAALFM_C208530CA
gene-CAALFM_C405310WA
gene-CAALFM_C305330CA
gene-CAALFM_C104120CA
gene-CAALFM_C210120WA
gene-CAALFM_C106080CA
gene-CAALFM_C603330CA
gene-CAALFM_CR05310WA
gene-CAALFM_C206660WA
gene-CAALFM_C306540CA
gene-CAALFM_C206770WA
gene-CAALFM_C701500WA
gene-CAALFM_C302330CA
gene-CAALFM_C402420CA

gene-CAALFM_C601380CA
gene-CAALFM_C703280CA
gene-CAALFM_C208170WA
gene-CAALFM_C305790CA
gene-CAALFM_C105530CA
gene-CAALFM_C602420WA
gene-CAALFM_C109060CA
gene-CAALFM_C112480WA
gene-CAALFM_C701290WA
gene-CAALFM_C112660WA
gene-CAALFM_C111950WA
gene-CAALFM_CR09570WA
gene-CAALFM_C301820WA
gene-CAALFM_CR06570CA
gene-CAALFM_C305450CA
gene-CAALFM_C403820CA
gene-CAALFM_C209840WA
gene-CAALFM_CR04410WA
gene-CAALFM_CR06500CA
gene-CAALFM_C201730WA
gene-CAALFM_C102580WA
gene-CAALFM_C500620WA
gene-CAALFM_C208540CA
gene-CAALFM_C304510WA
gene-CAALFM_C103910CA
gene-CAALFM_C113540WA
gene-CAALFM_C103980WA
gene-CAALFM_C206690CA
gene-CAALFM_C101280CA
gene-CAALFM_C301070CA
gene-CAALFM_C601360WA
gene-CAALFM_C109740CA
gene-CAALFM_CR09220CA
gene-CAALFM_C106390WA
gene-CAALFM_C604340WA
gene-CAALFM_C703830CA
gene-CAALFM_C204730WA
gene-CAALFM_CR06650CA
gene-CAALFM_C204580WA
gene-CAALFM_C106120CA
gene-CAALFM_C403430WA
gene-CAALFM_CR00600CA
gene-CAALFM_C204740CA
gene-CAALFM_C102480WA

| |
|---|
| gene-CAALFM_C405020WA |
| gene-CAALFM_C402460WA |
| gene-CAALFM_C105510CA |
| gene-CAALFM_CR02380CA |
| gene-CAALFM_C208290CA |
| gene-CAALFM_C112650CA |
| gene-CAALFM_C401430CA |
| gene-CAALFM_C305620WA |
| gene-CAALFM_CR09070CA |
| gene-CAALFM_C201320WA |
| gene-CAALFM_C302390WA |
| gene-CAALFM_C210060CA |
| gene-CAALFM_CR09350CA |
| gene-CAALFM_C111910WA |
| gene-CAALFM_C601120CA |
| gene-CAALFM_C112850WA |
| gene-CAALFM_C104110WA |
| gene-CAALFM_C307120WA |
| gene-CAALFM_CR09590WA |
| gene-CAALFM_C206790WA |
| gene-CAALFM_C100820WA |
| gene-CAALFM_C703820CA |
| gene-CAALFM_CR04860CA |
| gene-CAALFM_C301540WA |
| gene-CAALFM_C104280CA |
| gene-CAALFM_C112280CA |
| gene-CAALFM_CR02630CA |
| gene-CAALFM_CR04890WA |
| gene-CAALFM_C703550CA |
| gene-CAALFM_C604070CA |
| gene-CAALFM_C402220CA |
| gene-CAALFM_C400530CA |
| gene-CAALFM_C202070WA |
| gene-CAALFM_C109700WA |
| gene-CAALFM_C107640CA |
| gene-CAALFM_C601680CA |
| gene-CAALFM_C208750WA |
| gene-CAALFM_CR07840CA |
| gene-CAALFM_C703570WA |
| gene-CAALFM_C403760WA |
| gene-CAALFM_CR01830CA |
| gene-CAALFM_C302250CA |
| gene-CAALFM_CR07140CA |
| gene-CAALFM_C301440CA |

| |
|---|
| gene-CAALFM_C103840WA |
| gene-CAALFM_CR06590CA |
| gene-CAALFM_C601480WA |
| gene-CAALFM_C109110WA |
| gene-CAALFM_C702290WA |
| gene-CAALFM_C503270WA |
| gene-CAALFM_C107660WA |
| gene-CAALFM_C301110CA |
| gene-CAALFM_C109940WA |
| gene-CAALFM_C404950WA |
| gene-CAALFM_C208690CA |
| gene-CAALFM_C209910CA |
| gene-CAALFM_C503910CA |
| gene-CAALFM_C601940WA |
| gene-CAALFM_C603570WA |
| gene-CAALFM_C303140CA |
| gene-CAALFM_CR02600WA |
| gene-CAALFM_C206620WA |
| gene-CAALFM_C405380CA |
| gene-CAALFM_C307910WA |
| gene-CAALFM_C307050WA |
| gene-CAALFM_C604400WA |
| gene-CAALFM_CR04850CA |
| gene-CAALFM_C208710WA |
| gene-CAALFM_C112730WA |
| gene-CAALFM_C601140CA |
| gene-CAALFM_C700400WA |
